# A Generalized Approach for Distal C–H Arylation of Organic Building Blocks: Unveiling the Role of Counter Anion

**DOI:** 10.1002/advs.202519731

**Published:** 2025-12-25

**Authors:** Jagrit Grover, Gaurav Prakash, Astam Mandal, Devika Ghosh, Siddhartha Maiti, Claire Empel, Debabrata Maiti

**Affiliations:** ^1^ Department of Chemistry Indian Institute of Technology Bombay Mumbai India; ^2^ School of Biosciences, Engineering and Technology VIT Bhopal University Kothrikalan Sehore Madhya Pradesh India; ^3^ Institute of Organic Chemistry RWTH Aachen University Aachen Germany

**Keywords:** C–H activation, C–H *arylation*, computational studies, *meta*‐C–H functionalization, Pd‐catalysis

## Abstract

Aryl–aryl coupling at remote C–H sites remain a formidable challenge in organic synthesis, particularly for electronically biased arenes and heteroarenes. Herein, we report a generalized and versatile protocol for *meta‐selective* C–H arylation using readily available aryl iodides as coupling partners. This strategy overcomes key limitations of prior methods such as reliance on boronic acids and limited substrate scope by employing a Pd(II)/Pd(IV) catalytic cycle assisted by a removable meta‐directing group. Importantly, DFT studies revealed that the counter anion critically influences selectivity: trifluoroacetate reduces the activation energy for meta‐C–H activation and thereby improving selectivity. This protocol is applicable to a wide array of functional groups and scaffolds, including phenylacetic acids, anilines, phenols, indolines, and pharmaceutical derivatives like Naproxen and Ketoprofen. Moreover, mechanistic investigations via isotope labeling and kinetic studies provide valuable insights into the reaction pathway. Post‐arylation transformations, including directing group removal, further underscore the synthetic utility of this method. Overall, this work introduces a practical, mechanistically guided platform for remote C–H arylation, with broad implications for medicinal chemistry and complex molecule construction.

## Graphical abstract

We report a general strategy for *meta*‐selective C–H arylation using aryl iodides and a Pd(II)/Pd(IV) catalytic system guided by a removable directing group. DFT and experimental studies reveal that counter anion tuning (acetate to trifluoroacetate) significantly enhances selectivity. Broad substrate scope and application to pharmaceutically relevant molecules are demonstrated.

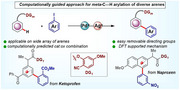

## 1 Introduction

Biaryl scaffolds are characterized by the presence of two aromatic rings directly linked by a carbon‐carbon bond, are prevalent in numerous natural products and drug molecules. These biaryl motifs exhibit a diverse range of biological activities, making their efficient synthesis a topic of significant interest in synthetic chemistry [1, 2]. Traditionally, cross‐coupling reactions have served as the workhorse for biaryl construction. Among these, the Suzuki‐Miyaura cross‐coupling reaction stands out as a cornerstone method, extensively employed by academic and industrial researchers alike [3, 4, 5, 6, 7, 8, 9]. Notably, a study revealed that Suzuki coupling ranks as the second most frequently utilized chemical transformation in medicinal chemistry for the synthesis of various drug molecules [10]. In recent years, transition metal‐catalyzed C–H arylation has emerged as a powerful alternative for the construction of biaryl motifs [11, 12, 13, 14, 15, 16, 17, 18, 19, 20, 21, 22, 23]. This approach elegantly bypasses the need for pre‐functionalized arenes, offering a more streamlined and atom‐economical strategy compared to traditional cross‐coupling methods. However, to distinguish between almost identical C–H bonds, or control over the site of C─H bond activation, has remained a significant challenge in C–H functionalization. The advent of directing group‐assisted transition‐metal‐catalyzed C–H functionalization has significantly improved regioselectivity in these reactions [24, 25, 26, 27, 28, 29, 30, 31, 32, 33]. By incorporating a directing group onto the substrate molecule, it becomes feasible to direct the metal catalyst toward a specific C─H bond, leading to the desired product. The atom economy and broad applicability of C–H arylation have propelled it to the forefront of biaryl synthesis methodologies. In most directing‐group‐assisted C–H activation processes, functionalization occurs predominantly at the *ortho*‐position, as the *metal*‐center readily forms a stable cyclometalated intermediate; in contrast, achieving meta‐selective activation is considerably more challenging due to the greater distance from the coordinating site.

**SCHEME 1:**
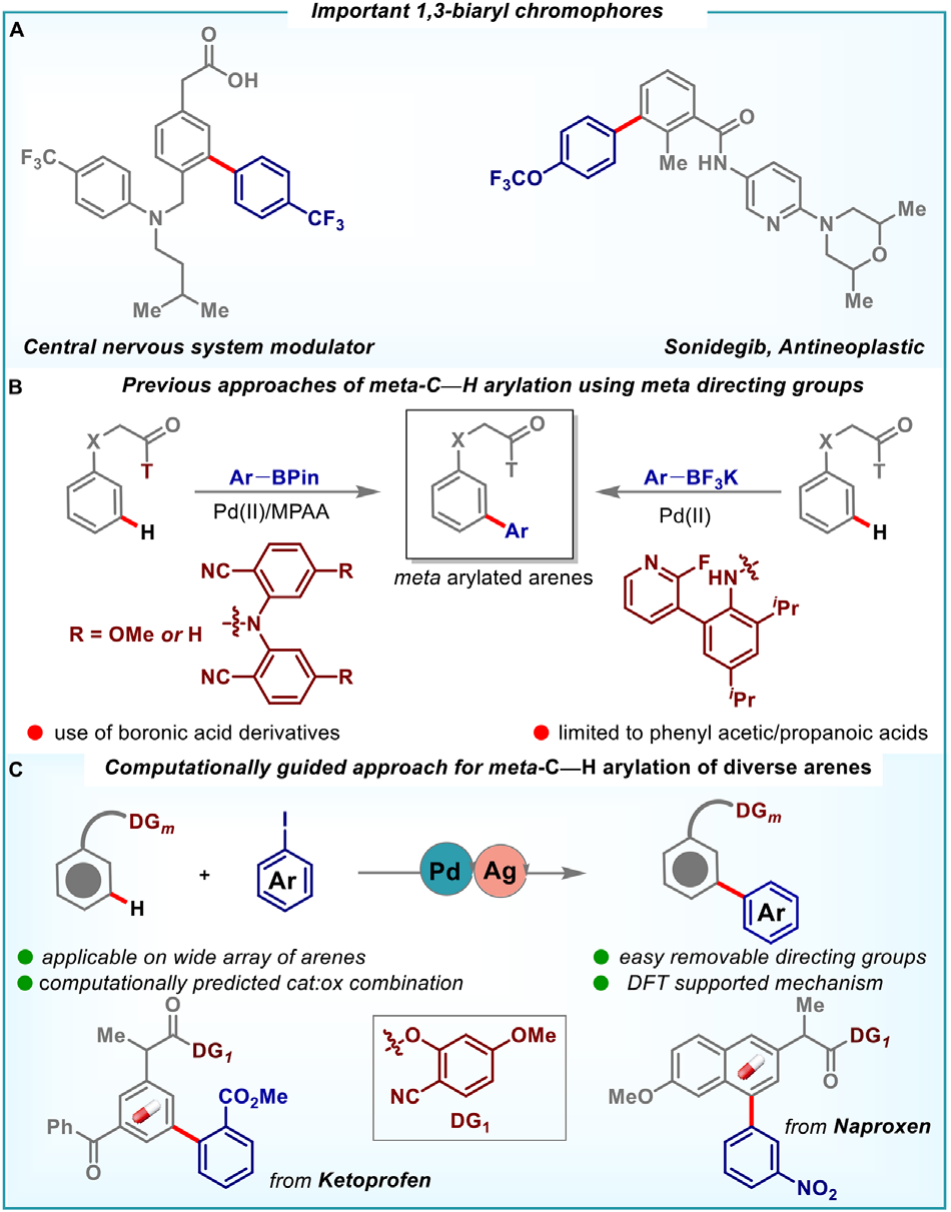
(A) Important 1,3‐biaryl motifs. (B) State of the art of *meta*‐C−H arylation using *meta*‐directing groups. (C) This work: Generalized approach for *meta*‐selective C−H arylation of diverse arenes and heteroarenes.

However, in 2009, Gaunt and co‐workers made a significant breakthrough in *meta*‐C–H arylation by employing hypervalent iodine salts in conjunction with copper catalysis. This transformation proceeded via a dearomative oxy‐cupration mechanism that facilitated selective *meta*‐C–H functionalization [34]. In 2013, the Yu group disclosed the first example of cross‐coupling at *meta*‐C─H bond with organoboron compounds using a removable U‐shaped nitrile based directing template (Scheme 1B) [35]. Although this method represented a significant advancement, its applicability was majorly limited to phenylpropanoic acids. Subsequently, Yu et al. demonstrated the *meta*‐C–H arylation of indolines and phenyl acetic acids, employing a pyridine‐based template (Scheme 1B) [36, 37].

While previous studies have predominantly relied on boronic acid derivatives as aryl sources, our approach provides a complementary alternative based on aryl iodides, enabling broader substrate applicability and improved versatility [38, 39, 40, 41].

**SCHEME 2:**
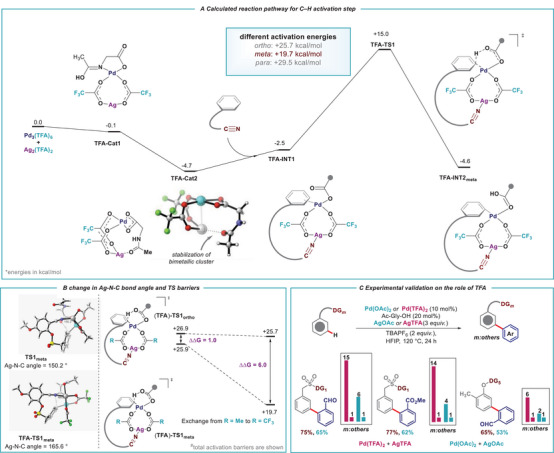
(A) Gibbs energy profile for the Pd(TFA)_2_/AgTFA combination for *meta*‐C(*sp^2^*)–H activation. (B) Changes in geometry and activation energy for different counter ions. (C) Experimental validation.

Other approaches to achieve distal *meta*‐C–H arylation were demonstrated by the same group through the Catellani reaction, which employs norbornene as a transient mediator. This method undoubtedly represents a significant advancement in the field of *meta*‐C–H activation [42, 43, 44, 45, 46, 47, 48, 49]. Unfortunately, achieving selective mono‐C–H functionalization in Catellani‐type reactions often necessitates the pre‐blocking of *ortho*‐ or *meta*‐positions to suppress undesired multiple substitutions. Notably, the elegant studies by Fernández‐Ibáñez and co‐workers have demonstrated the general feasibility of selective mono *meta*‐C–H arylation even in the absence of *ortho*‐ or *meta*‐substituents [46], providing valuable insights into the design principles governing site selectivity. Their contributions highlight how the careful choice of catalysts and ligands can finely balance steric and electronic factors to enable high *meta*‐selectivity without the aid of directing groups or positional blocking (Scheme 2).

Nevertheless, in many cases, *meta*‐selectivity continues to depend strongly on substrate electronics, directing group effects, or steric modulation around the reactive site. A key limitation in the field is the lack of a general method for distal C–H arylation that bypasses the use of aryl boronates and enables activation of *meta*‐C–H bonds in substrates such as anilines and phenols, which inherently favor *ortho*‐ and *para*‐functionalization due to their electronic properties. To overcome these limitations, we envisioned developing a general approach for *meta‐*C–H arylation that leverages aryl iodides as the coupling partner and is applicable on a wide variety of substrates. However, implementation of aryl iodides also comes with several synthetic challenges such as dehydrohalogenation and catalytic poisoning by accumulation of free iodide ions [50]. These issues need to be tackled to develop a generalized approach for distal C–H arylation using aryl iodides as aryl surrogates. By addressing the limitations of previous methodologies and capitalizing on the advantages of aryl iodides, this work aims to advance the field of *meta*‐C–H arylation. This approach provides a robust and versatile tool for the synthesis of 1,3‐biaryl compounds, thereby expanding the toolbox of synthetic organic chemistry (Scheme 1C).

## 2 Reaction Development

**SCHEME 3:**
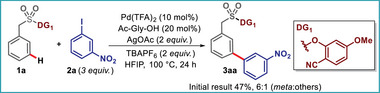
Initial trial for *meta*‐C−H arylation.

We selected substrate **1a** with easily removable, low molecular weight, and commercially accessible 2‐cyano‐4‐methoxy phenol as the directing group to establish a robust technique for *meta*‐C–H arylation. The initial reaction of **1a** with 1‐iodo‐3‐nitrobenzene **2a** as the aryl coupling partner in the presence of Pd(OAc)_2_, *N*‐Ac‐glycine as an ancillary ligand, and AgOAc as an oxidant furnished the desired product **3aa** in 47% yield with 6:1 (*meta:others*) selectivity (Scheme 3).

Intrigued by this finding, we performed rapid optimization of various reaction parameters including different palladium sources, ancillary ligands, bases, oxidants, and temperatures to further increase yield and selectivity (for details, please see Supporting Information). These efforts resulted in an improvement in the yield and selectivity of the desired product; however, overall, the *meta*‐selectivity remained moderate. Consequently, it remained challenging to establish a highly selective meta‐C–H arylation method. To further improve on our reaction, we turned our attention toward experimental and computational studies to get an in‐depth understanding of the reaction mechanism to fine‐tune our protocol.

### 2.1 Mechanistic Investigations

**SCHEME 4:**
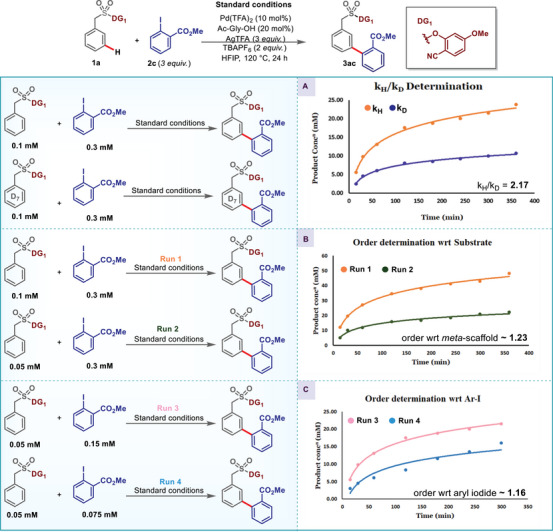
(A) *k*_H_/*k*_D_ determination. (B) Order with respect to *meta‐*substrate. (C) Order determination with respect to aryl iodide.

Initial DFT studies indicated the involvement of an Ag–Pd heterobimetallic cluster in the key *meta*‐C–H activation step that governs selectivity (see Figure S1) [51]. Within this cluster, the anionic counterion (e.g. OAc^–^) acts as a bridging ligand, imparting conformational flexibility to the transition state and enabling preferential positioning of the active palladium center near the *meta*‐C─H bond. This mechanistic insight prompted us to further investigate how the counterion might modulate the geometry and spatial orientation of the Ag–Pd cluster, thereby influencing the overall selectivity of the reaction. When acetate was used as the anionic bridging ligand, the activation energy barrier (25.9 kcal/mol) for the desired *meta*‐C–H activation was slightly lower compared to the *ortho*‐C–H activation (26.9 kcal/mol; *∆∆G* ^‡^ = 1.0 kcal/mol). The small energy difference between these two transition states resulted in low *meta*‐selectivity. In contrast, the *para‐*C–H activation proceeds via a transition state with an activation energy of +32.0 kcal/mol and should therefore not account to any product formation (Figure S2). When the anionic bridging ligand was changed from acetate toward trifluoroacetate, the energy barrier for the *meta‐*C–H activation step decreased significantly (from +25.9 kcal/mol to +19.7 kcal/mol). Whereas there was only a small reduction in the energy barrier for the *ortho*‐C–H activation (reduced by 1.2 kcal/mol). Therefore, the difference in the energy barrier for *ortho*‐ and *meta*‐C–H activation increased from 1 kcal/mol to 6 kcal/mol upon changing the bridging ligand from acetate to trifluoroacetate (Scheme 2B and Figure S3). While the calculated *∆∆G* ^‡^ value is larger than those derived from the experimental selectivity, the DFT results consistently reproduce the observed trend – showing that the trifluoroacetate ligand significantly lowers the activation barrier for *meta*‐C–H activation compared to the acetate ligand –thereby qualitatively supporting the experimentally observed enhancement in *meta*‐selectivity. Such deviations between computed and experimental selectivity are known in DFT studies of transition‐metal catalysis, where inherent uncertainties remain and relative energetic trends are typically more reliable than absolute free‐energy values [52, 53, 54]. The observed selectivity trend can be rationalized by changes in the geometry of the corresponding transition states. In the acetate‐bridged complex, the transition state for meta‐C–H activation (TS1*_meta_*) suffers from conformational strain due to a compressed Ag–N–C angle of approximately 150.2°, which deviates significantly from the nearly linear coordination geometry preferred by the *sp*‐hybridized nitrile donor. When trifluoroacetate is used as the bridging ligand, the Ag–N–C angle expands to about 165.6°, closely matching the natural coordination preference of Ag–nitrile interactions. This more open geometry relieves angle strain within the macrocyclic framework and permits a more favorable approach of the Pd center toward the distal *meta*‐C─H bond. Consequently, the TFA‐bridged transition state is both geometrically and electronically better aligned for selective *meta*‐C–H activation, consistent with the experimentally observed trend (Scheme 2B). Based on the computational output, we next sought to investigate experimentally the effect of trifluoro acetate counter anion on various substrates for *meta*‐C–H arylation reaction. To our delight, a significant improvement in selectivity and reaction yield was observed for all systems under investigation when exchanging the counter anion from acetate to trifluoro acetate, which was found to be consistent with our computational studies (Scheme 2C). For further understanding of the reaction mechanism, isotope labeling experiments were performed involving an intermolecular competition using substrate **1a** and its deuterated analogue *D*7‐**1a**, P_H_/P_D_ (intermolecular competition experiment) value of 2.3 and k_H_/k_D_ value of 2.17 were obtained (Scheme 4; Page S67).

To further explore more insights about the mechanism, we carried out the order determination studies using substrate **1a** and methyl 2‐iodobenzoate **2c** (Scheme 4; Pages S59–S66). The order with respect to *meta‐*scaffold **1a** was found to be 1.23; furthermore, the order with respect to the aryl iodide was also found to be 1.16. The observed first‐order dependence on both substrate and aryl iodide, along with a primary KInetic isotope effect [KIE] (k_H_/k_D_ = 2.17), indicates that C–H activation and oxidative addition are kinetically coupled, with C─H bond cleavage contributing to the rate‐limiting oxidative addition sequence. The amount of silver had a profound effect on the kinetics of reaction and with three equivalents of the silver salt maximum product formation was observed (for details please see Page S65). One crucial observation was the inability of Pd(0) salts such as Pd_2_(dba)_3_, Pd(PPh_3_)_4_ to promote the reaction; this suggests the involvement of a Pd(II)/Pd(IV) pathway rather than Pd(0)/Pd(II) catalytic cycle. With the optimal conditions in hand, we went for the reaction scope.

## 3 Reaction Scope

### 3.1 Benzyl Sulfonate Esters

**SCHEME 5:**
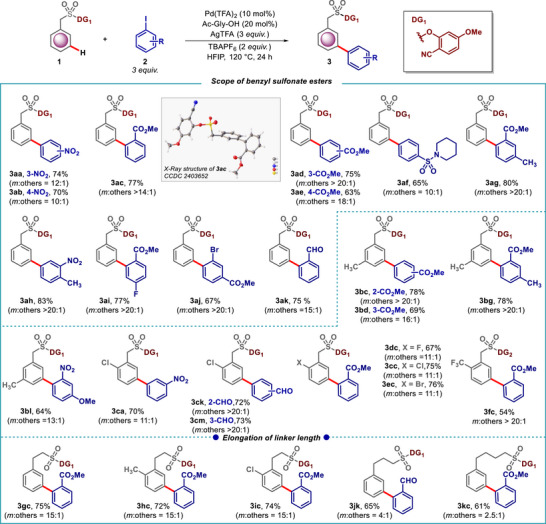
Pd‐catalyzed *meta‐*C–H arylation of various benzyl sulfonate esters.

After establishing the optimized reaction conditions, we investigated the scope of the reaction with various benzyl sulfonate esters (Scheme 5). A range of aryl coupling partners containing functional groups such as ‐NO_2_ (**2a** and **2b**)_,_ ‐CO_2_Me (**2c**‐**2e**), ‐CHO (**2k**), and ‐SO_2_ (**2f**) at different positions on the arene ring were well tolerated under the reaction conditions. Di‐substituted aryl iodides also furnished the desired *meta*‐product with good yield and *meta*‐selectivity (**2g**‐**2j**). Arenes with both electron‐donating and electron‐withdrawing substituents at the *ortho‐* and *meta*‐positions were well tolerated, resulting in the desired *meta*‐arylated products with synthetically useful yields and selectivity (**3bc**‐**3fc**). Notably, *ortho*‐chloro substrates were also compatible, yielding *meta*‐C–H arylated products (**3ca, 3cc, 3ck, 3 cm**) without interference from potential cross‐coupling reactions. The distance and geometric relationship between the directing group and the target site of C–H activation was crucial for achieving regioselective distal C–H activation. Phenethyl, phenpropropyl, phenbutyl sulfonate esters were also compatible under the optimized reaction conditions, producing *meta*‐arylated arenes effectively (**3gc**‐**3kc**). However, a systematic decrease in the *meta‐*selectivity was observed (15:1 to 2.5:1) when the linker length was increased from two to four carbons, indicating that shorter linkers provided better control over regioselectivity.

### 3.2 Phenyl Acetic Acid and Hydrocinnamic Acid Derivatives

**SCHEME 6:**
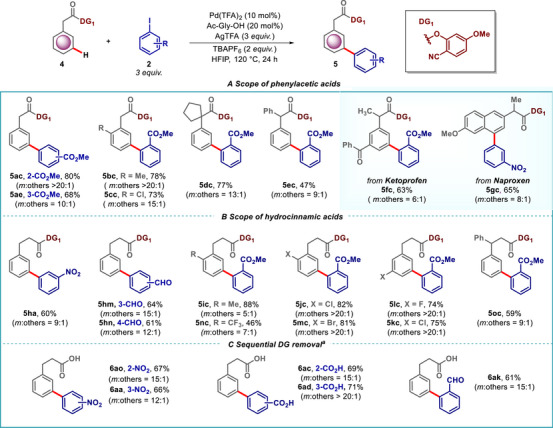
Pd‐catalyzed *meta‐*C–H arylation of phenylacetic acids and hydrocinnamic acids. *^a^* 1. Standard reaction condition. 2. LiOH.H_2_O (3 *equiv*.) THF:H_2_O:MeOH (3:1:1) RT, 6 h.

The versatility of the developed protocol was further demonstrated with phenyl acetic acid and phenyl propanoic acid derivatives (Scheme 6). Considering the prevalence of phenylacetic acid derivatives in pharmaceuticals, *meta*‐selective arylation offers a unique opportunity to study the impact of structurally modified pharmaceutical cores. Unsubstituted phenyl acetic acids worked well with both 2‐ and 4‐iodobenzoate to furnish the desired products (**5ac** and **5ae**). *Ortho‐*methyl and *ortho‐*chloro substituted phenylacetic acid derivatives (**5bc** and **5cc**) yielded the desired *meta‐*arylated compounds in good yields, displaying the protocol's efficacy (Scheme 6A). Further, phenylacetic acid derivative possessing a cyclopentyl group and a phenyl group at the benzylic position also afforded a useful yield of the *meta*‐arylated product without compromising the *meta*‐selectivity (**5dc** and **5ec**). This indicates that our method can accommodate sterically hindered substrates, thus broadening its scope. Notably, pharmaceutically relevant derivatives such as Ketoprofen and Naproxen provided the *meta‐*arylated products (**5fc** and **5gc**) with 71% and 83% yield respectively. Notably, introducing a substituent on the methylene carbon reduced the selectivity of the desired product, likely due to decreased conformational flexibility (**5ec‐5gc**). This highlights the potential of our protocol in modifying complex pharmaceutical molecules. This methodology was also extended to phenyl propionic acids, where various aryl iodides were successfully coupled at the *meta*‐position (Scheme 6b). Unsubstituted phenyl propionic acids (**4** **h**), *and ortho‐* and *meta‐*Me, Cl, Br, CF_3_ hydrocinnamic acid derivatives (**4i**‐**4n**) yielded the desired *meta*‐arylated products with good yield and selectivity. Phenyl substitution at the benzylic position was also well tolerated, providing the desired *meta‐*functionalized product (**5oc**).

### 3.3 Anilines, Benzyl Alcohols, Phenethyl Ethers, and Biphenyl Phenols

**SCHEME 7:**
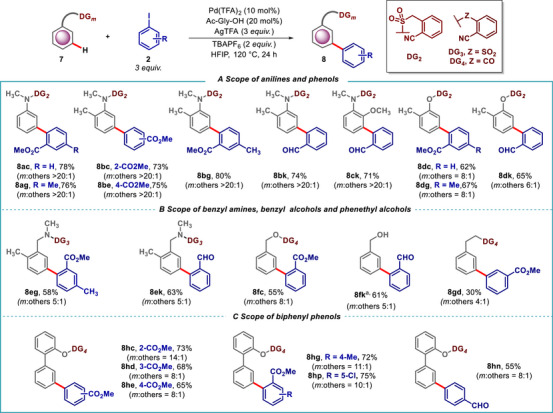
Pd‐catalyzed *meta*‐C–H arylation of anilines, phenols, benzyl amines, benzyl alcohols, and phenethyl ether, and biphenyl phenols. ^a^1. Standard reaction condition. 2. LiOH.H_2_O (3 equiv.) THF:H_2_O:MeOH (3:1:1) RT, 6 h.

Anilines and phenols are prevalent structural motifs prevalent in biologically relevant compounds, making their synthetic transformations highly desirable for obtaining functionalized derivatives. While the majority of the reports focus on *ortho*‐functionalization of anilines and phenols via electrophilic and auxiliary‐assisted *ortho*‐C–H activation [55, 56, 57], *meta‐*C–H functionalization remains challenging due to the electron‐donating nature of the nitrogen in anilines and oxygen in phenols. In our protocol, the intrinsic selectivity of these substrates *viz*. anilines, benzylamines, phenols, and benzyl alcohols have been reversed by using a suitable *meta‐*directing template (Scheme 7a,b). Both mono‐ and di‐substituted aryl iodides were successfully coupled at the *meta*‐position of the aniline and phenol derivatives (Scheme 7A). In case of methoxy‐containing aniline scaffold, sterically crowded *meta*‐C–H selectively got functionalized, owing to the electron donating effect posed by the methoxy‐ group (**8ck**). Benzyl amines and benzyl alcohols (**7e‐7f**), which can cause catalyst deactivation, were also tolerated under the reaction conditions and afforded the corresponding *meta*‐arylated product. Additionally, phenethyl ether was arylated at the *meta‐*position with methyl 3‐iodobenzoate as the arylating agent albeit in low yield and moderate *meta‐*selectivity, 40%, 4:1 (**8gd**). Our method also proved effective for *meta‐*selective arylation of biphenyl phenol scaffolds utilizing an acid‐based directing group (Scheme 7C). This protocol was extended to the synthesis of more conjugated arenes using aryl iodides bearing aldehyde and ester groups (**8hc‐8he, 8hg, 8hn, 8 hp**).

### 3.4 *N*‐Containing Heterocyles Indolines and Tetrahydro Isoquinolines

**SCHEME 8:**
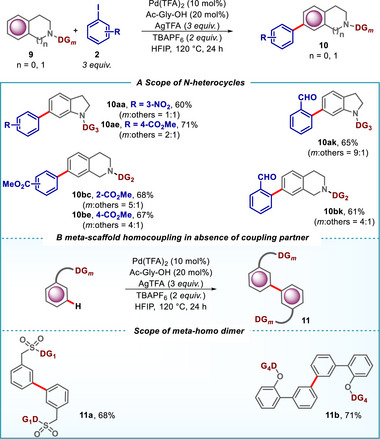
(A) Pd‐catalyzed *meta*‐C–H arylation of *N‐*heterocycles. (B) Homo‐coupling of *meta*‐scaffolds.

This methodology was also applicable to nitrogen‐containing heterocycles which are crucial structural motifs in various pharmaceuticals and natural products. Under the standard conditions, indoline and tetrahydro isoquinoline derivatives effectively delivered the C‐6 and C‐7 arylated products, respectively using a suitable directing group (Scheme 8A). Furthermore, we performed DFT studies on the C–H activation step and calculated a ∆∆G of 0.3 kcal/mol, which is in line with the observed lower selectivity compared to other directing groups (for details please see Figure S6). The successful application of our protocol to these amine‐based heterocycles (**9a** and **9b**) underscores its versatility. Under the standard reaction conditions and in the absence of the aryl iodide coupling partner, we observed the formation of the dimer of the corresponding *meta‐*scaffolds (**11a** and **11b**), which can also be utilized for further functionalization or as a building block in the synthesis of complex biaryl structures (Scheme 8B).

## 4 Conclusion and Future Outlook

In conclusion, with the insights from computational studies, we have developed a versatile protocol for *meta‐*selective arylation of arenes and heteroarenes. This protocol is applicable on a range of substrates such as benzyl sulfonate esters, phenylacetic acids, and hydrocinnamic acid derivatives. The protocol also proved effective for reversing the intrinsic selectivity of phenols, anilines, and benzyl amines and benzyl alcohols. Finally, the methodology applicability to nitrogen‐containing heterocycles such as indoline and isoquinoline, yielding C‐6 and C‐7 arylated products, which highlights its broad substrate scope. Computational studies explained the role of counter anion in providing excellent regioselectivity. Overall, our protocol presents a valuable tool for synthetic organic chemistry, offering a versatile and efficient approach for the selective functionalization of complex aromatic and heterocyclic compounds. The ability to achieve high selectivity and yield across a diverse range of substrates positions makes this methodology a promising strategy for the development of novel bioactive molecules and the modification of existing pharmaceutical cores.

## Conflicts of Interest

The authors declare no conflicts of interest.

## Supporting information




**Supporting File 1**: advs73459‐sup‐0001‐SuppMat.pdf.

**Supporting File 2**: advs73459‐sup‐0002‐DataFile.zip.

## Data Availability

The data that support the findings of this study is found in the Supporting Information file that accompanies the article. The authors have cited additional references within the Supporting Information.
