# Supplementary material for: A Generalized Approach for Distal C–H Arylation of Organic Building Blocks: Unveiling the Role of Counter Anion
Source: Adv Sci (Weinh). 2025 Dec 25;13(10):e19731. doi: 10.1002/advs.202519731 (PMC12915134; doi:10.1002/advs.202519731)
Supplement: Supplementary file 1 — Supporting File 1: advs73459‐sup‐0001‐SuppMat.pdf. [file ADVS-13-e19731-s001.pdf]

Supporting Information

A Generalized Approach for Distal C–H Arylation of Organic Building Blocks: Unveiling the Role of Counter Anion

Jagrit Grover,[1,#] Gaurav Prakash,[1,#] Astam Mandal,[1,$] Devika Ghosh,[1,$] Siddhartha Maiti,[2] Claire Empel[3*] and Debabrata Maiti[1*]

[1] Dr. J. Grover, Dr. G. Prakash, A. Mandal, D. Ghosh, Prof. Dr. D. Maiti
Department of Chemistry, Indian Institute of Technology Bombay, Powai, Mumbai-400076, India, e-mail: dmaiti@iitb.ac.in

[2] Dr. S. Maitri
School of Biosciences, Engineering and Technology, VIT Bhopal University Kothrikalan, Sehore, Madhya Pradesh, 466114 India

[c] Dr. C. Empel
Institute of Organic Chemistry, RWTH Aachen University, Landoltweg 1, 52074 Aachen, Germany, e-mail: claire.empel@rwth-aachen.de

#,$ These authors contributed equally

Supporting information for this article is given via a link at the end of the document.

List of Contents

Section	Content	Page No.
1	General Consideration	S2
2	Optimization Details	S2 – S9
3	General Procedures	S10 – S15
4	Crystal Data	S15 – S16
5	Characterization Data	S17 – S60
6	Kinetic Studies	S60 – S66
7	Reversibility Studies	S67 – S70
8	Failed Aryl iodide coupling partners	S71
9	Computational Methods	S72 – S129
10	References	S130
11	NMR Spectra	S131 – S210

1. General Consideration:

a. Reagents Information.

Unless otherwise stated, all reactions were carried out under atmospheric condition in screw cap reaction tubes. All the commercial materials and solvents were used as received unless otherwise noted. DCM was dried by distillation over CaH_2. THF was dried by distillation over sodium/benzophenone. For column chromatography, silica gel (100–200 mesh) obtained from SRL Co. and neutral activated alumina from Merck was used. A gradient elution using petroleum ether and ethyl acetate was performed, based on Merck aluminum TLC sheets (silica gel 60F254). All the benzyl chlorides and bromides were bought from Sigma Aldrich/Alfa Aesar (India)/TCI (India)/Spectrochem. $Pd(OAc)_2$ (Johnson Matthey), $Pd(TFA)_2$ (BLD pharm), AgTFA (BLD pharm), N-Ac-Gly-OH (Sigma Aldrich), were used in the Pd-catalyzed arylation reactions.

b. Analytical information. All isolated compounds are characterized by ^{1}H NMR, ^{13}C NMR spectroscopy. NMR spectra were recorded either on a Bruker 500 or 400 MHz instrument. All ^{1}H NMR experiments are reported in units, parts per million (ppm), and were measured relative to the signals for residual chloroform (7.26 ppm) in the deuterated solvent, unless otherwise stated. All ^{13}C NMR spectra were reported in ppm relative to $CDCl_3$ (77.20 ppm), All coupling constants were reported in Hertz (Hz) and all were obtained with ^{1}H decoupling. High-resolution mass spectra (HRMS) were recorded on a micro-mass ESI TOF (time of flight) mass spectrometer.

2. Optimization details for palladium-catalyzed *meta*-C–H arylation

Yield and selectivity were determined by ^{1}H NMR of crude reaction mixture using 1,3,5-trimethoxybenzene as internal standard:

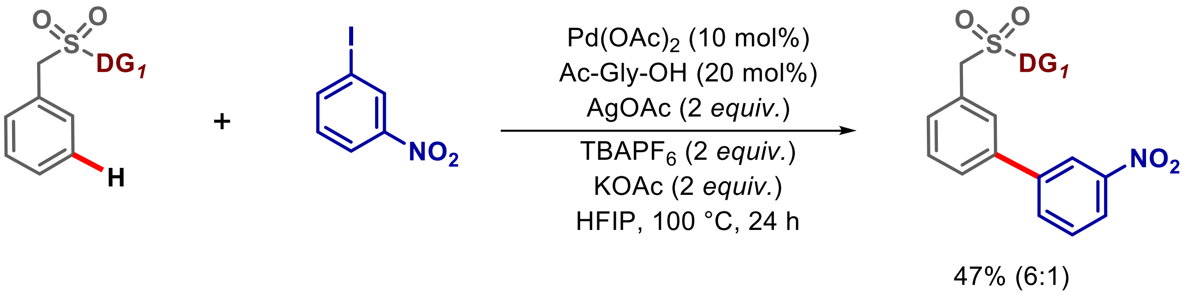

Table 1. Deviation from Initial condition:

Control Experiment	Yield (selectivity *m:others)*
Without $Pd(OAc)_2$	ND
Without Ag(OAc)	ND
**Without KOAc**	**47% (6:1)**
Without $TBAPF_6$	27% (5:1)
Without ligand	Trace

Table 2. Screening of various directing groups

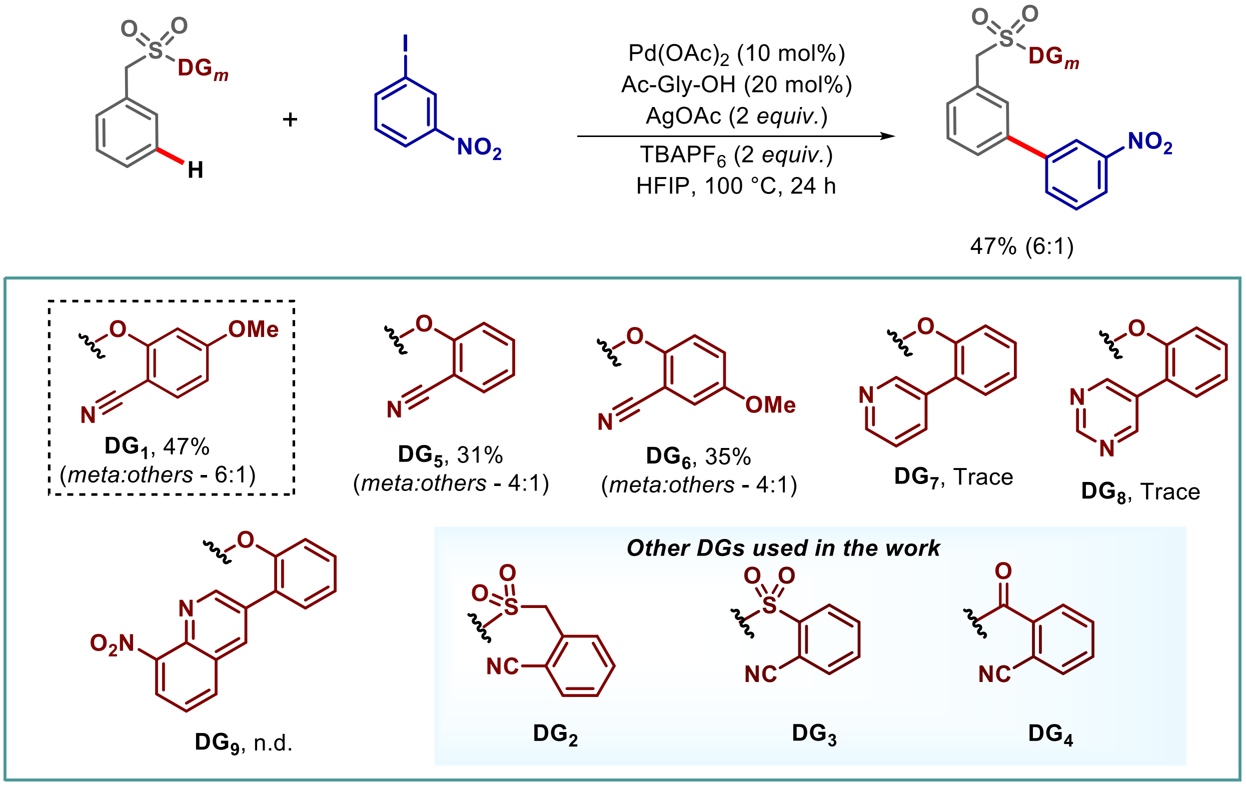

Table 3. Palladium catalyst optimization

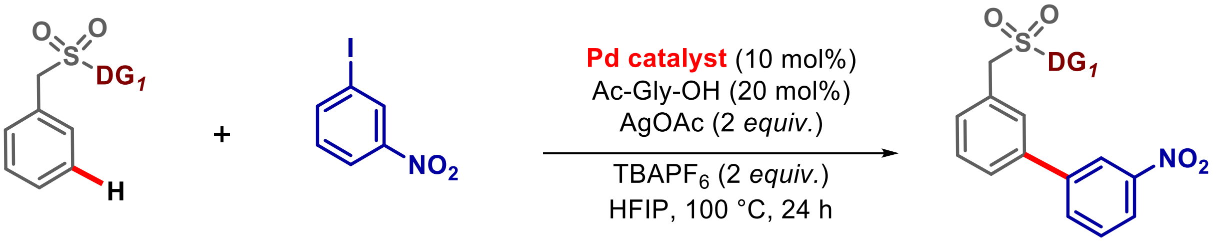

**S. No.**	**Pd catalyst**	**Yield (selectivity) (*m:others*)**
**1.**	**Pd(OAc)$_2$**	**47% (6:1)**
2.	Pd(TFA)$_2$	45% (5:1)
3.	Pd(PPh$_3$)$_4$	ND
4.	Pd(OPiv)$_2$	12% (3:1)
5.	Pd(PPh$_3$)$_2$Cl$_2$	11% (4:1)
6.	PdCl$_2$	Trace

7.	PdO	ND
8.	$Pd(acac)_2$	36% (3.7:1)
9.	$Pd(COD)Cl_2$	ND
10.	$Pd_2(dba)_3$	ND

Table 4. Silver oxidant optimization

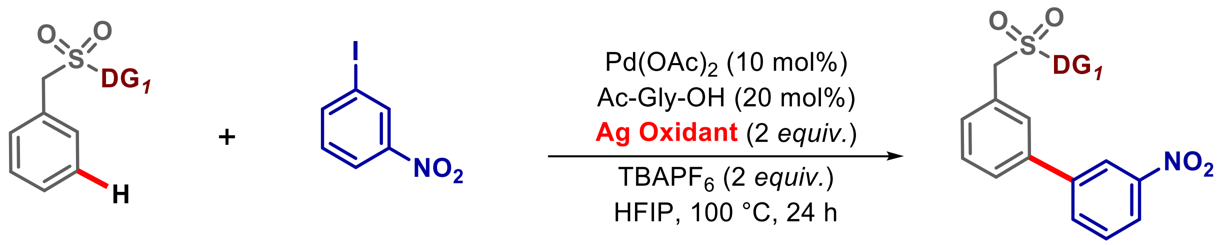

S. No.	Oxidant	Yield (selectivity *m:others)*
1.	AgOAc	47% (6:1)
2.	Ag_2CO_3	41% (5:1)
**3.**	**AgTFA**	**55% (8:1)**
4.	Ag_2SO_4	39% (4:1)
5.	Ag_2O	ND
6.	AgF	ND
7.	$AgNO_2$	ND
8.	$AgSbF_6$	ND
9.	$AgNO_3$	ND

Table 5. Oxidant amount optimization

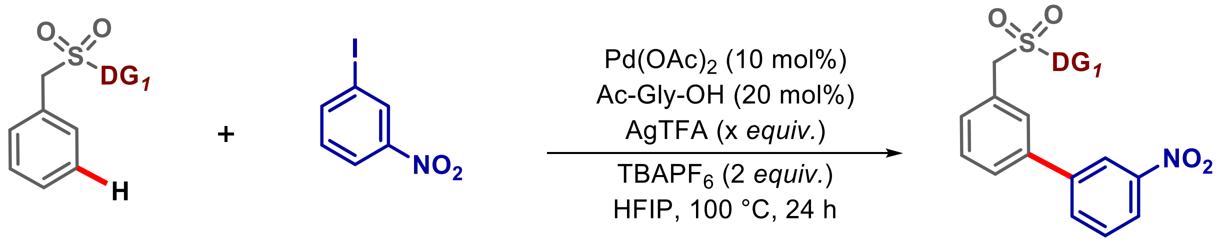

**S. No.**	**Amount of AgTFA**	**Yield (selectivity *m:others*)**
1.	1 *equiv.*	30% (3:1)
2.	2 *equiv.*	55% (8:1)
**3.**	**3 *equiv.***	**64% (9:1)**
4.	4 *equiv.*	62% (9:1)

Table 6. Ligand optimization

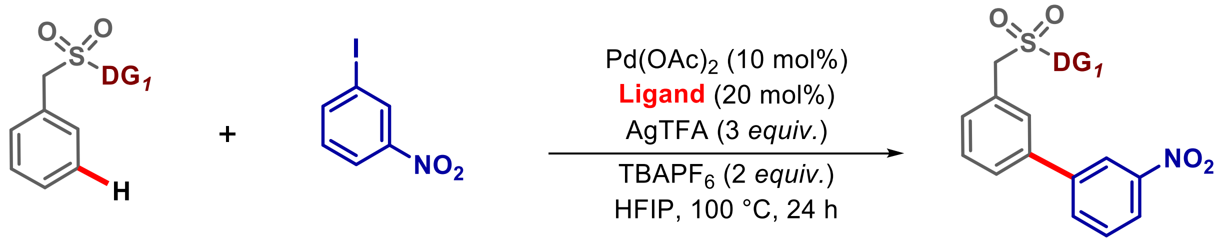

**S. No.**	**Amino Acid**	**Yield (selectivity *m:others*)**
**1.**	***N*-Ac-Gly**	**64% (9:1)**
2.	*N*-Ac-Ala	53% (8:1)
3.	*N*-Ac-Val	47% (7:1)
4.	*N*-Ac-Phenala	35% (5:1)
5.	*N*-Boc-glycine	ND
6.	Proline	ND

7.	Boc-L-Valine	Trace
8.	Gly-Gly-Gly	ND
9.	Ac-Trp-OH	ND
10.	*N*-Boc-Phenala	ND
11.	Gly-Phe	ND
12.	Fmoc-Glycine	ND
13.	*N*-Ac-DL-2-aminobutyric acid	25% (6:1)
14.	Fmoc-Val-OH	ND

Table 7. Solvent optimization

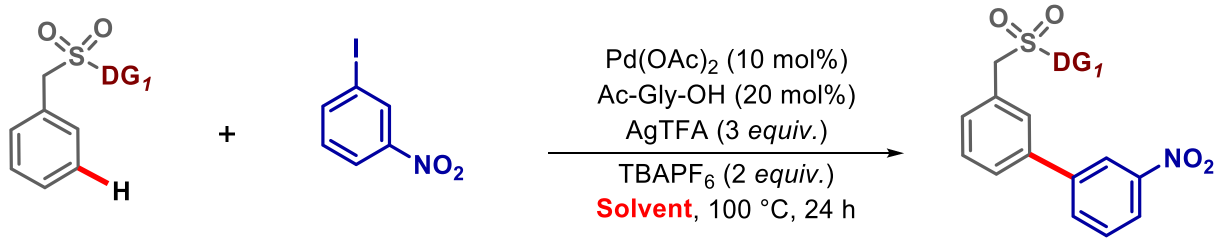

**S. No.**	**Solvent**	**Yield (selectivity *m:others*)**
**1.**	**HFIP**	**64% (9:1)**
2.	Toluene	ND
3.	TFT	ND
4.	Water	ND
5.	EtOH	ND
6.	^{t}BuOH	ND
7.	*tert*-butyl methyl ether	Trace
8.	CCl_4	ND
9.	MeCN	ND

10.	TFE	31% (6:1)
11.	Hexafluorobenzene	ND
12.	$CHCl_3$	ND
13.	Benzene	ND
14.	NMP	ND

Table 8. Additive optimization

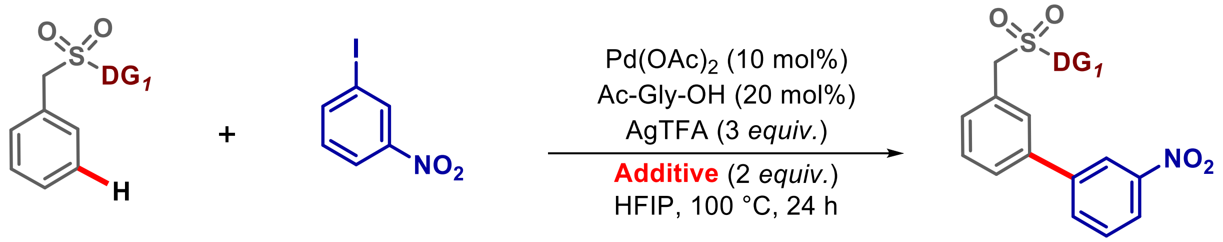

S. No.	Additive Optimization	Yield (selectivity *m:others)*
**1.**	**$TBAPF_6$**	**64% (9:1)**
2.	TBACl	58% (8:1)
3.	TBAB	50% (6:1)
4.	LiCl	45% (5:1)
5.	NaCl	43% (5:1)
6.	LiF	47% (5:1)
7.	$Cu(OAc)_2$	41% (8:1)
8.	$CuCl_2$	48% (9:1)
9.	$Cr_2Cu_2O_5$	40% (9:1)
10.	CuO	35% (8:1)
11.	$Cu(NO_3)_2$	Trace

12.	$CuBr_2$	Trace
13.	$CuCO_3$	38% (7:1)

Table 9. Temperature optimization

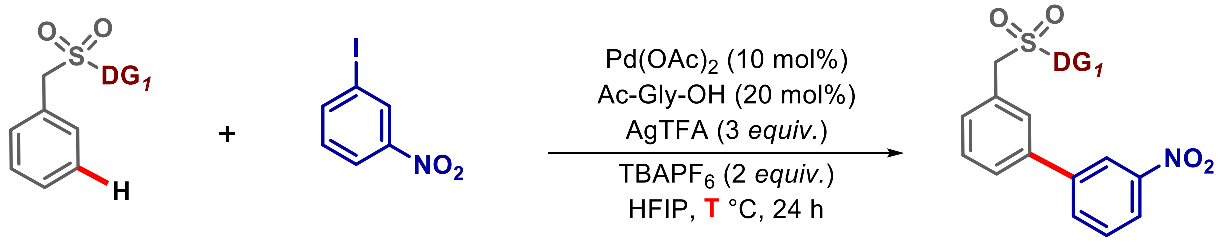

S. No.	Temperature	Yield (selectivity *m:others)*
1.	80 °C	45% (7:1)
2.	90 °C	54% (7:1)
3.	100 °C	64% (9:1)
4.	110 °C	68% (9:1)
**5.**	**120 °C**	**70% (9:1)**
6.	130 °C	66% (8:1)

Table 10. Effect of combination of Pd:Ag Salt

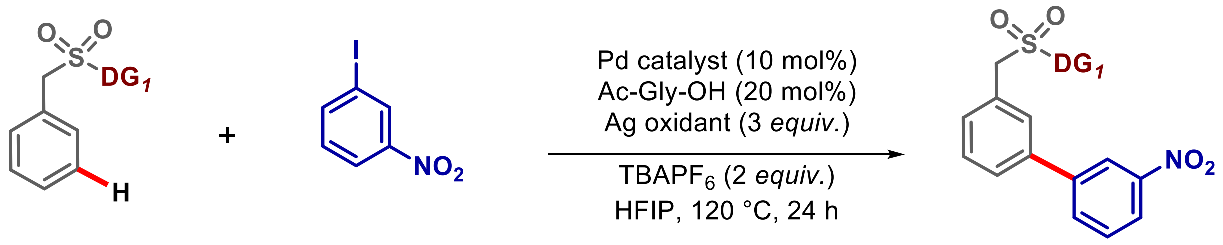

S. No.	Combination of Pd:Ag	Yield (selectivity *m:others)*
1.	$Pd(OAc)_2$ + AgTFA	70% (9:1)

**2.**	**Pd(TFA)$_2$ + AgTFA**	**74% (12:1)**

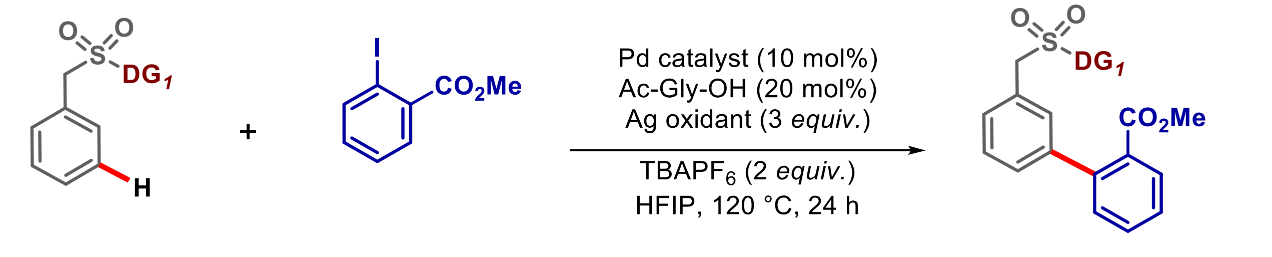

1.	Pd(OAc)$_2$ + AgOAc	62% (4:1)
**2.**	**Pd(TFA)$_2$ + AgTFA**	**77% (14:1)**

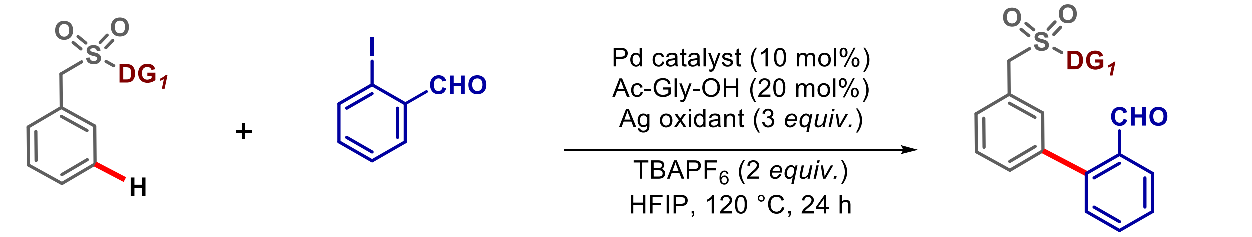

1.	Pd(OAc)$_2$ + AgOAc	65% (6:1)
**2.**	**Pd(TFA)$_2$ + AgTFA**	**75% (15:1)**

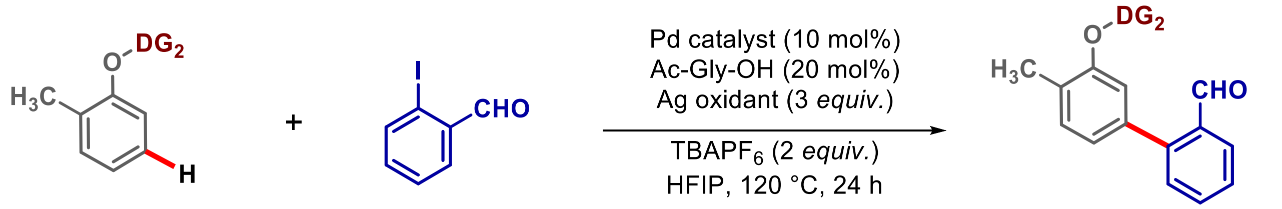

1.	Pd(OAc)$_2$ + AgOAc	53% (2:1)
**2.**	**Pd(TFA)$_2$ + AgTFA**	**65% (6:1)**

3. General procedures for the preparation of substrates and coupling partners

a. General procedure A for the preparation of 2-Hydroxy-4-methoxybenzonitrile (DG)

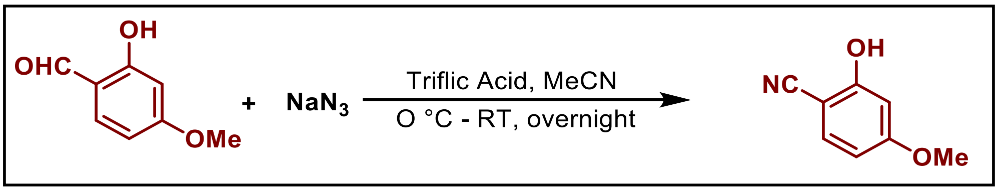

In an oven dried round bottom flask (250 mL), charged with stir-bar, 2-Hydroxy-4-methoxy benzaldehyde (20 mmol) and NaN_3 (3 equiv.) were taken. Acetonitrile (60 mL) was added to it

and stirred at room temperature for 15 mins. Then 3 equiv. of triflic acid was added to the mixture in portion with a plastic dropper. After the addition, the reaction was allowed to stir at room temperature for 12 hr. Upon completion the organic solvent was evaporated under reduced pressure. The solid residue was dissolved in ethyl acetate and washed with saturated $NaHCO_3$ solution (3 times). The organic fraction was then dried over anhydrous Na_2SO_4 and purified through column chromatography using silica gel and petroleum ether/ethyl acetate (80/20, v/v) as the eluent. Quantitative conversion; off-white solid.

(b) General procedure B for preparation of sulfonyl ester scaffolds

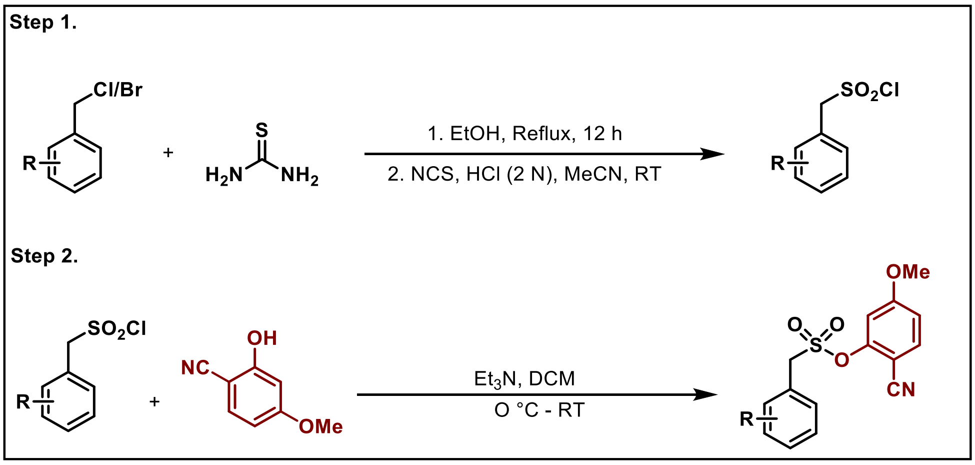

Step 1. An oven dried clean round bottom flask was charged with magnetic stir-bar, substituted benzyl chloride (10 mmol) and thiourea (10 mmol, 760 mg). 10 ml of absolute ethanol was added and refluxed at 96 °C. After 3 h the reaction was taken out and solvent was evaporated under reduced pressure to obtained white solid thiourea salt. The obtained solid salt was suspended in 14 ml of CH_3CN and 3 ml 2N HCl was added to it. The mixture was stirred at 0 °C for 15 min. *N*-chlorosuccinimide (NCS) (40 mmol; 5.34 g) was added in portion to the suspension in order to obtain a clear solution. The solution was stirred for another 30 min at room temperature. The solution was evaporated under reduced pressure to remove the CH_3CN. The remaining aqueous portion was extracted with ethyl acetate. The organic portion was dried over anhydrous Na_2SO_4 and the crude mixture was evaporated and purified by column chromatography using silica gel and ethyl acetate/petroleum ether as the eluent. Quantitative yield.

Step 2. To an ice-cold solution of 2-hydroxy-4-methoxybenzonitrile (5 mmol) and triethylamine (1.5 equiv, 1.04 mL) in 10 mL dichloromethane under nitrogen atmosphere, substituted benzyl sulfonyl chloride was added portion wise. Stirring was continued for additional 20 minutes, after that the ice bath was removed and the reaction mixture was left for vigorous stirring at room temperature for overnight. CH_2Cl_2 was removed under reduced pressure. The residual was diluted and extracted with ethyl acetate (3 x 20 mL) and brine solution (3 x 10 mL). The organic layer was collected and dried over anhydrous Na_2SO_4. After filtration and evaporation of the solvent, the crude mixture was purified by column chromatography using neutral alumina and petroleum-ether/ethyl acetate (85/15, v/v) as the eluent.

(c). General procedure C for the synthesis of carbonyl ester

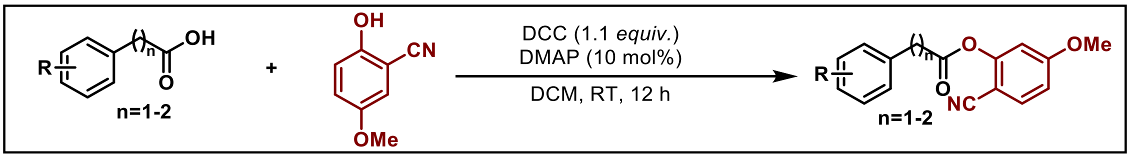

To a stirred solution of carboxylic acid (1 equiv, 10 mmol) and DMAP (1 mmol) in 30 mL anhydrous CH_2Cl_2, 2-hydroxy-4-methoxybenzonitrile (1.5 equiv, 15 mmol) was added. After 15 minute of stirring, DCC (11 mmol) was added to the reaction mixture at 0˚C and then allowed to stir overnight at room temperature. Upon completion of reaction, precipitated urea is then filtered off. Filtrate was evaporated and the residue was dissolved in CH_2Cl_2 and washed with saturated $NaHCO_3$ solution, and then dried over anhydrous Na_2SO_4. The solvent was removed under reduced pressure and the residue was purified by column chromatography on silica gel (eluent: ethyl acetate/petroleum ether) to give the desired ester.

(d) General procedure D for the synthesis of biphenyl phenol based scaffolds

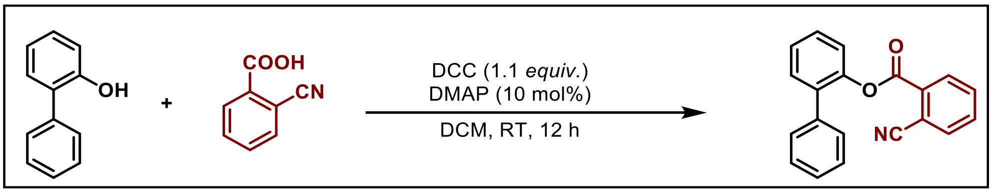

To a stirred solution of 2-cyanonenzoic acid (1 equiv, 10 mmol) and DMAP (1 mmol) in 30 mL anhydrous CH_2Cl_2, 2-phenyl phenol (1.5 equiv, 15 mmol) was added. After 15 minute of stirring, DCC (11 mmol) was added to the reaction mixture at 0˚C and then allowed to stir overnight at room temperature. Upon completion of reaction, precipitated urea is then filtered off. Filtrate was evaporated and the residue was dissolved in CH_2Cl_2 and washed with saturated $NaHCO_3$ solution, and then dried over anhydrous Na_2SO_4. The solvent was removed under reduced pressure and the residue was purified by column chromatography on silica gel (eluent: ethyl acetate/petroleum ether) to give the desired ester.

(e) General procedure E for the synthesis of aniline based scaffolds

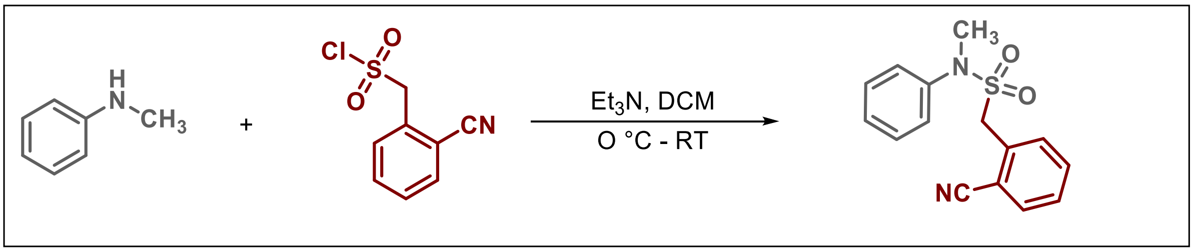

To an ice-cold solution of *N*-methyl Aniline (5 mmol) and triethylamine (1.5 equiv, 1.04 mL) in 10 mL dichloromethane under nitrogen atmosphere, (2-cyanophenyl)methanesulfonyl chloride (2 equiv) was added portion wise. Stirring was continued for additional 20 minutes, after that the ice bath was removed and the reaction mixture was left for vigorous stirring at room temperature for overnight. CH_2Cl_2 was removed under reduced pressure. The residual was diluted and extracted with ethyl acetate (3 x 20 mL) and brine solution (3 x 10 mL). The organic layer was collected and dried over anhydrous Na_2SO_4. After filtration and evaporation

of the solvent, the crude mixture was purified by column chromatography on silica gel (eluent: ethyl acetate/petroleum ether) to give the desired scaffold. (eluent: petroleum-ether/ethyl acetate, 85/15, v/v)

(f) General procedure F for the synthesis of benzyl amine based scaffolds

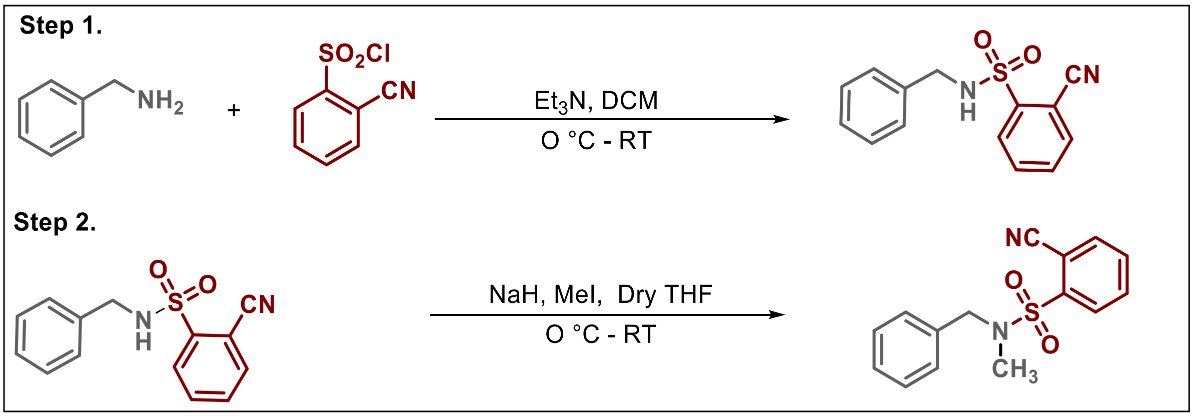

Step 1: To an ice-cold solution of benzyl amine (5 mmol) and triethylamine (1.5 equiv, 1.04 mL) in 10 mL dichloromethane under nitrogen atmosphere, 2-cyanobenzenesulfonyl chloride (2 equiv) was added portion wise. Stirring was continued for additional 20 minutes, after that the ice bath was removed and the reaction mixture was left for vigorous stirring at room temperature for overnight. CH_2Cl_2 was removed under reduced pressure. The residual was diluted and extracted with ethyl acetate (3 x 20 mL) and brine solution (3 x 10 mL). The organic layer was collected and dried over anhydrous Na_2SO_4. After filtration and evaporation of the solvent, the crude mixture was purified by column chromatography on silica gel (eluent: ethyl acetate/petroleum ether) to give the desired *N*-benzyl-2-cyanobenzenesulfonamide. (eluent: petroleum-ether/ethyl acetate, 85/15, v/v)

Step 2: *N*-benzyl-2-cyanobenzenesulfonamide was dissolved in 10 mL of dry THF and NaH (2 equiv) was added portion wise. Stirring was continued for additional 20 minutes under ice-cold conditions. Methyl iodide (2 equiv) was added to the solution. After that the ice bath was removed and the reaction mixture was left for vigorous stirring at room temperature for overnight. CH_2Cl_2 was removed under reduced pressure. The residual was diluted and extracted with ethyl acetate (3 x 20 mL) and brine solution (3 x 10 mL). The organic layer was collected and dried over anhydrous Na_2SO_4. After filtration and evaporation of the solvent, the crude mixture was purified by column chromatography on silica gel (eluent: ethyl acetate/petroleum ether) to give the desired *N*-benzyl-2-cyano-*N*-methylbenzenesulfonamide. (eluent: petroleum-ether/ethyl acetate, 90/10, v/v)

(G) General procedure G for the synthesis of benzyl alcohol based scaffolds

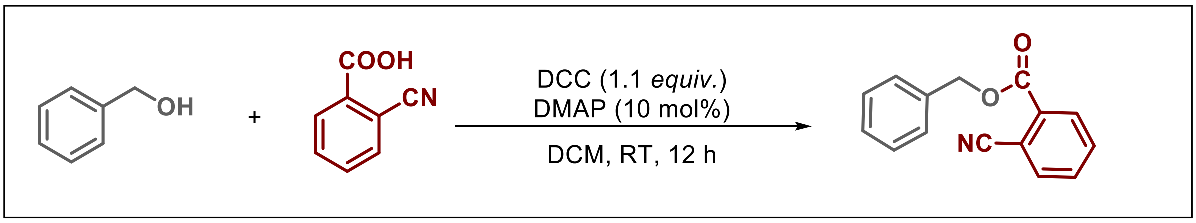

To a stirred solution of 2-cyanonenzoic acid (1 equiv, 10 mmol) and DMAP (1 mmol) in 30 mL anhydrous CH_2Cl_2, benzyl alcohol (1.5 equiv, 15 mmol) was added. After 15 minute of

stirring, DCC (11 mmol) was added to the reaction mixture at 0˚C and then allowed to stir overnight at room temperature. Upon completion of reaction, precipitated urea is then filtered off. Filtrate was evaporated and the residue was dissolved in CH_2Cl_2 and washed with saturated $NaHCO_3$ solution, and then dried over anhydrous Na_2SO_4. The solvent was removed under reduced pressure and the residue was purified by column chromatography on silica gel (eluent: ethyl acetate/petroleum ether) to give the desired ester.

(H) General procedure H for the synthesis of phenol based scaffolds

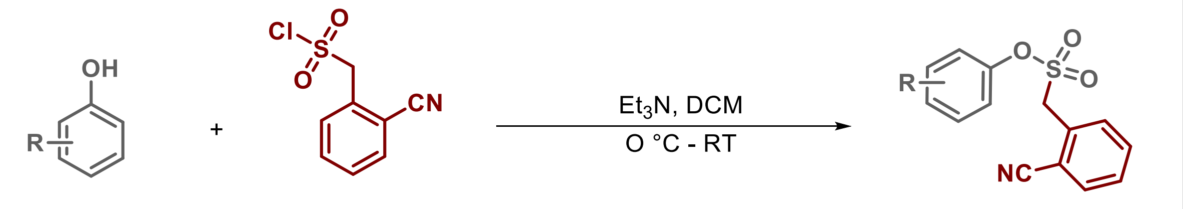

To an ice-cold solution of Phenol (5 mmol) and triethylamine (1.5 equiv, 1.04 mL) in 10 mL dichloromethane under nitrogen atmosphere, (2-cyanophenyl)methanesulfonyl chloride (2 equiv) was added portion wise. Stirring was continued for additional 20 minutes, after that the ice bath was removed and the reaction mixture was left for vigorous stirring at room temperature for overnight. CH_2Cl_2 was removed under reduced pressure. The residual was diluted and extracted with ethyl acetate (3 x 20 mL) and brine solution (3 x 10 mL). The organic layer was collected and dried over anhydrous Na_2SO_4. After filtration and evaporation of the solvent, the crude mixture was purified by column chromatography on silica gel (eluent: ethyl acetate/petroleum ether) to give the desired phenyl (2-cyanophenyl)methanesulfonate. (eluent: petroleum-ether/ethyl acetate, 85/15, v/v)

(I) General procedure I for the synthesis of indoline based scaffolds

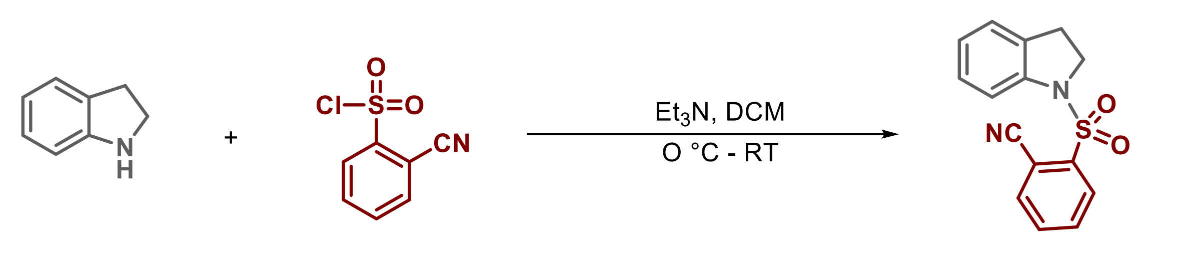

To an ice-cold solution of indoline (5 mmol) and triethylamine (1.5 equiv, 1.04 mL) in 10 mL dichloromethane under nitrogen atmosphere, 2-cyanobenzenesulfonyl chloride chloride (2 equiv) was added portion wise. Stirring was continued for additional 20 minutes, after that the ice bath was removed and the reaction mixture was left for vigorous stirring at room temperature for overnight. CH_2Cl_2 was removed under reduced pressure. The residual was diluted and extracted with ethyl acetate (3 x 20 mL) and brine solution (3 x 10 mL). The organic layer was collected and dried over anhydrous Na_2SO_4. After filtration and evaporation of the solvent, the crude mixture was purified by column chromatography on silica gel (eluent: ethyl acetate/petroleum ether) to give the desired 2-((indolin-1-ylsulfonyl)methyl)benzonitrile. (eluent: petroleum-ether/ethyl acetate, 85/15, v/v)

(J) General procedure J for the synthesis of isoquinoline based scaffolds

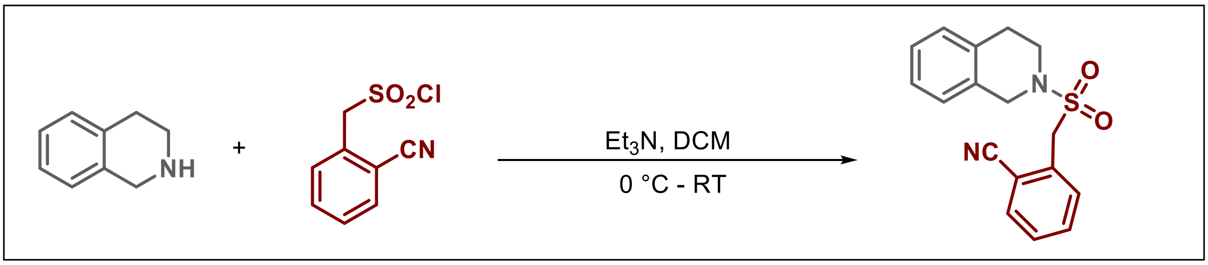

To an ice-cold solution of isoquinoline (5 mmol) and triethylamine (1.5 equiv, 1.04 mL) in 10 mL dichloromethane under nitrogen atmosphere, (2-cyanophenyl)methanesulfonyl chloride (2 equiv) was added portion wise. Stirring was continued for additional 20 minutes, after that the ice bath was removed and the reaction mixture was left for vigorous stirring at room temperature for overnight. CH_2Cl_2 was removed under reduced pressure. The residual was diluted and extracted with ethyl acetate (3 x 20 mL) and brine solution (3 x 10 mL). The organic layer was collected and dried over anhydrous Na_2SO_4. After filtration and evaporation of the solvent, the crude mixture was purified by column chromatography on silica gel (eluent: ethyl acetate/petroleum ether) to give the desired 2-((3,4-dihydroisoquinolin-2(1H)-yl)sulfonyl)benzonitrile (eluent: petroleum-ether/ethyl acetate, 85/15, v/v)

(K) General procedure K for palladium-catalyzed *meta*-C–H arylation of arenes

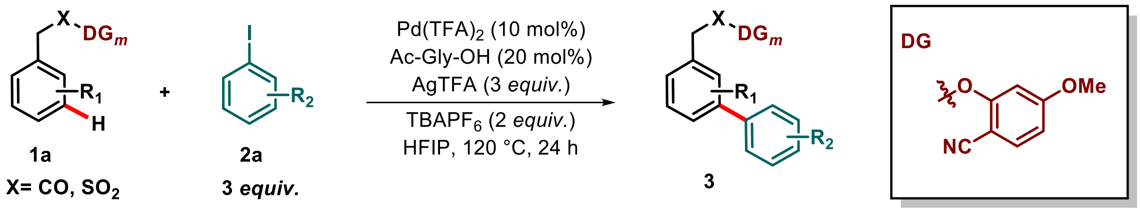

In an oven-dried screw cap reaction tube charged with a magnetic stir-bar sulphonyl ester scaffold (0.2 mmol), $Pd(TFA)_2$ (10 mol%), *N*-Ac-Gly (20 mol%), AgTFA (3 *equiv*., 0.6 mmol), $TBAPF_6$ (2 *equiv*., 0.4 mmol) and corresponding aryl halide (3 *equiv*., 0.6mmol) were taken. After that 2 mL HFIP was added under aerobic condition. The tube was tightly capped and placed in a preheated oil bath at 120 °C and the reaction mixture was stirred for 24 h. After completion of the reaction, the reaction mixture was then cooled to room temperature and filtered through a celite pad with ethyl acetate. The filtrate was concentrated under vacuum and the desired *meta*-arylated compounds were purified using column chromatography using silica gel and ethyl acetate/petroleum ether as the eluent.

(L) General procedure L for one-pot directing group removal of acid derivatives

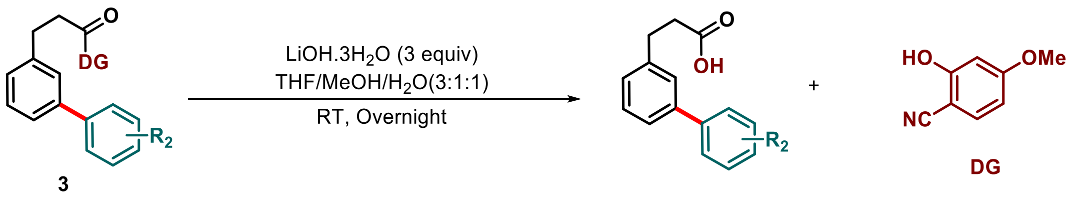

When the reaction was subjected for *meta*-C–H arylation using the general procedure K and after completion of the reaction, the reaction mixture was cooled to room temperature and

filtered through a celite pad with ethyl acetate. The filtrate was concentrated under vacuum and then crude reaction mixture was subjected to hydrolysis using 3 equivalents of $LiOH.3H_2O$ in THF/MeOH/H_2O (3:1:1) as solvent at room temperature for overnight. The reaction was further quenched by 2N HCl and purified using column chromatography using silica gel and ethyl acetate/petroleum ether or DCM/Methanol as the eluent.

4. Crystal data

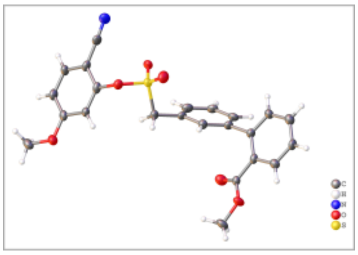

CCDC 2403652

Crystal data and structure refinement for DM_JG_764_RRR_autored.

Identification code	DM_JG_764_RRR_autored
Empirical formula	$C_{23}H_{19}NO_6S$
Formula weight	437.48
Temperature/K	150.00(10)
Crystal system	monoclinic
Space group	$P2_1/c$
a/Å	7.6531(15)
b/Å	16.702(2)
c/Å	15.820(2)
α/°	90
β/°	90.802(14)
γ/°	90
Volume/Å^3	2022.0(5)
Z	4
ρ_{calc}g/cm^3	1.4370
μ/mm^{-1}	0.202
F(000)	913.0
Crystal size/mm^3	0.114 × 0.098 × 0.095

Radiation	Mo Kα (λ = 0.71073)
2Θ range for data collection/°	4.88 to 50
Index ranges	-11 ≤ h ≤ 10, -27 ≤ k ≤ 25, -22 ≤ l ≤ 24
Reflections collected	30690
Independent reflections	3513 [R_{int} = 0.1678, R_{sigma} = 0.1682]
Data/restraints/parameters	3513/0/282
Goodness-of-fit on F^2	1.483
Final R indexes [I>=2σ (I)]	R_1 = 0.1474, wR_2 = 0.3733
Final R indexes [all data]	R_1 = 0.1685, wR_2 = 0.3963
Largest diff. peak/hole / e $Å^{-3}$	1.22/-1.05

5. Characterization data of product

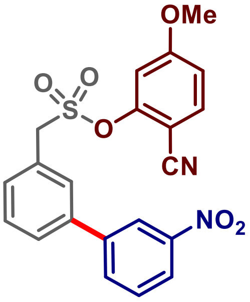

2-cyano-5-methoxyphenyl (3'-nitro-[1,1'-biphenyl]-3-yl)methanesulfonate (3aa): Compound 3aa was synthesized using the general procedure K.

Column material: 100-200 mesh silica

Eluent: petroleum ether/ethyl acetate (85:15, v/v).

Isolated Yield: 74%

^{1}H NMR (400 MHz, $CDCl_3$) δ 8.45 (t, J = 2.0 Hz, 1H), 8.22 (ddd, J = 8.2, 2.4, 1.0 Hz, 1H), 7.93 (dt, J = 7.8, 1.4 Hz, 1H), 7.77 (s, 1H), 7.73 – 7.51 (m, 5H), 6.95 (d, J = 2.4 Hz, 1H), 6.90 (dd, J = 8.8, 2.4 Hz, 1H), 4.81 (s, 2H), 3.85 (s, 3H).

^{13}C NMR (101 MHz, $CDCl_3$) δ 164.33, 151.80, 148.91, 141.96, 139.85, 134.65, 133.39, 131.31, 130.16, 130.07, 129.99, 128.55, 127.64, 122.69, 122.23, 115.70, 114.02, 109.43, 98.70, 58.54, 56.31.

HRMS: [ESI, (+) ve]: Cald. 425.0807 for $C_{21}H_{17}N_2O_6S$ observed 425.0809.

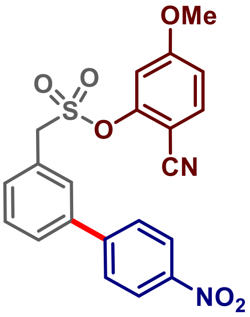

2-cyano-5-methoxyphenyl (4'-nitro-[1,1'-biphenyl]-3-yl)methanesulfonate (3ab): Compound 3ab was synthesized using the general procedure K.

Column material: 100-200 mesh silica

Eluent: petroleum ether/ethyl acetate (85:15, v/v).

Isolated Yield: 70%

^{1}H NMR (500 MHz, CDCl$_3$) δ 8.31 (d, *J* = 8.8 Hz, 2H), 7.78 (d, *J* = 1.9 Hz, 1H), 7.76 (d, *J* = 8.8 Hz, 2H), 7.69 (dt, *J* = 7.5, 1.6 Hz, 1H), 7.63 – 7.54 (m, 3H), 6.95 (d, *J* = 2.4 Hz, 1H), 6.90 (dd, *J* = 8.7, 2.4 Hz, 1H), 4.81 (s, 2H), 3.85 (s, 3H).

^{13}C NMR (126 MHz, CDCl$_3$) δ 164.18, 151.61, 147.41, 146.48, 139.79, 134.49, 131.46, 130.05, 129.98, 128.58, 128.03, 127.49, 124.20, 115.56, 113.86, 109.31, 98.54, 58.41, 56.16.

HRMS: [ESI, (+) ve]: Cald. 425.0807 for $C_{21}H_{17}N_2O_6S$ observed 425.0809.

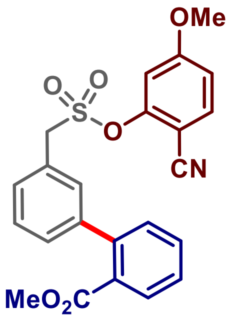

methyl 3'-(((2-cyano-5-methoxyphenoxy)sulfonyl)methyl)-[1,1'-biphenyl]-2-carboxylate (3ac): Compound 3ac was synthesized using the general procedure K.

Column material: 100-200 mesh silica

Eluent: petroleum ether/ethyl acetate (85:15, v/v).

Isolated Yield: 77%

^{1}H NMR (400 MHz, CDCl$_3$) δ 7.86 (dd, *J* = 7.8, 1.4 Hz, 1H), 7.58 (d, *J* = 8.6 Hz, 1H), 7.56 – 7.49 (m, 2H), 7.49 – 7.39 (m, 3H), 7.37 (td, *J* = 7.5, 1.4 Hz, 2H), 7.01 – 6.84 (m, 2H), 4.75 (s, 2H), 3.83 (s, 3H), 3.63 (s, 3H).

^{13}C NMR (101 MHz, CDCl$_3$) δ 168.77, 164.26, 151.84, 142.54, 141.65, 134.58, 131.60, 131.17, 130.99, 130.77, 130.20, 130.04, 129.71, 128.92, 127.75, 126.22, 115.64, 113.98, 109.51, 98.88, 58.57, 56.26, 52.21.

HRMS: [ESI, (+) ve]: Cald. 460.0831 for $C_{23}H_{19}O_6NNaS$ observed 460.0831.

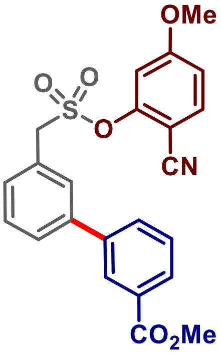

methyl 3'-(((2-cyano-5-methoxyphenoxy)sulfonyl)methyl)-[1,1'-biphenyl]-3-carboxylate (3ad): Compound 3ad was synthesized using the general procedure K.

Column material: 100-200 mesh silica

Eluent: petroleum ether/ethyl acetate (85:15, v/v).

Isolated Yield: 75%

^{1}H NMR (500 MHz, CDCl$_3$) δ 8.27 (s, 1H), 8.09 – 7.97 (m, 1H), 7.83 – 7.76 (m, 1H), 7.75 (s, 1H), 7.69 (d, *J* = 6.9 Hz, 1H), 7.59 (s, 1H), 7.54 (d, *J* = 7.6 Hz, 3H), 6.96 – 6.83 (m, 2H), 4.80 (s, 2H), 3.95 (s, 3H), 3.83 (s, 3H).

^{13}C NMR (126 MHz, CDCl$_3$) δ 167.12, 164.30, 151.91, 141.28, 140.54, 134.59, 131.83, 130.98, 130.54, 129.99, 129.89, 129.20, 129.02, 128.56, 128.46, 127.23, 115.70, 114.09, 109.36, 98.80, 58.66, 56.29, 52.45.

HRMS: [ESI, (+) ve]: Cald. 438.1011 for $C_{23}H_{20}O_6NS$ observed 438.1010.

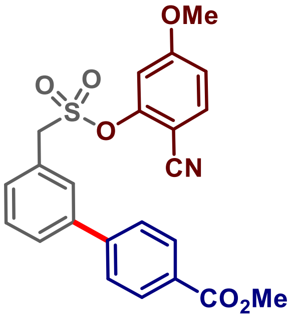

methyl 3'-(((2-cyano-5-methoxyphenoxy)sulfonyl)methyl)-[1,1'-biphenyl]-4-carboxylate (3ae): Compound 3ae was synthesized using the general procedure K.

Column material: 100-200 mesh silica

Eluent: petroleum ether/ethyl acetate (85:15, v/v).

Isolated Yield: 63%

^{1}H NMR (500 MHz, CDCl$_3$) δ 8.11 (d, *J* = 8.3 Hz, 2H), 7.76 (d, *J* = 1.9 Hz, 1H), 7.68 (t, *J* = 8.1 Hz, 3H), 7.61 – 7.51 (m, 3H), 6.98 – 6.83 (m, 2H), 4.80 (s, 2H), 3.94 (s, 3H), 3.83 (s, 3H).

^{13}C NMR (126 MHz, CDCl$_3$) δ 167.07, 164.32, 151.90, 144.64, 141.18, 134.60, 130.90, 130.38, 130.11, 129.91, 129.55, 128.65, 127.36, 127.31, 115.71, 114.08, 109.39, 98.81, 58.69, 56.30, 52.37.

HRMS: [ESI, (+) ve]: Cald. 460.0831 for $C_{23}H_{19}O_6NNaS$ observed 460.0831.

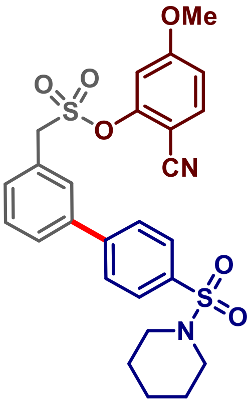

2-cyano-5-methoxyphenyl (4'-(piperidin-1-ylsulfonyl)-[1,1'-biphenyl]-3-yl)methanesulfonate (3af): Compound 3af was synthesized using the general procedure K.

Column material: 100-200 mesh silica

Eluent: petroleum ether/ethyl acetate (80:20, v/v).

Isolated Yield: 65%

^{1}H NMR (400 MHz, CDCl$_3$) δ 7.90 – 7.82 (m, 2H), 7.76 (s, 1H), 7.73 (t, J = 2.4 Hz, 2H), 7.66 (dt, J = 6.8, 1.9 Hz, 1H), 7.57 (d, J = 8.5 Hz, 1H), 7.53 (d, J = 2.2 Hz, 2H), 4.78 (s, 2H), 3.82 (s, 3H), 3.04 (t, J = 5.5 Hz, 4H), 1.66 (dd, J = 11.7, 6.1 Hz, 4H), 1.48 – 1.40 (m, 2H).

^{13}C NMR (101 MHz, CDCl$_3$) δ 164.27, 151.90, 143.63, 141.19, 136.39, 134.59, 130.52, 130.02, 129.85, 128.56, 128.02, 127.22, 115.71, 114.04, 109.24, 98.74, 58.61, 56.28, 47.14, 25.34, 23.65.

HRMS: [ESI, (+) ve]: Cald. 549.1124 for $C_{26}H_{26}O_6N_2NaS_2$ observed 549.1121.

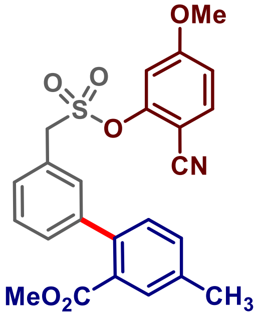

methyl 3'-(((2-cyano-5-methoxyphenoxy)sulfonyl)methyl)-4,5'-dimethyl-[1,1'-biphenyl]-2-carboxylate (3ag): Compound 3ag was synthesized using the general procedure K.

Column material: 100-200 mesh silica

Eluent: petroleum ether/ethyl acetate (85:15, v/v).

Isolated Yield: 80%

^{1}H NMR (400 MHz, CDCl$_3$) δ 7.72 – 7.65 (m, 1H), 7.58 (d, J = 8.6 Hz, 1H), 7.53 – 7.39 (m, 3H), 7.39 – 7.30 (m, 2H), 7.24 (s, 1H), 6.95 – 6.82 (m, 2H), 4.74 (s, 2H), 3.84 (s, 3H), 3.62 (s, 3H), 2.42 (s, 3H).

^{13}C NMR (101 MHz, CDCl$_3$) δ 169.03, 164.29, 151.92, 142.57, 138.85, 137.72, 134.59, 132.35, 131.26, 130.95, 130.73, 130.60, 129.88, 129.82, 128.90, 126.18, 115.67, 114.04, 109.50, 98.94, 58.66, 56.29, 52.20, 21.12.

HRMS: [ESI, (+) ve]: Cald. 474.0987 for $C_{24}H_{21}O_6NNaS$ observed 474.0984.

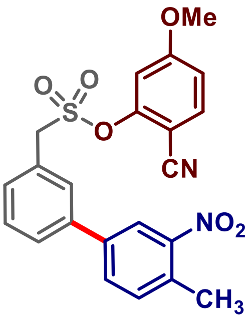

2-cyano-5-methoxyphenyl (4'-methyl-3'-nitro-[1,1'-biphenyl]-3-yl)methanesulfonate (3ah): Compound 3ah was synthesized using the general procedure K.

Column material: 100-200 mesh silica

Eluent: petroleum ether/ethyl acetate (85:15, v/v).

Isolated Yield: 83%

^{1}H NMR (500 MHz, CDCl$_3$) δ 8.20 (d, J = 2.0 Hz, 1H), 7.74 (dt, J = 5.3, 1.9 Hz, 2H), 7.66 (dt, J = 7.2, 1.7 Hz, 1H), 7.61 – 7.52 (m, 3H), 7.42 (d, J = 7.9 Hz, 1H), 6.94 (d, J = 2.4 Hz, 1H), 6.89 (dd, J = 8.7, 2.4 Hz, 1H), 4.79 (s, 2H), 3.85 (s, 3H), 2.64 (s, 3H).

^{13}C NMR (126 MHz, CDCl$_3$) δ 164.33, 151.86, 149.80, 139.73, 139.41, 134.63, 133.57, 133.01, 131.62, 131.02, 130.09, 129.76, 128.32, 127.55, 123.26, 115.70, 114.04, 109.40, 98.73, 58.60, 56.31, 20.36.

HRMS: [ESI, (+) ve]: Cald. 461.0778 for $C_{22}H_{18}O_6N_2NaS$ observed 461.0777.

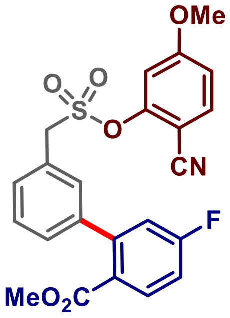

methyl 3'-(((2-cyano-5-methoxyphenoxy)sulfonyl)methyl)-5-fluoro-[1,1'-biphenyl]-2-carboxylate (3ai): Compound 3ai was synthesized using the general procedure K.

Column material: 100-200 mesh silica

Eluent: petroleum ether/ethyl acetate (85:15, v/v).

Isolated Yield: 77%

^{1}H NMR (400 MHz, CDCl$_3$) δ 7.62 – 7.55 (m, 2H), 7.52 (dt, *J* = 7.7, 1.5 Hz, 1H), 7.46 (td, *J* = 7.6, 0.6 Hz, 1H), 7.41 (t, *J* = 1.8 Hz, 1H), 7.38 – 7.30 (m, 2H), 7.26 – 7.20 (m, 1H), 6.93 (d, *J* = 2.4 Hz, 1H), 6.89 (dd, *J* = 8.7, 2.4 Hz, 1H), 4.74 (s, 2H), 3.85 (s, 3H), 3.64 (s, 3H).

^{13}C NMR (101 MHz, CDCl$_3$) δ 167.45, 164.30, 163.18, 160.71, 151.82, 141.58, 137.87, 134.62, 132.89, 132.82, 132.31, 131.26, 130.21, 129.82, 129.00, 126.32, 118.78, 118.57, 117.29, 117.05, 115.66, 114.00, 109.55, 98.87, 58.56, 56.30, 52.51.

HRMS: [ESI, (+) ve]: Cald. 478.0731 for $C_{23}H_{18}O_6FNNaS$ observed 478.0730.

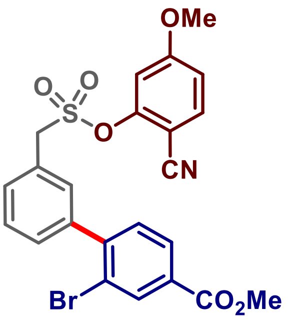

methyl 2-bromo-3'-(((2-cyano-5-methoxyphenoxy)sulfonyl)methyl)-[1,1'-biphenyl]-4-carboxylate (3aj): Compound 3aj was synthesized using the general procedure K.

Column material: 100-200 mesh silica

Eluent: petroleum ether/ethyl acetate (80:20, v/v).

Isolated Yield: 67%

^{1}H NMR (500 MHz, CDCl$_3$) δ 8.34 (d, *J* = 1.6 Hz, 1H), 8.01 (dd, *J* = 8.0, 1.7 Hz, 1H), 7.63 – 7.56 (m, 3H), 7.55 – 7.47 (m, 2H), 7.41 (d, *J* = 8.0 Hz, 1H), 7.04 – 6.76 (m, 2H), 4.78 (s, 2H), 3.95 (s, 3H), 3.84 (s, 3H).

^{13}C NMR (126 MHz, CDCl$_3$) δ 165.60, 164.13, 151.66, 145.76, 140.99, 134.46, 134.37, 131.83, 131.28, 130.93, 130.84, 130.42, 128.99, 128.54, 126.37, 122.48, 115.49, 113.87, 109.30, 98.70, 58.37, 56.14, 52.49.

HRMS: [ESI, (+) ve]: Cald. 537.9930 for $C_{23}H_{18}O_6BrNNaS$ observed 537.9932.

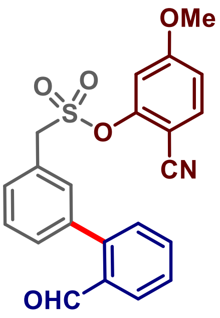

2-cyano-5-methoxyphenyl (2'-formyl-[1,1'-biphenyl]-3-yl)methanesulfonate (3ak): Compound 3ak was synthesized using the general procedure K.

Column material: 100-200 mesh silica

Eluent: petroleum ether/ethyl acetate (85:15, v/v).

Isolated Yield: 75%

^{1}H NMR (500 MHz, CDCl$_3$) δ 9.97 (s, 1H), 8.03 (d, *J* = 7.8 Hz, 1H), 7.69 – 7.60 (m, 2H), 7.59 – 7.48 (m, 4H), 7.45 (d, *J* = 7.6 Hz, 2H), 6.93 (d, *J* = 2.4 Hz, 1H), 6.89 (dd, *J* = 8.8, 2.4 Hz, 1H), 4.79 (s, 2H), 3.85 (s, 3H).

^{13}C NMR (126 MHz, CDCl$_3$) δ 192.21, 164.29, 151.71, 144.79, 138.91, 134.63, 133.87, 133.83, 132.62, 131.46, 131.01, 130.94, 129.36, 128.39, 128.02, 126.99, 115.62, 114.00, 109.48, 98.78, 58.41, 56.31.

HRMS: [ESI, (+) ve]: Cald. 408.0900 for $C_{22}H_{18}O_5NS$ observed 408.0901.

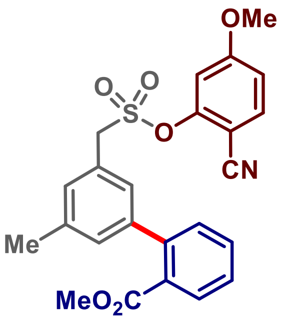

methyl 3'-(((2-cyano-5-methoxyphenoxy)sulfonyl)methyl)-5'-methyl-[1,1'-biphenyl]-2-carboxylate (3bc): Compound 3bc was synthesized using the general procedure K.

Column material: 100-200 mesh silica

Eluent: petroleum ether/ethyl acetate (85:15, v/v).

Isolated Yield: 78%

^{1}H NMR (500 MHz, CDCl$_3$) δ 7.83 (dd, *J* = 7.8, 1.5 Hz, 1H), 7.58 (d, *J* = 8.7 Hz, 1H), 7.52 (td, *J* = 7.5, 1.4 Hz, 1H), 7.41 (td, *J* = 7.6, 1.3 Hz, 1H), 7.35 (dd, *J* = 7.6, 1.3 Hz, 1H), 7.32 (d, *J* = 2.1 Hz, 1H), 7.28 – 7.21 (m, 1H), 7.19 (d, *J* = 1.9 Hz, 1H), 6.93 (d, *J* = 2.4 Hz, 1H), 6.88 (dd, *J* = 8.7, 2.4 Hz, 1H), 4.70 (s, 2H), 3.84 (s, 3H), 3.64 (s, 3H), 2.42 (s, 3H).

^{13}C NMR (126 MHz, CDCl$_3$) δ 168.97, 164.27, 151.91, 142.39, 141.73, 138.85, 134.56, 131.50, 130.94, 130.86, 130.76, 130.45, 130.08, 128.37, 127.62, 125.98, 115.64, 113.95, 109.48, 98.87, 58.58, 56.25, 52.23, 21.44.

HRMS: [ESI, (+) ve]: Cald. 474.0982 for $C_{24}H_{21}O_6NNaS$ observed 474.0984.

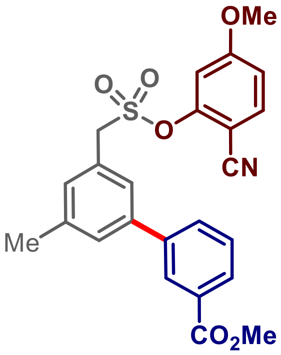

methyl 3'-(((2-cyano-5-methoxyphenoxy)sulfonyl)methyl)-5'-methyl-[1,1'-biphenyl]-3-carboxylate (3bd): Compound 3bd was synthesized using the general procedure K.

Column material: 100-200 mesh silica

Eluent: petroleum ether/ethyl acetate (85:15, v/v).

Isolated Yield: 69%

^{1}H NMR (500 MHz, CDCl$_3$) δ 8.37 – 8.11 (m, 1H), 8.03 (dd, J = 7.5, 1.8 Hz, 1H), 7.83 – 7.74 (m, 1H), 7.58 (d, J = 8.7 Hz, 1H), 7.56 – 7.45 (m, 3H), 7.35 (s, 1H), 6.93 (d, J = 2.4 Hz, 1H), 6.88 (dd, J = 8.7, 2.4 Hz, 1H), 4.75 (s, 2H), 3.95 (s, 3H), 3.83 (s, 3H), 2.46 (s, 3H).

^{13}C NMR (126 MHz, CDCl$_3$) δ 167.16, 164.29, 151.99, 141.17, 140.70, 139.81, 134.57, 131.83, 131.22, 130.92, 129.39, 129.12, 128.90, 128.43, 127.14, 127.02, 115.69, 114.03, 109.31, 98.79, 58.66, 56.26, 52.42, 21.55.

HRMS: [ESI, (+) ve]: Cald. 474.0982 for $C_{24}H_{21}O_6NNaS$ observed 474.0985.

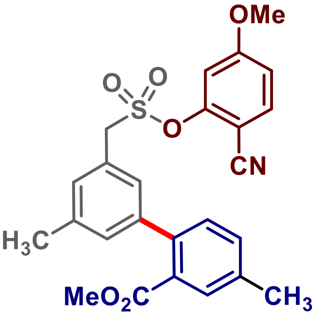

methyl 3'-(((2-cyano-5-methoxyphenoxy)sulfonyl)methyl)-4,5'-dimethyl-[1,1'-biphenyl]-2-carboxylate (3bg): Compound 3bg was synthesized using the general procedure K.

Column material: 100-200 mesh silica

Eluent: petroleum ether/ethyl acetate (85:15, v/v).

Isolated Yield: 78%

^{1}H NMR (400 MHz, CDCl$_3$) δ 7.64 (d, J = 1.9 Hz, 1H), 7.58 (d, J = 8.7 Hz, 1H), 7.36 – 7.28 (m, 2H), 7.28 – 7.13 (m, 3H), 6.92 (d, J = 2.4 Hz, 1H), 6.88 (dd, J = 8.7, 2.5 Hz, 1H), 4.70 (s, 2H), 3.84 (s, 3H), 3.63 (s, 3H), 2.41 (s, 6H).

^{13}C NMR (101 MHz, CDCl$_3$) δ 169.14, 164.23, 151.90, 142.33, 138.83, 138.78, 137.54, 134.55, 132.23, 130.84, 130.63, 130.56, 130.48, 128.41, 125.89, 115.65, 113.95, 109.44, 98.84, 58.56, 56.24, 52.19, 21.45, 21.08.

HRMS: [ESI, (+) ve]: Cald. 488.1138 for $C_{25}H_{23}O_6NNaS$ observed 488.1140.

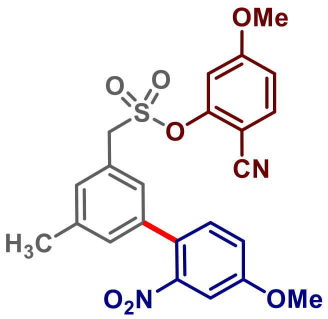

2-cyano-5-methoxyphenyl (4'-methoxy-5-methyl-2'-nitro-[1,1'-biphenyl]-3 yl)methanesulfonate (3bl): Compound 3bl was synthesized using the general procedure K.

Column material: 100-200 mesh silica

Eluent: petroleum ether/ethyl acetate (83:17, v/v).

Isolated Yield: 64%

^{1}H NMR (400 MHz, CDCl$_3$) δ 7.57 (d, J = 8.7 Hz, 1H), 7.38 (d, J = 2.6 Hz, 1H), 7.36 – 7.31 (m, 2H), 7.23 (d, J = 1.9 Hz, 1H), 7.18 – 7.11 (m, 2H), 6.93 (d, J = 2.4 Hz, 1H), 6.88 (dd, J = 8.7, 2.4 Hz, 1H), 4.70 (s, 2H), 3.90 (s, 3H), 3.84 (s, 3H), 2.40 (s, 3H).

^{13}C NMR (101 MHz, CDCl$_3$) δ 164.27, 159.49, 151.82, 149.60, 139.50, 138.44, 134.57, 133.11, 131.41, 130.19, 128.03, 127.99, 126.75, 118.97, 115.65, 114.05, 109.33, 98.84, 58.39, 56.29, 56.13, 21.47.

HRMS: [ESI, (+) ve]: Cald. 491.0883 for $C_{23}H_{20}O_7N_2NaS$ observed 491.0885.

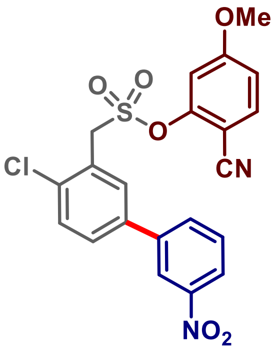

2-cyano-5-methoxyphenyl (4-chloro-3'-nitro-[1,1'-biphenyl]-3-yl)methanesulfonate (3ca): Compound 3ca was synthesized using the general procedure K.

Column material: 100-200 mesh silica

Eluent: petroleum ether/ethyl acetate (85:15, v/v).

Isolated Yield: 70%

^{1}H NMR (400 MHz, $CDCl_3$) δ 8.43 (t, *J* = 2.0 Hz, 1H), 8.34 – 8.18 (m, 1H), 8.01 – 7.80 (m, 2H), 7.72 – 7.54 (m, 4H), 6.99 (d, *J* = 2.4 Hz, 1H), 6.91 (dd, *J* = 8.7, 2.5 Hz, 1H), 5.04 (s, 2H), 3.86 (s, 3H).

^{13}C NMR (101 MHz, $CDCl_3$) δ 164.34, 151.52, 148.97, 140.84, 138.41, 136.24, 134.75, 133.26, 131.77, 131.23, 130.23, 129.81, 126.05, 122.98, 122.14, 115.52, 114.12, 109.45, 98.87, 56.33, 55.51.

HRMS: [ESI, (+) ve]: Cald. 459.0412 for $C_{21}H_{16}ClN_2O_6S$ observed 459.0400.

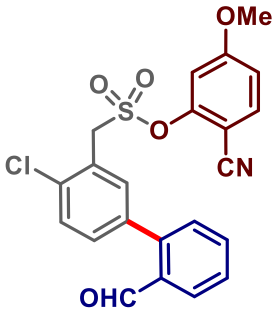

2-cyano-5-methoxyphenyl (4-chloro-2'-formyl-[1,1'-biphenyl]-3-yl)methanesulfonate (3ck): Compound 3ck was synthesized using the general procedure K.

Column material: 100-200 mesh silica

Eluent: petroleum ether/ethyl acetate (80:20, v/v).

Isolated Yield: 72%

^{1}H NMR (500 MHz, $CDCl_3$) δ 9.99 (s, 1H), 8.03 (dd, *J* = 7.8, 1.4 Hz, 1H), 7.68 (d, *J* = 2.2 Hz, 1H), 7.65 (dd, *J* = 7.6, 1.5 Hz, 1H), 7.63 – 7.49 (m, 3H), 7.44 (dd, *J* = 7.7, 1.2 Hz, 1H), 7.40 (dd, *J* = 8.3, 2.2 Hz, 1H), 6.97 (d, *J* = 2.4 Hz, 1H), 6.90 (dd, *J* = 8.7, 2.4 Hz, 1H), 5.02 (s, 2H), 3.86 (s, 3H).

^{13}C NMR (126 MHz, $CDCl_3$) δ 191.84, 164.30, 151.37, 143.44, 137.60, 135.89, 134.73, 134.38, 133.98, 133.85, 132.66, 131.00, 130.46, 128.73, 128.48, 125.41, 115.40, 114.15, 109.55, 99.07, 56.33, 55.33.

HRMS: [ESI, (+) ve]: Cald. 442.0510 for $C_{22}H_{17}ClNO_5S$ observed 442.0512.

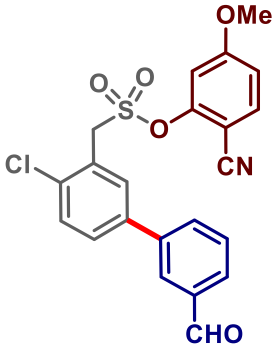

2-cyano-5-methoxyphenyl (4-chloro-3'-formyl-[1,1'-biphenyl]-3-yl)methanesulfonate (3cm): Compound 3cm was synthesized using the general procedure K.

Column material: 100-200 mesh silica

Eluent: petroleum ether/ethyl acetate (83:17, v/v).

Isolated Yield: 73%

^{1}H NMR (400 MHz, CDCl$_3$) δ 10.09 (s, 1H), 8.09 (d, J = 1.8 Hz, 1H), 7.95 – 7.80 (m, 3H), 7.71 – 7.54 (m, 4H), 6.97 (d, J = 2.4 Hz, 1H), 6.90 (dd, J = 8.7, 2.4 Hz, 1H), 5.04 (s, 2H), 3.85 (s, 3H).

^{13}C NMR (101 MHz, CDCl$_3$) δ 192.09, 164.15, 151.41, 139.93, 139.24, 137.03, 135.46, 134.56, 132.99, 131.56, 130.88, 129.79, 129.63, 129.33, 128.11, 125.56, 115.38, 113.96, 109.23, 98.71, 56.15, 55.40.

HRMS: [ESI, (+) ve]: Cald. 442.0510 for $C_{22}H_{17}ClNO_5S$ observed 442.0511.

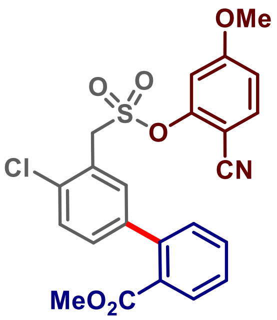

methyl 4'-chloro-3'-(((2-cyano-5-methoxyphenoxy)sulfonyl)methyl)-[1,1'-biphenyl]-2-carboxylate (3cc): Compound 3cc was synthesized using the general procedure K.

Column material: 100-200 mesh silica

Eluent: petroleum ether/ethyl acetate (80:20, v/v).

Isolated Yield: 75%

^{1}H NMR (500 MHz, CDCl$_3$) δ 7.90 (dd, J = 7.8, 1.4 Hz, 1H), 7.65 – 7.48 (m, 4H), 7.44 (td, J = 7.7, 1.3 Hz, 1H), 7.33 (ddd, J = 11.9, 7.9, 1.7 Hz, 2H), 6.96 (d, J = 2.4 Hz, 1H), 6.89 (dd, J = 8.7, 2.4 Hz, 1H), 4.98 (s, 2H), 3.85 (s, 3H), 3.67 (s, 3H).

^{13}C NMR (126 MHz, CDCl$_3$) δ 168.34, 164.26, 151.58, 141.19, 140.73, 134.76, 134.67, 133.05, 131.84, 131.20, 131.06, 130.47, 130.42, 129.94, 128.09, 124.57, 115.46, 114.07, 109.53, 99.07, 56.29, 55.47, 52.31.

HRMS: [ESI, (+) ve]: Cald. 494.0436 for $C_{23}H_{18}ClNNaO_6S$ observed 494.0432.

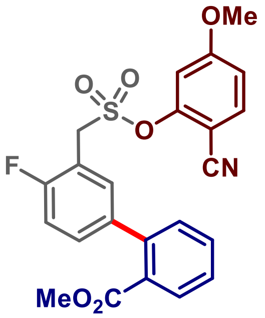

methyl 3'-(((2-cyano-5-methoxyphenoxy)sulfonyl)methyl)-4'-fluoro-[1,1'-biphenyl]-2-carboxylate (3dc): Compound 3dc was synthesized using the general procedure K.

Column material: 100-200 mesh silica

Eluent: petroleum ether/ethyl acetate (81:19, v/v).

Isolated Yield: 67%

^{1}H NMR (500 MHz, CDCl$_3$) δ 7.88 (dd, *J* = 7.7, 1.4 Hz, 1H), 7.53 (td, *J* = 7.6, 1.4 Hz, 1H), 7.48 (dd, *J* = 6.9, 2.3 Hz, 1H), 7.43 (td, *J* = 7.6, 1.3 Hz, 1H), 7.37 – 7.31 (m, 3H), 7.19 (t, *J* = 9.0 Hz, 1H), 7.17 – 7.08 (m, 2H), 4.80 (s, 2H), 3.83 (s, 3H), 3.67 (s, 3H).

^{13}C NMR (126 MHz, CDCl$_3$) δ 168.52, 161.91, 159.91, 158.23, 143.36, 140.92, 138.46, 132.79, 131.91, 131.84, 131.75, 131.15, 130.60, 130.38, 130.01, 127.91, 127.34, 125.07, 120.60, 117.96, 115.95, 115.77, 114.96, 114.06, 113.93, 108.37, 56.22, 52.30, 51.41, 51.39.

^{19}F NMR (471 MHz, CDCl$_3$) δ -118.02.

HRMS: [ESI, (+) ve]: Cald. 478.0731 for $C_{23}H_{18}FNNaO_6S$ observed 478.0735

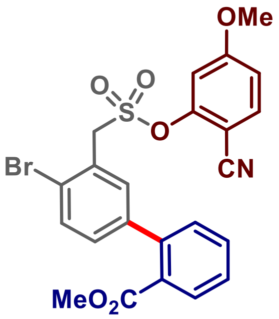

methyl 4'-bromo-3'-(((2-cyano-5-methoxyphenoxy)sulfonyl)methyl)-[1,1'-biphenyl]-2-carboxylate (3ec): Compound 3ec was synthesized using the general procedure K.

Column material: 100-200 mesh silica

Eluent: petroleum ether/ethyl acetate (80:20, v/v).

Isolated Yield: 66%

^{1}H NMR (400 MHz, CDCl$_3$) δ 7.89 (dd, *J* = 7.7, 1.4 Hz, 1H), 7.68 (d, *J* = 8.3 Hz, 1H), 7.62 – 7.49 (m, 3H), 7.44 (td, *J* = 7.6, 1.3 Hz, 1H), 7.33 (dd, *J* = 7.6, 1.3 Hz, 1H), 7.23 (dd, *J* = 8.3,

2.2 Hz, 1H), 6.96 (d, J = 2.4 Hz, 1H), 6.89 (dd, J = 8.7, 2.4 Hz, 1H), 5.01 (s, 2H), 3.84 (s, 3H), 3.67 (s, 3H).

^{13}C NMR (101 MHz, CDCl$_3$) δ 168.30, 164.24, 151.52, 141.81, 140.69, 134.66, 133.28, 133.01, 131.85, 131.31, 130.98, 130.48, 130.31, 128.09, 126.39, 125.02, 115.46, 114.07, 109.55, 99.05, 57.88, 56.28, 52.32.

HRMS: [ESI, (+) ve]: Cald. 537.9930 for $C_{23}H_{18}BrNNaO_6S$ observed 537.9929.

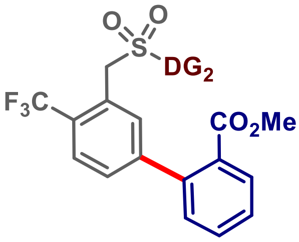

methyl 3'-(((2-cyano-5-methoxyphenoxy)sulfonyl)methyl)-4'-(trifluoromethyl)-[1,1'-biphenyl]-2-carboxylate (3fc): Compound 3fc was synthesized using the general procedure K.

Column material: 100-200 mesh silica

Eluent: petroleum ether/ethyl acetate (80:20, v/v).

Isolated Yield: 54%

^{1}H NMR (500 MHz, CDCl$_3$) δ 7.95 (d, J = 7.8 Hz, 1H), 7.76 (s, 1H), 7.65 (s, 1H), 7.63 (s, 1H), 7.59 (ddd, J = 11.5, 6.3, 3.7 Hz, 2H), 7.49 (t, J = 7.6 Hz, 1H), 7.36 (d, J = 7.6 Hz, 1H), 6.95 (t, J = 1.7 Hz, 1H), 6.91 (dt, J = 8.8, 1.8 Hz, 1H), 4.80 (s, 2H), 3.86 (s, 3H), 3.65 (s, 3H).

^{13}C NMR (101 MHz, CDCl$_3$) δ 168.1, 164.4, 151.7, 143.7, 140.5, 134.7, 134.6, 132.1, 131.7, 131.4, 131.1, 130.8, 130.5, 128.5, 127.3, 126.7 (dd, $J_{C\text{–}F}$ = 15.3, 3.8 Hz), 125.2, 115.6, 114.3, 109.7, 98.9, 58.1, 56.4, 52.4.

^{19}F NMR (376 MHz, CDCl$_3$) δ -62.5.

HRMS: [ESI, (+) ve]: calcd. 506.0885 for $C_{24}H_{19}F_3NO_6S$, observed: 506.0884

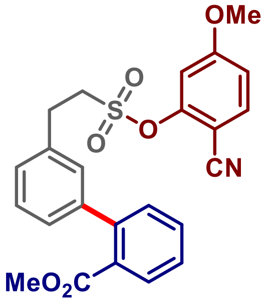

methyl 3'-(2-((2-cyano-5-methoxyphenoxy)sulfonyl)ethyl)-[1,1'-biphenyl]-2-carboxylate (3gc): Compound 3gc was synthesized using the general procedure K.

Column material: 100-200 mesh silica

Eluent: petroleum ether/ethyl acetate (82:18, v/v).

Isolated Yield: 75%

^{1}H NMR (400 MHz, CDCl$_3$) δ 8.26 (t, *J* = 1.7 Hz, 1H), 8.03 (dt, *J* = 7.8, 1.4 Hz, 1H), 7.78 (ddd, *J* = 7.7, 1.9, 1.1 Hz, 1H), 7.60 (d, *J* = 8.8 Hz, 1H), 7.58 – 7.47 (m, 3H), 7.44 (dd, *J* = 8.2, 7.6 Hz, 1H), 7.31 (dt, *J* = 7.7, 1.4 Hz, 1H), 7.05 (d, *J* = 2.4 Hz, 1H), 6.91 (dd, *J* = 8.8, 2.4 Hz, 1H), 3.96 (s, 3H), 3.89 (s, 3H), 3.80 – 3.73 (m, 2H), 3.50 – 3.42 (m, 2H).

^{13}C NMR (101 MHz, CDCl$_3$) δ 167.20, 164.32, 151.54, 141.08, 137.57, 134.66, 131.75, 130.92, 129.78, 129.14, 128.79, 128.42, 128.07, 127.90, 127.59, 126.37, 115.63, 114.09, 109.64, 98.76, 56.34, 53.86, 52.43, 29.98.

HRMS: [ESI, (+) ve]: Cald. 474.0982 for $C_{24}H_{21}NNaO_6S$ observed 474.0979.

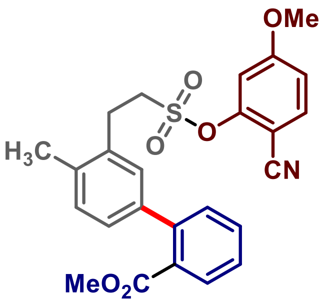

methyl 3'-(2-((2-cyano-5-methoxyphenoxy)sulfonyl)ethyl)-4'-methyl-[1,1'-biphenyl]-2-carboxylate (3hc): Compound 3hc was synthesized using the general procedure K.

Column material: 100-200 mesh silica

Eluent: petroleum ether/ethyl acetate (82:18, v/v).

Isolated Yield: 72%

^{1}H NMR (400 MHz, CDCl$_3$) δ 7.82 (dd, *J* = 7.7, 1.4 Hz, 1H), 7.59 (d, *J* = 8.7 Hz, 1H), 7.54 – 7.49 (m, 1H), 7.40 (td, *J* = 7.6, 1.3 Hz, 1H), 7.35 (dd, *J* = 7.7, 1.3 Hz, 1H), 7.25 – 7.16 (m, 2H), 7.13 (dd, *J* = 7.7, 2.0 Hz, 1H), 7.06 (d, *J* = 2.4 Hz, 1H), 6.90 (dd, *J* = 8.8, 2.5 Hz, 1H), 3.88 (s, 3H), 3.68 (s, 3H), 3.48 – 3.37 (m, 2H), 2.42 (s, 3H), 1.26 (t, *J* = 7.1 Hz, 2H).

^{13}C NMR (101 MHz, CDCl$_3$) δ 169.09, 164.26, 151.35, 142.08, 139.77, 135.33, 134.84, 134.65, 131.50, 130.94, 130.75, 130.66, 129.99, 129.07, 127.68, 127.33, 115.51, 113.95, 109.40, 98.87, 56.29, 52.52, 52.20, 27.42, 19.14.

HRMS: [ESI, (+) ve]: Cald. 488.1138 for $C_{25}H_{23}NNaO_6S$ observed 488.1140.

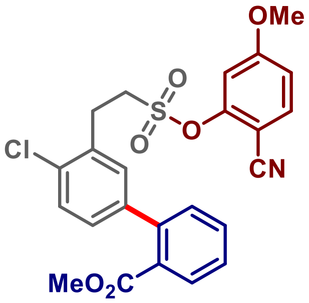

methyl 4'-chloro-3'-(2-((2-cyano-5-methoxyphenoxy)sulfonyl)ethyl)-[1,1'-biphenyl]-2-carboxylate (3ic): Compound 3ic was synthesized using the general procedure K.

Column material: 100-200 mesh silica

Eluent: petroleum ether/ethyl acetate (86:14, v/v).

Isolated Yield: 74%

^{1}H NMR (400 MHz, CDCl$_3$) δ 8.26 (t, *J* = 1.6 Hz, 1H), 8.03 (dt, *J* = 7.8, 1.4 Hz, 1H), 7.78 (ddd, *J* = 7.7, 1.9, 1.2 Hz, 1H), 7.60 (d, *J* = 8.8 Hz, 1H), 7.57 – 7.48 (m, 2H), 7.44 (dd, *J* = 8.2, 7.6 Hz, 1H), 7.31 (dt, *J* = 7.7, 1.4 Hz, 1H), 7.05 (d, *J* = 2.4 Hz, 1H), 6.91 (dd, *J* = 8.8, 2.4 Hz, 1H), 3.96 (s, 3H), 3.89 (s, 3H), 3.82 – 3.71 (m, 2H), 3.51 – 3.43 (m, 2H).

^{13}C NMR (101 MHz, CDCl$_3$) δ 167.20, 164.32, 151.54, 141.08, 137.57, 134.66, 131.75, 130.92, 129.78, 129.30, 129.14, 128.79, 128.42, 128.07, 127.90, 127.59, 126.37, 115.63, 114.09, 109.64, 98.76, 56.34, 53.86, 52.43, 29.98.

HRMS: [ESI, (+) ve]: Cald. 508.0592 for $C_{24}H_{20}NClNaO_6S$ observed 508.0597.

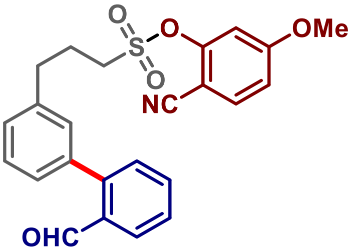

2-(((3-(2'-formyl-[1,1'-biphenyl]-3-yl)propyl)sulfonyl)methyl)-4-methoxybenzonitrile (3jk): Compound 3jk was synthesized using the general procedure K.

Column material: 100-200 mesh silica

Eluent: petroleum ether/ethyl acetate (87:13, v/v).

Isolated Yield: 65%

^{1}H NMR (500 MHz, CDCl$_3$) δ 9.98 (s, 1H), 8.02 (dd, *J* = 7.8, 1.6 Hz, 1H), 7.71 – 7.54 (m, 2H), 7.50 (q, *J* = 6.8 Hz, 1H), 7.43 (dd, *J* = 14.0, 7.1 Hz, 1H), 7.34 (s, 2H), 7.26 (s, 2H), 7.03 (d, *J* = 2.1 Hz, 1H), 6.91 (dd, *J* = 8.7, 2.5 Hz, 1H), 3.88 (t, *J* = 2.4 Hz, 3H), 3.49 (dd, *J* = 9.2, 6.5 Hz, 2H), 2.94 (t, *J* = 7.5 Hz, 2H), 2.47 (p, *J* = 7.6 Hz, 2H).

^{13}C NMR (126 MHz, CDCl$_3$) δ 192.66, 164.32, 151.53, 145.81, 139.76, 136.29, 134.66, 133.89, 133.79, 130.97, 130.67, 130.30, 128.80, 128.44, 127.93, 127.80, 115.60, 114.03, 109.70, 98.75, 56.32, 51.79, 33.73, 25.17.

HRMS: [ESI, (+) ve]: Cald. 434.1421 for $C_{25}H_{24}NO_4S$ observed 434.1427.

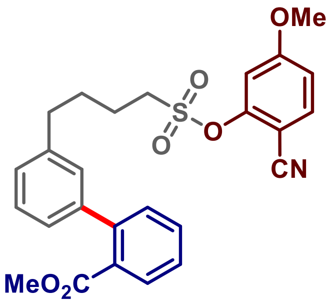

methyl 3'-(4-((2-cyano-5-methoxybenzyl)sulfonyl)butyl)-[1,1'-biphenyl]-2-carboxylate (3kc): Compound 3kc was synthesized using the general procedure K.

Column material: 100-200 mesh silica

Eluent: petroleum ether/ethyl acetate (87:13, v/v).

Isolated Yield: 61%

^{1}H NMR (400 MHz, CDCl$_3$) δ 7.80 (dd, *J* = 7.7, 1.4 Hz, 1H), 7.58 (dd, *J* = 8.8, 4.9 Hz, 1H), 7.54 – 7.48 (m, 1H), 7.45 – 7.35 (m, 2H), 7.32 (t, *J* = 7.9 Hz, 1H), 7.23 (d, *J* = 4.7 Hz, 1H), 7.20 – 7.10 (m, 2H), 7.02 (dd, *J* = 4.0, 2.4 Hz, 1H), 6.89 (dt, *J* = 8.8, 2.9 Hz, 1H), 3.87 (s, 3H), 3.64 (s, 3H), 3.54 – 3.41 (m, 2H), 2.74 (td, *J* = 7.6, 2.7 Hz, 2H), 2.23 – 2.04 (m, 2H), 1.89 (dt, *J* = 15.4, 6.0 Hz, 2H).

^{13}C NMR (101 MHz, CDCl$_3$) δ 164.27, 151.55, 142.53, 141.64, 141.05, 140.23, 134.62, 131.40, 130.86, 129.87, 128.61, 128.51, 128.36, 128.28, 127.48, 127.30, 126.35, 113.98, 109.63, 98.78, 56.29, 52.42, 52.15, 35.30, 35.02, 29.98, 23.28.

HRMS: [ESI, (+) ve]: Cald. 478.1683 for $C_{27}H_{28}NO_5S$ observed 478.1685.

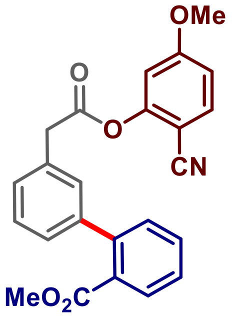

methyl 3'-(2-(2-cyano-5-methoxyphenoxy)-2-oxoethyl)-[1,1'-biphenyl]-2-carboxylate (5ac): Compound 5ac was synthesized using the general procedure K.

Column material: 100-200 mesh silica

Eluent: petroleum ether/ethyl acetate (88:12, v/v).

Isolated Yield: 80%

^{1}H NMR (400 MHz, CDCl$_3$) δ 7.80 (dd, *J* = 8.3, 1.4 Hz, 1H), 7.53 (d, *J* = 8.6 Hz, 1H), 7.52 – 7.47 (m, 1H), 7.47 – 7.34 (m, 4H), 7.31 (s, 1H), 7.27 – 7.20 (m, 1H), 6.80 (dd, *J* = 8.7, 2.5 Hz, 1H), 6.76 (d, *J* = 2.5 Hz, 1H), 3.97 (s, 2H), 3.81 (s, 3H), 3.60 (s, 3H).

^{13}C NMR (101 MHz, CDCl$_3$) δ 169.22, 169.08, 164.09, 154.17, 142.16, 142.03, 134.27, 132.51, 131.51, 130.98, 130.96, 129.98, 129.64, 128.70, 128.60, 127.83, 127.51, 115.69, 112.89, 109.08, 98.80, 56.10, 52.19, 41.11.

HRMS [ESI, (+) ve]: calcd. 424.1155 for $C_{24}H_{19}NNaO_5$ observed 424.1150.

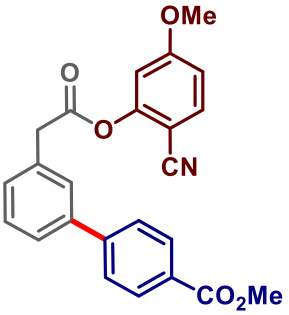

methyl 3'-(2-(2-cyano-5-methoxyphenoxy)-2-oxoethyl)-[1,1'-biphenyl]-4-carboxylate (5ae): Compound 5ae was synthesized using the general procedure K.

Column material: 100-200 mesh silica

Eluent: petroleum ether/ethyl acetate (88:12, v/v).

Isolated Yield: 68%

^{1}H NMR (500 MHz, CDCl$_3$) δ 7.70 (d, *J* = 8.0 Hz, 2H), 7.35 (s, 1H), 7.26 (t, *J* = 8.1 Hz, 3H), 7.21 – 7.08 (m, 3H), 6.50 (d, *J* = 2.4 Hz, 1H), 6.47 (dd, *J* = 8.7, 2.4 Hz, 1H), 4.38 (s, 2H), 3.53 (s, 3H), 3.42 (s, 3H).

^{13}C NMR (126 MHz, CDCl$_3$) δ 167.07, 164.32, 151.90, 144.65, 141.19, 134.60, 130.90, 130.38, 130.11, 129.92, 129.55, 128.66, 127.36, 127.31, 115.71, 114.08, 109.39, 98.81, 58.69, 56.30, 52.37.

HRMS [ESI, (+) ve]: calcd. 424.1155 for $C_{24}H_{19}NNaO_5$ observed 424.1150.

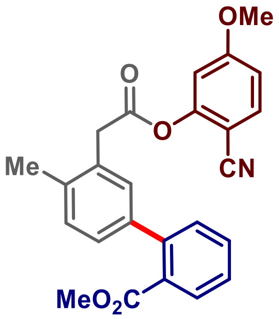

methyl 3'-(2-(2-cyano-5-methoxyphenoxy)-2-oxoethyl)-4'-methyl-[1,1'-biphenyl]-2-carboxylate (5bc): Compound 5bc was synthesized using the general procedure K.

Column material: 100-200 mesh silica

Eluent: petroleum ether/ethyl acetate (88:12, v/v).

Isolated Yield: 78%

^{1}H NMR (400 MHz, CDCl$_3$) δ 7.81 (dd, J = 8.2, 1.3 Hz, 1H), 7.55 (d, J = 8.7 Hz, 1H), 7.53 – 7.49 (m, 1H), 7.39 (dd, J = 8.1, 6.8 Hz, 2H), 7.29 – 7.24 (m, 2H), 7.20 (dd, J = 7.8, 1.9 Hz, 1H), 6.82 (dd, J = 8.7, 2.4 Hz, 1H), 6.78 (d, J = 2.4 Hz, 1H), 4.00 (s, 2H), 3.83 (s, 3H), 3.64 (s, 3H), 2.46 (s, 3H).

^{13}C NMR (101 MHz, CDCl$_3$) δ 169.39, 168.97, 164.11, 154.17, 142.04, 139.47, 136.36, 134.29, 131.45, 131.28, 131.02, 130.96, 130.56, 130.52, 129.91, 127.99, 127.30, 115.67, 112.88, 109.14, 98.87, 56.10, 52.20, 39.04, 19.67.

HRMS (ESI): calcd. 416.1498 for $C_{25}H_{22}NO_5$ observed 416.149.

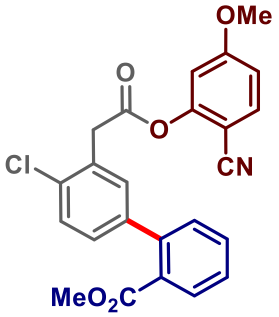

methyl 4'-chloro-3'-(2-(2-cyano-5-methoxyphenoxy)-2-oxoethyl)-[1,1'-biphenyl]-2-carboxylate (5cc): Compound 5cc was synthesized using the general procedure K.

Column material: 100-200 mesh silica

Eluent: petroleum ether/ethyl acetate (88:12, v/v).

Isolated Yield: 73%

^{1}H NMR (400 MHz, CDCl$_3$) δ 7.86 (dd, *J* = 7.7, 1.4 Hz, 1H), 7.60 – 7.51 (m, 2H), 7.44 (td, *J* = 7.9, 6.4 Hz, 2H), 7.39 – 7.33 (m, 2H), 7.22 (dd, *J* = 8.2, 2.2 Hz, 1H), 6.89 – 6.79 (m, 2H), 4.14 (s, 2H), 3.85 (s, 3H), 3.66 (s, 3H).

^{13}C NMR (101 MHz, CDCl$_3$) δ 168.81, 168.16, 164.09, 154.07, 141.13, 140.75, 134.30, 133.92, 131.85, 131.72, 130.97, 130.68, 130.26, 129.38, 127.86, 115.60, 112.94, 109.13, 98.79, 56.11, 52.28, 39.21.

HRMS (ESI): calcd. 458.0766 for $C_{24}H_{18}ClNNaO_5$ observed 458.0759.

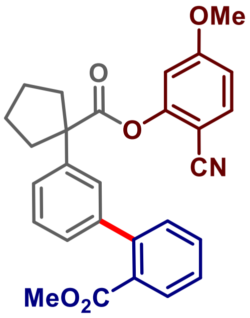

methyl 3'-(1-((2-cyano-5-methoxyphenoxy)carbonyl)cyclopentyl)-[1,1'-biphenyl]-2-carboxylate (5dc): Compound 5dc was synthesized using the general procedure K.

Column material: 100-200 mesh silica

Eluent: petroleum ether/ethyl acetate (85:15, v/v).

Isolated Yield: 77%

^{1}H NMR (500 MHz, CDCl$_3$) δ 7.81 (dd, *J* = 8.0, 1.4 Hz, 1H), 7.60 – 7.47 (m, 3H), 7.48 – 7.37 (m, 4H), 7.32 – 7.26 (m, 1H), 6.77 (dd, *J* = 8.7, 2.5 Hz, 1H), 6.57 (d, *J* = 2.4 Hz, 1H), 3.77 (s, 3H), 3.61 (s, 3H), 2.96 – 2.78 (m, 2H), 2.18 – 2.06 (m, 2H), 1.99 – 1.69 (m, 4H).

^{13}C NMR (126 MHz, CDCl$_3$) δ 173.65, 169.17, 164.03, 154.41, 142.35, 141.97, 141.55, 134.14, 131.43, 131.14, 130.90, 129.85, 128.57, 127.47, 127.44, 127.33, 126.14, 115.61, 112.70, 108.86, 98.85, 59.54, 56.03, 52.17, 36.32, 23.73.

HRMS (ESI): calcd. 478.1625 for $C_{28}H_{25}NNaO_5$ observed 478.1632.

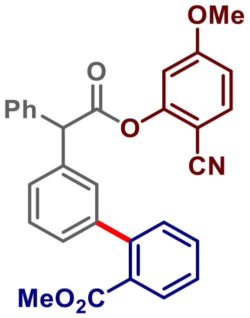

methyl 3'-(2-(2-cyano-5-methoxyphenoxy)-2-oxo-1-phenylethyl)-[1,1'-biphenyl]-2-carboxylate (5ec): Compound 5ec was synthesized using the general procedure K.

Column material: 100-200 mesh silica

Eluent: petroleum ether/ethyl acetate (85:15, v/v).

Isolated Yield: 47%

^{1}H NMR (400 MHz, CDCl$_3$) δ 8.26 (t, *J* = 1.8 Hz, 1H), 8.02 (dt, *J* = 7.9, 1.3 Hz, 1H), 7.90 – 7.72 (m, 1H), 7.67 (d, *J* = 2.1 Hz, 1H), 7.61 – 7.44 (m, 7H), 7.45 – 7.30 (m, 3H), 6.83 (dd, *J* = 8.7, 2.4 Hz, 1H), 6.74 (d, *J* = 2.4 Hz, 1H), 5.44 (s, 1H), 3.94 (s, 3H), 3.82 (s, 3H).

^{13}C NMR (101 MHz, CDCl$_3$) δ 170.15, 167.21, 164.04, 154.03, 141.22, 140.96, 138.24, 137.42, 134.43, 131.92, 130.87, 129.63, 129.14, 129.08, 128.92, 128.74, 128.50, 128.30, 128.04, 127.85, 126.85, 115.66, 112.82, 108.99, 98.90, 57.06, 56.11, 52.41.

HRMS (ESI): calcd. 477.1576 for $C_{30}H_{23}NO_5$ observed 477.1578.

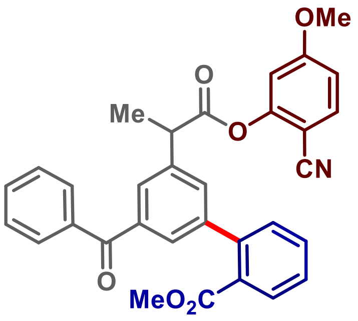

methyl 3'-benzoyl-5'-(1-(2-cyano-5-methoxyphenoxy)-1-oxopropan-2-yl)-[1,1'-biphenyl]-2-carboxylate (5fc): Compound 5fc was synthesized using the general procedure K.

Column material: 100-200 mesh silica

Eluent: petroleum ether/ethyl acetate (80:20, v/v).

Isolated Yield: 63%

^{1}H NMR (400 MHz, CDCl$_3$) δ 7.96 – 7.81 (m, 4H), 7.68 (t, J = 1.6 Hz, 1H), 7.60 (dd, J = 3.6, 1.8 Hz, 1H), 7.58 – 7.49 (m, 4H), 7.47 (dt, J = 8.0, 1.4 Hz, 1H), 7.42 (dq, J = 9.9, 2.2 Hz, 2H), 6.82 (dd, J = 8.7, 2.5 Hz, 1H), 6.75 (d, J = 2.4 Hz, 1H), 4.17 (q, J = 7.1 Hz, 1H), 3.83 (s, 3H), 3.68 (s, 3H), 1.75 (d, J = 7.2 Hz, 3H).

^{13}C NMR (101 MHz, CDCl$_3$) δ 196.46, 171.70, 168.53, 164.12, 154.04, 142.23, 141.54, 139.35, 138.13, 137.56, 134.25, 132.80, 131.97, 131.82, 131.19, 130.59, 130.36, 130.33, 129.75, 128.55, 128.38, 127.98, 115.53, 112.99, 109.01, 98.81, 56.14, 52.28, 45.50, 18.59.

HRMS (ESI): calcd. 520.1755 for $C_{32}H_{26}NO_6$ observed 520.1761.

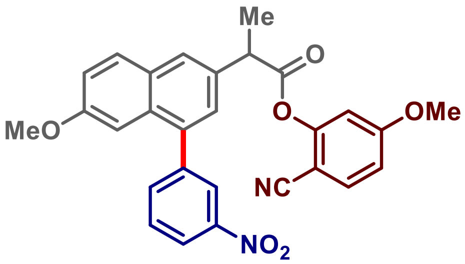

2-cyano-5-methoxyphenyl 2-(6-methoxy-4-(3-nitrophenyl)naphthalen-2-yl)propanoate (5gc): Compound 5gc was synthesized using the general procedure K.

Column material: 100-200 mesh silica

Eluent: petroleum ether/ethyl acetate (80:20, v/v).

Isolated Yield: 65%

^{1}H NMR (400 MHz, CDCl$_3$) δ 8.49 (t, *J* = 2.0 Hz, 1H), 8.23 (ddd, *J* = 8.3, 2.4, 1.1 Hz, 1H), 7.99 – 7.71 (m, 5H), 7.63 – 7.56 (m, 2H), 7.54 (d, *J* = 8.7 Hz, 1H), 6.80 (dd, *J* = 8.7, 2.5 Hz, 1H), 6.68 (d, *J* = 2.4 Hz, 1H), 4.21 (q, *J* = 7.2 Hz, 1H), 3.95 (s, 3H), 3.80 (s, 3H), 1.78 (d, *J* = 7.2 Hz, 3H).

^{13}C NMR (101 MHz, CDCl$_3$) δ 172.13, 164.03, 155.04, 154.15, 148.27, 140.04, 136.02, 135.15, 134.33, 134.01, 130.44, 129.07, 128.88, 127.42, 127.09, 126.81, 124.93, 122.29, 115.71, 112.66, 109.00, 106.09, 98.76, 56.07, 55.86, 45.71, 18.61.

HRMS (ESI): calcd. 483.1551 for $C_{28}H_{23}NO_6$ observed 483.1553.

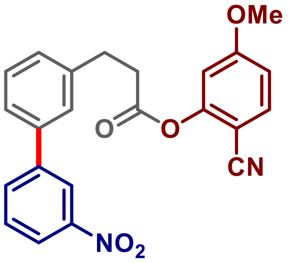

2-cyano-5-methoxyphenyl 3-(3'-nitro-[1,1'-biphenyl]-3-yl)propanoate (5ha): Compound 5ha was synthesized using the general procedure K.

Column material: 100-200 mesh silica

Eluent: petroleum ether/ethyl acetate (85:15, v/v).

Isolated Yield: 60%

^{1}H NMR (500 MHz, CDCl$_3$) δ 8.45 (s, 1H), 8.21 (ddd, *J* = 8.1, 2.4, 0.9 Hz, 1H), 7.92 (dt, *J* = 7.7, 1.4 Hz, 1H), 7.59 (dd, *J* = 17.4, 8.3 Hz, 2H), 7.55 – 7.45 (m, 3H), 7.38 – 7.34 (m, 1H), 6.83 (dd, *J* = 8.7, 2.5 Hz, 1H), 6.70 (d, *J* = 2.4 Hz, 1H), 3.83 (s, 3H), 3.21 (t, *J* = 7.7 Hz, 2H), 3.05 (dd, *J* = 8.8, 6.6 Hz, 2H).

^{13}C NMR (126 MHz, CDCl$_3$) δ 170.29, 164.13, 154.03, 142.91, 141.00, 139.29, 134.36, 133.33, 129.92, 129.71, 129.43, 128.76, 127.75, 127.55, 125.67, 122.31, 122.19, 115.76, 112.72, 109.23, 56.07, 35.85, 30.88.

HRMS [ESI, (+) ve]: calcd. 425.1108 for $C_{23}H_{18}N_2NaO_5$ observed 425.1109.

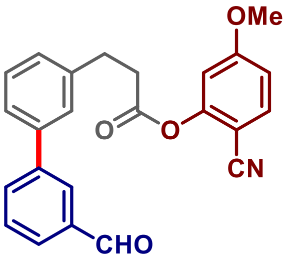

2-cyano-5-methoxyphenyl 3-(3'-formyl-[1,1'-biphenyl]-3-yl)propanoate (5hm): Compound 5hm was synthesized using the general procedure K.

Column material: 100-200 mesh silica

Eluent: petroleum ether/ethyl acetate (85:15, v/v).

Isolated Yield: 64%

^{1}H NMR (500 MHz, CDCl$_3$) δ 10.09 (s, 1H), 8.10 (d, *J* = 1.8 Hz, 1H), 7.87 (dd, *J* = 7.6, 1.8 Hz, 2H), 7.61 (t, *J* = 7.6 Hz, 1H), 7.58 – 7.48 (m, 3H), 7.44 (t, *J* = 7.6 Hz, 1H), 7.32 (d, *J* = 7.6 Hz, 1H), 6.83 (dd, *J* = 8.7, 2.4 Hz, 1H), 6.69 (d, *J* = 2.4 Hz, 1H), 3.81 (d, *J* = 2.1 Hz, 3H), 3.20 (t, *J* = 7.7 Hz, 2H), 3.04 (t, *J* = 7.7 Hz, 2H).

^{13}C NMR (126 MHz, CDCl$_3$) δ 192.58, 170.35, 164.12, 154.07, 142.16, 140.76, 140.26, 137.08, 134.30, 133.31, 129.70, 129.53, 128.83, 128.46, 128.23, 127.47, 125.61, 115.74, 112.76, 109.18, 101.72, 98.79, 56.04, 35.87, 30.93.

HRMS [ESI, (+) ve]: calcd. 408.1206 for $C_{24}H_{19}NNaO_4$ observed 408.1201.

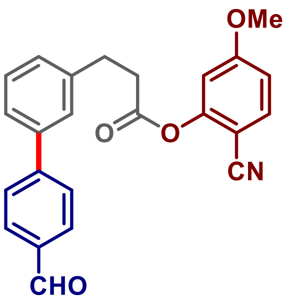

2-cyano-5-methoxyphenyl 3-(4'-formyl-[1,1'-biphenyl]-3-yl)propanoate (5hn): Compound 5hn was synthesized using the general procedure K.

Column material: 100-200 mesh silica

Eluent: petroleum ether/ethyl acetate (85:15, v/v).

Isolated Yield: 61%

^{1}H NMR (500 MHz, CDCl$_3$) δ 10.06 (s, 1H), 7.95 (d, J = 8.2 Hz, 2H), 7.76 (d, J = 8.2 Hz, 2H), 7.60 – 7.48 (m, 3H), 7.44 (t, J = 7.6 Hz, 1H), 7.38 – 7.30 (m, 1H), 6.83 (dd, J = 8.7, 2.4 Hz, 1H), 6.69 (d, J = 2.4 Hz, 1H), 3.81 (s, 3H), 3.20 (t, J = 7.8 Hz, 2H), 3.04 (t, J = 7.7 Hz, 2H).

^{13}C NMR (126 MHz, CDCl$_3$) δ 192.23, 170.33, 164.13, 154.04, 147.23, 140.77, 140.27, 135.39, 134.31, 130.47, 129.54, 128.69, 127.91, 127.68, 125.84, 112.74, 109.19, 101.73, 98.76, 56.04, 35.84, 30.90.

HRMS [ESI, (+) ve]: calcd. 408.1206 for $C_{24}H_{19}NNaO_4$ observed 408.1210.

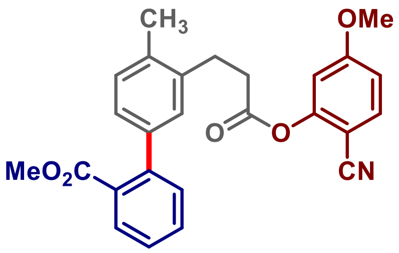

methyl 3'-(2-(2-cyano-5-methoxyphenoxy)-2-oxoethyl)-4'-methyl-[1,1'-biphenyl]-2-carboxylate (5ic): Compound 5ic was synthesized using the general procedure K.

Column material: 100-200 mesh silica

Eluent: petroleum ether/ethyl acetate (86:14, v/v).

Isolated Yield: 88%

^{1}H NMR (400 MHz, $CDCl_3$) δ 7.81 (dd, J = 8.2, 1.3 Hz, 1H), 7.55 (d, J = 8.7 Hz, 1H), 7.53 – 7.49 (m, 1H), 7.39 (dd, J = 8.1, 6.8 Hz, 2H), 7.29 – 7.24 (m, 2H), 7.20 (dd, J = 7.8, 1.9 Hz, 1H), 6.82 (dd, J = 8.7, 2.4 Hz, 1H), 6.78 (d, J = 2.4 Hz, 1H), 3.81 (s, 3H), 3.67 (s, 3H), 3.16 (t, J = 7.8 Hz, 2H), 2.96(t, J = 7.8 Hz, 2H) 2.42 (s, 3H).

^{13}C NMR (101 MHz, $CDCl_3$) δ 169.39, 168.97, 164.11, 154.17, 142.04, 139.47, 136.36, 134.29, 131.45, 131.28, 131.02, 130.96, 130.56, 130.52, 129.91, 127.99, 127.30, 115.67, 112.88, 109.14, 98.87, 56.10, 52.20, 39.04, 19.67.

HRMS (ESI): calcd. 430.1649 for $C_{26}H_{24}NO_5$ observed 430.1649.

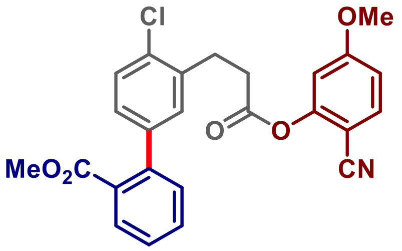

methyl 4'-chloro-3'-(3-(2-cyano-5-methoxyphenoxy)-3-oxopropyl)-[1,1'-biphenyl]-2-carboxylate (5jc): Compound 5jc was synthesized using the general procedure K.

Column material: 100-200 mesh silica

Eluent: petroleum ether/ethyl acetate (86:14, v/v).

Isolated Yield: 82%

^{1}H NMR (500 MHz, $CDCl_3$) δ 7.85 (dd, J = 7.7, 1.4 Hz, 1H), 7.57 – 7.51 (m, 2H), 7.44 – 7.39 (m, 2H), 7.33 (dd, J = 7.6, 1.3 Hz, 1H), 7.28 (d, J = 2.2 Hz, 1H), 7.14 (dd, J = 8.2, 2.3 Hz, 1H), 6.82 (dd, J = 8.7, 2.4 Hz, 1H), 6.76 (d, J = 2.4 Hz, 1H), 3.81 (s, 3H), 3.67 (s, 3H), 3.25 (t, J = 7.8 Hz, 2H), 3.03 (dd, J = 8.5, 7.1 Hz, 2H).

^{13}C NMR (126 MHz, $CDCl_3$) δ 170.17, 168.77, 164.11, 154.11, 141.43, 140.59, 137.17, 134.25, 133.29, 131.65, 130.92, 130.70, 130.68, 130.20, 129.43, 128.36, 127.73, 115.71, 112.84, 109.20, 98.82, 56.06, 52.24, 34.05, 28.97.

HRMS (ESI): calcd. 450.1108 for $C_{25}H_{21}ClNO_5$ observed 450.1112.

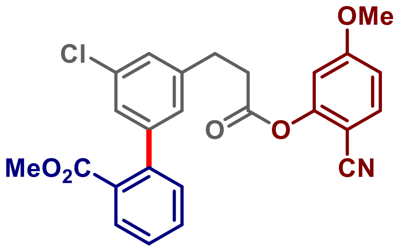

methyl 3'-chloro-5'-(3-(2-cyano-5-methoxyphenoxy)-3-oxopropyl)-[1,1'-biphenyl]-2-carboxylate (5kc): Compound 5kc was synthesized using the general procedure K.

Column material: 100-200 mesh silica

Eluent: petroleum ether/ethyl acetate (85:15, v/v).

Isolated Yield: 75%

^{1}H NMR (500 MHz, $CDCl_3$) 7.85 (dd, J = 7.5, 1.4 Hz, 1H), 7.58 – 7.50 (m, 2H), 7.47 – 7.40 (m, 1H), 7.34 (d, J = 7.6 Hz, 1H), 7.27 – 7.24 (m, 1H), 7.19 (d, J = 1.9 Hz, 1H), 7.09 (d, J = 1.8 Hz, 1H), 6.83 (dd, J = 8.7, 2.5 Hz, 1H), 6.72 (d, J = 2.5 Hz, 1H), 3.83 (s, 3H), 3.68 – 3.64 (m, 3H), 3.12 (t, J = 7.7 Hz, 2H), 2.99 (t, J = 7.7 Hz, 2H).

^{13}C NMR (126 MHz, $CDCl_3$) δ 170.06, 168.70, 164.16, 154.07, 143.61, 141.47, 141.19, 134.26, 134.15, 131.67, 130.83, 130.78, 130.22, 127.95, 127.51, 127.01, 126.93, 115.72, 112.96, 109.14, 98.81, 56.10, 52.28, 35.60, 30.53.

HRMS (ESI): calcd. 450.1108 for $C_{25}H_{21}ClNO_5$ observed 450.1110.

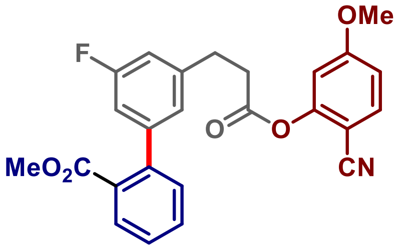

methyl 3'-(3-(2-cyano-5-methoxyphenoxy)-3-oxopropyl)-5'-fluoro-[1,1'-biphenyl]-2-carboxylate (5lc): Compound 5lc was synthesized using the general procedure K.

Column material: 100-200 mesh silica

Eluent: petroleum ether/ethyl acetate (85:15, v/v).

Isolated Yield: 74%

^{1}H NMR (500 MHz, $CDCl_3$) δ 7.84 (dd, J = 7.7, 1.4 Hz, 1H), 7.59 – 7.50 (m, 2H), 7.43 (td, J = 7.6, 1.3 Hz, 1H), 7.35 (dd, J = 7.8, 1.3 Hz, 1H), 7.01 – 6.95 (m, 2H), 6.91 (dt, J = 9.3, 2.2 Hz, 1H), 6.83 (dd, J = 8.7, 2.4 Hz, 1H), 6.72 (d, J = 2.4 Hz, 1H), 3.83 (s, 3H), 3.67 (s, 3H), 3.14 (t, J = 7.7 Hz, 2H), 3.03 – 2.96 (m, 2H).

^{13}C NMR (126 MHz, $CDCl_3$) δ 170.14, 168.83, 164.17, 163.72, 161.76, 154.07, 143.99, 143.92, 142.01, 141.95, 141.36, 134.30, 133.91, 131.64, 130.87, 130.80, 130.15, 127.91, 124.42, 124.40, 115.75, 114.45, 114.28, 113.97, 113.80, 112.94, 109.16, 98.81, 56.10, 52.31, 35.60, 30.63.

^{19}**F NMR (377 MHz, CDCl$_3$)** δ -113.79.

HRMS (ESI): calcd. 434.1404 for $C_{25}H_{21}FNO_5$ observed 434.1405.

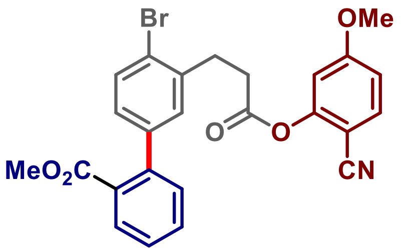

methyl 4'-bromo-3'-(3-(2-cyano-5-methoxyphenoxy)-3-oxopropyl)-[1,1'-biphenyl]-2-carboxylate (5mc): Compound 5mc was synthesized using the general procedure K.

Column material: 100-200 mesh silica

Eluent: petroleum ether/ethyl acetate (86:14, v/v).

Isolated Yield: 81%

^{1}**H NMR (400 MHz, CDCl$_3$)** δ 7.85 (dd, J = 7.6, 1.4 Hz, 1H), 7.62 – 7.49 (m, 3H), 7.42 (td, J = 7.6, 1.3 Hz, 1H), 7.36 – 7.30 (m, 1H), 7.28 (d, J = 2.2 Hz, 1H), 7.06 (dd, J = 8.2, 2.3 Hz, 1H), 6.83 (dd, J = 8.7, 2.4 Hz, 1H), 6.76 (d, J = 2.4 Hz, 1H), 3.82 (s, 3H), 3.67 (s, 3H), 3.30 – 3.21 (m, 2H), 3.07 – 2.99 (m, 2H).

^{13}**C NMR (101 MHz, CDCl$_3$)** δ 170.16, 168.77, 164.12, 154.11, 141.42, 141.28, 138.90, 134.27, 132.77, 131.71, 130.90, 130.68, 130.61, 130.25, 128.62, 127.78, 123.59, 115.74, 112.87, 109.21, 98.82, 56.09, 52.29, 34.20, 31.43.

HRMS (ESI): calcd. 494.0603 for $C_{25}H_{21}BrNO_5$ observed 494.0600.

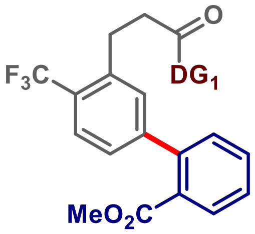

methyl 3'-(3-(2-cyano-5-methoxyphenoxy)-3-oxopropyl)-4'-(trifluoromethyl)-[1,1'-biphenyl]-2-carboxylate (5nc): Compound 5nc was synthesized using the general procedure K.

Column material: 100-200 mesh silica

Eluent: petroleum ether/ethyl acetate (86:14, v/v).

Isolated Yield: 46%

^{1}**H NMR (400 MHz, CDCl$_3$)** δ 7.91 (d, *J* = 7.2 Hz, 1H), 7.68 (d, *J* = 8.1 Hz, 1H), 7.61 – 7.54 (m, 1H), 7.55 (s, 1H), 7.46 (t, *J* = 7.8 Hz, 1H), 7.37 (s, 1H), 7.34 (d, *J* = 7.7 Hz, 1H), 7.28 (d, *J* = 8.9 Hz, 1H), 6.83 (dd, *J* = 8.7, 2.3 Hz, 1H), 6.77 (d, *J* = 2.4 Hz, 1H), 3.83 (s, 3H), 3.68 (s, 3H), 3.32 (t, *J* = 8.0 Hz, 2H), 3.00 (t, *J* = 8.0 Hz, 2H).

^{13}C NMR (101 MHz, CDCl$_3$) δ 170.0, 168.4, 164.2, 154.1, 145.7, 141.4, 138.4, 134.3, 131.9, 131.4, 131.0, 130.5, 130.5, 128.2, 127.7 (d, $J_{C\text{–}F}$ = 30.3 Hz), 127.0, 126.2 (dd, $J_{C\text{–}F}$ = 11.6, 5.8 Hz), 115.7, 112.9, 109.2, 98.9, 56.1, 52.3, 35.9, 27.8.

^{19}F NMR (376 MHz, CDCl$_3$) δ -59.4

HRMS (ESI): calcd. 506.1191 for $C_{26}H_{20}F_3NNaO_5$ observed 506.1190.

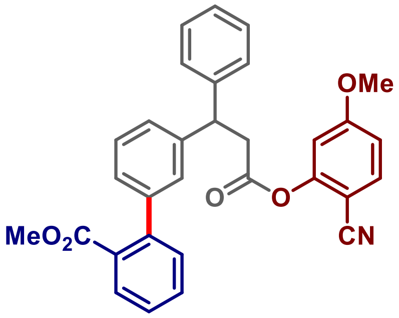

methyl 3'-(3-(2-cyano-5-methoxyphenoxy)-3-oxo-1-phenylpropyl)-[1,1'-biphenyl]-2-carboxylate (5oc): Compound 5oc was synthesized using the general procedure K.

Column material: 100-200 mesh silica

Eluent: petroleum ether/ethyl acetate (83:17, v/v).

Isolated Yield: 59%

^{1}H NMR (400 MHz, CDCl$_3$) δ 7.79 (dd, J = 7.6, 1.4 Hz, 1H), 7.53 – 7.48 (m, 2H), 7.40 (dd, J = 8.5, 7.0 Hz, 2H), 7.35 (d, J = 4.0 Hz, 3H), 7.33 (s, 2H), 7.25 – 7.18 (m, 4H), 6.77 (dd, J = 8.8, 2.5 Hz, 1H), 6.32 (d, J = 2.5 Hz, 1H), 4.71 (t, J = 8.2 Hz, 1H), 3.73 (s, 3H), 3.46 (s, 3H), 3.43 (d, J = 8.3 Hz, 2H).

^{13}C NMR (126 MHz, CDCl$_3$) δ 169.34, 169.28, 163.99, 154.00, 142.86, 142.71, 142.26, 141.97, 134.15, 131.42, 131.20, 130.86, 129.94, 128.98, 128.81, 128.72, 128.08, 128.00, 127.83, 127.46, 127.10, 127.04, 126.74, 115.71, 112.97, 108.88, 98.70, 56.04, 52.06, 47.25, 40.72.

HRMS (ESI): calcd. 492.1811 for $C_{31}H_{26}NO_5$ observed 492.1810.

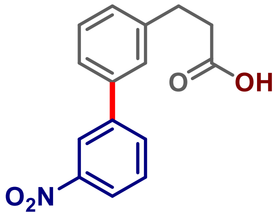

3-(3'-nitro-[1,1'-biphenyl]-3-yl)propanoic acid (6aa): Compound 6aa was synthesized using the general procedure K followed by general procedure L.

Column material: 100-200 mesh silica

Eluent: petroleum ether/ethyl acetate (60:40, v/v).

Isolated Yield: 66% (over two steps)

^{1}H NMR (500 MHz, $CDCl_3$) δ 8.41 (d, *J* = 2.7 Hz, 1H), 8.18 (d, *J* = 8.2 Hz, 1H), 7.89 (d, *J* = 7.7 Hz, 1H), 7.59 (td, *J* = 8.0, 1.8 Hz, 1H), 7.46 (d, *J* = 6.3 Hz, 2H), 7.42 (td, *J* = 7.9, 1.9 Hz, 1H), 7.28 (d, *J* = 7.5 Hz, 1H), 3.05 (t, *J* = 7.7 Hz, 2H), 2.76 (t, *J* = 7.7 Hz, 2H).

^{13}C NMR (126 MHz, $CDCl_3$) δ 179.19, 148.78, 142.83, 141.34, 139.05, 133.19, 129.82, 129.52, 128.59, 127.31, 125.40, 122.17, 122.03, 35.65, 30.64.

HRMS [ESI, (+) ve]: calcd. 272.0917 for $C_{15}H_{14}NO_4$ observed 272.0925.

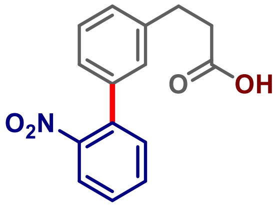

3-(2'-nitro-[1,1'-biphenyl]-3-yl)propanoic acid (6ao): Compound 6ao was synthesized using the general procedure K followed by general procedure L.

Column material: 100-200 mesh silica

Eluent: petroleum ether/ethyl acetate (60:40, v/v).

Isolated Yield: 67% (over two steps)

^{1}H NMR (500 MHz, $CDCl_3$) δ 7.93 (d, *J* = 8.1 Hz, 1H), 7.69 (t, *J* = 7.6 Hz, 1H), 7.56 (t, *J* = 7.8 Hz, 1H), 7.51 (d, *J* = 7.7 Hz, 1H), 7.44 (t, *J* = 7.8 Hz, 1H), 7.33 (d, *J* = 7.4 Hz, 1H), 7.25 (d, *J* = 6.2 Hz, 2H), 3.08 (t, *J* = 7.8 Hz, 2H), 2.79 (t, *J* = 7.8 Hz, 2H).

^{13}C NMR (126 MHz, $CDCl_3$) δ 178.71, 149.43, 140.85, 137.80, 136.39, 132.44, 132.10, 129.09, 128.35, 128.03, 126.16, 124.24, 35.59, 30.64.

HRMS [ESI, (+) ve]: calcd. 272.0917 for $C_{15}H_{14}NO_4$ observed 272.0928.

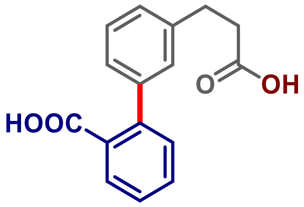

3'-(2-carboxyethyl)-[1,1'-biphenyl]-2-carboxylic acid (6ac): Compound 6ac was synthesized using the general procedure K followed by general procedure L.

Column material: 100-200 mesh silica

Eluent: DCM/MeOH (95:5, v/v).

Isolated Yield: 69% (over two steps)

^{1}H NMR (500 MHz, DMSO) δ 7.68 (d, *J* = 7.6 Hz, 1H), 7.54 (t, *J* = 7.5 Hz, 1H), 7.42 (t, *J* = 7.5 Hz, 1H), 7.36 (d, *J* = 7.6 Hz, 1H), 7.29 (t, *J* = 7.5 Hz, 1H), 7.21 – 7.16 (m, 2H), 7.14 (d, *J* = 7.6 Hz, 1H), 2.84 (t, *J* = 7.7 Hz, 2H), 2.54 (t, *J* = 7.7 Hz, 2H).

^{13}C NMR (126 MHz, DMSO) δ 174.38, 170.37, 141.23, 141.18, 141.15, 132.79, 131.31, 130.90, 129.45, 128.67, 128.63, 127.71, 127.60, 126.53, 35.68, 30.75.

HRMS [ESI, (+) ve]: calcd. 293.0784 for $C_{16}H_{14}NaO_4$ observed 293.0790.

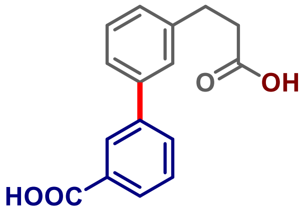

3'-(2-carboxyethyl)-[1,1'-biphenyl]-3-carboxylic acid (6ad): Compound 6ad was synthesized using the general procedure K followed by general procedure L.

Column material: 100-200 mesh silica

Eluent: DCM/MeOH (95:5, v/v).

Isolated Yield: 71% (over two steps)

^{1}H NMR (400 MHz, MeOD) δ 8.13 (t, *J* = 1.8 Hz, 1H), 7.87 (dt, *J* = 7.7, 1.4 Hz, 1H), 7.72 (ddd, *J* = 7.8, 2.0, 1.2 Hz, 1H), 7.46 – 7.38 (m, 2H), 7.35 (dt, *J* = 7.7, 1.5 Hz, 1H), 7.26 (t, *J* = 7.6 Hz, 1H), 7.14 (dt, *J* = 7.6, 1.5 Hz, 1H), 2.88 (t, *J* = 7.6 Hz, 2H), 2.54 (t, *J* = 7.6 Hz, 2H).

^{13}C NMR (101 MHz, MeOD) δ 176.82, 169.92, 143.20, 143.00, 141.76, 132.73, 132.62, 130.33, 130.20, 129.66, 129.29, 129.01, 128.28, 126.12, 36.87, 32.18.

HRMS: [ESI, (+) ve]: Cald. 271.0965 for $C_{16}H_{15}O_4$ observed 271.0965.

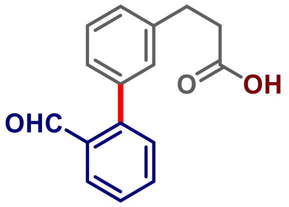

3-(2'-formyl-[1,1'-biphenyl]-3-yl)propanoic acid (6ak): Compound 6ak was synthesized using the general procedure L.

Column material: 100-200 mesh silica

Eluent: petroleum ether/ethyl acetate (60:40, v/v).

Isolated Yield: 61% (over two steps)

^{1}H NMR (400 MHz, $CDCl_3$) δ 9.96 (s, 1H), 8.03 (dd, *J* = 7.8, 1.5 Hz, 1H), 7.64 (td, *J* = 7.5, 1.5 Hz, 1H), 7.49 (tt, *J* = 7.5, 1.1 Hz, 1H), 7.46 – 7.36 (m, 2H), 7.30 (dt, *J* = 7.8, 1.5 Hz, 1H), 7.27 – 7.20 (m, 2H), 3.03 (t, *J* = 7.6 Hz, 2H), 2.73 (t, *J* = 7.6 Hz, 2H).

^{13}C NMR (101 MHz, CDCl$_3$) δ 192.83, 178.30, 146.10, 140.70, 138.13, 133.81, 130.91, 130.37, 128.86, 128.36, 128.28, 127.97, 127.76, 35.61, 30.65.

HRMS [ESI, (+) ve]: calcd. 255.1024 for $C_{16}H_{14}O_3$ observed 255.1024.

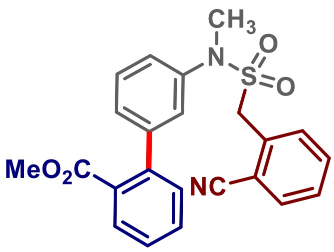

methyl 3'-((1-(2-cyanophenyl)-N-methylmethyl)sulfonamido)-[1,1'-biphenyl]-2-carboxylate (8ac): Compound 8ac was synthesized using the general procedure K.

Column material: 100-200 mesh silica

Eluent: petroleum ether/ethyl acetate (83:17, v/v).

Isolated Yield: 78%

^{1}H NMR (500 MHz, CDCl$_3$) δ 7.85 (dd, *J* = 7.8, 1.4 Hz, 1H), 7.71 – 7.61 (m, 2H), 7.55 (dtd, *J* = 12.9, 7.6, 1.4 Hz, 2H), 7.42 (ddd, *J* = 16.4, 8.8, 7.5 Hz, 2H), 7.34 (dd, *J* = 16.2, 7.8 Hz, 1H), 7.27 (d, *J* = 6.1 Hz, 1H), 7.21 (dt, *J* = 7.7, 1.4 Hz, 1H), 7.11 (s, 1H), 4.54 (s, 2H), 3.67 (s, 3H), 3.40 (s, 3H).

^{13}C NMR (126 MHz, CDCl$_3$) δ 168.71, 142.63, 141.58, 140.79, 133.26, 133.23, 133.19, 132.33, 131.60, 130.96, 130.17, 129.36, 129.10, 127.78, 127.39, 126.34, 125.54, 124.92, 117.47, 114.62, 54.00, 52.28, 38.90.

HRMS: [ESI, (+) ve]: Cald. 438.1488 for $C_{23}H_{24}N_3O_4S$ observed 438.1488.

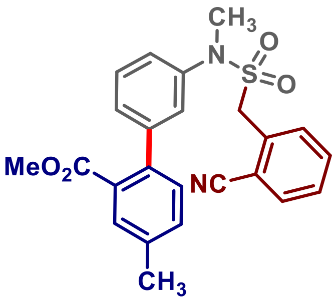

methyl 3'-((1-(2-cyanophenyl)-N-methylmethyl)sulfonamido)-4-methyl-[1,1'-biphenyl]-2-carboxylate (8ag): Compound 8ag was synthesized using the general procedure K.

Column material: 100-200 mesh silica

Eluent: petroleum ether/ethyl acetate (83:17, v/v).

Isolated Yield: 76%

¹H NMR (500 MHz, CDCl₃) δ 7.75 – 7.63 (m, 3H), 7.59 (t, *J* = 7.7 Hz, 1H), 7.43 (t, *J* = 7.8 Hz, 1H), 7.40 – 7.33 (m, 2H), 7.25 (ddd, *J* = 20.5, 12.2, 4.7 Hz, 4H), 7.14 (q, *J* = 1.9 Hz, 1H), 4.56 (d, *J* = 1.7 Hz, 2H), 3.68 (d, *J* = 1.8 Hz, 3H), 3.41 (d, *J* = 1.8 Hz, 3H), 2.45 (s, 3H).

¹³C NMR (126 MHz, CDCl₃) δ 168.93, 142.64, 140.78, 138.73, 137.74, 133.23, 133.17, 132.50, 132.32, 130.89, 130.66, 130.55, 129.35, 129.05, 127.50, 126.38, 124.81, 117.47, 114.64, 53.99, 52.23, 38.93, 21.13.

HRMS: [ESI, (+) ve]: Cald. 449.1535 for $C_{25}H_{25}N_2O_4S$ observed 449.1559.

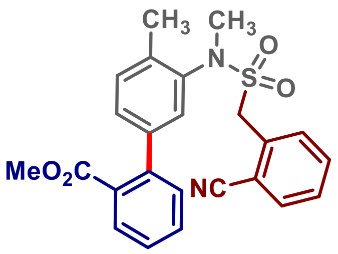

methyl 3'-((1-(2-cyanophenyl)-N-methylmethyl)sulfonamido)-4'-methyl-[1,1'-biphenyl]-4-carboxylate (8bc): Compound 8bc was synthesized using the general procedure K.

Column material: 100-200 mesh silica

Eluent: petroleum ether/ethyl acetate (83:17, v/v).

Isolated Yield: 73%

¹H NMR (400 MHz, CDCl₃) δ 7.83 (dd, *J* = 7.7, 1.4 Hz, 1H), 7.76 – 7.60 (m, 2H), 7.57 – 7.47 (m, 2H), 7.42 (td, *J* = 7.6, 1.3 Hz, 1H), 7.36 – 7.26 (m, 3H), 7.19 (dd, *J* = 7.8, 1.9 Hz, 1H), 6.82 (d, *J* = 1.9 Hz, 1H), 4.64 (s, 2H), 3.68 (s, 3H), 3.33 (s, 3H), 2.40 (s, 3H).

¹³C NMR (101 MHz, CDCl₃) δ 168.88, 141.30, 140.23, 139.54, 137.67, 133.22, 133.16, 132.80, 132.24, 131.54, 131.52, 130.95, 130.75, 130.04, 129.25, 128.69, 128.30, 127.61, 117.59, 114.62, 54.62, 52.29, 39.14, 18.25.

HRMS: [ESI, (+) ve]: Cald. 435.1379 for $C_{24}H_{23}N_2O_4S$ observed 435.1383.

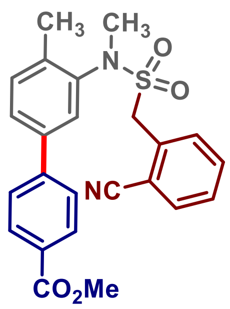

methyl 3'-((1-(2-cyanophenyl)-N-methylmethyl)sulfonamido)-4'-methyl-[1,1'-biphenyl]-4-carboxylate (8be): Compound 8be was synthesized using the general procedure K.

Column material: 100-200 mesh silica

Eluent: petroleum ether/ethyl acetate (83:17, v/v).

Isolated Yield: 75%

^{1}H NMR (400 MHz, $CDCl_3$) δ 8.09 (d, *J* = 8.5 Hz, 2H), 7.81 – 7.65 (m, 2H), 7.61 – 7.43 (m, 5H), 7.35 (d, *J* = 8.0 Hz, 1H), 7.10 (d, *J* = 2.0 Hz, 1H), 4.68 (s, 2H), 3.95 (s, 3H), 3.33 (s, 3H), 2.40 (s, 3H).

^{13}C NMR (126 MHz, $CDCl_3$) δ 167.09, 144.33, 140.64, 139.31, 138.61, 133.33, 133.15, 132.91, 132.42, 132.39, 130.28, 129.49, 127.62, 127.09, 127.06, 117.68, 114.57, 55.18, 52.38, 39.12, 18.27.

HRMS: [ESI, (+) ve]: Cald. 435.1379 for $C_{24}H_{23}N_2O_4S$ observed 435.1383.

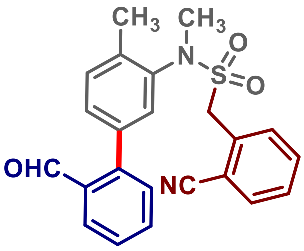

1-(2-cyanophenyl)-N-(2'-formyl-4-methyl-[1,1'-biphenyl]-3-yl)-N-methylmethanesulfonamide (8bk): Compound 8bk was synthesized using the general procedure K.

Column material: 100-200 mesh silica

Eluent: petroleum ether/ethyl acetate (81:19, v/v).

Isolated Yield: 74%

^{1}H NMR (400 MHz, $CDCl_3$) δ 9.91 (s, 1H), 8.01 (dd, *J* = 7.8, 1.4 Hz, 1H), 7.70 – 7.59 (m, 3H), 7.53 (dq, *J* = 14.6, 6.8 Hz, 2H), 7.35 (td, *J* = 7.7, 1.6 Hz, 3H), 7.24 (dd, *J* = 7.8, 1.9 Hz, 1H), 6.88 (d, *J* = 1.8 Hz, 1H), 4.64 (s, 2H), 3.33 (s, 3H), 2.42 (s, 3H).

^{13}C NMR (101 MHz, $CDCl_3$) δ 192.28, 144.43, 140.14, 138.70, 136.92, 133.80, 133.27, 133.11, 132.70, 132.32, 131.89, 130.98, 130.38, 129.69, 129.45, 128.26, 128.09, 117.54, 114.57, 55.02, 39.11, 18.28.

HRMS: [ESI, (+) ve]: Cald. 405.1273 for $C_{23}H_{21}N_2O_3S$ observed 405.1290.

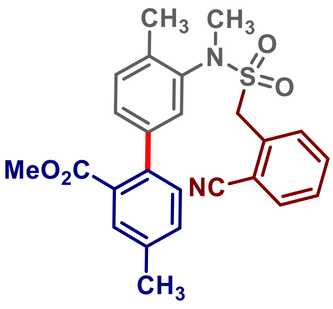

methyl 3'-((1-(2-cyanophenyl)-N-methylmethyl)sulfonamido)-4,4'-dimethyl-[1,1'-biphenyl]-2-carboxylate (8bg): Compound 8bg was synthesized using the general procedure K.

Column material: 100-200 mesh silica

Eluent: petroleum ether/ethyl acetate (82:18, v/v).

Isolated Yield: 80%

^{1}H NMR (500 MHz, CDCl$_3$) δ 7.67 (d, *J* = 1.3 Hz, 1H), 7.66 – 7.63 (m, 2H), 7.53 (td, *J* = 7.7, 1.4 Hz, 1H), 7.36 – 7.32 (m, 2H), 7.28 (d, *J* = 7.9 Hz, 1H), 7.19 – 7.11 (m, 2H), 6.84 (d, *J* = 1.9 Hz, 1H), 4.64 (s, 2H), 3.67 (s, 3H), 3.32 (s, 3H), 2.43 (s, 3H), 2.39 (s, 3H).

^{13}C NMR (126 MHz, CDCl$_3$) δ 169.06, 140.20, 139.48, 138.43, 137.54, 137.42, 133.22, 133.15, 132.78, 132.26, 132.23, 131.46, 130.87, 130.51, 129.24, 128.77, 128.25, 117.58, 114.61, 54.56, 52.24, 39.15, 21.11, 18.23.

HRMS: [ESI, (+) ve]: Cald. 449.1535 for $C_{25}H_{25}N_2O_4S$ observed 449.1559.

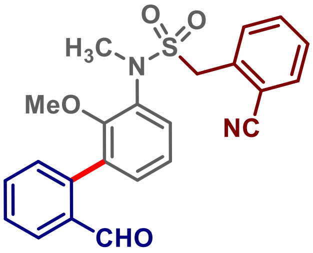

1-(2-cyanophenyl)-N-(2'-formyl-2-methoxy-[1,1'-biphenyl]-3-yl)-N-methylmethanesulfonamide (8ck): Compound 8g was synthesized using the general procedure K.

Column material: 100-200 mesh silica

Eluent: petroleum ether/ethyl acetate (82:18, v/v).

Isolated Yield: 71%

^{1}H NMR (400 MHz, CDCl$_3$) δ 9.98 (d, *J* = 0.8 Hz, 1H), 8.00 (dd, *J* = 7.8, 1.4 Hz, 1H), 7.68 (td, *J* = 7.1, 1.3 Hz, 2H), 7.63 – 7.53 (m, 2H), 7.52 – 7.45 (m, 1H), 7.41 (ddd, *J* = 13.5, 7.6, 1.3 Hz, 2H), 7.31 (dd, *J* = 8.4, 2.3 Hz, 1H), 7.25 (d, *J* = 2.3 Hz, 1H), 7.06 (d, *J* = 8.5 Hz, 1H), 4.61 (s, 2H), 4.00 (s, 3H), 3.37 (s, 3H).

^{13}C NMR (101 MHz, CDCl$_3$) δ 192.49, 156.19, 144.36, 133.81, 133.77, 133.48, 133.15, 133.12, 133.01, 131.98, 131.78, 131.06, 130.86, 129.18, 129.04, 128.01, 127.98, 117.52, 114.74, 112.02, 56.07, 55.45, 38.35.

HRMS: [ESI, (+) ve]: Cald. 421.1222 for $C_{23}H_{21}N_2O_4S$ observed 421.1231

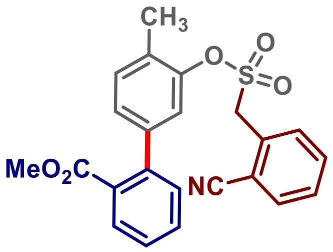

methyl 3'-(((2-cyanobenzyl)sulfonyl)oxy)-4'-methyl-[1,1'-biphenyl]-2-carboxylate (8dc): Compound 8dc was synthesized using the general procedure K.

Column material: 100-200 mesh silica

Eluent: petroleum ether/ethyl acetate (82:18, v/v).

Isolated Yield: 62%

^{1}H NMR (400 MHz, CDCl$_3$) δ 7.83 (dd, J = 7.7, 1.4 Hz, 1H), 7.78 – 7.73 (m, 2H), 7.67 (td, J = 7.7, 1.4 Hz, 1H), 7.57 – 7.48 (m, 2H), 7.46 – 7.37 (m, 1H), 7.36 – 7.30 (m, 1H), 7.24 (s, 1H), 7.14 (dd, J = 7.8, 1.8 Hz, 1H), 7.06 (d, J = 1.8 Hz, 1H), 4.84 (s, 2H), 3.67 (s, 3H), 2.25 (s, 3H).

^{13}C NMR (126 MHz, CDCl$_3$) δ 168.84, 146.98, 140.98, 140.87, 133.60, 133.38, 132.39, 131.61, 131.57, 131.25, 131.00, 130.53, 130.21, 130.02, 127.76, 127.48, 122.38, 117.05, 114.92, 55.32, 52.34, 16.48.

HRMS: [ESI, (+) ve]: Cald. 422.1062 for $C_{23}H_{20}NO_5S$ observed 422.1059

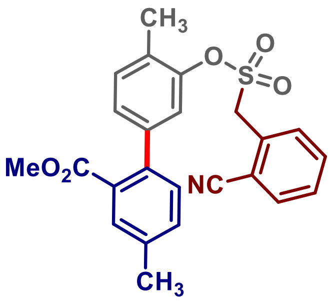

methyl 3'-(((2-cyanobenzyl)sulfonyl)oxy)-4,4'-dimethyl-[1,1'-biphenyl]-2-carboxylate (8dg): Compound 8dg was synthesized using the general procedure K.

Column material: 100-200 mesh silica

Eluent: petroleum ether/ethyl acetate (82:18, v/v).

Isolated Yield: 67%

^{1}H NMR (500 MHz, CDCl$_3$) δ 7.75 (ddd, J = 7.9, 6.6, 1.3 Hz, 2H), 7.67 (td, J = 7.7, 1.4 Hz, 1H), 7.64 (d, J = 2.0 Hz, 1H), 7.52 (td, J = 7.6, 1.3 Hz, 1H), 7.32 (dd, J = 7.9, 1.9 Hz, 1H), 7.22 (dd, J = 14.5, 7.9 Hz, 2H), 7.12 (dd, J = 7.8, 1.8 Hz, 1H), 7.05 (d, J = 1.9 Hz, 1H), 4.83 (s, 2H), 3.66 (s, 3H), 2.41 (s, 3H), 2.25 (s, 3H).

^{13}C NMR (126 MHz, CDCl$_3$) δ 169.05, 146.97, 140.86, 138.12, 137.71, 133.59, 133.37, 132.38, 132.33, 131.51, 131.26, 130.92, 130.69, 130.53, 130.28, 130.01, 127.54, 122.38, 117.05, 114.90, 55.29, 52.29, 21.12, 16.46.

HRMS: [ESI, (+) ve]: Cald. 436.1219 for $C_{24}H_{22}NO_5S$ observed 436.1215.

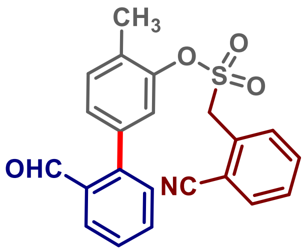

2'-formyl-4-methyl-[1,1'-biphenyl]-3-yl (2-cyanophenyl)methanesulfonate (8dk): Compound 8dk was synthesized using the general procedure K.

Column material: 100-200 mesh silica

Eluent: petroleum ether/ethyl acetate (82:18, v/v).

Isolated Yield: 65%

^{1}H NMR (500 MHz, CDCl$_3$) δ 9.95 (s, 1H), 8.00 (dd, *J* = 7.9, 1.5 Hz, 1H), 7.76 (d, *J* = 8.0 Hz, 1H), 7.69 (td, *J* = 7.8, 1.3 Hz, 1H), 7.65 – 7.57 (m, 2H), 7.57 – 7.47 (m, 2H), 7.39 (d, *J* = 7.7 Hz, 1H), 7.33 (d, *J* = 7.7 Hz, 1H), 7.22 – 7.15 (m, 1H), 7.12 (d, *J* = 1.8 Hz, 1H), 4.86 (s, H), 2.29 (s, 3H).

^{13}C NMR (126 MHz, CDCl$_3$) δ 192.19, 147.25, 144.13, 137.31, 133.86, 133.78, 133.60, 133.47, 132.43, 131.95, 131.75, 131.00, 130.15, 129.26, 129.07, 128.38, 128.07, 123.75, 114.90, 55.45, 16.48.

HRMS: [ESI, (+) ve]: Cald. 392.0957 for $C_{22}H_{18}NO_4S$ observed 392.0955.

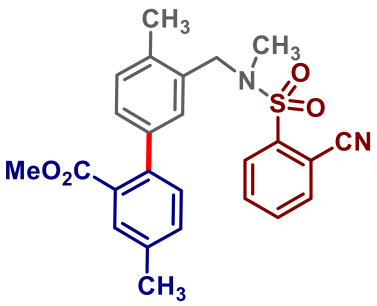

methyl 3'-(((2-cyano-N-methylphenyl)sulfonamido)methyl)-4,4'-dimethyl-[1,1'-biphenyl]-2-carboxylate (8eg): Compound 8eg was synthesized using the general procedure K.

Column material: 100-200 mesh silica

Eluent: petroleum ether/ethyl acetate (87:13, v/v).

Isolated Yield: 58%

^{1}H NMR (400 MHz, CDCl$_3$) δ 8.07 (dd, *J* = 7.9, 1.3 Hz, 1H), 7.88 (dd, *J* = 7.5, 1.4 Hz, 1H), 7.75 (td, *J* = 7.8, 1.5 Hz, 1H), 7.70 – 7.66 (m, 1H), 7.61 (d, *J* = 1.9 Hz, 1H), 7.31 (dd, *J* = 7.9,

1.9 Hz, 1H), 7.22 – 7.13 (m, 3H), 7.09 (d, J = 1.9 Hz, 1H), 4.41 (s, 2H), 3.65 (s, 3H), 2.74 (s, 3H), 2.41 (s, 3H), 2.39 (s, 3H).

^{13}C NMR (101 MHz, CDCl$_3$) δ 169.18, 140.84, 139.29, 137.21, 136.24, 135.85, 133.18, 132.78, 132.40, 132.20, 130.84, 130.77, 130.61, 130.58, 130.50, 129.64, 128.34, 116.73, 110.97, 52.59, 52.13, 34.43, 21.07, 19.05.

HRMS: [ESI, (+) ve]: Cald. 449.1535 for $C_{25}H_{25}N_2O_4S$ observed 449.1559.

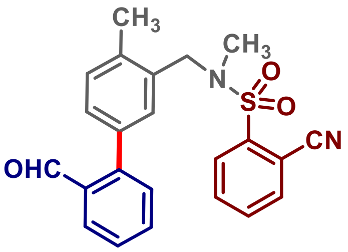

2-cyano-N-((2'-formyl-4-methyl-[1,1'-biphenyl]-3-yl)methyl)-N-methylbenzenesulfonamide(8ek): Compound 8ek was synthesized using the general procedure K.

Column material: 100-200 mesh silica

Eluent: petroleum ether/ethyl acetate (87:13, v/v).

Isolated Yield: 63%

^{1}H NMR (400 MHz, CDCl$_3$) δ 9.94 (d, J = 0.9 Hz, 1H), 8.11 – 8.06 (m, 1H), 8.00 (dd, J = 7.8, 1.5 Hz, 1H), 7.89 (dd, J = 7.6, 1.4 Hz, 1H), 7.77 (td, J = 7.8, 1.6 Hz, 1H), 7.69 (td, J = 7.6, 1.4 Hz, 1H), 7.63 (td, J = 7.5, 1.5 Hz, 1H), 7.49 (tt, J = 7.6, 1.1 Hz, 1H), 7.40 (dd, J = 7.6, 1.3 Hz, 1H), 7.28 (d, J = 7.7 Hz, 1H), 7.25 – 7.18 (m, 2H), 4.49 (s, 2H), 2.77 (s, 3H), 2.44 (s, 3H).

^{13}C NMR (101 MHz, CDCl$_3$) δ 192.42, 145.42, 140.80, 137.51, 135.89, 133.82, 133.32, 133.23, 132.93, 131.16, 130.97, 130.73, 130.68, 130.02, 127.98, 116.68, 110.91, 52.54, 34.65, 19.12.

HRMS: [ESI, (+) ve]: Cald. 405.1273 for $C_{23}H_{21}N_2O_3S$ observed 405.1290.

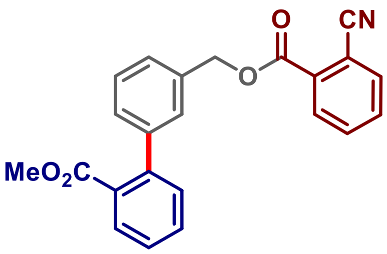

methyl 3'-(((2-cyanobenzoyl)oxy)methyl)-[1,1'-biphenyl]-2-carboxylate (8fc): Compound 8fc was synthesized using the general procedure K.

Column material: 100-200 mesh silica

Eluent: petroleum ether/ethyl acetate (85:15, v/v).

Isolated Yield: 55%

^{1}H NMR (400 MHz, $CDCl_3$) δ 8.25 – 8.11 (m, 1H), 7.89 – 7.76 (m, 2H), 7.70 – 7.62 (m, 2H), 7.58 – 7.46 (m, 2H), 7.46 – 7.35 (m, 4H), 7.30 (dt, J = 7.6, 1.5 Hz, 1H), 5.48 (s, 2H), 3.63 (s, 3H).

^{13}C NMR (101 MHz, $CDCl_3$) δ 169.06, 163.97, 142.09, 141.90, 135.08, 135.02, 132.94, 132.64, 132.44, 131.54, 131.42, 130.94, 130.85, 130.02, 128.79, 128.60, 128.52, 127.61, 127.54, 117.67, 113.20, 67.85, 52.14.

HRMS: [ESI, (+) ve]: Cald. 349.1050 for $C_{23}H_{17}NNaO_4$ observed 349.1051.

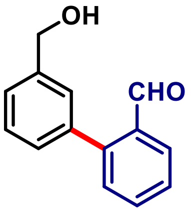

3'-(hydroxymethyl)-[1,1'-biphenyl]-2-carbaldehyde (8fk): Compound 8fk was synthesized using the general procedure K followed by general procedure L.

Column material: 100-200 mesh silica

Eluent: petroleum ether/ethyl acetate (75:25, v/v).

Isolated Yield: 61% (over two steps)

^{1}H NMR (400 MHz, $CDCl_3$) δ 9.94 (s, 1H), 8.01 (dd, J = 7.8, 1.4 Hz, 1H), 7.63 (td, J = 7.5, 1.5 Hz, 1H), 7.52 – 7.46 (m, 1H), 7.46 – 7.41 (m, 3H), 7.38 (d, J = 2.0 Hz, 1H), 7.30 – 7.25 (m, 1H), 4.76 (s, 2H).

^{13}C NMR (101 MHz, $CDCl_3$) δ 192.74, 145.97, 141.43, 138.13, 133.80, 133.77, 130.92, 129.47, 128.74, 128.62, 128.00, 127.78, 126.77, 65.01.

GC-MS: Cald. 212.0837 for $C_{14}H_{12}O_2$ observed 212.08.

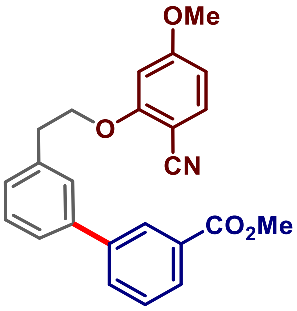

methyl 3'-(2-(2-cyano-5-methoxyphenoxy)ethyl)-[1,1'-biphenyl]-3-carboxylate (8gd): Compound 8gd was synthesized using the general procedure K.

Column material: 100-200 mesh silica

Eluent: petroleum ether/ethyl acetate (87:13, v/v).

Isolated Yield: 30%

^{1}**H NMR (400 MHz, CDCl$_3$)** δ 8.28 (t, *J* = 1.8 Hz, 1H), 8.02 (dt, *J* = 7.9, 1.4 Hz, 1H), 7.83 (ddd, *J* = 7.8, 2.0, 1.2 Hz, 1H), 7.57 (d, *J* = 1.8 Hz, 1H), 7.55 – 7.50 (m, 1H), 7.48 – 7.42 (m, 2H), 7.41 – 7.30 (m, 2H), 6.49 (dd, *J* = 8.6, 2.2 Hz, 1H), 6.42 (d, *J* = 2.3 Hz, 1H), 4.27 (t, *J* = 6.9 Hz, 2H), 3.95 (s, 3H), 3.81 (s, 3H), 3.24 (t, *J* = 6.8 Hz, 2H).

^{13}**C NMR (101 MHz, CDCl$_3$)** δ 167.28, 164.66, 162.25, 141.43, 140.64, 138.55, 135.08, 131.88, 130.83, 130.06, 129.38, 129.08, 128.78, 128.43, 128.16, 125.83, 117.08, 106.19, 99.31, 94.49, 69.87, 55.85, 52.39, 35.83, 31.12.

HRMS: [ESI, (+) ve]: Cald. 410.1363 for $C_{24}H_{21}NNaO_4$ observed 410.1365.

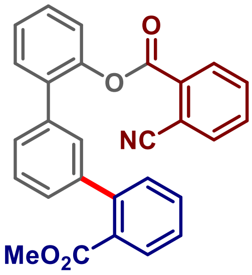

methyl 2''-((2-cyanobenzoyl)oxy)-[1,1':3',1''-terphenyl]-2-carboxylate (8hc): Compound 8hc was synthesized using the general procedure K.

Column material: 100-200 mesh silica

Eluent: petroleum ether/ethyl acetate (86:14, v/v).

Isolated Yield: 73%

^{1}**H NMR (400 MHz, CDCl$_3$)** δ 8.04 (dd, *J* = 7.7, 1.4 Hz, 1H), 7.80 (ddd, *J* = 8.9, 7.6, 1.5 Hz, 2H), 7.65 (td, *J* = 7.6, 1.5 Hz, 1H), 7.58 (dd, *J* = 7.7, 1.4 Hz, 1H), 7.52 (dd, *J* = 7.4, 1.9 Hz, 1H), 7.45 (tdd, *J* = 7.7, 2.2, 1.3 Hz, 4H), 7.42 – 7.32 (m, 4H), 7.24 – 7.18 (m, 2H), 3.52 (s, 3H).

^{13}**C NMR (101 MHz, CDCl$_3$)** δ 169.04, 162.50, 147.60, 142.22, 141.60, 137.19, 135.06, 134.78, 133.21, 132.63, 131.70, 131.65, 131.41, 131.19, 130.83, 130.79, 130.01, 129.07, 128.97, 128.09, 127.96, 127.77, 127.44, 127.07, 122.97, 117.23, 113.49, 52.05.

HRMS [ESI, (+) ve]: calcd. 456.1206 for $C_{28}H_{19}NNaO_4$ observed 456.1212.

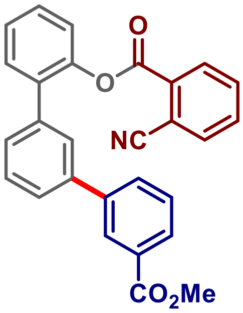

methyl 2''-((2-cyanobenzoyl)oxy)-[1,1':3',1''-terphenyl]-3-carboxylate (8hd): Compound 8hd was synthesized using the general procedure K.

Column material: 100-200 mesh silica

Eluent: petroleum ether/ethyl acetate (86:14, v/v).

Isolated Yield: 68%

^{1}H NMR (500 MHz, CDCl$_3$) δ 8.20 (t, *J* = 1.8 Hz, 1H), 8.04 (dd, *J* = 7.8, 1.4 Hz, 1H), 8.00 (d, *J* = 7.7 Hz, 1H), 7.77 (dd, *J* = 7.7, 1.5 Hz, 1H), 7.72 (t, *J* = 1.7 Hz, 1H), 7.71 – 7.67 (m, 1H), 7.62 (td, *J* = 7.2, 2.3 Hz, 1H), 7.58 – 7.51 (m, 3H), 7.51 – 7.41 (m, 5H), 7.37 (dd, *J* = 7.9, 1.4 Hz, 1H), 3.94 (s, 3H).

^{13}C NMR (126 MHz, CDCl$_3$) δ 167.19, 162.62, 147.68, 141.15, 140.26, 138.10, 135.14, 134.84, 134.52, 133.29, 132.66, 131.67, 131.64, 131.58, 131.22, 130.87, 129.15, 129.05, 128.67, 128.61, 128.30, 128.02, 127.14, 126.50, 123.04, 117.27, 113.50, 52.41.

HRMS [ESI, (+) ve]: calcd. 456.1206 for $C_{28}H_{19}NNaO_4$ observed 456.1210.

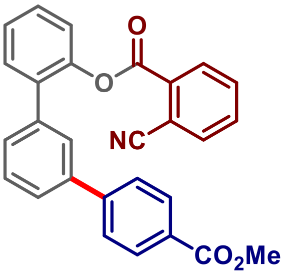

methyl 2''-((2-cyanobenzoyl)oxy)-[1,1':3',1''-terphenyl]-4-carboxylate (8he): Compound 8he was synthesized using the general procedure K.

Column material: 100-200 mesh silica

Eluent: petroleum ether/ethyl acetate (86:14, v/v).

Isolated Yield: 65%

^{1}H NMR (500 MHz, CDCl$_3$) δ 8.04 (td, *J* = 6.3, 1.5 Hz, 2H), 7.78 (dd, *J* = 7.6, 1.3 Hz, 1H), 7.73 (t, *J* = 1.8 Hz, 1H), 7.67 – 7.59 (m, 2H), 7.57 – 7.50 (m, 5H), 7.48 – 7.35 (m, 5H), 3.94 (s, 3H).

^{13}C NMR (126 MHz, CDCl$_3$) δ 167.15, 162.60, 147.63, 145.30, 140.13, 138.10, 135.17, 134.69, 133.38, 132.63, 131.58, 131.19, 130.23, 129.72, 129.20, 129.15, 128.97, 128.08, 127.18, 127.14, 126.63, 123.05, 117.24, 113.50, 52.36, 29.88.

HRMS [ESI, (+) ve]: calcd. 456.1206 for $C_{28}H_{19}NNaO_4$ observed 456.1210.

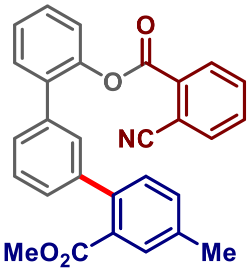

methyl 2''-((2-cyanobenzoyl)oxy)-4-methyl-[1,1':3',1''-terphenyl]-2-carboxylate (8hg): Compound 8hg was synthesized using the general procedure K.

Column material: 100-200 mesh silica

Eluent: petroleum ether/ethyl acetate (85:15, v/v).

Isolated Yield: 72%

^{1}H NMR (400 MHz, CDCl$_3$) δ 8.04 (dd, *J* = 7.9, 1.4 Hz, 1H), 7.83 – 7.74 (m, 1H), 7.69 – 7.55 (m, 3H), 7.51 (dd, *J* = 7.5, 1.9 Hz, 1H), 7.47 – 7.30 (m, 7H), 7.20 – 7.14 (m, 1H), 7.10 (d, *J* = 7.9 Hz, 1H), 3.51 (s, 3H), 2.41 (s, 3H).

^{13}C NMR (126 MHz, CDCl$_3$) δ 169.24, 162.51, 147.61, 141.59, 139.38, 137.32, 137.17, 135.05, 134.88, 133.18, 132.63, 132.14, 131.75, 131.65, 131.21, 130.72, 130.64, 130.50, 129.10, 128.92, 128.03, 127.85, 127.77, 127.05, 122.96, 117.24, 113.51, 51.99, 21.07.

HRMS [ESI, (+) ve]: calcd. 470.1363 for $C_{29}H_{21}NNaO_4$ observed 470.1365.

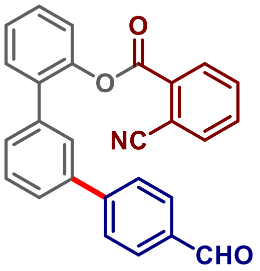

4''-formyl-[1,1':3',1''-terphenyl]-2-yl 2-cyanobenzoate (8hn): Compound 8hn was synthesized using the general procedure K.

Column material: 100-200 mesh silica

Eluent: petroleum ether/ethyl acetate (85:15, v/v).

Isolated Yield: 55%

^{1}H NMR (500 MHz, CDCl$_3$) δ 10.04 (s, 1H), 8.05 (dd, *J* = 7.8, 1.4 Hz, 1H), 7.90 (d, *J* = 8.4 Hz, 2H), 7.79 (dd, *J* = 7.6, 1.4 Hz, 1H), 7.74 (t, *J* = 1.9 Hz, 1H), 7.65 (dd, *J* = 8.1, 1.7 Hz, 2H), 7.61 – 7.49 (m, 5H), 7.52 – 7.45 (m, 2H), 7.42 (d, *J* = 1.3 Hz, 1H), 7.37 (dd, *J* = 8.0, 1.3 Hz, 1H).

^{13}C NMR (126 MHz, CDCl$_3$) δ 192.14, 162.61, 147.65, 146.88, 139.89, 138.25, 135.42, 135.19, 134.63, 133.40, 132.64, 131.60, 131.19, 130.42, 129.82, 129.28, 128.19, 127.81, 127.22, 126.72, 123.07, 117.23, 113.51.

HRMS [ESI, (+) ve]: calcd. 426.1101 for $C_{27}H_{17}NNaO_3$ observed 426.1105.

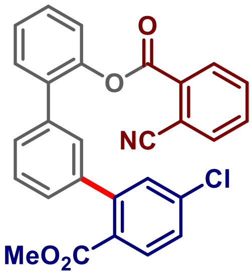

methyl 5-chloro-2''-((2-cyanobenzoyl)oxy)-[1,1':3',1''-terphenyl]-2-carboxylate (8hp): Compound 8hp was synthesized using the general procedure K.

Column material: 100-200 mesh silica

Eluent: petroleum ether/ethyl acetate (85:15, v/v).

Isolated Yield: 75%

^{1}H NMR (400 MHz, CDCl$_3$) δ 8.06 (dd, J = 7.8, 1.5 Hz, 1H), 7.84 – 7.75 (m, 2H), 7.64 (dtd, J = 23.8, 7.6, 1.4 Hz, 2H), 7.55 – 7.41 (m, 5H), 7.40 – 7.31 (m, 3H), 7.17 (dt, J = 7.8, 1.4 Hz, 1H), 7.13 (d, J = 2.1 Hz, 1H), 3.53 (s, 3H).

^{13}C NMR (101 MHz, CDCl$_3$) δ 167.90, 162.48, 147.60, 144.23, 140.35, 137.47, 137.35, 135.18, 134.63, 133.36, 132.71, 131.65, 131.60, 131.19, 130.84, 129.11, 129.05, 128.94, 128.86, 128.49, 128.31, 127.63, 127.61, 127.14, 123.00, 117.24, 113.50, 52.21.

HRMS [ESI, (+) ve]: calcd. 490.0817 for $C_{28}H_{18}ClNNaO_4$ observed 490.0815.

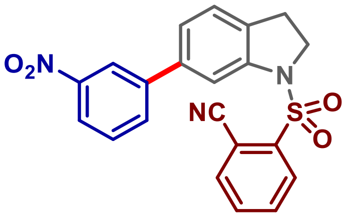

2-((6-(3-nitrophenyl)indolin-1-yl)sulfonyl)benzonitrile (10aa): Compound 10aa was synthesized using the general procedure K.

Column material: 100-200 mesh silica

Eluent: petroleum ether/ethyl acetate (88:12, v/v).

Isolated Yield: 60%

^{1}H NMR (400 MHz, CDCl$_3$) δ 8.32 (dt, J = 16.1, 2.0 Hz, 1H), 8.26 – 8.10 (m, 2H), 7.97 – 7.66 (m, 4H), 7.64 – 7.34 (m, 3H), 7.25 (d, J = 5.2 Hz, 1H), 4.36 (td, J = 8.4, 6.2 Hz, 2H), 3.17 (dt, J = 17.4, 8.4 Hz, 2H).

^{13}C NMR (101 MHz, CDCl$_3$) δ 142.27, 141.12, 136.04, 134.77, 133.41, 133.28, 132.81, 132.37, 130.55, 129.94, 127.07, 126.11, 124.37, 123.27, 122.41, 122.04, 114.53, 113.09, 110.91, 51.44, 27.98.

HRMS: [ESI, (+) ve]: Cald. 406.0862 for $C_{21}H_{16}N_3O_4S$ observed 406.0871.

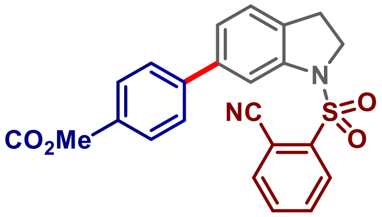

methyl 4-(1-((2-cyanophenyl)sulfonyl)indolin-6-yl)benzoate (10ae): Compound 10ae was synthesized using the general procedure K.

Column material: 100-200 mesh silica

Eluent: petroleum ether/ethyl acetate (88:12, v/v).

Isolated Yield: 71%

^{1}H NMR (500 MHz, CDCl$_3$) δ 8.17 (td, *J* = 8.1, 1.4 Hz, 1H), 8.11 – 8.01 (m, 2H), 7.85 (ddd, *J* = 7.6, 3.6, 1.5 Hz, 1H), 7.73 (dtd, *J* = 16.6, 7.6, 1.4 Hz, 1H), 7.64 – 7.57 (m, 1H), 7.57 – 7.51 (m, 1H), 7.44 – 7.34 (m, 2H), 7.24 – 7.20 (m, 1H), 4.34 (td, *J* = 8.4, 4.1 Hz, 2H), 3.93 (d, *J* = 7.2 Hz, 3H), 3.14 (dt, *J* = 21.1, 8.3 Hz, 2H).

^{13}C NMR (126 MHz, CDCl$_3$) δ 167.11, 145.19, 144.92, 141.08, 141.03, 140.17, 136.10, 136.03, 136.00, 133.33, 133.25, 133.20, 132.80, 132.00, 130.48, 130.39, 130.28, 129.24, 128.87, 127.22, 127.10, 126.78, 125.87, 124.42, 123.43, 116.35, 114.36, 113.14, 110.87, 52.30, 51.41, 27.97.

HRMS: [ESI, (+) ve]: Cald. 419.1066 for $C_{23}H_{19}N_2O_4S$ observed 419.1073.

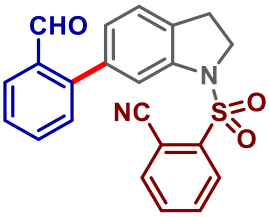

2-((6-(2-formylphenyl)indolin-1-yl)sulfonyl)benzonitrile (10ak): Compound 10ak was synthesized using the general procedure K.

Column material: 100-200 mesh silica

Eluent: petroleum ether/ethyl acetate (88:12, v/v).

Isolated Yield: 65%

^{1}H NMR (500 MHz, CDCl$_3$) δ 9.91 (s, 1H), 8.22 (d, *J* = 7.9 Hz, 1H), 8.04 – 7.93 (m, 1H), 7.88 (dd, *J* = 7.6, 1.4 Hz, 1H), 7.78 (td, *J* = 7.7, 1.4 Hz, 1H), 7.72 (td, *J* = 7.6, 1.3 Hz, 1H), 7.61 (td, *J* = 7.6, 1.5 Hz, 1H), 7.47 (t, *J* = 7.6 Hz, 1H), 7.38 (dd, *J* = 14.1, 8.0 Hz, 2H), 7.17 (s, 1H), 7.13 (d, *J* = 8.2 Hz, 1H), 4.39 (t, *J* = 8.4 Hz, 2H), 3.18 (t, *J* = 8.4 Hz, 2H).

^{13}C NMR (126 MHz, CDCl$_3$) δ 192.52, 145.33, 141.26, 141.12, 136.06, 133.88, 133.82, 133.73, 133.43, 133.30, 132.41, 130.86, 130.59, 129.98, 127.96, 127.25, 116.34, 113.80, 110.95, 51.47, 27.96.

HRMS: [ESI, (+) ve]: Cald. 389.0960 for $C_{22}H_{17}N_2O_3S$ observed 389.0963.

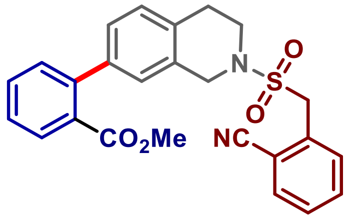

methyl 2-(2-((2-cyanobenzyl)sulfonyl)-1,2,3,4-tetrahydroisoquinolin-7-yl)benzoate (10bc): Compound 10bc was synthesized using the general procedure K.

Column material: 100-200 mesh silica

Eluent: petroleum ether/ethyl acetate (88:12, v/v).

Isolated Yield: 68%

^{1}H NMR (500 MHz, $CDCl_3$) δ 7.82 (dd, J = 7.8, 1.4 Hz, 1H), 7.76 – 7.59 (m, 4H), 7.50 (dtd, J = 16.6, 7.6, 1.4 Hz, 2H), 7.40 (td, J = 7.6, 1.3 Hz, 1H), 7.33 (td, J = 7.8, 1.3 Hz, 1H), 7.16 – 7.08 (m, 1H), 6.96 (s, 1H), 4.50 (s, 2H), 4.45 (s, 2H), 3.68 (s, 3H), 3.56 (t, J = 6.1 Hz, 2H), 2.93 (t, J = 5.9 Hz, 2H).

^{13}C NMR (126 MHz, $CDCl_3$) δ 169.05, 142.07, 139.79, 133.28, 133.17, 132.93, 132.40, 131.90, 131.57, 130.93, 130.72, 130.07, 129.44, 128.89, 127.46, 127.37, 126.87, 126.05, 117.53, 114.37, 55.56, 52.27, 47.39, 43.95, 28.97.

HRMS: [ESI, (+) ve]: Cald. 447.1379 for $C_{25}H_{23}N_2O_4S$ observed 447.1380.

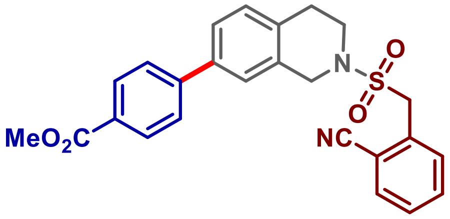

methyl 4-(2-((2-cyanobenzyl)sulfonyl)-1,2,3,4-tetrahydroisoquinolin-7-yl)benzoate (10be): Compound 10be was synthesized using the general procedure K.

Column material: 100-200 mesh silica

Eluent: petroleum ether/ethyl acetate (88:12, v/v).

Isolated Yield: 67%

^{1}H NMR (500 MHz, $CDCl_3$) δ 8.09 (d, J = 8.0 Hz, 2H), 7.73 (d, J = 7.8 Hz, 1H), 7.66 (d, J = 7.9 Hz, 2H), 7.62 – 7.55 (m, 2H), 7.51 – 7.39 (m, 3H), 7.24 – 7.21 (m, 1H), 4.51 (s, 2H), 4.48 (s, 2H), 3.94 (s, 3H), 3.56 (t, J = 5.7 Hz, 2H), 2.94 (q, J = 5.6 Hz, 2H).

^{13}C NMR (126 MHz, $CDCl_3$) δ 167.13, 145.06, 138.48, 133.57, 133.29, 133.13, 132.90, 132.78, 132.39, 130.32, 129.91, 129.44, 128.04, 127.01, 126.04, 125.07, 117.53, 114.34, 55.65, 52.34, 47.45, 43.91, 28.93.

HRMS: [ESI, (+) ve]: Cald. 447.1379 for $C_{25}H_{23}N_2O_4S$ observed 447.1380.

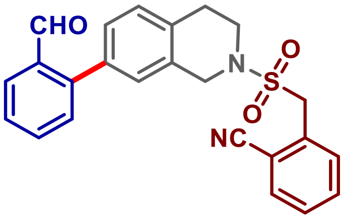

2-(((7-(2-formylphenyl)-3,4-dihydroisoquinolin-2(1H)-yl)sulfonyl)methyl)benzonitrile (10bk): Compound 10bk was synthesized using the general procedure K.

Column material: 100-200 mesh silica

Eluent: petroleum ether/ethyl acetate (88:12, v/v).

Isolated Yield: 61%

^{1}H NMR (400 MHz, CDCl$_3$) δ 9.95 (s, 1H), 8.01 (dd, J = 7.9, 1.6 Hz, 1H), 7.77 – 7.45 (m, 7H), 7.39 (d, J = 7.4 Hz, 1H), 7.19 (d, J = 1.9 Hz, 1H), 7.01 (s, 1H), 4.52 (s, 2H), 4.46 (s, 2H), 3.56 (dt, J = 9.7, 5.9 Hz, 2H), 3.02 – 2.78 (m, 2H).

^{13}C NMR (101 MHz, CDCl$_3$) δ 192.47, 145.40, 136.29, 133.83, 133.57, 133.33, 133.14, 132.90, 132.44, 130.89, 129.51, 129.37, 128.98, 128.07, 127.91, 127.76, 126.46, 117.52, 114.32, 55.77, 45.30, 43.87, 28.99.

HRMS: [ESI, (+) ve]: Cald. 417.1273 for $C_{24}H_{21}N_2O_3S$ observed 417.1280.

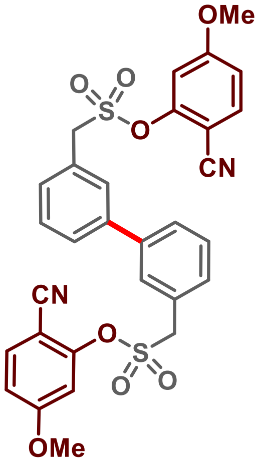

bis(2-cyano-5-methoxyphenyl) [1,1'-biphenyl]-3,3'-diyldimethanesulfonate (11a): Compound 11a was synthesized using the general procedure K in absence of the aryl iodide.

Column material: 100-200 mesh silica

Eluent: petroleum ether/ethyl acetate (60:40, v/v).

Isolated Yield: 68%

^{1}H NMR (500 MHz, CDCl$_3$) δ 7.73 (d, *J* = 1.9 Hz, 2H), 7.66 (dt, *J* = 7.0, 1.9 Hz, 2H), 7.57 (d, *J* = 8.7 Hz, 2H), 7.55 – 7.48 (m, 4H), 6.90 (d, *J* = 2.4 Hz, 2H), 6.88 (dt, *J* = 8.6, 2.7 Hz, 2H), 4.79 (s, 4H), 3.83 (s, 6H).

^{13}C NMR (101 MHz, CDCl$_3$) δ 164.29, 151.95, 141.24, 134.60, 130.54, 130.05, 129.87, 128.60, 127.25, 115.72, 114.07, 109.25, 58.67, 56.30.

HRMS: [ESI, (+) ve]: Cald. 605.1052 for $C_{30}H_{25}N_2O_8S_2$ observed 605.1042.

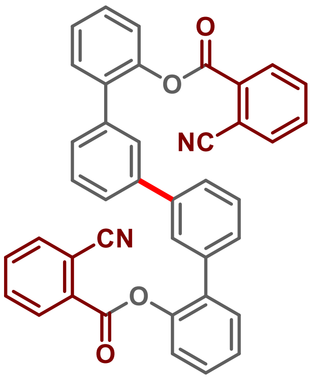

[1,1':3',1'':3'',1'''-quaterphenyl]-2,2'''-diyl bis(2-cyanobenzoate) (11b): Compound 11b was synthesized using the general procedure K in absence of the aryl iodide.

Column material: 100-200 mesh silica

Eluent: petroleum ether/ethyl acetate (70:30, v/v).

Isolated Yield: 71%

^{1}H NMR (400 MHz, CDCl$_3$) δ 8.00 (dd, *J* = 7.9, 1.3 Hz, 2H), 7.65 (dd, *J* = 7.8, 1.3 Hz, 2H), 7.57 – 7.32 (m, 20H).

^{13}C NMR (101 MHz, CDCl$_3$) δ 162.53, 147.64, 140.84, 137.82, 135.05, 134.88, 133.31, 132.66, 131.53, 131.51, 131.21, 129.10, 129.04, 128.27, 127.71, 127.13, 126.51, 123.07, 117.24, 113.37.

HRMS: [ESI, (+) ve]: Cald. 597.1814 for $C_{40}H_{25}N_2O_4$ observed 597.1803.

6. Kinetic Studies

k_H/k_D Experiment

To understand the reversibility of the protocol and to learn more about the rate-determining step of the reaction, we tried to perform the kinetic isotope effect experiment. Substrate (1a) treated towards the kinetic isotope experiment to predict the importance of C–H activation step in the overall methodology. The results are as follows:

Set (I) Yield with Substrate (**1a**)

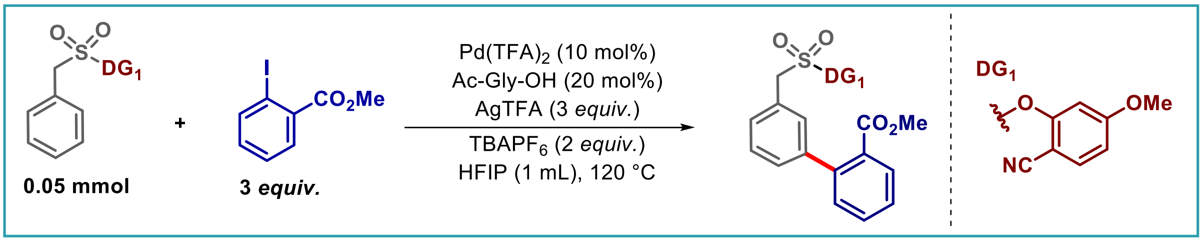

S. No.	Time (min)	Yield (%)	Concentration (mM)
1	15	11	5.50
2	30	20	9.75
3	60	26	13.00
4	120	35	17.50
5	180	38	18.75
6	240	40	20.00
7	300	43	21.50
8	360	47.5	23.75

Set (II) Yield with deuterated Substrate (**d_7-1a**)

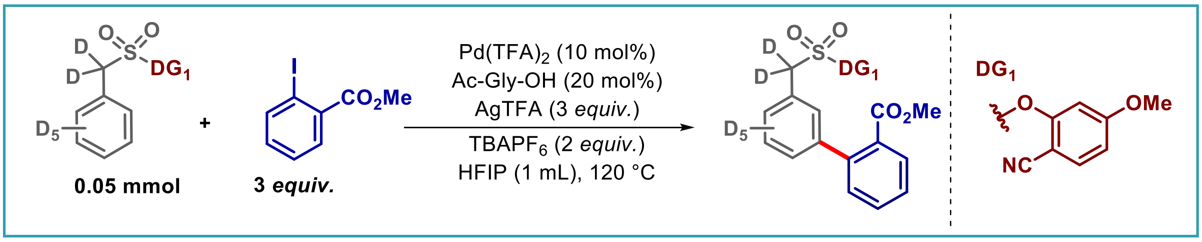

S. No.	Time (min)	Yield (%)	Concentration (mM)
1	15	5	2.45
2	30	9	4.53
3	60	12	5.98
4	120	16	8.01
5	180	17	8.43
6	240	18.5	9.15
7	300	20	9.90
8	360	21.5	10.70

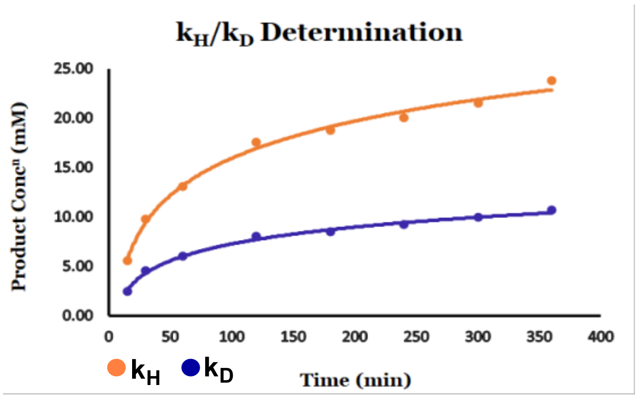

k_H/k_D = Initial rate of 1/ Initial rate of 2

$k_H/k_D = 0.0974/0.0447$

$k_H/k_D = 2.17$

Initial rate is determined by calculating the slope of tangent at time = Zero.

Order determination Studies:

To probe more about the mechanistic insights and to get a full proof idea about the components involved in the rate-determining step of the reaction, we carried out the order determination studies using substrate (**1a**) and 2-iodomethyl benzoate (**2a**) with respect to substrate and aryl iodide. The results are as follows:

Order with respect to substrate (**1a**)

Run 1

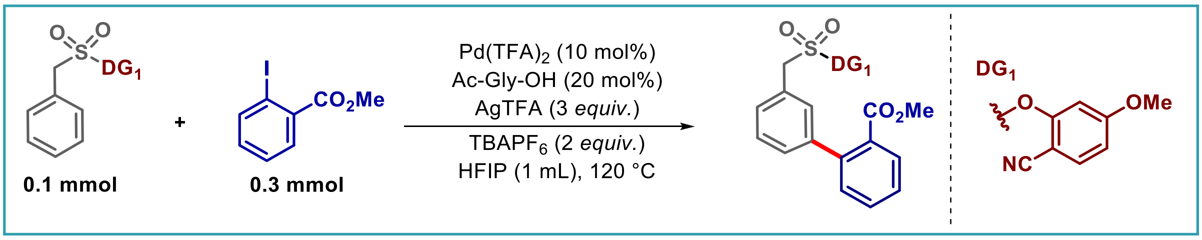

S. No.	Time (min)	Yield (%)	Concentration (mM)
1	15	12.00	12
2	30	19.50	19.5
3	60	27.00	27
4	120	34.50	34.50
5	180	38.00	38.00
6	240	41.00	41.00
7	300	42.80	42.80
8	360	48.00	48.00

Run 2

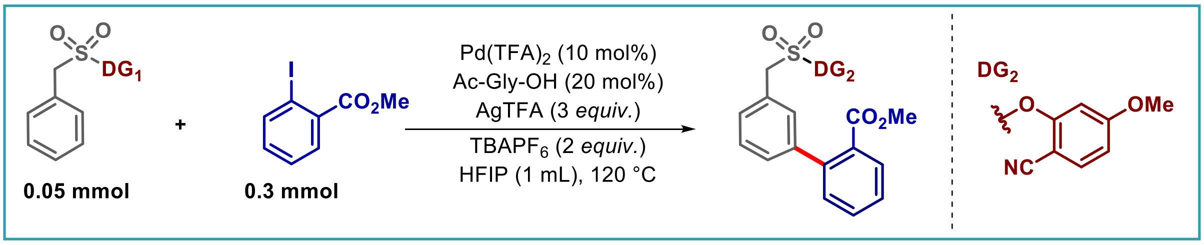

S. No.	Time (min)	Yield (%)	Concentration (mM)
1	15	10.00	5.00
2	30	18.80	9.90
3	60	23.50	11.75

4	120	31.50	15.75
5	180	33.00	16.50
6	240	36.50	18.25
7	300	41.50	20.75
8	360	44.00	22.00

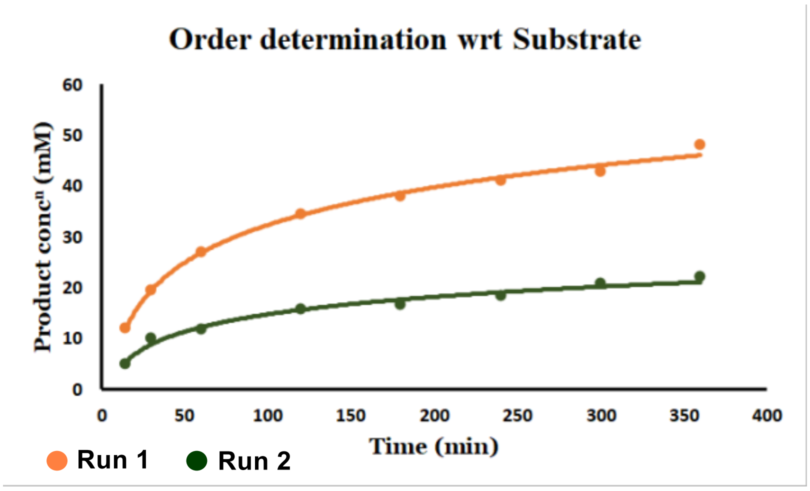

Initial rate of 1/Initial rate of 2 = $(2)^n$

$(2)^n = 0.1913/0.0809$

$(2)^n = 2.364$

$n\text{Log}(2) = \text{Log}(2.134)$

$0.3010n = 0.373$

$n = 0.373/0.3010 = 1.23$

Order with respect to meta scafold is 1.23

Order with respect to aryl iodide

Run 3

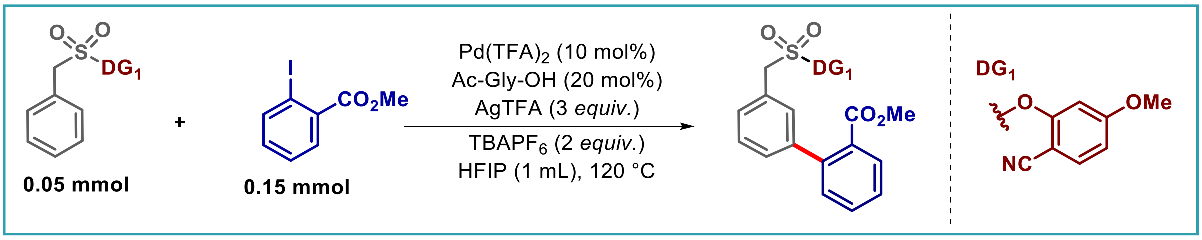

S. No.	Time (min)	Yield (%)	Concentration (mM)
1	15	11.00	5.50
2	30	19.50	9.75
3	60	26.00	13.00
4	120	35.00	17.50
5	180	37.50	18.75
6	240	40.00	20.00

7	300	43.00	21.50

Run 4

S. No.	Time (min)	Yield (%)	Concentration (mM)
1	15	6.00	3
2	30	9.00	4.5
3	60	12.00	6
4	120	16.60	8.3
5	180	23.00	11.5
6	240	27.00	13.5
7	300	32.00	16

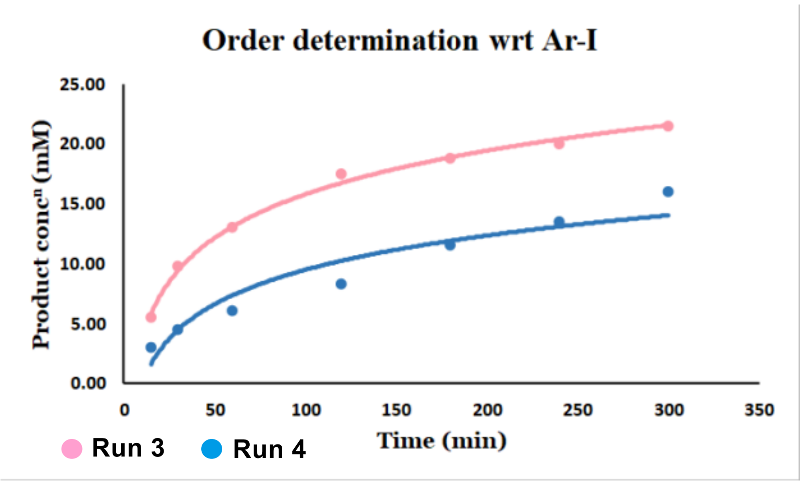

Initial rate of 3/ Initial rate of 4 = $(2)^n$

0.1213/0.0541 = $(2)^n$

$(2)^n = 2.24$

nLog(2) = Log (2.24)

n= 0.350/0.3010 = 1.16 ~1 = Order of aryl iodide

Kinetics with respect to amount of silver:

Run 5

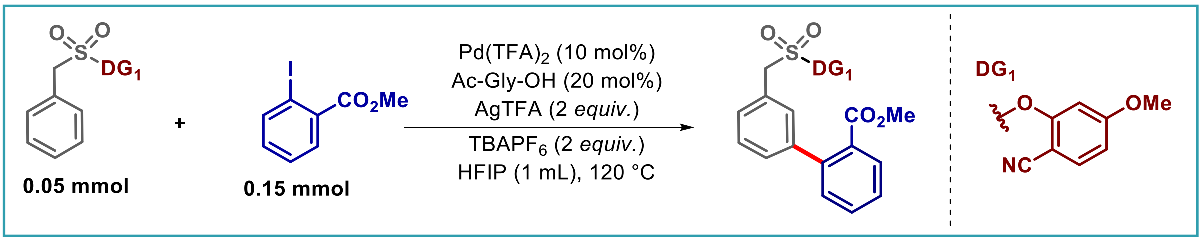

S. No.	Time (min)	Yield (%)	Concentration (mM)
1	15	6.00	3.00

2	30	10.00	5.00
3	60	18.00	9.00
4	120	24.00	12.00
5	180	26.00	13.00
6	240	30.00	15.00
7	300	33.00	16.50
8	360	36.50	18.25

Run 6

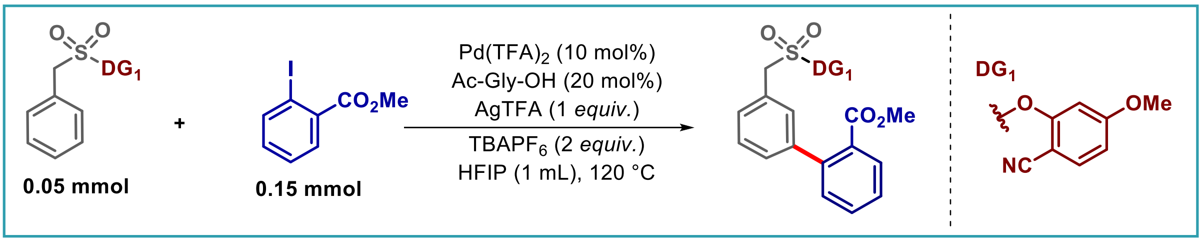

S. No.	Time (min)	Yield (%)	Concentration (mM)
1	15	2.50	1.25
2	30	4.00	2.00
3	60	7.00	3.50
4	120	11.00	5.50
5	180	12.00	6.00
6	240	14.50	7.25
7	300	16.00	8.00
8	360	18.00	9.00

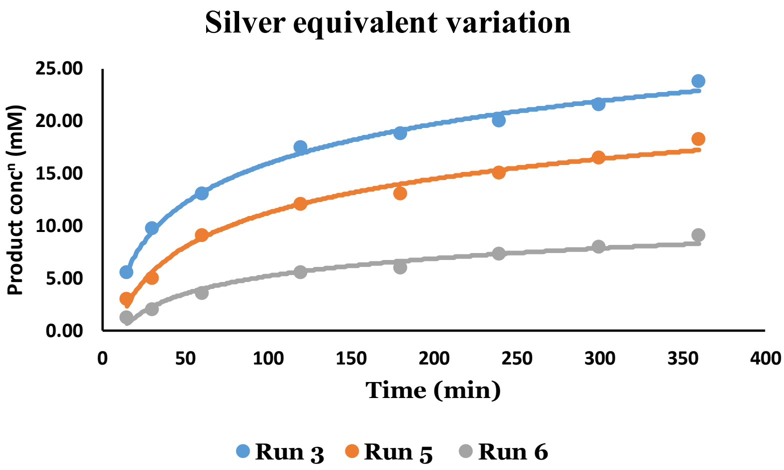

Amount of silver is having a profound effect on kinetics of reaction. With Silver three equivalent in amount, maximum product formation was observed. While, a significant decrease was observed when 1 equivalent of silver was used.

P_H/P_D Experiment:

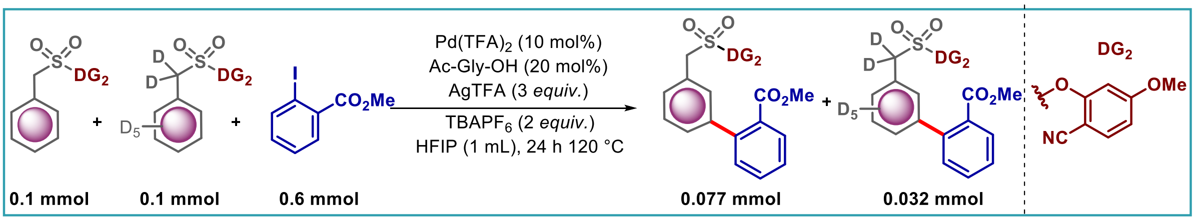

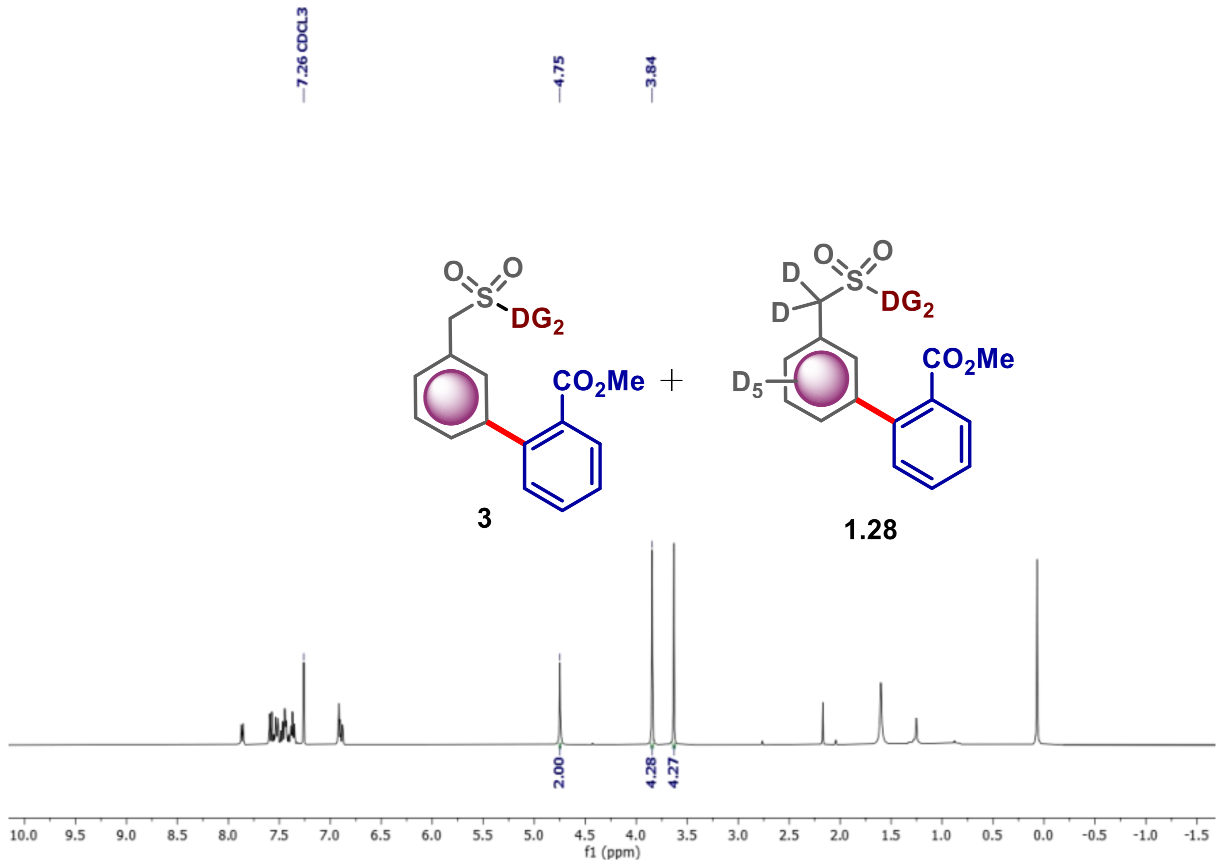

$$\frac{P_H}{P_D} = 2.3$$

7. Reversibility studies

a)

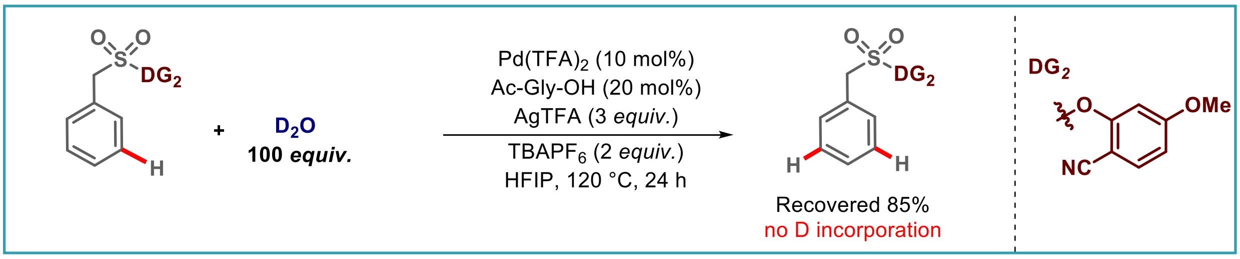

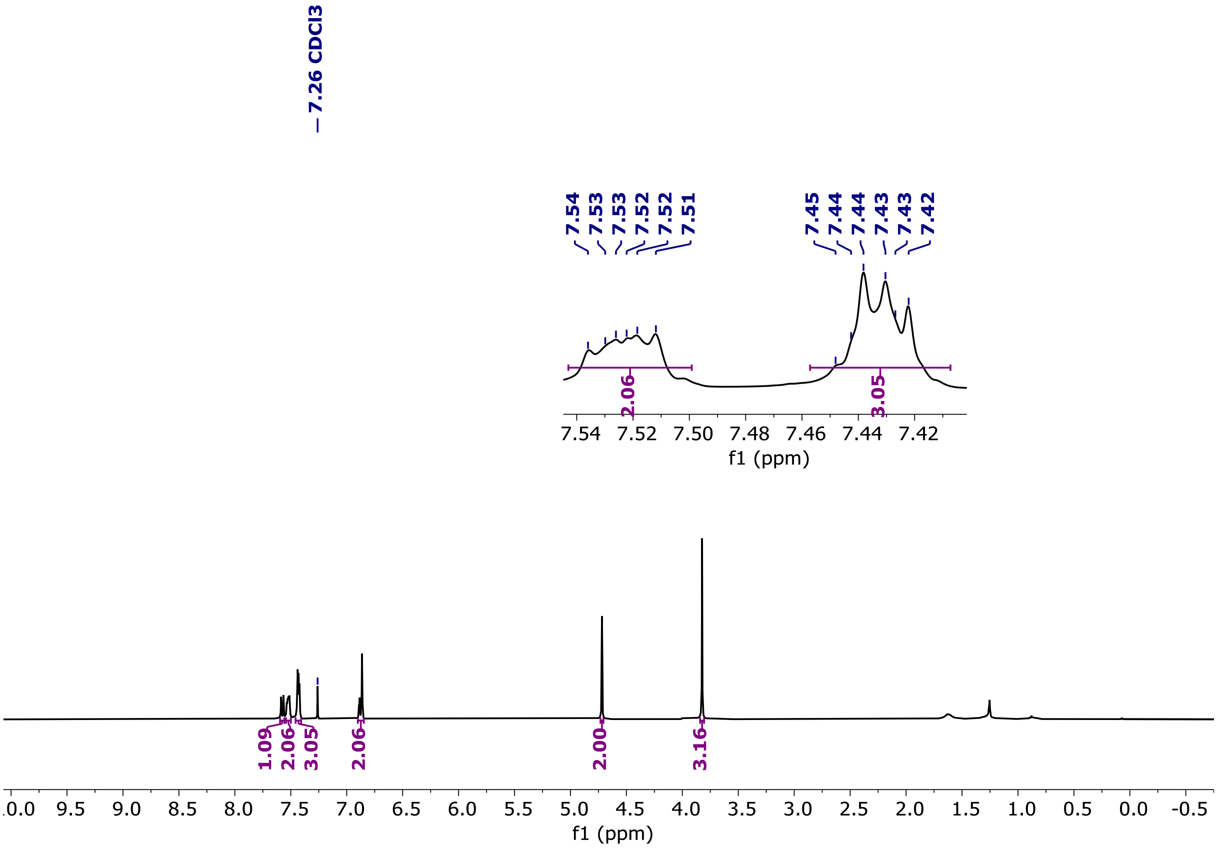

b)

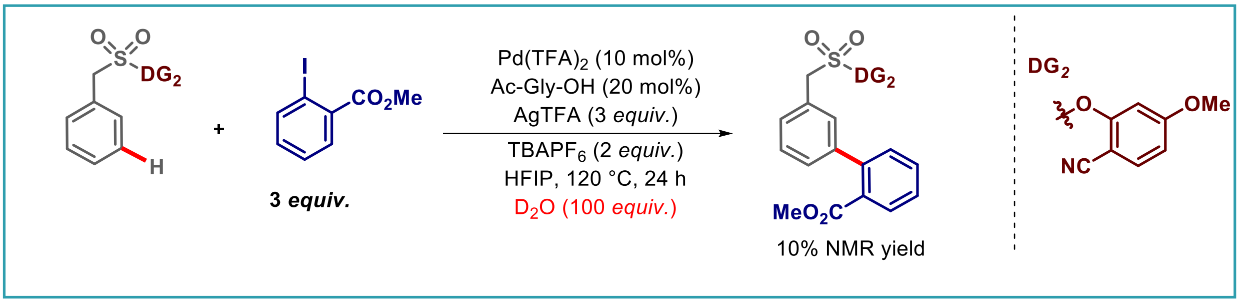

DG2
3 equiv.
Pd(TFA)2 (10 mol%)
Ac-Gly-OH (20 mol%)
AgTFA (3 equiv.)
TBAPF6 (2 equiv.)
HFIP, 120 °C, 24 h
D2O (100 equiv.)
10% NMR yield
DG2
OMe
NC

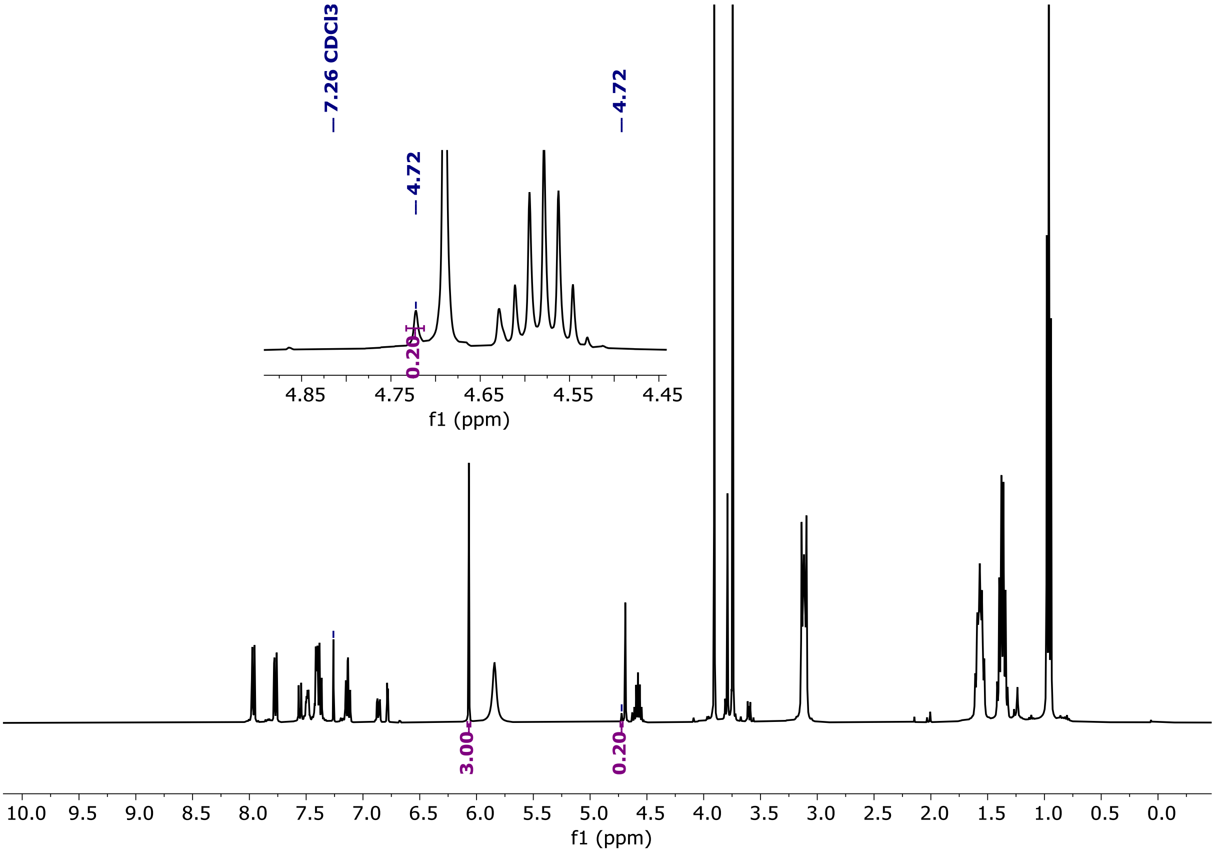

7.26 CDCl3
4.72
4.72
0.20
4.85
4.75
4.65
4.55
4.45
f1 (ppm)
3.00
0.20
10.0 9.5 9.0 8.5 8.0 7.5 7.0 6.5 6.0 5.5 5.0 4.5 4.0 3.5 3.0 2.5 2.0 1.5 1.0 0.5 0.0
f1 (ppm)

c)

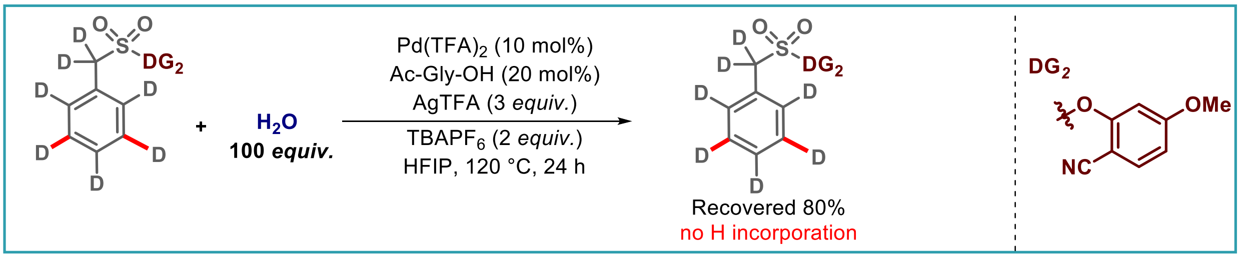

H2O
100 equiv.
Pd(TFA)2 (10 mol%)
Ac-Gly-OH (20 mol%)
AgTFA (3 equiv.)
TBAPF6 (2 equiv.)
HFIP, 120 °C, 24 h
Recovered 80%
no H incorporation
DG2
OMe
NC

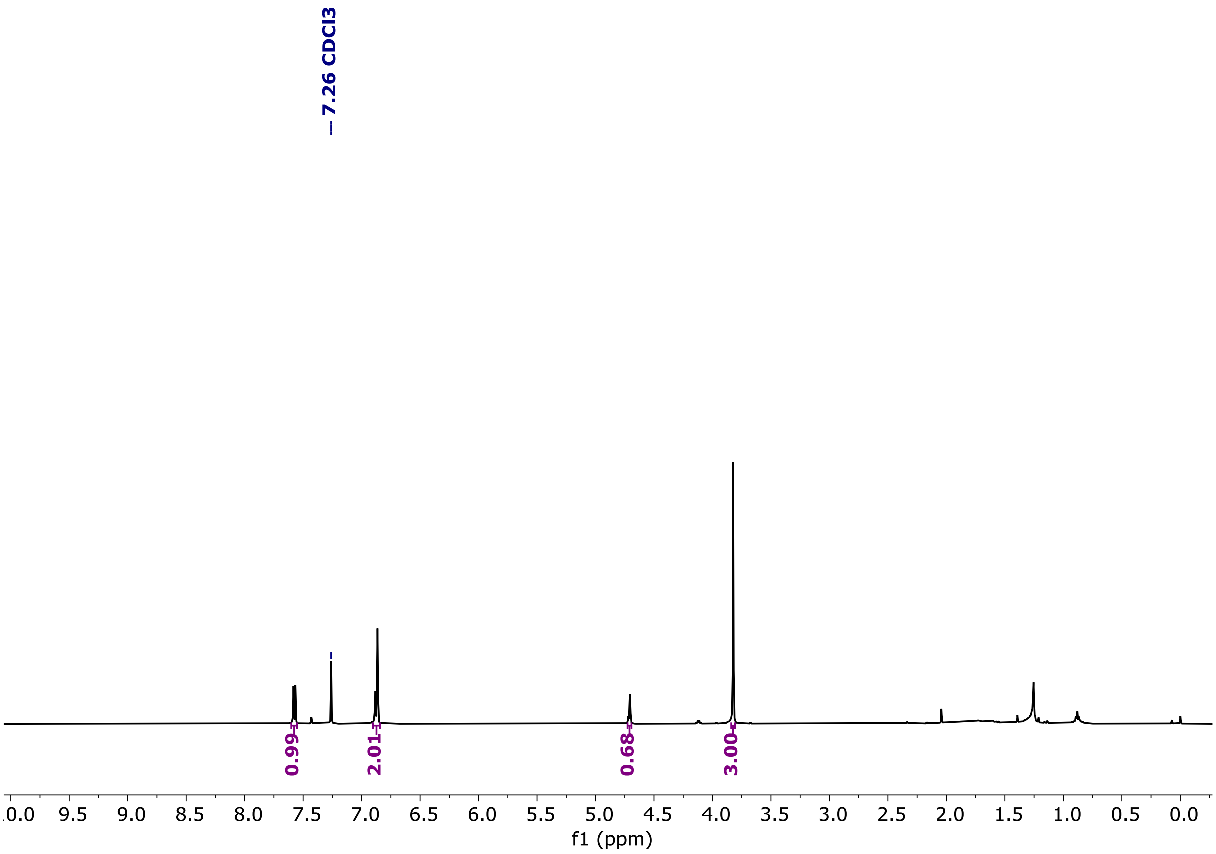

7.26 CDCl3
0.99
2.01
0.68
3.00
f1 (ppm)

d)

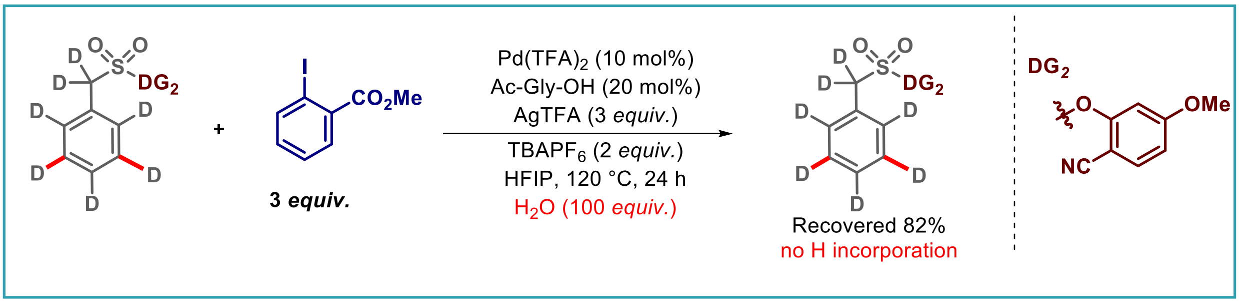

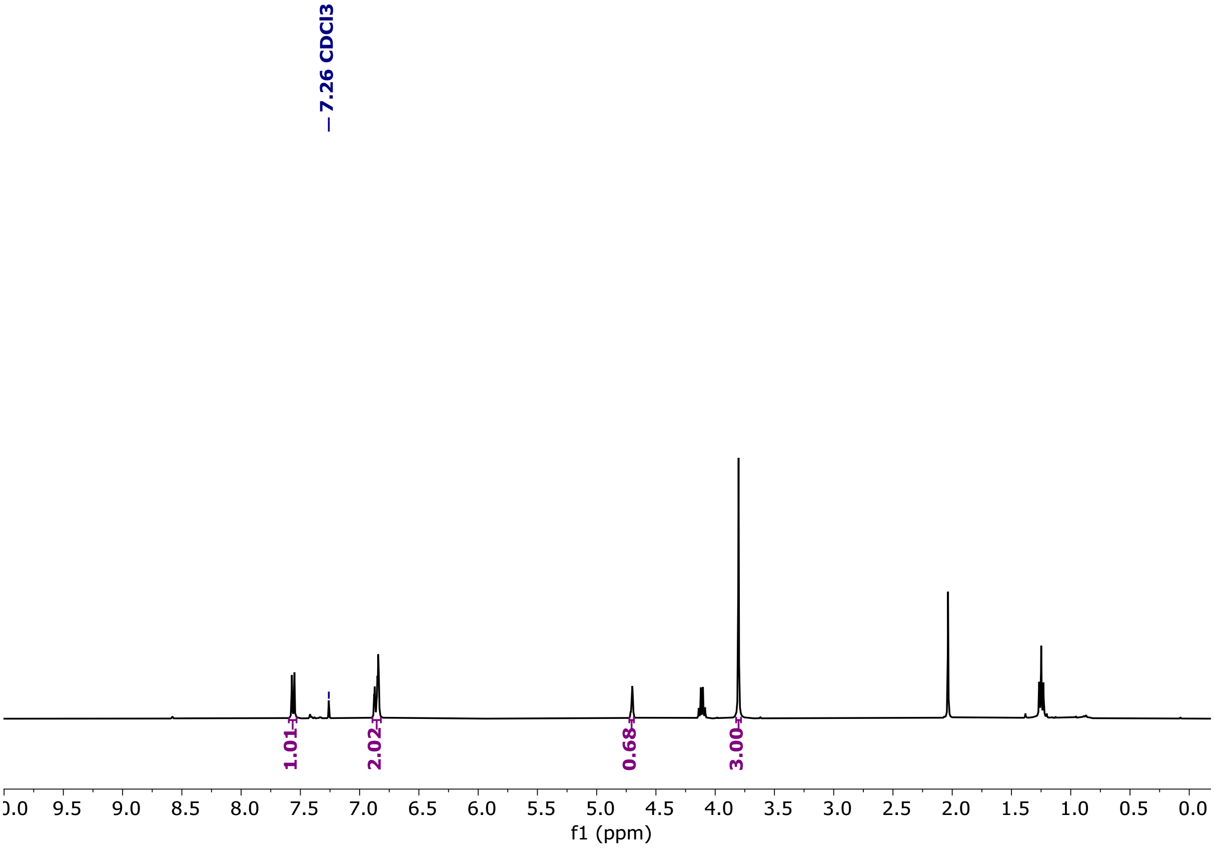

We performed reversibility experiment using 100 *equiv.* D_2O under standard reaction conditions, both in absence as well as presence of aryl iodide.

a) In absence of aryl iodide, no [D] incorporation was observed.

b) In presence of aryl iodide, trace amount of product formation (yield 7%) and no [D] incorporation was observed.

We also performed the reversibility experiment using deuterated substrate and 100 *equiv.* H_2O under standard reaction conditions, both in absence as well as presence of aryl iodide.

c) In absence of aryl iodide, no [H] incorporation was observed.

d) In presence of aryl iodide, neither [H] incorporation nor product formation was observed.

With these experiments we concluded that the presence of D_2O or H_2O was detrimental for our *meta*-arylation reaction. Therefore, these experiment cannot predict the reversibility of C–H activation step.

8. Failed Aryl iodide coupling partners

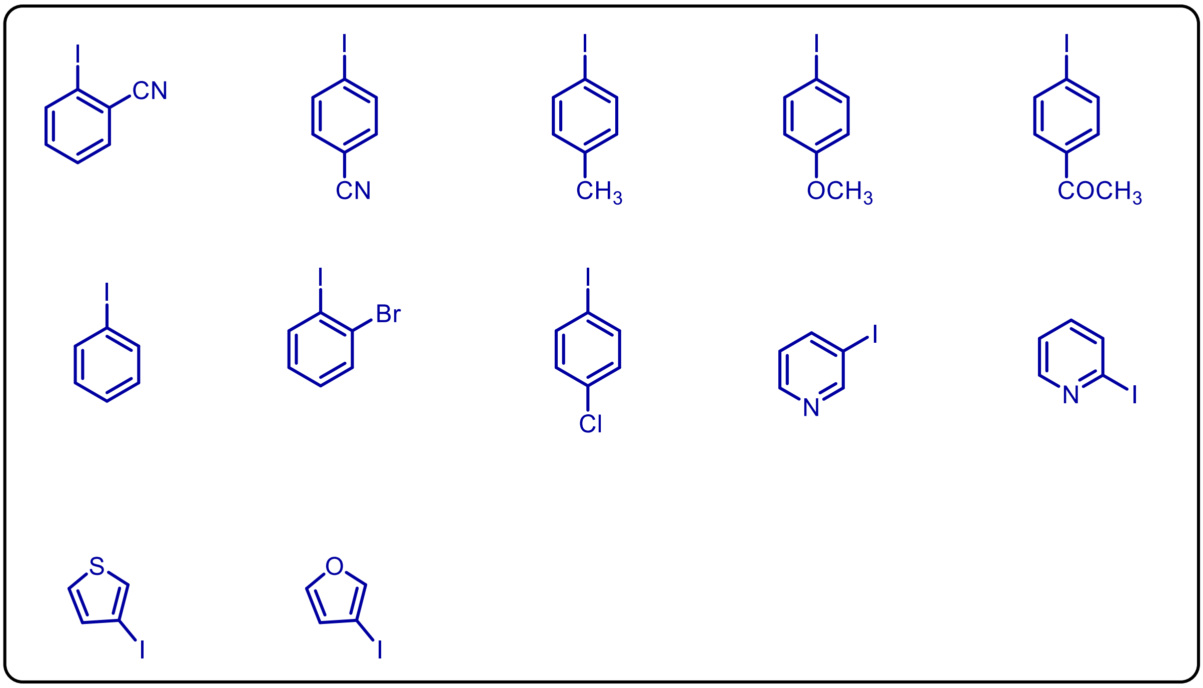

9. Computational Details

All the calculations were performed with the Gaussian 16 program.[1] Structure optimization was performed using the PBE0 functional,[2] and the def2-svp basis set[3] for H, C, N, O, F and S; Ag and Pd were displayed using the SDD basis set.[4] Harmonic vibrational frequencies were calculated at the same level for all stationary points to confirm them as a local minima or transition structures. Key transition-state structures were confirmed to connect corresponding reactants and products by intrinsic reaction coordinate (IRC) calculations.[5] To improve the calculation accuracy, single point calculations were performed using the M06-2X[6] functional and the def2-tzvpp basis set[3] for H, C, N, O, F and S; Ag and Pd were displayed using the SDD basis set.[4] Furthermore, we have also considered the solvent effects in 1,1,1,3,3,3-hexafluoroisopropanol (HFIP) using the SMD solvation model,[7] for all single-point energy calculations. As HFIP is not defined by Gaussin, we included HFIP using the following parameters: eps = 15.7, epsinf = 1.257, H-bond acidity = 0771, H-bond basicity = 0.730, SurfaceTensionAtInterface = 14.7, CarbonAromaticity = 0, ElectronegativeHalogenicity = 0.500. The CYL View software was employed to show the 3D structures of the studied species.[8]

9.1. Schemes of Computed Reaction Pathways

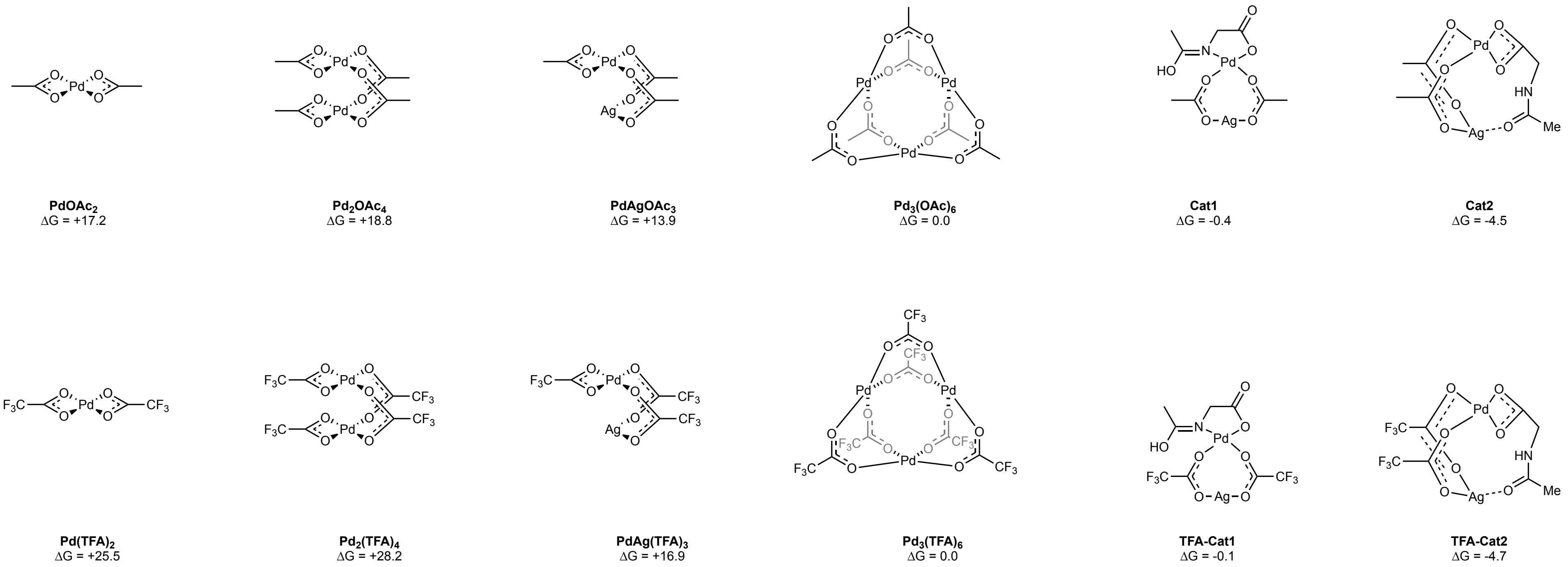

Figure S1: Active catalytic species; energies in kcal/mol.

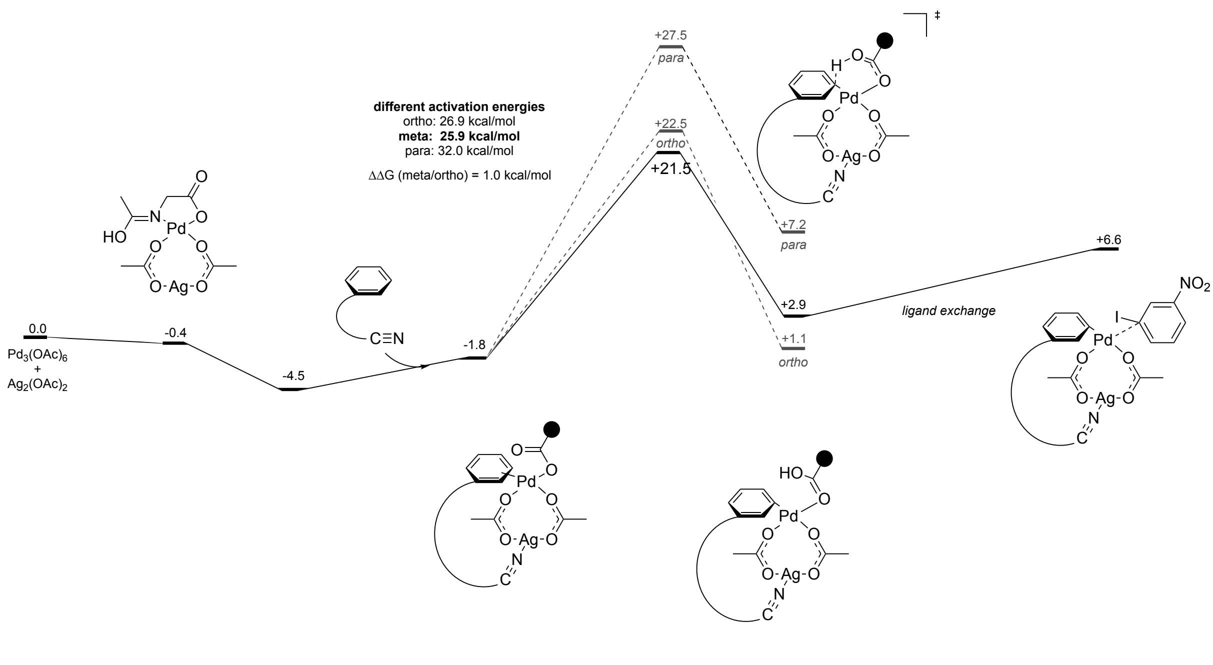

Figure S2:C-H activation pathway from $Pd_3(OAc)_6$ and $Ag_2(OAc)_2$; energies in kcal/mol.

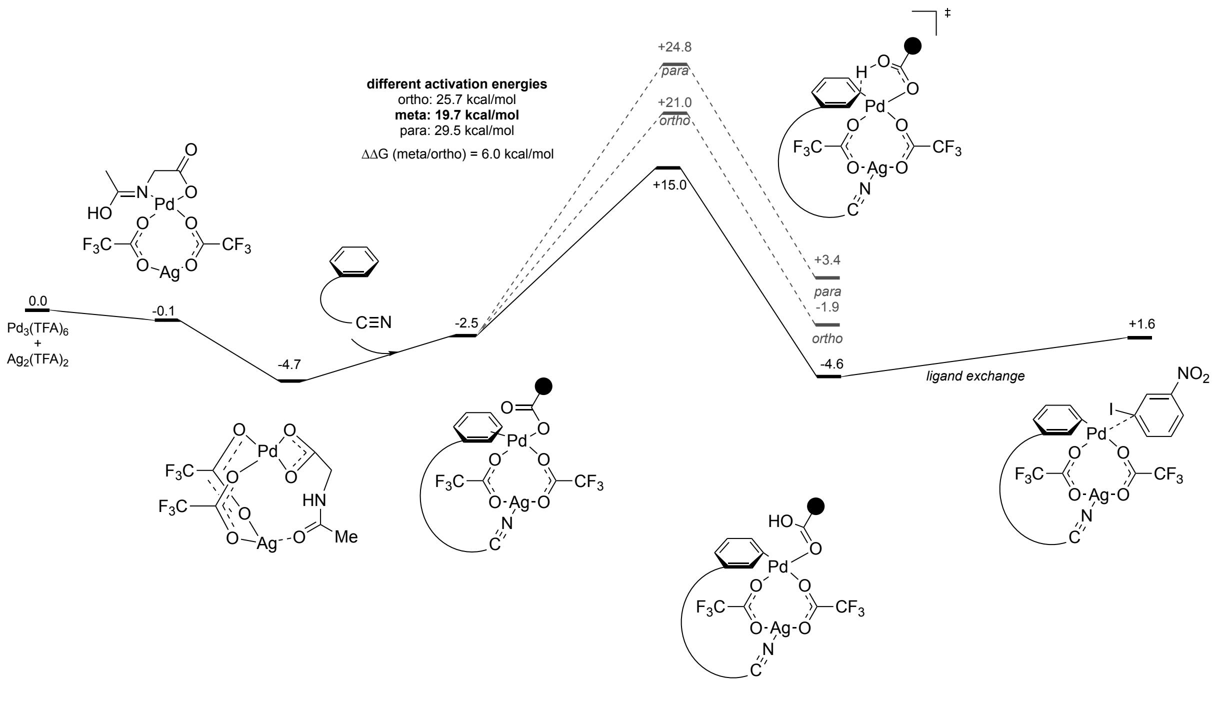

Figure S3: C-H activation pathway from $Pd_3(TFA)_6$ and $Ag_2(TFA)_2$; energies in kcal/mol.

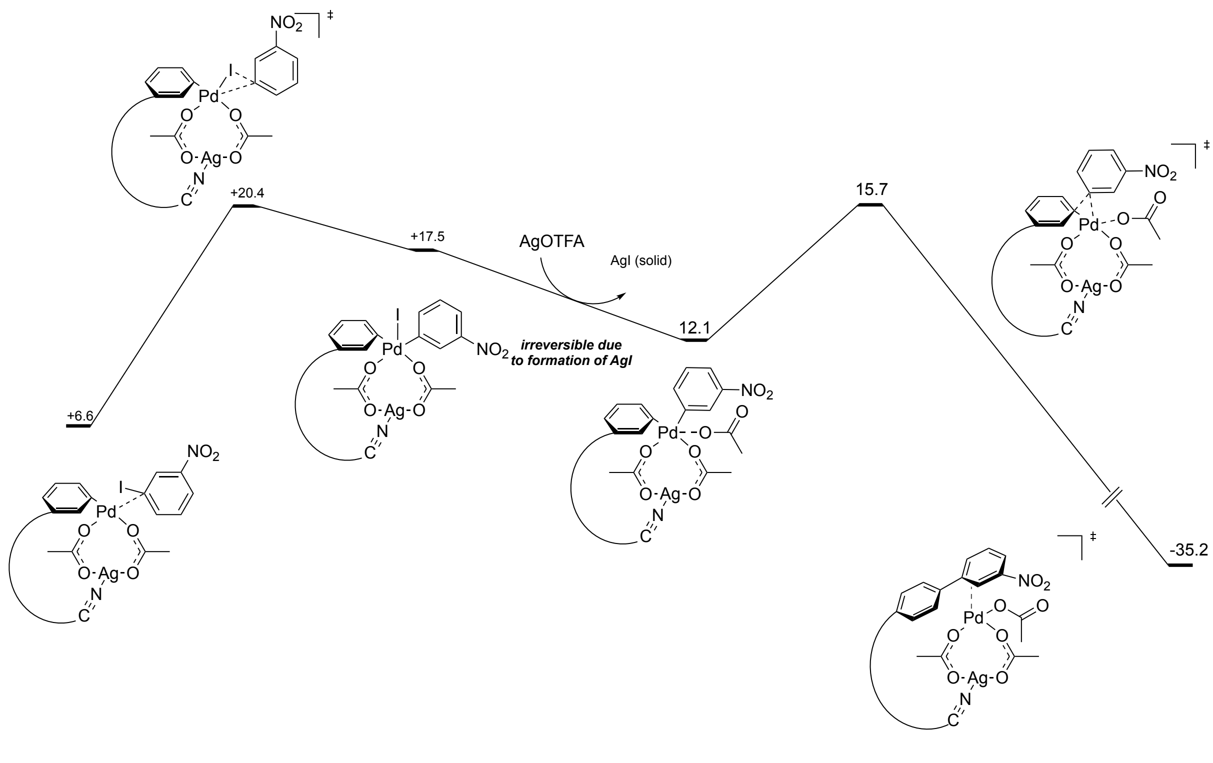

Figure S4: Further reaction pathway from $Pd_3(OAc)_6$ and $Ag_2(OAc)_2$; energies in kcal/mol.

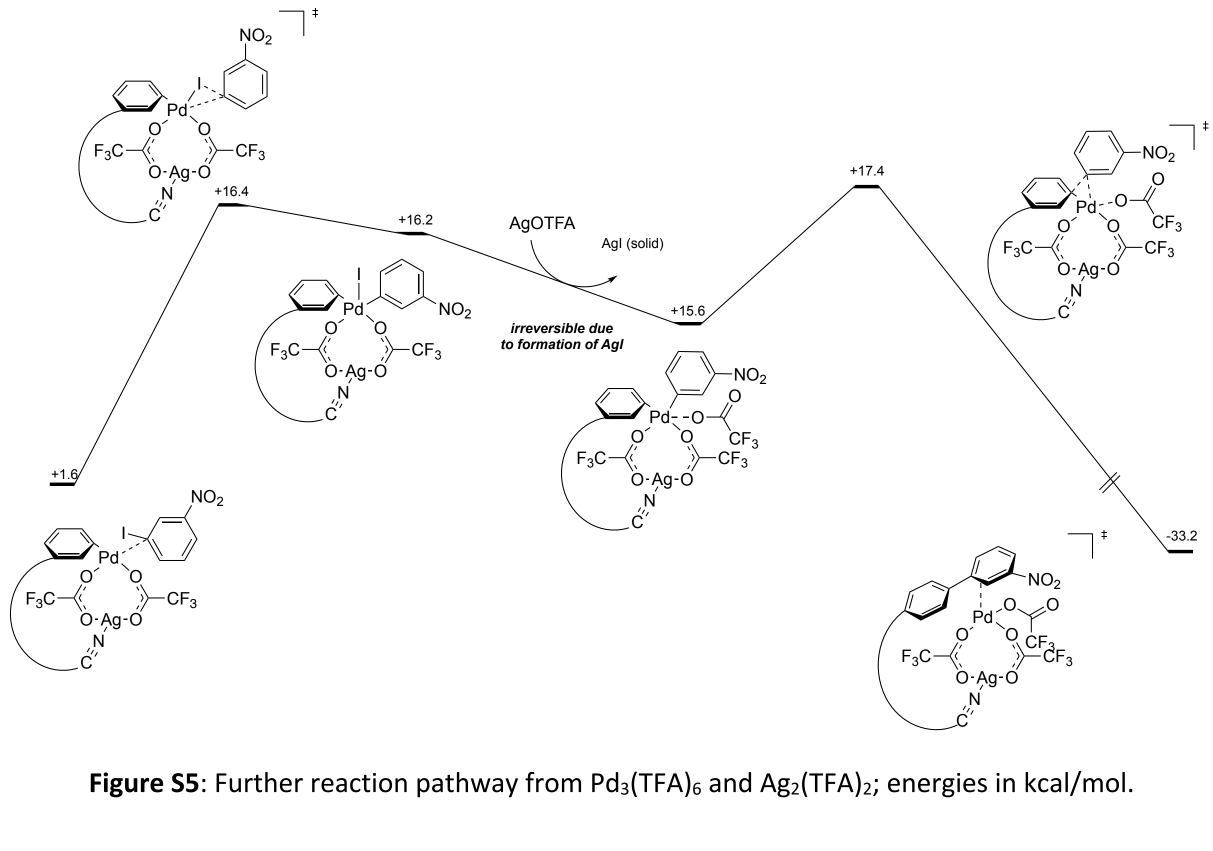

Figure S5: Further reaction pathway from $Pd_3(TFA)_6$ and $Ag_2(TFA)_2$; energies in kcal/mol.

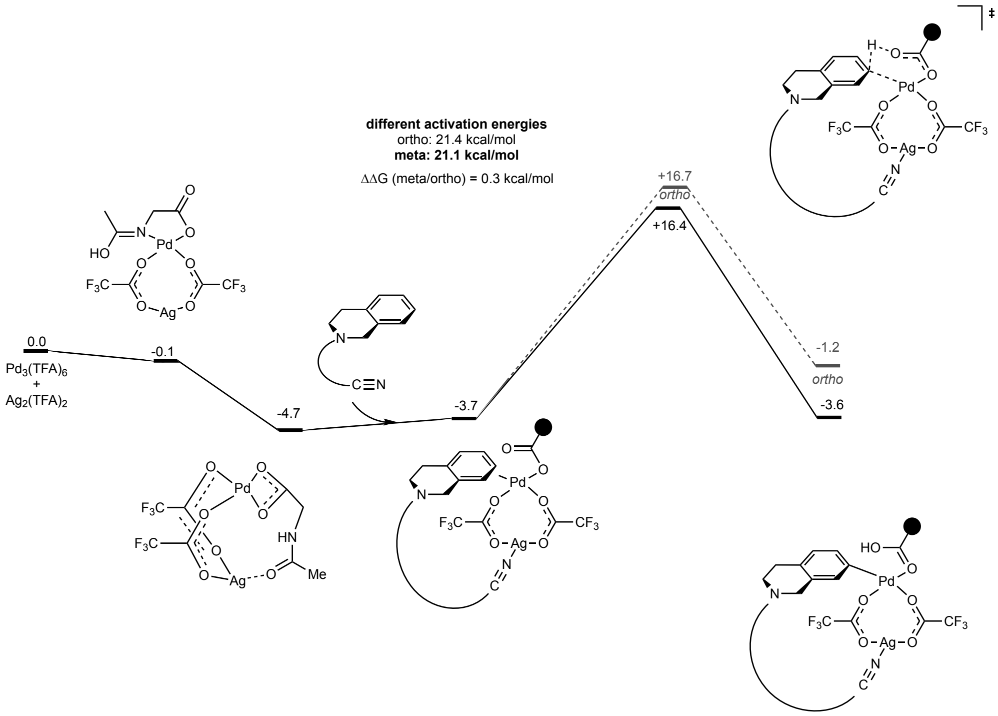

Figure S6: Calculated reaction pathway for the C–H activation of tetrahydro isoquinolines; energies in kcal/mol.

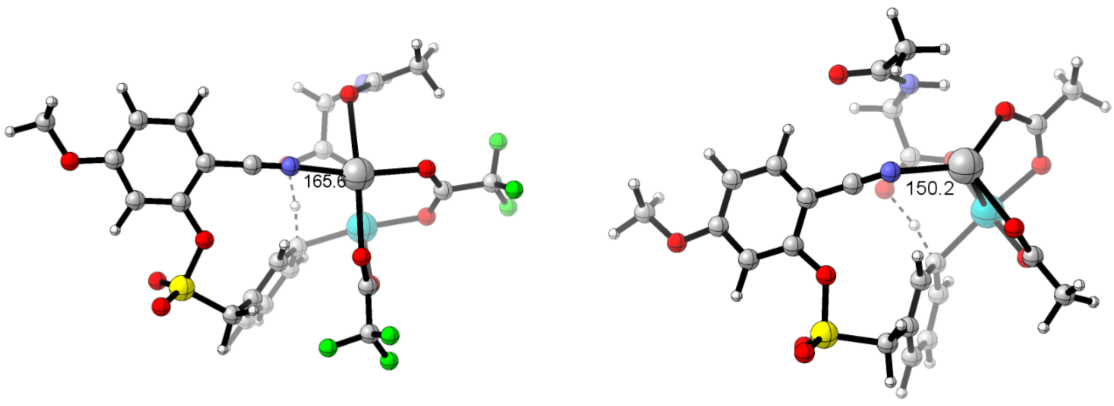

Figure S7: Angel of Ag-N-C bond in different transition states.

9.2. Computed Energies of all Stationary Points

Table 11 Calculated Energies of all Stationary Points for Reaction Pathways.
Thermal correction to Gibbs free energies (***TCG***, in Hartree), thermal correction to enthalpies (***TCH***, in Hartree), sum of electronic and thermal free energies (***G***, in Hartree), sum of electronic and thermal enthalpies (***H***, in Hartree), at the PBE0/ def2-2svp // SDD level, single point energies in HFIP computed at the M06-2X / def2-tzvpp // SDD level (E_{sol}, in Hartree).

Name	*TCG* / Hartree	*TCH* / Hartree	*G* / Hartree	*H* / Hartree	E_{sol} /Hartree
**AgOAc**	0.005813	0.060991	-375.016389	-374.961212	-375.410625
**Ag_2OAc_2**	0.039676	0.125197	-750.113281	-750.02776	-750.870284
**AgTFA**	-0.023223	0.041612	-672.270179	-672.205344	-673.184433
**$Ag_2(TFA)_2$**	-0.017297	0.08637	-1344.614931	-1344.511264	-1346.413872
**2AgI**	-0.052289	0.01256	-316.949786	-316.884937	-316.704331
**$PdOAc_2$**	0.048607	0.121646	-583.992592	-583.919553	-584.788147
**Pd_3OAc_6**	0.210945	0.37167	-1752.073325	-1751.9126	-1754.505811
**$Pd(TFA)_2$**	-0.009281	0.082886	-1178.474611	-1178.382443	-1180.302072
**$Pd_3(TFA)_6$**	0.036873	0.255193	-3535.559184	-3535.340864	-3541.086677
**$Pd_2(OAc)_4$**	0.12316	0.246241	-1168.013462	-1167.890381	-1169.624098
**$Pd_2(TFA)_4$**	0.009779	0.168643	-2356.987267	-2356.828403	-2360.665789
**$PdAg(OAc)_3$**	0.083275	0.185515	-959.051721	-958.949481	-960.241834
**$PdAg(TFA)_3$**	-0.002701	0.127335	-1850.79079	-1850.660754	-1853.53641
**AcOH**	0.02456	0.070512	-228.643091	-228.597138	-229.095598
**AcO**	0.01004	0.056167	-228.072556	-228.026428	-228.623569
**TFAH**	-0.003674	0.051023	-525.888302	-525.833604	-526.856315
**TFA**	-0.017437	0.037281	-525.355748	-525.301031	-526.408131
**Ligand1**	0.066624	0.133424	-436.240461	-436.173661	-437.119621
**Ligand2**	0.066142	0.132789	-436.205725	-436.139078	-437.097127
**Ligand3**	0.055681	0.119175	-435.695881	-435.632387	-436.65267
**Ligand4**	0.056084	0.117512	-435.640133	-435.578705	-436.625116
**XXX**	0.184941	0.289915	-1331.010337	-1330.905362	-1333.222925
**Cat1**	0.132599	0.248619	-1166.632158	-1166.516138	-1168.274005
**Cat2**	0.131111	0.248762	-1166.65439	-1166.536739	-1168.278913
**INT1**	0.340746	0.541198	-2497.678139	-2497.477686	-2501.519276
**$TS1_{meta}$**	0.346238	0.535771	-2497.656022	-2497.466489	-2501.487664
**$TS1_{ortho}$**	0.345178	0.535594	-2497.650275	-2497.45476	-2501.485078
**$TS1_{para}$**	0.350898	0.535452	-2497.63791	-2497.453356	-2501.482617
**$INT2_{meta}$**	0.350175	0.541544	-2497.673676	-2497.482306	-2501.521158
**$INT2_{ortho}$**	0.346796	0.541088	-2497.668146	-2497.473854	-2501.519298
**$INT2_{para}$**	0.353864	0.541748	-2497.6595	-2497.471615	-2501.518085
**$INT3_{meta}$**	0.321066	0.515962	-2508.142772	-2507.947876	-2511.899668
**TS2**	0.322766	0.514757	-2508.123134	-2507.931143	-2511.879322
**INT4**	0.319052	0.51671	-2508.142638	-2507.94498	-2511.880297
**INT5**	0.365185	0.572909	-2724.713498	-2724.505774	-2728.972005
**TS3**	0.367115	0.571596	-2724.705592	-2724.501112	-2728.968268
**INT6**	0.367254	0.574128	-2724.772492	-2724.565618	-2729.049481

**TFA-Ca1**	0.074616	0.210025	-1761.158423	-1761.023014	-1763.819108
**TFA-Cat2**	0.076396	0.209793	-1761.137296	-1761.0039	-1763.81352
**TFA-IN1**	0.283779	0.502365	-3092.182696	-3091.96411	-3097.059694
**TFA-TS1$_{meta}$**	0.28892	0.496659	-3092.16619	-3091.958451	-3097.036988
**TFA-TS1$_{ortho}$**	0.289007	0.49655	-3092.154695	-3091.947152	-3097.02752
**TFA-TS1$_{para}$**	0.294313	0.496676	-3092.148491	-3091.946129	-3097.026796
**TFA-INT2$_{meta}$**	0.28948	0.502273	-3092.184343	-3091.97155	-3097.068749
**TFA-INT2$_{ortho}$**	0.288608	0.502429	-3092.181089	-3091.967268	-3097.063648
**TFA-INT2$_{para}$**	0.299238	0.503206	-3092.172702	-3091.968734	-3097.069811
**TFA-INT3$_{meta}$**	0.264507	0.477267	-3102.654688	-3102.654688	-3107.447302
**TFA-TS2**	0.265454	0.476075	-3102.634549	-3102.423928	-3107.424731
**TFA-INT4**	0.259034	0.477788	-3102.646797	-3102.428043	-3107.418679
**TFA-INT5**	0.279601	0.514803	-3616.475311	-3616.240109	-3622.277473
**TFA-TS3**	0.283692	0.513546	-3616.466221	-3616.236367	-3622.278022
**TFA-INT6**	0.28562	0.516098	-3616.536178	-3616.305699	-3622.360643
**9**	0.234702	0.335462	-1313.339226	-1313.238466	-1315.56262
**TFA-INT7**	0.341595	0.548155	-3074.517729	-3074.311169	-3079.409487
**TFA-TS4$_{meta}$**	0.338953	0.542285	-3074.496465	-3074.293133	-3079.374709
**TFA-TS4$_{ortho}$**	0.339269	0.541994	-3074.496814	-3074.294089	-3079.374543
**TFA-INT8**	0.340351	0.548158	-3074.512817	-3074.30501	-3079.408058
**TFA-INT9**	0.341537	0.548321	-3074.515539	-3074.308755	-3079.405354

9.3. 3D Structure and Coordinates of all Stationary Points

AgOAc

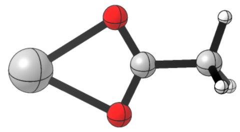

Charge: 0

Spin: 1

C 1.50204300 0.01171100 -0.00009800

O 0.89926400 -1.09715000 -0.00000300

O 0.90409100 1.11960000 -0.00007800

C 3.01229700 -0.00690600 -0.00005000

Ag -1.09920500 -0.00218200 0.00003300

H 3.42161100 1.00978700 -0.00055700

H 3.36403900 -0.55735200 0.88465500

H 3.36412700 -0.55833900 -0.88409700

Ag_2OAc_2

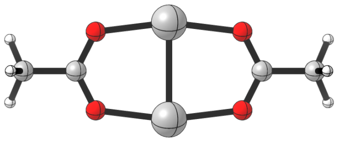

Charge: 0

Spin: 1

C -2.66668600 -0.00002600 -0.00426500

O -2.09957200 1.12606800 -0.00669600

O -2.09956900 -1.12611500 -0.00670500

O 2.09956900 1.12611600 -0.00668500

O 2.09957200 -1.12606800 -0.00669400

C 2.66668600 0.00002600 -0.00425100

C 4.17375400 -0.00000400 0.04001600

H 4.48438400 -0.00078400 1.09658600

H 4.57429500 -0.90449800 -0.43363300

H 4.57435300 0.90509700 -0.43241000

C -4.17375400 0.00000300 0.03999600

H -4.57429400 0.90450500 -0.43363900

H -4.57435100 -0.90509000 -0.43244700

H -4.48438800 0.00076500 1.09656400

Ag 0.00000600 -1.38694300 -0.00719400

Ag -0.00000600 1.38694300 -0.00718300

AgTFA

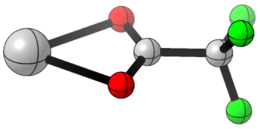

Charge: 0

Spin: 1

C 0.66060900 0.03094700 -0.00001500

O 0.10016300 -1.08638400 -0.00000900

O 0.08960700 1.13950400 -0.00002200

C 2.20427200 0.00446400 -0.00001200

F 2.72613200 1.22243400 -0.00069100

F 2.64717400 -0.63764000 1.07994200

F 2.64718000 -0.63886700 -1.07921300

Ag -1.93386900 -0.00320800 0.00000100

$Ag_2(TFA)_2$

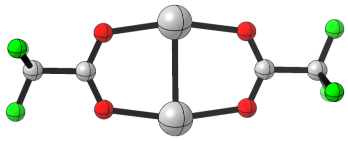

Charge: 0

Spin: 1

C -2.63742500 -0.02304000 0.17260900

O -2.11475500 1.11127100 0.15954600

O -2.09738100 -1.14721300 0.16650900

O 2.10281800 1.15477600 0.08561300

O 2.12016100 -1.10371000 0.09260200

C 2.64284900 0.03059900 0.07958000

C 4.18927200 0.00774400 0.05220500

C -4.18383400 -0.00026400 0.20073900

Ag 0.01602700 -1.41289700 0.13020900

Ag -0.01060800 1.42039300 0.12198000

F -4.60131400 0.64328900 1.28772600

F -4.64096900 0.64135200 -0.87124700

F -4.69769600 -1.21925700 0.21129100

F 4.60744300 -0.63941300 -1.03233600

F 4.64572800 -0.63026100 1.12667100

F 4.70311000 1.22672200 0.03790700

2AgI

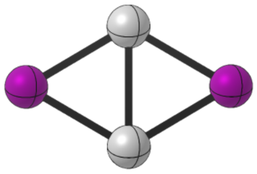

Charge: 0

Spin: 1

I -2.40987900 0.00065500 0.00021300

I 2.41032300 0.00057100 0.00021200

Ag -0.00017000 1.41930000 -0.00024000

Ag -0.00033100 -1.42068200 -0.00023900

$PdOAc_2$

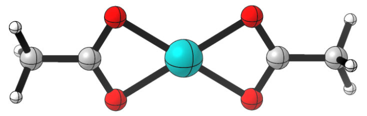

Charge: 0

Spin: 1

Pd 0.00000000 -0.00001600 -0.00003300

O -1.74751600 -1.07880200 -0.01917100

O -1.74749900 1.07878800 -0.01916000

C -3.90212600 0.00003100 0.01422800

H -4.22813500 0.00012900 1.06596100

H -4.29079700 0.90512100 -0.46915800

H -4.29087200 -0.90508900 -0.46903600

O 1.74746400 1.07880200 0.01912100

C 2.41580500 0.00003100 0.01897600

O 1.74753800 -1.07878000 0.01913700

C 3.90213500 0.00004900 -0.01402400

H 4.29076500 -0.90430400 0.47078800

H 4.22832800 -0.00168200 -1.06569400

H 4.29075700 0.90587300 0.46801300

C -2.41580200 -0.00000900 -0.01897400

Pd_3OAc_6

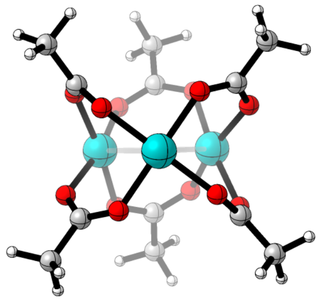

Charge: 0

Spin: 1

Pd -1.80263900 0.42519000 0.00071700

Pd 0.52740600 -1.77142400 -0.01830900

Pd 1.26468700 1.34398800 0.00813000

C 2.49078000 -0.65964500 1.85234800

O 1.67336100 -1.58872700 1.62559900

O 2.58713300 0.42714500 1.22737900

O 2.11575700 -1.54443800 -1.24349100

O 2.20576600 0.66818400 -1.63613400

C 2.53211800 -0.52795400 -1.85373300

C 3.55345100 -0.76147100 -2.93179100

H 3.37178400 -1.72251100 -3.42799500

H 3.54654000 0.06610200 -3.65107200

H 4.54592600 -0.79972900 -2.45659100

C 3.43781600 -0.85226300 3.00376500

H 3.51130900 -1.91094300 3.27729500

H 4.42267100 -0.44116100 2.74732900

H 3.05223500 -0.28073400 3.86187900

O 0.28183800 2.61938700 -1.20925500

O -1.67488500 1.59416700 -1.63144300

C -0.79338300 2.46666400 -1.84270600

C -1.03360000 3.41376800 -2.98510000

H -2.09131400 3.42089100 -3.27175300

H -0.69233600 4.42015000 -2.71026000

H -0.42661400 3.07884300 -3.84005800

O 0.52131400 2.22615300 1.65655200

O -1.67829400 2.01413700 1.23947700

C -0.69371700 2.46909500 1.87509800

C -1.00965200 3.37933000 3.02899100

H -1.83840900 4.04609600 2.75772900

H -1.34635400 2.75811300 3.87289700

H -0.12534500 3.95277300 3.32909300

O -0.91871200 -2.47310800 1.20243100

O -2.20455700 -0.67928200 1.63410400

C -1.80849600 -1.85540500 1.84074300

C -2.44465100 -2.59762200 2.98277000

H -3.38701300 -2.12433700 3.28088600

H -2.60223100 -3.64659000 2.70033600

H -1.74445200 -2.58419300 3.83191300

O -0.53946300 -2.22562400 -1.66211400

O -2.40836600 -1.04875700 -1.23765100

C -1.73458600 -1.89638000 -1.87668200

C -2.42760600 -2.56530100 -3.03077300

H -1.90560700 -3.48408800 -3.32119400

H -3.47282900 -2.77148700 -2.76669100

H -2.43084800 -1.86470300 -3.87972800

Pd(TFA)$_2$

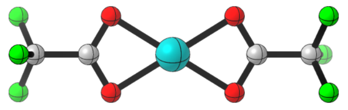

Charge: 0

Spin: 1

Pd -0.00000100 0.00003700 -0.00001200

O 1.75473400 1.08365900 -0.03933700

O 1.75469900 -1.08357600 -0.03932300

C 3.92765800 -0.00002800 -0.00193600

O -1.75467100 -1.08362900 0.03926900

C -2.39313900 -0.00003300 0.04331700

O -1.75476200 1.08361000 0.03928000

C -3.92765600 -0.00005400 0.00196900

C 2.39314200 0.00003700 -0.04333500

F 4.40253600 1.07885200 -0.59950600

F 4.40243500 -1.07883700 -0.59971200

F 4.32174000 -0.00016800 1.26723700

F -4.40248800 1.07791300 0.60125000

F -4.40243800 -1.07977400 0.59806800

F -4.32178300 0.00182100 -1.26718700

Pd$_3$(TFA)$_6$

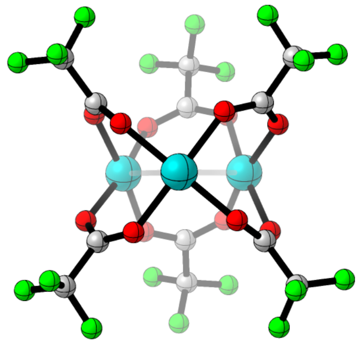

Charge: 0

Spin: 1

Pd 0.00138500 0.81685600 1.71479300

Pd -0.00245700 -1.89159800 -0.15188700

Pd 0.00561300 1.07844800 -1.56730600

C 1.84013800 -1.14322800 -2.30422200

O 1.59343400 -1.93077200 -1.36886300

O 1.29271800 -0.06771600 -2.60982100

O -1.29136700 -1.97683700 -1.69705400

O -1.59043900 0.15969400 -2.36549600

C -1.84045300 -1.06196300 -2.33875700

C -3.04285000 -1.47959100 -3.22071100

C 3.02710800 -1.58270600 -3.19649300

O -1.28091900 2.46170200 -0.86805300

O -1.59300000 1.96852300 1.31396400

C -1.83417900 2.56058200 0.24281500

C -3.02430000 3.54806900 0.32546100

O 1.60203300 2.14803700 -0.98738000

O 1.29089400 2.29137400 1.24508500

C 1.84525300 2.56222600 0.16377800
C 3.03959600 3.54523500 0.23808100
O 1.28457200 -2.22521200 1.36108900
O 1.59608200 -0.22107700 2.35468100
C 1.83802400 -1.42546400 2.13821200
C 3.02823600 -1.98333100 2.95698400
O -1.59845700 -2.11894200 1.04376000
O -1.28780400 -0.47756500 2.56373000
C -1.84161800 -1.48776500 2.09175500
C -3.03438400 -2.05233100 2.90220800
F -3.12667200 -1.47723500 4.08581800
F -4.15237300 -1.82439400 2.22116200
F -2.88932500 -3.35893600 3.06621400
F -4.14453600 2.84722500 0.46599500
F -2.87889800 4.34198100 1.37602300
F -3.11269000 4.28680900 -0.76382900
F 3.12936300 4.10364600 1.42975200
F 4.15708200 2.87005500 -0.00952300
F 2.89932900 4.49465100 -0.67523200
F 2.87599800 -1.67800400 4.23722600
F 3.12405400 -3.29325200 2.83497000
F 4.14729600 -1.42333100 2.50997600
F 2.85291000 -2.83627800 -3.58905000
F 4.14468900 -1.50555200 -2.48216200
F 3.14177500 -0.81041800 -4.25970300
F -3.09648200 -2.78931400 -3.36981800
F -2.95216400 -0.91164100 -4.41325300
F -4.15871900 -1.06473600 -2.62872000

$Pd_2(OAc)_4$

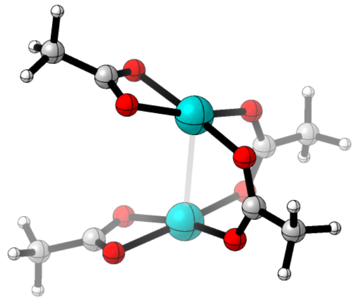

Charge: 0

Spin: 1

Pd 0.13953800 1.48209300 -0.02571900
O 1.54246300 1.14830500 1.38365500
O 1.50054100 -1.10170900 1.46299100
C 2.96526800 0.04739200 2.90151600
H 2.91604300 0.98184300 3.47300100
H 2.85723800 -0.82513300 3.55698800
H 3.94870100 -0.00932500 2.40978900
O -1.49293900 2.07876900 1.07251600
C -2.13618100 2.28570100 -0.00250800
O -1.52882800 2.04290100 -1.09064300
C -3.52469600 2.81867000 0.01227200
H -4.06488600 2.49082900 -0.88477200
H -4.04173200 2.49900700 0.92587700
H -3.47766500 3.91872500 0.00754400
C 1.90791600 0.03046900 1.83351900
C 1.90790200 -0.03046500 -1.83353100
O 1.50053400 1.10171000 -1.46299500
O 1.54244000 -1.14830300 -1.38367200
C 2.96526100 -0.04740800 -2.90152200
H 2.85741400 0.82525000 -3.55684700
H 2.91583300 -0.98174700 -3.47317500
H 3.94870400 0.00899900 -2.40978500
Pd 0.13953800 -1.48208800 0.02572200

O -1.52882700 -2.04290400 1.09065600

C -2.13618200 -2.28570000 0.00252500

O -1.49294500 -2.07876100 -1.07250500

C -3.52469100 -2.81868200 -0.01227400

H -4.04180700 -2.49875600 -0.92574500

H -3.47764800 -3.91873800 -0.00789400

H -4.06481300 -2.49111000 0.88490800

$Pd_2(TFA)_4$

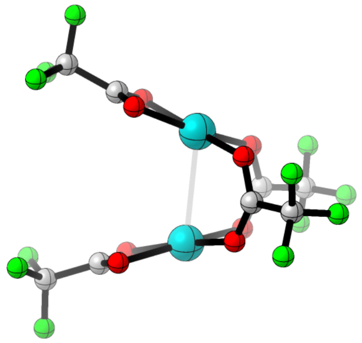

Charge: 0

Spin: 1

Pd 0.22453800 -1.50975300 0.00376900

O 1.60769800 -1.13305000 -1.41276000

O 1.60584600 1.12628100 -1.42026800

C 3.10135000 -0.00733300 -2.85900300

O -1.42804600 -2.09622000 -1.08189900

C -2.02361400 -2.32148900 0.00420000

O -1.42953400 -2.09106200 1.09002100

C -3.43744700 -2.91803700 0.00445800

C 1.97541700 -0.00432800 -1.79697100

C 1.97541000 0.00433000 1.79697400

O 1.60584900 -1.12628300 1.42026900

O 1.60768300 1.13304900 1.41276100

C 3.10133700 0.00735200 2.85901200

Pd 0.22452600 1.50974000 -0.00377200

O -1.42955300 2.09102300 -1.09003000

C -2.02363100 2.32146300 -0.00421100

O -1.42807100 2.09617800 1.08188900

C -3.43744900 2.91804600 -0.00446300

F -4.09653400 2.53099100 1.07399100

F -4.09562600 2.53272400 -1.08402000

F -3.34457400 4.24313900 -0.00333300

F -4.09567400 -2.53253600 1.08392200

F -3.34460300 -4.24313200 0.00353400

F -4.09646300 -2.53113000 -1.07409000

F 3.02750900 -1.08391000 -3.62110600

F 3.03241700 1.06850300 -3.62250600

F 4.26904400 -0.00957700 -2.22082500

F 4.26903500 0.00970600 2.22083900

F 3.03247300 -1.06852700 3.62246000

F 3.02742000 1.08388500 3.62116900

$PdAg(OAc)_3$

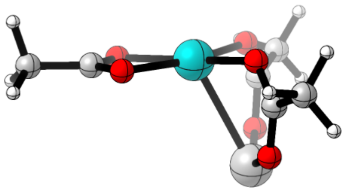

Charge: 0

Spin: 1

Pd 0.83665600 0.00027100 -0.58267100

O 2.38569700 1.08329700 0.23660000

O 2.38601700 -1.08229500 0.23644900

C 4.32505200 0.00059700 1.16737200

H 4.46052100 0.90908800 1.76754900

H 4.45478700 -0.90113800 1.77900400

H 5.08606300 -0.00739500 0.37159100

O -1.46712800 2.06219700 0.52535400

C -1.02702400 2.28263300 -0.62583200

O -0.30629200 1.49714700 -1.31843900

C -1.34213800 3.60409800 -1.27379700

H -1.42388900 3.49000400 -2.36185400

H -2.25865300 4.03077800 -0.84949500

H -0.50519900 4.28948500 -1.06827500

C 2.97967900 0.00058200 0.53355600

C -1.02591700 -2.28294000 -0.62604900

O -0.30559800 -1.49703700 -1.31857500

O -1.46597700 -2.06293500 0.52525200

C -1.34052800 -3.60446000 -1.27413400

Ag -1.47544000 -0.00037700 1.12648000

H -2.25829800 -4.03022600 -0.85160100

H -0.50467600 -4.29040700 -1.06602500

H -1.41966600 -3.49101700 -2.36243400

PdAg(TFA)₃

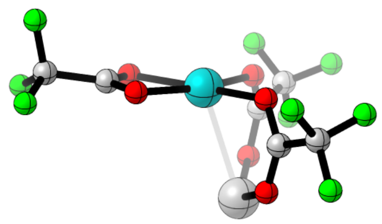

Charge: 0

Spin: 1

Pd 0.55244600 0.00015000 -0.61387800

O 2.27393600 1.08818500 -0.23659300

O 2.27429100 -1.08724600 -0.23664700

C 4.41747000 0.00084100 0.08812500

O -1.35539900 2.04237900 1.10383600

C -1.28155200 2.21201900 -0.11963600

O -0.76289500 1.47303700 -0.99939900

C -1.91371400 3.49341100 -0.71509400

C 2.89777100 0.00057600 -0.12562400

C -1.28055400 -2.21251700 -0.11966300

O -0.76230200 -1.47327300 -0.99946200

O -1.35440000 -2.04290800 1.10380700

C -1.91211900 -3.49422000 -0.71507600

Ag -1.09482400 -0.00020200 1.81807600

F 4.78659900 -1.07814500 0.75763400

F 4.78614900 1.07969600 0.75811200

F 5.01891400 0.00122100 -1.09644300

F -2.87859800 -3.16030000 -1.56403300

F -0.98992600 -4.18918000 -1.37055700

F -2.42607200 -4.25817300 0.23311200

F -2.87985200 3.15900200 -1.56425300

F -0.99177800 4.18891900 -1.37036400

F -2.42826700 4.25701900 0.23304200

AcOH

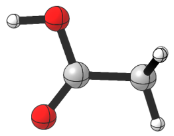

Charge: 0

Spin: 1

O 0.77572300 -1.03455600 -0.00000300

C 0.09332000 0.12214400 0.00003500

O 0.63788000 1.19357900 -0.00000500

C -1.38801000 -0.11038900 0.00001600

H -1.91155900 0.85153900 0.00065300

H -1.67245900 -0.69796700 -0.88519700

H -1.67243300 -0.69929500 0.88433500

H 1.71576900 -0.79698900 -0.00003200

AcO

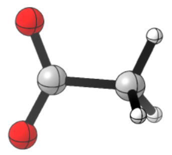

Charge: -1

Spin: 1

O 0.68968200 1.15708900 0.00005700

C 0.22041800 0.00178000 -0.00003300

O 0.80564800 -1.09802500 -0.00006300

C -1.34731800 -0.05751900 0.00000700

H -1.72487800 -1.09310500 -0.00062200

H -1.73810500 0.47691000 0.88388300

H -1.73825600 0.47812200 -0.88306200

TFAH

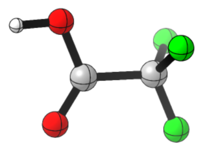

Charge: 0

Spin: 1

O -1.51129400 -1.03694900 -0.00008000

C -0.93298400 0.15612500 -0.00070200

O -1.48988900 1.21188400 -0.00001000

C 0.60014700 -0.00063300 -0.00002800

H -2.47177900 -0.89872400 0.00031800

F 0.99313500 -0.67247200 -1.07889900

F 1.17886700 1.18500400 -0.00015700

F 0.99225000 -0.67183300 1.07958600

TFA

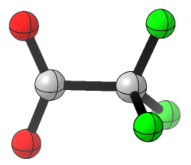

Charge: -1

Spin: 1

O -1.51765400 -1.14022500 -0.00002200

C -1.05800400 0.00980400 -0.00025500

O -1.58392500 1.12878700 -0.00001700

C 0.51383900 0.01407300 -0.00003300

F 1.02423300 -0.62304200 -1.07744400

F 1.07167100 1.23949700 -0.00041000

F 1.02383100 -0.62220600 1.07807900

Ligand1

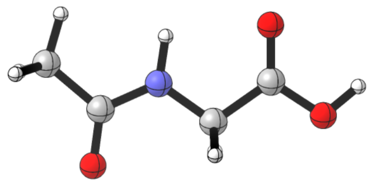

Charge: 0

Spin: 1

C 1.86263400 -0.14081600 0.00000400

O 1.94606700 -1.34230700 0.00004700

O 2.91805100 0.67173000 0.00004000

H 3.70961500 0.11153500 0.00009900

C 0.56808600 0.62451900 -0.00007300

N -0.53193800 -0.28785700 -0.00015600

H -0.30393900 -1.27582300 -0.00001200

C -1.81233600 0.16680400 -0.00001100

C -2.88622900 -0.89638200 0.00004000

H -2.49833300 -1.92441600 0.00005500

H -3.52286200 -0.74865300 0.88406300

H -3.52289000 -0.74871300 -0.88397200

O -2.06824600 1.35581900 0.00007200

H 0.55096700 1.29720600 0.87627700

H 0.55110100 1.29717100 -0.87645600

Ligand2

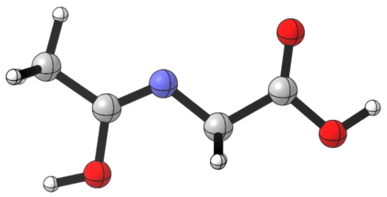

Charge: 0

Spin: 1

C 1.90392900 0.16208000 0.00015400

O 2.13357200 1.33754400 0.00033200

O 2.87360400 -0.77235600 -0.00021200

H 3.71411800 -0.29041300 -0.00010900

C 0.54834300 -0.50442200 -0.00017400

N -0.52036300 0.44421800 -0.00034400

C -1.70580000 0.01084200 -0.00003400

C -2.88888900 0.92747900 -0.00009100

H -2.54128900 1.96618400 -0.00043800

H -3.51528800 0.75607000 -0.89120500

H -3.51497500 0.75656600 0.89133800

O -1.97611400 -1.31969300 0.00033600

H 0.51713200 -1.18547800 -0.87243500

H 0.51681700 -1.18564800 0.87194500

H -2.92796200 -1.46663900 0.00052200

Ligand3

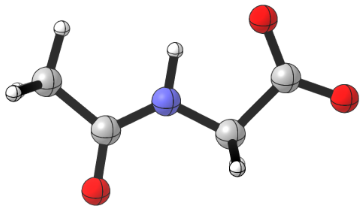

Charge: -1

Spin: 1

C -1.98711300 0.10363100 0.00004100

O -1.78716900 1.34319700 -0.00004100

O -3.04501600 -0.53459200 0.00024600

C -0.67681400 -0.76159600 -0.00017100

N 0.44020100 0.13901200 -0.00033100

H 0.05825400 1.09854600 -0.00031900

C 1.73724400 -0.18058300 -0.00005900

C 2.68745500 1.01075400 0.00015900

H 3.33778000 0.94041400 0.88536200

H 3.33931900 0.93951300 -0.88382400

H 2.17793000 1.98516300 -0.00072500

O 2.18160100 -1.32419300 0.00009100

H -0.67716700 -1.42263700 0.88405800

H -0.67748100 -1.42261100 -0.88441700

Ligand4

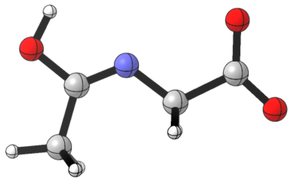

Charge: -1

Spin: 1

C 2.05239000 -0.06402400 -0.00007800

O 2.19845900 -1.29311000 -0.00041100

O 2.89115000 0.86019100 0.00007700

C 0.58279000 0.49621100 0.00024100

N -0.48748900 -0.47196500 0.00016900

C -1.69047000 -0.10538800 0.00003900

C -2.23228200 1.29470600 -0.00006700

H -1.85862800 1.83940300 -0.88149800

H -3.33023500 1.30093700 -0.00030600

H -1.85901900 1.83938400 0.88154300

O -2.67367600 -1.05026200 0.00000400

H 0.51258300 1.16913500 0.87937200

H 0.51238000 1.16956400 -0.87854700

H -2.16668900 -1.87824600 0.00008500

XXXXX

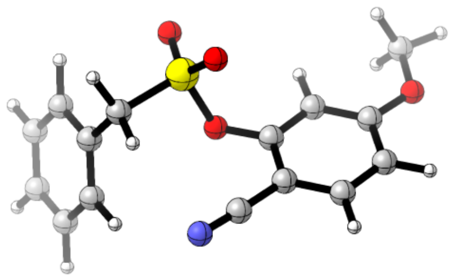

Charge: 0

Spin: 1

C 4.62304000 0.23009200 1.92345500

C 3.92528400 -0.95570100 1.69791900

C 3.08073600 -1.07217700 0.59594800

C 2.93766300 -0.00213300 -0.29345700

C 3.63542800 1.18844600 -0.06118200

C 4.47495300 1.30248800 1.04336100

H 5.28453700 0.32018700 2.78868600

H 4.03654000 -1.79762500 2.38535500

H 2.52511000 -1.99711700 0.41958100

H 3.50435300 2.03262700 -0.74222500

H 5.01708500 2.23483400 1.21845000

C 2.02330300 -0.13038100 -1.47163100

H 1.70107000 -1.16426300 -1.65825300

H 2.45107800 0.28734400 -2.39698800

S 0.50108400 0.80094200 -1.30057300

O 0.78551400 2.19605100 -1.03897900

O -0.43711600 0.40773300 -2.33403100

C -1.32789000 -0.29214700 0.21567500

C -2.34005300 0.61167700 0.51329200

C -1.59546200 -1.65413800 0.01726300

C -3.65671700 0.15036600 0.63001500

C -2.92140500 -2.10349700 0.14614300

C -3.93647000 -1.21671100 0.44940700

H -3.14279600 -3.16178000 -0.00199400

H -4.97054000 -1.54918700 0.55008800

C -0.51796600 -2.52225400 -0.32268500

N 0.38776500 -3.19029200 -0.60673900

O -0.03590100 0.14777400 0.14544500

C -4.49853400 2.31618500 1.08138500

H -5.48314400 2.75250900 1.28903400

H -3.82651000 2.52608200 1.93128500

H -4.08395600 2.78062200 0.17027900

H -2.06368900 1.65674400 0.64606700

O -4.70203300 0.93535400 0.91100500

Cat1

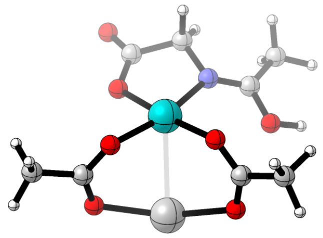

Charge: 0

Spin: 1

Pd 0.32491700 0.43841900 0.60381500

O -0.97329400 -2.54569900 -0.26483300

C -0.43610800 -2.45623100 0.86421100

O -0.01609100 -1.40180700 1.42352200

C -0.23012300 -3.72421700 1.65400200

H -0.47071500 -3.55292500 2.71150000

H -0.83295800 -4.54325000 1.24482000

H 0.83603700 -3.99311700 1.59929700

C -2.33061800 1.67498000 0.38358300

O -1.46659900 1.21391200 1.19377700

O -2.56241400 1.23606100 -0.76782300

C -3.12052200 2.85572700 0.87472400

Ag -1.71696300 -0.70969800 -1.08299600

C 2.01716700 2.51343500 -0.23731400

O 0.75640400 2.25254700 -0.06411400

O 2.47599000 3.57731000 -0.57435300

C 2.96161700 1.33571200 0.05722400

N 2.23672700 0.07527300 0.03397400

C 2.77839600 -0.99875700 -0.40286200

C 4.20520300 -1.09734400 -0.85482200

H 4.88202900 -0.62979200 -0.12701300

H 4.50909400 -2.14565400 -0.98153000

H 4.33941200 -0.58035200 -1.81796700

O 2.02745800 -2.08918000 -0.48294800

H 3.78742000 1.37751700 -0.66642900

H 3.38276300 1.50412800 1.06397000

H -3.31917600 2.76239400 1.95002700

H -2.49542300 3.75023000 0.72702100

H -4.05121600 2.96899700 0.30675200

H 2.52501200 -2.82152800 -0.86121000

Cat2

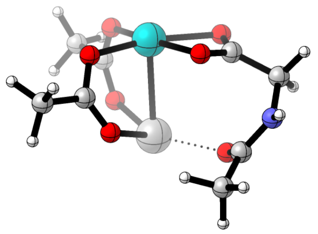

Charge: 0

Spin: 1

Pd -0.68921000 -0.54627400 -1.13200600

O 1.31321200 -0.48877500 -1.63103100

O 0.48262700 -2.29338100 -0.73273500

C 2.88576300 -1.95595700 -0.57476900

H 2.80000200 -2.62119100 0.29515000

H 3.41331900 -2.49656300 -1.37343200

O 0.14407400 2.19080500 0.00412100

C -0.63013800 2.29234800 -0.95815400

O -1.23099300 1.32636100 -1.55158600

C -0.91930800 3.64638300 -1.55926500

H -1.97295900 3.71800200 -1.85897200

H -0.65989700 4.44006600 -0.84862100

H -0.30752300 3.76165600 -2.46721400

C 1.48342500 -1.59253600 -1.02857700

C -2.83643000 -0.74970900 0.78044000

O -2.52673900 -0.91807100 -0.44539300

O -2.09209500 -0.33105700 1.68549000

C -4.24817200 -1.14407100 1.13470600

Ag 0.00757500 0.34608700 1.48173300

C 3.10416200 0.08572300 0.68490600

N 3.61316500 -0.75339000 -0.24504700

O 2.20967300 -0.29976300 1.44231700

C 3.62082800 1.48958100 0.72503600

H 4.53429200 1.64275000 0.13529600

H 2.81432600 2.12894800 0.32746300

H 3.79540600 1.78244700 1.76871700

H 4.23673200 -0.36486300 -0.94011000

H -4.63821300 -0.46803600 1.90621800

H -4.22390400 -2.16058400 1.55677100

H -4.89496400 -1.14593100 0.24950100

INT1

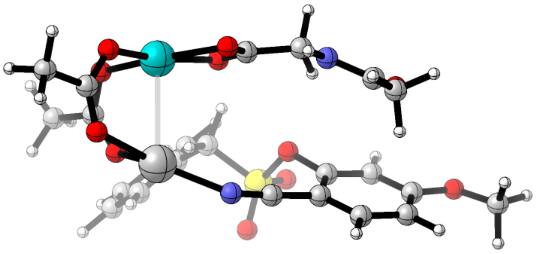

Charge: 0

Spin: 1

Pd 2.18364400 -1.44149200 -1.17049400

O 3.45443700 -2.32340000 1.63529100

C 3.94602900 -2.88163600 0.64129400

O 3.56576300 -2.74674500 -0.56918600

C 5.13003000 -3.79664700 0.82539800

H 5.15009600 -4.57390300 0.05162400

H 6.04475800 -3.19200100 0.72528900

H 5.11353200 -4.23783300 1.82934300

C 3.54568000 0.85450000 -0.14841100

O 3.57605500 -0.01884200 -1.08374400

O 2.73953100 0.87563100 0.79493000

C 4.57570600 1.94754400 -0.27675300

Ag 1.64719400 -1.04259400 1.66739600

C 2.14811300 6.22968300 -0.21248100

C 2.39709700 5.21898200 0.71541300

C 1.77280500 3.97982000 0.58652800

C 0.90064400 3.74391300 -0.48206000

C 0.64442400 4.76436200 -1.40468500

C 1.27006500 6.00099300 -1.27241500

H 2.63933500 7.20050200 -0.10975700

H 3.08417800 5.39449500 1.54673400

H 1.98025500 3.17385200 1.29439400

H -0.05639900 4.58734600 -2.22414800

H 1.06946500 6.79168700 -1.99922300

C 0.20565300 2.42138200 -0.59999400

H 0.73531400 1.61403600 -0.07520700

H 0.00599600 2.10793600 -1.63346000

S -1.40015900 2.54285100 0.17113100

O -1.30641500 2.52621600 1.61790300

O -2.23451400 3.51615100 -0.50421600

C -3.05477700 0.53402700 0.32958200

C -4.31492100 0.88788400 -0.09876200

C -2.87569700 -0.39625100 1.37674600

C -5.43844700 0.27759600 0.47916200

C -4.00440900 -0.98350500 1.96136800

C -5.27699600 -0.65678300 1.51725700

H -3.87618100 -1.70355500 2.77169300

H -6.14015200 -1.13599200 1.97743200

C -1.55051600 -0.70484300 1.76868400

N -0.44370300 -0.92553800 2.03182800

O -1.94390000 1.01502600 -0.30200100

C -7.78739100 -0.00479200 0.40849800

H -8.61124300 0.42214100 -0.17606900

H -7.73791900 -1.09092500 0.22142000

H -7.97981500 0.17497600 1.48029200

H -4.43749900 1.59573800 -0.91738900

O -6.61668600 0.64045200 -0.02535700

H 4.07337700 2.84862500 -0.66151300

H 5.38023800 1.66275300 -0.96469200

H 4.97622100 2.19030000 0.71631600

C -0.15694800 -1.54544600 -1.84358800

O 0.43883200 -2.57924000 -1.41818200

O 0.49639300 -0.46683500 -1.93108600

C -1.59697200 -1.62167500 -2.25582100

N -2.24416900 -2.73114200 -1.61584700

H -1.65357800 -3.50980600 -1.34761300

C -3.56774100 -2.71840100 -1.31010800

C -4.07153500 -3.92656800 -0.55565200

H -4.38626700 -3.59691800 0.44551000

H -4.96347900 -4.31203100 -1.06828400

H -3.33143400 -4.73186200 -0.45208400

O -4.30008900 -1.79163500 -1.60721600

H -2.10317700 -0.68201800 -1.99714700

H -1.61834200 -1.69489200 -3.36060500

TS1$_{meta}$

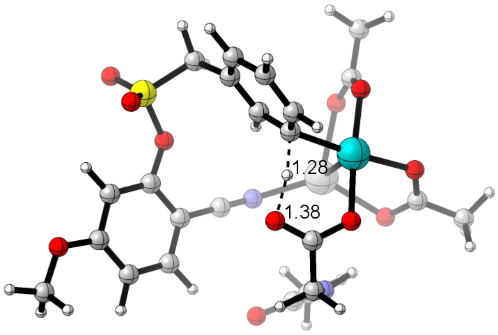

Charge: 0

Spin: 1

Pd -2.26488000 -0.73527900 1.11026900

O -2.37411900 -1.68493600 -1.87407900

C -2.68132200 -2.57486100 -1.06507600

O -2.69236100 -2.47124900 0.20684500

C -3.03993700 -3.94882200 -1.57719700

H -2.14301200 -4.58460000 -1.51397300

H -3.81781500 -4.40510500 -0.95182300

H -3.36040700 -3.89275000 -2.62432300

C -4.40105600 1.00261900 0.13463900

O -4.17996500 -0.07601100 0.75903500

O -3.57651600 1.62000500 -0.58199900

C -5.77778300 1.59076300 0.31491600

Ag -1.80723300 0.49284900 -1.45235200

C -0.19739900 -2.36269000 2.57822200

C -0.32761000 -1.57095800 1.42093000

C 0.38823700 -1.95433000 0.27203100

C 1.22660400 -3.06555100 0.27083100

C 1.32737400 -3.84031400 1.43365200

C 0.60575900 -3.50011700 2.57655800

H -0.72107200 -2.07111200 3.49283300

H -0.18413900 -0.30474000 1.58306000

H 0.31917000 -1.34374300 -0.63006500

H 1.99292100 -4.70773100 1.45004500

H 0.69441900 -4.11324500 3.47621900

C 2.07562500 -3.35545300 -0.92474000

H 1.61468900 -3.06908000 -1.88090200

H 2.40206400 -4.40458200 -0.98944300

S 3.63672500 -2.46432700 -0.87397800

O 4.19172700 -2.49191400 0.46404800

O 4.43491700 -2.81424400 -2.02674700

C 3.51047500 0.21495400 -0.66896000

C 4.79380700 0.35614900 -0.17508000

C 2.63966100 1.32899300 -0.71530400

C 5.21839900 1.60715400 0.30419000

C 3.07510900 2.57075600 -0.23156300

C 4.35577800 2.71450900 0.27961100

H 2.37656100 3.41251600 -0.25673100

H 4.67219900 3.68679200 0.65473600

C 1.33478600 1.18952600 -1.24438000

N 0.26547900 1.07814700 -1.68143200

O 2.99772000 -0.94738700 -1.16538400

C 6.99235500 2.84497800 1.27197300

H 8.02055300 2.62915700 1.58695500

H 7.01098700 3.63559100 0.50242900

H 6.41736400 3.20171800 2.14351400

H 5.48814700 -0.48076800 -0.12772200

O 6.47174900 1.64134700 0.76581000

H -6.05523600 2.19571600 -0.55700300

H -5.75310600 2.24478300 1.20083700

H -6.51719900 0.80162700 0.49921400

C -0.85161600 1.62674300 1.88197400

O 0.21883800 1.00410900 1.72890200

O -1.99181600 1.08250500 1.96722700

C -0.88028200 3.14225800 1.81731800

N -1.45510900 3.48424700 0.53860500

H -2.35221500 3.05865300 0.29871000

C -0.70553800 4.01634600 -0.46386200

C -1.41732300 4.15505800 -1.78601400

H -0.83914400 3.60258400 -2.54053400

H -2.44742800 3.77351600 -1.76652200

H -1.41536200 5.21455200 -2.07944000

O 0.45408500 4.36952200 -0.31028300

H -1.49084500 3.53506500 2.64488800

H 0.13342900 3.55636000 1.86166700

TS1$_{ortho}$

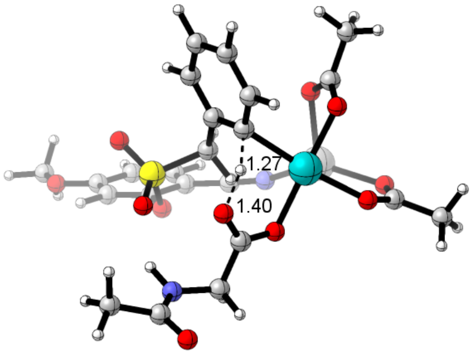

Charge: 0

Spin: 1

Pd -2.49801400 0.15767400 0.04458100

O -1.64304500 -2.55905500 1.18751900

C -2.74308400 -2.19839700 1.63535700

O -3.39961000 -1.16622500 1.26114500

C -3.39086500 -2.97803300 2.75237300

H -3.07512000 -2.52461200 3.70529300

H -4.48468900 -2.91372400 2.69315600

H -3.05625300 -4.02226000 2.73332000

C -3.84373400 -1.45938600 -2.08411100

O -3.77009700 -0.36415800 -1.45093400

O -3.01382800 -2.38858600 -2.06128600

C -5.08993700 -1.62714300 -2.91904500

Ag -1.06867700 -2.30024600 -1.07149500

C -1.88398100 0.70468900 4.24890100

C -2.30670800 1.00057500 2.95838800

C -1.46579100 0.79584900 1.84815000

C -0.17102100 0.28440100 2.07967900

C 0.25612900 -0.01001500 3.37566600

C -0.59833100 0.20020600 4.45540900

H -2.55180700 0.87134300 5.09735700

H -3.30758300 1.40659000 2.79315900

H -1.54416300 1.75520400 1.01797500

H 1.26222000 -0.40417000 3.53875400

H -0.25736300 -0.03293300 5.46715600

C 0.73633800 0.00438100 0.92068400

H 0.24168700 0.15920500 -0.04835200

H 1.13600800 -1.01811800 0.96515900

S 2.17630400 1.06361500 0.86302700

O 1.80316800 2.42618400 0.55949800

O 3.10778700 0.74612300 1.92666100

C 3.83110900 -0.42768400 -0.57799800

C 5.12120800 -0.08370200 -0.22574300

C 3.52534500 -1.74535900 -0.99172900

C 6.14498900 -1.04300200 -0.28909200

C 4.55720600 -2.69138200 -1.06157900

C 5.85667500 -2.35122600 -0.71656400

H 4.32861700 -3.70794900 -1.38657800

H 6.63868600 -3.10695500 -0.77898400

C 2.17815500 -2.08213500 -1.28159800

N 1.05640200 -2.32368500 -1.44733000

O 2.83618600 0.50406700 -0.58144800

C 8.44667800 -1.50926800 0.03928900

H 9.32034100 -0.93840800 0.37560500

H 8.29122700 -2.36254900 0.72107300

H 8.63473200 -1.88582500 -0.98053900

H 5.35052600 0.92922300 0.10330800

O 7.35675700 -0.61935800 0.06898000

H -4.92615200 -2.37163600 -3.70721300

H -5.40158400 -0.66469500 -3.34411800

H -5.89727200 -1.98055200 -2.25913600

C -1.14918500 2.51277300 -0.86936700

O -1.30392100 2.92938800 0.29413100

O -1.52550900 1.36752100 -1.27012900

C -0.43433200 3.37454400 -1.89196600

N 0.38965200 4.37316900 -1.29110400

H 1.06892600 4.04657200 -0.61216100

C -0.00066000 5.68175500 -1.26191000

C 0.82099400 6.56033700 -0.34793200

H 1.87804500 6.25917400 -0.31228900

H 0.73679800 7.60067400 -0.68401500

H 0.41092300 6.48922700 0.67192500

O -0.93754700 6.10379300 -1.91061900

H -1.19835600 3.89017900 -2.49694500

H 0.12892800 2.70711700 -2.56248800

TS1$_{para}$

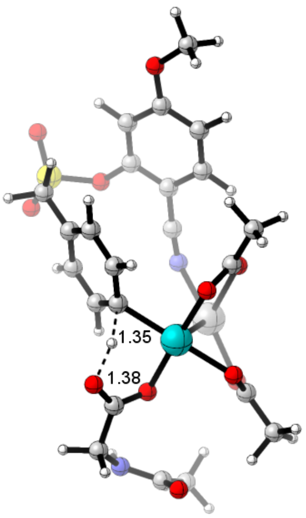

Charge: 0

Spin: 1

Pd -1.78491600 -0.45216100 1.05575000

O 0.06617600 -2.55106800 -0.34221400

C 0.04738600 -2.66419300 0.88798300

O -0.58629300 -1.91435400 1.70921500

C	0.87420400	-3.73628000	1.56566800
H	0.36211100	-4.11718100	2.45858000
H	1.08530700	-4.55405200	0.86511100
H	1.82696700	-3.28757100	1.88956200
C	-3.81532800	-1.80908600	-0.54998200
O	-3.22317900	-1.82576900	0.57087300
O	-3.34943100	-1.33194700	-1.61257100
C	-5.20675700	-2.37815900	-0.56035000
Ag	-1.23025200	-0.88452200	-1.73582000
C	-0.28485300	0.97517000	1.48207900
C	-0.02894900	1.92435800	0.47306400
C	1.23541000	2.46936800	0.28492400
C	2.29531300	2.05774800	1.10131100
C	2.06173500	1.10560200	2.10314700
C	0.78878500	0.58353600	2.30165600
H	-1.45703800	1.28702200	2.06338400
H	-0.84909100	2.24997000	-0.17269700
H	1.40614900	3.20307300	-0.50557900
H	2.88855800	0.77561700	2.73866400
H	0.62188600	-0.15962500	3.08411300
C	3.67480000	2.63510500	0.96913000
H	4.42854300	2.03214000	1.49305800
H	3.74134500	3.66868100	1.35046300
S	4.30339200	2.83906300	-0.70506900
O	3.56625100	3.86319200	-1.40435300
O	5.75045600	2.85905700	-0.64518500
C	4.07316900	0.20010500	-0.96631600
C	5.26057200	-0.14958200	-0.34799400
C	3.04789700	-0.75192400	-1.14210400
C	5.43403700	-1.46142400	0.12737100
C	3.23766000	-2.05763900	-0.67060000
C	4.41733300	-2.41637000	-0.03367400
H	2.41821200	-2.77044200	-0.78722200
H	4.53572000	-3.43578500	0.33171000
C	1.81557800	-0.40727400	-1.75501400
N	0.76991000	-0.22086400	-2.22129500
O	3.80858800	1.44578300	-1.45851200
C	6.88689000	-2.99319400	1.20542900
H	7.89721000	-2.95097000	1.62981600
H	6.17737200	-3.29086600	1.99636700
H	6.86720300	-3.74540900	0.39863500
H	6.07234500	0.56945500	-0.23981700
O	6.60732900	-1.70381500	0.71721200
H	-5.87654500	-1.58762100	-0.18158600
H	-5.27588800	-3.23316200	0.12408600
H	-5.50728300	-2.66014200	-1.57657600
C	-3.29115100	1.87723400	1.35925700
O	-3.08993800	0.98523400	0.48885800
O	-2.55771600	2.05911400	2.35813600
C	-4.51426200	2.75439500	1.14864700
N	-5.12580300	2.56519200	-0.13230500
H	-4.66267800	2.98650100	-0.92713200
C	-5.84337200	1.42019900	-0.37233100
C	-6.05498400	1.08210900	-1.82426700
H	-7.02412600	0.58124200	-1.93994400
H	-6.00649100	1.95730100	-2.48784300
H	-5.26238400	0.37088400	-2.11348900
O	-6.24718800	0.71592700	0.53269600
H	-5.23236600	2.47267700	1.93428900
H	-4.23719800	3.80523400	1.31395800

INT2$_{meta}$

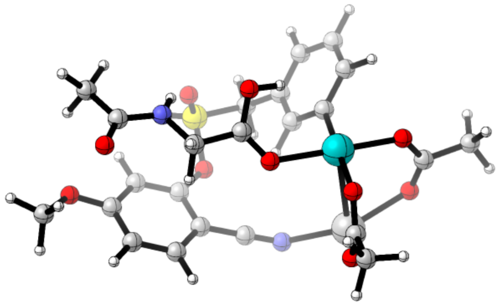

Charge: 0

Spin: 1

Pd 2.16788800 -0.16062100 1.08187100

O 4.33710800 0.42675300 -1.16808200

C 4.71054200 0.70963100 -0.01753100

O 4.05437700 0.53161700 1.06119800

C 6.05624300 1.36638700 0.17369200

H 6.52539700 1.02289700 1.10456100

H 6.70411500 1.16798000 -0.68824600

H 5.89928200 2.45323000 0.25596700

C 2.96526200 -3.02091600 0.60452000

O 2.78631700 -2.12340300 1.48099300

O 2.85711000 -2.88122000 -0.63041200

C 3.38245700 -4.37462800 1.13820400

Ag 2.58854600 -0.85982800 -1.65923800

C 2.10230700 2.77694500 1.51584200

C 1.56767300 1.70241600 0.78458700

C 0.56490100 1.97931500 -0.15325300

C 0.06481100 3.27398500 -0.32941800

C 0.58379400 4.32219600 0.43550000

C 1.61082900 4.07185400 1.34382300

H 2.91556100 2.59217900 2.22308200

H 0.28289000 1.06130700 2.39065400

H 0.19031700 5.33561500 0.31751800

H 2.02763400 4.89518800 1.92979900

C -1.03611800 3.54109600 -1.31022900

H -0.78851500 3.29491800 -2.35470900

H -1.39552500 4.58008900 -1.27470800

S -2.53300800 2.59999700 -0.99238000

O -2.64550900 2.21533200 0.40603700

O -3.66040100 3.20435800 -1.66798200

C -2.70940400 0.03898200 -1.64263800

C -4.03078600 -0.07232400 -1.25114000

C -1.91702700 -1.11207000 -1.84924000

C -4.58215600 -1.34174100 -1.00837100

C -2.49295000 -2.37313900 -1.63227600

C -3.80334400 -2.49670700 -1.19823300

H -1.88076700 -3.26420400 -1.78269000

H -4.21284700 -3.48577800 -1.00029000

C -0.55709100 -0.98469800 -2.23242900

N 0.56355400 -0.88441900 -2.51779800

O -2.09314600 1.23825800 -1.86004300

C -6.43748300 -2.55960200 -0.16629000

H -7.45984300 -2.30551900 0.13746800

H -6.48117100 -3.29888500 -0.98307500

H -5.88888900 -2.95576300 0.70253000

H -4.65702300 0.81002600 -1.11801000

O -5.84907000 -1.34865700 -0.60185200

H 3.19244000 -5.16004400 0.39646700

H 2.86731700 -4.59374700 2.08282800

H 4.46289000 -4.34434600 1.34859900

C -0.70428200 -0.41841700 1.82362000

O -0.58614200 0.63619000 2.58013700

O 0.20015200 -0.86951100 1.12601500

C -2.04077200 -1.09420800 1.85072500

N -3.12388000 -0.17357800 2.03334100

H -2.98854000 0.79085800 1.74083300

C -4.37992100 -0.65060900 2.21861500

C -5.47359300 0.38667300 2.23667100

H -5.09825900 1.41750400 2.28517500

H -6.06884200 0.26269500 1.31959600

H -6.13383900 0.18851500 3.09163200

O -4.60101300 -1.84629400 2.33739000

H -2.03046600 -1.83174400 2.67334800

H -2.12836800 -1.68238000 0.92385100

H 0.14933600 1.16602200 -0.75050600

INT2$_{ortho}$

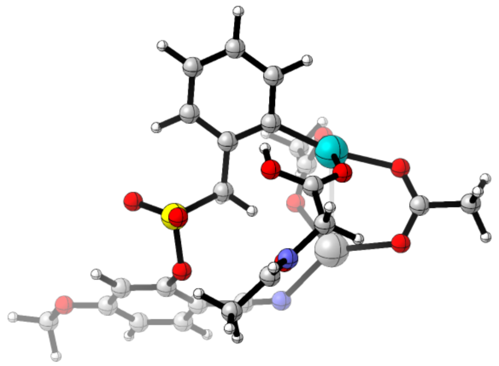

Charge:0

Spin: 1

Pd 2.52869500 0.50439400 -0.79188200

O 0.23470600 -1.05596300 -2.17111800

C 0.93613100 -0.43313100 -2.98566200

O 1.96728000 0.26306700 -2.71056300

C 0.54961500 -0.42628400 -4.44702500

H 0.02437700 -1.35440200 -4.70464500

H -0.13541900 0.41952200 -4.61589700

H 1.42965100 -0.28297000 -5.08630600

C 4.04844500 -2.08552700 -0.27895000

O 3.98055500 -1.01355200 -0.93847000

O 3.09735000 -2.65529500 0.30426300

C 5.41977500 -2.71451700 -0.17825600

Ag 1.04507100 -2.06084600 -0.14127700

C 1.57840300 4.52517100 -1.52122100

C 2.18419900 3.30350100 -1.23988500

C 1.44135500 2.17502200 -0.84469800

C 0.04120300 2.31730100 -0.72063400

C -0.56630300 3.54445100 -1.03814900

C 0.19278100 4.64050400 -1.43120300

H 2.18753300 5.38056400 -1.82486700

H 3.26965300 3.21433300 -1.34606400

H 1.51780900 2.34076000 1.13516500

H -1.65113400 3.64483000 -0.96968100

H -0.30266600 5.58572500 -1.66525300

C -0.79537000 1.13844400 -0.27949400

H -0.19886700 0.40099000 0.27629600

H -1.29367600 0.61977200 -1.11360800

S -2.08471100 1.60364000 0.86814700

O -3.13984600 2.35310700 0.20761700

C -3.71791300 -0.46621700 0.50266900

C -4.97029400 0.11089600 0.39353100

C -3.44170600 -1.70670000 -0.11452400

C -5.97237200 -0.53236500 -0.34949500

C -4.45391900 -2.34221500 -0.84348400

C -5.71030500 -1.76558200 -0.96868400

H -4.24367400 -3.30148500 -1.31960100

H -6.47758200 -2.28038800 -1.54566300

C -2.14777300 -2.27300900 0.02620300

N -1.07668100 -2.69806800 0.15114300

O -2.72584200 0.09773000 1.25034800

C -8.20821000 -0.46194000 -1.13288400

H -9.05205000 0.23253700 -1.04035000

H -7.95390700 -0.57586100 -2.20072500

H -8.50573300 -1.44329700 -0.72463700

H -5.18030600 1.07008400 0.86330100

O -7.14717800 0.10292500 -0.40504900

H 5.93739700 -2.64673400 -1.14452400

H 5.35156800 -3.75721200 0.15451200

H 6.01172300 -2.14089600 0.55175000

C 2.43016500 1.04523300 2.14464000

O 1.62704800 2.06520000 2.07899300

O 3.09058200 0.60520700 1.21110000

C 2.55040800 0.42621000 3.52029500

N 1.26479800 0.23943700 4.13576500

C 0.25829500 -0.32615400 3.42082100

C -1.06345800 -0.48981400 4.11058500

H -1.72680900 0.30704600 3.74412800

H -1.49606500 -1.45203900 3.80901500

H -0.99207100 -0.43601800 5.20545600

O 0.44630500 -0.65414200 2.25676100

H 3.08612000 -0.52562300 3.38350700

H 3.16219400 1.08742500 4.15164100

H 1.09143900 0.59939700 5.06322600

O -1.52572900 2.05222400 2.12420800

INT2$_{para}$

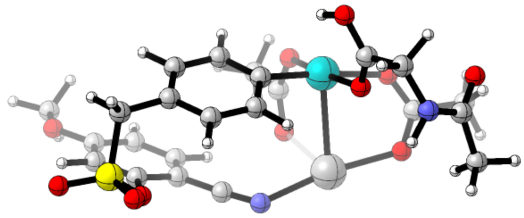

Charge: 0

Spin: 1

Pd -1.66366500 -0.54721300 1.08645400

O -0.05721900 -2.67353900 -0.52759200

C -0.06212300 -2.89528500 0.68846300

O -0.61841500 -2.17711000 1.58937200

C 0.69294500 -4.08427700 1.24308200

H 0.16543400 -4.51465900 2.10405600

H 0.84054300 -4.84065500 0.46229700

H 1.67721500 -3.73462100 1.59430200

C -3.97531500 -1.65819800 -0.42513100

O -3.39400800 -1.69869200 0.69125700

O -3.47620900 -1.21809000 -1.49831700

C -5.40492400 -2.13126000 -0.46156900

Ag -1.34222600 -0.89837800 -1.73283400

C -0.03958100 0.56263900 1.31066900

C 0.07847100 1.75178700 0.56950200

C 1.27261200 2.46477900 0.52016000

C 2.39795700 1.99793500 1.20624900

C 2.28374400 0.83525300 1.97572500

C 1.08026900 0.13517300 2.03934400

H -2.10613900 1.20328400 2.80319000

H -0.76131900 2.11208000 -0.02986300

H 1.34802300 3.35973300 -0.10269900

H 3.15781500 0.45548200 2.51408200

H 1.02282300 -0.78701600 2.62177000

C 3.72581400 2.67621800 1.07485800

H 4.53285100 2.12446200 1.57444400

H 3.73621500 3.71768000 1.43780600

S 4.27406200 2.90417300 -0.63417600

O 3.51671900 3.96047600 -1.26233100

O 5.72330400 2.89474400 -0.66934000

C 3.95936300 0.27648300 -0.97147800

C 5.14037400 -0.10249900 -0.35646200

C 2.93572600 -0.66908900 -1.19437200

C 5.30683600 -1.42881100 0.07641700

C 3.12081000 -1.99065800 -0.76594800

C 4.29109100 -2.37562300 -0.12721200

H 2.30362300 -2.69950300 -0.91886200

H 4.40289700 -3.40754300 0.20343200

C 1.69882300 -0.32573000 -1.80092400

N 0.64420200 -0.17618800 -2.26151100

O 3.70503900 1.54278400 -1.40603200

C 6.74397500 -2.99792400 1.12007300

H 7.74946200 -2.97364100 1.55743900

H 6.02430800 -3.31869500 1.89278600

H 6.73116300 -3.72456200 0.28985500

H 5.95473500 0.60806500 -0.21707900

O 6.47465500 -1.69326600 0.67045500

H -6.03343400 -1.28319400 -0.14400500

H -5.55431700 -2.95025300 0.25342500

H -5.69277800 -2.43646200 -1.47518300

C -3.22197100 1.89391400 1.48593100

O -2.83943900 1.09741800 0.63847000

O -2.79064200 1.90678600 2.71644500

C -4.24177600 2.95133200 1.13860200

N -4.73720600 2.79626000 -0.19142900

H -4.18437100 3.17457800 -0.94967600

C -5.54409100 1.71491800 -0.46006400

C -5.72899000 1.37338100 -1.91056700

H -6.75501400 1.01764700 -2.06720900

H -5.51623500 2.21155200 -2.58869300

H -5.04021500 0.54005300 -2.13504400

O -6.03559100 1.06947800 0.44529300

H -5.06455700 2.85760900 1.86400800

H -3.78526700 3.94221700 1.28557900

INT3$_{meta}$

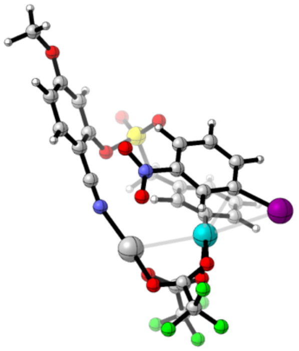

Charge: 0

Spin: 1

Pd -2.10636300 0.13015300 0.23249900

O -1.15593600 -0.59309600 3.19825800

C -2.25922900 -0.03691000 3.15877900

O -2.91664300 0.25024900 2.09798900

C -2.93983500 0.39392600 4.43676600

C -3.45030100 -2.58469000 0.38296900

O -3.42150900 -1.48013800 -0.23401200

O -2.55670300 -3.03377400 1.13211500

C -4.70245000 -3.40873900 0.20066300

Ag -0.60996100 -2.11756500 1.47217900

C -1.70104300 2.94170400 0.83321200

C -1.05469900 1.70034200 0.81155300

C 0.26773100 1.61082100 1.24153200

C 0.95636400 2.76178700 1.65215100

C 0.31792500 4.00415400 1.62256500

C -1.01417500 4.08999800 1.22363400

H -2.75049400 3.01671300 0.53313300

H 0.77629800 0.64427000 1.27236100

H 0.85676300 4.90315200 1.93415000

H -1.52209200 5.05791300 1.22128700

C 2.36163600 2.67925800 2.15620800

H 2.51409800 1.91148600 2.92962600

H 2.72792100 3.64106400 2.54213700

S 3.59338500 2.28107400 0.90246600

O 3.09908200 2.41099800 -0.45485900

O 4.86456500 2.86963500 1.27776400

C 4.38986100 -0.18578600 0.42398100

C 5.60828000 0.16356500 -0.13053200

C 3.82711500 -1.45636200 0.18041600

C 6.28247800 -0.75343400 -0.95310800

C 4.51536000 -2.36318900 -0.63642900

C 5.73326400 -2.02294200 -1.20283000

H 4.06402000 -3.33394600 -0.84456500

H 6.24324800 -2.74243300 -1.84187000

C 2.55849500 -1.77511100 0.72661400

N 1.51214700 -2.02871300 1.15458000

O 3.67437900 0.63847600 1.24247100

C 8.19991000 -1.17610100 -2.28189500

H 9.10046000 -0.61506100 -2.55911600

H 8.49950800 -2.09947800 -1.75749300

H 7.64617700 -1.44168300 -3.19846700

H 6.05868500 1.13656700 0.06462100

O 7.44626600 -0.32922700 -1.44915700

C 0.96845500 0.43567300 -1.93913500

C 0.09157300 -0.62173900 -1.83931800

C -1.31473200 -0.45591800 -1.82255700

C -1.81760100 0.85520200 -1.95888700

C -0.91645000 1.94714100 -2.08438700

C 0.44618700 1.73482800 -2.05185900

H 2.04393900 0.25963500 -1.93591600

H -1.95323900 -1.33305900 -1.93481100

H -1.30929100 2.95846600 -2.19656900

H 1.13528800 2.57891600 -2.09814800

I -3.84584400 1.19523200 -2.54033200

N 0.60818600 -1.99022200 -1.82768500

O 1.78930100 -2.14533700 -2.06272100

O -0.17415600 -2.88351200 -1.58979200

H -4.01904700 0.19869700 4.38224700

H -2.80469200 1.48091800 4.54813800

H -2.49494700 -0.11614200 5.29919700

H -5.05742100 -3.33785900 -0.83595300

H -5.48636500 -2.98722800 0.84876600

H -4.52862400 -4.45339800 0.48468300

TS2

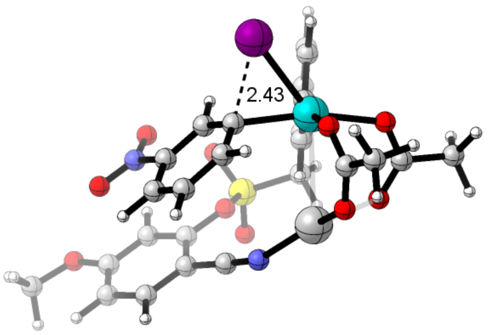

Charge: 0

Spin: 1

Pd -2.36002600 -0.18940000 0.33323900

O -1.79701200 -1.86682100 -2.33719000

C -2.76381400 -2.30833700 -1.69278700

O	-3.26945500	-1.77511100	-0.65383900
C	-3.40283900	-3.60980800	-2.11540800
C	-4.05269700	1.37848400	-1.70041900
O	-3.85607900	1.10774700	-0.48106400
O	-3.21851300	1.25289900	-2.62404900
C	-5.42896500	1.88948300	-2.05198100
Ag	-1.22631900	0.38221100	-2.38837200
C	-1.41711000	-2.26975300	2.25290400
C	-1.15460600	-1.59834800	1.05697800
C	-0.08299900	-2.00384300	0.26295400
C	0.72421600	-3.07832200	0.65440100
C	0.48056400	-3.72468200	1.86868300
C	-0.59020900	-3.32008300	2.65930600
H	-2.26885600	-1.98913500	2.87482300
H	0.12714000	-1.50827100	-0.68556200
H	1.12348700	-4.54802800	2.18951400
H	-0.79698400	-3.83367500	3.60150200
C	1.84049100	-3.52311200	-0.23599500
H	1.64465500	-3.35164400	-1.30397300
H	2.10636200	-4.58185600	-0.09295200
S	3.39844900	-2.69886200	0.07413300
O	3.72126500	-2.68878900	1.48383500
O	4.37972800	-3.10095400	-0.91299900
C	3.75239700	-0.17237900	-0.61570800
C	4.99307900	-0.07079000	-0.01695000
C	3.30535400	0.82048400	-1.51837400
C	5.81783600	1.02346600	-0.31674600
C	4.14442000	1.90558900	-1.80967100
C	5.39485500	2.00994900	-1.22647000
H	3.79715700	2.67571300	-2.50078700
H	6.02619900	2.86500100	-1.46266800
C	1.99319400	0.72745000	-2.04255300
N	0.90351000	0.65269900	-2.43204900
O	2.86557700	-1.16537800	-0.35617300
C	7.85465600	2.14208200	0.14719800
H	8.72300100	1.95179300	0.78932500
H	8.19531600	2.22823200	-0.89880100
H	7.37858600	3.08784700	0.45661900
H	5.32748400	-0.79993900	0.72033200
O	6.98678500	1.04943000	0.32133000
C	1.05213400	3.05193000	0.26482700
C	1.22078900	1.93339000	1.08018200
C	0.16964800	1.10441500	1.44480500
C	-1.11171800	1.40810200	0.95968200
C	-1.31201800	2.54954300	0.16258300
C	-0.22480800	3.34378400	-0.20084100
H	1.91599200	3.67220300	0.02678400
H	0.35767100	0.25683800	2.10307600
H	-2.32131700	2.80393400	-0.16014700
H	-0.38874600	4.21165000	-0.84300500
I	-2.79703100	1.05500800	2.67058900
N	2.56069400	1.64049700	1.61203500
O	3.43912400	2.44348800	1.37590700
O	2.70351400	0.62098000	2.24600800
H	-5.81430000	2.53646600	-1.25313300
H	-5.41889900	2.41406700	-3.01493000
H	-6.10220100	1.02147600	-2.12804600
H	-4.48368700	-3.59139100	-1.92546200
H	-3.19326300	-3.81636900	-3.17170000
H	-2.96913000	-4.41453800	-1.50132500

INT4

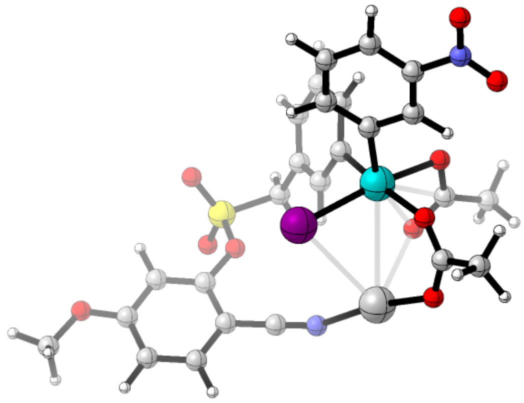

Charge: 0

Spin: 1

Pd -1.74596700 0.07310600 0.28946600

O -1.15189200 -0.91362000 2.31613500

C -2.23101400 -0.43568700 2.74159900

O -2.93821900 0.30085000 1.97935500

C -2.73033300 -0.73694700 4.11799500

C -2.87199700 -2.89024500 0.01375100

O -2.94949700 -1.64786500 -0.17283700

O -1.90128000 -3.52054100 0.50053300

C -4.06833300 -3.70025600 -0.42674500

Ag 0.04241900 -2.75871800 0.88959700

C -1.26513400 2.95259300 0.98631500

C -0.71542900 1.67407700 0.89594800

C 0.55540200 1.42372600 1.40959300

C 1.29336500 2.45785800 1.99663800

C 0.76218800 3.74970100 2.04349400

C -0.51480100 3.98856100 1.54737200

H -2.27854000 3.15283000 0.63738100

H 0.98252100 0.42086600 1.37025600

H 1.34220100 4.56437500 2.48451600

H -0.94540500 4.99105400 1.60777400

C 2.63950900 2.17315000 2.58284300

H 2.70857200 1.19677700 3.08350200

H 2.96774900 2.95030200 3.28999300

S 3.98474600 2.15436000 1.40095900

O 3.90043100 3.26046900 0.47279400

O 5.22662000 1.83962100 2.07771200

C 4.36608800 0.07780800 -0.18506100

C 5.40336400 0.67276100 -0.87996500

C 4.14533100 -1.31590400 -0.28260000

C 6.25218200 -0.11507600 -1.67314000

C 4.99207600 -2.08598300 -1.09317700

C 6.04359000 -1.50205300 -1.78017200

H 4.81419400 -3.15950600 -1.17816800

H 6.68948200 -2.12354500 -2.39865300

C 3.04633100 -1.89759300 0.39362300

N 2.12422600 -2.36865200 0.91666600

O 3.49397500 0.76157500 0.59634400

C 8.12321300 -0.15081900 -3.12966700

H 8.82038700 0.59710900 -3.52585800

H 8.69272600 -0.90896700 -2.56575900

H 7.60194700 -0.63720200 -3.97161100

H 5.57171000 1.74873400 -0.83537200

O 7.22616700 0.54537200 -2.29895600

C -4.69340100 2.55757500 -2.52356800

C -5.10785300 1.57535700 -1.63002000

C -4.21686100 0.84829600 -0.84503200

C -2.86324400 1.13214200 -0.97028000

C -2.40668800 2.10889300 -1.85417300

C -3.33119600 2.81827400 -2.62635600

H -5.43689000 3.09228700 -3.11423300

H -4.59296500 0.08434300 -0.16598800

H -1.34281800 2.33116100 -1.94040800

H -2.97324500 3.58312900 -3.31938000

I -0.05480400 -0.45838600 -1.65791700

N -6.54502300 1.28243300 -1.50766300

O -7.30652300 1.94306900 -2.17963700

O -6.87645100 0.40122600 -0.74644800

H -4.99127600 -3.11518100 -0.32624200

H -3.93675800 -3.94458600 -1.49250600

H -4.13052000 -4.63922600 0.13692800

H -3.43641700 0.03150700 4.45499800

H -1.88480700 -0.82736100 4.81175600

H -3.25160500 -1.70627400 4.08657600

INT5

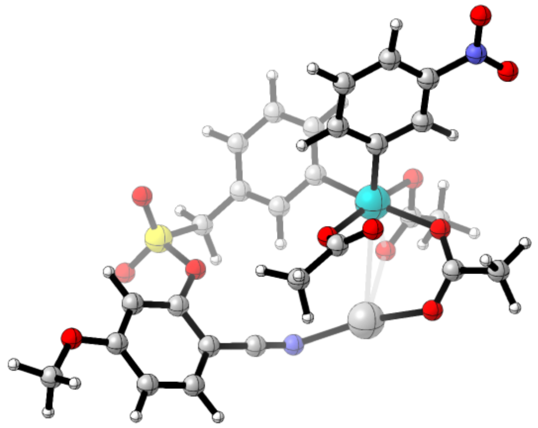

Charge: 0

Spin: 1

Pd -1.79199100 0.02429100 -0.40916100

O -1.45213900 0.96289100 -2.52242000

C -2.56461000 0.44868400 -2.78058400

O -3.14595800 -0.28026700 -1.90482200

C -3.26368000 0.68610800 -4.07963800

C -2.68491200 2.94347400 0.26002100

O -2.97477600 1.73666300 0.06039500

O -1.64119200 3.53215200 -0.12452000

C -3.68019100 3.74332400 1.05820700

Ag 0.04894200 2.65176000 -1.05722300

C -1.48500000 -2.84412100 -1.15951200

C -0.84269500 -1.61531600 -1.01054700

C 0.48362500 -1.45758400 -1.39345800

C 1.18829800 -2.54438400 -1.92714000

C 0.56071400 -3.78769100 -2.04191400

C -0.77149900 -3.93198600 -1.66414300

H -2.53569900 -2.95828500 -0.88713500

H 0.97960600 -0.49273000 -1.27916400

H 1.11209700 -4.64258900 -2.44196400

H -1.26680400 -4.90018400 -1.77021800

C 2.59942700 -2.37076800 -2.39149300

H 2.77922800 -1.42571800 -2.92398400

H 2.93885700 -3.19934200 -3.03187900

S 3.83277100 -2.37607200 -1.09190300

O 3.59809300 -3.42426400 -0.12530800

O 5.14535900 -2.16419600 -1.66880500

C 4.27155500 -0.15632700 0.27584300

C 5.28418200 -0.68107100 1.05747400

C 4.11086400 1.24355700 0.15490900

C 6.16861100 0.18249400 1.72311700

C 4.99130800 2.09346200 0.84014300

C 6.01831600 1.57727800 1.61318500

H 4.86008700 3.17399600 0.75876300

H 6.69187800 2.25865500 2.13092200

C 3.02724200 1.75273300 -0.60038600

N 2.11440200 2.16669400 -1.18571200

O 3.36486800 -0.91874300 -0.38196000

C 8.04555100 0.36464400 3.16149300

H 8.71172900 -0.34219800 3.67026100

H 8.64395500 1.00008300 2.48694700

H 7.54974100 0.99797600 3.91653800

H 5.40665700 -1.75802100 1.17396400

O 7.11474200 -0.41304400 2.44751400

C -4.07866100 -2.39870000 2.96878600

C -4.73121200 -1.60947100 2.02711100

C -4.05052200 -0.89873000 1.04077900

C -2.66954900 -1.00467400 1.02418200

C -1.97302000 -1.78075500 1.94398500

C -2.69031400 -2.47900200 2.91764000

H -4.66561900 -2.93007300 3.71748400

H -4.59952100 -0.28152200 0.32949900

H -0.88464200 -1.84319200 1.90289200

H -2.15349300 -3.09152200 3.64560700

N -6.19891600 -1.51627300 2.07027600

O -6.77210200 -2.13957200 2.93710200

O -6.74244400 -0.82563100 1.23754600

O -0.21750600 0.44497500 0.74015500

C -0.30713900 1.02602100 1.91871500

O -1.32886300 1.27538400 2.51400700

C 1.06548400 1.38642700 2.45217800

H 1.38255700 2.33236500 1.98521400

H 1.01052400 1.53421700 3.53752000

H 1.80654800 0.61640100 2.19857100

H -3.49355800 3.51115000 2.11853900

H -3.54903600 4.81975100 0.89601900

H -4.70435800 3.42665700 0.82298200

H -2.53229600 0.89721200 -4.86930400

H -3.91363600 1.56679400 -3.96012700

H -3.89391100 -0.17231700 -4.34333900

TS3

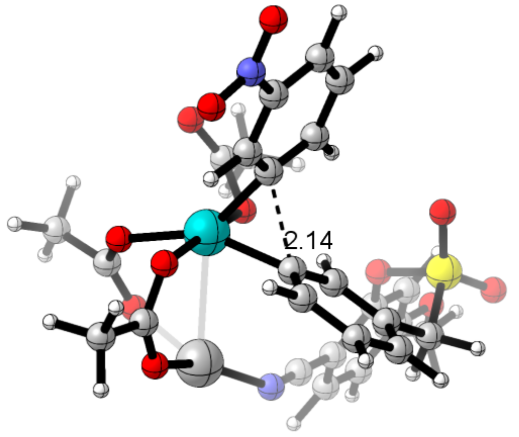

Charge: 0

Spin: 1

Pd 1.77429400 0.60530600 -0.50094400

O 2.14001000 2.79869700 1.68864600

C 3.21990900 2.32621000 1.30439200

O 3.37059700 1.33789200 0.50389500

C 4.50927900 2.93853100 1.78383100

C 0.78792100 3.08480900 -1.85177700

O 1.80226700 2.36282500 -1.55474300

O -0.20232800 3.25209600 -1.12639800

C 0.86873600 3.74909000 -3.20222700

Ag 0.04484700 2.44451400 1.04438000

C 2.07883900 -0.63731000 2.26521400

C 1.28172000 -0.53892800 1.12262200

C -0.05784000 -0.91927000 1.16108700

C -0.61371700 -1.41646400 2.34223300

C 0.18319600 -1.52712800 3.48433700

C 1.51729300 -1.12437100 3.44263600

H 3.13225000 -0.35977500 2.23144600

H -0.65372900 -0.82414900 0.25342900

H -0.23533800 -1.92943600 4.41061800

H 2.13967200 -1.21040100 4.33632200

C -2.05974700 -1.81384800 2.41140500

H -2.74159300 -0.96548100 2.58953000

H -2.24683800 -2.55665100 3.20171000

S -2.71702300 -2.62039100 0.94630600

O -1.70991800 -3.39862500 0.26966700

O -4.02321400 -3.15095900 1.27913400

C -4.13396400 -0.62766500 -0.08923800

C -5.27875000 -1.21455700 -0.59257100

C -4.14881000 0.71145900 0.36306100

C -6.47174000 -0.47754600 -0.66254200

C -5.34326600 1.44058000 0.27383300

C -6.49737600 0.85960000 -0.22971200

H -5.35758000 2.47992200 0.60671300

H -7.41003100 1.45163200 -0.28602100

C -2.97085100 1.30253600 0.88936800

N -2.01591300 1.77822600 1.34409200

O -2.94872300 -1.30408300 -0.07128500

C -8.76087300 -0.46694200 -1.27942800

H -9.45975200 -1.19522900 -1.70780300

H -9.14099300 -0.13998100 -0.29664300

H -8.69288800 0.40439000 -1.95280800

H -5.26429300 -2.24944700 -0.93144700

O -7.52375400 -1.12696800 -1.16143500

C 3.76025800 -3.65876200 -1.28325200

C 4.45373400 -2.58163800 -0.74302700

C 3.82420400 -1.40857100 -0.33911800

C 2.44519100 -1.32132800 -0.49921300

C 1.71321500 -2.38990700 -1.02072600

C 2.37881600 -3.54962200 -1.41440400

H 4.30677600 -4.55335700 -1.58070400

H 4.41644400 -0.59213900 0.07427400

H 0.62841100 -2.33041000 -1.10585700

H 1.80201500 -4.38137800 -1.82429600

N 5.91286600 -2.68010800 -0.57844100

O 6.45011900 -3.69260000 -0.96891000

O 6.48273000 -1.74680800 -0.05776100

O 0.11170700 0.06043600 -1.45049400

C 0.16068600 -0.09741700 -2.75150400

O 1.17390100 -0.07073800 -3.41636600

C -1.22030400 -0.31404000 -3.32971800

H 1.83421500 4.26307000 -3.30794300

H 0.03945100 4.45116500 -3.34645100

H 0.84092000 2.95912600 -3.96833300

H 4.83997200 3.66265900 1.02323500

H 5.29309800 2.17668500 1.88206000

H 4.35037600 3.46938700 2.72993800

H -1.14456500 -0.60146500 -4.38510500

H -1.79229600 0.62207300 -3.23960600

H -1.75699800 -1.08196500 -2.75416800

INT6

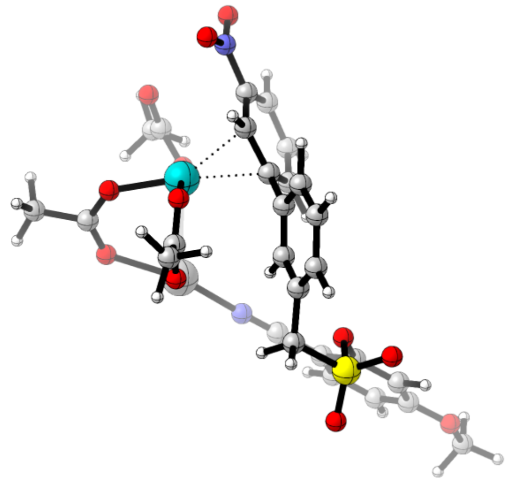

Charge: 0

Spin: 1

Pd 2.51956900 0.12380600 -0.17248300

O 0.62491300 1.18159700 -2.29927000

C 1.39084600 2.07115600 -1.90150200

O 2.25702200 1.95213500 -0.96454900

C 1.31259100 3.45769500 -2.49055100

C 3.29525700 -1.29664900 -2.67387300

O 3.56253400 -0.37660700 -1.83912600

O 2.21025300 -1.89159300 -2.79669000

C 4.44906900 -1.67242400 -3.56996500

Ag 0.42480700 -1.17401500 -1.76176100

C 0.47333900 3.28681200 1.55660100

C 0.25380300 1.97030200 1.13030300

C -0.80796300 1.71654000 0.26262900

C -1.61802400 2.74932700 -0.20766600

C -1.40448900 4.05418100 0.24428300

C -0.35939400 4.31674900 1.12684800

H 1.29312400 3.50556800 2.24391600

H -0.97693000 0.70314400 -0.09814100

H -2.04983200 4.86511000 -0.10233600

H -0.18659000 5.33596700 1.47899900

C -2.67947000 2.44435200 -1.21519600

H -2.39501900 1.63281600 -1.90037600

H -2.96529700 3.32366100 -1.81254000

S -4.24131400 1.94204300 -0.49805800

O -4.67358900 2.87925400 0.51364500

O -5.14114500 1.47932400 -1.53492000

C -4.45854700 -0.52640100 0.39801100

C -5.74335400 -0.47547100 0.90269500

C -3.89304000 -1.75321800 -0.01654700

C -6.49731700 -1.65595000 1.00301100

C -4.64846700 -2.92710700 0.10690600

C -5.94032900 -2.88651300 0.60852400

H -4.21379700 -3.87859500 -0.20462000

H -6.50998700 -3.81141400 0.68831800

C -2.58075900 -1.73907500 -0.55317000

N -1.50888700 -1.67182000 -0.98897600

O -3.66718100 0.57443900 0.30687800

C -8.56183400 -2.63873900 1.63318700

H -9.50887400 -2.27109400 2.04575200

H -8.75688200 -3.12011900 0.66000100

H -8.13105300 -3.37962600 2.32792000

H -6.17722900 0.47080800 1.22763000

O -7.72742300 -1.51373500 1.49454600

C 2.52409500 -0.93029200 3.30444600

C 3.14789700 0.17355700 2.77071000

C 2.48369000 1.06788100 1.90665400

C 1.10644200 0.86168000 1.63441200

C 0.48495000 -0.30702700 2.15796500

C 1.18322500 -1.17997600 2.96592700

H 3.09185000 -1.59234400 3.95845800

H 2.98564600 1.99837300 1.63444400

H -0.57550600 -0.47215500 1.95775400

H 0.68551000 -2.06511700 3.36668300

N 4.54704000 0.45579500 3.12069000

O 5.08651100 -0.29882900 3.89426800

O 5.04615500 1.44468000 2.63441900

O 2.67958200 -1.75646800 0.44419000

C 3.87437700 -2.17647800 0.76362800

O 4.83115000 -1.45211400 0.96367900

C 3.94996700 -3.68255000 0.86831200

H 2.32077100 3.85112200 -2.67662200

H 0.82817200 4.11720200 -1.75407500

H 0.72300900 3.44809300 -3.41495600

H 4.92634500 -0.76607000 -3.96600600

H 5.20175200 -2.19595500 -2.96137800

H 4.11115000 -2.32296900 -4.38472600

H 4.89657100 -3.97999300 1.33501300

H 3.88479900 -4.11265600 -0.14287300

H 3.09651800 -4.07146400 1.44163100re

TFA-Cat1

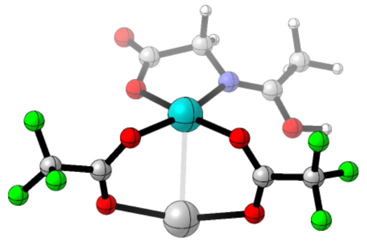

Charge: 0

Spin: 1

Pd -0.05927100 0.80558800 0.46608100

O 1.55161100 -1.82712900 -0.88420200

C 1.80825200 -1.50186500 0.28698500

O 1.23072700 -0.69029900 1.04323700

C 3.06895200 -2.16946000 0.88271100

C -2.60016600 -0.62761200 0.10700700

O -1.73974600 -0.25935400 0.94401600

O -2.46050200 -0.87990900 -1.10077700

C -4.00157500 -0.85098100 0.71967400

Ag -0.38321000 -1.20102600 -1.63634200

C -0.72078700 3.47945800 -0.04290500

O -1.25718900 2.29026400 -0.00170300

O -1.31858100 4.50030500 -0.26414200

C 0.78580000 3.51265300 0.24936300

N 1.36802700 2.18761700 0.09848400

C 2.59387000 2.03178600 -0.24440000

C 3.56632300 3.15791200 -0.40884300

H 3.47117400 3.88703900 0.40645400

H 4.59982400 2.78560700 -0.42587900

H 3.37738000 3.68486500 -1.35782900

O 3.00636800 0.80448400 -0.50843100

H 1.24145800 4.25872900 -0.41691400

H 0.90737600 3.86463100 1.28909100

H 3.96113500 0.75036200 -0.62986500

F -3.97623000 -1.96012100 1.46208900

F -4.92374400 -0.99832400 -0.21824200

F -4.34591800 0.16604700 1.49707500

F 3.16325600 -2.00076300 2.19054400

F 4.15612900 -1.61238700 0.31757500

F 3.09287200 -3.46685600 0.61677700

TFA-Cat2

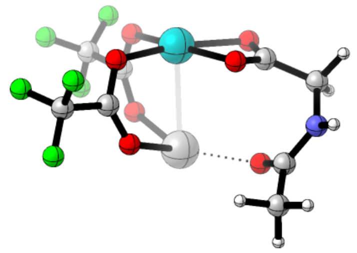

Charge: 0

Spin: 1

Pd -0.08565100 -0.28973500 -1.33291000

O 1.95672100 -0.21691200 -1.60771000

O 0.91364000 -2.12442400 -1.54673000

C 3.31513500 -2.16328100 -1.21307300

H 3.09522000 -3.12265700 -0.72290800

H 3.83601500 -2.37116500 -2.15854200

O 0.50387200 1.78761900 0.93894600

C -0.09529600 2.23901800 -0.03320300

O -0.53958600 1.63917800 -1.05859000

C -0.38281500 3.75886100 -0.08972500

C 1.99542900 -1.47987000 -1.49955400

C -2.34215400 -1.06408600 0.23583100

O -1.99087400 -0.77307200 -0.94282200

O -1.67646900 -1.09795800 1.27177700

C -3.84710900 -1.41327700 0.32422500

Ag 0.49147300 -0.50993100 1.48844300

C 3.67098600 -0.86469200 0.79246900

N 4.14283000 -1.32170000 -0.38543500

O 2.61395300 -1.30930300 1.24976700

C 4.45303200 0.20204900 1.49435300

H 5.46123400 0.35044000 1.08587400

H 3.88685300 1.14287100 1.40388700

H 4.51730700 -0.04508200 2.56228300

H 4.95638200 -0.87843500 -0.79039000

F -4.57690100 -0.35475700 -0.01768700

F -4.18534700 -1.77708500 1.55134900

F -4.13757600 -2.41165500 -0.50547600

F -1.68952900 3.97958000 -0.19319100

F 0.06090900 4.37089500 0.99836600

F 0.22117400 4.29758900 -1.14782100

TFA-INT1

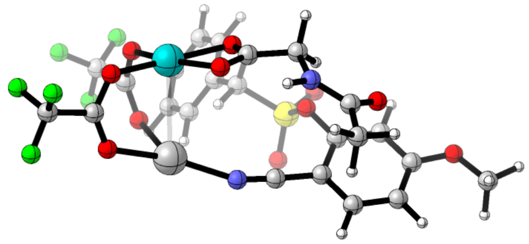

Charge: 0

Spin: 1

Pd 1.66703500 -1.04270900 -1.29969600

O 2.94294500 -2.12755100 1.50199800

C 3.53697300 -2.41306600 0.46293400

O 3.25013100 -2.13289200 -0.73375400

C 4.84703000 -3.22910300 0.57129600

C 2.81612300 1.25957600 -0.06305600

O 2.80256000 0.59563600 -1.14353400

O 2.19811800 1.05993600 0.97821200

C 3.76826000 2.47796000 -0.15138700

Ag 1.05995300 -0.94395800 1.61433000

C 1.38646900 6.12539500 -0.02924200

C 1.34672300 5.23101000 1.03910600

C 0.70998900 4.00127900 0.89692100

C 0.11504700 3.65772100 -0.32154700

C 0.14517200 4.56326800 -1.38674700

C 0.78407400 5.79113500 -1.24197800

H 1.89409200 7.08667300 0.08148400

H 1.82641600 5.48495700 1.98678400

H 0.69435100 3.29066800 1.72592900

H -0.33033500 4.29912600 -2.33456200

H 0.81498000 6.48977100 -2.08122900

C -0.57670400 2.33925600 -0.47209900

H -0.10296100 1.53905100 0.11223300

H -0.68081900 2.00697300 -1.51284800

S -2.24428600 2.45306700 0.15769600

O -2.27007100 2.40807200 1.60793700

O -3.01730700 3.43320000 -0.57262100

C -3.79628500 0.35236500 0.27684500

C -5.09205800 0.61878000 -0.10483100

C -3.51929600 -0.57155500 1.30820600

C -6.14970900 -0.07218200 0.50670700

C -4.58244900 -1.24360800 1.92422700

C -5.88834300 -1.00256300 1.52830200

H -4.37658800 -1.95992000 2.72176800

H -6.69953100 -1.54445200 2.01259400

C -2.16510700 -0.79093700 1.65170600

N -1.03894100 -0.94237900 1.88003900

O -2.74547600 0.92446800 -0.37976000

C -8.48517400 -0.46681300 0.56241900

H -9.35788300 -0.07934800 0.02315700

H -8.40722100 -1.55322700 0.38736900

H -8.61864100 -0.27810900 1.64141000

H -5.29309700 1.32792500 -0.90710300

O -7.36763200 0.21653900 0.05112900

C -0.62239000 -1.47687600 -1.96800500

O 0.13065300 -2.42088600 -1.58294500

O -0.12719300 -0.31238000 -2.01925000

C -2.04010500 -1.73774800 -2.37075200

N -2.52667000 -2.95579700 -1.79216200

H -1.86064900 -3.70790100 -1.66243300

C -3.79939200 -3.06692100 -1.32027300

C -4.11892300 -4.36565200 -0.62015800

H -4.16350400 -4.17146900 0.46248600

H -5.11646200 -4.69770500 -0.93609700

H -3.38720700 -5.16393900 -0.80580600

O -4.61588900 -2.17146900 -1.43082400

H -2.66937000 -0.89571800 -2.04884600

H -2.06875700 -1.74472300 -3.47773900

F 4.76891500 -4.32781500 -0.17517300

F 5.08788700 -3.58941500 1.82188200

F 5.86991500 -2.49696100 0.13831900

F 4.99718900 2.07376400 -0.46310600

F 3.82402400 3.12613500 1.00285000

F 3.35244900 3.32256700 -1.09128200

TFA-TS1$_{meta}$

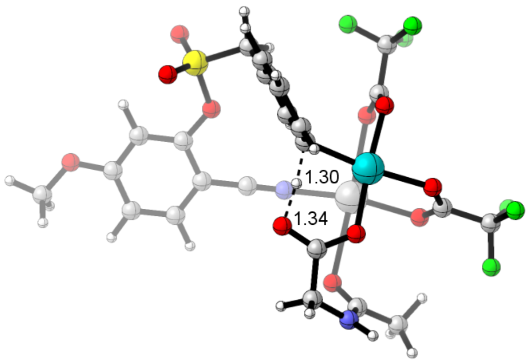

Charge: 0

Spin: 1

Pd 1.76814700 0.57713300 1.17460100

O 0.94936400 1.62233500 -1.63132600

C 1.24362300 2.50679400 -0.83809000

O 1.64445300 2.41665600 0.36186900

C 1.02051800 3.96728700 -1.29520900

C 4.08548800 -0.24111500 -0.42488700

O 3.72893000 0.46307700 0.54564500

O 3.40831300 -0.89893000 -1.22560000

C 5.61365200 -0.32409900 -0.64848300

Ag 1.19198800 -0.92327400 -1.32939800

C -0.24286100 1.56341500 3.14024700

C -0.19523700 0.95572000 1.86935200

C -1.08240900 1.41432700 0.87808300

C -1.96795600 2.45966600 1.12438800

C -1.99205200 3.04451000 2.39658200

C -1.13407700 2.59979100 3.40096500

H 0.42262300 1.20895300 3.93259000

H -0.11414300 -0.34007700 1.92399700

H -1.05933000 0.95866100 -0.11339300

H -2.69224600 3.85851100 2.60335800

H -1.16387100 3.06771600 4.38745000

C -2.88915300 2.93329300 0.04552300

H -2.44342400 2.89897700 -0.95858800

H -3.27038400 3.95089200 0.21872900

S -4.38908100 1.96142100 -0.05567500

O -4.87656200 1.61311200 1.26237200

O -5.27662600 2.50608300 -1.05963800

C -4.26677200 -0.61613700 -0.71111600

C -5.61915700 -0.81160200 -0.49271500

C -3.41906000 -1.71822800 -0.97234100

C -6.14748400 -2.11203000 -0.51256600

C -3.96510500 -3.00850500 -0.99486500

C -5.31567500 -3.21643200 -0.76337800

H -3.30770900 -3.85609300 -1.19686100

H -5.71276500 -4.23026200 -0.78284600

C -2.03979600 -1.50658700 -1.22576500

N -0.91548500 -1.34105100 -1.44677700

O -3.66256100 0.59951900 -0.71902800

C -8.08754900 -3.45604200 -0.28845900

H -9.14886800 -3.27212700 -0.08253800

H -7.99349500 -3.95528800 -1.26796100

H -7.67886000 -4.11482900 0.49663900

H -6.29563300 0.02057700 -0.30207500

O -7.45977800 -2.19775600 -0.28636500

C 1.01880800 -2.04997700 2.08002600

O -0.16849400 -1.66725400 2.12858000

O 2.01592900 -1.30033600 1.88791400

C 1.31267600 -3.53108000 2.28195900

N 2.45287300 -3.97992400 1.53799300

H 3.33234800 -4.12523300 2.01424100

C 2.44209100 -3.86198700 0.18302000

C 3.71747700 -4.18716100 -0.53795500

H 3.47385300 -4.74514400 -1.45123500

H 4.18166200 -3.23734100 -0.84314900

H 4.43113200 -4.76007800 0.06970700

O 1.43104400 -3.48490400 -0.39585000

H 1.48033900 -3.70384700 3.35524400

H 0.40789200 -4.08030500 1.98618700

F -0.28535600 4.26259500 -1.13948400

F 1.31712100 4.11978500 -2.57804600

F 1.71824600 4.84816600 -0.59448300

F 5.92017500 0.03450700 -1.89109900

F 6.30011600 0.43576700 0.18915400

F 6.01239100 -1.59299200 -0.47511000

TFA-TS1$_{ortho}$

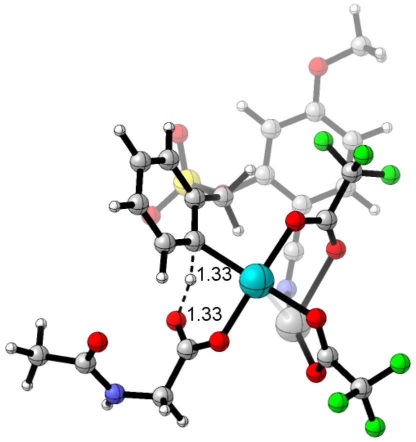

Charge: 0

Spin: 1

Pd -1.41549900 -0.31224400 0.99005400

O 1.01011800 -1.99304900 -0.13395700

C 1.14201700 -1.60964800 1.02133000

O 0.37910000 -0.88870300 1.72889100

C 2.46678500 -1.94758200 1.74937600

C -2.61044600 -2.79578400 0.00251500

O -2.09916600 -2.26200500 1.01327200

O -2.50059400 -2.48147300 -1.19237600

C -3.50278100 -4.01273400 0.33785800

Ag -0.77721700 -1.31232900 -1.81648500

C -1.35410100 3.22211600 3.16038700

C -1.78606100 2.28955800 2.22324400

C -0.88266300 1.70097000 1.31401000

C 0.47070400 2.11004000 1.34423100

C 0.89866500 3.04443800 2.28694500

C -0.00892200 3.58574800 3.19768500

H -2.06810300 3.66834800 3.85592500

H -2.84758600 2.03529100 2.16447700

H -1.40502100 1.63038700 0.08918500

H 1.94329300 3.36396000 2.29995600

H 0.34117700 4.31275400 3.93476100

C 1.42894800 1.55358400 0.33524100

H 0.91542300 0.91388000 -0.39481700

H 2.25827300 0.99693200 0.79458000

S 2.19469400 2.86669500 -0.62169700

O 1.21278700 3.65237200 -1.32303000

O 3.25573600 3.47875300 0.15673300

C 3.74630100 0.93548400 -1.52228100

C 4.93106100 1.11915100 -0.83697400

C 3.33438200 -0.35584800 -1.92159400

C 5.72255800 0.00175100 -0.51792900

C 4.14068000 -1.45908700 -1.61105500

C 5.32632100 -1.28754100 -0.91572600

H 3.80745700 -2.45965400 -1.88915800

H 5.92345000 -2.16213100 -0.66232100

C 2.05532100 -0.50902300 -2.50498600

N 0.96715100 -0.63052600 -2.89507200

O 2.91075600 1.96604300 -1.83980300

C 7.65875400 -0.79645700 0.59176700

H 8.48176800 -0.34052100 1.15486200

H 7.11420100 -1.49421100 1.25005600

H 8.07482500 -1.35151900 -0.26604700

H 5.23528000 2.11422200 -0.51417200

O 6.83225800 0.26280600 0.17135000

C -3.23995500 1.13160600 -0.59767200

O -2.22842300 1.78696100 -0.94553000

O -3.22791400 0.21440900 0.26951300

C -4.57664300 1.43524600 -1.24978100

N -4.70077800 2.81864800 -1.60202100

H -4.56855600 3.09713800 -2.56463800
C -4.55085400 3.74555600 -0.61029200
C -4.47618800 5.18355400 -1.04976400
H -4.92572600 5.81390700 -0.27262100
H -3.41411200 5.46196600 -1.13784700
H -4.97037100 5.36896700 -2.01372700
O -4.45908400 3.40217100 0.55427100
H -5.36172800 1.12908600 -0.54408500
H -4.66787900 0.80727600 -2.14859500
F 2.28155200 -2.26808800 3.01968500
F 3.27321900 -0.86716500 1.70921100
F 3.11240000 -2.94241100 1.15243800
F -3.94971800 -4.61135700 -0.75683400
F -4.55262700 -3.61528600 1.05520000
F -2.81944000 -4.90282600 1.05246600

TFA-TS1$_{para}$

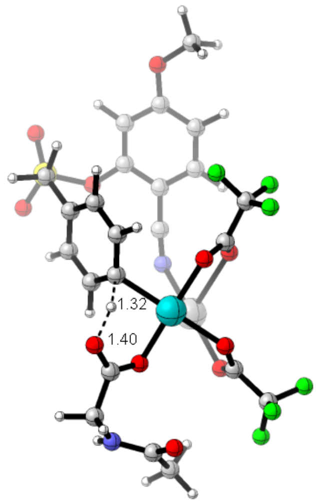

Charge: 0

Spin: 1

Pd -1.50622500 -0.03559000 1.05565400
O 0.12263400 -2.14342400 -0.62655100
C 0.18679900 -2.28959700 0.58611700
O -0.35523200 -1.61923000 1.51458000
C 1.08827400 -3.40725100 1.16752500
C -3.61898300 -1.17089300 -0.60467200
O -3.04575800 -1.28706800 0.50061200
O -3.22225000 -0.59873800 -1.63536100
C -4.97022700 -1.91217000 -0.72099000
Ag -1.10976700 -0.14266700 -1.83888300
C 0.07529000 1.25808100 1.57957900
C 0.37048200 2.27455300 0.64849600
C 1.66680600 2.73746300 0.46274500
C 2.71606200 2.17047600 1.19657100
C 2.44179100 1.14923700 2.11708300
C 1.13970200 0.70929000 2.31923700
H -1.03461000 1.58195100 2.21653800
H -0.44288600 2.72074600 0.06990600
H 1.86999200 3.52817900 -0.26222500
H 3.25933300 0.69556300 2.68392600
H 0.94404000 -0.09013900 3.03647700
C 4.13294600 2.64921000 1.06332900
H 4.85233700 1.93517100 1.48613700
H 4.29580100 3.62501500 1.55262300
S 4.71372000 2.98948300 -0.60561900
O 4.02615800 4.13079100 -1.15846500
O 6.15880200 2.90361300 -0.60585300
C 4.28853600 0.41454500 -1.14256100
C 5.45956700 -0.07856500 -0.59692900
C 3.20241000 -0.44652500 -1.40518200
C 5.55280300 -1.44557200 -0.27496300
C 3.31942400 -1.80986800 -1.10089500
C 4.47768900 -2.31166900 -0.52917700
H 2.46583300 -2.46415300 -1.28474400

H 4.52288600 -3.36913900 -0.27337300

C 1.97391700 0.04444000 -1.91265900

N 0.92083800 0.35270700 -2.28953000

O 4.08981400 1.72390900 -1.47718600

C 6.90049100 -3.17414000 0.62525700

H 7.91124800 -3.24217500 1.04486600

H 6.17077200 -3.49795300 1.38644300

H 6.82945800 -3.83810500 -0.25259400

H 6.31864500 0.56926600 -0.42382300

O 6.70969300 -1.82488300 0.26867000

C -2.87790900 2.31473500 1.61567100

O -2.71452500 1.51451100 0.64749700

O -2.12766000 2.36653700 2.61331800

C -4.08221300 3.23706700 1.52961600

N -4.77463800 3.17508400 0.28014000

H -4.44565100 3.76187600 -0.47466000

C -5.48360600 2.04453300 -0.04387300

C -5.91133300 1.93605900 -1.48364700

H -6.89783200 1.45831700 -1.52472300

H -5.93944200 2.90271900 -2.00611600

H -5.20172600 1.26574300 -1.99387200

O -5.72086100 1.18696900 0.78241600

H -4.75546800 2.91328300 2.33935600

H -3.76203800 4.26304000 1.75959900

F -5.81717800 -1.24139400 -1.49509000

F -4.74537800 -3.10101300 -1.29333400

F -5.53633300 -2.11694400 0.45271700

F 1.60947000 -4.16878900 0.20550000

F 0.42190200 -4.19244400 2.00141500

F 2.11006700 -2.85580200 1.83766400

TFA-INT2$_{meta}$

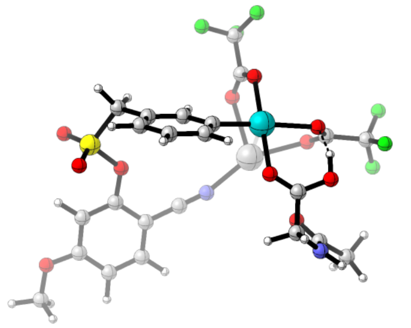

Charge: 0

Spin: 1

Pd -1.72157600 -0.80118400 1.15189700

O -0.89387600 -2.00446100 -1.71579900

C -1.47266200 -2.77659000 -0.95496500

O -1.93255800 -2.57232800 0.20328600

C -1.60221100 -4.24924700 -1.40609400

C -3.89450800 0.39581600 -0.70634900

O -3.67069000 -0.05400900 0.45689100

O -3.13771200 0.44954700 -1.66915700

C -5.31923900 0.95769100 -0.92929600

Ag -0.89032900 0.37855600 -1.38636900

C 0.26737100 -1.24770400 3.21517100

C 0.01416400 -1.37452400 1.84607300

C 1.00923900 -1.89093400 1.01288400

C 2.24170700 -2.28861600 1.54346600

C 2.48523700 -2.16784900 2.91519200

C 1.49650700 -1.64714300 3.74385500

H -0.49382600 -0.83253500 3.88054900

H -3.78245600 1.00597800 1.59489700

H 0.83731400 -1.99146600 -0.06132800

H 3.45175400 -2.46606200 3.32712300

H 1.68232400 -1.55078100 4.81673500

C 3.29028600 -2.84505800 0.63232900
H 2.92495300 -3.05806500 -0.38221000
H 3.77085900 -3.75560800 1.02585800
S 4.70261900 -1.77383800 0.39335300
O 5.19264400 -1.23869000 1.64720800
O 5.61846700 -2.36497700 -0.56034300
C 4.46704000 0.65326500 -0.64281800
C 5.82541000 0.89701800 -0.52537700
C 3.59420800 1.68531800 -1.06202200
C 6.33334700 2.17381800 -0.80950900
C 4.12066200 2.95190600 -1.34915600
C 5.47669500 3.20824200 -1.22296400
H 3.44404300 3.74328200 -1.67682300
H 5.85846900 4.20251500 -1.45004900
C 2.20882500 1.41985400 -1.20896200
N 1.07829000 1.21079700 -1.34539600
O 3.87909000 -0.54337800 -0.39963700
C 8.26309300 3.54568600 -0.93100000
H 9.33501700 3.40932300 -0.74366100
H 8.11883500 3.85076700 -1.98160400
H 7.88133800 4.33933500 -0.26614600
H 6.52343300 0.11904100 -0.21921800
O 7.65332100 2.30780400 -0.66277200
C -2.25584600 1.94869800 2.22953600
O -3.53992100 1.85584100 2.08734000
O -1.45479000 1.02003600 2.14500000
C -1.77035400 3.35028100 2.54344900
N -2.42206600 4.31544300 1.69877200
H -3.02570100 5.01471100 2.10784800
C -2.46557100 4.07528200 0.35852100
C -3.29474700 5.00479900 -0.48136200
H -2.70368800 5.31202400 -1.35446300
H -4.15540900 4.43093800 -0.85740400
H -3.65218500 5.89350200 0.05594400
O -1.87414800 3.11641900 -0.11213200
H -1.99041600 3.57947100 3.59649300
H -0.67930200 3.34984200 2.40589600
F -0.52246600 -4.91935600 -0.98215300
F -1.65172700 -4.34723300 -2.72856000
F -2.67830000 -4.84191600 -0.90446500
F -6.03306200 0.12970300 -1.68292700
F -5.96488400 1.14087200 0.22117300
F -5.25771800 2.13897400 -1.54932600

TFA-INT2$_{ortho}$

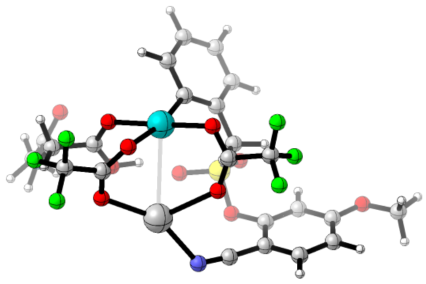

Charge: 0

Spin: 1

Pd -1.11979100 -0.30531000 1.42074700
O 1.19755400 -1.91916900 -0.00506000
C 1.29976100 -1.85187800 1.22195900
O 0.55105900 -1.28455900 2.05752200
C 2.60894900 -2.41355800 1.81885800
C -2.90313800 -2.22677300 -0.06029100
O -2.21322600 -2.13823300 0.96657300
O -2.67653000 -1.75333800 -1.19715600
C -4.24318100 -2.98753800 0.04863500

Ag -0.55118100 -1.43069300 -1.61681700

C -0.73299500 3.16812500 3.72588100

C -1.18739100 2.02553000 3.06868900

C -0.38457800 1.37238500 2.12619900

C 0.88300800 1.90083600 1.83624000

C 1.33215800 3.05202200 2.50046000

C 0.53379700 3.68070300 3.44788700

H -1.37651400 3.66027600 4.45951700

H -2.18508400 1.63978700 3.29404200

H -1.37893000 1.72678700 -0.80472700

H 2.31377300 3.46404900 2.25091700

H 0.89453800 4.57504200 3.96055700

C 1.73773800 1.31056300 0.75796100

H 1.37317700 0.33967600 0.40230900

H 2.80557900 1.24551400 1.00797700

S 1.60345100 2.36356600 -0.68367100

O 0.21673700 2.43000200 -1.11698100

O 2.38753400 3.57101500 -0.54370600

C 3.47104700 0.78001600 -1.70724800

C 4.59790700 1.37051200 -1.16813100

C 3.48048800 -0.57272200 -2.11465400

C 5.76007200 0.59816600 -0.99487100

C 4.64710400 -1.32544700 -1.94335100

C 5.77998900 -0.75264600 -1.38424400

H 4.65349100 -2.37591300 -2.23823500

H 6.67237600 -1.36194800 -1.24851700

C 2.26433000 -1.09795300 -2.61556400

N 1.21063100 -1.43654300 -2.96868200

O 2.28681700 1.43403500 -1.88531500

C 8.00038000 0.54653300 -0.21866700

H 8.68804300 1.27026600 0.23479900

H 7.85468000 -0.29647800 0.47768400

H 8.44079100 0.17272300 -1.15852300

H 4.59315900 2.41925000 -0.86915900

O 6.79592100 1.23722700 -0.45127900

C -3.14542900 1.12626000 -0.26158100

O -2.31108200 1.62368100 -1.11559300

O -2.86068200 0.62021100 0.82432300

C -4.58602200 1.11194900 -0.71067500

N -4.91267100 2.11690200 -1.67198700

H -5.03695400 1.85920300 -2.64099500

C -4.75440200 3.42741600 -1.31809500

C -4.97858700 4.44198100 -2.40914100

H -5.39967700 5.35080200 -1.96164200

H -4.00292100 4.70498900 -2.84694700

H -5.63830700 4.08003600 -3.21027000

O -4.42527900 3.72674100 -0.18699700

H -5.19557500 1.20344200 0.20021000

H -4.76271000 0.10755800 -1.12522600

F 2.52219000 -2.68901300 3.10895400

F 3.56453300 -1.47883800 1.65505900

F 3.00463000 -3.50736300 1.17791700

F -4.30467300 -3.96436700 -0.85300000

F -5.25136800 -2.13594000 -0.19992800

F -4.43693400 -3.50954300 1.24738800

TFA-INT2$_{para}$

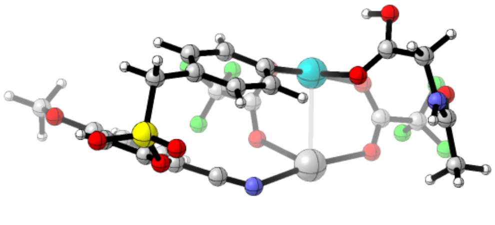

Charge: 0

Spin: 1

Pd 1.35692300 0.13158900 1.14564100

O -0.04862500 2.15155800 -0.87587600

C -0.09262200 2.46843700 0.30592500

O 0.41729100 1.90704800 1.31824400

C -0.92043800 3.71349800 0.71184900

C 3.75042200 0.92387800 -0.42383300

O 3.24623500 1.04910800 0.71053300

O 3.25196200 0.42791600 -1.45595600

C 5.15009200 1.55274200 -0.60225100

Ag 1.12603300 0.06112200 -1.73659100

C -0.36646400 -0.78647300 1.41322000

C -0.52773600 -2.07718700 0.88647500

C -1.77245000 -2.70061300 0.88196400

C -2.89152500 -2.04217000 1.40165100

C -2.72829100 -0.77073900 1.95975000

C -1.47975700 -0.15117600 1.97709200

H 3.07470600 -0.52380900 2.90846700

H 0.32128700 -2.59894800 0.43999400

H -1.88591000 -3.68729100 0.42563700

H -3.59526800 -0.24076400 2.36588800

H -1.38899900 0.85434700 2.39124200

C -4.25969900 -2.64192300 1.31196800

H -5.04079600 -1.96958100 1.69006800

H -4.35709900 -3.61476300 1.82206800

S -4.76324600 -3.08246900 -0.36830000

O -4.05625500 -4.26295900 -0.80450600

O -6.20602900 -2.98705400 -0.46890300

C -4.25690900 -0.55567200 -1.06791800

C -5.41550300 -0.01116900 -0.54233200

C -3.16822000 0.27708100 -1.40491100

C -5.49030400 1.37309000 -0.30540400

C -3.26863700 1.65822900 -1.18620700

C -4.41109300 2.21016200 -0.62848200

H -2.41394700 2.29135900 -1.43026000

H -4.44103700 3.28256600 -0.44168800

C -1.94050000 -0.24009600 -1.89026200

N -0.88006300 -0.53817800 -2.25658700

O -4.07706200 -1.88572000 -1.30156700

C -6.80627500 3.16996600 0.50158100

H -7.80983600 3.27823200 0.93030100

H -6.06173700 3.53403800 1.22979400

H -6.74040600 3.77620700 -0.41768500

H -6.27940200 -0.63610500 -0.31695400

O -6.63664300 1.79936600 0.23010600

C 3.27781600 -1.99588800 1.77869300

O 2.36972100 -1.67022400 1.01828600

O 3.58805500 -1.35595100 2.86779500

C 4.06807300 -3.25796100 1.51257700

N 4.37507000 -3.36201200 0.11547800

H 3.99286900 -4.11244300 -0.44228800

C 4.97465300 -2.27923000 -0.45977400

C 5.19731800 -2.29615200 -1.94060500

H 6.24247300 -2.02546000 -2.14109400

H 4.96092300 -3.25769600 -2.41528700

H 4.57224800 -1.49593100 -2.36635400

O 5.27366800 -1.33087800 0.24761600

H 4.97621900 -3.22921700 2.13306500

H 3.45553800 -4.11661500 1.82577400

F 5.85680900 0.93214400 -1.54159300

F 4.98469000 2.82416700 -0.99393200

F 5.84861700 1.55942500 0.52084000

F -1.47909100 4.29731000 -0.34777000

F -0.17311500 4.61391600 1.33657800

F -1.91426700 3.35141400 1.53469700

TFA-INT3$_{meta}$

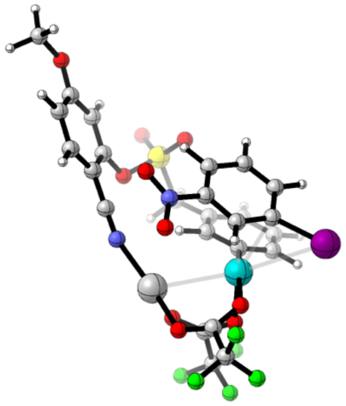

Charge: 0

Spin: 1

Pd 1.65597500 0.25483000 0.41548400

O 1.04399500 1.55248800 -2.50533400

C 2.04450200 1.96684900 -1.92998400

O 2.58481600 1.55351100 -0.86330900

C 2.78919600 3.18748600 -2.51884700

C 3.21816800 -1.72275700 -1.25561000

O 3.07515000 -1.26873600 -0.09809600

O 2.48234000 -1.60569800 -2.24496700

C 4.50362100 -2.56464900 -1.42699400

Ag 0.46436200 -0.74576100 -2.18745200

C 1.04291200 2.80186300 1.63830400

C 0.50660800 1.80379800 0.81891000

C -0.76357100 1.96146200 0.27206100

C -1.51999800 3.10346100 0.57705700

C -0.99926100 4.07323400 1.43662400

C 0.28500800 3.92674200 1.95666800

H 2.05670200 2.70155200 2.03670400

H -1.17855500 1.20845200 -0.40244800

H -1.59028700 4.96005900 1.68044400

H 0.70192600 4.69855900 2.60830100

C -2.86749300 3.32149400 -0.03393900

H -2.88227200 3.21559800 -1.12915300

H -3.29329300 4.30053900 0.22694400

S -4.14608200 2.18445500 0.53257400

O -3.75724000 1.41858900 1.70160800

O -5.43352500 2.84866100 0.48649200

C -4.76164700 -0.02408900 -0.76822300

C -6.05038500 -0.13157400 -0.27838800

C -4.10311700 -1.14739700 -1.31194100

C -6.70234700 -1.37552500 -0.31395200

C -4.76868500 -2.38024800 -1.34744400

C -6.05748900 -2.50206000 -0.85451000

H -4.24772500 -3.25167100 -1.74598000

H -6.55032600 -3.47275300 -0.88556700

C -2.76836100 -1.01686300 -1.76770100

N -1.66959600 -0.91080600 -2.11920300

O -4.05999000 1.14628600 -0.78482900

C -8.68012600 -2.58358100 0.18852400

H -9.65038100 -2.34321400 0.63928100

H -8.84324300 -2.96265600 -0.83456000

H -8.18680000 -3.36384100 0.79246400

H -6.57325300 0.73349200 0.12906400

O -7.93852500 -1.38730200 0.18433400

F 2.63254800 4.23616300 -1.70506100

F 2.31763500 3.51874100 -3.71235800

F 4.09116100 2.94422000 -2.63590800

F 4.65401600 -3.00280100 -2.66794900

F 5.57641500 -1.84442200 -1.10513400

F 4.45599000 -3.62339800 -0.61405000

C -1.59396700 -0.92649200 1.92703700

C -0.62124500 -1.65992400 1.28544500

C 0.76086900 -1.50149300 1.54952500

C 1.13395100 -0.56790200 2.53961600

C 0.13219600 0.18157100 3.21435500

C -1.19928000 0.01101100 2.89637000

H -2.64581400 -1.07434900 1.68364100

H 1.46042500 -2.24621300 1.16707200

H 0.42356900 0.89923900 3.98239300

H -1.96290200 0.61819900 3.38408400

I 3.08039600 -0.62042600 3.41170600

N -1.00813200 -2.70151800 0.33409700

O -2.18103400 -3.00642400 0.28318700

O -0.13191400 -3.19360900 -0.34335400

TFA-TS2

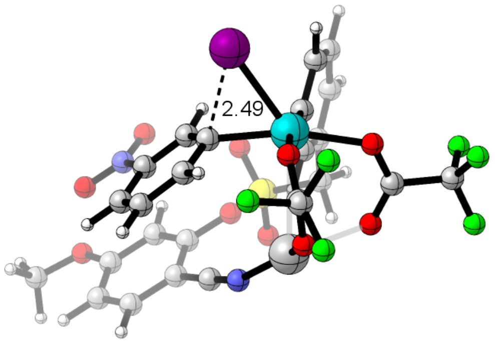

Charge: 0

Spin: 1

Pd -1.82143900 -0.01504900 0.76468700

O -1.60504200 -2.00984500 -1.79570900

C -2.46239300 -2.32337200 -0.96687200

O -2.85794400 -1.68743600 0.04348800

C -3.12795600 -3.71151300 -1.11356400

C -3.63338000 1.41230900 -1.26512900

O -3.36982900 1.23732400 -0.05585600

O -2.98398600 1.08588300 -2.26905200

C -4.91127300 2.24748400 -1.50622600

Ag -0.96895200 0.20132100 -2.19294700

C -0.76392900 -1.99377900 2.72263300

C -0.57174400 -1.38121200 1.48473000

C 0.41834900 -1.84084100 0.62096900

C 1.21992900 -2.92928600 0.98669100

C 1.05201300 -3.52195200 2.23983800

C 0.06213900 -3.05503100 3.09891100

H -1.55795900 -1.66508600 3.39464100

H 0.57484200 -1.37963000 -0.35515600

H 1.68931500 -4.35772200 2.53844500

H -0.08506800 -3.53066800 4.07142000

C 2.24448000 -3.44756200 0.02821700

H 1.94978700 -3.34908400 -1.02622900

H 2.51197900 -4.49810800 0.21926600

S 3.83060800 -2.62467600 0.13602900

O 4.27099800 -2.50381200 1.50753800

O 4.71789200 -3.11763000 -0.89676700

C 4.13992200 -0.17710800 -0.81675700

C 5.43449400 -0.03986100 -0.35677100

C 3.61452500 0.72824200 -1.76858200

C 6.23531100 1.00285200 -0.84738700

C 4.42916700 1.76322600 -2.25116900

C 5.73186400 1.90117400 -1.80725500

H 4.02203900 2.46596000 -2.98033400

H 6.34470600 2.71541600 -2.19052700

C 2.25811800 0.59889300 -2.14882000

N 1.13531700 0.48757700 -2.41622600

O 3.27335500 -1.12034000 -0.37003500

C 8.32129200 2.11969600 -0.70730100

H 9.24744600 1.97548200 -0.13833300

H 8.55471400 2.08965900 -1.78515100

H 7.89069700 3.10240800 -0.45161100

H 5.83459600 -0.69767700 0.41422800

O 7.46114700 1.07026000 -0.33467700

F -2.55823300 -4.55453600 -0.24580400

F -2.96641300 -4.20495900 -2.33371400

F -4.42831400 -3.66106000 -0.85121500

F -5.33267100 2.16797200 -2.75892300

F -5.89952500 1.87352800 -0.70426000

F -4.62449000 3.53139100 -1.24357700

C 1.56294000 3.19928100 0.05194800

C 1.82290300 2.13067300 0.90781600

C 0.82077100 1.32002100 1.42291700

C -0.50252300 1.58738800 1.04558300

C -0.79628500 2.69032400 0.22472600

C 0.24158900 3.46635100 -0.29016300

H 2.39244300 3.80794400 -0.30771400

H 1.08328600 0.51416800 2.10754700

H -1.83380400 2.94186100 0.00494900

H 0.00449500 4.30411100 -0.94930300

I -2.01634700 1.38368400 3.01747000

N 3.21229800 1.87315400 1.32039600

O 4.06123000 2.63997800 0.91779600

O 3.41864400 0.91514300 2.02811400

TFA-INT4

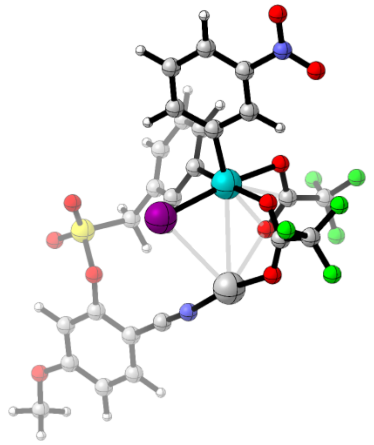

Charge: 0

Spin: 1

Pd -1.50797000 0.32473700 0.12115900

O -0.58824700 -1.13039100 1.86253700

C -1.53021400 -0.64947900 2.50661500

O -2.33620200 0.20527400 2.04633700

C -1.72797300 -1.04379300 3.98139700

C -2.67160600 -2.47969400 -0.67094700

O -2.85335100 -1.26857100 -0.43577900

O -1.62812800 -3.15255600 -0.63687600

C -3.95616500 -3.22655000 -1.10439500

Ag 0.34252300 -2.39952800 -0.27716500

C -0.80578500 2.79526000 1.57797600

C -0.28860300 1.72073100 0.85269800

C 1.08394800 1.50288400 0.79205600

C 1.95735100 2.38652600 1.43848500

C 1.44499700 3.48203600 2.13637800

C 0.06871800 3.67599100 2.21367800

H -1.88308300 2.94395500 1.66393800

H 1.49288100 0.65596800 0.23928600

H 2.12449600 4.18075500 2.63142900
H -0.33426900 4.51902800 2.77978600
C 3.43467300 2.17405300 1.36229400
H 3.75300600 1.15546200 1.63458300
H 3.99445000 2.88724300 1.98298500
S 4.11578700 2.46703100 -0.29492800
O 3.15535700 3.07297800 -1.17737300
O 5.46198800 2.98243800 -0.13805000
C 5.23636300 0.04343900 -0.54781200
C 6.57417400 0.38440400 -0.64711300
C 4.86755600 -1.26340200 -0.14322800
C 7.56544500 -0.55485400 -0.31797900
C 5.86954900 -2.19495900 0.16824000
C 7.20918400 -1.85122100 0.09398800
H 5.58206300 -3.20365000 0.47062600
H 7.96621300 -2.59302100 0.34481300
C 3.50357400 -1.64505600 -0.10221800
N 2.39895000 -1.98994800 -0.07097500
O 4.22468000 0.88850400 -0.88580000
C 9.88988300 -0.99535900 -0.14835600
H 10.80834500 -0.42215400 -0.32157700
H 9.86723700 -1.32813500 0.90315700
H 9.88615600 -1.87705400 -0.81132800
H 6.87173700 1.37952500 -0.97127300
O 8.82073200 -0.12651400 -0.43831100
F -1.22422700 -0.08266900 4.75791600
F -1.09970100 -2.17732300 4.25879000
F -3.01343500 -1.18780500 4.27008400
F -3.81628000 -4.54193000 -1.01868200
F -4.98901500 -2.86463100 -0.35083700
F -4.23328300 -2.91597200 -2.37199100
C -4.72245300 3.52133800 -1.32633100
C -5.04858500 2.31582200 -0.71562000
C -4.09061200 1.37477300 -0.34403700
C -2.76419300 1.69061100 -0.59737500
C -2.38676300 2.88402500 -1.20917300
C -3.38088300 3.79717600 -1.56985300
H -5.51891400 4.21395500 -1.59766000
H -4.40331800 0.43866900 0.11944600
H -1.33810400 3.11309000 -1.39941700
H -3.09404900 4.73522500 -2.05038300
I -0.34992400 0.22803200 -2.22411900
N -6.46557000 2.01252900 -0.44575000
O -7.28232000 2.83787700 -0.79006300
O -6.71865400 0.96465100 0.10156300

TFA-INT5

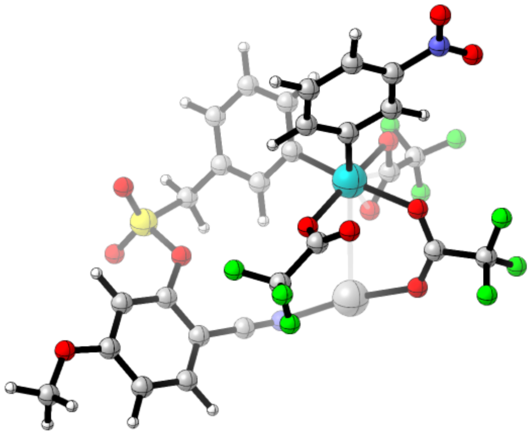

Charge: 0

Spin: 1

Pd 1.47154600 0.45367400 0.04730000
O 1.52795700 0.57791500 -2.36268700
C 2.54933700 1.22476700 -2.10997200
O 2.94898500 1.46714400 -0.92851700
C 3.39807200 1.83504300 -3.23889800

C	2.60179300	-2.34035800	-0.75456700
O	2.74640700	-1.26866000	-0.14237700
O	1.67691000	-2.71643800	-1.50041300
C	3.75040500	-3.36607700	-0.60390500
Ag	-0.12730500	-1.73622300	-2.02823400
C	0.98600200	3.34465800	0.52892600
C	0.43814400	2.14968000	0.06764400
C	-0.81349800	2.10844200	-0.53027700
C	-1.54729400	3.29480000	-0.66922400
C	-1.02393200	4.49215400	-0.17542700
C	0.23671000	4.51568500	0.41519600
H	1.98494400	3.37208100	0.96711900
H	-1.23114600	1.16689700	-0.88985500
H	-1.60034300	5.41614600	-0.26681300
H	0.65045700	5.45748600	0.78297900
C	-2.87725500	3.28276200	-1.35477100
H	-2.91109000	2.63939100	-2.24560700
H	-3.21129800	4.29121700	-1.64230900
S	-4.23616700	2.70376900	-0.34018400
O	-4.19052100	3.27231400	0.98743100
O	-5.45699300	2.70010000	-1.11961200
C	-4.60424000	0.10125900	-0.07432100
C	-5.70596500	0.18619300	0.75369500
C	-4.32890000	-1.08765100	-0.78650800
C	-6.56254700	-0.91913000	0.88398200
C	-5.17979800	-2.19123100	-0.62985000
C	-6.29331800	-2.11360500	0.18952600
H	-4.95441400	-3.11846900	-1.15937000
H	-6.94219900	-2.98214200	0.29239300
C	-3.15489000	-1.16112800	-1.56900800
N	-2.16696900	-1.22725500	-2.17531300
O	-3.70988000	1.10966000	-0.23066000
C	-8.50841300	-1.80092900	1.91474500
H	-9.26072100	-1.42078300	2.61592700
H	-9.00864200	-2.09993900	0.97825400
H	-8.01154600	-2.67858300	2.36142600
H	-5.91685800	1.09274500	1.32135900
O	-7.59962700	-0.74693800	1.70051800
F	3.21206600	3.15445300	-3.25821300
F	3.04372600	1.34136300	-4.41401600
F	4.68607000	1.59154400	-3.04098000
F	3.26406400	-4.55291600	-0.25381600
F	4.37694400	-3.49900700	-1.77457300
F	4.63773500	-2.99085800	0.30301200
C	3.11766000	1.21781200	4.42057000
C	3.94610700	0.93269500	3.34106900
C	3.45784300	0.70989300	2.05514900
C	2.08686600	0.79041600	1.89257500
C	1.21138600	1.07358400	2.93319700
C	1.74420100	1.28780000	4.20616000
H	3.56152700	1.37814000	5.40288500
H	4.14452300	0.47264700	1.24167000
H	0.13527600	1.13051600	2.76245800
H	1.07241500	1.51117600	5.03786600
N	5.40262800	0.86212700	3.55620300
O	5.80305100	1.02760600	4.68667400
O	6.09892400	0.64904900	2.59092200
O	-0.15031300	-0.51917200	0.68296800
C	-0.09289300	-1.51951400	1.50797300
O	0.84885100	-1.98554300	2.08870500

C -1.50532300 -2.13899600 1.67292400

F -1.84124400 -2.80587500 0.55344600

F -1.54273300 -2.98943000 2.67981000

F -2.43520700 -1.20370700 1.87469600

TFA-TS3

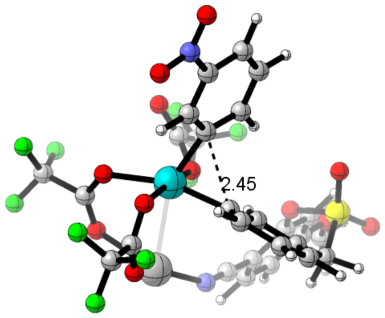

Charge: 0

Spin: 1

Pd -1.54039400 -0.03416500 -0.18645000

O -2.30466100 -1.43767100 2.34748700

C -3.25028800 -0.81310300 1.87654800

O -3.28204200 -0.09173600 0.83233300

C -4.60170300 -0.81395900 2.62634500

C -1.52884200 -2.98727800 -0.86307200

O -2.10494800 -1.88664500 -1.03670600

O -0.70663200 -3.30532100 0.00316300

C -1.86429500 -4.06666900 -1.91922700

Ag -0.14036400 -1.89923400 1.68652500

C -1.76708700 2.11136200 1.90415000

C -0.89799700 1.38534500 1.08914400

C 0.47887800 1.50764300 1.21715800

C 1.01444100 2.35273600 2.19639100

C 0.15360500 3.07849000 3.02145800

C -1.22708800 2.95018500 2.87716700

H -2.84861600 2.03434200 1.78723200

H 1.13463500 0.96127400 0.54075200

H 0.56039500 3.75415000 3.77831900

H -1.89833000 3.52368700 3.52055000

C 2.49969700 2.48329800 2.35789200

H 2.98094000 1.58810400 2.78436000

H 2.77575700 3.34400600 2.98507000

S 3.38608700 2.79737800 0.82346000

O 2.57683800 3.56124600 -0.09219900

O 4.75145900 3.15253400 1.15020000

C 4.46864400 0.41998800 0.28169100

C 5.69900600 0.73947700 -0.25685600

C 4.26292500 -0.82602800 0.91782800

C 6.75854400 -0.17884400 -0.17451600

C 5.32466100 -1.74005500 0.97938900

C 6.56450200 -1.42588600 0.44588300

H 5.16551900 -2.71072700 1.45233100

H 7.37125200 -2.15503600 0.50798100

C 2.99136100 -1.15011800 1.45211500

N 1.95462900 -1.41019600 1.90251300

O 3.39865900 1.25500300 0.16297200

C 9.02158200 -0.64271700 -0.71000900

H 9.83130500 -0.10358600 -1.21580900

H 9.33736000 -0.88136100 0.31970800

H 8.81685900 -1.57881800 -1.25651000

H 5.85723400 1.69840200 -0.74773600

O 7.90635500 0.21641000 -0.72200800

F -4.84410400 0.41955200 3.07880300

F -4.57894100 -1.64117000 3.65625500

F -5.58926500 -1.16532300 1.81450800

F -0.93281500 -4.02672600 -2.87463400

F -1.85453900 -5.28062500 -1.37972900

F -3.04804600 -3.86766600 -2.47858000

C -2.65667700 3.84236300 -2.70629100

C -3.60779800 3.05007300 -2.07556600

C -3.27014400 1.97323600 -1.25719200

C -1.92051800 1.70799700 -1.11014000

C -0.92422200 2.48082200 -1.69643700

C -1.31048500 3.54997500 -2.50561600

H -2.98480600 4.67214800 -3.33196600

H -4.05692000 1.38902800 -0.77850400

H 0.13295400 2.27328900 -1.52854400

H -0.53978000 4.16302800 -2.97752700

N -5.03712100 3.35919400 -2.25981100

O -5.31291700 4.28191200 -2.99243300

O -5.83658300 2.67487700 -1.66323300

O 0.25354100 -0.18241100 -1.01896400

C 0.39164100 -0.42279500 -2.28721500

O -0.43779900 -0.42225300 -3.15488700

C 1.88984000 -0.66176100 -2.60118600

F 2.45725300 -1.47917200 -1.70643700

F 2.05253400 -1.18382900 -3.80173300

F 2.54417900 0.50411100 -2.55387500

TFA-INT6

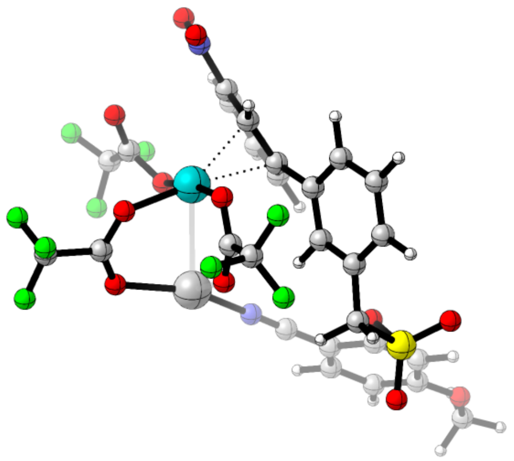

Charge: 0

Spin: 1

Pd -2.00908500 -0.34148000 0.45643800

O -0.19220300 -1.92030000 -1.30094100

C -0.75100100 -2.63256900 -0.47474700

O -1.55997700 -2.30137100 0.44824200

C -0.47853500 -4.15431600 -0.53225000

C -3.03287100 -0.24730400 -2.32773800

O -3.18997700 -0.69009300 -1.16233200

O -2.08343500 0.36767400 -2.81796000

C -4.25442200 -0.52714400 -3.23598300

Ag -0.20668800 0.63697000 -1.77100300

C 0.49458600 -2.29776900 3.06127200

C 0.55234600 -1.25378100 2.12835500

C 1.49739800 -1.31752300 1.10403000

C 2.33658500 -2.42320500 0.96964000

C 2.28084500 -3.44889900 1.91616300

C 1.36491200 -3.37855000 2.96214300

H -0.23176300 -2.26196200 3.87570500

H 1.53906300 -0.52617000 0.35675200

H 2.94069500 -4.31429400 1.82127000

H 1.31399500 -4.18470500 3.69656700

C 3.23942500 -2.52050100 -0.21825900
H 2.79385300 -2.09082900 -1.12684400
H 3.53847300 -3.55655300 -0.43717900
S 4.80847600 -1.68810500 -0.00820800
O 5.45253400 -2.08851000 1.22214500
O 5.53064300 -1.64755000 -1.26230100
C 4.88065200 0.95001000 -0.13072300
C 6.21817700 1.11915900 0.16955000
C 4.16606600 1.95146600 -0.82855300
C 6.87455300 2.29608500 -0.22761300
C 4.82743000 3.13018300 -1.20163000
C 6.17027400 3.30656700 -0.91005500
H 4.27732100 3.90830500 -1.73387000
H 6.66568700 4.22705000 -1.21573500
C 2.80488700 1.70640900 -1.13058500
N 1.69665300 1.45171000 -1.35421000
O 4.17047400 -0.14336600 0.24421600
C 8.91184700 3.51181300 -0.25413200
H 9.92985000 3.33558400 0.11274700
H 8.94313400 3.65312300 -1.34750600
H 8.51041800 4.42204000 0.22237000
H 6.77092100 0.35418300 0.71595800
O 8.16261100 2.37198700 0.09704300
F -0.62069100 -4.73950500 0.65014700
F 0.76693900 -4.38583400 -0.95683000
F -1.31815400 -4.72605500 -1.39059700
F -5.28562200 0.20584900 -2.82416900
F -3.99714100 -0.21890100 -4.49801100
F -4.59458300 -1.80964600 -3.17699900
C -1.88330000 2.12467700 3.13167500
C -2.40774200 0.85481600 3.18110500
C -1.69918400 -0.27236000 2.71185900
C -0.37294900 -0.09781200 2.23513000
C 0.13869300 1.22789300 2.14251800
C -0.60343800 2.30620600 2.57586500
H -2.48035300 2.96182700 3.49377700
H -2.10462300 -1.26484300 2.92124500
H 1.15636700 1.37313100 1.77541800
H -0.19367900 3.31511200 2.50315400
N -3.74983500 0.64276200 3.74649200
O -4.31696500 1.60865400 4.19549700
O -4.17208400 -0.49091300 3.74067600
O -2.29124300 1.60837300 0.22875700
C -3.45360100 2.11603500 0.46759900
O -4.41114400 1.59508600 0.98253800
C -3.46899600 3.60912100 0.06402000
F -3.00952000 3.78354000 -1.17472900
F -4.68241300 4.12197800 0.13929700
F -2.67105100 4.29949500 0.89588600

9

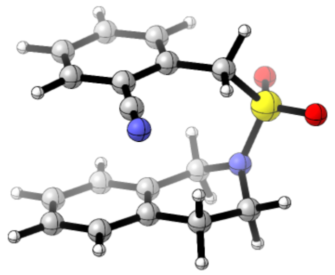

Charge: 0

Spin: 1

S 2.56835400 0.10043300 -0.16981600
O 3.65905800 -0.52991000 0.55824100
O 2.81360400 1.00351900 -1.28868300

C 0.36432900 1.64593800 0.61312500

C 0.32965700 2.53597800 -0.46495300

C -0.85514600 1.32069600 1.24524600

C -0.87587100 3.06798000 -0.91515300

C -2.06773200 1.85804700 0.78972300

C -2.07653900 2.72692700 -0.29308500

H -2.99537300 1.57811300 1.29089100

H -3.02190900 3.13941000 -0.65157200

C -0.87167700 0.45063400 2.38218900

N -0.87381800 -0.25237500 3.30511100

H 1.26612400 2.78863400 -0.96566100

C 0.58174400 -0.84751200 -1.72891100

C 1.46955800 -2.37365700 -0.03967800

H 0.77326800 -1.51073000 -2.59444600

H 0.69232700 0.18051000 -2.09899400

C 0.15088400 -2.46496000 0.71579100

H 1.53110700 -3.16176700 -0.81100200

H 2.33461700 -2.50551700 0.62304700

H -0.06821800 -3.51852200 0.95381700

H 0.23518600 -1.95466400 1.69010600

C -0.80969600 -1.08556200 -1.19529100

C -1.00876000 -1.86357400 -0.04288200

C -2.31462700 -2.04128000 0.43126200

C -3.40417800 -1.48029700 -0.22638000

C -3.20176600 -0.72277200 -1.38114500

C -1.90934600 -0.52875700 -1.85567200

H -2.47048600 -2.62689800 1.34153900

H -4.41399500 -1.63421900 0.16190000

H -4.05043600 -0.27503000 -1.90396300

H -1.74196100 0.08055200 -2.74870600

N 1.59330800 -1.10079300 -0.72536200

H -0.87553900 3.75767700 -1.76257100

C 1.65999000 1.05646100 1.07962300

H 1.53805600 0.38093800 1.93719900

H 2.39090100 1.83214500 1.35789700

TFA-INT7

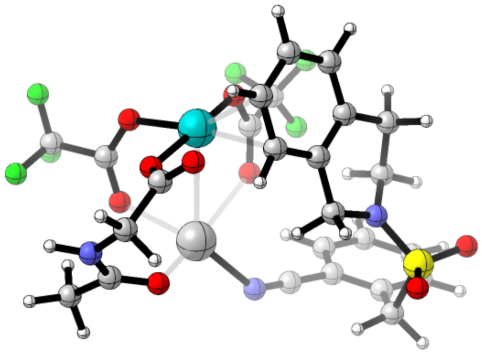

Charge: 0

Spin: 1

Pd -1.73748600 0.03784400 1.42057300

O -2.65895600 0.89742400 -1.61574500

C -3.26284700 1.41741300 -0.67614000

O -3.05237200 1.34917100 0.55904400

C -4.47992800 2.28367100 -1.07885100

C 0.30808500 1.88293500 0.80118800

O -0.51645800 1.60895400 1.72154500

O 0.54287400 1.28369300 -0.24109000

C 1.09770100 3.17947400 1.10542800

Ag -0.84823800 -0.51076300 -1.50789900

S 4.43049300 -2.09646300 -0.02396500

O 5.70324600 -1.62743900 0.50927400

O 4.01202200 -3.48496500 0.09405200

C 4.60364000 -0.26557700 -2.04472200

C 5.83875400 0.36575000 -1.88323000

C 3.47659100 0.54150700 -2.30892200

C 5.94939000 1.75095100 -1.97753000

C 3.58320500 1.93815900 -2.39231400

C 4.82379000 2.53884000 -2.22425500

H 2.68629300 2.53432700 -2.56535900

H 4.91179300 3.62566200 -2.27928500

C 2.19162200 -0.05546400 -2.46481500

N 1.14044100 -0.53190000 -2.57802600

H 6.71427700 -0.24170500 -1.64598500

C -3.13940800 -2.58265000 1.17204900

O -3.03639800 -1.34791300 0.82213000

O -2.71370200 -3.11201600 2.18208600

C -3.86504500 -3.46519700 0.14748200

N -4.26928300 -2.83357000 -1.07712700

H -5.24724800 -2.62069300 -1.21772800

C -3.37793000 -2.34233800 -1.95788800

C -3.93903400 -1.60883800 -3.14280800

H -3.88211400 -0.52967800 -2.93198400

H -3.31214600 -1.81779600 -4.01913400

H -4.98198100 -1.87632300 -3.36147800

O -2.16279100 -2.48011500 -1.78308300

H -3.16803700 -4.29041800 -0.06543000

H -4.74259900 -3.90466100 0.64075500

F -5.35731400 1.51707200 -1.73909400

F -4.09620300 3.26329500 -1.89368800

F -5.10310100 2.82076200 -0.04282400

F 0.30869300 4.24184500 0.98809100

F 2.12043300 3.32725700 0.26246300

F 1.59660900 3.16283600 2.34353800

C 3.53447300 0.19344200 1.09129300

C 1.88792200 -1.64976000 0.72021700

C 3.16812300 0.28165500 2.56221100

H 2.96947000 0.93288100 0.50170900

H 4.60339000 0.38896800 0.94561500

C 1.16537200 -1.07369100 1.90481900

H 1.29394100 -1.43662100 -0.18760100

H 1.93405800 -2.74489500 0.82287100

H 3.27583900 1.31854200 2.91488400

H 3.87281800 -0.33650800 3.14512400

C 1.75990700 -0.19283800 2.79410000

C 1.02501000 0.25724600 3.92020200

C -0.26194100 -0.16262100 4.16484300

C -0.88546100 -1.05894000 3.26592000

C -0.17723700 -1.49273400 2.12266000

H 1.50722000 0.95755600 4.60739800

H -0.80036100 0.17875600 5.05076700

H -1.82100500 -1.56682400 3.51350700

H -0.56604400 -2.34533600 1.55891500

N 3.23966000 -1.14287400 0.59544000

C 4.46364800 -1.73106700 -1.80672100

H 5.32287100 -2.30841100 -2.17759300

H 3.53996900 -2.16074800 -2.21837000

H 6.92536700 2.22288400 -1.84266900

TFA-TS4$_{meta}$

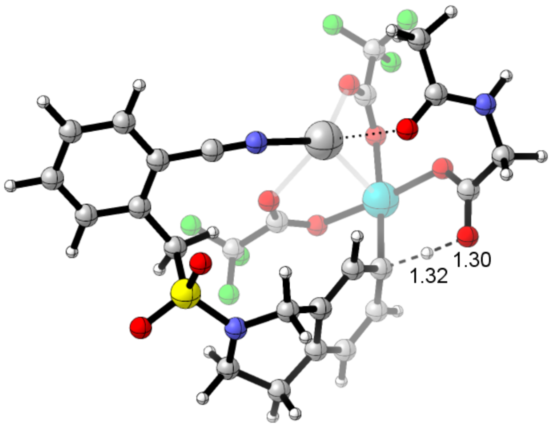

Charge: 0

Spin: 1

Pd -2.05326500 1.27401600 -0.67240000

O -2.73521300 -1.87236100 -0.36851400

C -3.35163500 -1.27287000 -1.25727400

O -3.21362200 -0.10438400 -1.68533700

C	-4.48029100	-2.09277100	-1.92428300
C	0.38813900	0.47702200	-1.90481300
O	-0.63378700	1.18416100	-2.11342000
O	0.64332400	-0.27586400	-0.96737700
C	1.49623000	0.65533900	-2.96883700
Ag	-0.89592200	-1.15145100	0.78629400
S	4.57452200	0.28462200	1.03078400
O	5.95937200	0.48200500	0.62242600
O	4.22998100	-0.38095500	2.28104400
C	-3.40660600	1.86340900	1.80743700
O	-3.48962800	1.20258000	0.74212500
O	-2.68300100	2.86926700	1.98120400
C	-4.24467000	1.38437900	2.98454700
N	-4.51260300	-0.02488100	2.93674300
H	-5.46698700	-0.34533700	2.85091100
C	-3.50746100	-0.91584100	2.76674500
C	-3.90416000	-2.34950600	2.57525100
H	-4.90458500	-2.57860000	2.96717800
H	-3.88014000	-2.55807300	1.49352400
H	-3.15866700	-2.99285000	3.05972700
O	-2.33556200	-0.54001600	2.74099400
H	-3.69398000	1.66300900	3.89565600
H	-5.19470900	1.93789700	2.98796200
F	-5.12376900	-1.42241100	-2.86668400
F	-5.37070900	-2.44729700	-0.98755500
F	-3.98842400	-3.20646600	-2.46273200
F	2.39032600	1.55116900	-2.50917800
F	1.04324500	1.09145500	-4.13079100
F	2.15136100	-0.48584000	-3.17212400
C	4.24396300	2.73668100	0.02614200
C	2.64138100	1.96255000	1.83476300
C	3.34514800	3.96945300	0.10697100
H	4.14493100	2.30034200	-0.98240500
H	5.29868600	3.01599600	0.16710000
C	1.54249600	2.54469100	0.99568100
H	2.34624900	1.00133800	2.27570800
H	2.86603100	2.63460400	2.68420200
H	3.57554200	4.64095000	-0.73235700
H	3.57686300	4.52577700	1.03349500
C	1.89649300	3.58346000	0.11886800
C	0.91886500	4.18709500	-0.67850900
C	-0.39617800	3.74236800	-0.62340700
C	-0.77122000	2.67963500	0.22762400
C	0.22597300	2.10959900	1.05000600
H	1.19846100	4.99810700	-1.35626800
H	-1.14793300	4.20425600	-1.26902600
H	-1.83810900	2.85555800	0.98809500
H	-0.04870500	1.31945400	1.75776800
N	3.85467600	1.76509000	1.04666500
C	3.77536800	-0.65869600	-0.29897000
H	4.15168200	-0.24104100	-1.24121600
C	4.13995800	-2.09637600	-0.13724800
C	5.36680900	-2.57569600	-0.59898800
C	3.30328500	-2.98490400	0.57111800
C	5.74321000	-3.89936200	-0.38684300
H	6.03865500	-1.88771200	-1.11657000
C	3.67986400	-4.31922600	0.78278300
C	4.90067800	-4.77372500	0.30067300
H	6.70786200	-4.25187500	-0.75914700
H	3.00995300	-4.98724400	1.32707100
H	5.19761300	-5.81154800	0.46472200
H	2.69133900	-0.48044500	-0.24124800
C	2.04568600	-2.52486300	1.06506200
N	1.01435300	-2.14521700	1.43324200

TFA-TS4$_{ortho}$

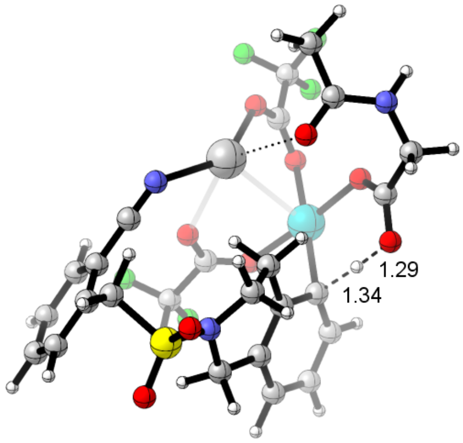

Charge: 0

Spin: 1

Pd 1.75312000 -0.36419200 1.41104300

O 3.08712300 -0.46046400 -1.63438100

C 3.66042900 -1.09386400 -0.73726500

O 3.29878300 -1.34038500 0.43263000

C 5.05177900 -1.64010200 -1.13350400

C -0.20956300 -2.20833500 0.49831300

O 0.63190300 -2.04265500 1.42308900

O -0.38100700 -1.56228400 -0.53191600

C -1.19718000 -3.37067600 0.76081900

Ag 0.98816900 0.32018900 -1.58519100

S -4.67921500 2.13411300 -0.37496500

O -5.83839300 1.51707700 0.25979800

O -4.61024100 3.57292300 -0.59187800

C -4.41553000 -0.09782900 -1.96560800

C -5.54468300 -0.85256100 -1.63941500

C -3.20000600 -0.78082300 -2.17466200

C -5.46660600 -2.23810600 -1.51967700

C -3.11957200 -2.17630400 -2.05350300

C -4.25538900 -2.90073600 -1.72040200

H -2.15479600 -2.66667100 -2.17785300

H -4.18711900 -3.98371100 -1.60192800

C -2.00118400 -0.07211000 -2.48065500

N -0.99503600 0.46489000 -2.69905900

H -6.48582300 -0.33485900 -1.44582700

C 2.52685200 2.40301800 1.67933900

O 2.91425600 1.28390900 1.26990200

O 1.59747800 2.59194000 2.50168800

C 3.21970100 3.63352400 1.11328900

N 3.81308300 3.40027400 -0.17172700

H 4.81677200 3.46132100 -0.27067400

C 3.08863400 2.87955200 -1.19144500

C 3.84348200 2.53955000 -2.44247100

H 4.06974500 1.46163500 -2.41237300

H 3.19745200 2.72458100 -3.30997300

H 4.78015800 3.10363600 -2.54933400

O 1.88233500 2.67386500 -1.06492200

H 2.46047800 4.42945300 1.07177500

H 3.99511900 3.95636600 1.82288300

F 4.95484400 -2.43057600 -2.19982900

F 5.63828800 -2.31944000 -0.16123500

F 5.84546300 -0.60929900 -1.45588600

F -2.39565900 -2.85733700 1.07024000

F -0.82361600 -4.16657500 1.74824100

F -1.35138000 -4.11777200 -0.33742700

C -2.17386600 2.50759900 0.45515500

C -3.55482700 0.81696400 1.64983500

C -0.92781500 1.66961300 0.63988400

H -2.25695600 3.26996500 1.25238000

H -2.13802800 3.04913700 -0.49948900

C -2.26989800 0.44948900 2.35179600

H -4.21873200 1.33842400 2.36346600

H -4.07620200 -0.10816400 1.35799500

H -0.03787800 2.31240700 0.66368700

H -0.82260400 1.00928800 -0.23566100

C -1.00976300 0.81586700 1.86864400

C 0.16478900 0.40713000 2.55516500

C 0.03390300 -0.30943400 3.76120400

C -1.21933400 -0.65865300 4.25078500

C -2.35660900 -0.29580600 3.53467400

H 0.98544700 1.46605600 2.60679400

H 0.93142300 -0.61183500 4.30772500

H -1.31442500 -1.22531700 5.17957600

H -3.34374800 -0.58874300 3.90568500

N -3.33102800 1.62263600 0.45380700

C -4.49257000 1.39099000 -2.01642500

H -6.36171700 -2.80544500 -1.25420500

H -5.38300600 1.75195100 -2.55291400

H -3.60513800 1.86392500 -2.45967900

TFA-INT8

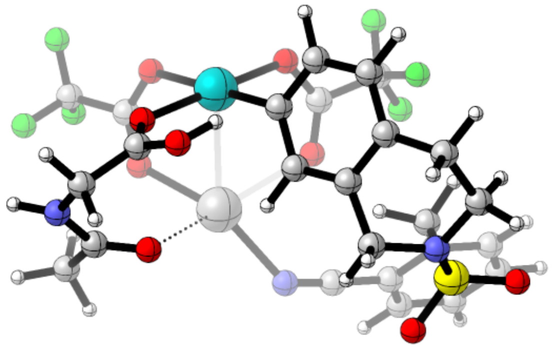

Charge: 0

Spin: 1

Pd -1.74251700 -0.70326500 1.32931000

O -3.13999000 1.56049200 -0.55951300

C -3.74395600 1.32015800 0.50245900

O -3.39220900 0.64182700 1.48372200

C -5.16011800 1.93602200 0.56688400

C 0.41973500 1.04786900 1.93269300

O -0.50631500 0.40490500 2.49943300

O 0.59253400 1.27672400 0.73819200

C 1.52659800 1.52273500 2.90085800

Ag -1.05514000 0.93402500 -1.08473000

S 4.44927700 -0.74248700 -1.28628100

O 5.86419300 -0.62906000 -0.96056900

O 3.94060300 -0.44425500 -2.61780000

C -2.85252100 -2.78030900 -0.57909300

O -3.01780300 -1.77702100 0.10279500

O -1.94668100 -3.68794600 -0.36799800

C -3.75793000 -3.07762300 -1.75356100

N -4.23100700 -1.88116600 -2.38082700

H -5.22424100 -1.70968500 -2.45131000

C -3.35162100 -0.91171400 -2.74927600

C -3.93638800 0.32969200 -3.35118400

H -4.90024600 0.15232200 -3.84775400

H -4.07565400 1.05808300 -2.53609100

H -3.21777700 0.74866500 -4.06635600

O -2.15023900 -1.06492300 -2.55014300

H -3.19096000 -3.71545000 -2.45013700

H -4.61240200 -3.66549600 -1.38563200

F -5.77503800 1.70272200 1.71537200

F -5.90735900 1.40285100 -0.41512200

F -5.11709800 3.25186700 0.37316400

F 2.42560800 0.53024000 3.02953300

F 1.06883600 1.81904500 4.10734300

F 2.16961800 2.58219900 2.42141000

C 4.62331400 -2.90135300 0.26558300

C 2.64438300 -2.70641600 -1.27328100

C 3.65430200 -3.86521400 0.95689700

H 4.92098100 -2.11489900 0.97852400

H 5.54729100 -3.42357800 -0.02846800

C 1.76133000 -2.66305000 -0.05837400

H 2.28428100 -2.05412400 -2.08005000
H 2.69478400 -3.72630200 -1.69368700
H 4.07121500 -4.15973000 1.93083700
H 3.57632100 -4.78936300 0.35679400
C 2.29554900 -3.24560600 1.10110000
C 1.57200800 -3.17959000 2.29104100
C 0.34304400 -2.51798800 2.33493900
C -0.19118900 -1.91757200 1.18522000
C 0.52934100 -2.01165400 -0.01787200
H 1.98167800 -3.62504700 3.20233600
H -0.18412100 -2.43029100 3.28859300
H -1.31940000 -3.36956300 0.32692400
H 0.14064300 -1.56305800 -0.93921300
N 4.00069300 -2.29220100 -0.91381200
C 3.54898100 0.34395900 -0.14186000
H 3.89032800 0.10310500 0.87356300
C 3.84282400 1.75929100 -0.51071500
C 5.00024600 2.38346700 -0.04483900
C 3.01979700 2.46995600 -1.40946000
C 5.32366200 3.67899900 -0.43898600
H 5.66097300 1.83209100 0.62693500
C 3.34397100 3.77540900 -1.80625700
C 4.49668700 4.37772400 -1.31862200
H 6.23377100 4.14738000 -0.05737600
H 2.68376600 4.30676400 -2.49400100
H 4.75080100 5.39419600 -1.62581700
H 2.48121200 0.09799900 -0.21918700
C 1.81426200 1.87987000 -1.89029200
N 0.80659800 1.41738700 -2.23237600

TFA-INT9

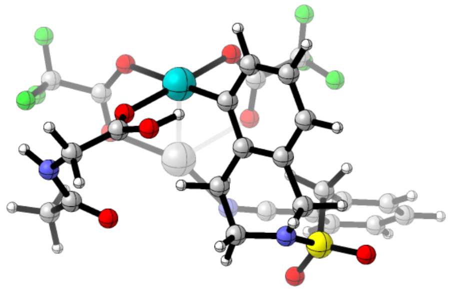

Charge: 0

Spin: 1

Pd 1.55868800 0.14625000 1.45336800
O 2.73276100 -1.75635000 -1.04848400
C 3.39240100 -1.72133700 0.01113200
O 3.03721300 -1.40048000 1.15533300
C 4.88791900 -2.07157600 -0.16937400
C -0.77027800 -1.42303200 1.93261100
O 0.24045300 -0.96602100 2.52505800
O -1.00839800 -1.47612700 0.72422300
C -1.89451600 -1.94684500 2.85280900
Ag 0.57555300 -1.50461100 -1.13810800
S -3.68616000 1.72205000 -1.44866600
O -4.93817600 2.39425500 -1.12903300
O -3.44792300 1.12097600 -2.75422100
C -4.37389900 -0.75569000 -0.64934200
C -5.72101200 -0.81328500 -0.29366400
C -3.86961200 -1.75727200 -1.50802600
C -6.53788700 -1.84490500 -0.75058200
C -4.68957600 -2.79732300 -1.96668900
C -6.02447000 -2.83905900 -1.58358000
H -4.27176200 -3.56335500 -2.62227700
H -6.66677800 -3.64737000 -1.93872600
C -2.49782700 -1.71685000 -1.89564300
N -1.36935100 -1.67398200 -2.15772200
H -6.13148200 -0.02684600 0.34299500

C	3.09726100	2.44322700	0.18138200
O	2.92894800	1.24728400	0.38964700
O	2.41578200	3.39786200	0.73959300
C	4.18332900	2.89322200	-0.76886400
N	4.52781400	1.87539100	-1.71094700
H	5.32528300	1.28010000	-1.52511900
C	3.54541200	1.41390600	-2.54449300
C	3.90198200	0.23763200	-3.40480500
H	3.59128300	-0.67557000	-2.87196900
H	3.33841900	0.30200000	-4.34357400
H	4.97847000	0.17267900	-3.61329500
O	2.44422900	1.94091500	-2.54547300
H	3.82338700	3.80144300	-1.27481600
H	5.06467000	3.17345900	-0.17153400
F	5.05932300	-3.09595600	-0.99510800
F	5.49183700	-2.34363400	0.97404400
F	5.51083900	-1.00387800	-0.71661700
F	-1.56320400	-1.95299700	4.13400700
F	-2.24747200	-3.18123600	2.50321600
F	-2.96754600	-1.15605100	2.70512100
C	-1.13087900	2.51677200	-1.70574300
C	-2.46846900	3.61110100	0.00026200
C	-0.32125100	1.71244400	-0.70279000
H	-0.64622500	3.48766100	-1.90199100
H	-1.21154100	1.99685100	-2.66812600
C	-1.53214800	3.07418000	1.06082500
H	-2.17997900	4.64253800	-0.27434400
H	-3.49884200	3.67497600	0.37972700
H	0.73799700	1.75289700	-0.99611400
H	-0.60303300	0.64661100	-0.77254300
C	-0.51663700	2.15593900	0.72812000
C	0.28801200	1.62749000	1.76161200
C	0.11012300	2.06026100	3.08118700
C	-0.86861200	3.00639000	3.39096000
C	-1.69244900	3.49528300	2.38444000
H	1.69190700	3.00942900	1.29223500
H	0.72104600	1.63220800	3.88049200
H	-1.00388300	3.33593800	4.42403600
H	-2.48531900	4.20967200	2.62534500
N	-2.46485400	2.81278200	-1.20708100
C	-3.51562500	0.38632400	-0.21889200
H	-7.58921400	-1.87108100	-0.45498000
H	-2.45950800	0.09872100	-0.14609000
H	-3.83310400	0.80742600	0.74348600

10. References

[1] Frisch, M. J. et al. *Gaussian 16, Revision C.01*, Gaussian, Inc., Wallingford CT, **2019**.

[2] Adamo, C.; Barone V. Toward reliable density functional methods without adjustable parameters: The PBE0 model, *J. Chem. Phys.* **1999**, *110*, 6158-6170.

[3] Weigend, F.; Ahlrichs, R. Balanced basis sets of split valence, triple zeta valence and quadruple zeta valence quality for H to Rn: Design and assessment of accuracy. *Phys. Chem. Chem. Phys*. **2005**, *7* 3297-3305.

[4] (a) Fuentealba, P.; Preuss, H.; Stoll, H.; Szentpály, L. v. A Proper Account of Core-polarization with Pseudopotentials – Single Valence-Electron Alkali Compounds, *Chem. Phys. Lett.* **1982**, *89,* 418-422. (b) Szentpály, L. v.; Fuentealba, P.; Preuss, H.; Stoll, H. Pseudopotential calculations on Rb+2, Cs+2, RbH+, CsH+ and the mixed alkali dimer ions, *Chem. Phys. Lett.* **1982**, *93,* 555-559. (c) Fuentealba, P.; Stoll, H.; Szentpály, L. v.; Schwerdtfeger, P.; Preuss, H. On the reliability of semi-empirical pseudopotentials – simulation of Hartree-Fock and Dirac-Fock results, *J. Phys. B*, **1983**, *16,* L323-L28.

[5] (a) Fukui, K. Formulation of the Reaction Coordinate. *J. Phys. Chem*. **1970**, *74*, 4161−4163. (b) Fukui, K. The Path of Chemical Reactions - the IRC Approach. *Acc. Chem. Res*. **1981**, *14*, 363−368.

[6] Zhao, Y., Truhlar, D. G. The M06 suite of density functionals for main group thermochemistry, thermochemical kinetics, noncovalent interactions, excited states, and transition elements: two new functionals and systematic testing of four M06-class functionals and 12 other functionals. *Theor. Chem. Acc.* **2008**, *120*, 215-41.

[7] Marenich, A. V.; Cramer, C. J.; and Truhlar, D. G. Universal Solvation Model Based on Solute Electron Density and on a Continuum Model of the Solvent Defined by the Bulk Dielectric Constant and Atomic Surface Tensions. *J. Phys. Chem. B* **2009**, *113*, 6378−6396.

[8] CYLview20; Legault, C. Y. Universitéde Sherbrooke, **2020**, (http://www.cylview.org)

NMR Spectra

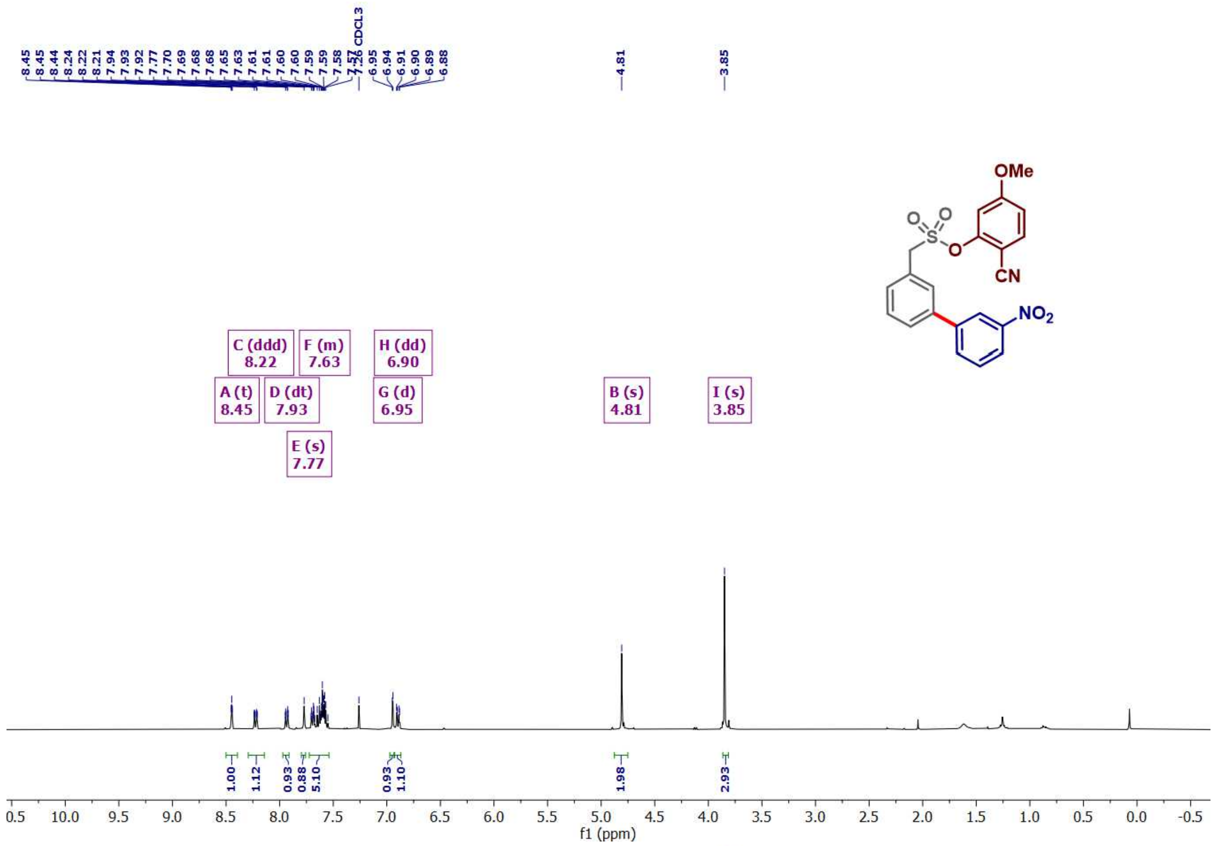
8.45
8.45
8.44
8.24
8.22
8.21
7.94
7.93
7.92
7.77
7.70
7.69
7.68
7.65
7.63
7.61
7.61
7.60
7.60
7.59
7.59
7.58
7.57
7.26 CDCL3
6.95
6.94
6.91
6.90
6.89
6.88
4.81
3.85
OMe
CN
NO2
C (ddd) 8.22
F (m) 7.63
H (dd) 6.90
A (t) 8.45
D (dt) 7.93
G (d) 6.95
B (s) 4.81
I (s) 3.85
E (s) 7.77
1.00
1.12
0.93
0.88
5.10
0.93
1.10
1.98
2.93
f1 (ppm)

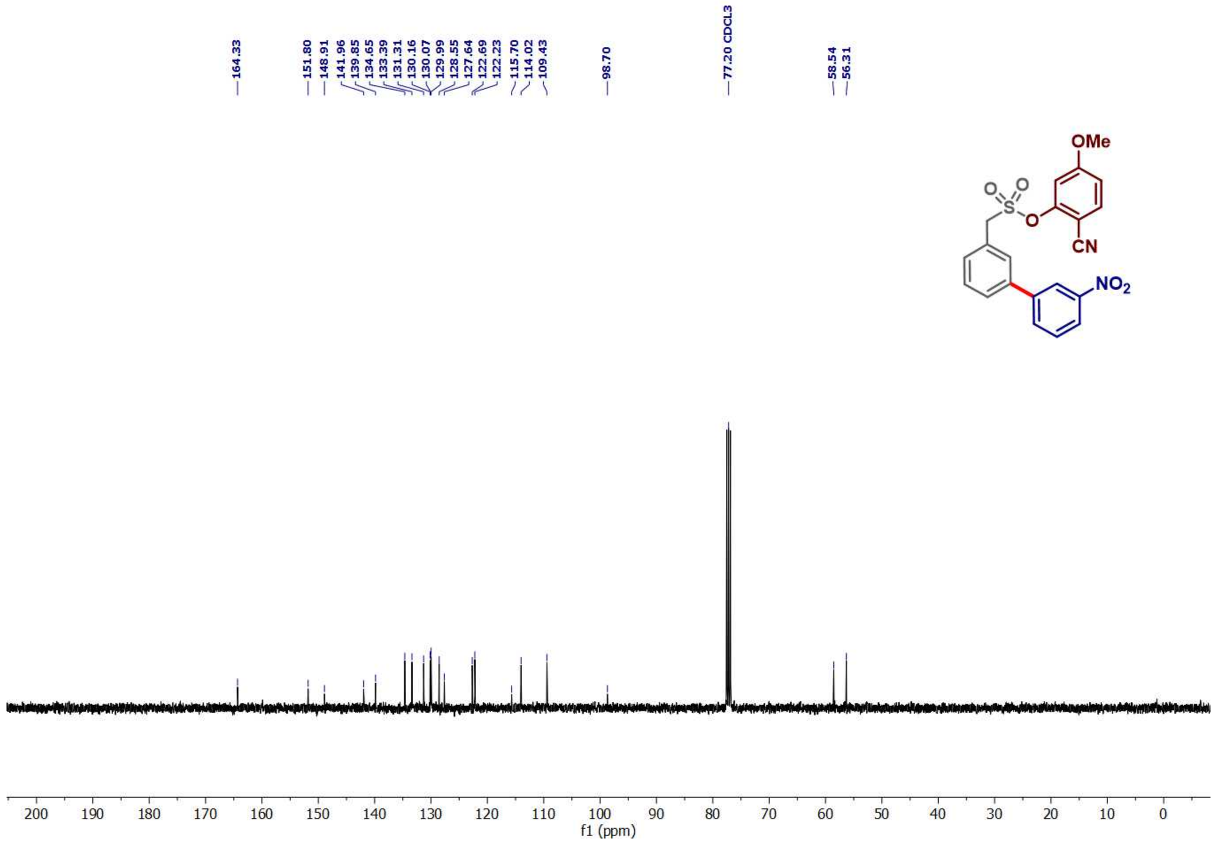
164.33
151.80
148.91
141.96
139.85
134.65
133.39
131.31
130.16
130.07
129.99
128.55
127.64
122.69
122.23
115.70
114.02
109.43
98.70
77.20 CDCL3
58.54
56.31
OMe
CN
NO2
f1 (ppm)

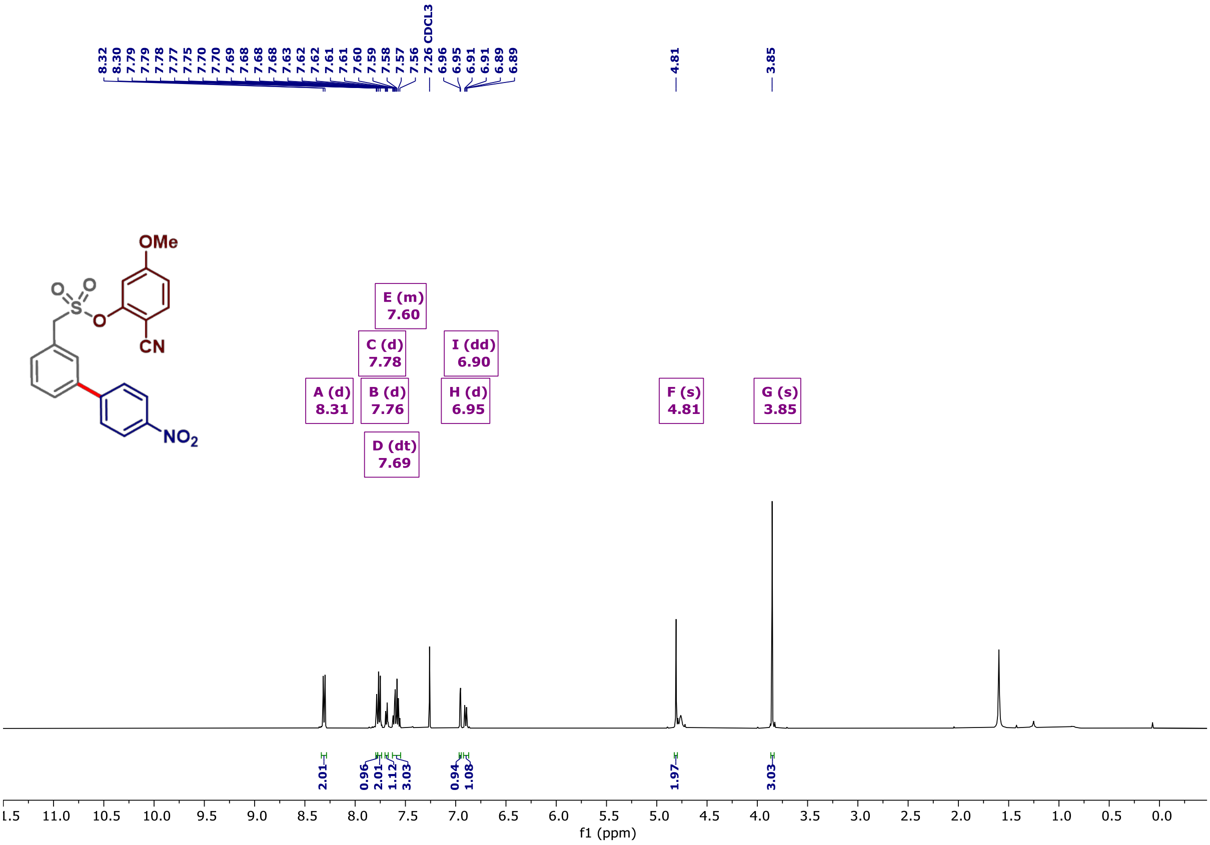
8.32
8.30
7.79
7.79
7.78
7.77
7.75
7.70
7.70
7.69
7.68
7.68
7.63
7.62
7.62
7.61
7.61
7.60
7.59
7.58
7.57
7.56
7.26 CDCL3
6.96
6.95
6.91
6.91
6.89
6.89
4.81
3.85
OMe
CN
NO_2
E (m) 7.60
C (d) 7.78
I (dd) 6.90
A (d) 8.31
B (d) 7.76
H (d) 6.95
F (s) 4.81
G (s) 3.85
D (dt) 7.69
2.01
0.96
2.01
1.12
3.03
0.94
1.08
1.97
3.03
f1 (ppm)

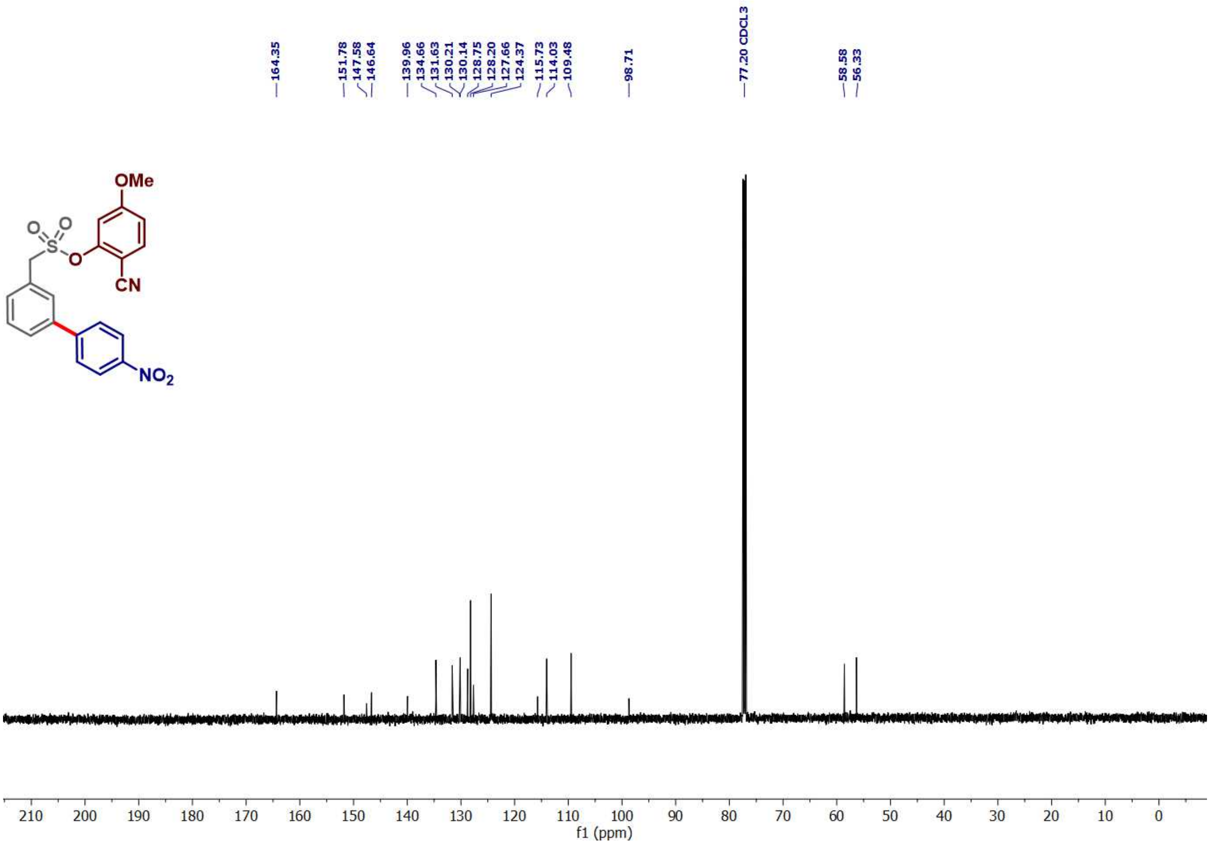
164.35
151.78
147.58
146.64
139.96
134.66
131.63
130.21
130.14
128.75
128.20
127.66
124.37
115.73
114.03
109.48
98.71
77.20 CDCL3
58.58
56.33
OMe
CN
NO_2
f1 (ppm)

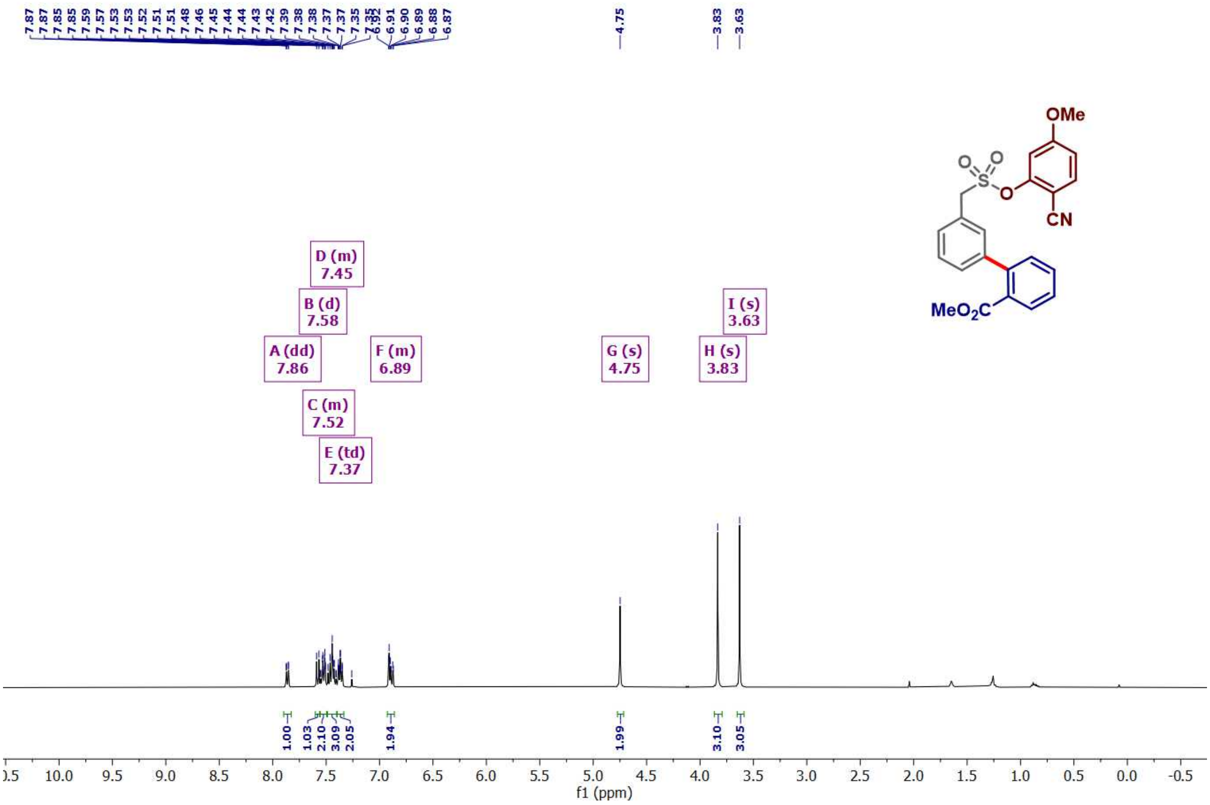

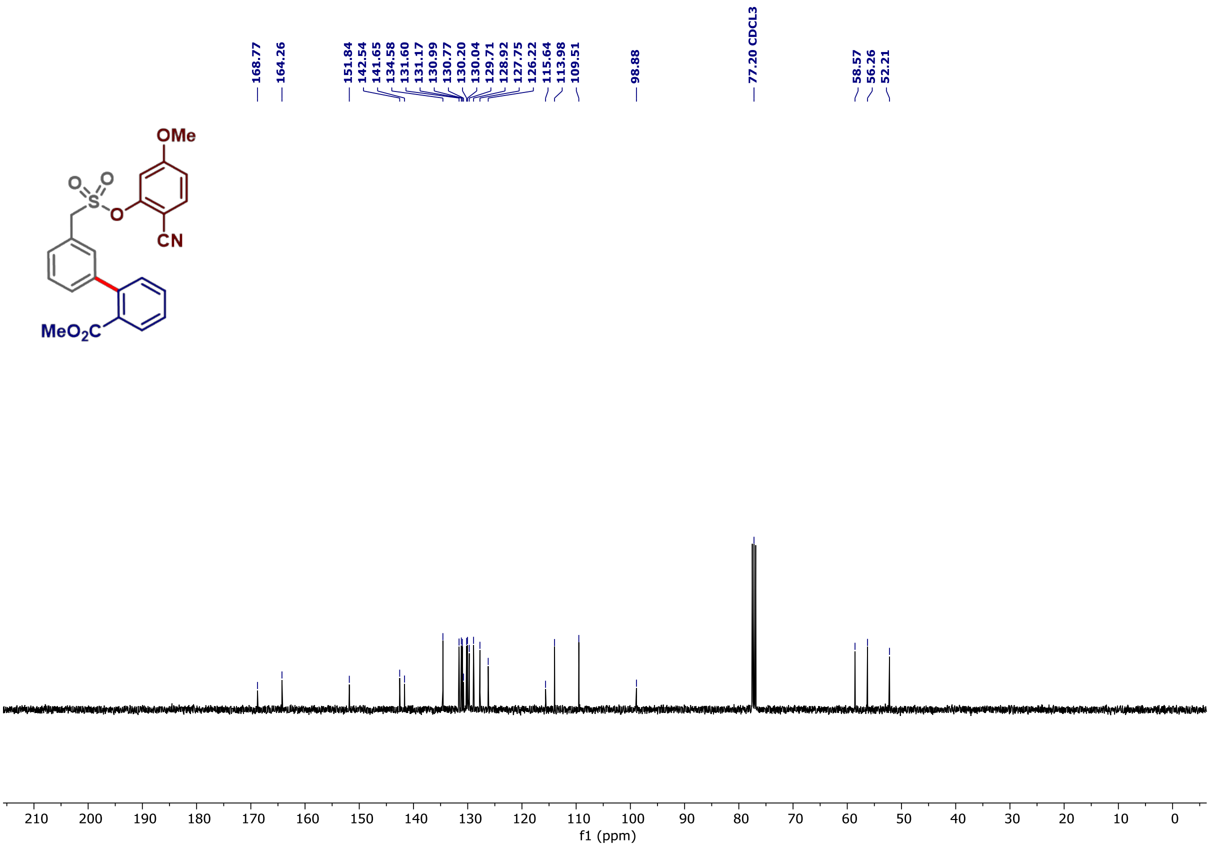

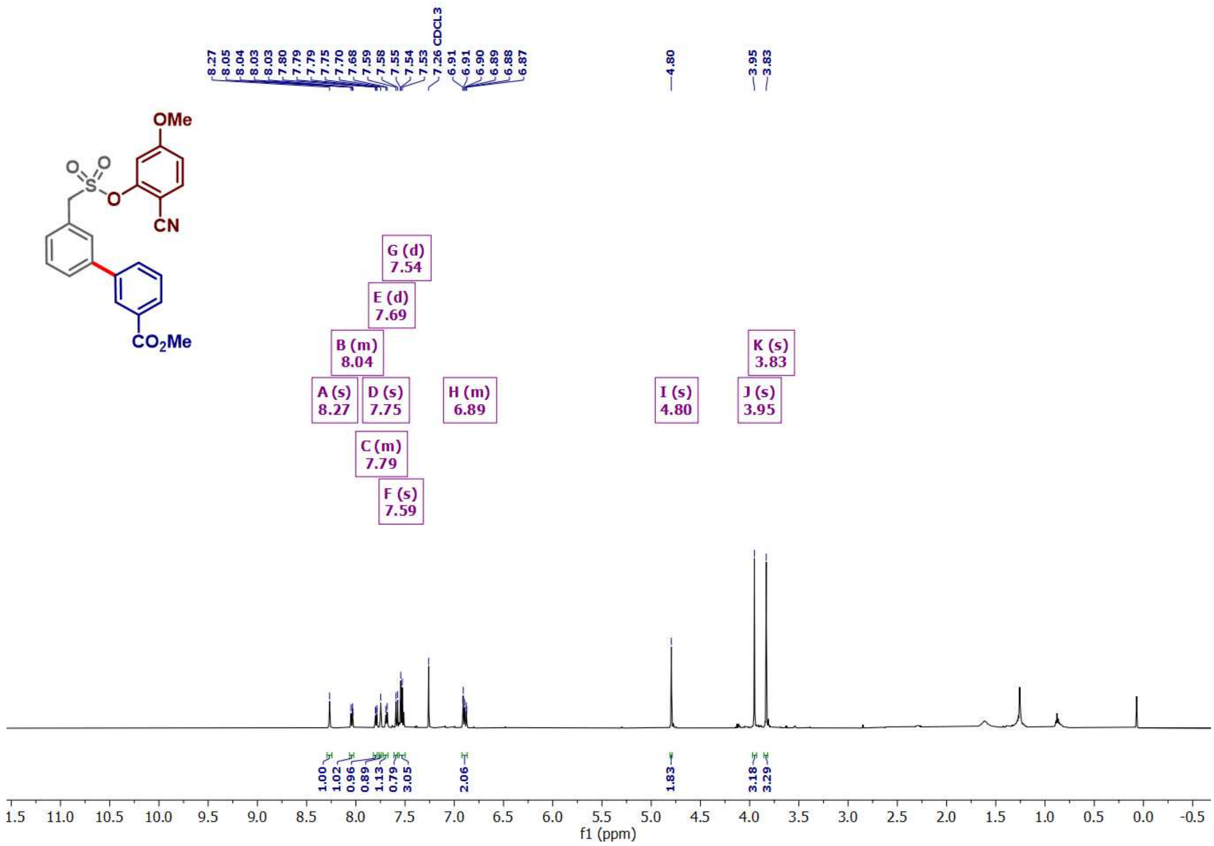

8.27
8.05
8.04
8.03
8.03
7.80
7.79
7.79
7.75
7.70
7.68
7.59
7.58
7.55
7.54
7.53
7.26 CDCL3
6.91
6.91
6.90
6.89
6.88
6.87
4.80
3.95
3.83
OMe
CN
CO_2Me
G (d) 7.54
E (d) 7.69
B (m) 8.04
K (s) 3.83
A (s) 8.27
D (s) 7.75
H (m) 6.89
I (s) 4.80
J (s) 3.95
C (m) 7.79
F (s) 7.59
1.00
1.02
0.96
0.89
1.13
0.79
3.05
2.06
1.83
3.18
3.29
f1 (ppm)

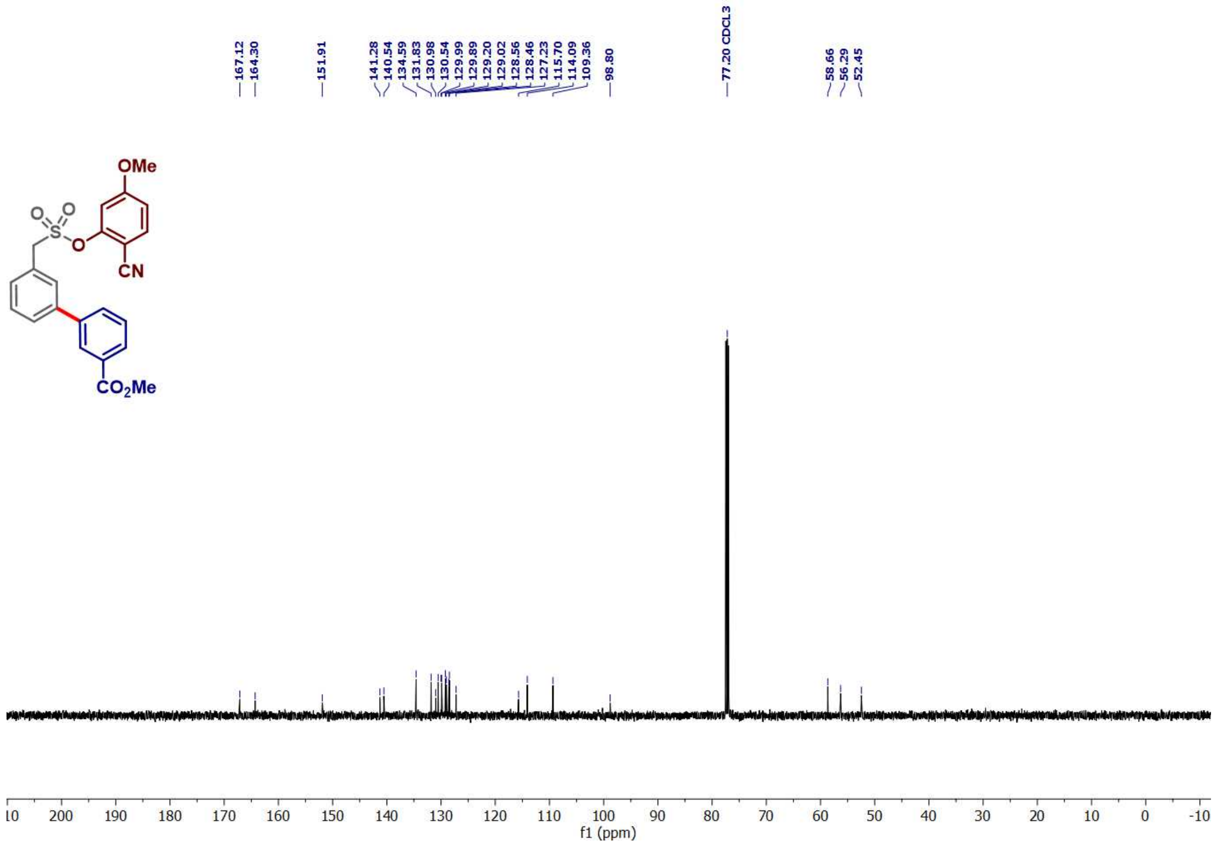

167.12
164.30
151.91
141.28
140.54
134.59
131.83
130.98
130.54
129.99
129.89
129.20
129.02
128.56
128.46
127.23
115.70
114.09
109.36
98.80
77.20 CDCL3
58.66
56.29
52.45
OMe
CN
CO_2Me
f1 (ppm)

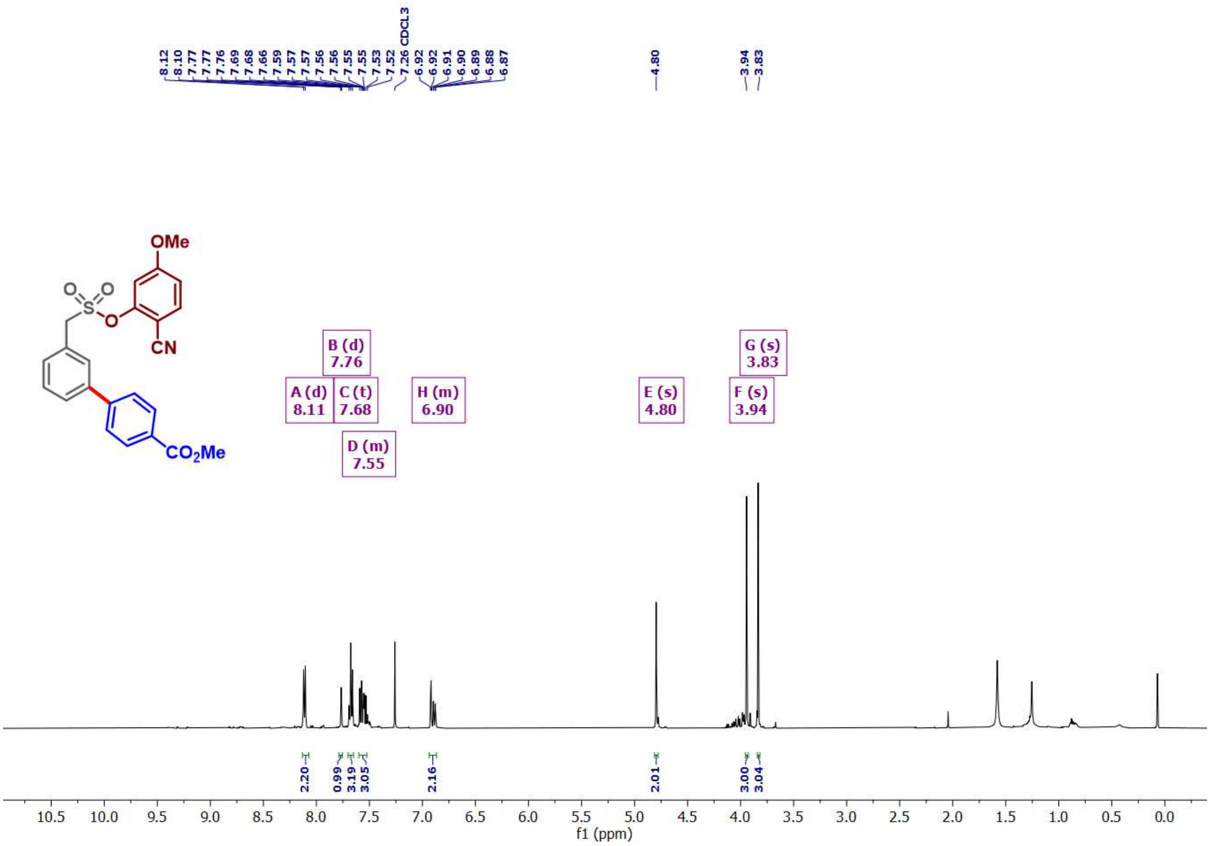
8.12
8.10
7.77
7.77
7.76
7.69
7.68
7.66
7.59
7.57
7.57
7.56
7.56
7.55
7.55
7.53
7.52
7.26 CDCL3
6.92
6.92
6.91
6.90
6.89
6.88
6.87
4.80
3.94
3.83
OMe
CN
CO2Me
A (d) 8.11
B (d) 7.76
C (t) 7.68
D (m) 7.55
H (m) 6.90
E (s) 4.80
F (s) 3.94
G (s) 3.83
2.20
0.99
3.19
3.05
2.16
2.01
3.00
3.04
f1 (ppm)

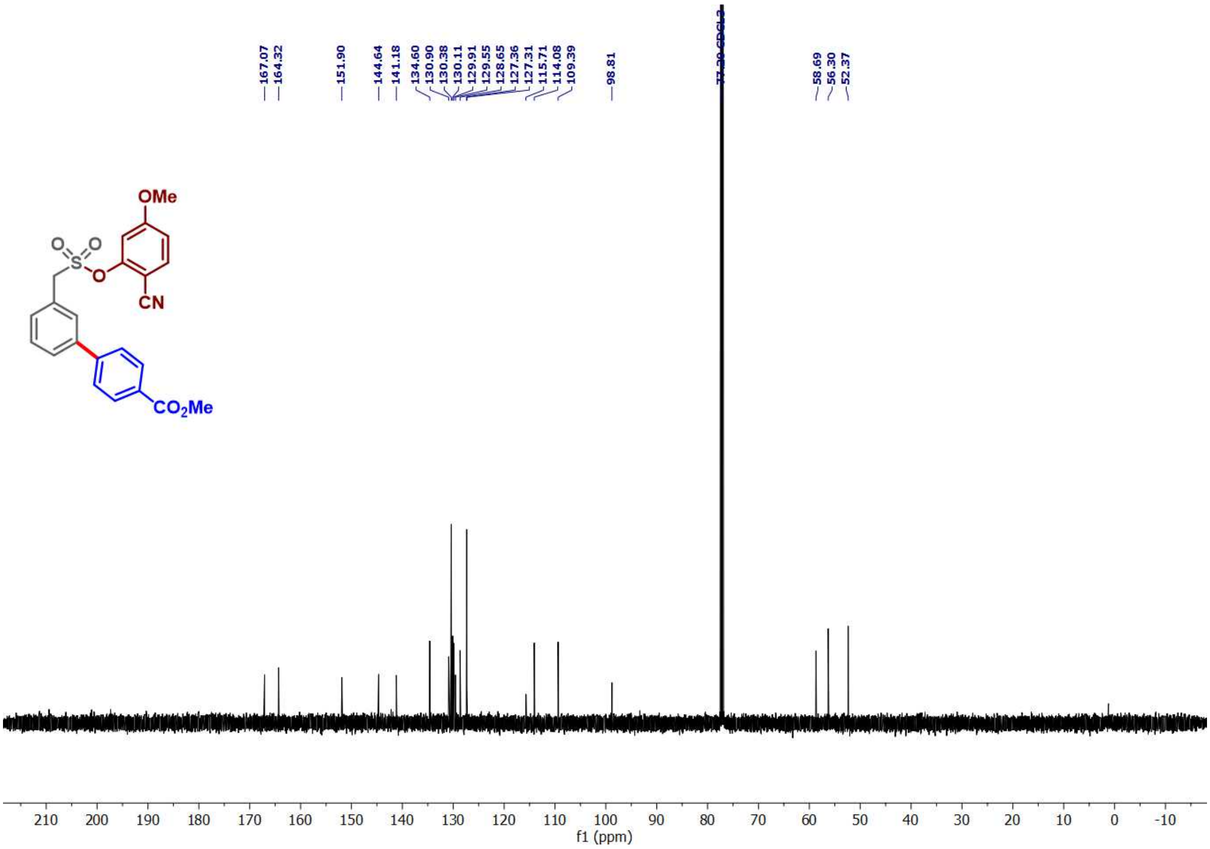
167.07
164.32
151.90
144.64
141.18
134.60
130.90
130.38
130.11
129.91
129.55
128.65
127.36
127.31
115.71
114.08
109.39
98.81
58.69
56.30
52.37
OMe
CN
CO2Me
f1 (ppm)

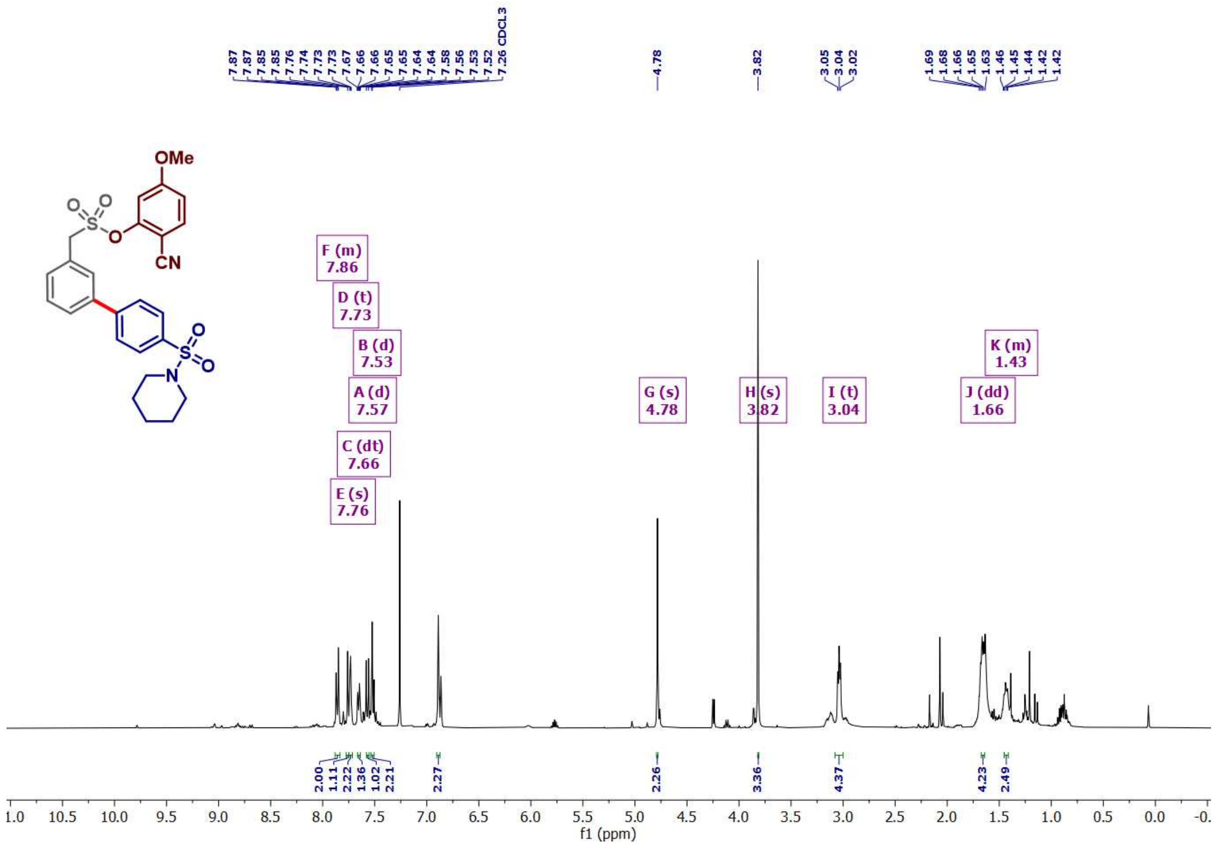
7.87
7.87
7.85
7.76
7.74
7.73
7.73
7.67
7.66
7.65
7.65
7.64
7.58
7.56
7.53
7.52
7.26 CDCL3
4.78
3.82
3.05
3.04
3.02
1.69
1.68
1.66
1.65
1.63
1.46
1.45
1.44
1.42
1.42
OMe
CN
F (m) 7.86
D (t) 7.73
B (d) 7.53
A (d) 7.57
C (dt) 7.66
E (s) 7.76
G (s) 4.78
H (s) 3.82
I (t) 3.04
J (dd) 1.66
K (m) 1.43
2.00
1.11
2.22
1.36
1.02
2.21
2.27
2.26
3.36
4.37
4.23
2.49
f1 (ppm)

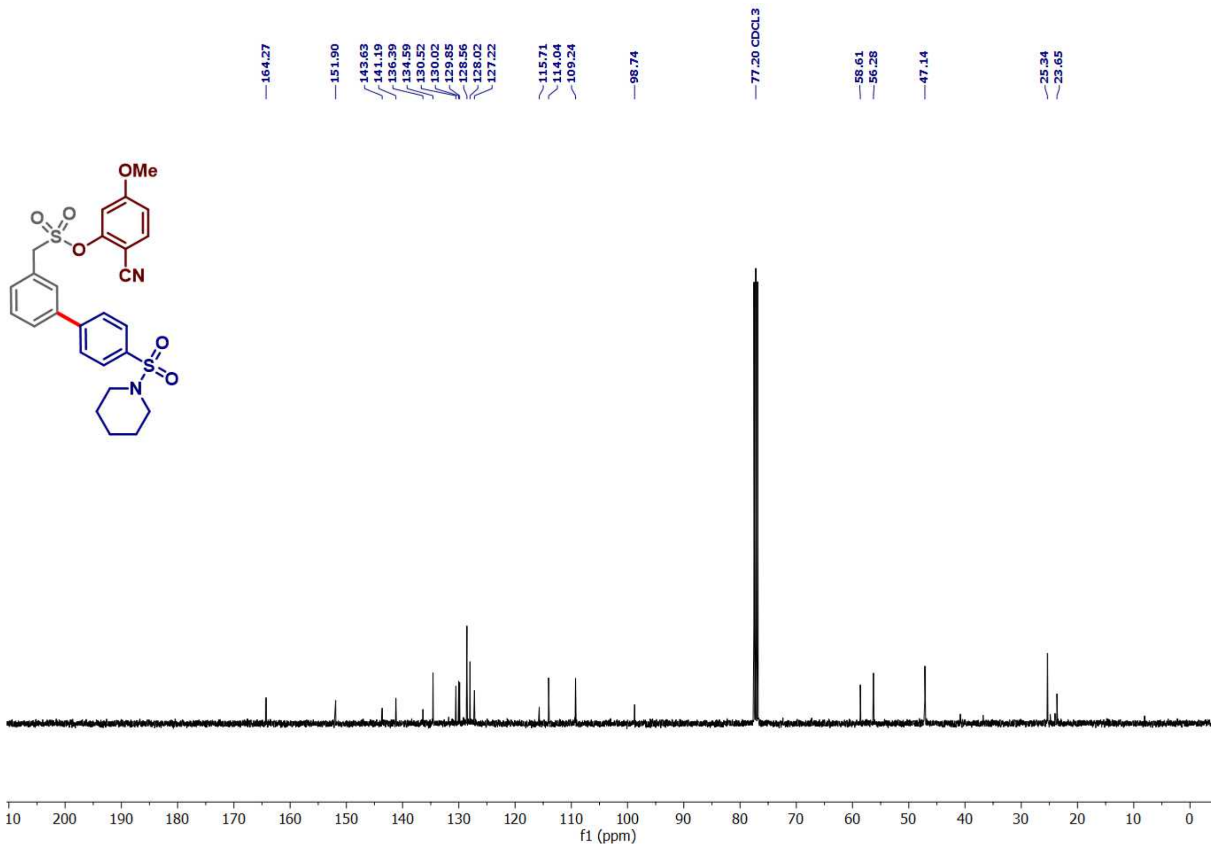
164.27
151.90
143.63
141.19
136.39
134.59
130.52
130.02
129.85
128.56
128.02
127.22
115.71
114.04
109.24
98.74
77.20 CDCL3
58.61
56.28
47.14
25.34
23.65
OMe
CN
f1 (ppm)

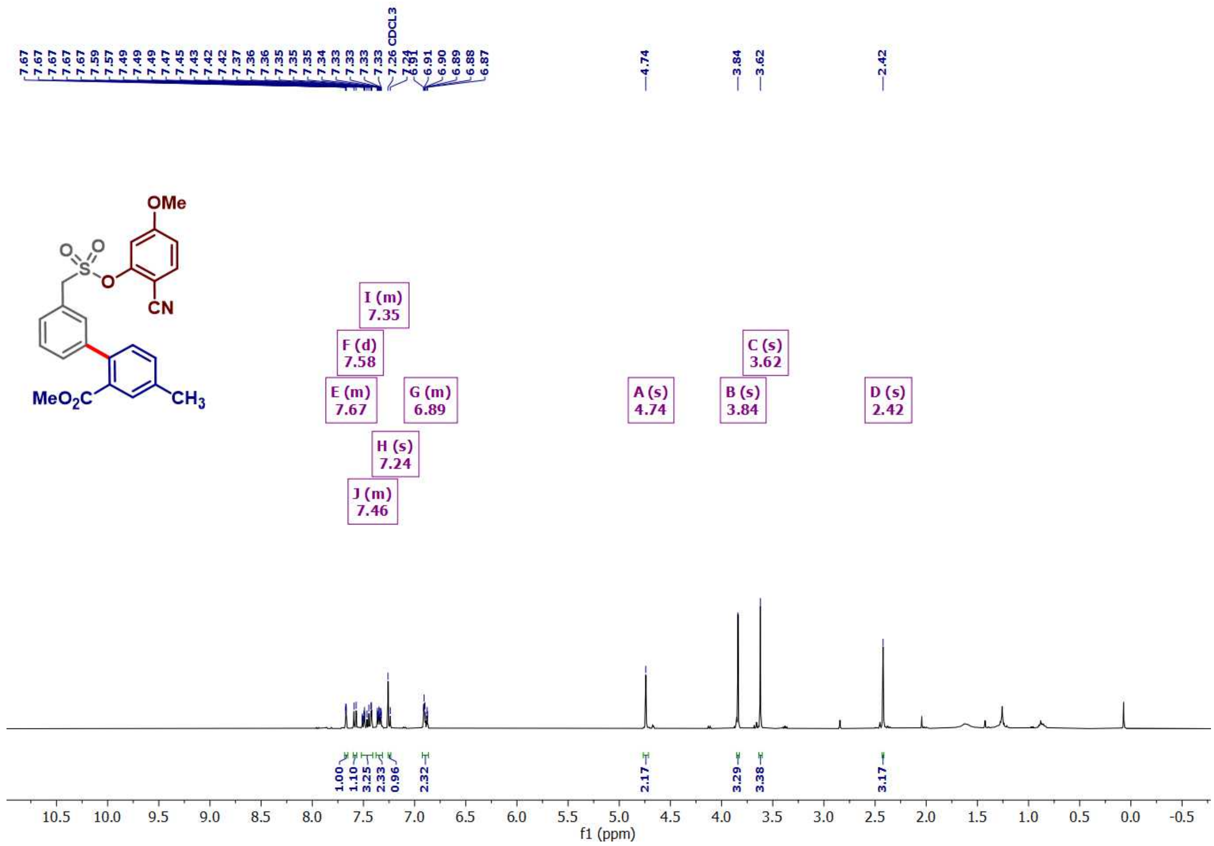

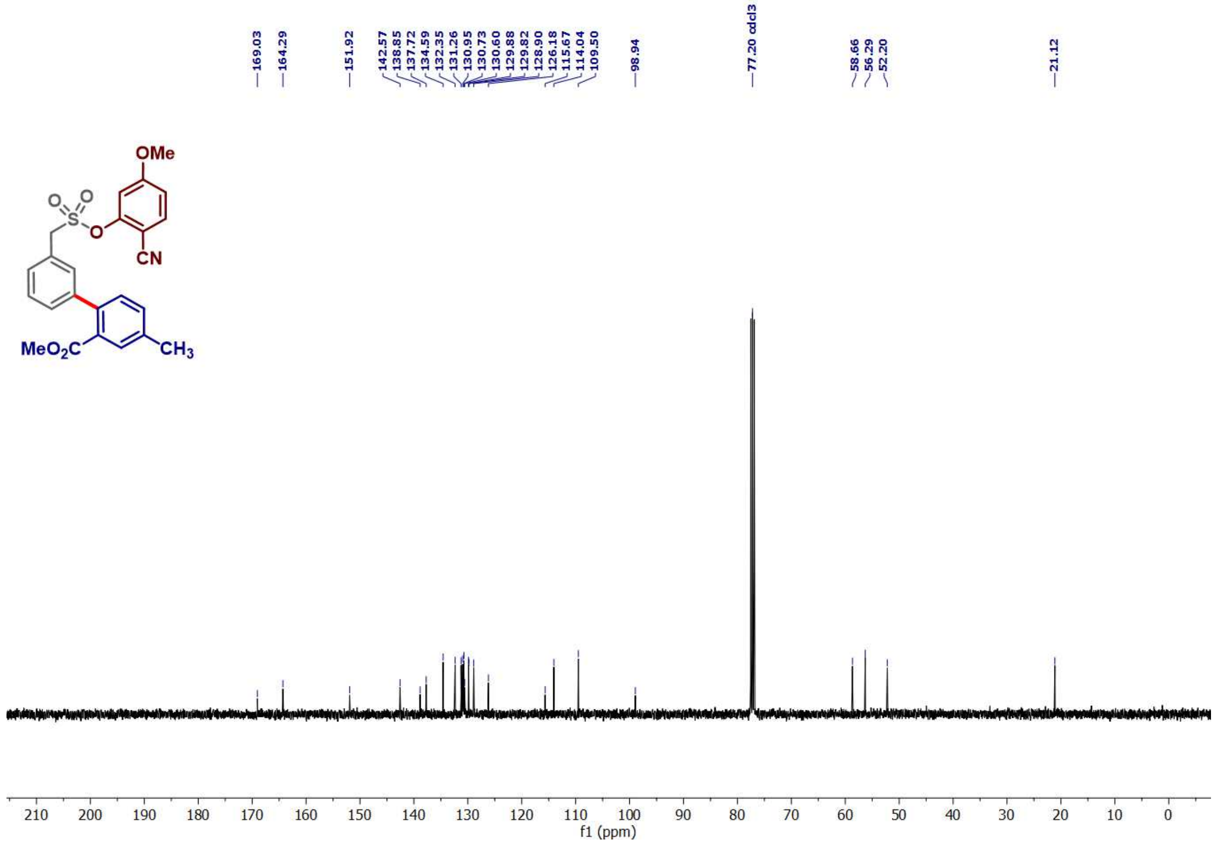

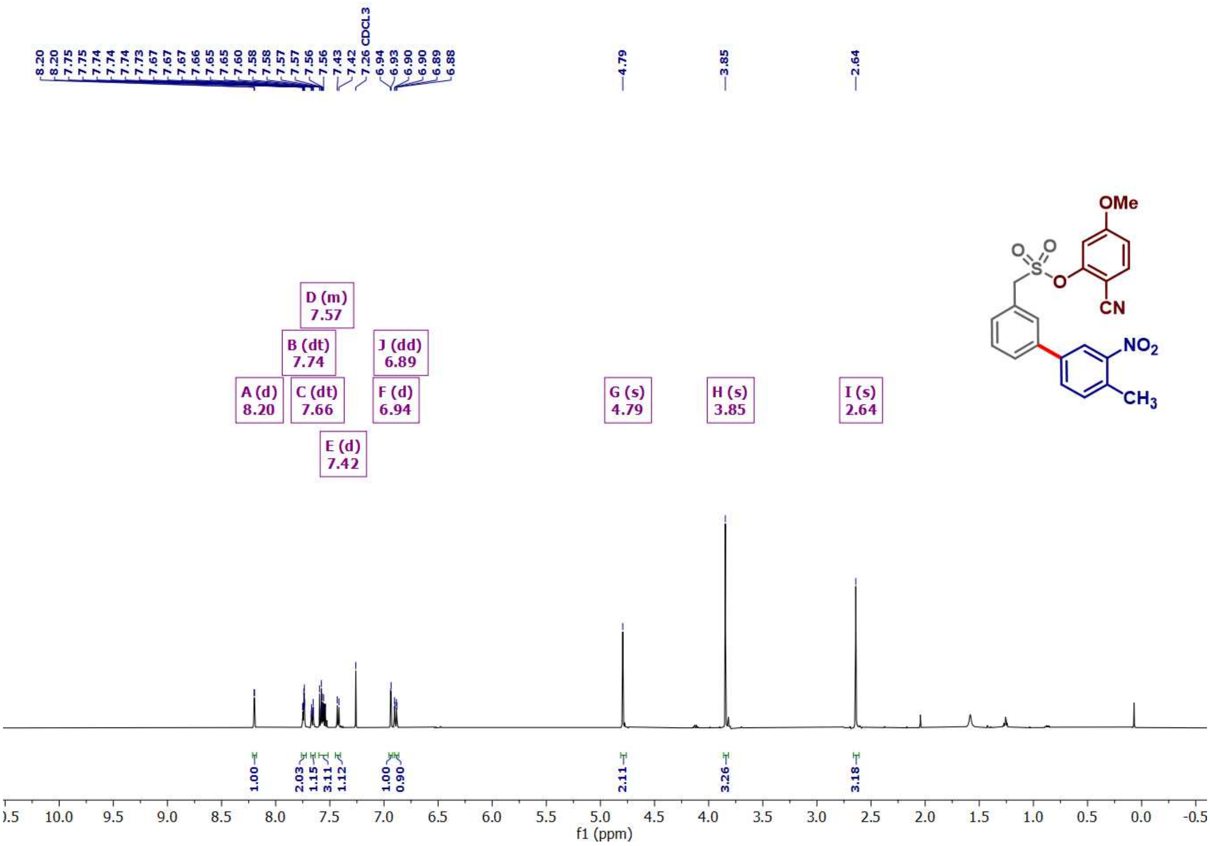
8.20 8.20 7.75 7.75 7.74 7.74 7.74 7.73 7.67 7.67 7.67 7.66 7.65 7.65 7.60 7.58 7.58 7.57 7.57 7.56 7.56 7.43 7.42 7.26 CDCL3 6.94 6.93 6.90 6.90 6.89 6.88
4.79
3.85
2.64
OMe
CN
NO2
CH3
D (m) 7.57
B (dt) 7.74
J (dd) 6.89
A (d) 8.20
C (dt) 7.66
F (d) 6.94
G (s) 4.79
H (s) 3.85
I (s) 2.64
E (d) 7.42
1.00
2.03 1.15 3.11 1.12
1.00 0.90
2.11
3.26
3.18
f1 (ppm)

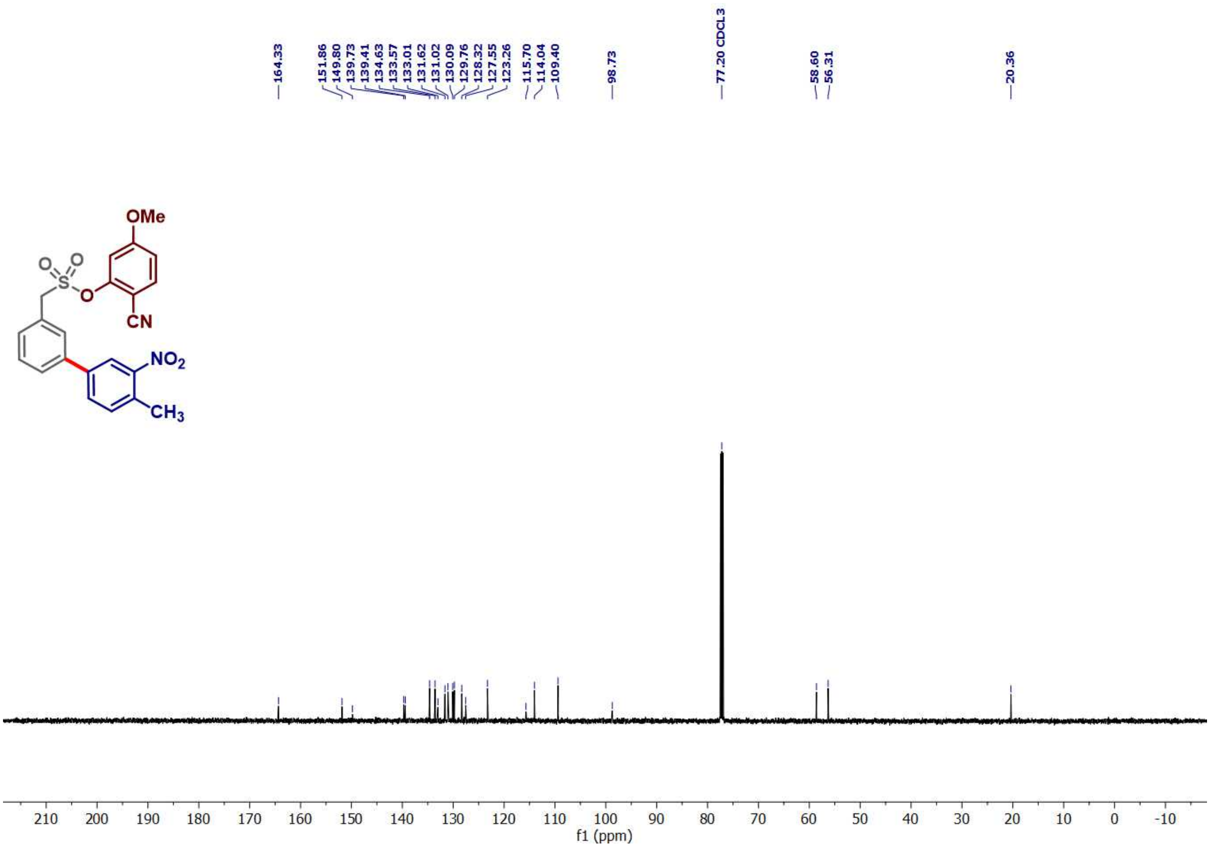
164.33
151.86 149.80 139.73 139.41 134.63 133.57 133.01 131.62 131.02 130.09 129.76 128.32 127.55 123.26 115.70 114.04 109.40
98.73
77.20 CDCL3
58.60 56.31
20.36
OMe
CN
NO2
CH3
f1 (ppm)

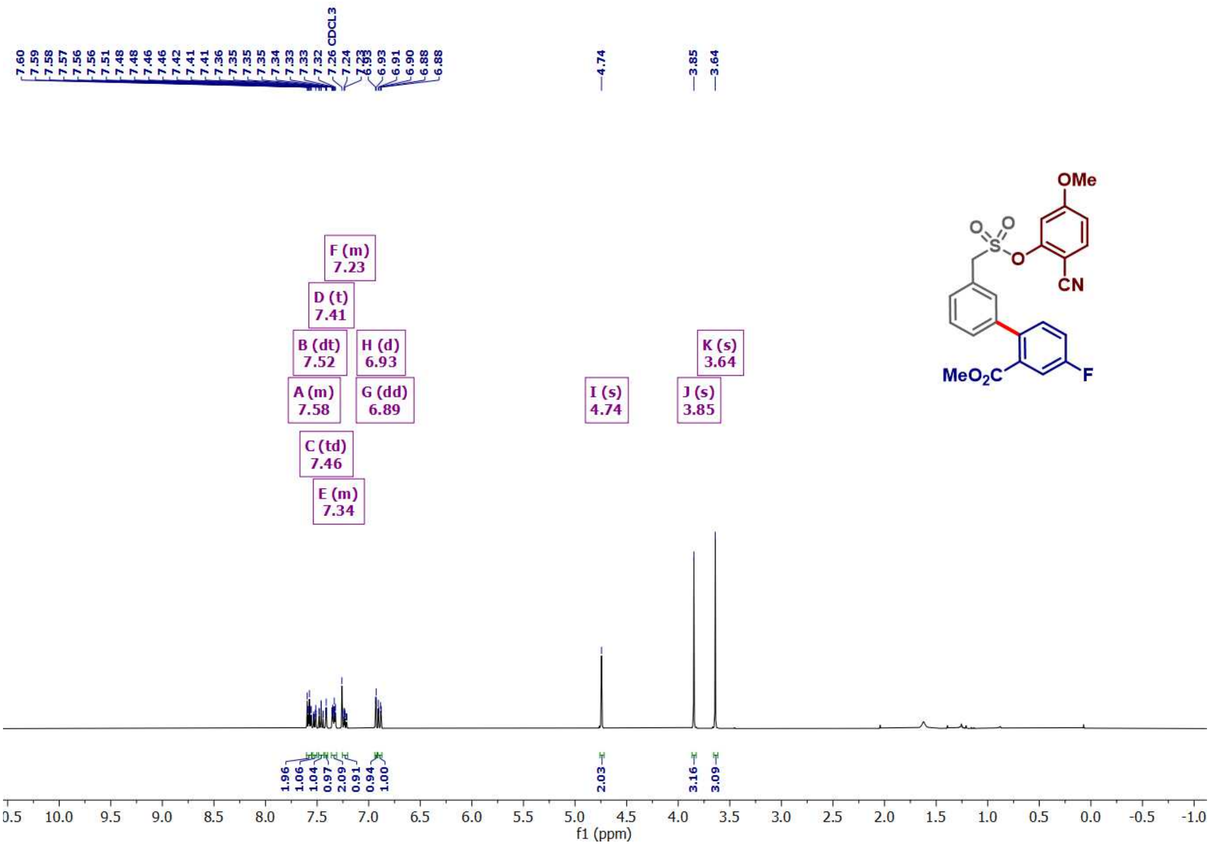
7.60 7.59 7.58 7.57 7.56 7.56 7.51 7.48 7.48 7.46 7.46 7.42 7.41 7.41 7.36 7.35 7.35 7.34 7.33 7.32 7.26 CDCL3 7.24 7.23 6.93 6.93 6.91 6.90 6.88 6.88
4.74
3.85
3.64
F (m) 7.23
D (t) 7.41
B (dt) 7.52
H (d) 6.93
A (m) 7.58
G (dd) 6.89
C (td) 7.46
E (m) 7.34
I (s) 4.74
J (s) 3.85
K (s) 3.64
OMe
CN
MeO2C
F
1.96 1.06 1.04 0.97 2.09 0.91 0.94 1.00
2.03
3.16
3.09
f1 (ppm)

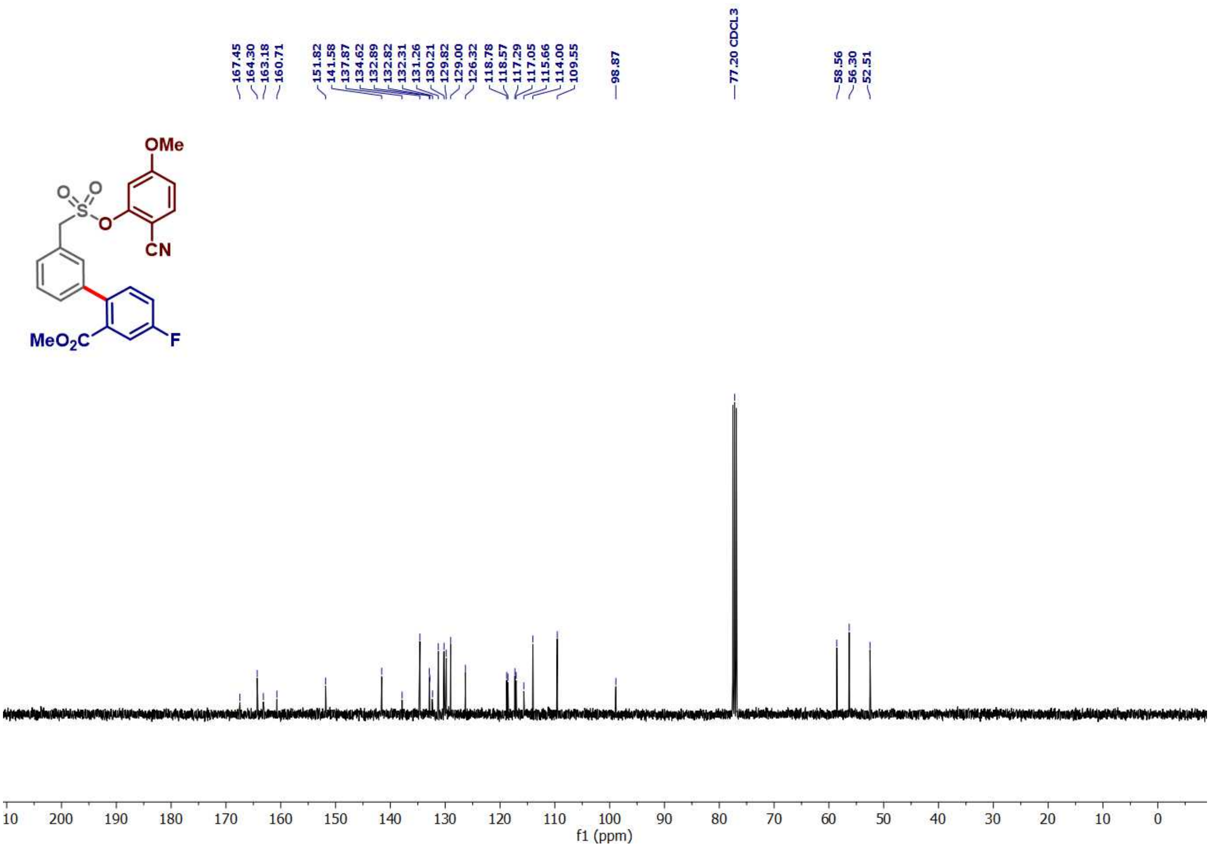
167.45 164.30 163.18 160.71
151.82 141.58 137.87 134.62 132.89 132.82 132.31 131.26 130.21 129.82 129.00 126.32 118.78 118.57 117.29 117.05 115.66 114.00 109.55
98.87
77.20 CDCL3
58.56 56.30 52.51
OMe
CN
MeO2C
F
f1 (ppm)

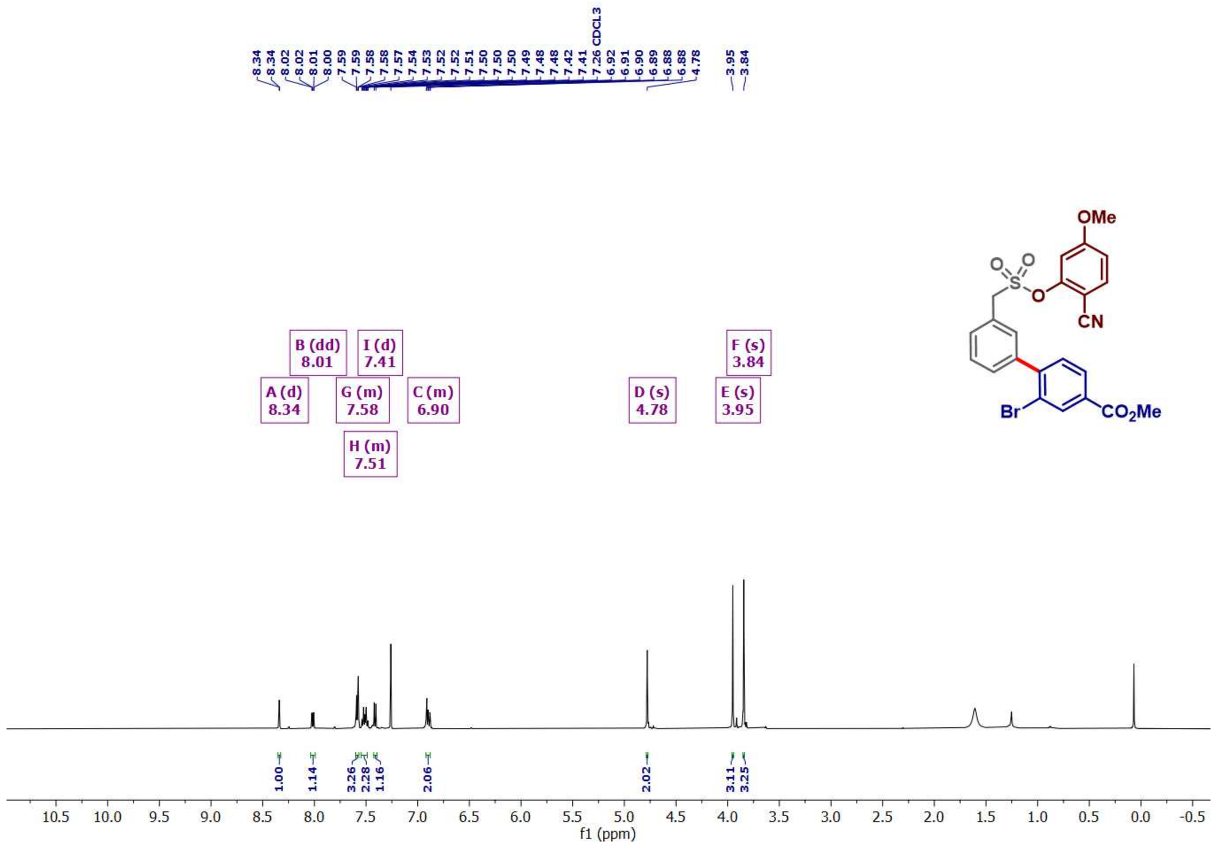

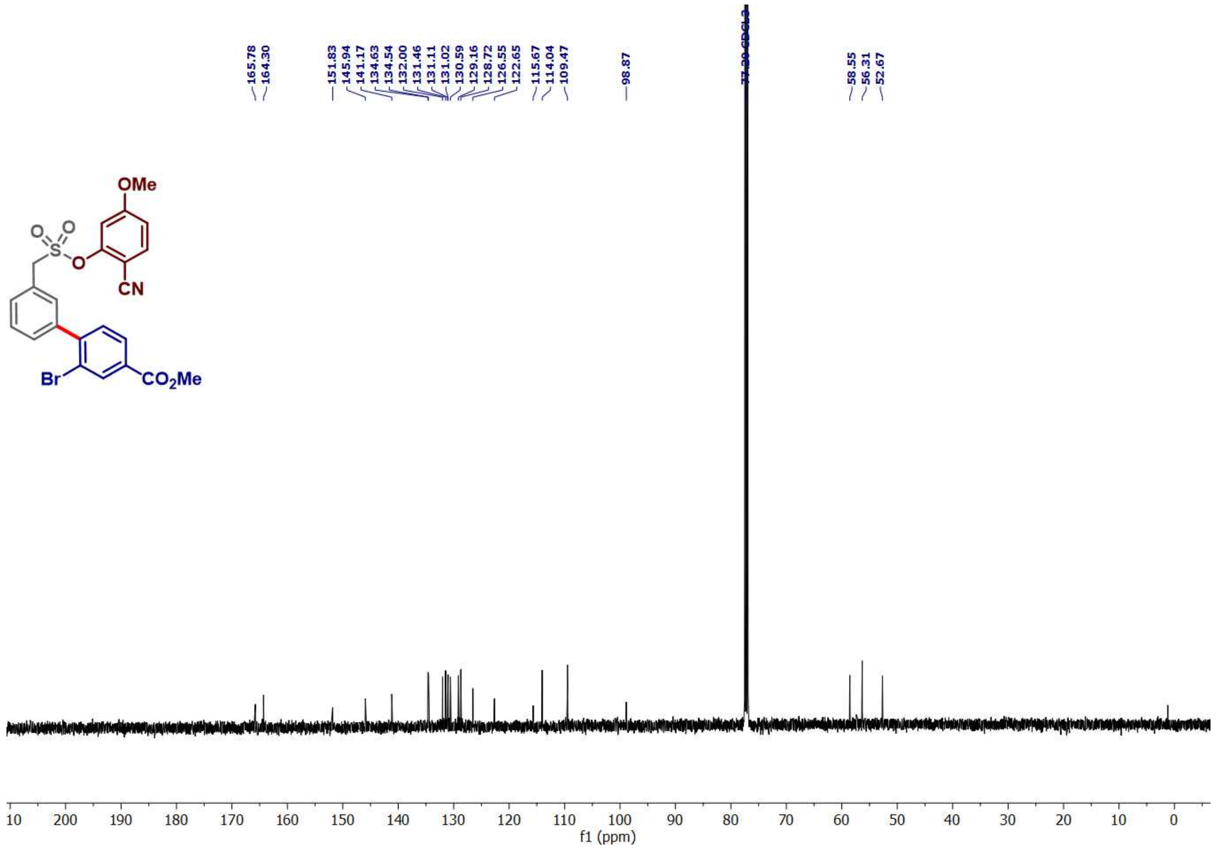

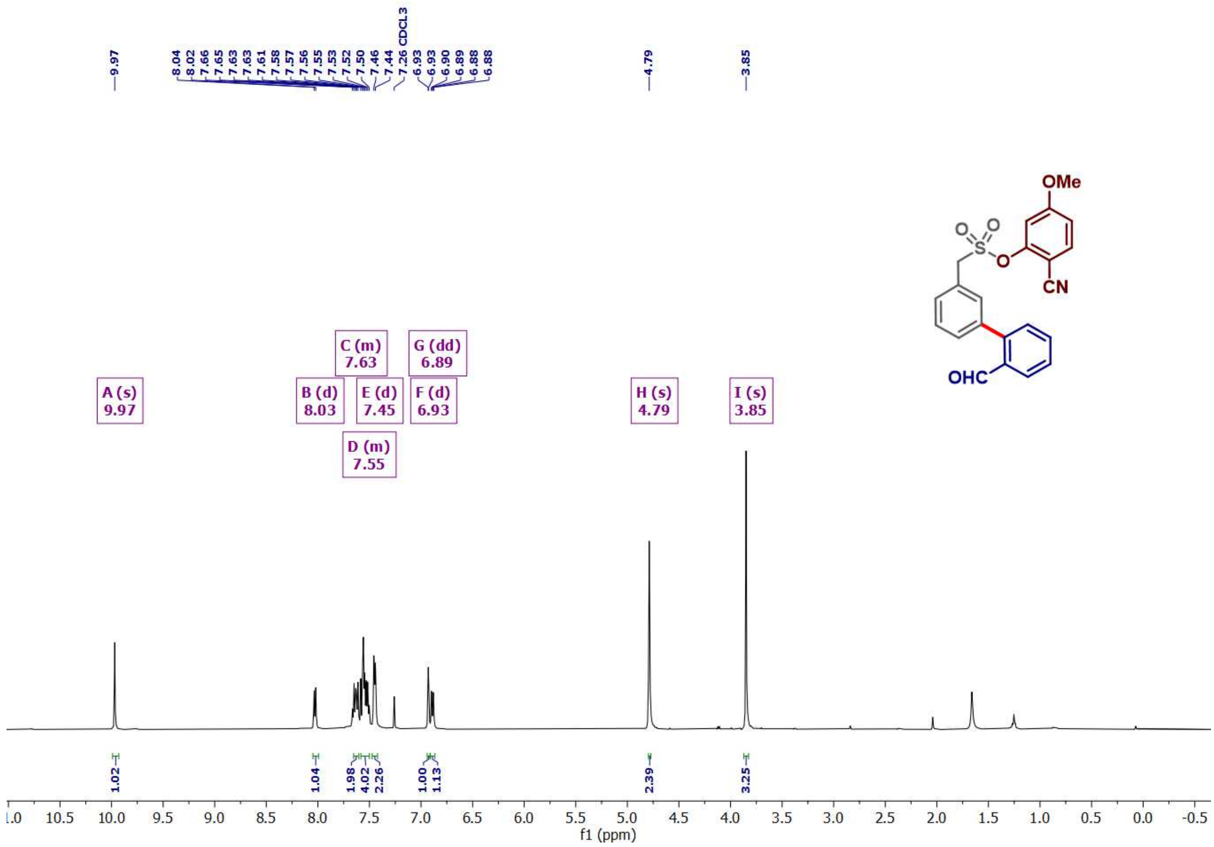

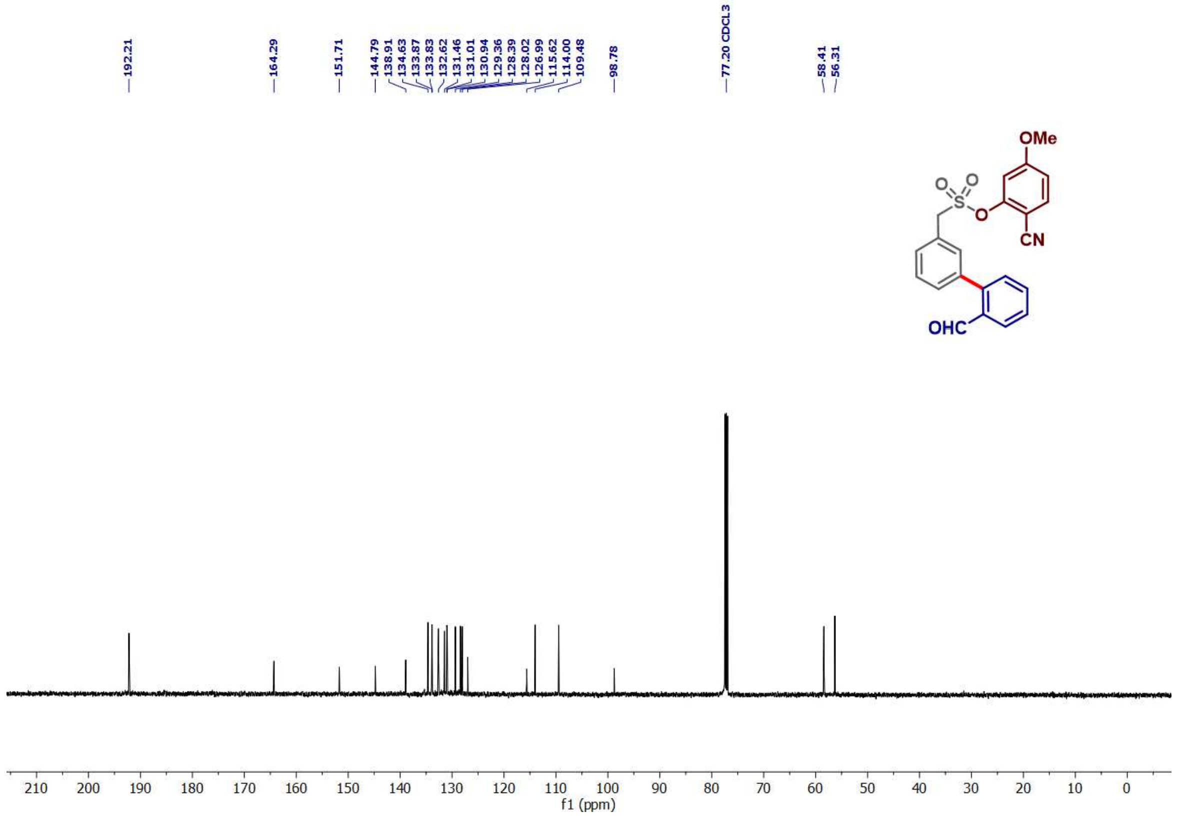

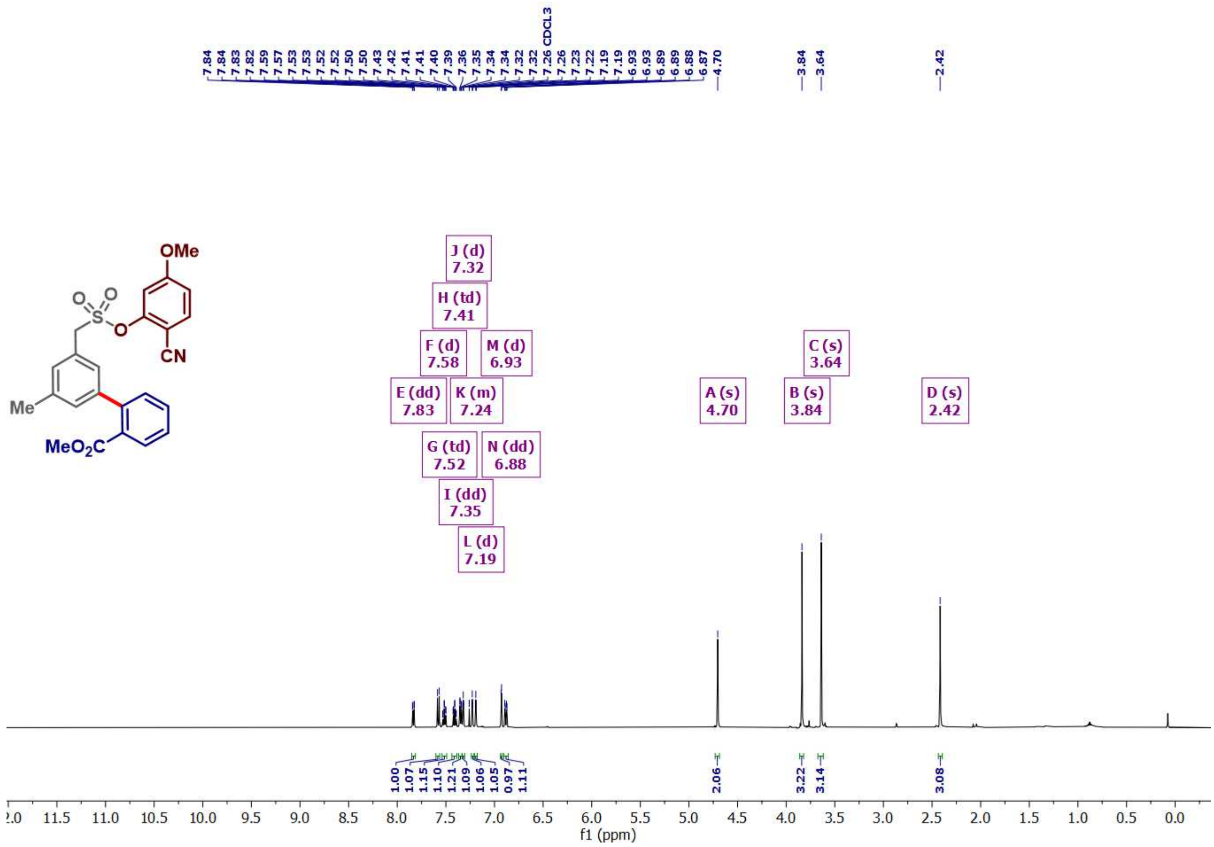
7.84
7.84
7.83
7.82
7.59
7.57
7.53
7.53
7.52
7.52
7.50
7.50
7.43
7.42
7.41
7.41
7.40
7.39
7.36
7.35
7.34
7.34
7.32
7.32
7.26 CDCL3
7.26
7.23
7.22
7.19
7.19
6.93
6.93
6.89
6.89
6.88
6.87
4.70
3.84
3.64
2.42
OMe
CN
Me
MeO2C
J (d) 7.32
H (td) 7.41
F (d) 7.58
M (d) 6.93
C (s) 3.64
E (dd) 7.83
K (m) 7.24
A (s) 4.70
B (s) 3.84
D (s) 2.42
G (td) 7.52
N (dd) 6.88
I (dd) 7.35
L (d) 7.19
1.00
1.07
1.15
1.10
1.21
1.09
1.06
1.05
0.97
1.11
2.06
3.22
3.14
3.08
f1 (ppm)

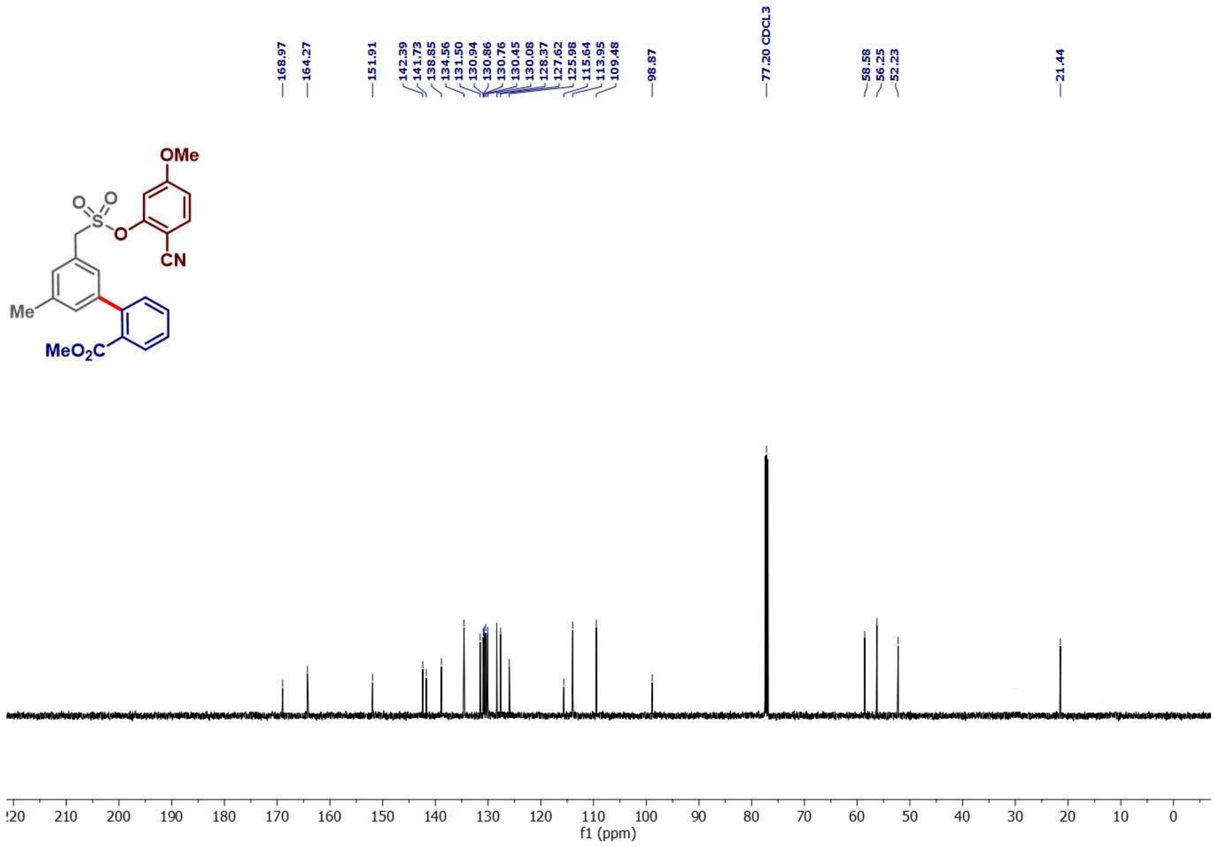
168.97
164.27
151.91
142.39
141.73
138.85
134.56
131.50
130.94
130.86
130.76
130.45
130.08
128.37
127.62
125.98
115.64
113.95
109.48
98.87
77.20 CDCL3
58.58
56.25
52.23
21.44
OMe
CN
Me
MeO2C
f1 (ppm)

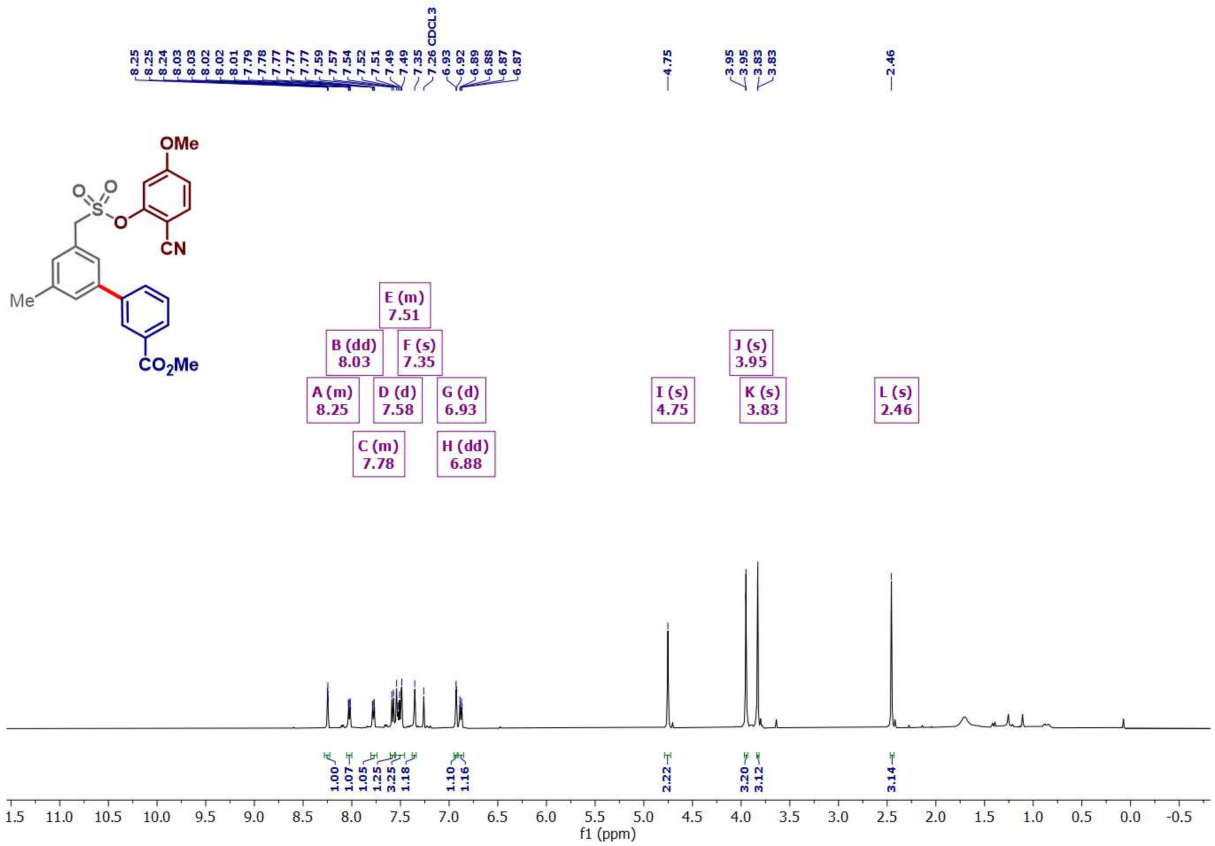

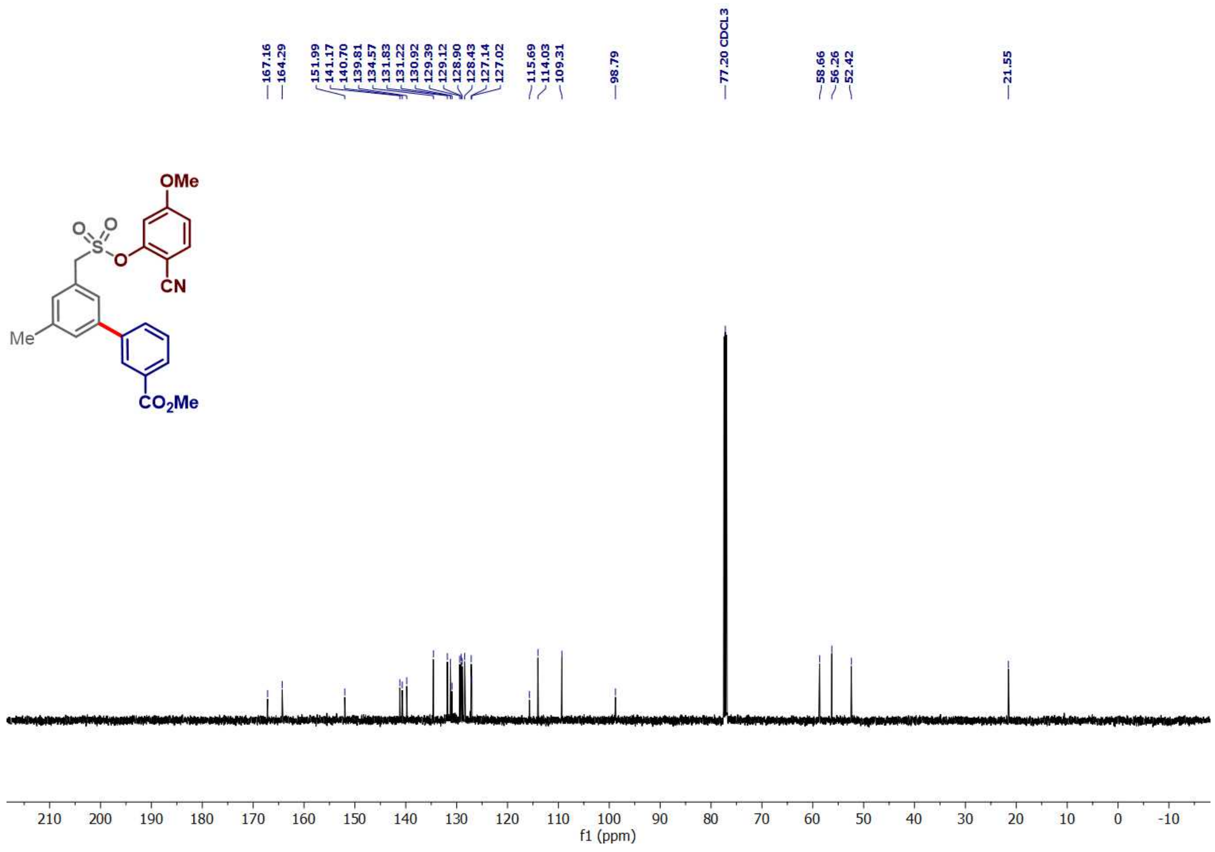

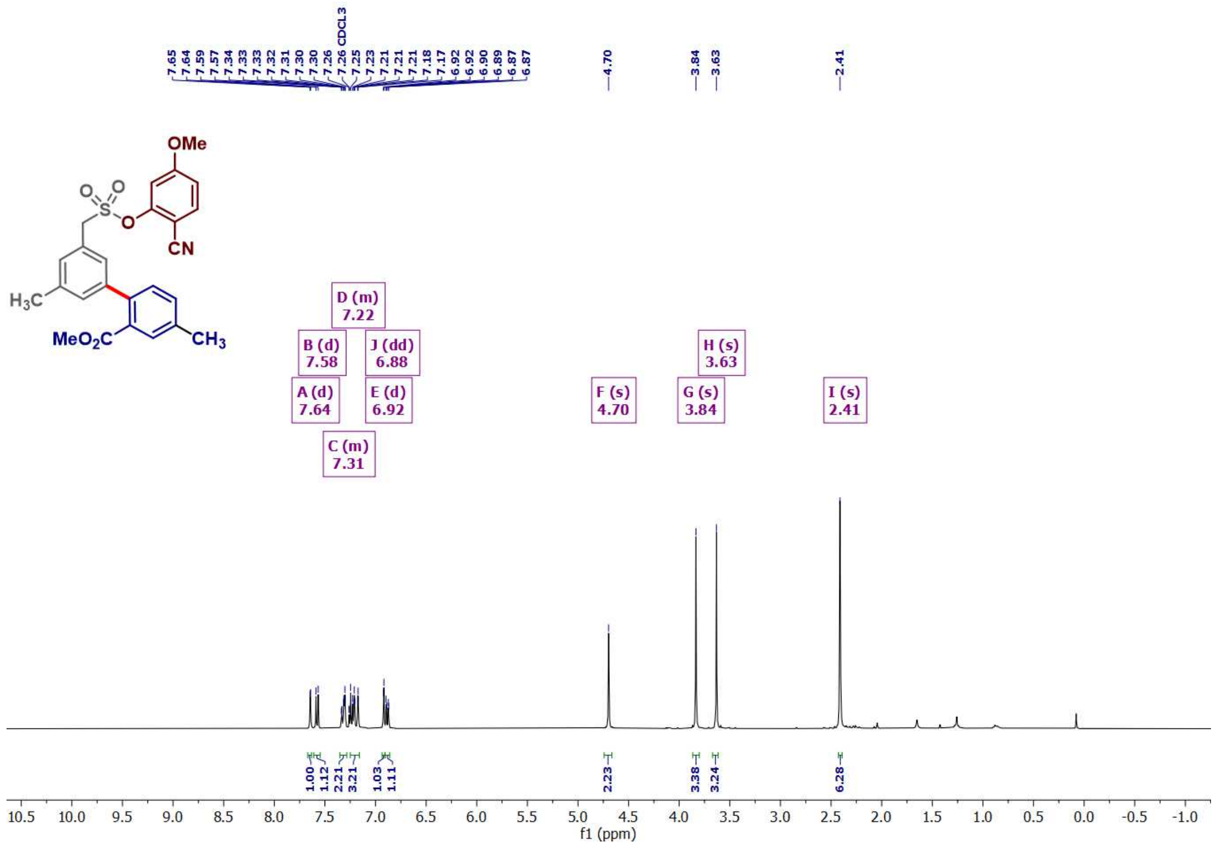
OMe
CN
H_3C
MeO_2C
CH_3
D (m) 7.22
B (d) 7.58
J (dd) 6.88
A (d) 7.64
E (d) 6.92
C (m) 7.31
F (s) 4.70
G (s) 3.84
H (s) 3.63
I (s) 2.41
f1 (ppm)

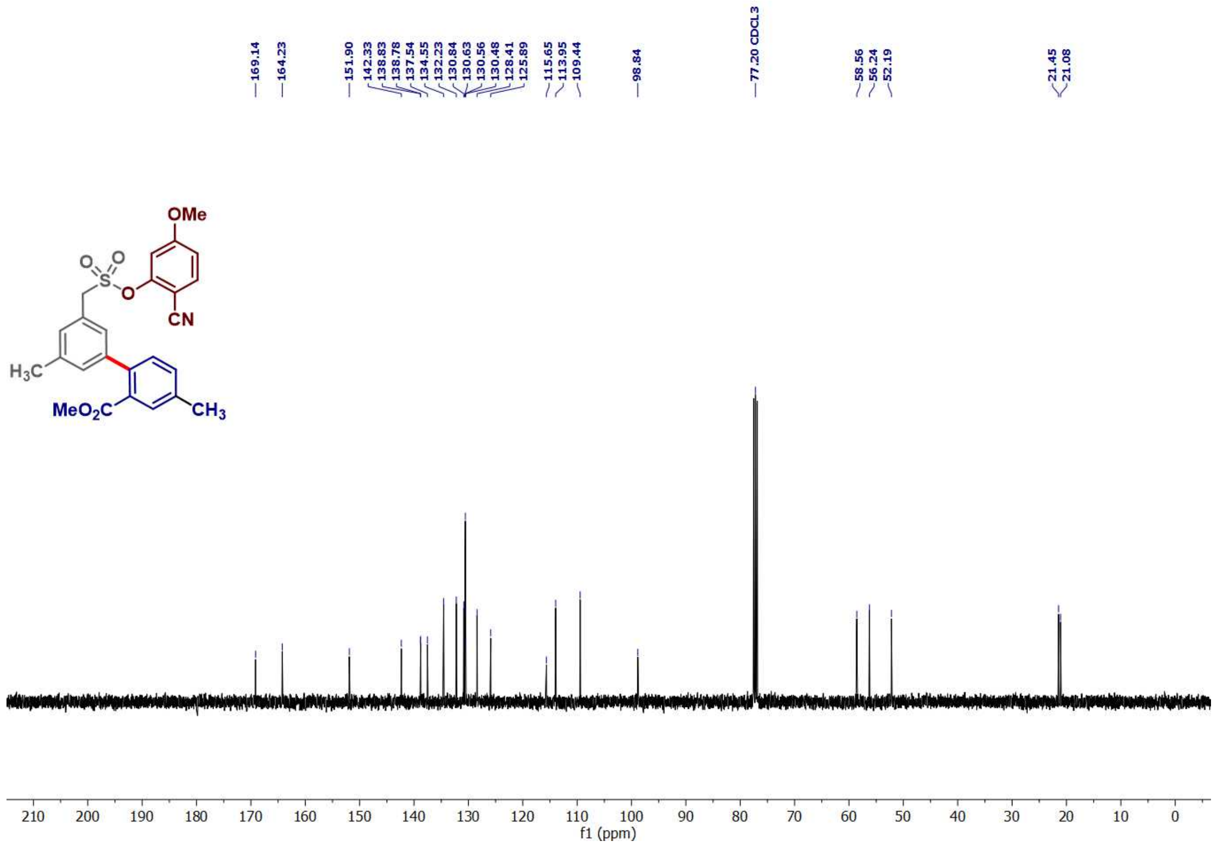
169.14
164.23
151.90
77.20 CDCL3
98.84
OMe
CN
H_3C
MeO_2C
CH_3
f1 (ppm)

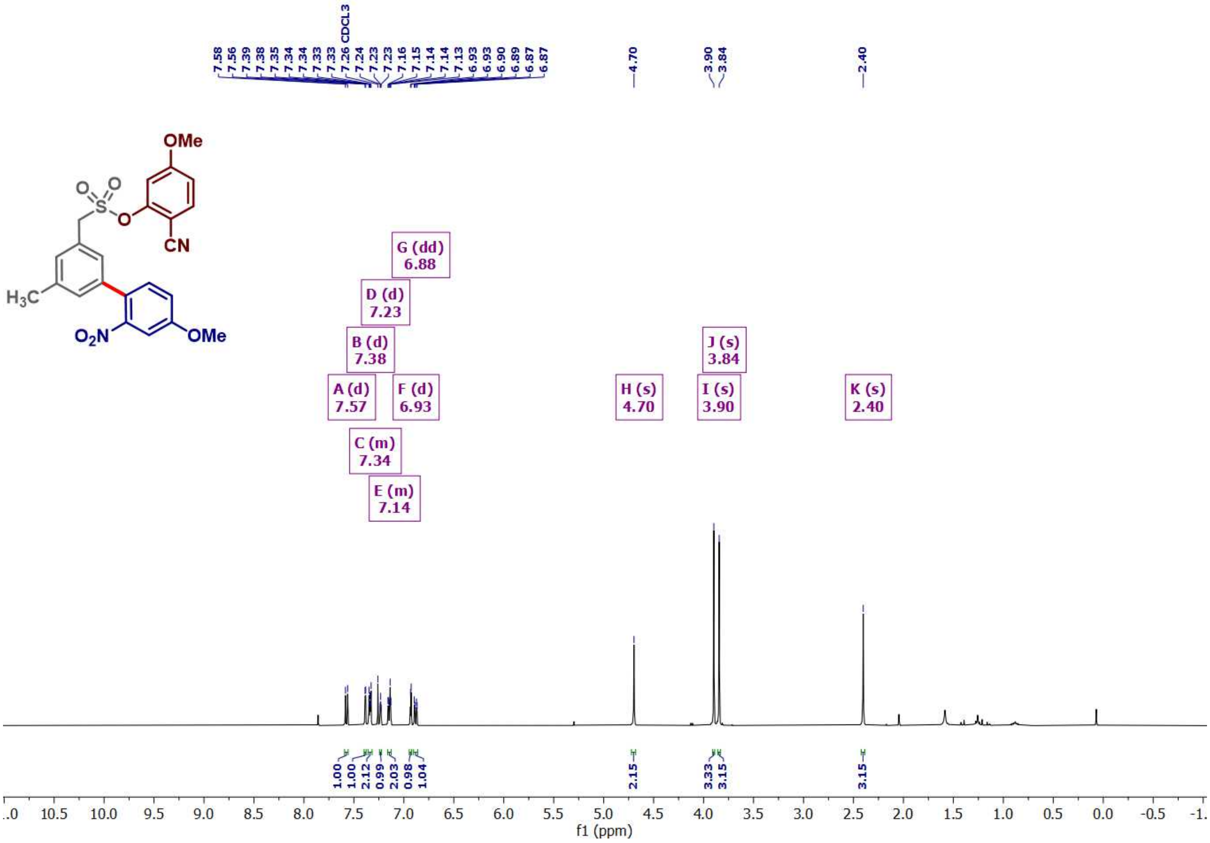
7.58 7.56 7.39 7.38 7.35 7.34 7.34 7.33 7.33 7.26 CDCL3 7.24 7.23 7.23 7.23 7.16 7.15 7.14 7.14 7.13 6.93 6.93 6.90 6.89 6.87 6.87
4.70
3.90 3.84
2.40
OMe
CN
H_3C
O_2N
OMe
G (dd) 6.88
D (d) 7.23
B (d) 7.38
A (d) 7.57
F (d) 6.93
C (m) 7.34
E (m) 7.14
H (s) 4.70
J (s) 3.84
I (s) 3.90
K (s) 2.40
1.00 1.00 2.12 0.99 2.03 0.98 1.04
2.15
3.33 3.15
3.15
f1 (ppm)

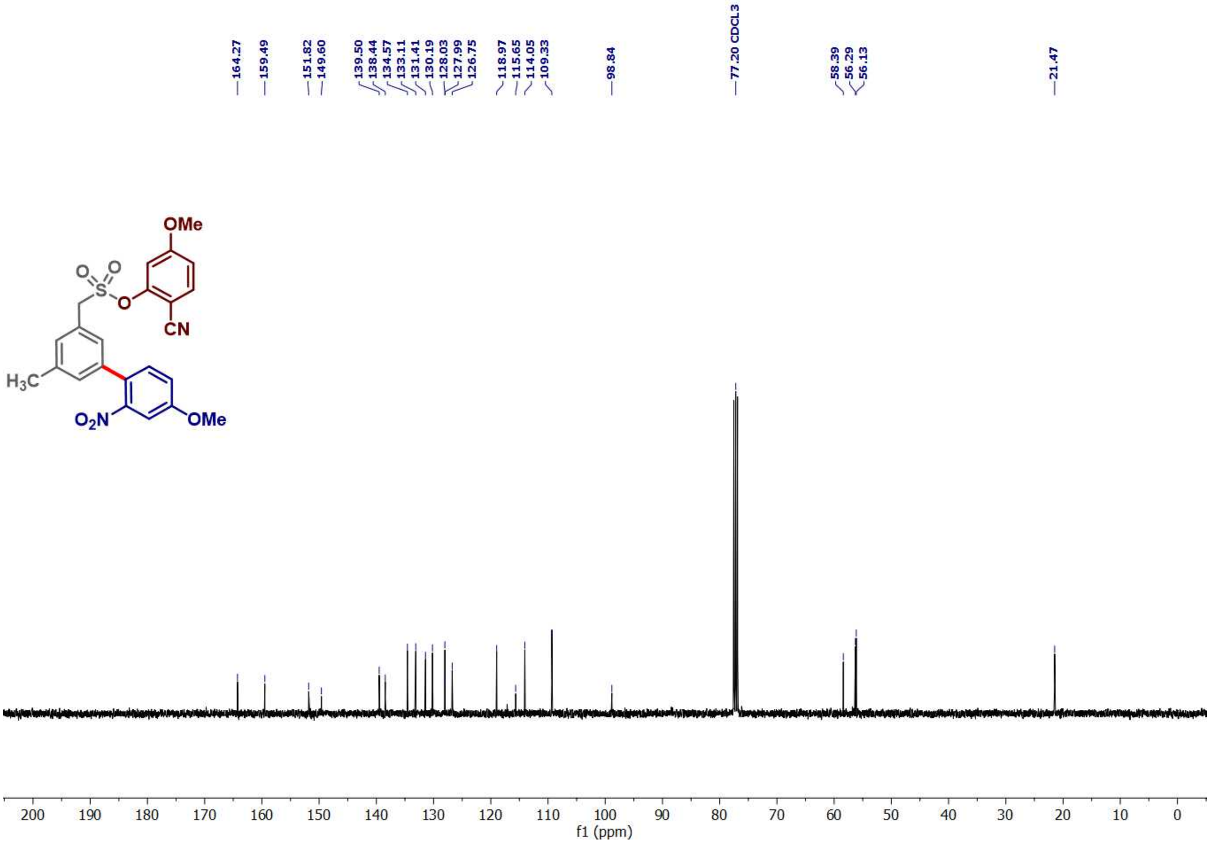
164.27
159.49
151.82 149.60
139.50 138.44 134.57 133.11 131.41 130.19 128.03 127.99 126.75
118.97 115.65 114.05 109.33
98.84
77.20 CDCL3
58.39 56.29 56.13
21.47
OMe
CN
H_3C
O_2N
OMe
f1 (ppm)

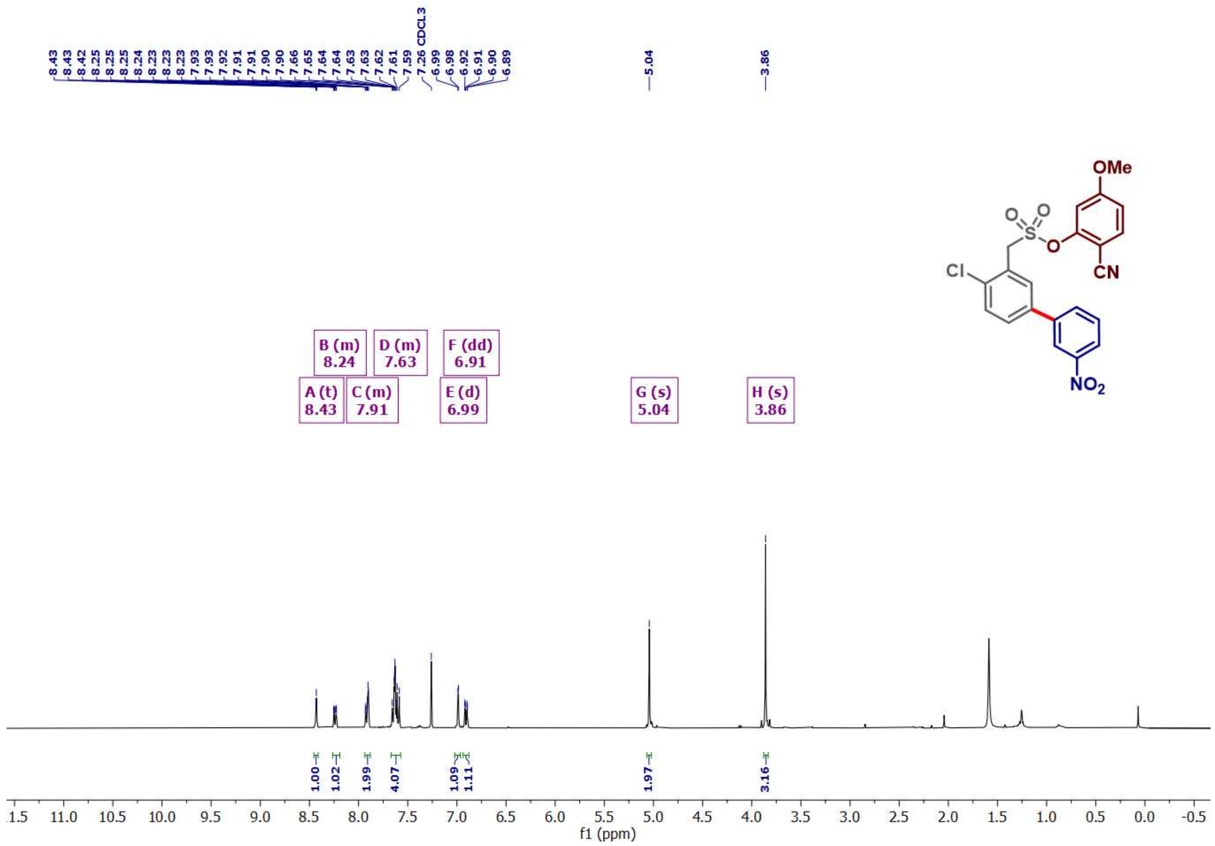
8.43 8.43 8.42 8.25 8.25 8.25 8.24 8.23 8.23 8.23 7.93 7.93 7.92 7.91 7.91 7.90 7.66 7.65 7.64 7.64 7.63 7.63 7.62 7.61 7.59 7.26 CDCL3 6.99 6.98 6.92 6.91 6.90 6.89
5.04
3.86
OMe
O
O
S
O
CN
Cl
NO2
B (m) 8.24
D (m) 7.63
F (dd) 6.91
A (t) 8.43
C (m) 7.91
E (d) 6.99
G (s) 5.04
H (s) 3.86
1.00 1.02 1.99 4.07 1.09 1.11 1.97 3.16
f1 (ppm)

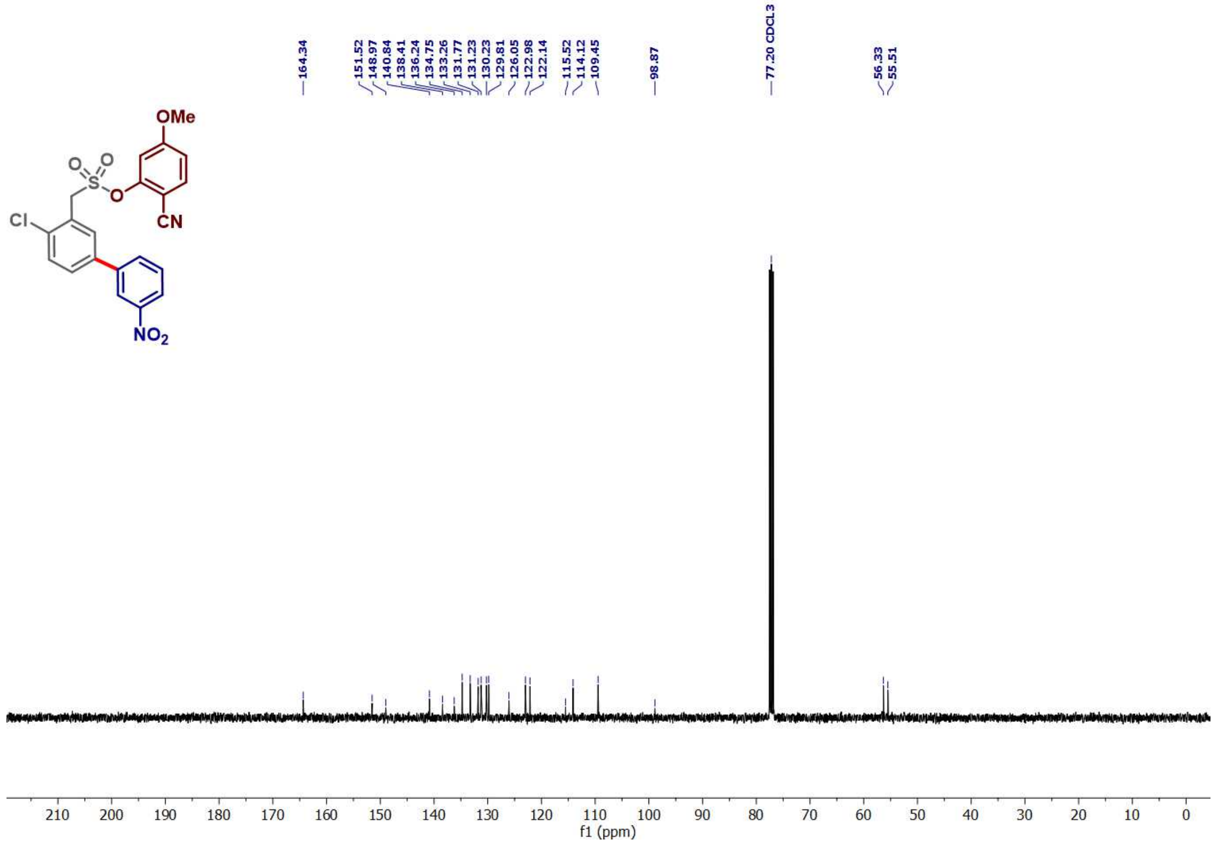
164.34
151.52 148.97 140.84 138.41 136.24 134.75 133.26 131.77 131.23 130.23 129.81 126.05 122.98 122.14 115.52 114.12 109.45
98.87
77.20 CDCL3
56.33 55.51
OMe
O
O
S
O
CN
Cl
NO2
f1 (ppm)

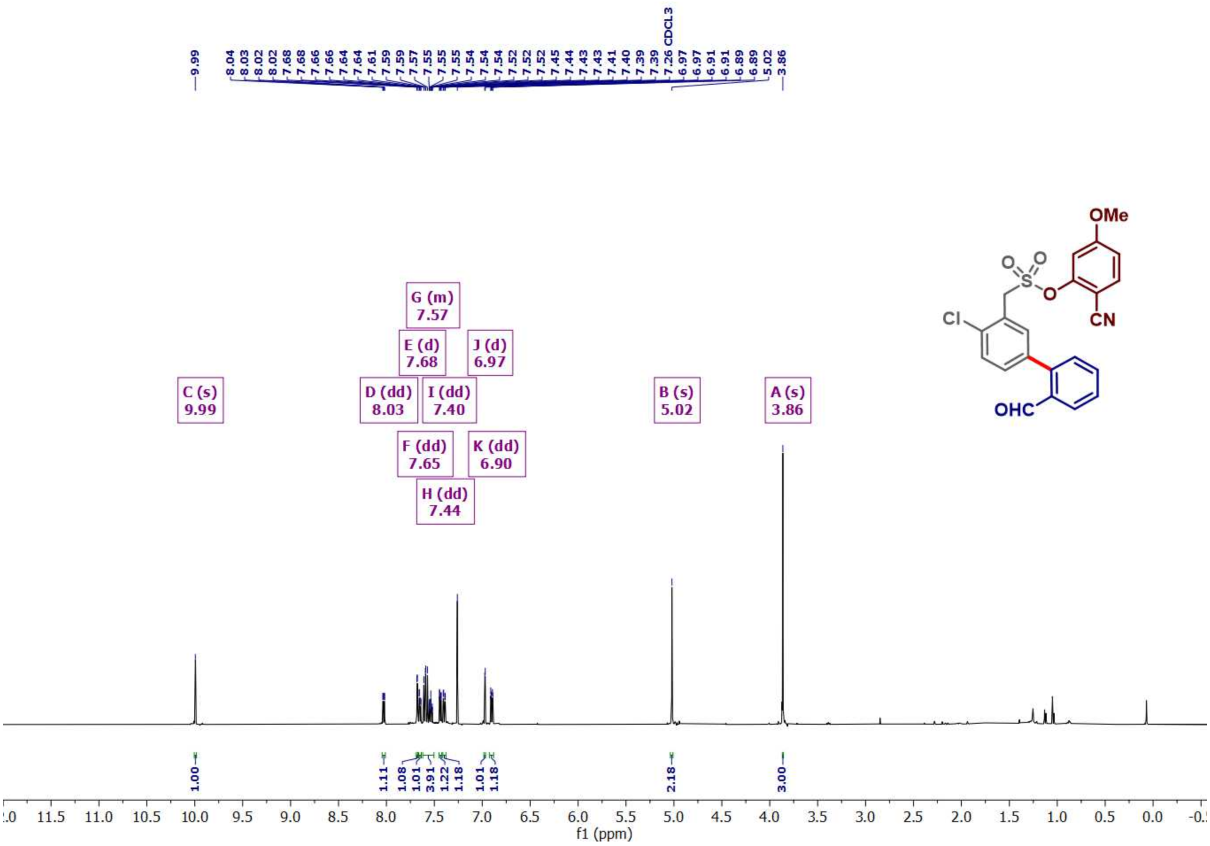
9.99
8.04 8.03 8.02 8.02 7.68 7.66 7.66 7.64 7.64 7.61 7.59 7.59 7.57 7.55 7.55 7.55 7.54 7.54 7.54 7.52 7.52 7.52 7.45 7.44 7.43 7.43 7.41 7.40 7.39 7.39 7.26 CDCL3 6.97 6.97 6.91 6.91 6.89 5.02 3.86
G (m) 7.57
E (d) 7.68
J (d) 6.97
C (s) 9.99
D (dd) 8.03
I (dd) 7.40
B (s) 5.02
A (s) 3.86
F (dd) 7.65
K (dd) 6.90
H (dd) 7.44
OMe
CN
Cl
OHC
1.00
1.11 1.08 1.01 3.91 1.22 1.18 1.01 1.18
2.18
3.00
f1 (ppm)

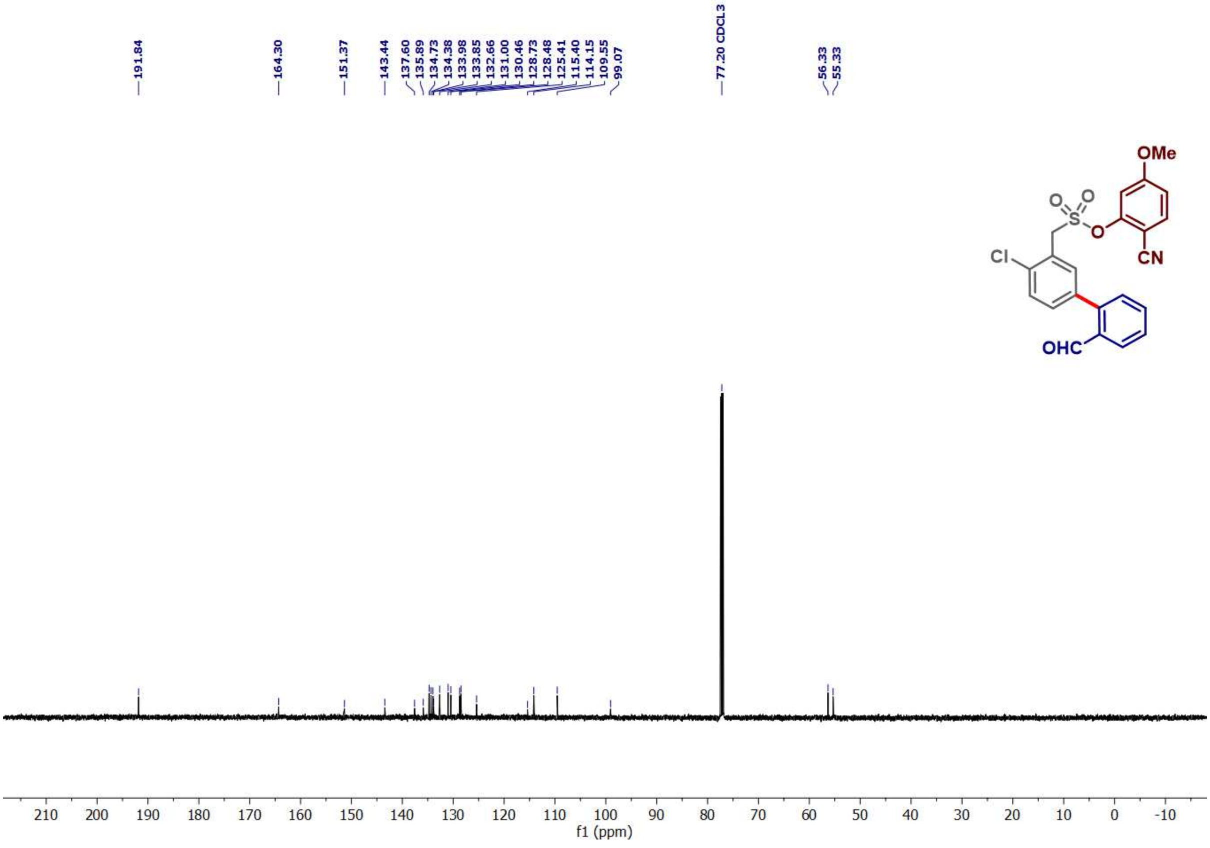
191.84
164.30
151.37
143.44
137.60 135.89 134.73 134.38 133.98 133.85 132.66 131.00 130.46 128.73 128.48 125.41 115.40 114.15 109.55 99.07
77.20 CDCL3
56.33 55.33
OMe
CN
Cl
OHC
f1 (ppm)

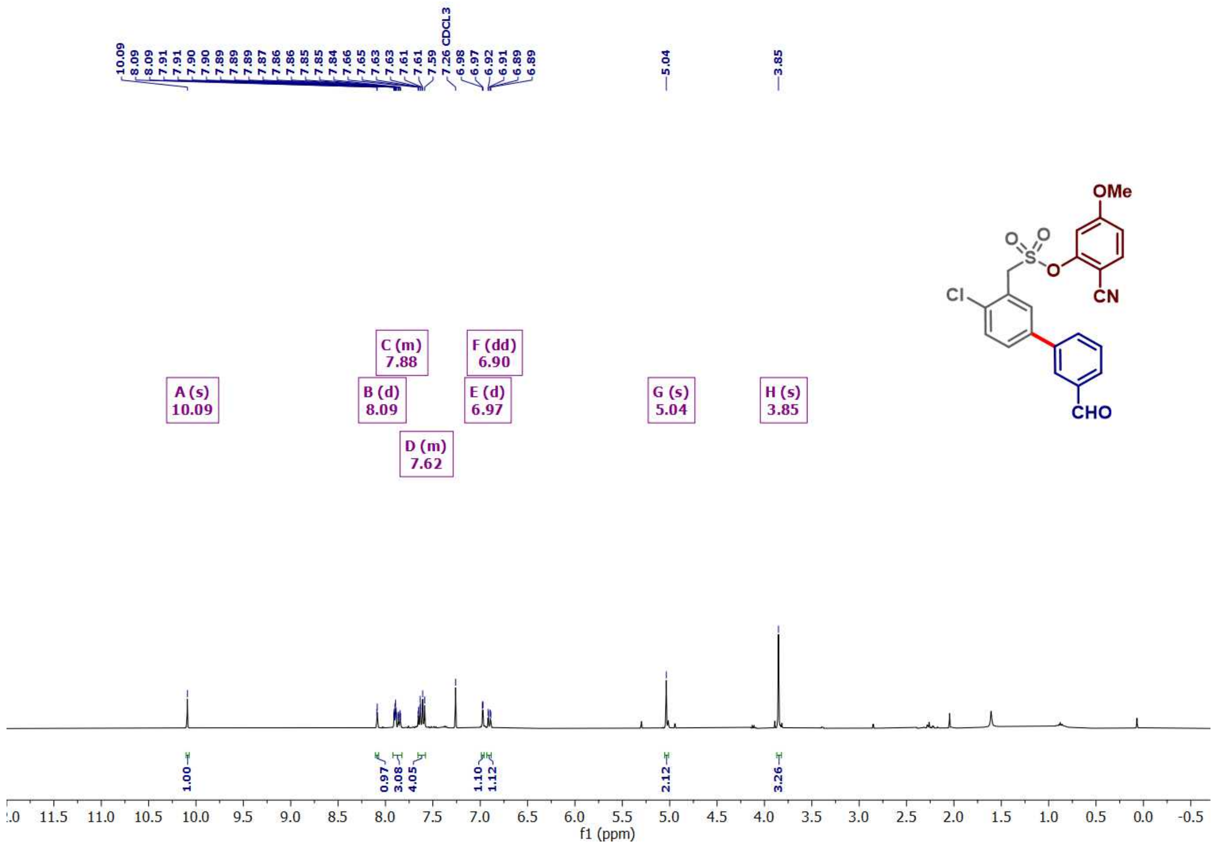

10.09
8.09
8.09
7.91
7.91
7.90
7.90
7.89
7.89
7.89
7.87
7.86
7.86
7.85
7.85
7.84
7.66
7.65
7.63
7.63
7.61
7.61
7.59
7.26 CDCL3
6.98
6.97
6.92
6.91
6.89
6.89
5.04
3.85
OMe
O
O
S
O
Cl
CN
CHO
C (m)
7.88
F (dd)
6.90
A (s)
10.09
B (d)
8.09
E (d)
6.97
G (s)
5.04
H (s)
3.85
D (m)
7.62
1.00
0.97
3.08
4.05
1.10
1.12
2.12
3.26
f1 (ppm)

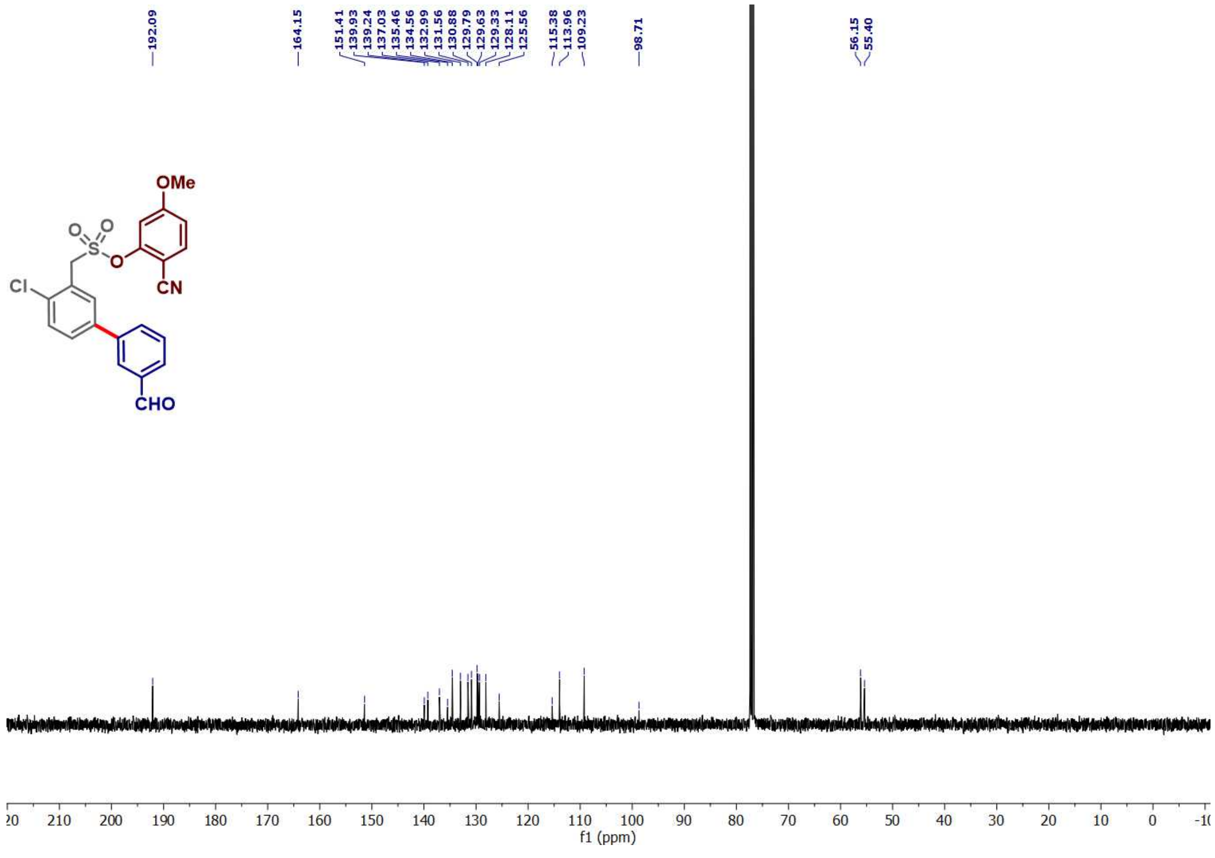

192.09
164.15
151.41
139.93
139.24
137.03
135.46
134.56
132.99
131.56
130.88
129.79
129.63
129.33
128.11
125.56
115.38
113.96
109.23
98.71
56.15
55.40
OMe
O
O
S
O
Cl
CN
CHO
f1 (ppm)

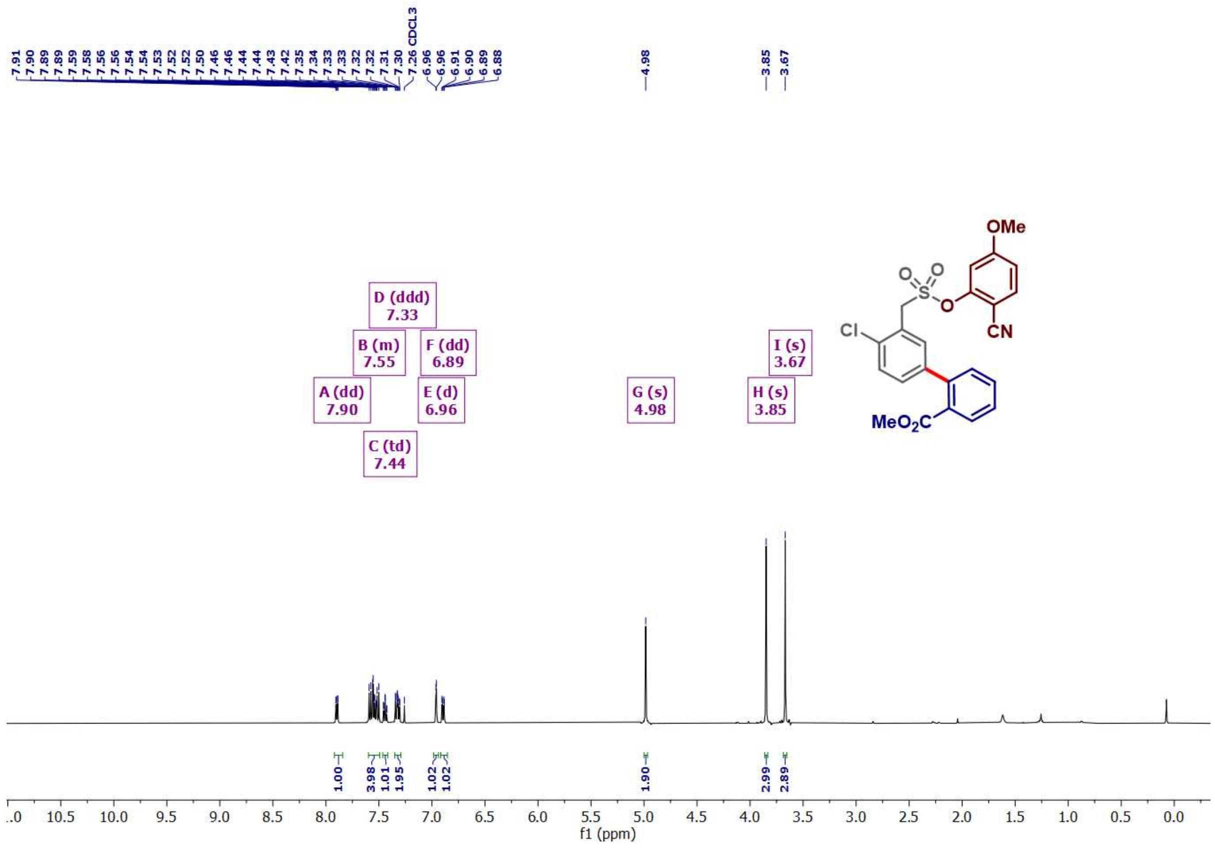
7.91 7.90 7.89 7.89 7.59 7.58 7.56 7.56 7.54 7.54 7.53 7.52 7.52 7.50 7.46 7.46 7.44 7.43 7.42 7.35 7.34 7.33 7.33 7.32 7.32 7.31 7.30 7.26 CDCL3 6.96 6.96 6.91 6.90 6.89 6.88
4.98
3.85 3.67
D (ddd) 7.33
B (m) 7.55
F (dd) 6.89
A (dd) 7.90
E (d) 6.96
C (td) 7.44
G (s) 4.98
I (s) 3.67
H (s) 3.85
OMe
CN
Cl
MeO2C
1.00 3.98 1.01 1.95 1.02 1.02 1.90 2.99 2.89
f1 (ppm)

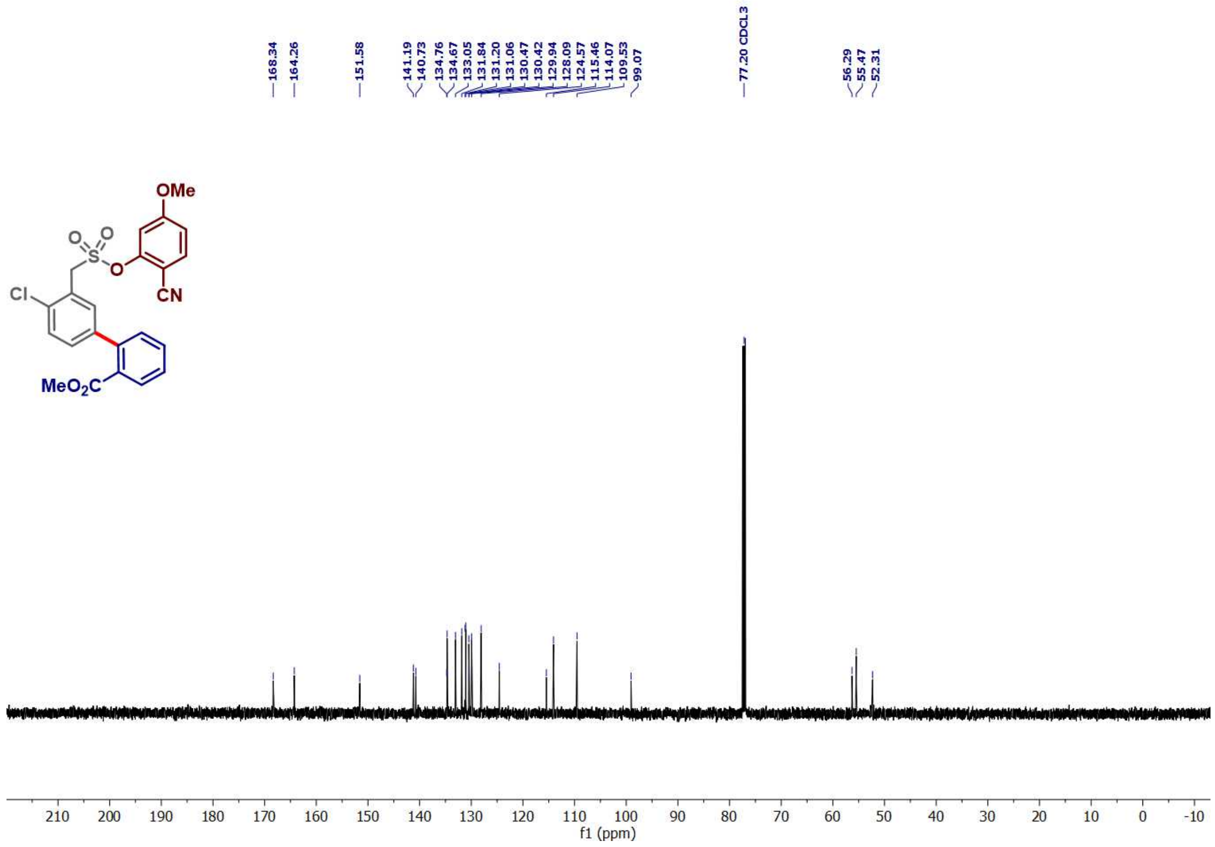
168.34 164.26 151.58 141.19 140.73 134.76 134.67 133.05 131.84 131.20 131.06 130.47 130.42 129.94 128.09 124.57 115.46 114.07 109.53 99.07
77.20 CDCL3
56.29 55.47 52.31
OMe
CN
Cl
MeO2C
f1 (ppm)

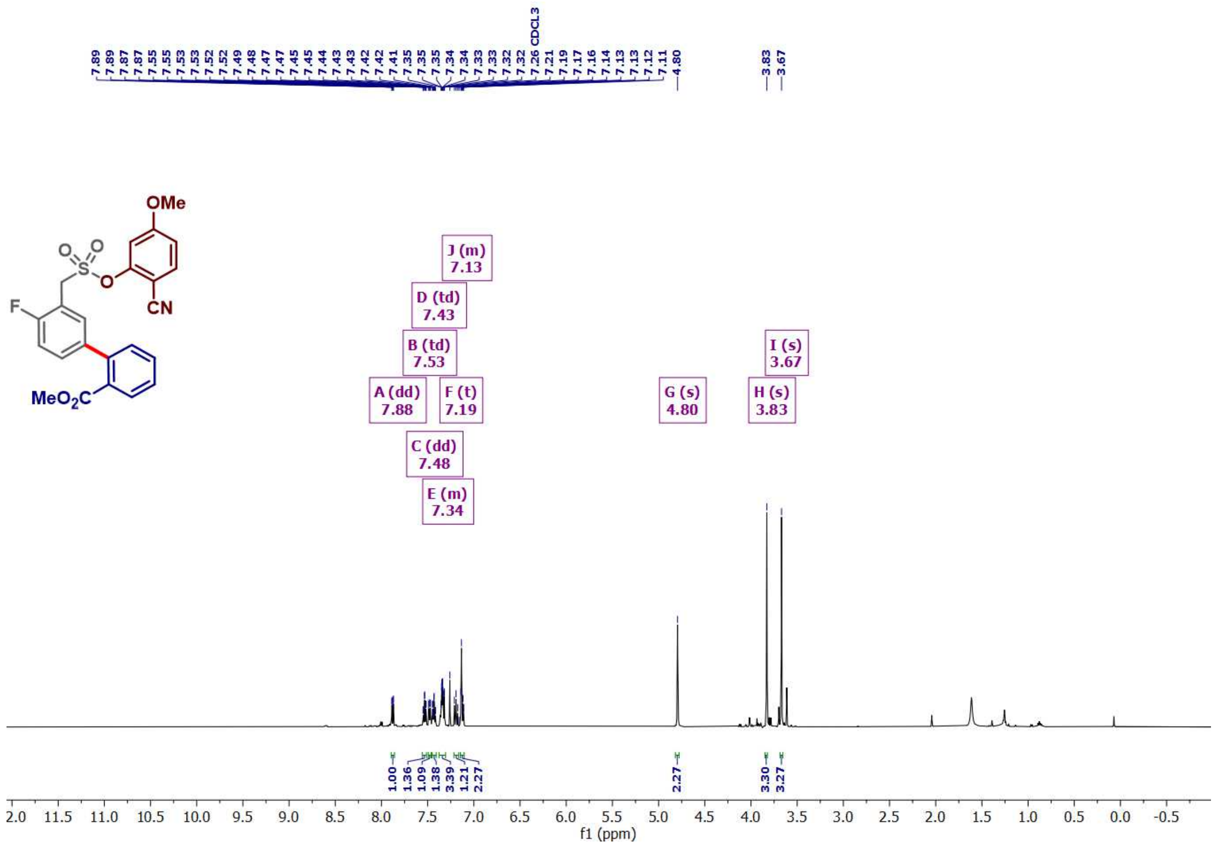
7.89 7.89 7.87 7.87 7.55 7.53 7.53 7.52 7.52 7.49 7.48 7.47 7.45 7.45 7.44 7.43 7.43 7.42 7.42 7.41 7.35 7.35 7.35 7.34 7.34 7.33 7.33 7.33 7.32 7.26 CDCL3 7.21 7.19 7.17 7.16 7.14 7.13 7.13 7.12 7.11 4.80 3.83 3.67
OMe
F
CN
MeO2C
J (m) 7.13
D (td) 7.43
B (td) 7.53
A (dd) 7.88
F (t) 7.19
C (dd) 7.48
E (m) 7.34
G (s) 4.80
I (s) 3.67
H (s) 3.83
1.00 1.36 1.09 1.38 3.39 1.21 2.27 2.27 3.30 3.27
f1 (ppm)

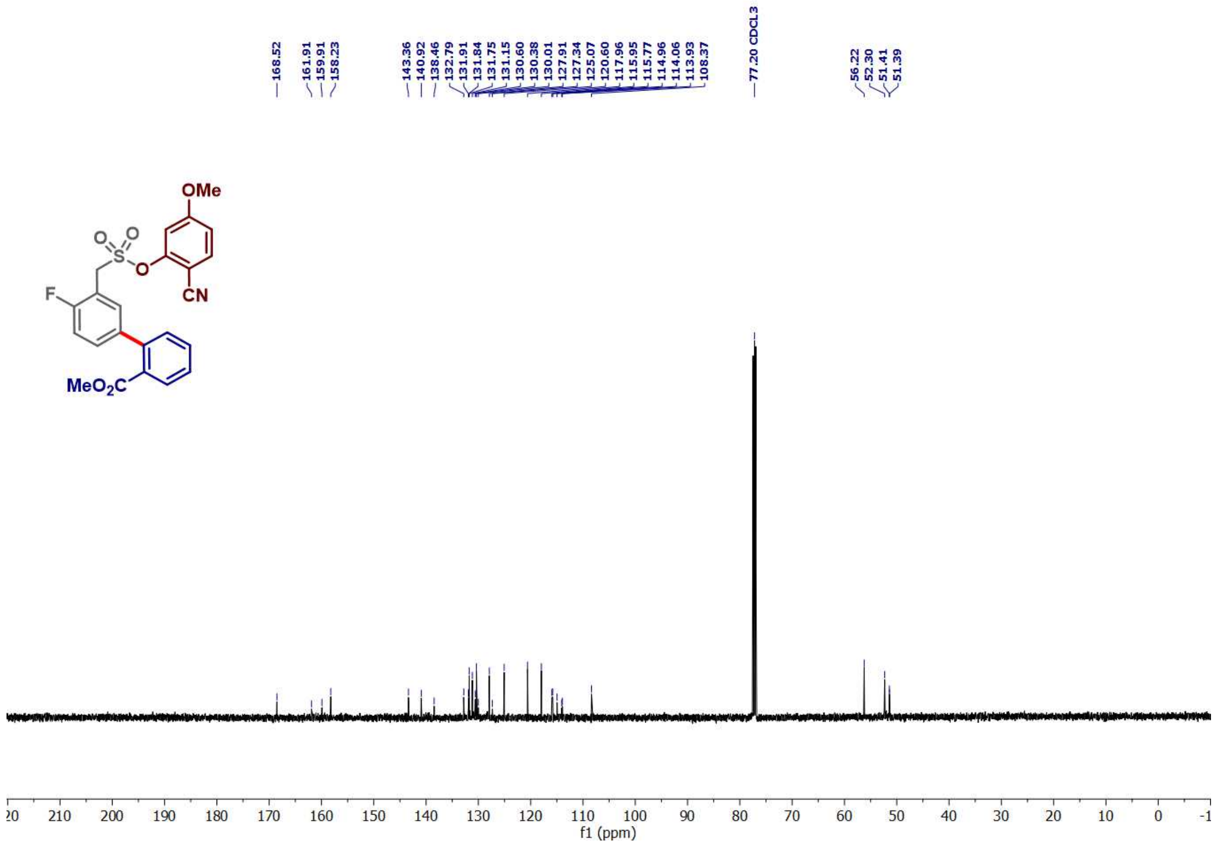
168.52 161.91 159.91 158.23 143.36 140.92 138.46 132.79 131.91 131.84 131.75 131.15 130.60 130.38 130.01 127.91 127.34 125.07 120.60 117.96 115.95 115.77 114.96 114.06 113.93 108.37 77.20 CDCL3 56.22 52.30 51.41 51.39
OMe
F
CN
MeO2C
f1 (ppm)

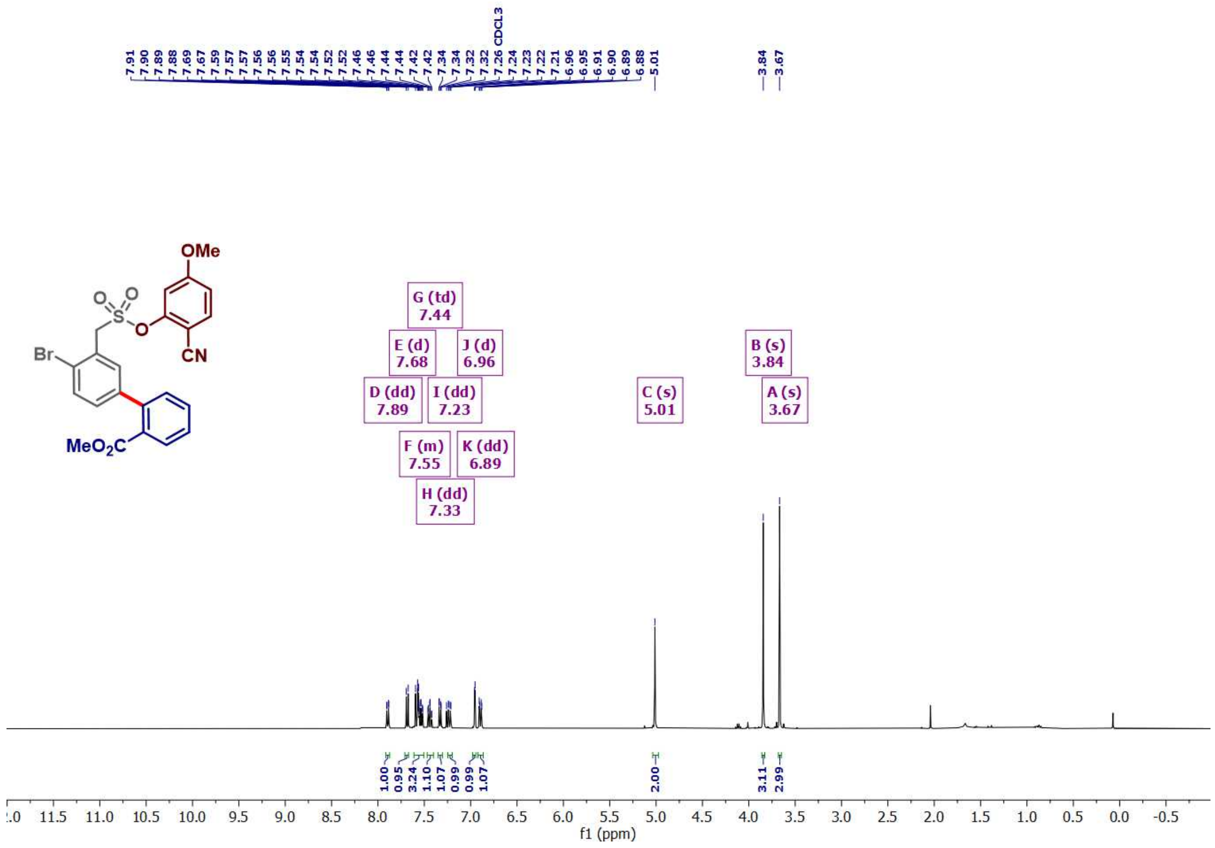

7.91
7.90
7.89
7.88
7.69
7.67
7.59
7.57
7.57
7.56
7.56
7.55
7.54
7.54
7.54
7.52
7.52
7.46
7.46
7.44
7.44
7.42
7.42
7.34
7.34
7.32
7.32
7.26 CDCL3
7.24
7.24
7.23
7.22
7.21
6.96
6.95
6.91
6.90
6.89
6.88
5.01
3.84
3.67
OMe
CN
Br
MeO_2C
G (td) 7.44
E (d) 7.68
J (d) 6.96
B (s) 3.84
D (dd) 7.89
I (dd) 7.23
C (s) 5.01
A (s) 3.67
F (m) 7.55
K (dd) 6.89
H (dd) 7.33
1.00
0.95
3.24
1.10
1.07
0.99
0.99
1.07
2.00
3.11
2.99
f1 (ppm)

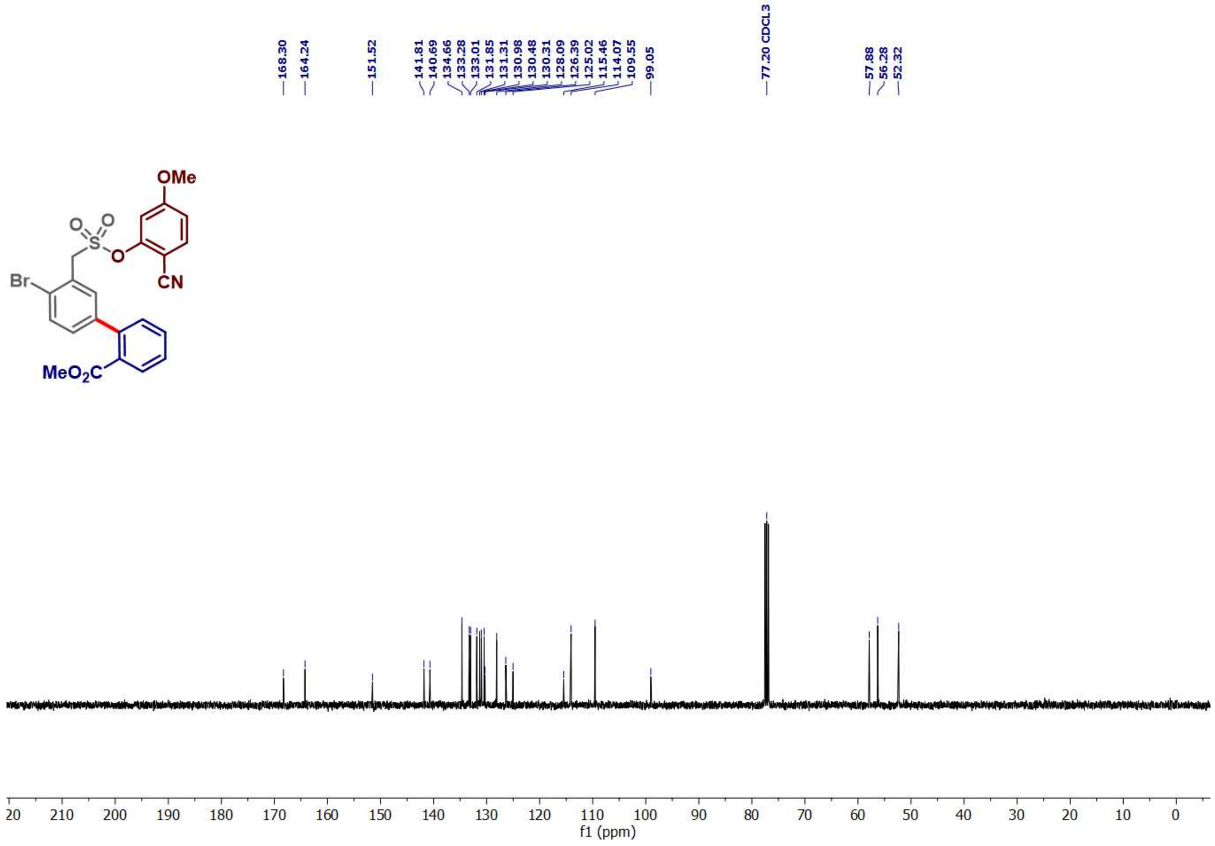

168.30
164.24
151.52
141.81
140.69
134.66
133.28
133.01
131.85
131.31
130.98
130.48
130.31
128.09
126.39
125.02
115.46
114.07
109.55
99.05
77.20 CDCL3
57.88
56.28
52.32
OMe
CN
Br
MeO_2C
f1 (ppm)

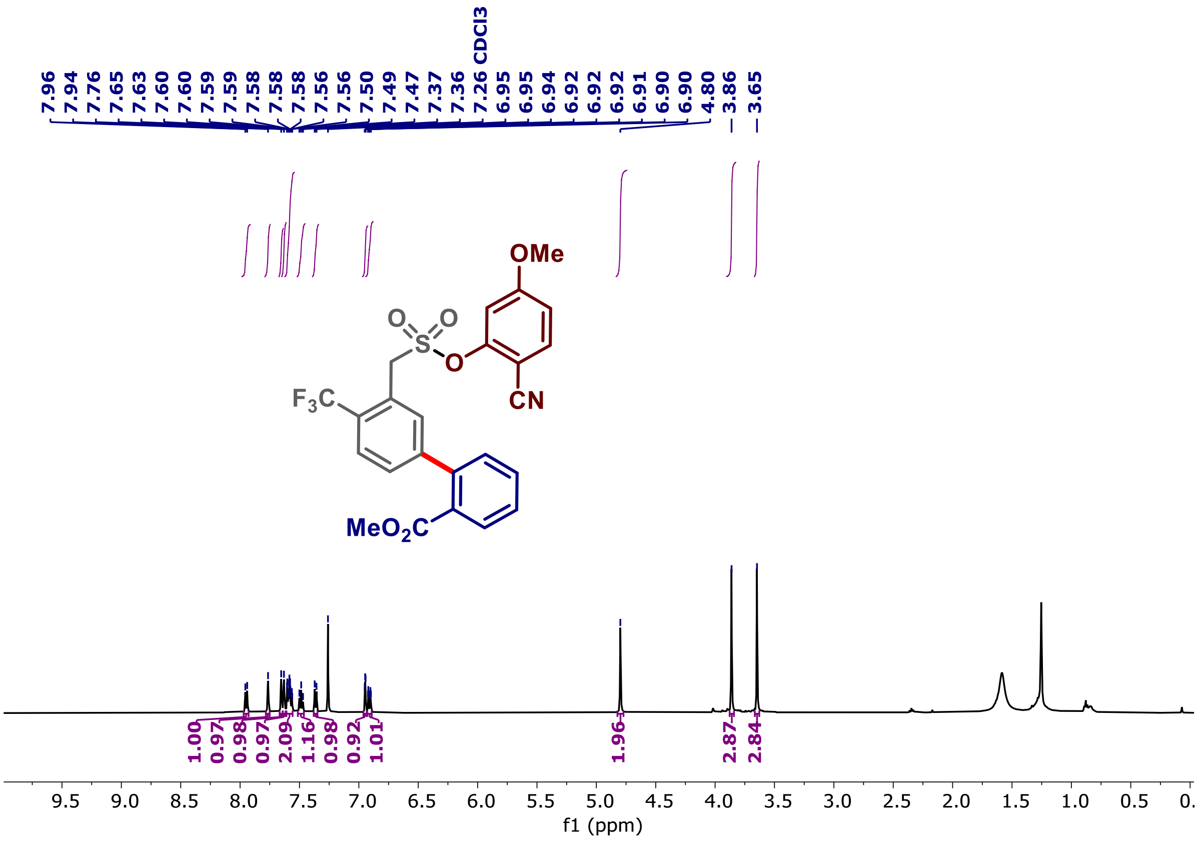
7.96
7.94
7.76
7.65
7.63
7.60
7.60
7.59
7.59
7.58
7.58
7.58
7.56
7.56
7.50
7.49
7.47
7.37
7.36
7.26 CDCl3
6.95
6.95
6.94
6.92
6.92
6.92
6.91
6.90
6.90
4.80
3.86
3.65
OMe
O
S
O
O
CN
F3C
MeO2C
1.00
0.97
0.98
0.97
2.09
1.16
0.98
0.92
1.01
1.96
2.87
2.84
9.5
9.0
8.5
8.0
7.5
7.0
6.5
6.0
5.5
5.0
4.5
4.0
3.5
3.0
2.5
2.0
1.5
1.0
0.5
f1 (ppm)

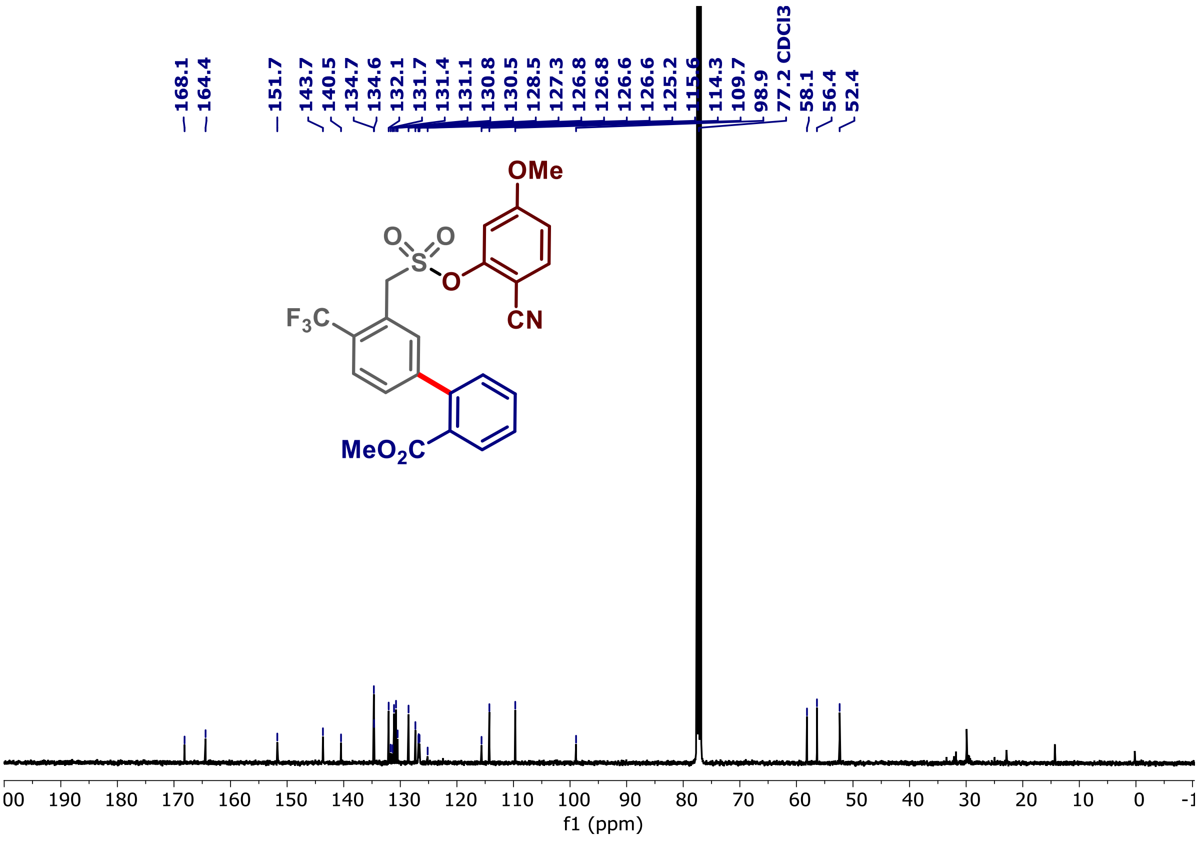
168.1
164.4
151.7
143.7
140.5
134.7
134.6
132.1
131.7
131.4
131.1
130.8
130.5
128.5
127.3
126.8
126.8
126.6
126.6
125.2
115.6
114.3
109.7
98.9
77.2 CDCl3
58.1
56.4
52.4
OMe
O
S
O
O
CN
F3C
MeO2C
190
180
170
160
150
140
130
120
110
100
90
80
70
60
50
40
30
20
10
0
f1 (ppm)

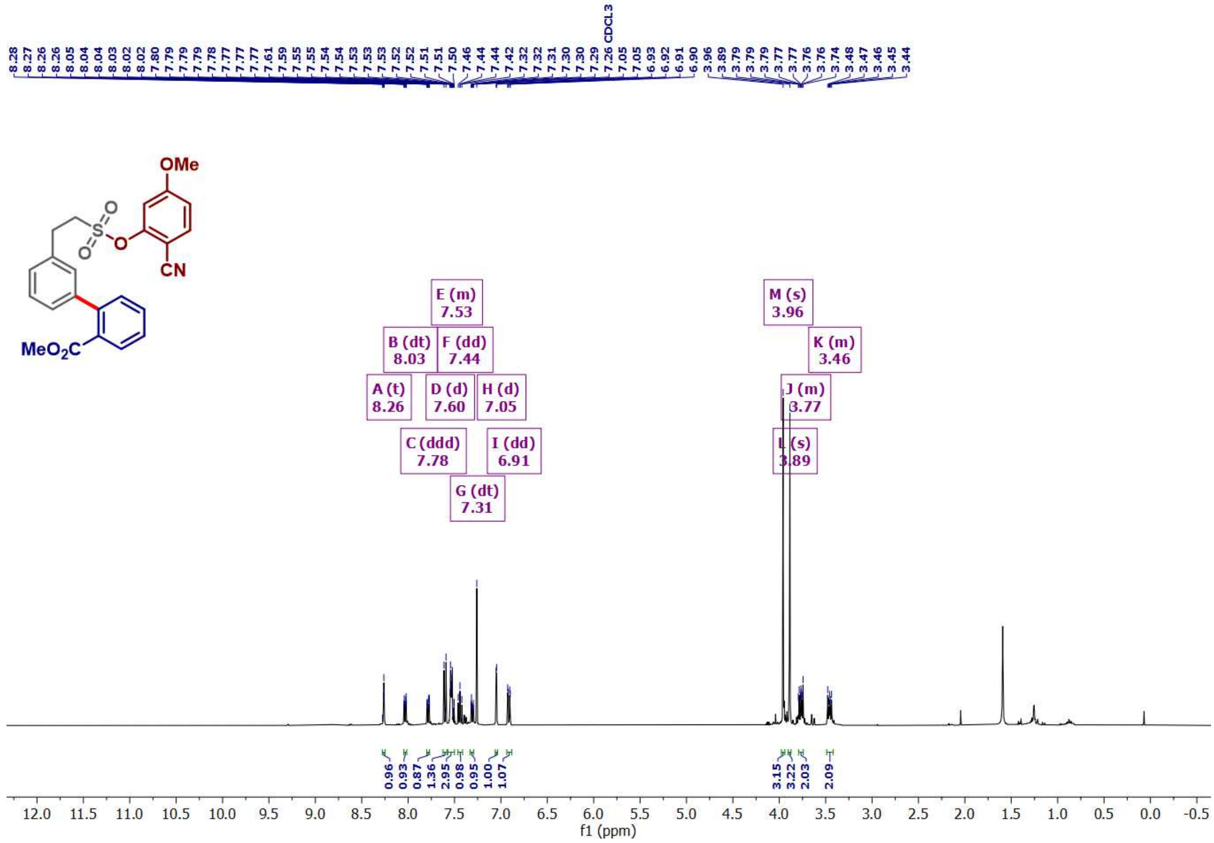

8.28 8.27 8.26 8.26 8.05 8.04 8.04 8.03 8.02 8.02 7.80 7.79 7.79 7.79 7.78 7.77 7.77 7.77 7.61 7.59 7.55 7.55 7.54 7.54 7.53 7.53 7.53 7.52 7.52 7.51 7.51 7.50 7.46 7.44 7.44 7.42 7.32 7.32 7.31 7.30 7.30 7.29 7.26 CDCL3 7.05 7.05 6.93 6.92 6.91 6.90 3.96 3.89 3.79 3.79 3.79 3.77 3.77 3.76 3.76 3.74 3.48 3.47 3.46 3.45 3.44
OMe
CN
MeO2C
E (m) 7.53
B (dt) 8.03
F (dd) 7.44
A (t) 8.26
D (d) 7.60
H (d) 7.05
C (ddd) 7.78
I (dd) 6.91
G (dt) 7.31
M (s) 3.96
K (m) 3.46
J (m) 3.77
L (s) 3.89
0.96 0.93 0.87 1.36 2.95 0.98 0.95 1.00 1.07 3.15 3.22 2.03 2.09
f1 (ppm)

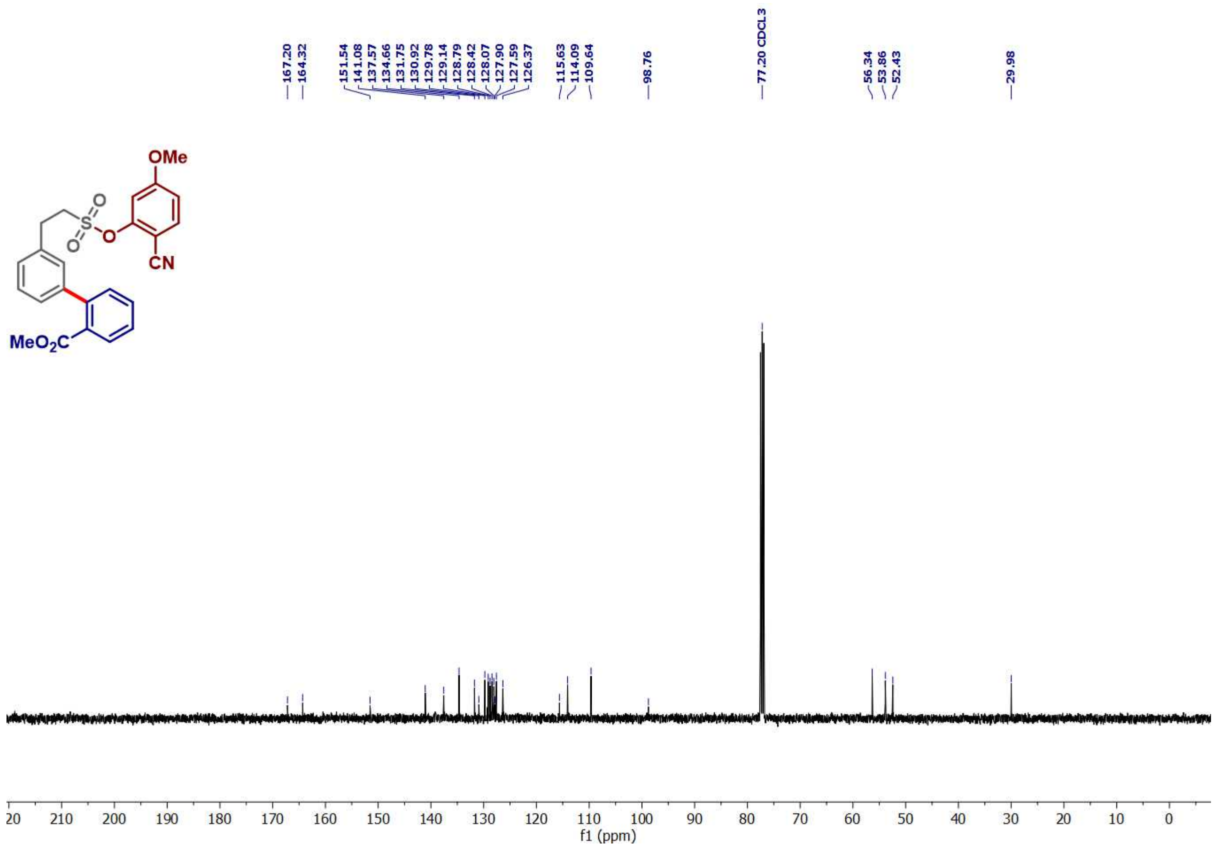

167.20 164.32 151.54 141.08 137.57 134.66 131.75 130.92 129.78 129.14 128.79 128.42 128.07 127.90 127.59 126.37 115.63 114.09 109.64 98.76 77.20 CDCL3 56.34 53.86 52.43 29.98
OMe
CN
MeO2C
f1 (ppm)

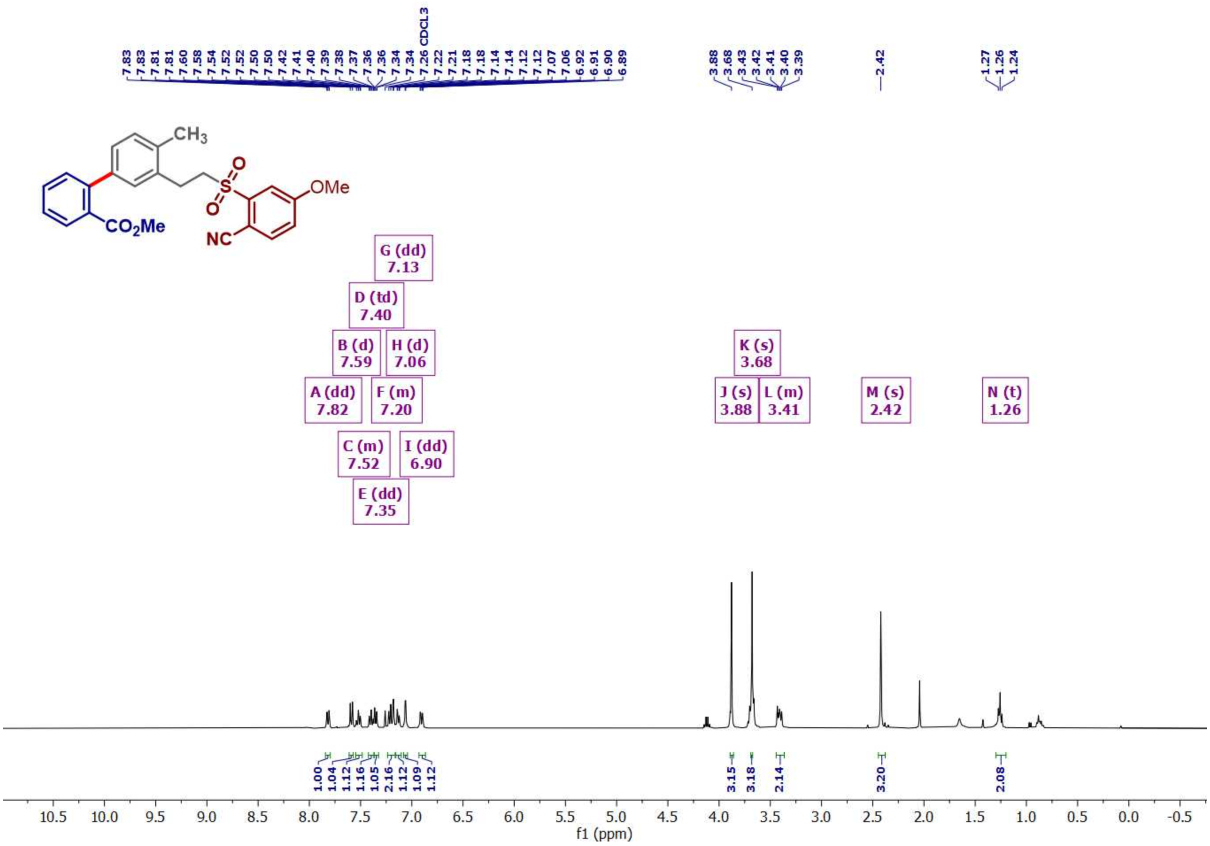
7.83 7.83 7.81 7.81 7.60 7.58 7.54 7.52 7.52 7.50 7.50 7.42 7.41 7.40 7.39 7.38 7.37 7.36 7.36 7.34 7.34 7.26 CDCL3 7.22 7.21 7.18 7.18 7.14 7.14 7.12 7.12 7.07 7.06 6.92 6.91 6.90 6.89
3.88 3.68 3.43 3.42 3.41 3.40 3.39
2.42
1.27 1.26 1.24
CH3
OMe
CO2Me
NC
G (dd) 7.13
D (td) 7.40
B (d) 7.59
H (d) 7.06
A (dd) 7.82
F (m) 7.20
C (m) 7.52
I (dd) 6.90
E (dd) 7.35
K (s) 3.68
J (s) 3.88
L (m) 3.41
M (s) 2.42
N (t) 1.26
1.00 1.04 1.12 1.16 1.05 2.16 1.12 1.09 1.12
3.15 3.18 2.14
3.20
2.08
f1 (ppm)

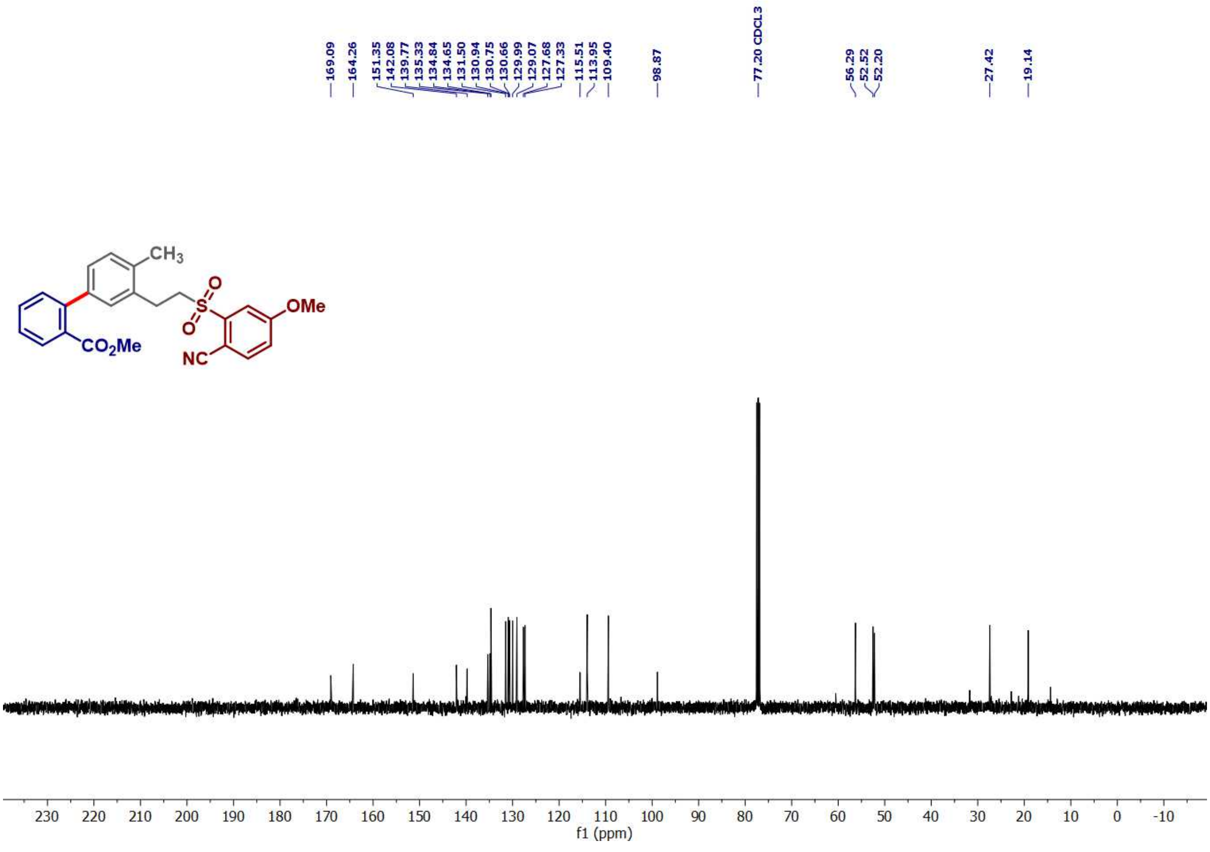
169.09
164.26
151.35 142.08 139.77 135.33 134.84 134.65 131.50 130.94 130.75 130.66 129.99 129.07 127.68 127.33
115.51 113.95 109.40
98.87
77.20 CDCL3
56.29 52.52 52.20
27.42
19.14
CH3
OMe
CO2Me
NC
f1 (ppm)

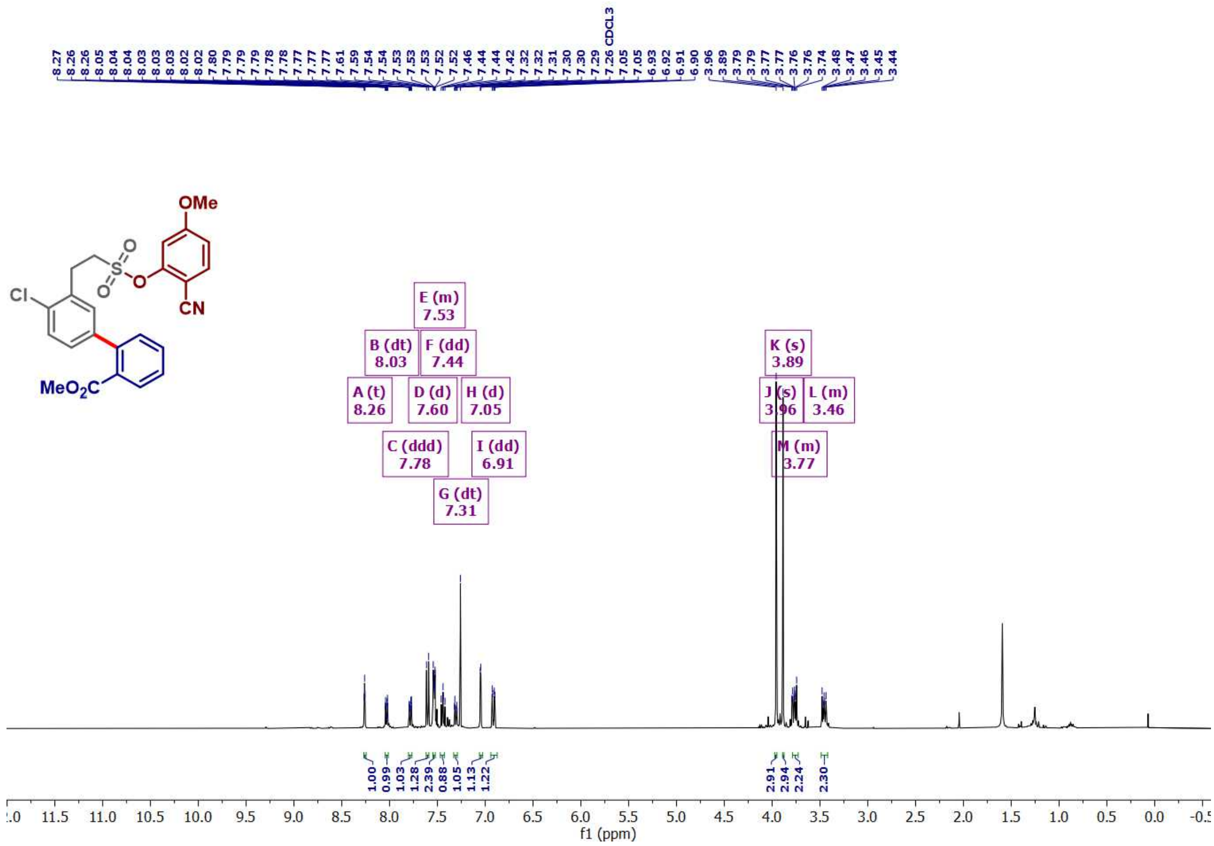
CDCL3
A (t) 8.26
B (dt) 8.03
C (ddd) 7.78
D (d) 7.60
E (m) 7.53
F (dd) 7.44
G (dt) 7.31
H (d) 7.05
I (dd) 6.91
J (s) 3.96
K (s) 3.89
L (m) 3.46
M (m) 3.77
f1 (ppm)
OMe
Cl
CN
MeO2C

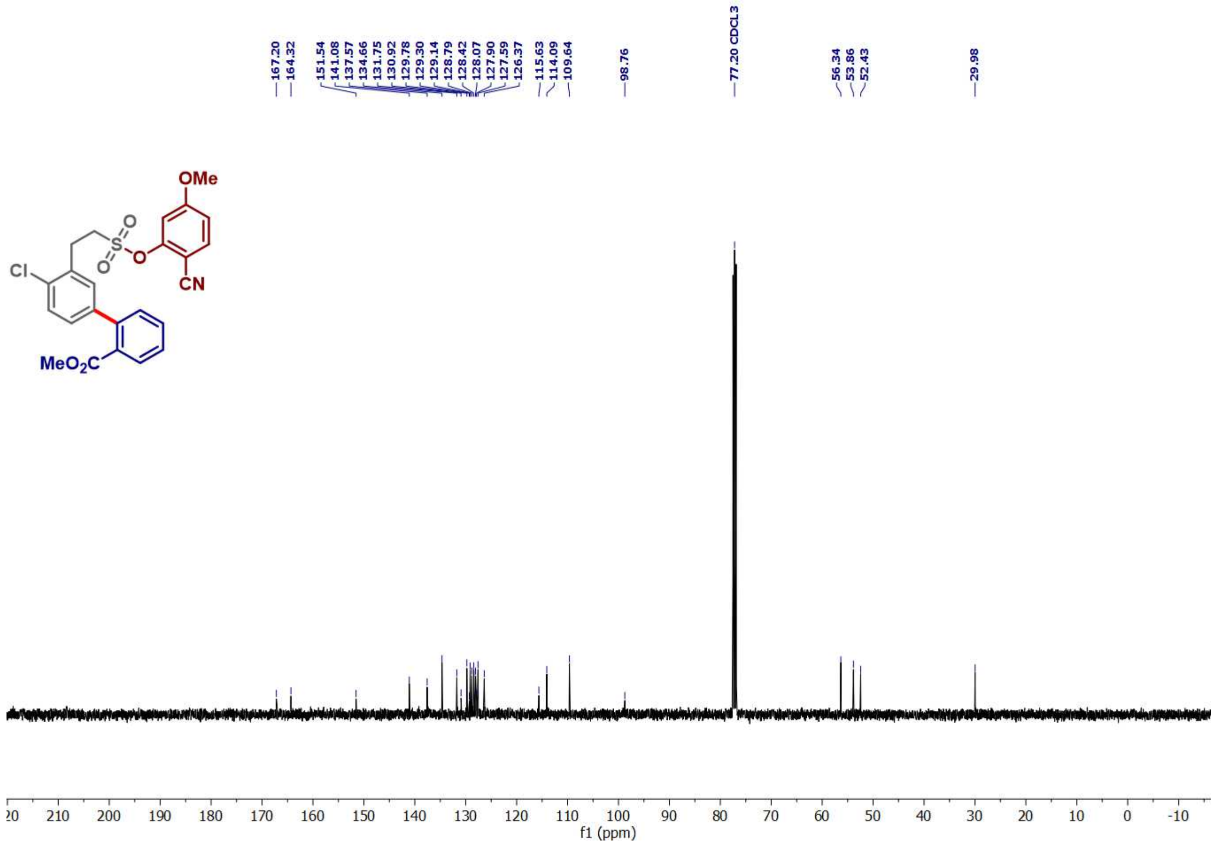
167.20
164.32
151.54
141.08
137.57
134.66
131.75
130.92
129.78
129.30
129.14
128.79
128.42
128.07
127.90
127.59
126.37
115.63
114.09
109.64
98.76
77.20 CDCL3
56.34
53.86
52.43
29.98
f1 (ppm)
OMe
Cl
CN
MeO2C

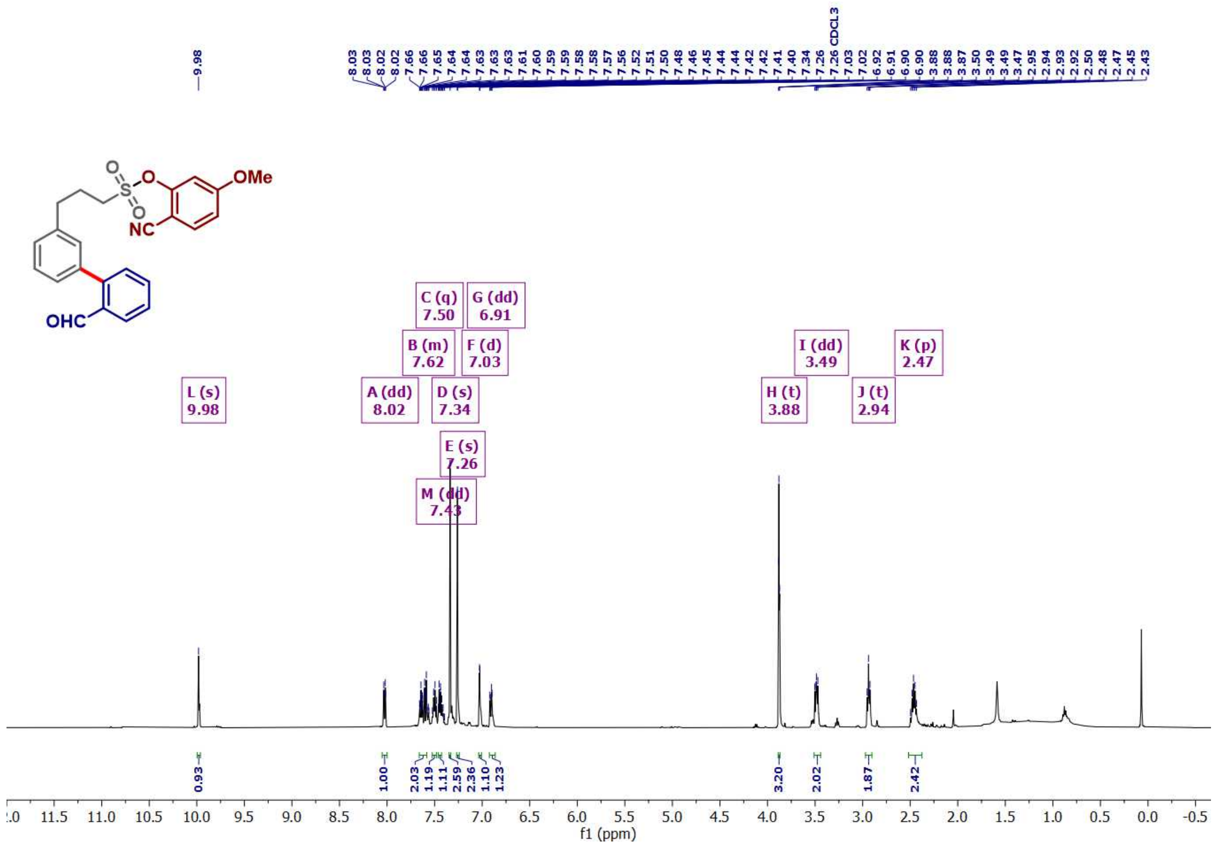

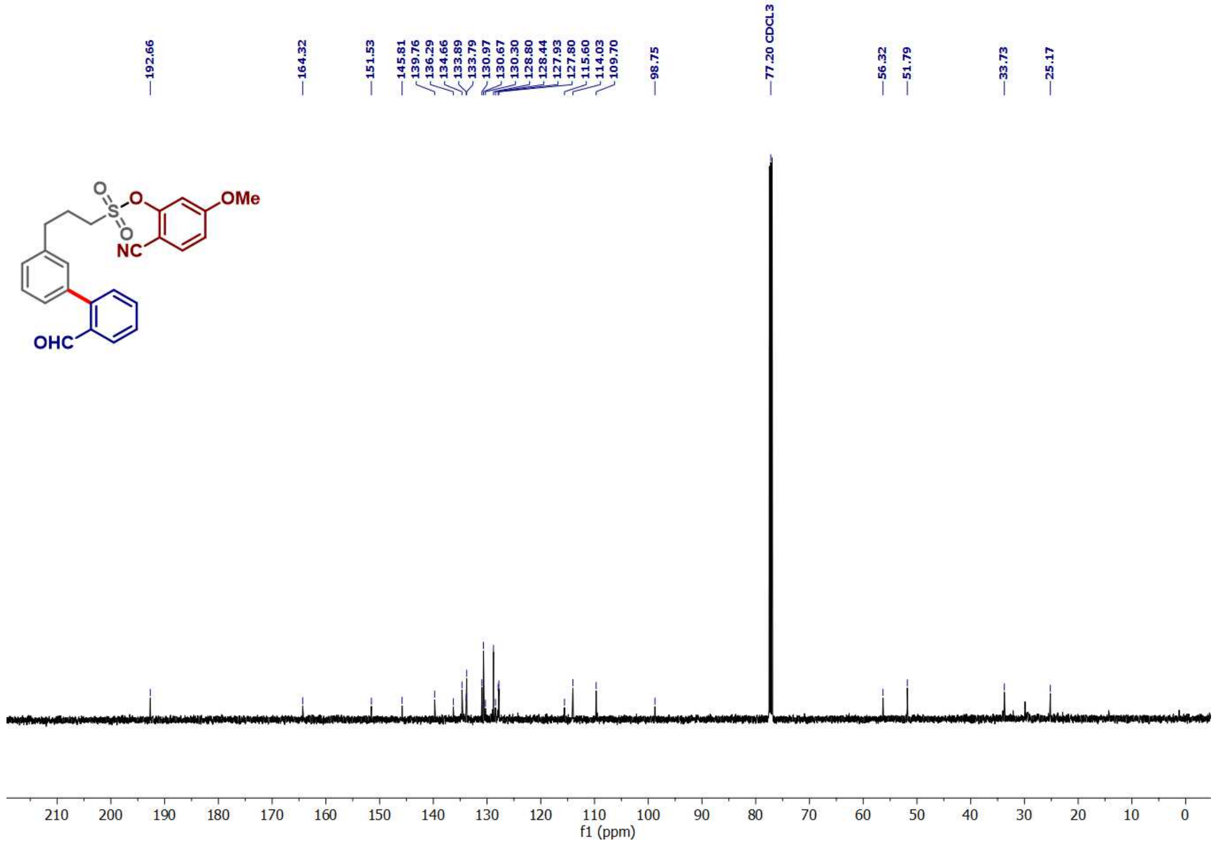

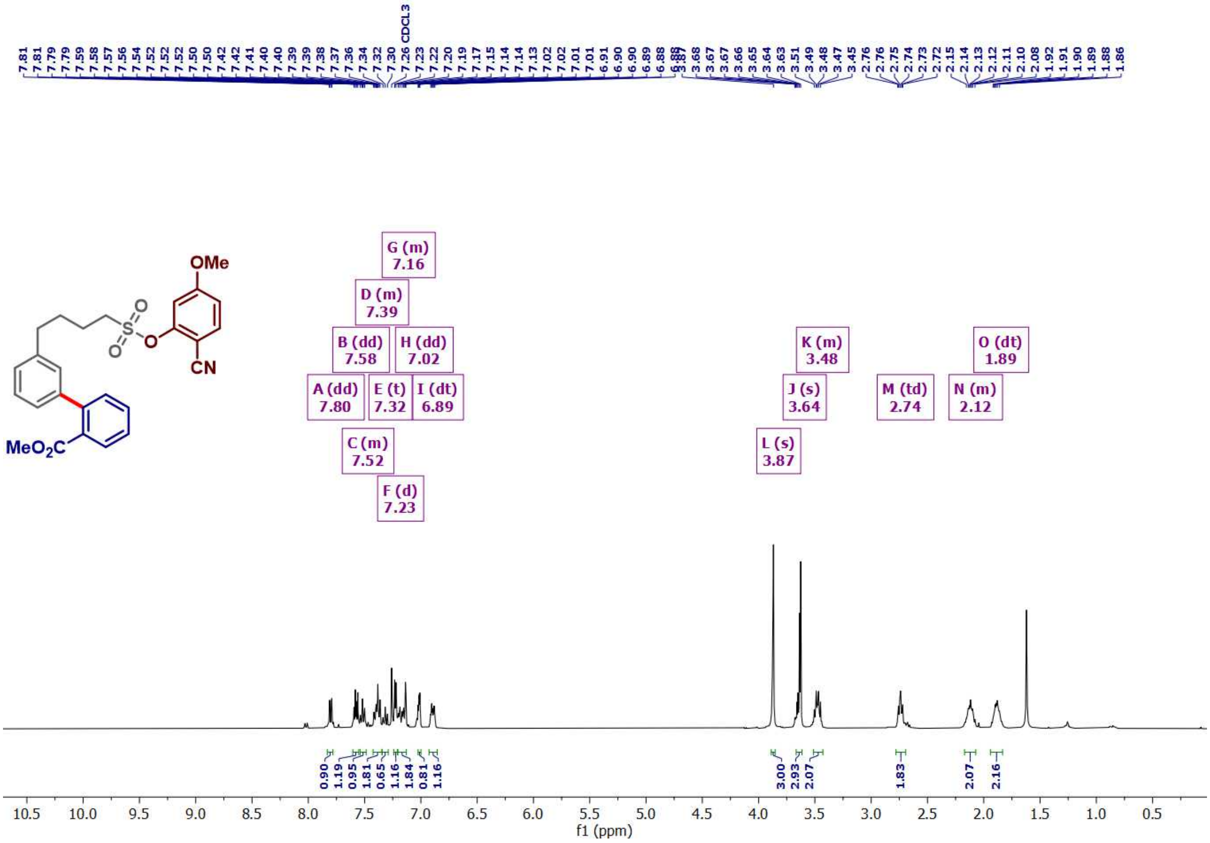
7.81 7.81 7.79 7.79 7.59 7.58 7.57 7.56 7.54 7.52 7.52 7.52 7.50 7.50 7.42 7.42 7.41 7.40 7.40 7.39 7.39 7.38 7.37 7.36 7.34 7.32 7.30 7.26 CDCL3 7.23 7.22 7.20 7.19 7.17 7.15 7.14 7.14 7.13 7.02 7.02 7.01 7.01 6.91 6.90 6.90 6.89 6.88
3.68 3.67 3.67 3.66 3.65 3.64 3.63 3.51 3.49 3.48 3.47 3.45 2.76 2.76 2.75 2.74 2.73 2.72 2.15 2.14 2.13 2.12 2.11 2.10 2.08 1.92 1.91 1.90 1.89 1.88 1.86
OMe
CN
MeO2C
G (m) 7.16
D (m) 7.39
B (dd) 7.58
H (dd) 7.02
A (dd) 7.80
E (t) 7.32
I (dt) 6.89
C (m) 7.52
F (d) 7.23
K (m) 3.48
J (s) 3.64
L (s) 3.87
M (td) 2.74
N (m) 2.12
O (dt) 1.89
0.90 1.19 0.95 1.81 0.65 1.16 1.84 0.81 1.16 3.00 2.93 2.07 1.83 2.07 2.16
f1 (ppm)

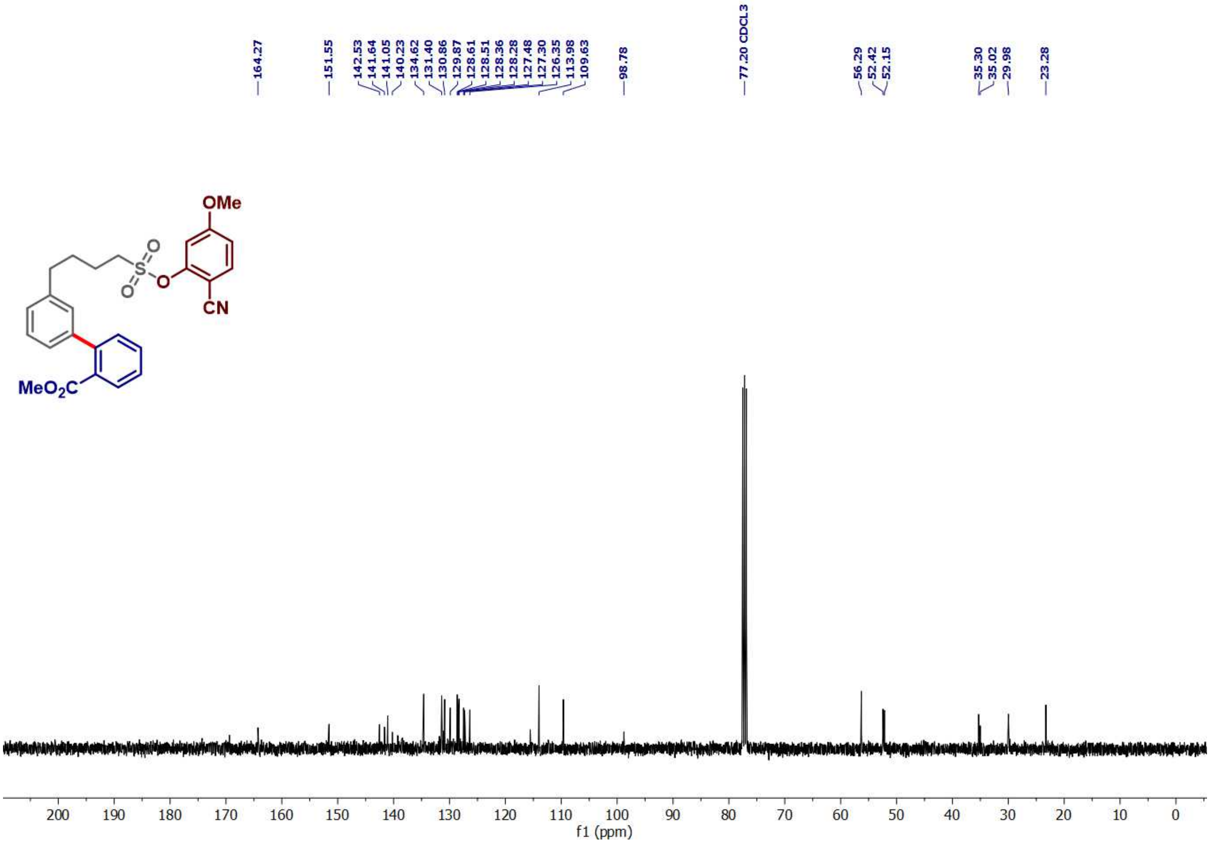
164.27
151.55
142.53 141.64 141.05 140.23 134.62 131.40 130.86 129.87 128.61 128.51 128.36 128.28 127.48 127.30 126.35 113.98 109.63
98.78
77.20 CDCL3
56.29 52.42 52.15
35.30 35.02 29.98
23.28
OMe
CN
MeO2C
f1 (ppm)

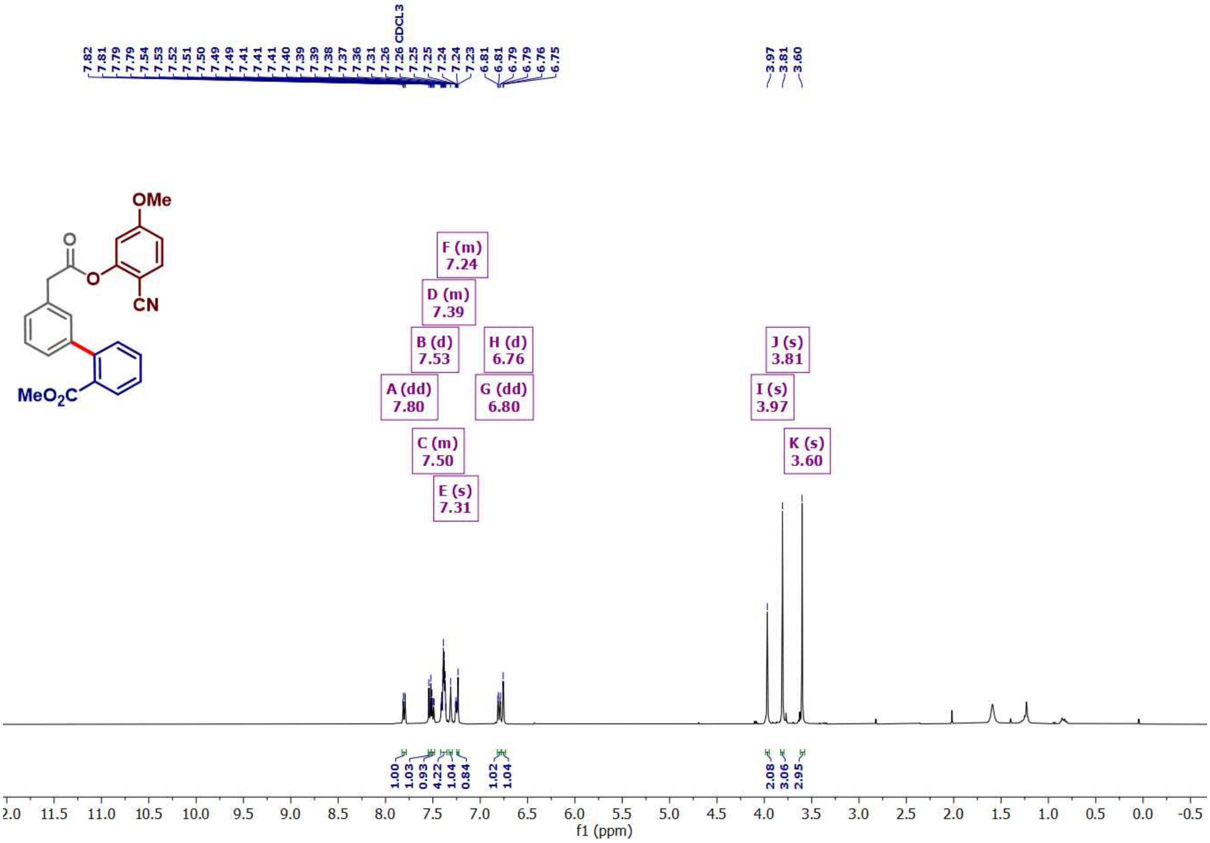
7.82 7.81 7.79 7.79 7.54 7.53 7.52 7.51 7.50 7.49 7.49 7.41 7.41 7.41 7.40 7.39 7.39 7.38 7.37 7.36 7.31 7.26 CDCL3 7.25 7.25 7.24 7.24 7.23 6.81 6.81 6.79 6.79 6.76 6.75
3.97 3.81 3.60
OMe
CN
MeO2C
F (m) 7.24
D (m) 7.39
B (d) 7.53
H (d) 6.76
A (dd) 7.80
G (dd) 6.80
C (m) 7.50
E (s) 7.31
J (s) 3.81
I (s) 3.97
K (s) 3.60
1.00 1.03 0.93 4.22 1.04 0.84 1.02 1.04
2.08 3.06 2.95
f1 (ppm)
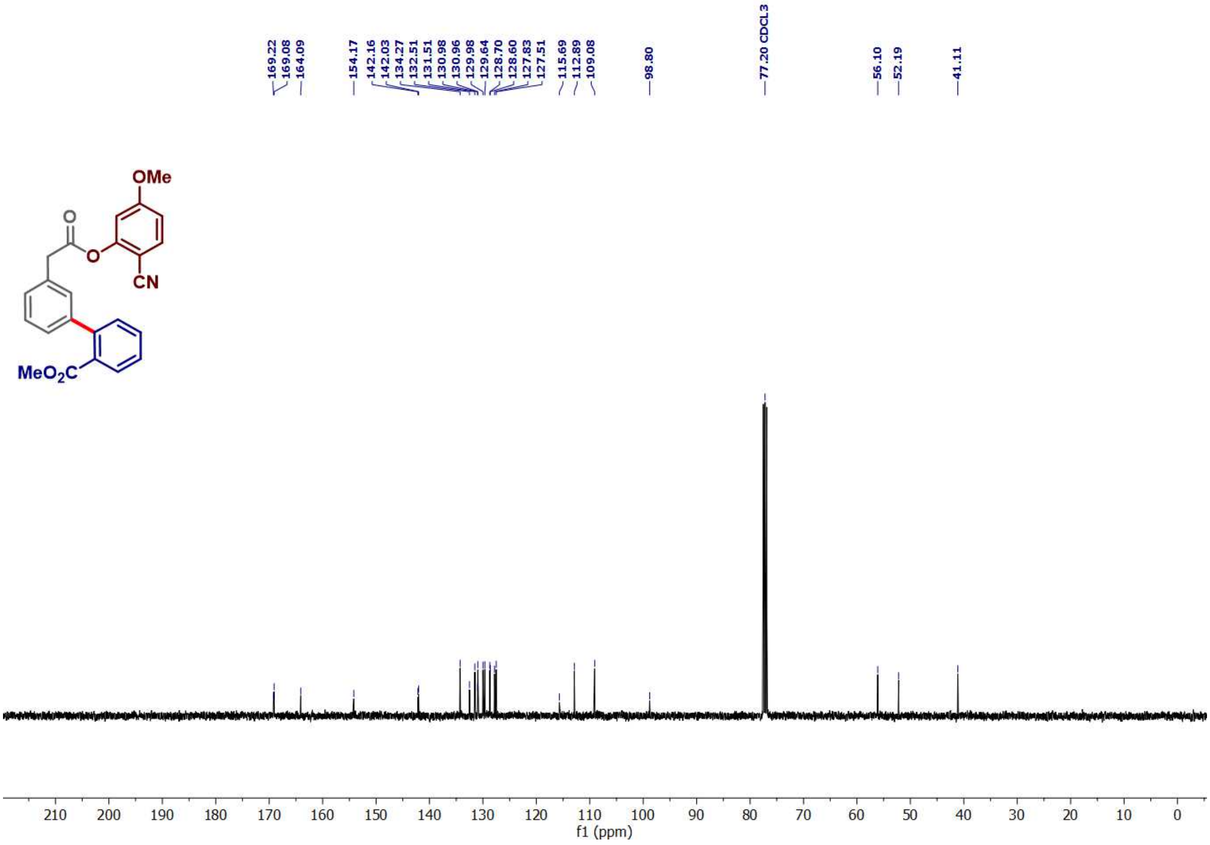
169.22 169.08 164.09 154.17 142.16 142.03 134.27 132.51 131.51 130.98 130.96 129.98 129.64 128.70 128.60 127.83 127.51 115.69 112.89 109.08 98.80 77.20 CDCL3 56.10 52.19 41.11
OMe
CN
MeO2C
f1 (ppm)

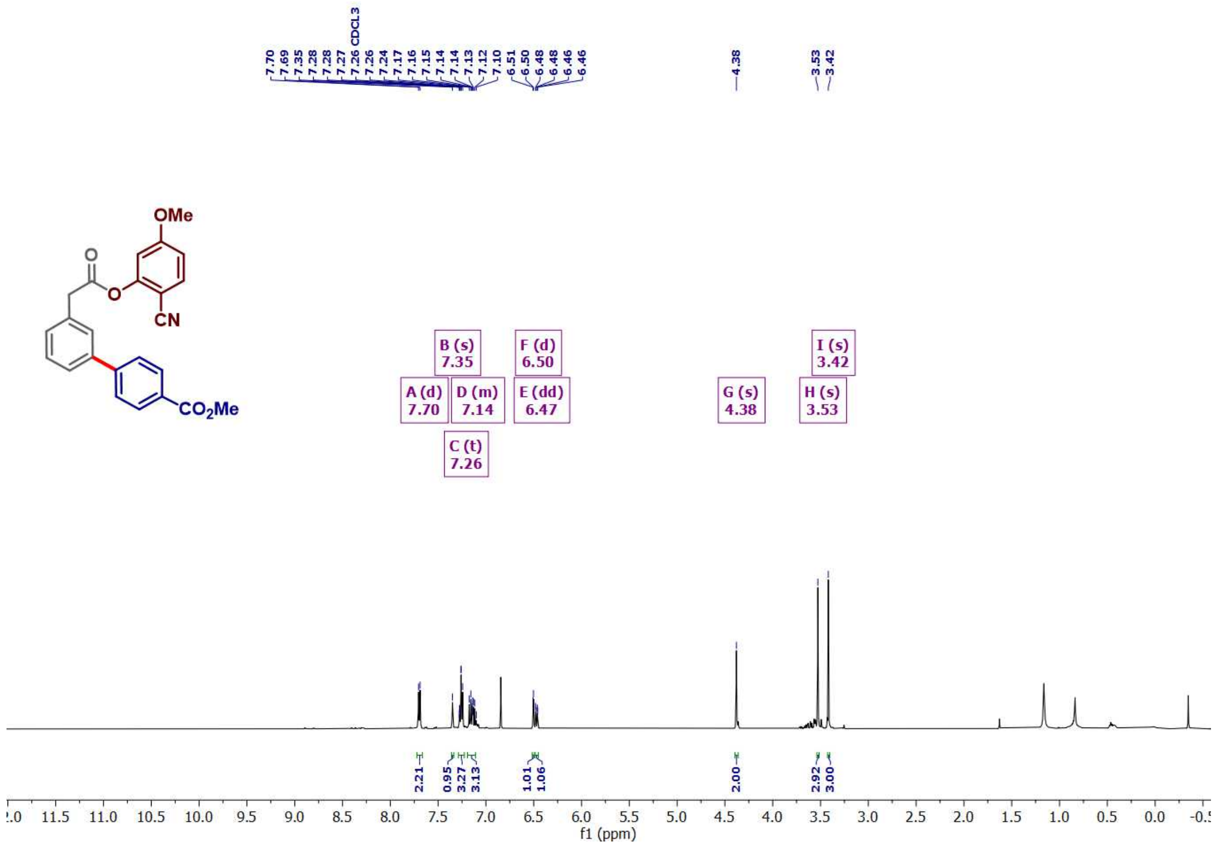
7.70 7.69 7.35 7.28 7.28 7.27 7.26 CDCL3 7.26 7.24 7.17 7.16 7.15 7.14 7.14 7.13 7.12 7.10 6.51 6.50 6.48 6.48 6.46 6.46
4.38
3.53 3.42
OMe
O
O
CN
CO2Me
B (s) 7.35
F (d) 6.50
I (s) 3.42
A (d) 7.70
D (m) 7.14
E (dd) 6.47
G (s) 4.38
H (s) 3.53
C (t) 7.26
2.21 0.95 3.27 3.13 1.01 1.06 2.00 2.92 3.00
f1 (ppm)

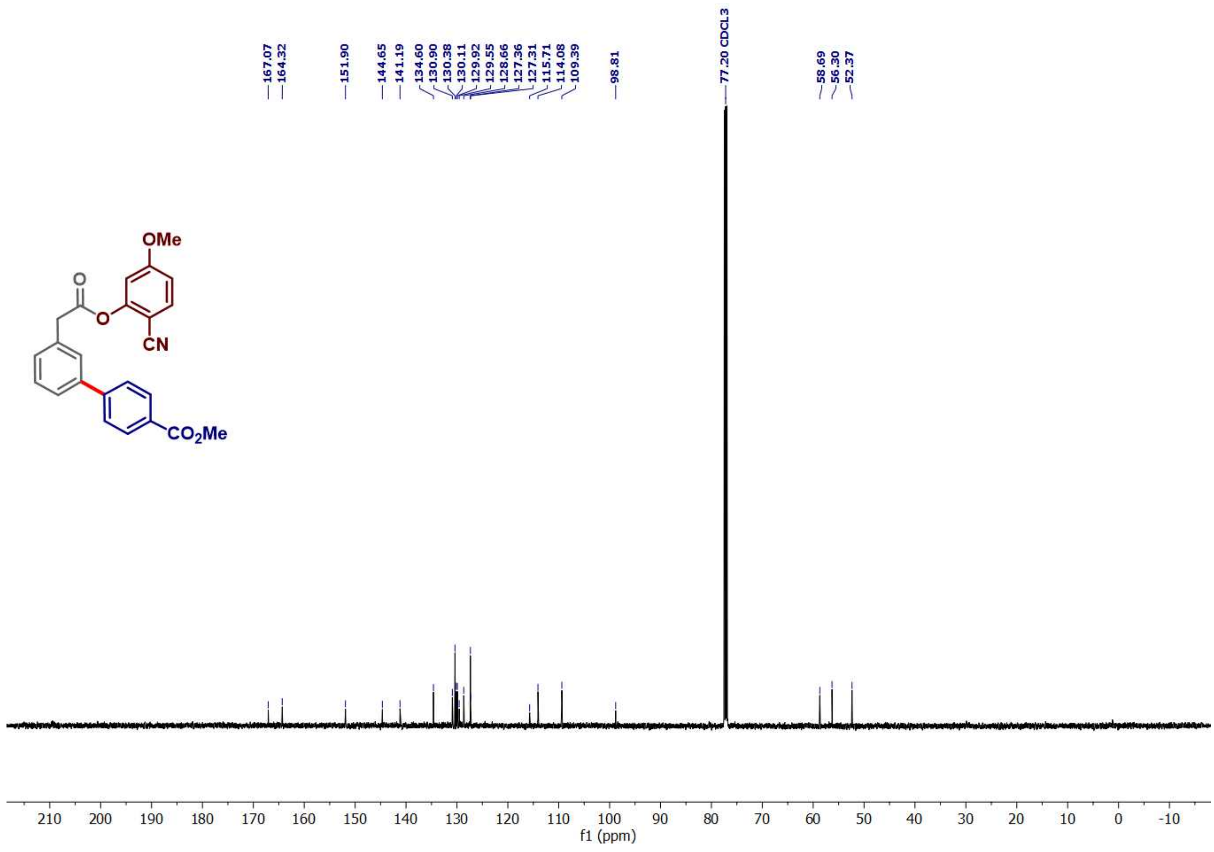
167.07 164.32 151.90 144.65 141.19 134.60 130.90 130.38 130.11 129.92 129.55 128.66 127.36 127.31 115.71 114.08 109.39 98.81
77.20 CDCL3
58.69 56.30 52.37
OMe
O
O
CN
CO2Me
f1 (ppm)

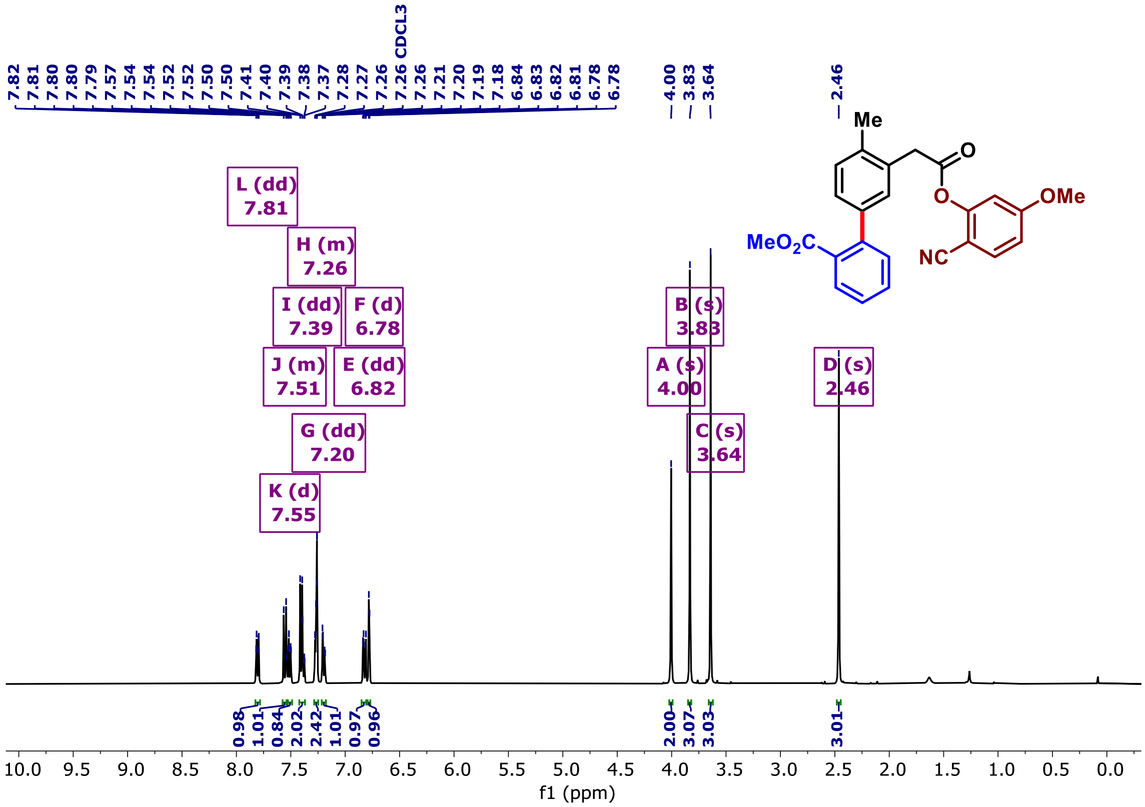
7.82 7.81 7.80 7.80 7.79 7.57 7.54 7.54 7.52 7.52 7.50 7.50 7.41 7.40 7.39 7.38 7.37 7.28 7.27 7.26 7.26 CDCL3 7.26 7.21 7.20 7.19 7.18 6.84 6.83 6.82 6.81 6.78 6.78
4.00 3.83 3.64
2.46
L (dd) 7.81
H (m) 7.26
I (dd) 7.39
F (d) 6.78
J (m) 7.51
E (dd) 6.82
G (dd) 7.20
K (d) 7.55
B (s) 3.83
A (s) 4.00
C (s) 3.64
D (s) 2.46
Me
O
O
OMe
MeO2C
NC
0.98 1.01 0.84 2.02 2.42 1.01 0.97 0.96
2.00 3.07 3.03
3.01
10.0 9.5 9.0 8.5 8.0 7.5 7.0 6.5 6.0 5.5 5.0 4.5 4.0 3.5 3.0 2.5 2.0 1.5 1.0 0.5 0.0
f1 (ppm)

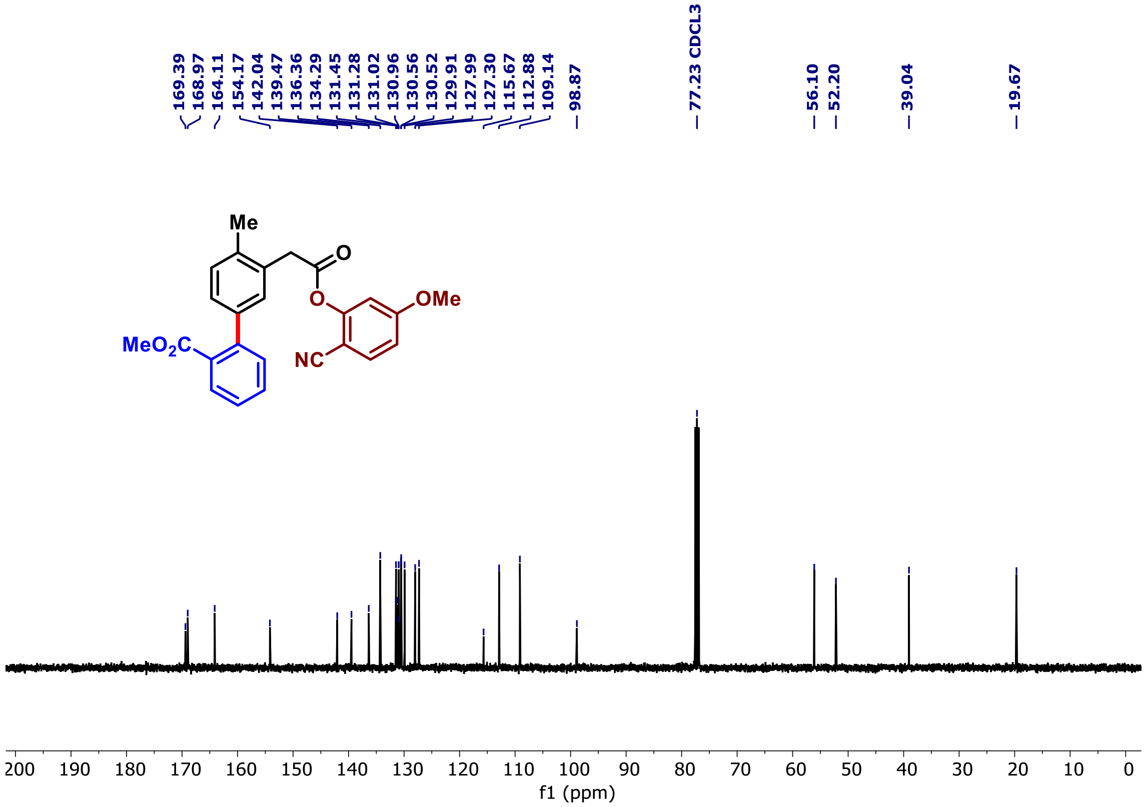
169.39 168.97 164.11 154.17 142.04 139.47 136.36 134.29 131.45 131.28 131.02 130.96 130.56 130.52 129.91 127.99 127.30 115.67 112.88 109.14 98.87
77.23 CDCL3
56.10 52.20
39.04
19.67
Me
O
O
OMe
MeO2C
NC
200 190 180 170 160 150 140 130 120 110 100 90 80 70 60 50 40 30 20 10 0
f1 (ppm)

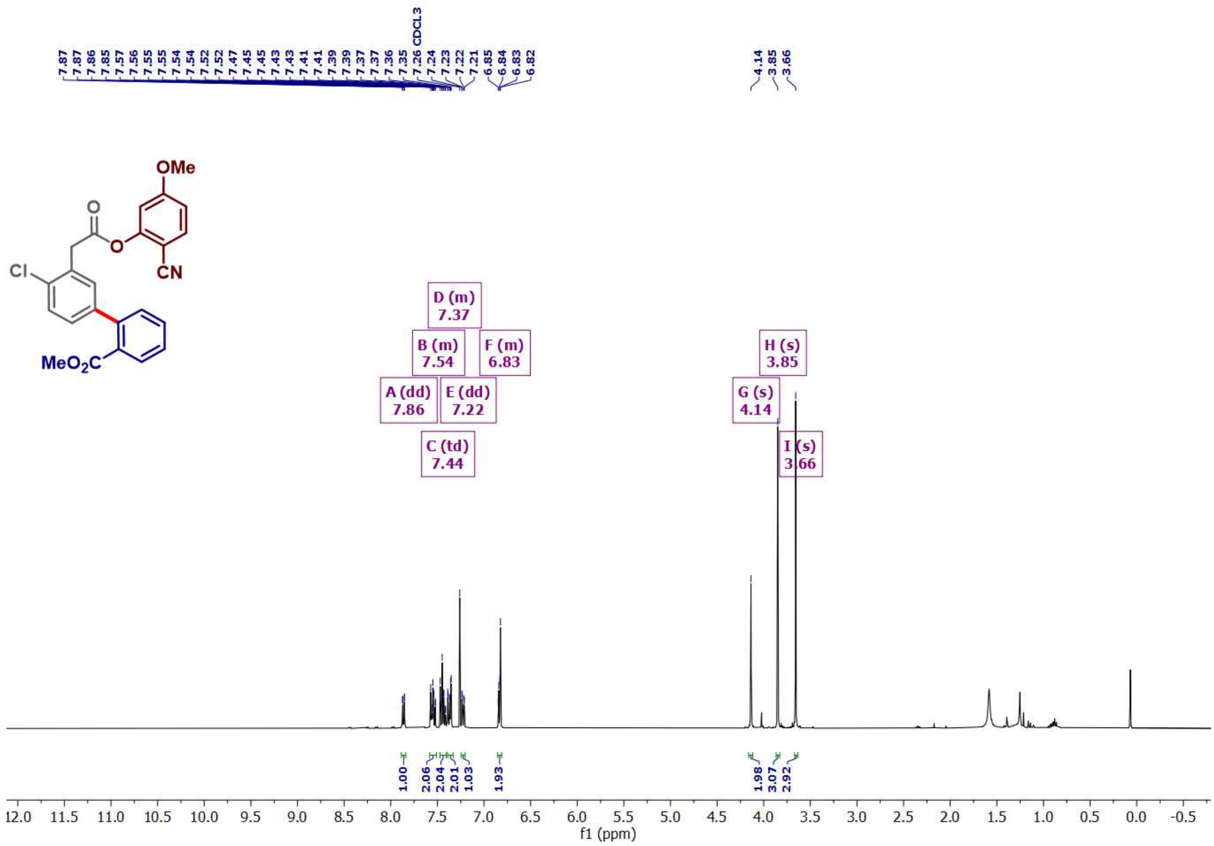
7.87
7.87
7.86
7.85
7.57
7.56
7.55
7.55
7.54
7.54
7.52
7.52
7.47
7.45
7.45
7.43
7.43
7.41
7.41
7.39
7.39
7.37
7.37
7.36
7.35
7.26 CDCL3
7.24
7.23
7.22
7.22
7.21
6.85
6.84
6.83
6.82
4.14
3.85
3.66
OMe
O
O
CN
Cl
MeO2C
D (m)
7.37
B (m)
7.54
F (m)
6.83
A (dd)
7.86
E (dd)
7.22
C (td)
7.44
H (s)
3.85
G (s)
4.14
I (s)
3.66
1.00
2.06
2.04
2.01
1.03
1.93
1.98
3.07
2.92
f1 (ppm)

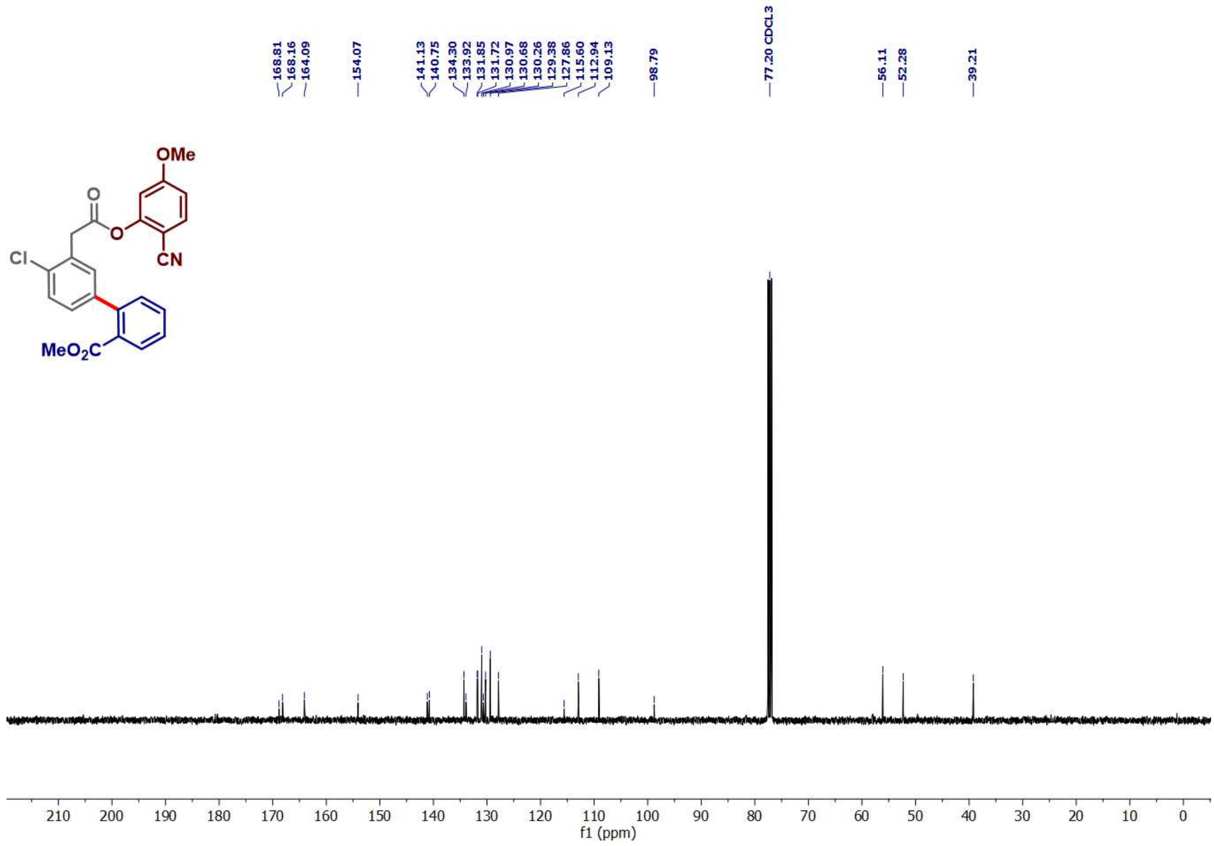
168.81
168.16
164.09
154.07
141.13
140.75
134.30
133.92
131.85
131.72
130.97
130.68
130.26
129.38
127.86
115.60
112.94
109.13
98.79
77.20 CDCL3
56.11
52.28
39.21
OMe
O
O
CN
Cl
MeO2C
f1 (ppm)

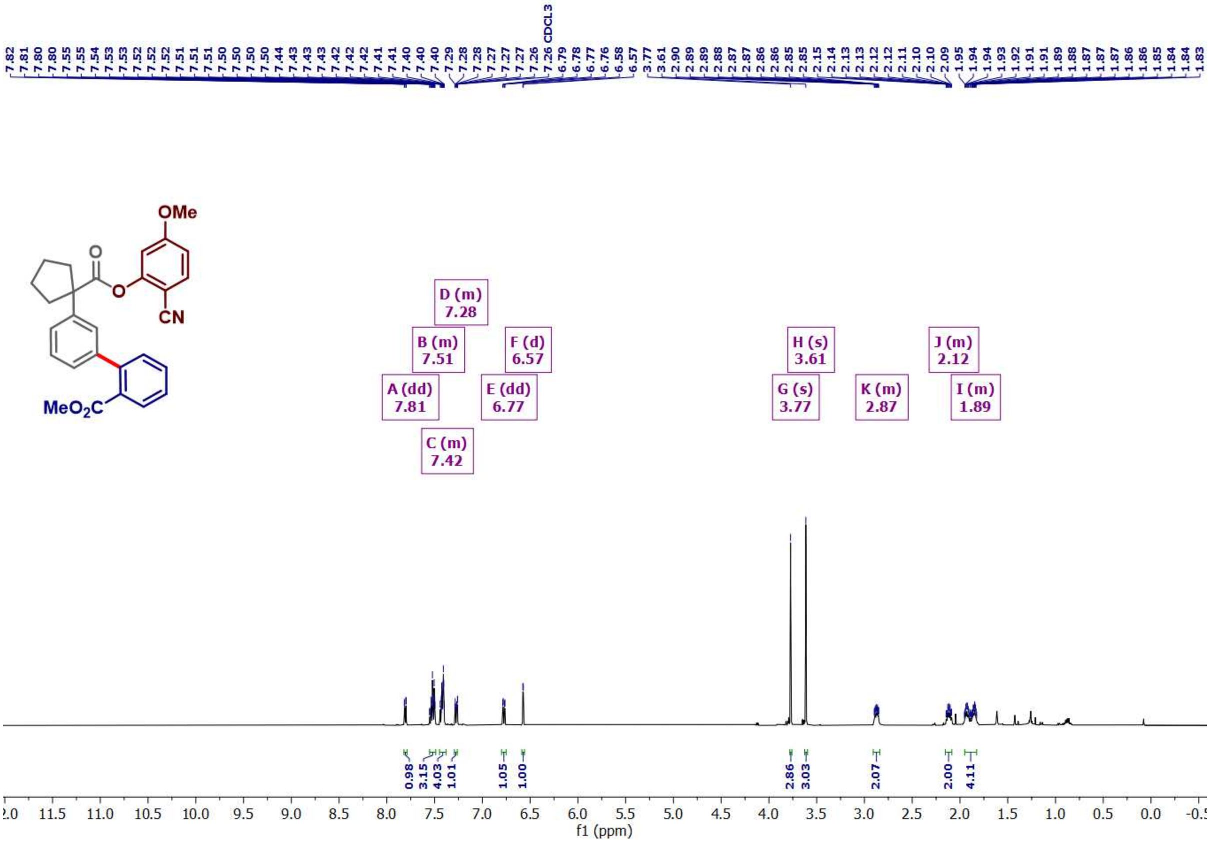

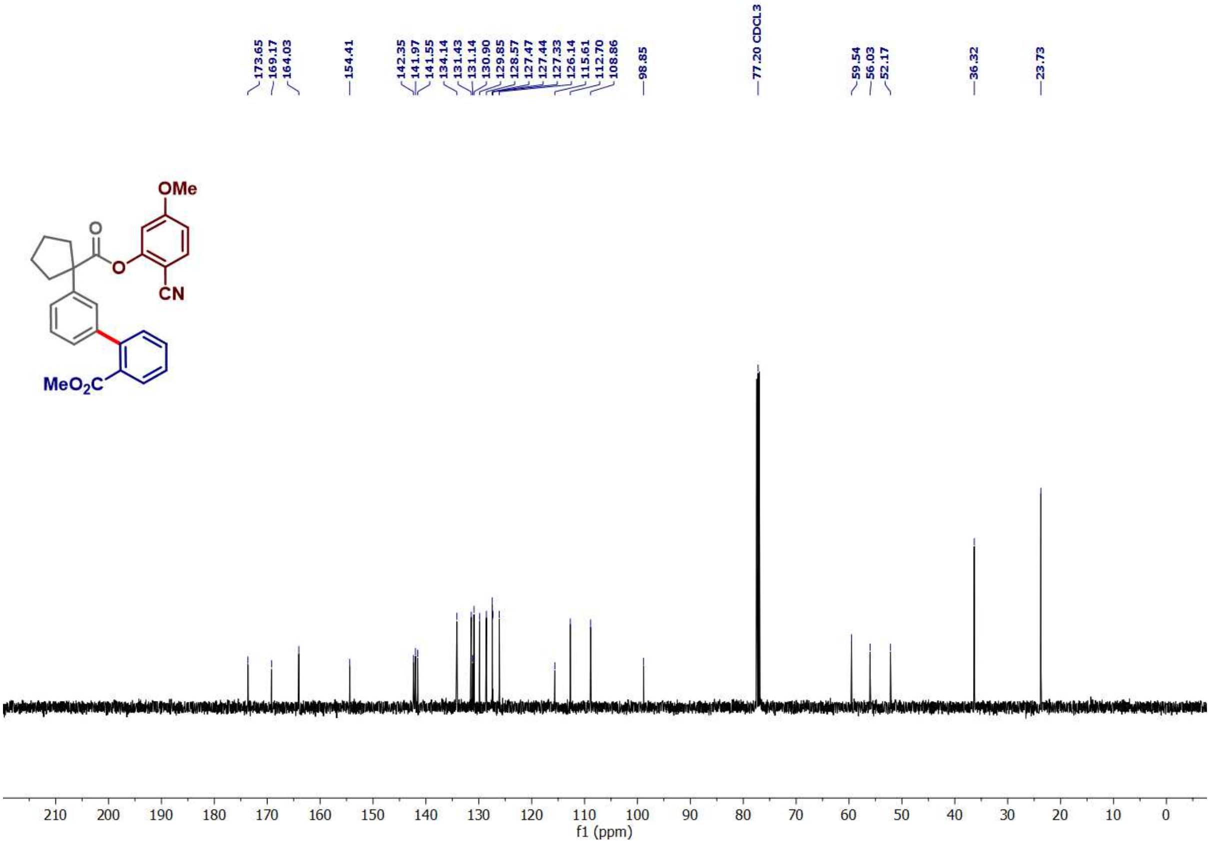

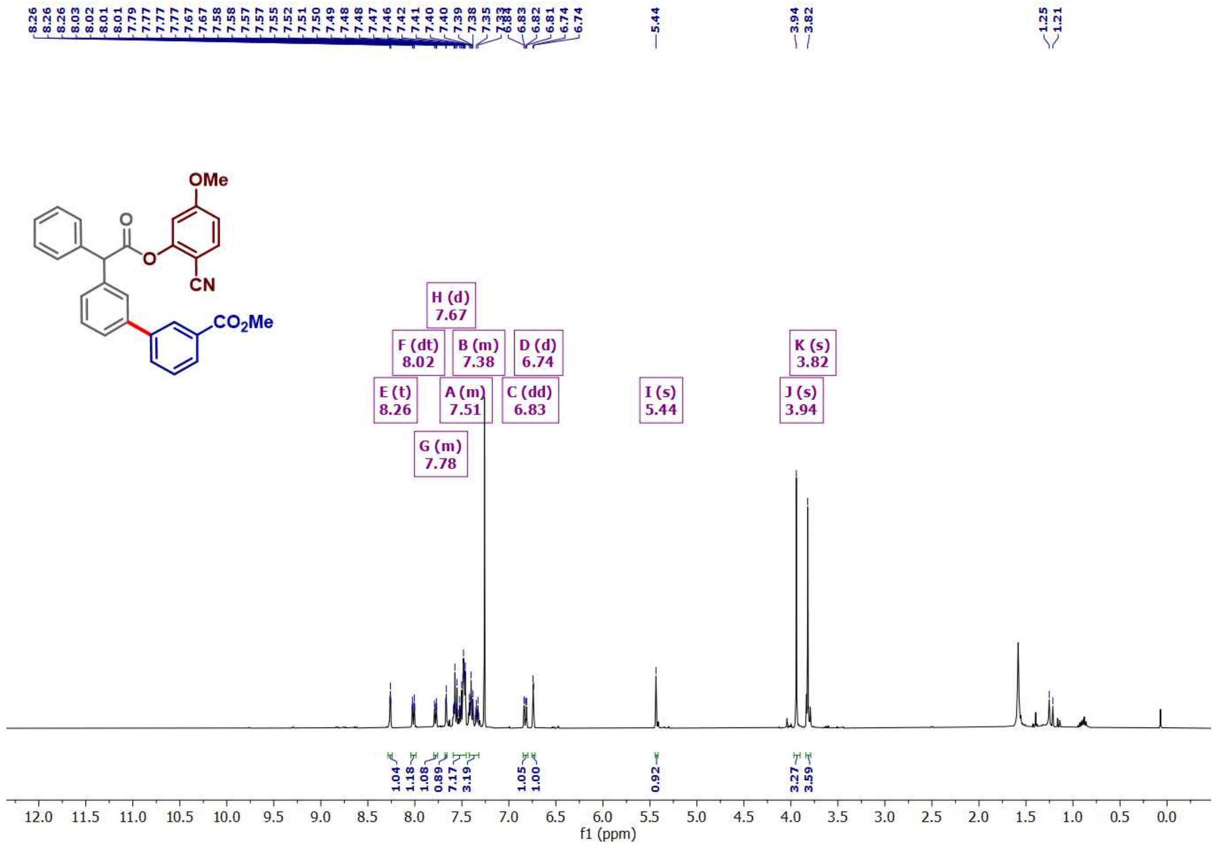

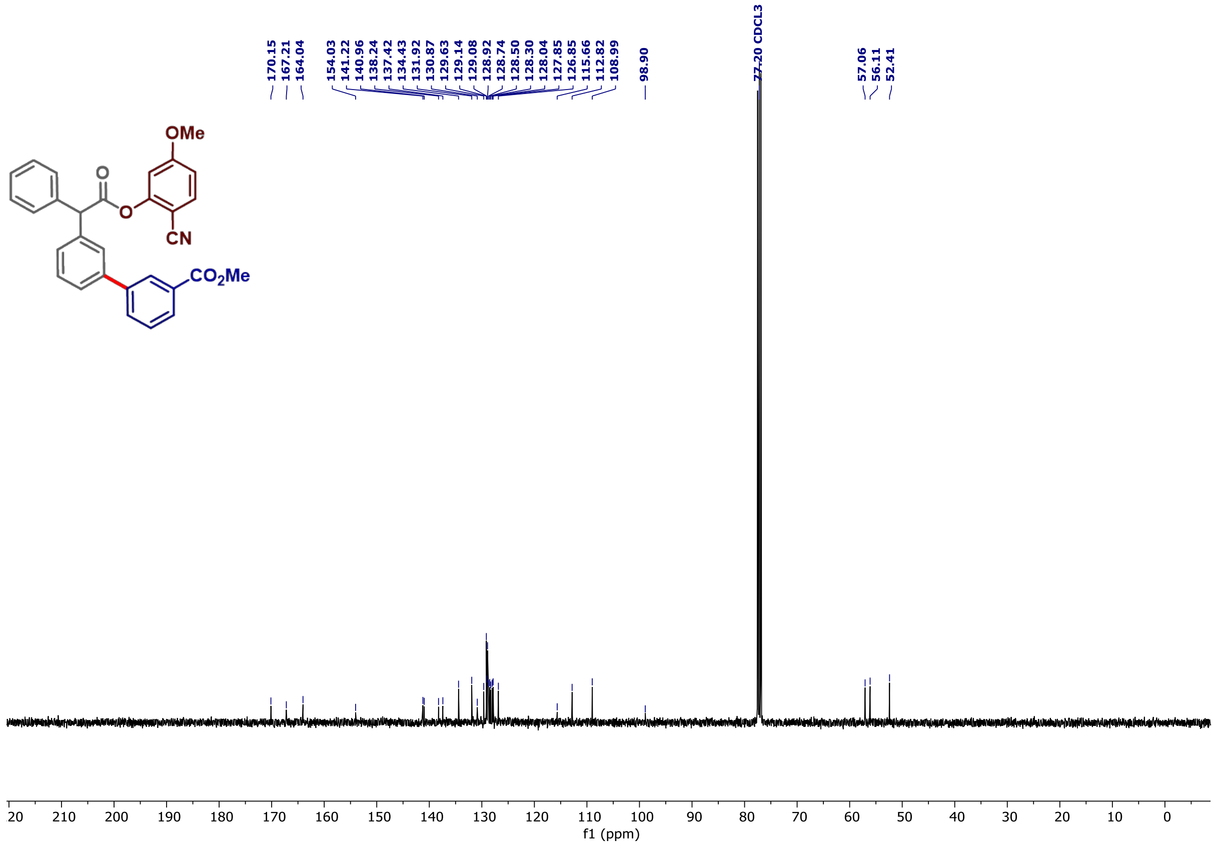

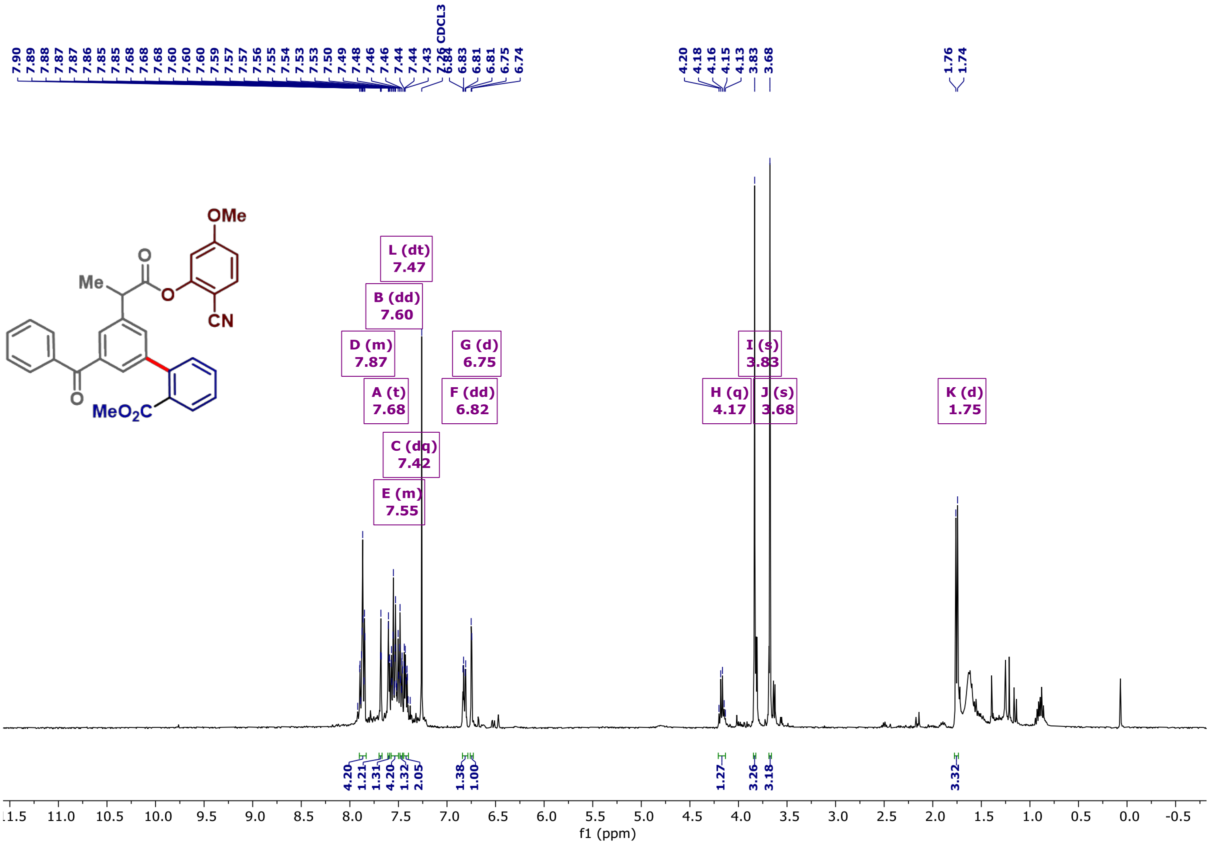
7.90 7.89 7.88 7.87 7.87 7.86 7.85 7.85 7.68 7.68 7.68 7.60 7.60 7.60 7.59 7.57 7.57 7.56 7.55 7.54 7.53 7.53 7.50 7.49 7.48 7.46 7.46 7.44 7.44 7.43 7.26 CDCL3 6.83 6.81 6.81 6.75 6.74
4.20 4.18 4.16 4.15 4.13 3.83 3.68
1.76 1.74
OMe
O
Me
O
CN
O
MeO2C
L (dt) 7.47
B (dd) 7.60
D (m) 7.87
A (t) 7.68
C (dq) 7.42
E (m) 7.55
G (d) 6.75
F (dd) 6.82
I (s) 3.83
H (q) 4.17
J (s) 3.68
K (d) 1.75
4.20 1.21 1.31 4.20 1.32 2.05
1.38 1.00
1.27
3.26 3.18
3.32
f1 (ppm)

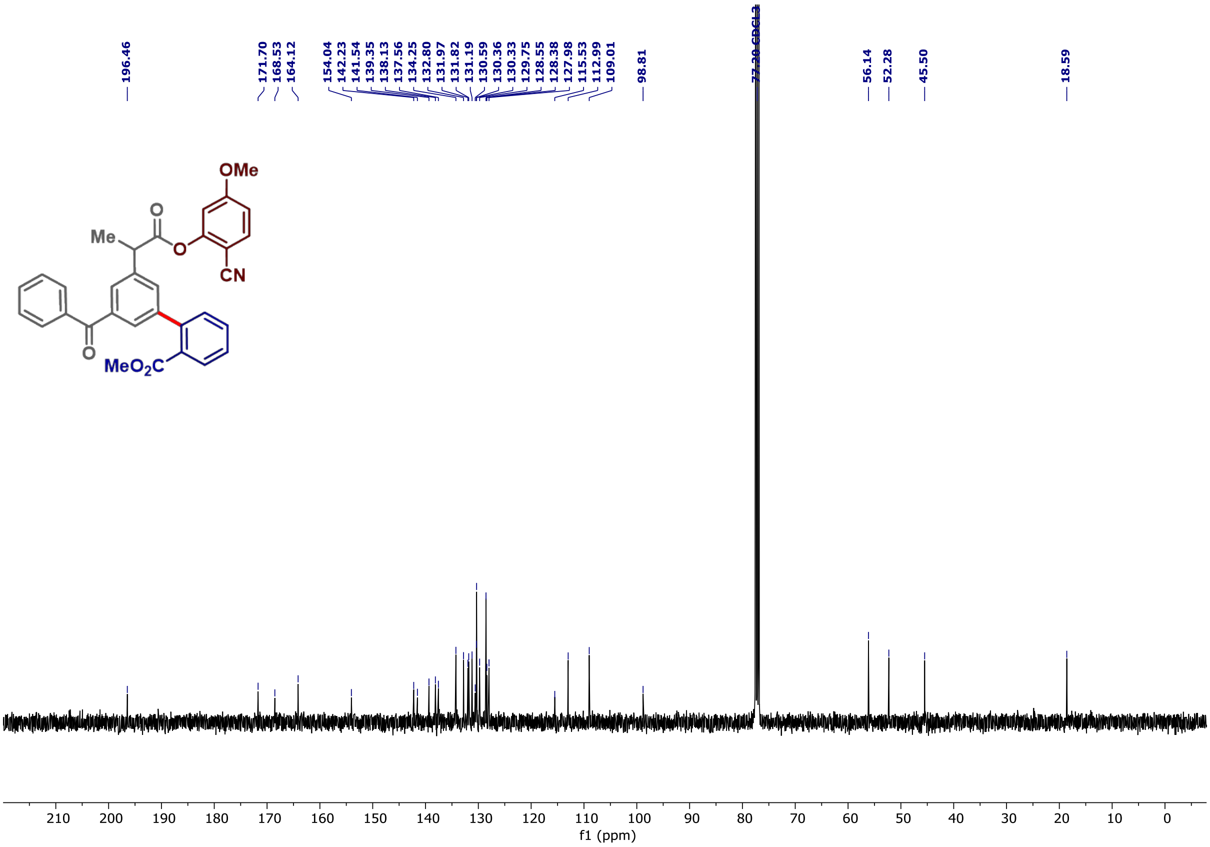
196.46
171.70 168.53 164.12
154.04 142.23 141.54 139.35 138.13 137.56 134.25 132.80 131.97 131.82 131.19 130.59 130.36 130.33 129.75 128.55 128.38 127.98 115.53 112.99 109.01
98.81
77.20 CDCL3
56.14 52.28 45.50
18.59
OMe
O
Me
O
CN
O
MeO2C
f1 (ppm)

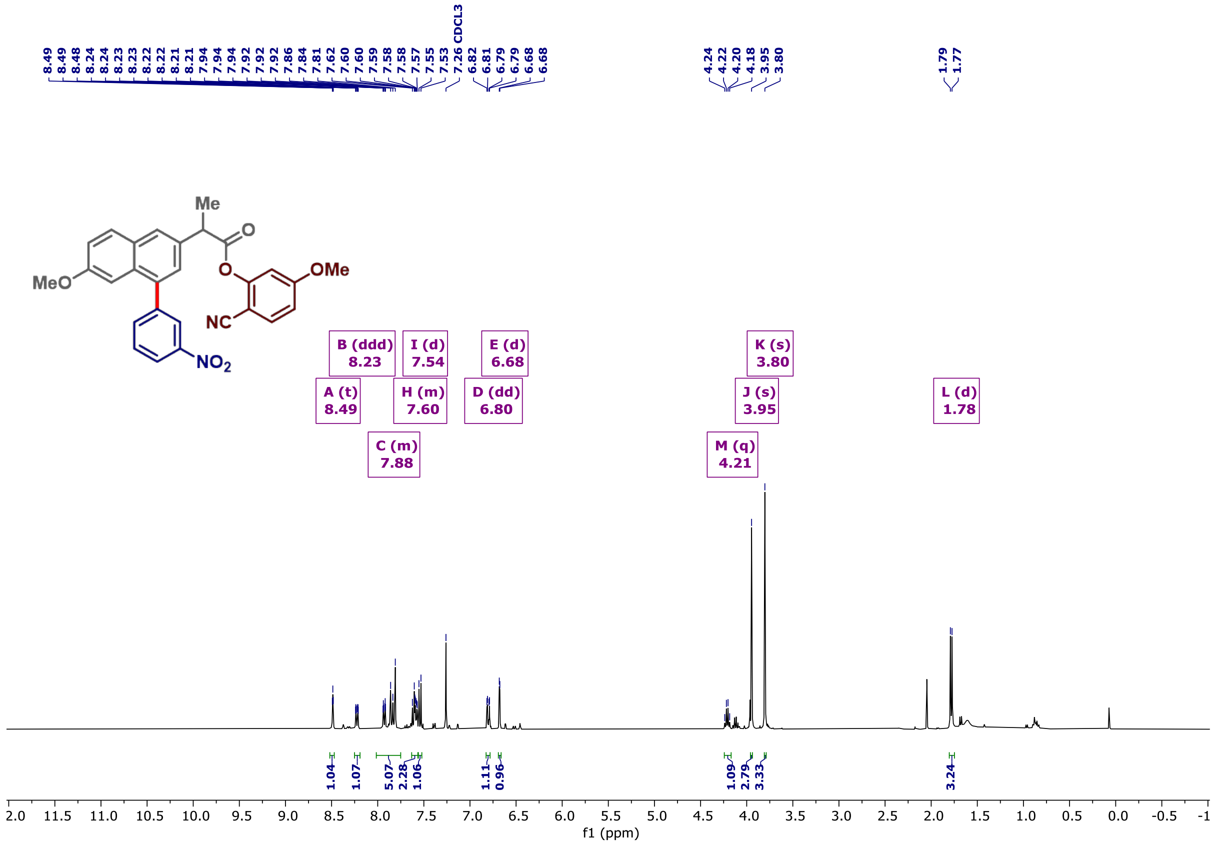
8.49 8.49 8.48 8.24 8.24 8.23 8.23 8.22 8.22 8.21 8.21 7.94 7.94 7.92 7.92 7.92 7.86 7.84 7.81 7.62 7.60 7.60 7.59 7.58 7.58 7.57 7.55 7.53 7.26 CDCL3 6.82 6.81 6.79 6.79 6.68 6.68
4.24 4.22 4.20 4.18 3.95 3.80
1.79 1.77
Me
O
MeO
O
OMe
NC
NO2
B (ddd) 8.23
I (d) 7.54
E (d) 6.68
K (s) 3.80
A (t) 8.49
H (m) 7.60
D (dd) 6.80
J (s) 3.95
L (d) 1.78
C (m) 7.88
M (q) 4.21
1.04 1.07 5.07 2.28 1.06 1.11 0.96 1.09 2.79 3.33 3.24
f1 (ppm)

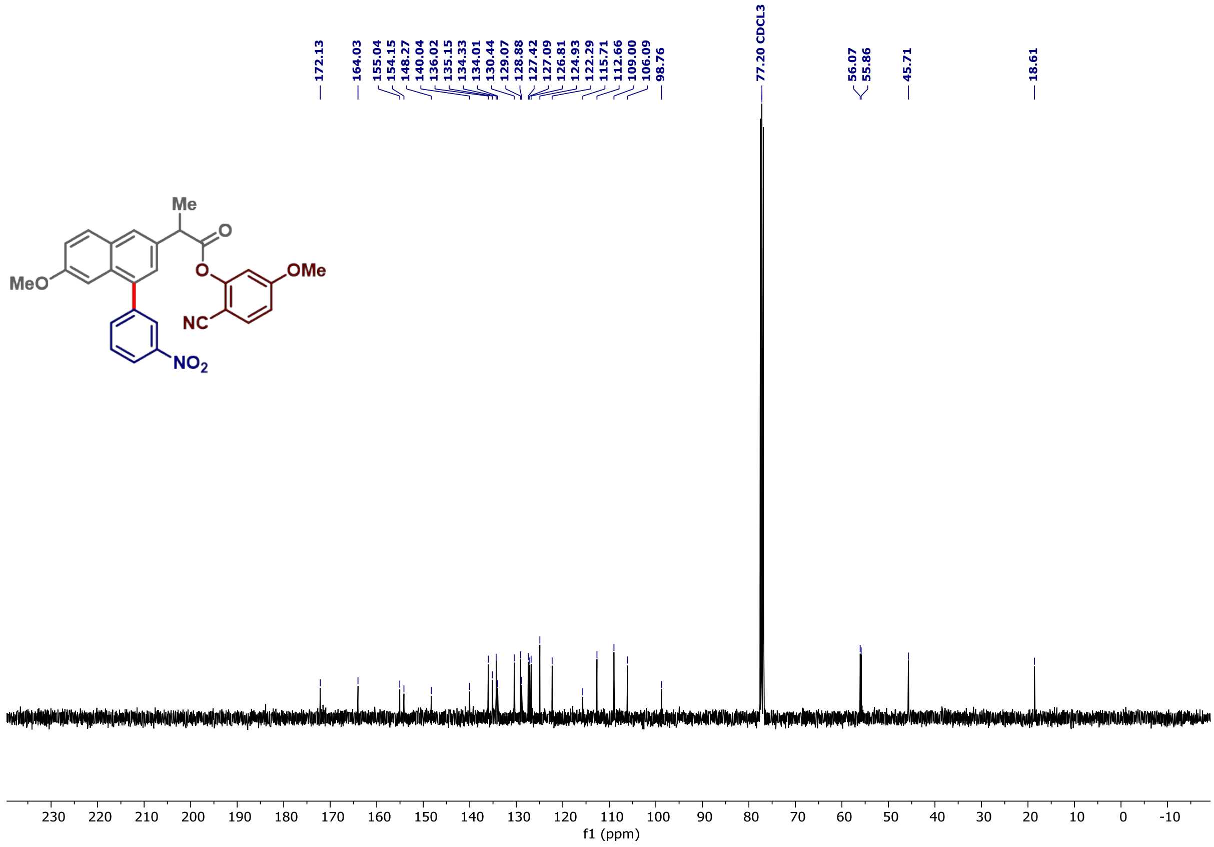
172.13 164.03 155.04 154.15 148.27 140.04 136.02 135.15 134.33 134.01 130.44 129.07 128.88 127.42 127.09 126.81 124.93 122.29 115.71 112.66 109.00 106.09 98.76
77.20 CDCL3
56.07 55.86 45.71 18.61
Me
O
MeO
O
OMe
NC
NO2
f1 (ppm)

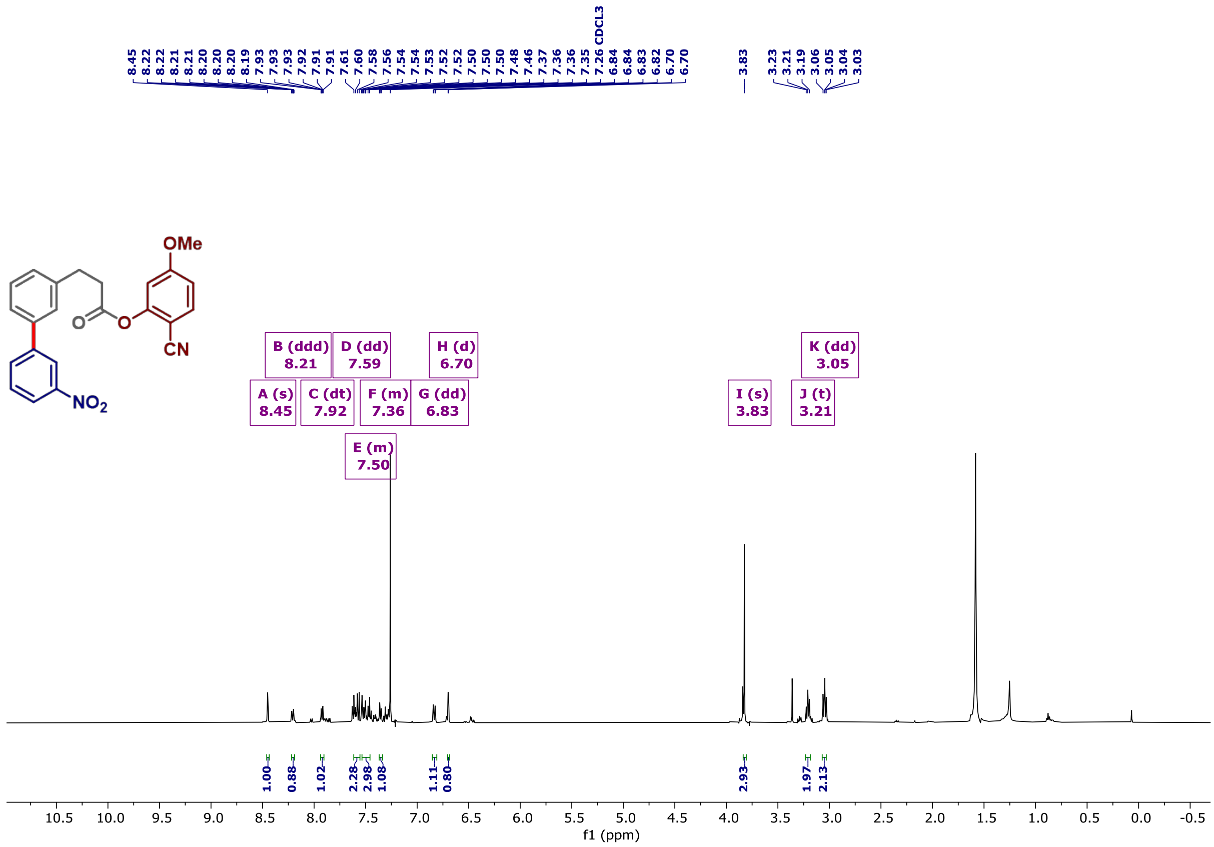
8.45 8.22 8.22 8.21 8.21 8.20 8.20 8.20 8.19 7.93 7.93 7.93 7.92 7.92 7.91 7.91 7.61 7.60 7.58 7.56 7.54 7.54 7.53 7.52 7.52 7.50 7.50 7.50 7.48 7.46 7.37 7.36 7.36 7.35 7.26 CDCL3 6.84 6.84 6.83 6.82 6.70 6.70
3.83
3.23 3.21 3.19 3.06 3.05 3.04 3.03
OMe
O
O
CN
NO2
A (s) 8.45
B (ddd) 8.21
C (dt) 7.92
D (dd) 7.59
E (m) 7.50
F (m) 7.36
G (dd) 6.83
H (d) 6.70
I (s) 3.83
J (t) 3.21
K (dd) 3.05
1.00 0.88 1.02 2.28 2.98 1.08 1.11 0.80 2.93 1.97 2.13
f1 (ppm)

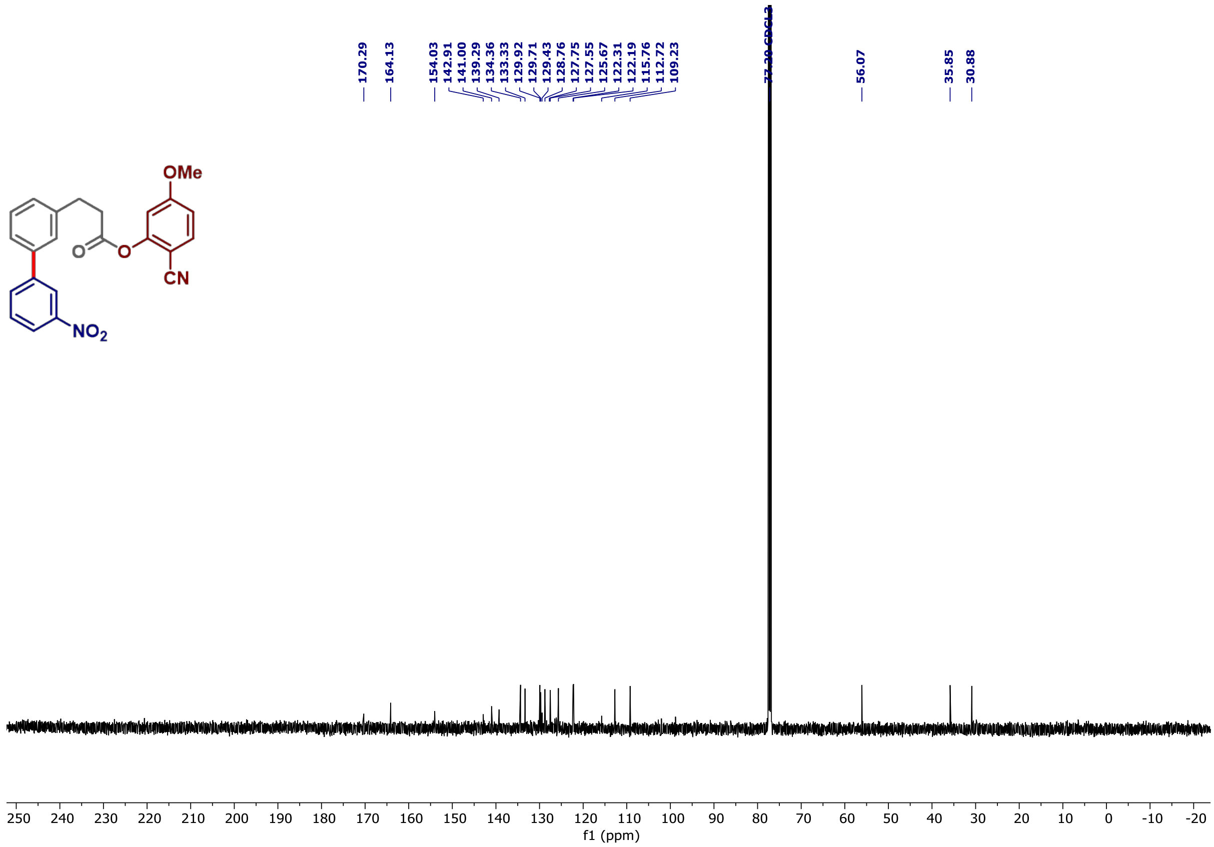
170.29
164.13
154.03 142.91 141.00 139.29 134.36 133.33 129.92 129.71 129.43 128.76 127.75 127.55 125.67 122.31 122.19 115.76 112.72 109.23
77.20 CDCL3
56.07
35.85 30.88
OMe
O
O
CN
NO2
f1 (ppm)

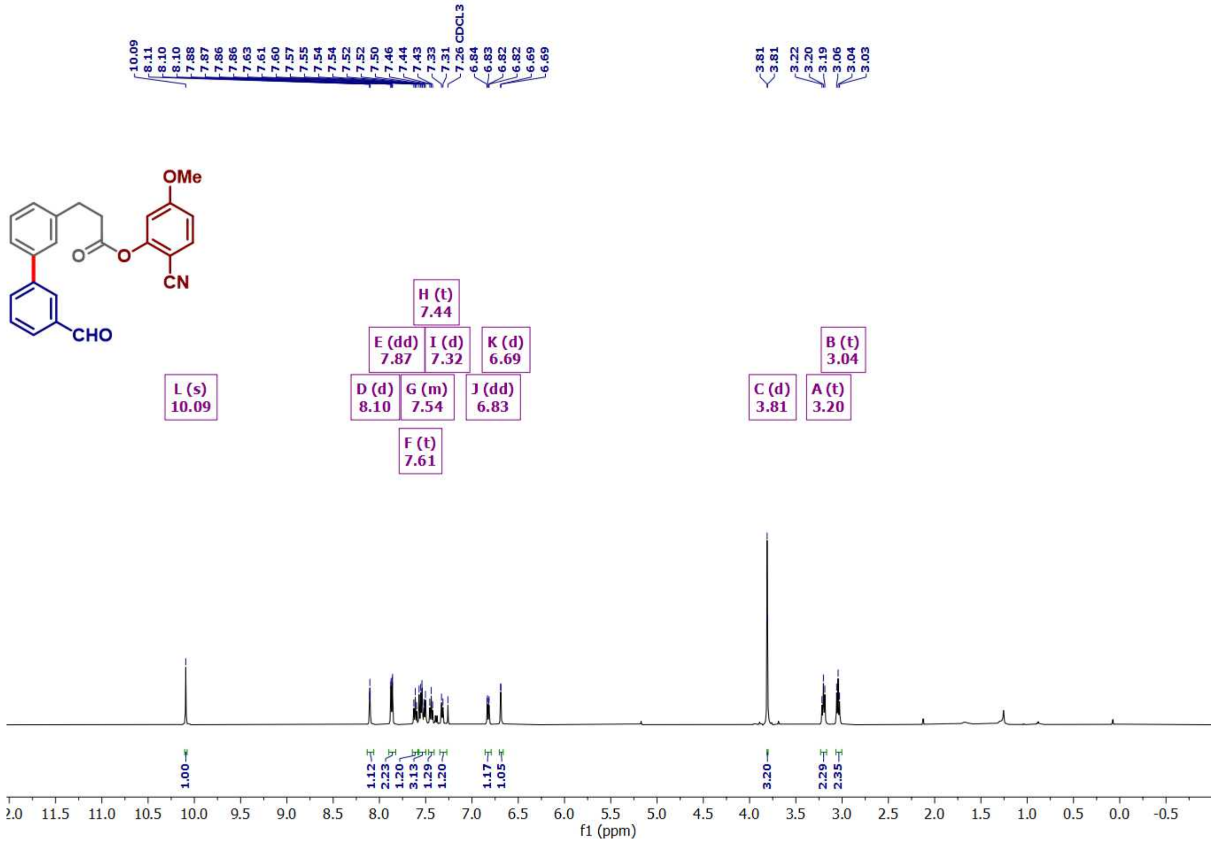
10.09 8.11 8.10 8.10 7.88 7.87 7.86 7.86 7.63 7.61 7.60 7.57 7.55 7.54 7.54 7.52 7.52 7.50 7.46 7.44 7.43 7.33 7.31 7.26 CDCL3 6.84 6.83 6.82 6.82 6.69 6.69
3.81 3.81 3.22 3.20 3.19 3.06 3.04 3.03
OMe
CN
CHO
H (t) 7.44
E (dd) 7.87
I (d) 7.32
K (d) 6.69
B (t) 3.04
L (s) 10.09
D (d) 8.10
G (m) 7.54
J (dd) 6.83
C (d) 3.81
A (t) 3.20
F (t) 7.61
1.00 1.12 2.23 1.20 3.13 1.29 1.20 1.17 1.05 3.20 2.29 2.35
f1 (ppm)

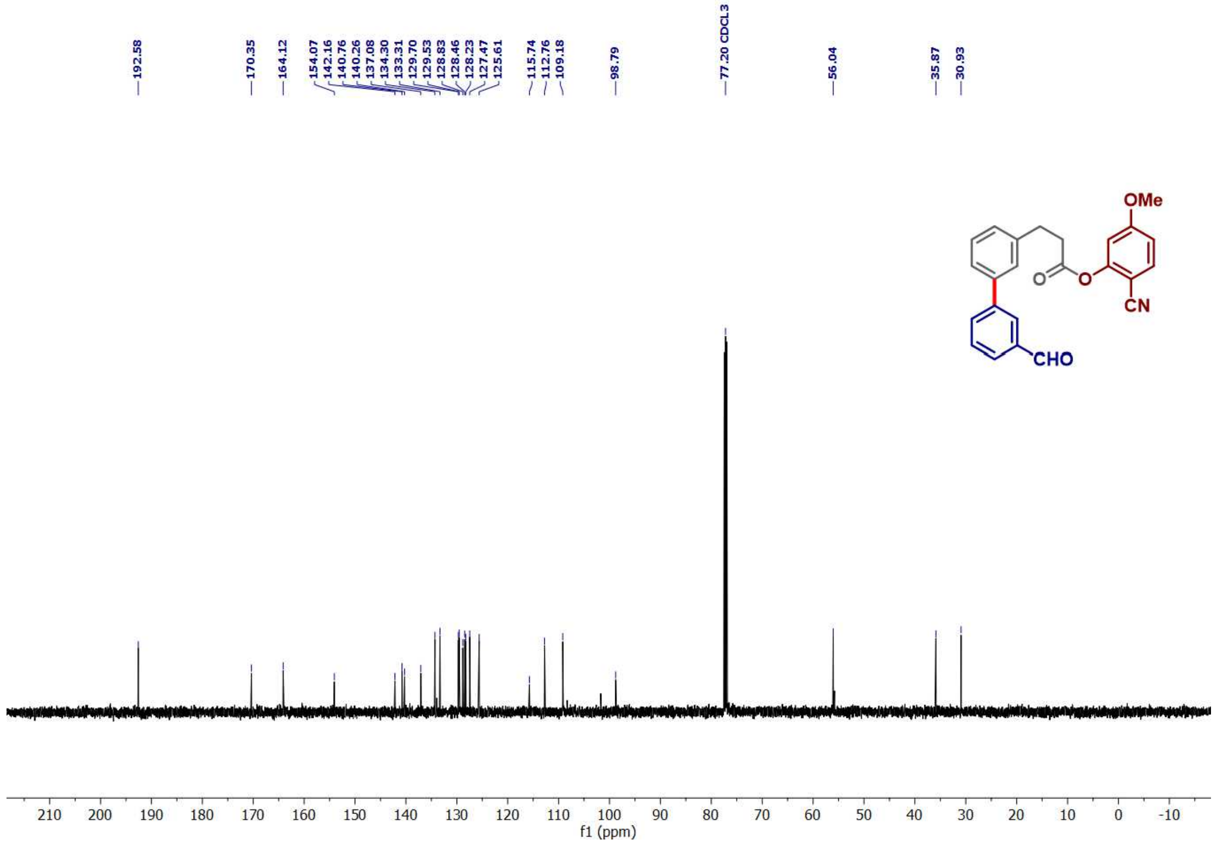
192.58 170.35 164.12 154.07 142.16 140.76 140.26 137.08 134.30 133.31 129.70 129.53 128.83 128.46 128.23 127.47 125.61 115.74 112.76 109.18 98.79 77.20 CDCL3 56.04 35.87 30.93
OMe
CN
CHO
f1 (ppm)

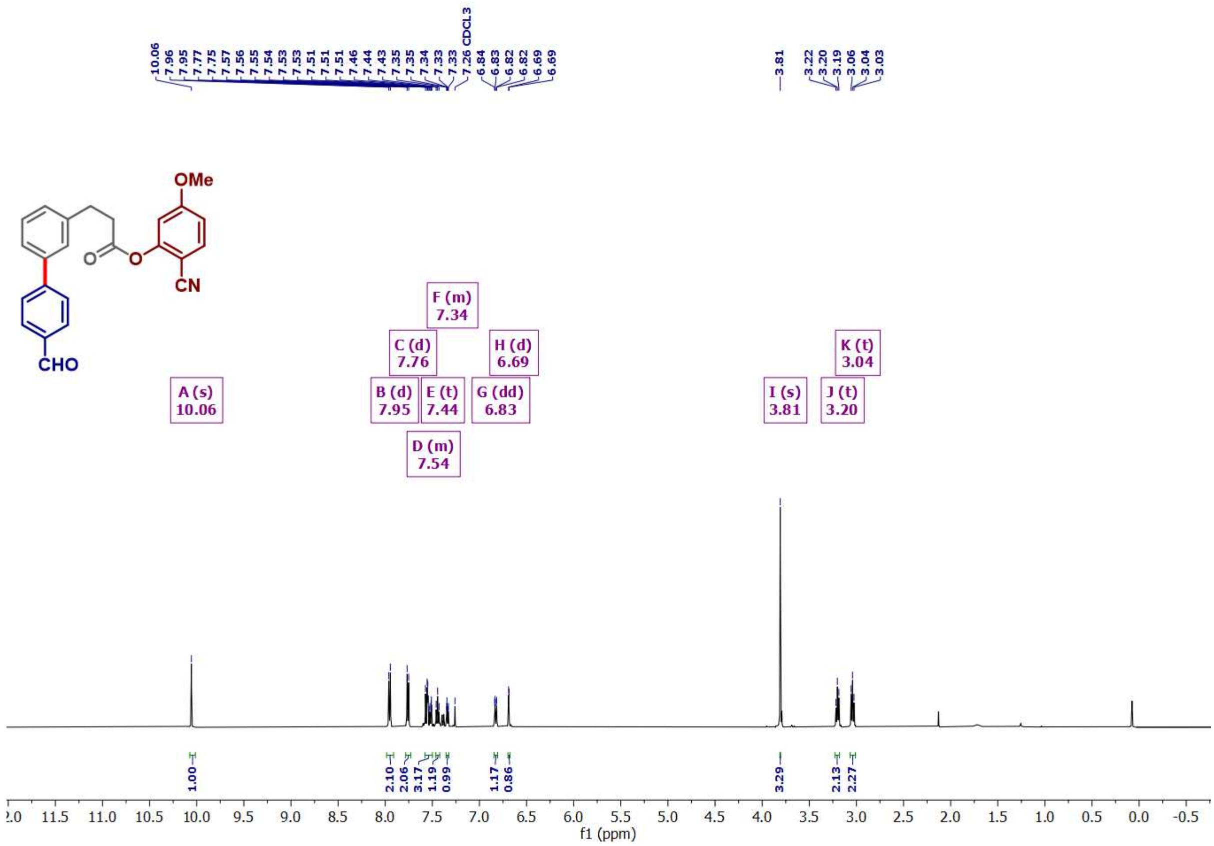
10.06 7.96 7.95 7.77 7.75 7.57 7.56 7.55 7.54 7.53 7.53 7.51 7.51 7.51 7.46 7.44 7.43 7.35 7.35 7.34 7.33 7.33 7.26 CDCL3 6.84 6.83 6.82 6.82 6.69 6.69
3.81 3.22 3.20 3.19 3.06 3.04 3.03
OMe
CN
CHO
A (s) 10.06
B (d) 7.95
C (d) 7.76
D (m) 7.54
E (t) 7.44
F (m) 7.34
G (dd) 6.83
H (d) 6.69
I (s) 3.81
J (t) 3.20
K (t) 3.04
1.00 2.10 2.06 3.17 1.19 0.99 1.17 0.86 3.29 2.13 2.27
f1 (ppm)

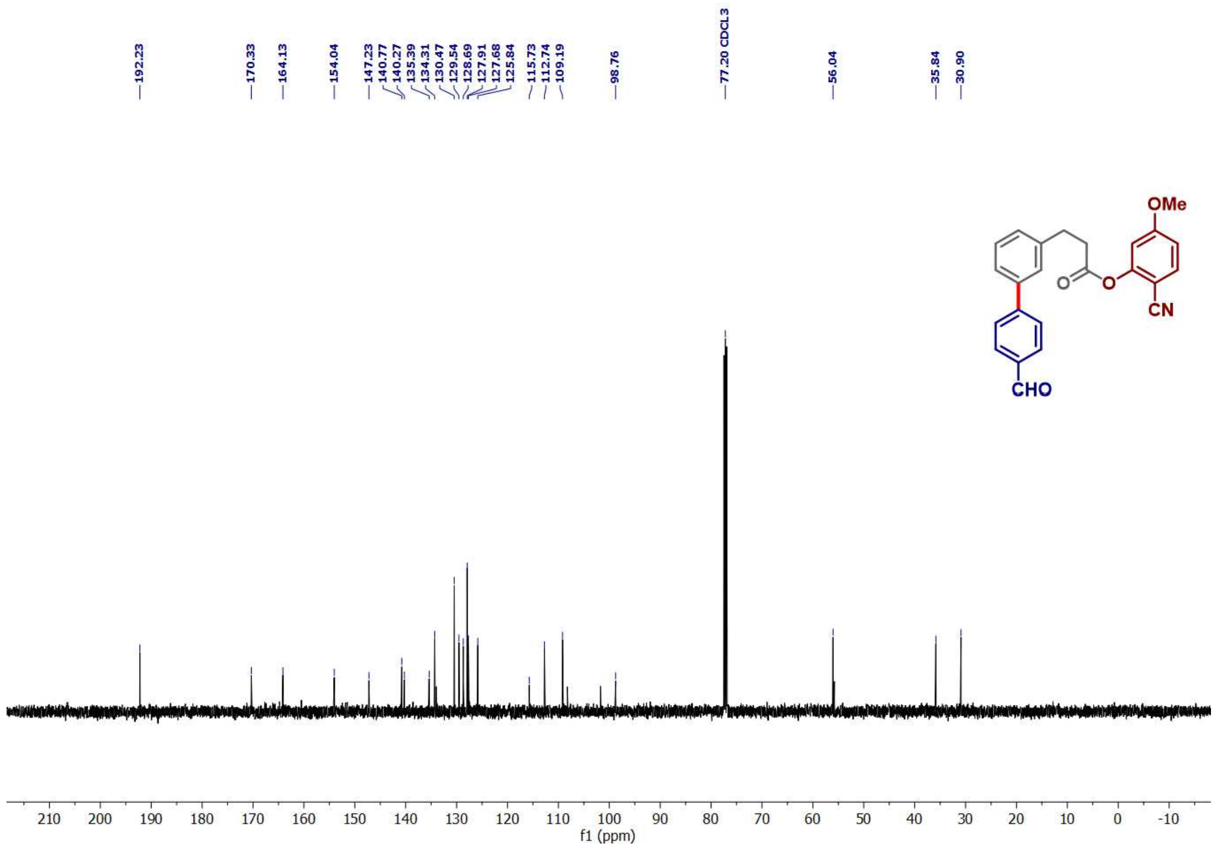
192.23 170.33 164.13 154.04 147.23 140.77 140.27 135.39 134.31 130.47 129.54 128.69 127.91 127.68 125.84 115.73 112.74 109.19 98.76 77.20 CDCL3 56.04 35.84 30.90
OMe
CN
CHO
f1 (ppm)

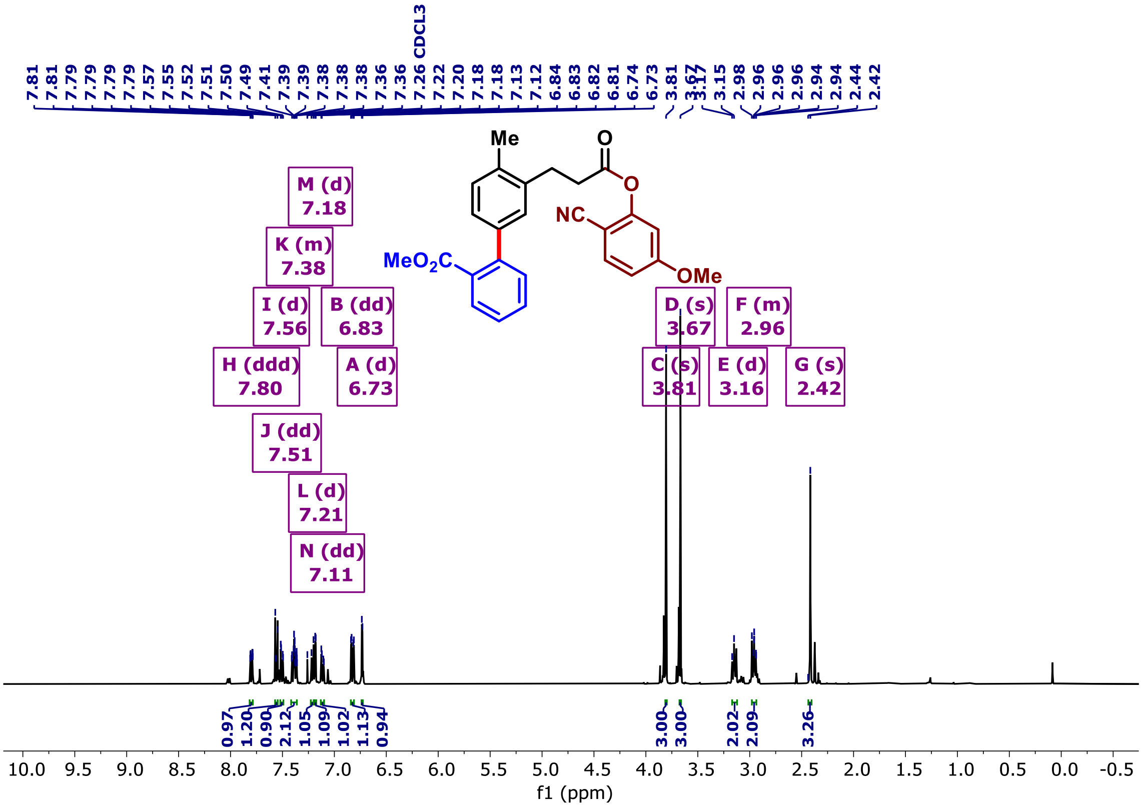
7.81
7.81
7.79
7.79
7.79
7.79
7.57
7.55
7.52
7.51
7.50
7.49
7.41
7.39
7.39
7.38
7.38
7.38
7.36
7.36
7.26 CDCL3
7.22
7.20
7.18
7.18
7.13
7.12
6.84
6.83
6.82
6.81
6.74
6.73
3.81
3.67
3.17
3.15
2.98
2.96
2.96
2.96
2.94
2.94
2.44
2.42
Me
O
O
NC
MeO2C
OMe
M (d) 7.18
K (m) 7.38
I (d) 7.56
B (dd) 6.83
H (ddd) 7.80
A (d) 6.73
J (dd) 7.51
L (d) 7.21
N (dd) 7.11
D (s) 3.67
F (m) 2.96
C (s) 3.81
E (d) 3.16
G (s) 2.42
0.97
1.20
0.90
2.12
1.05
1.09
1.02
1.13
0.94
3.00
3.00
2.02
2.09
3.26
10.0 9.5 9.0 8.5 8.0 7.5 7.0 6.5 6.0 5.5 5.0 4.5 4.0 3.5 3.0 2.5 2.0 1.5 1.0 0.5 0.0 -0.5
f1 (ppm)

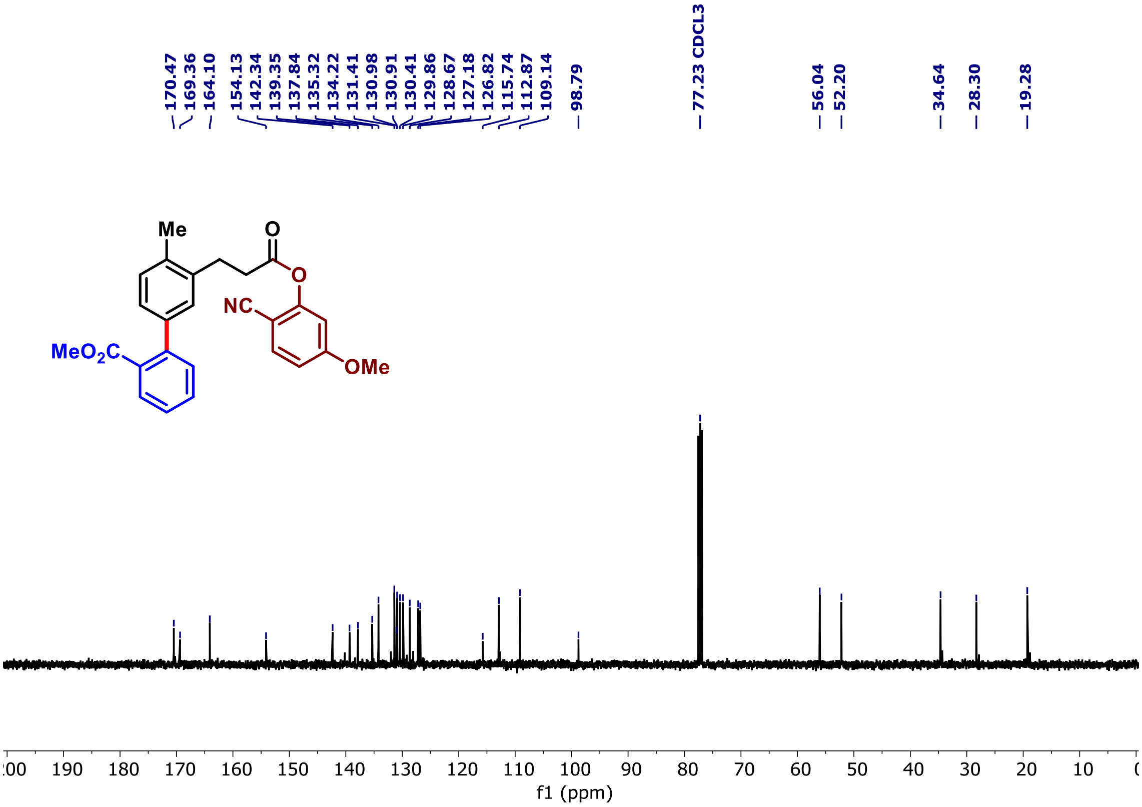
170.47
169.36
164.10
154.13
142.34
139.35
137.84
135.32
134.22
131.41
130.98
130.91
130.41
129.86
128.67
127.18
126.82
115.74
112.87
109.14
98.79
77.23 CDCL3
56.04
52.20
34.64
28.30
19.28
Me
O
O
NC
MeO2C
OMe
190 180 170 160 150 140 130 120 110 100 90 80 70 60 50 40 30 20 10
f1 (ppm)

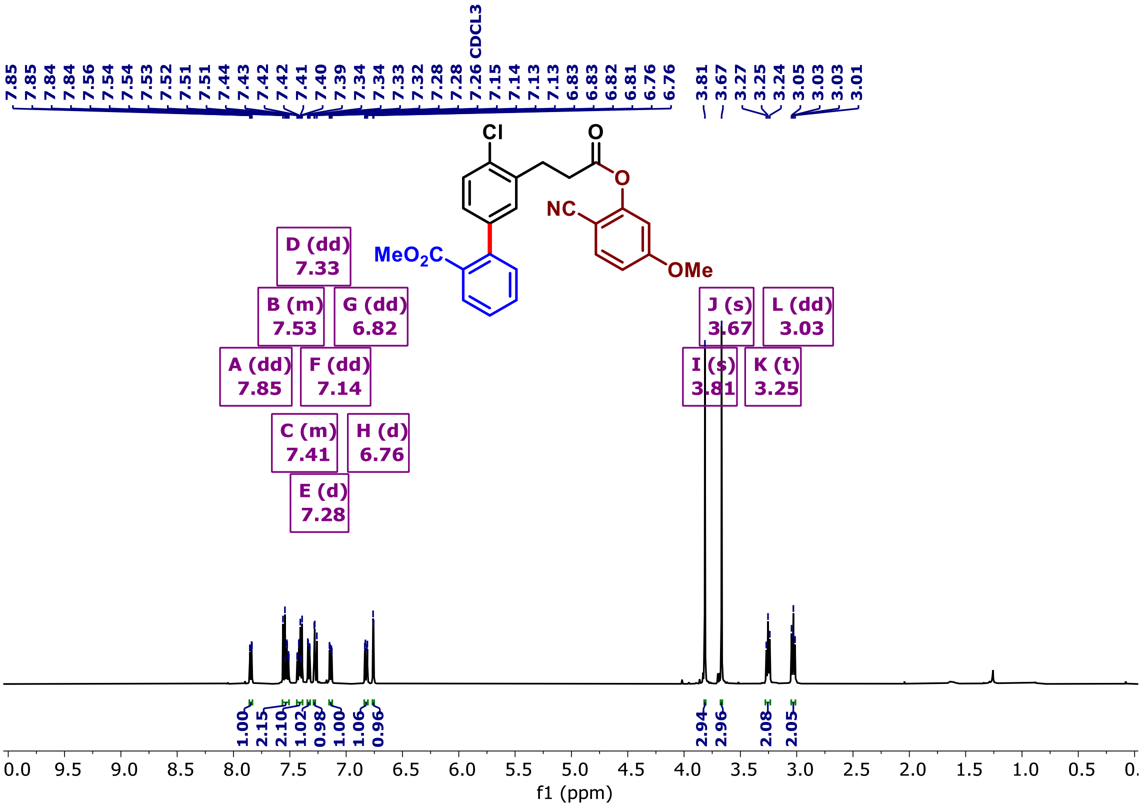
7.85 7.85 7.84 7.84 7.56 7.54 7.54 7.53 7.52 7.51 7.51 7.44 7.43 7.42 7.42 7.41 7.40 7.39 7.34 7.34 7.33 7.32 7.28 7.28 7.26 CDCL3 7.15 7.14 7.13 7.13 6.83 6.83 6.82 6.81 6.76 6.76
3.81 3.67 3.27 3.25 3.24 3.05 3.03 3.03 3.01
Cl
O
O
NC
MeO2C
OMe
D (dd) 7.33
B (m) 7.53
G (dd) 6.82
A (dd) 7.85
F (dd) 7.14
C (m) 7.41
H (d) 6.76
E (d) 7.28
J (s) 3.67
L (dd) 3.03
I (s) 3.81
K (t) 3.25
1.00 2.15 2.10 1.02 0.98 1.00 1.06 0.96
2.94 2.96 2.08 2.05
f1 (ppm)

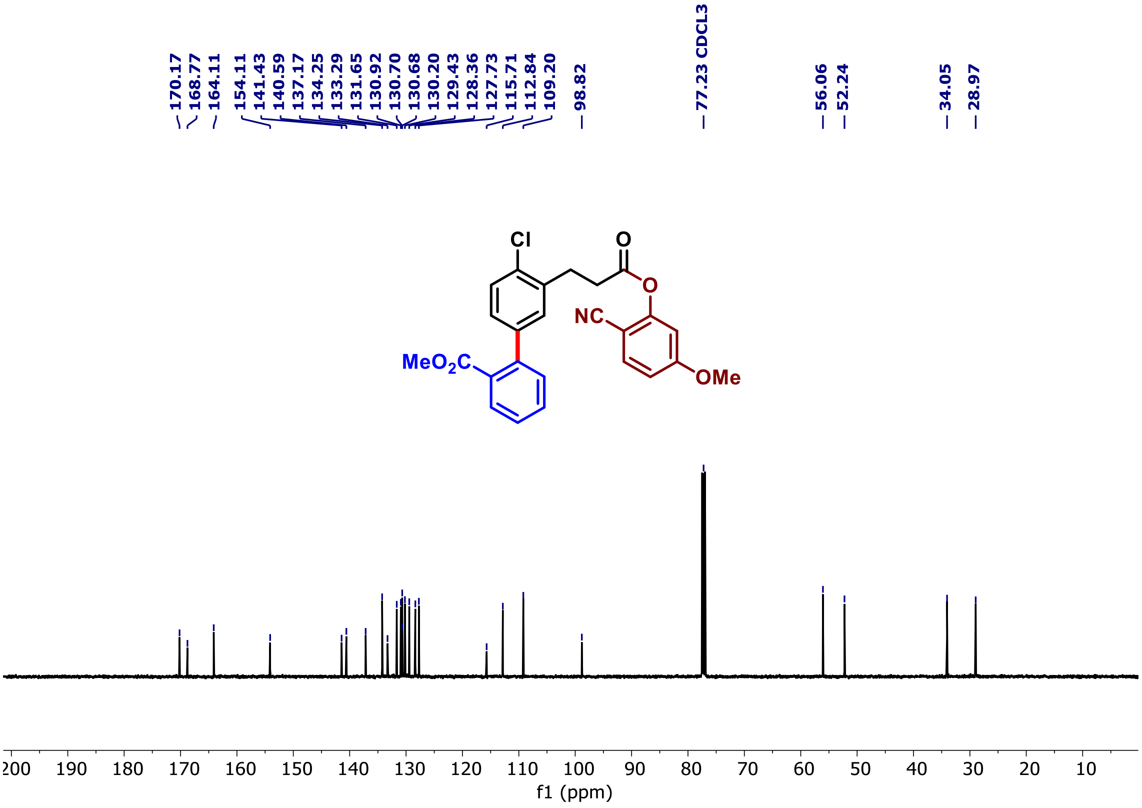
170.17 168.77 164.11 154.11 141.43 140.59 137.17 134.25 133.29 131.65 130.92 130.70 130.68 130.20 129.43 128.36 127.73 115.71 112.84 109.20 98.82
77.23 CDCL3
56.06 52.24
34.05 28.97
Cl
O
O
NC
MeO2C
OMe
f1 (ppm)

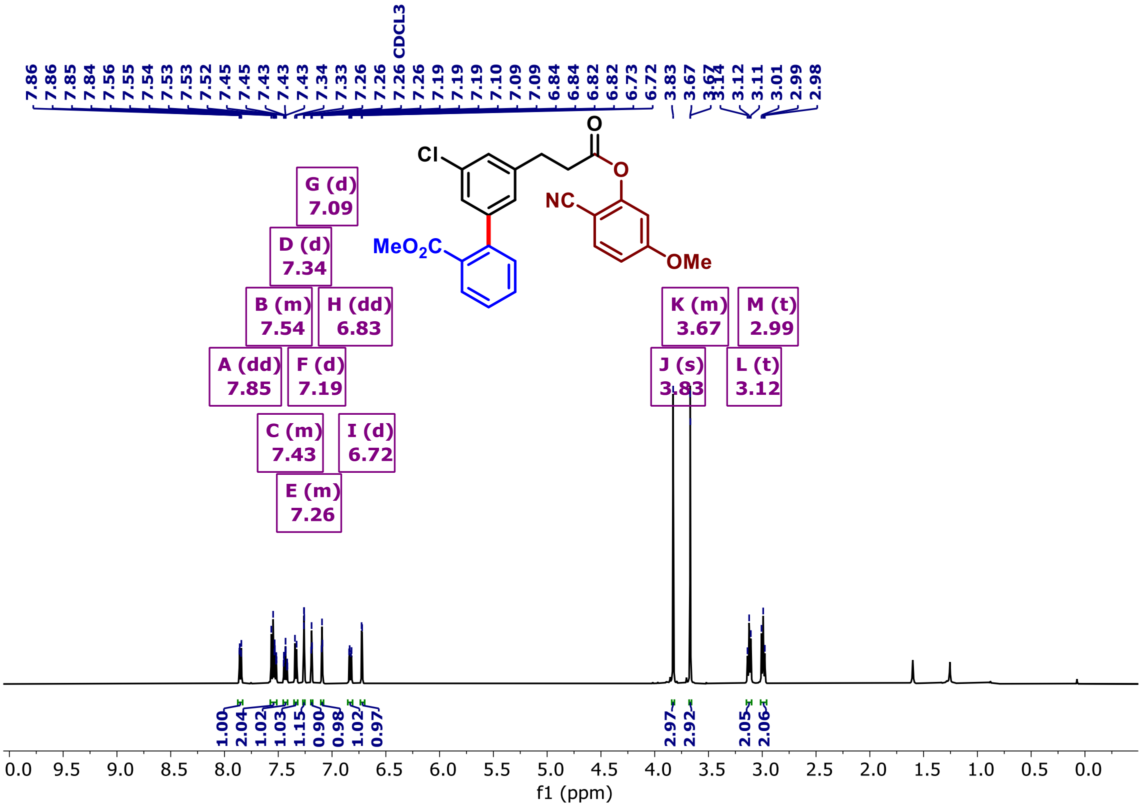

7.86 7.86 7.85 7.84 7.56 7.55 7.54 7.53 7.53 7.52 7.45 7.45 7.43 7.43 7.43 7.34 7.33 7.26 7.26 CDCL3 7.26 7.19 7.19 7.19 7.10 7.09 7.09 6.84 6.84 6.82 6.82 6.73 6.72 3.83 3.67 3.67 3.14 3.12 3.11 3.01 2.99 2.98
Cl
O
O
NC
MeO2C
OMe
G (d) 7.09
D (d) 7.34
B (m) 7.54
H (dd) 6.83
A (dd) 7.85
F (d) 7.19
C (m) 7.43
I (d) 6.72
E (m) 7.26
K (m) 3.67
M (t) 2.99
J (s) 3.83
L (t) 3.12
1.00 2.04 1.02 1.03 1.15 0.90 0.98 1.02 0.97 2.97 2.92 2.05 2.06
f1 (ppm)

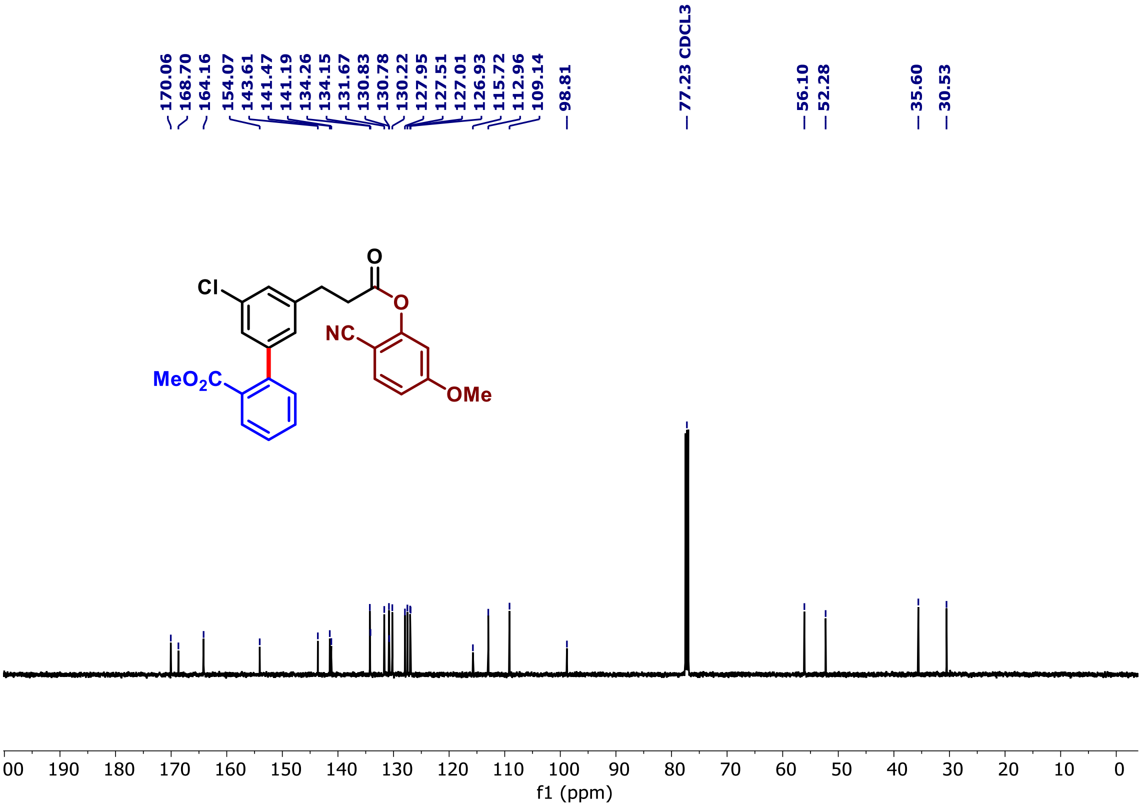

170.06 168.70 164.16 154.07 143.61 141.47 141.19 134.26 134.15 131.67 130.83 130.78 130.22 127.95 127.51 127.01 126.93 115.72 112.96 109.14 98.81
77.23 CDCL3
56.10 52.28
35.60 30.53
Cl
O
O
NC
MeO2C
OMe
f1 (ppm)

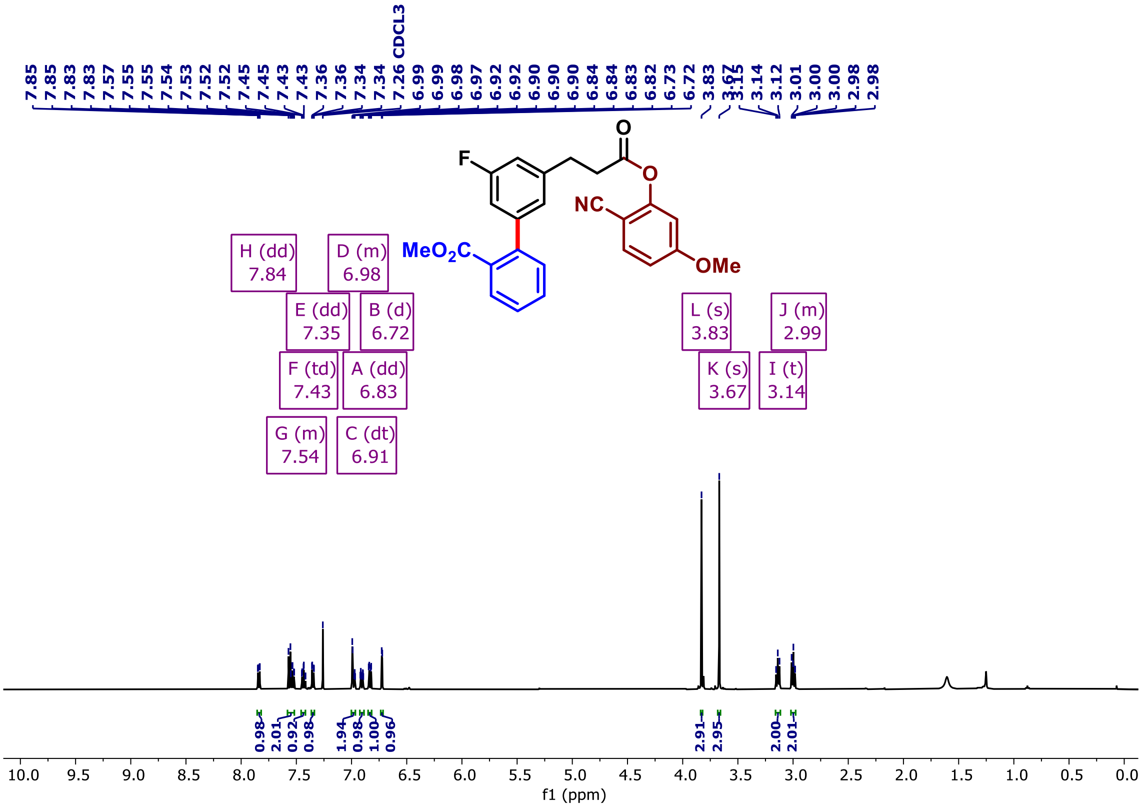

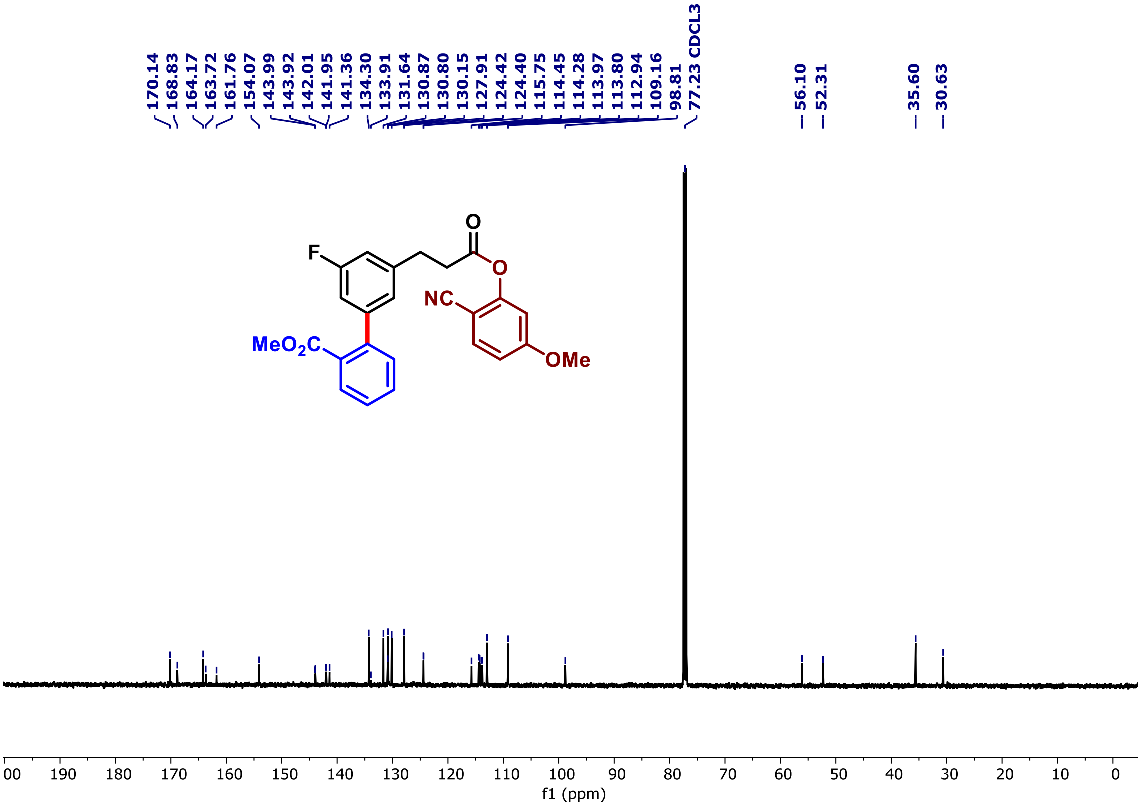

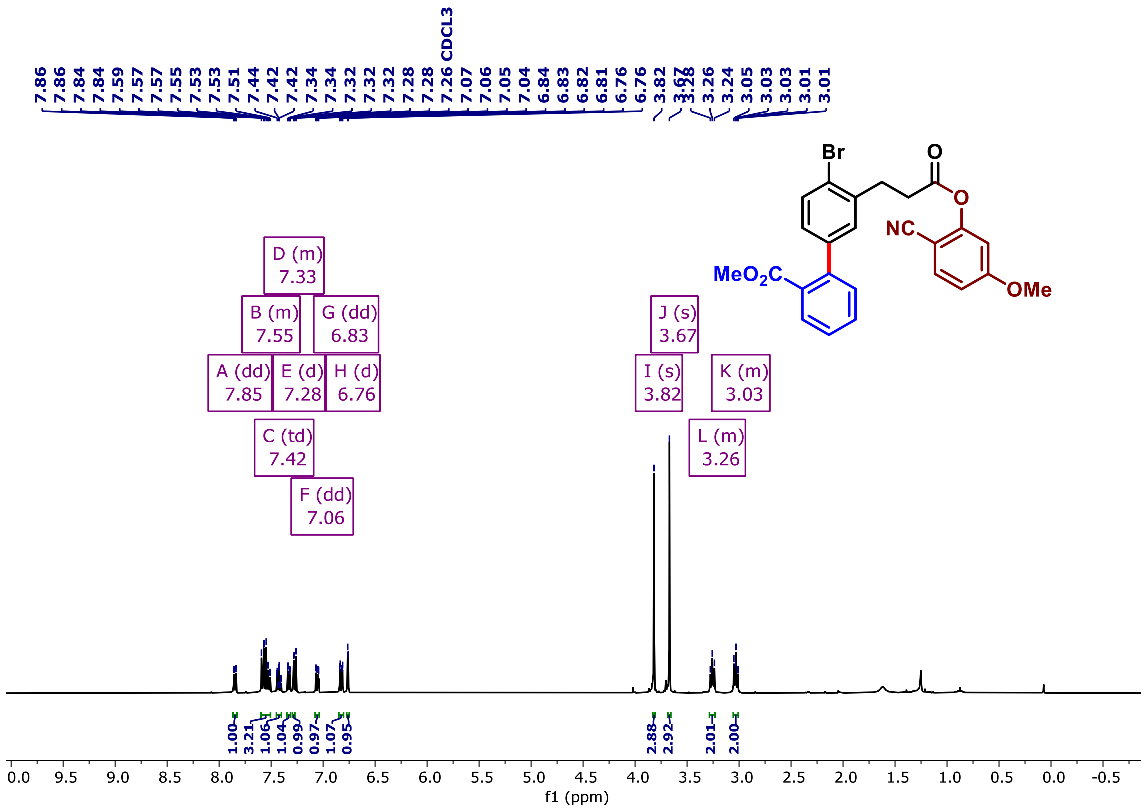
7.86
7.86
7.84
7.84
7.59
7.57
7.57
7.55
7.53
7.53
7.51
7.44
7.42
7.42
7.34
7.34
7.32
7.32
7.32
7.28
7.28
7.26 CDCL3
7.07
7.06
7.05
7.04
6.84
6.83
6.82
6.81
6.76
6.76
3.82
3.67
3.28
3.26
3.24
3.05
3.03
3.03
3.01
3.01
Br
O
O
NC
MeO2C
OMe
D (m) 7.33
B (m) 7.55
G (dd) 6.83
A (dd) 7.85
E (d) 7.28
H (d) 6.76
C (td) 7.42
F (dd) 7.06
J (s) 3.67
I (s) 3.82
K (m) 3.03
L (m) 3.26
1.00
3.21
1.06
1.04
0.99
0.97
1.07
0.95
2.88
2.92
2.01
2.00
f1 (ppm)

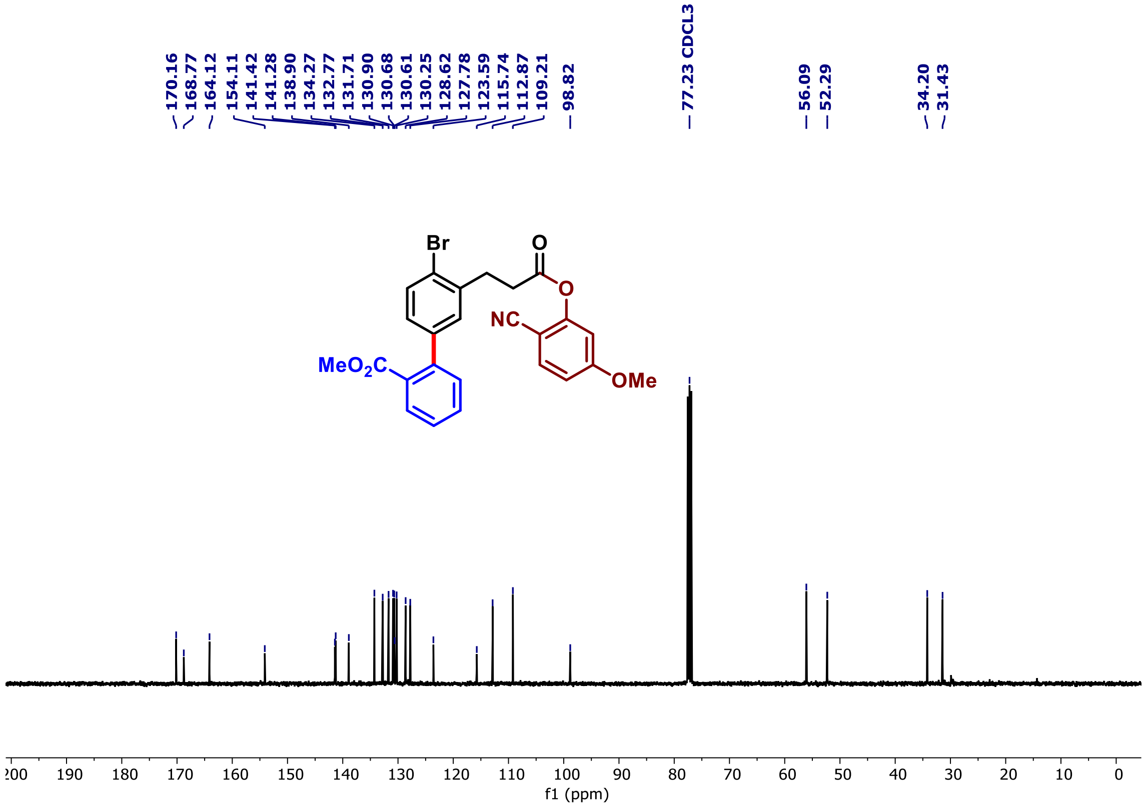
170.16
168.77
164.12
154.11
141.42
141.28
138.90
134.27
132.77
131.71
130.90
130.68
130.61
130.25
128.62
127.78
123.59
115.74
112.87
109.21
98.82
77.23 CDCL3
56.09
52.29
34.20
31.43
Br
O
O
NC
MeO2C
OMe
f1 (ppm)

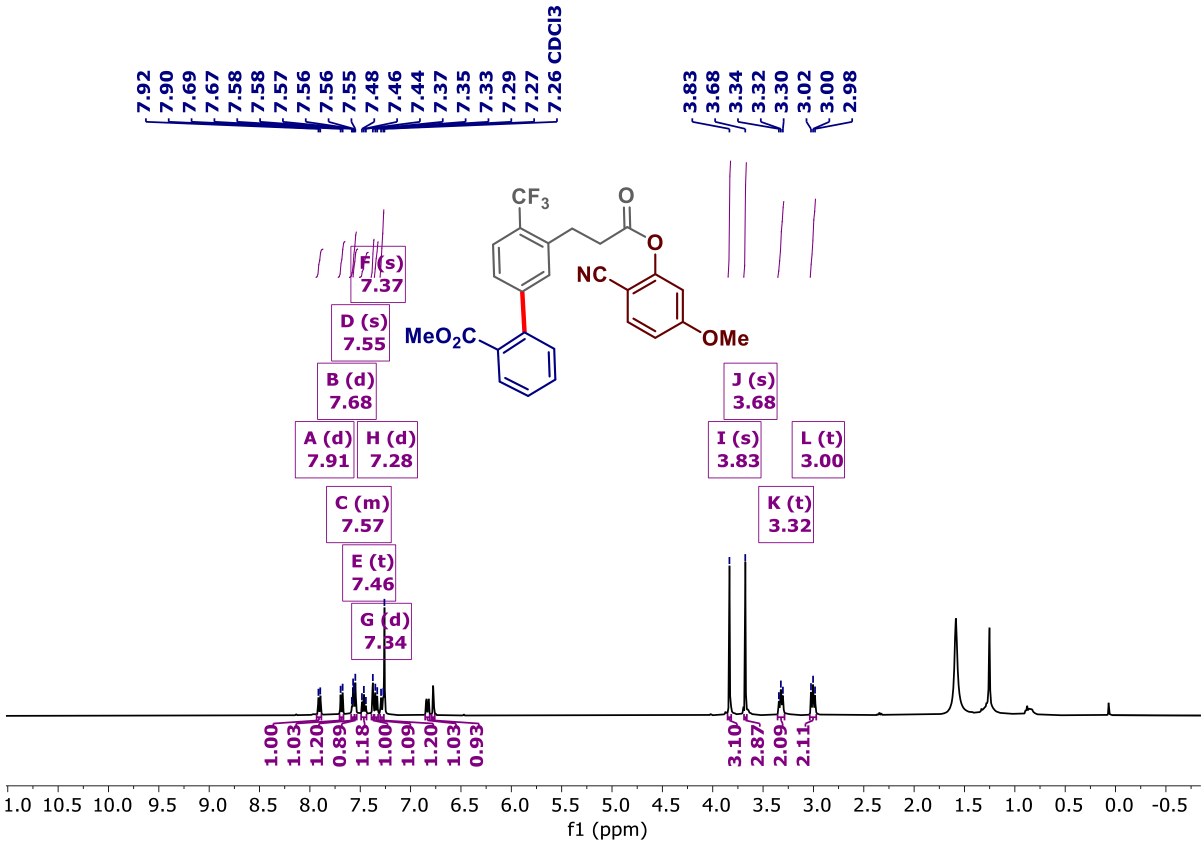

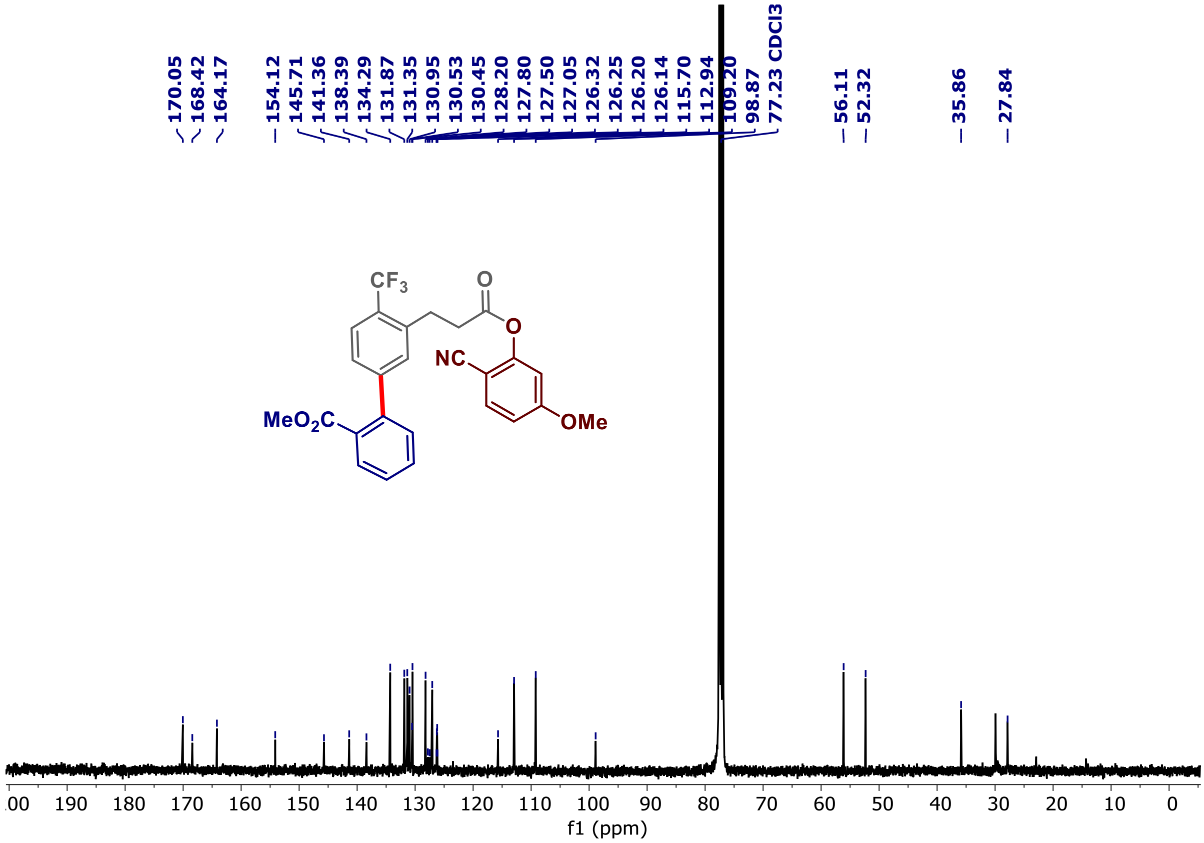

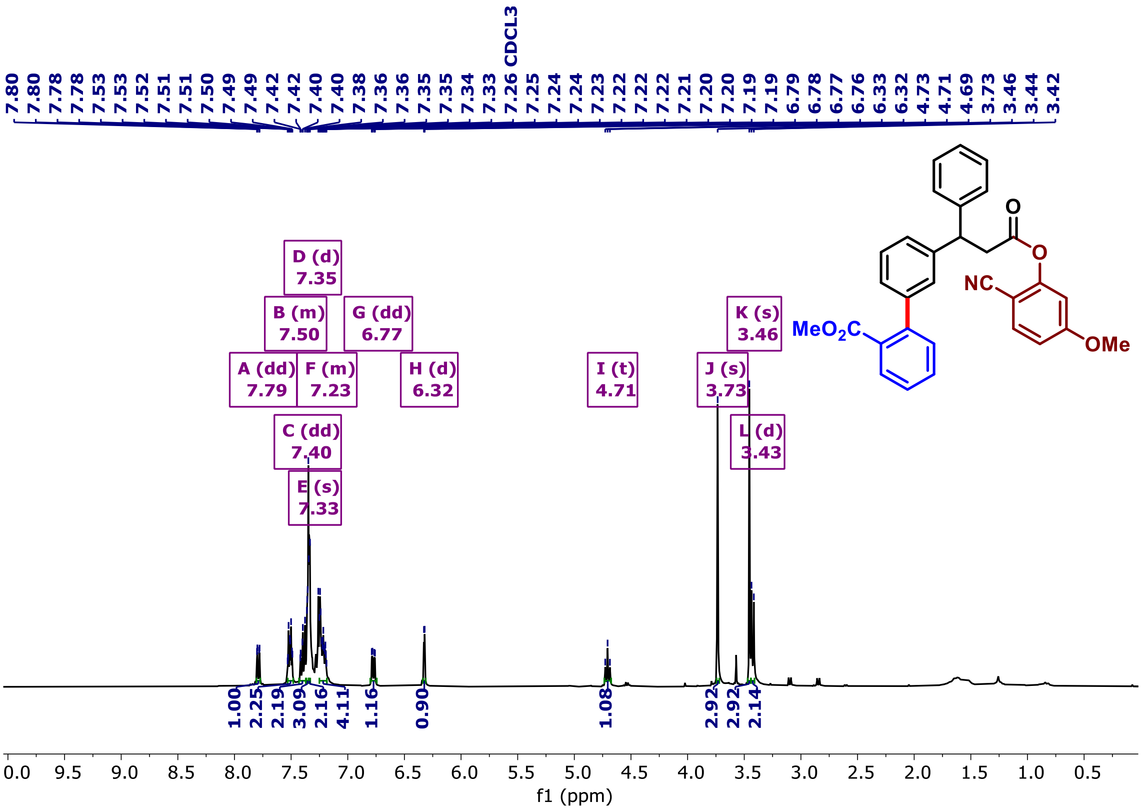

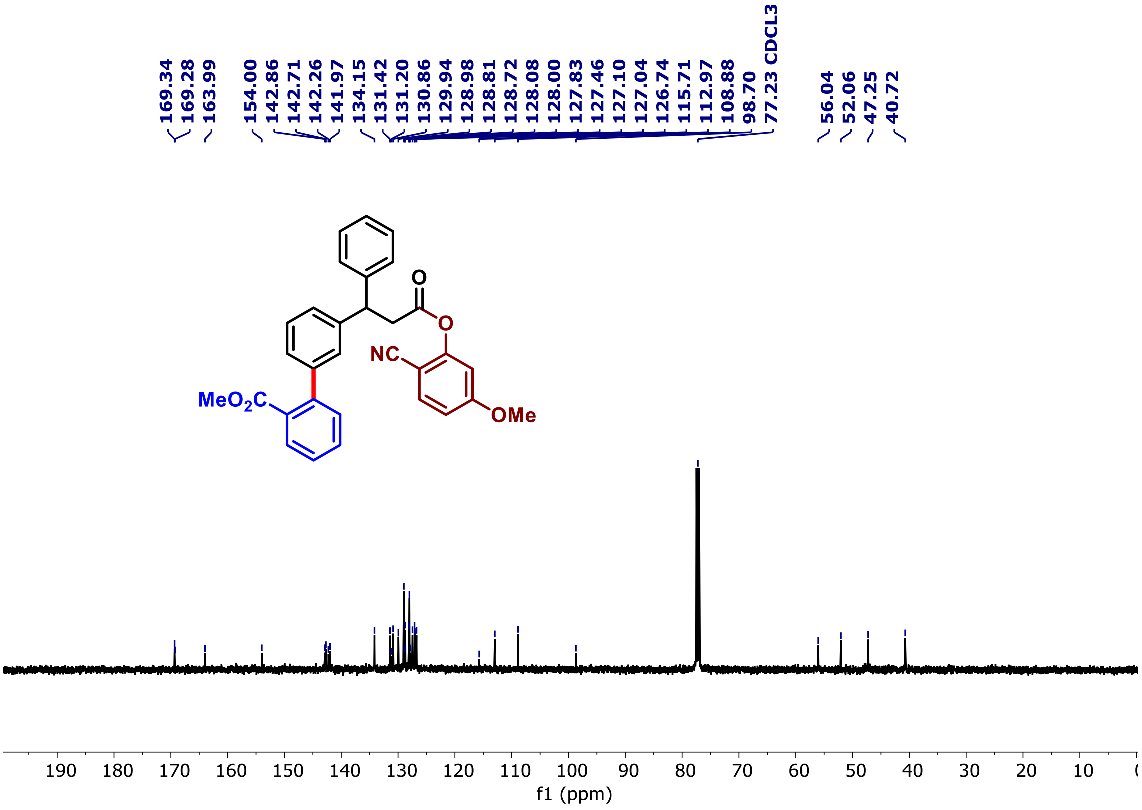

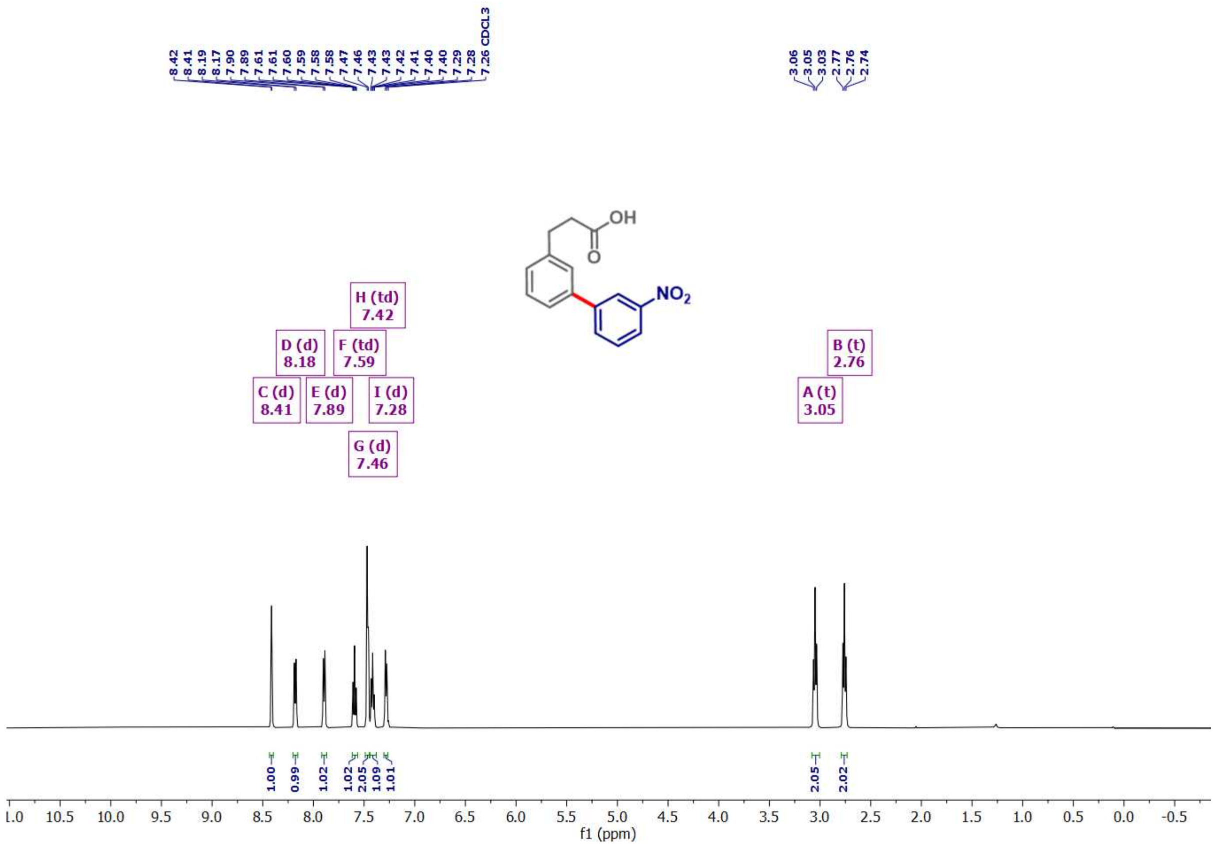
8.42
8.41
8.19
8.17
7.90
7.89
7.61
7.61
7.60
7.59
7.58
7.58
7.47
7.46
7.43
7.43
7.42
7.41
7.40
7.40
7.29
7.28
7.26 CDCL3
3.06
3.05
3.03
2.77
2.76
2.74
OH
O
NO2
H (td) 7.42
D (d) 8.18
F (td) 7.59
B (t) 2.76
C (d) 8.41
E (d) 7.89
I (d) 7.28
A (t) 3.05
G (d) 7.46
1.00
0.99
1.02
1.02
2.05
1.09
1.01
2.05
2.02
f1 (ppm)

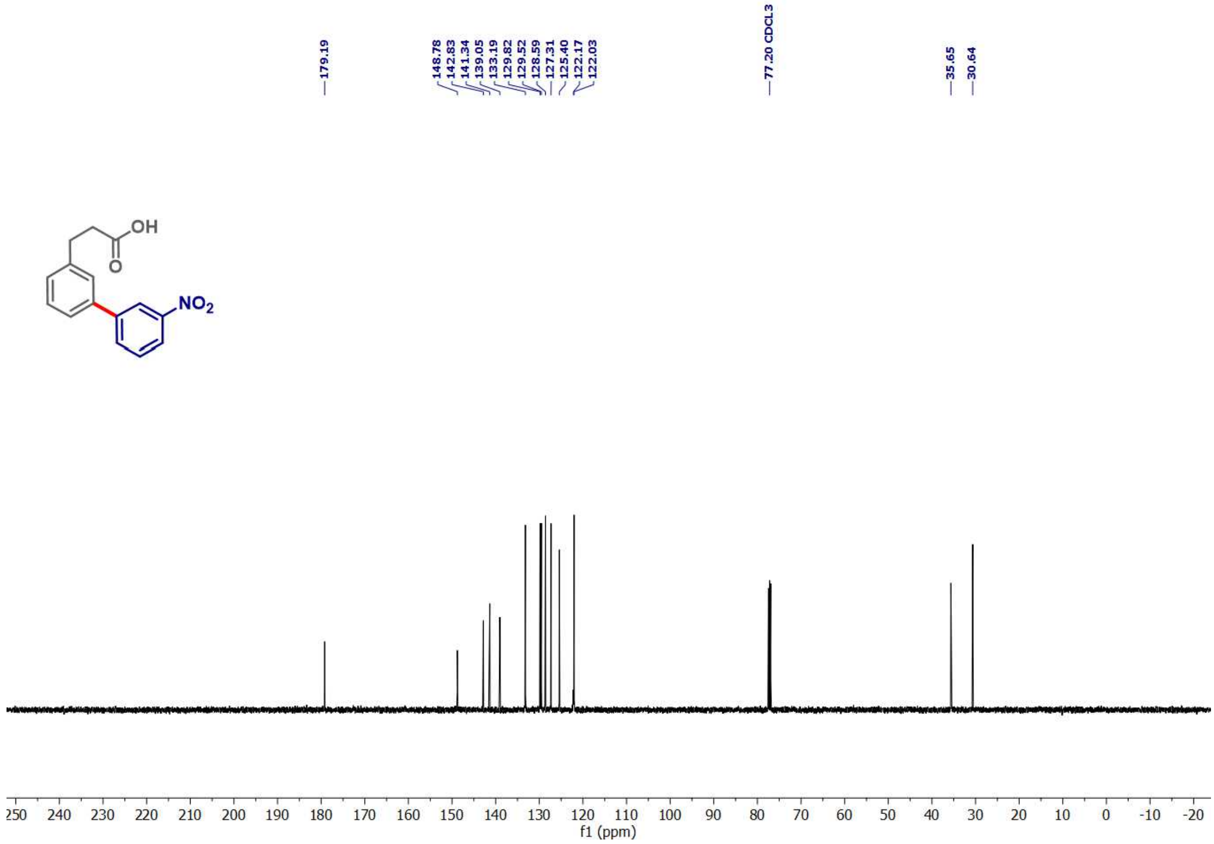
179.19
148.78
142.83
141.34
139.05
133.19
129.82
129.52
128.59
127.31
125.40
122.17
122.03
77.20 CDCL3
35.65
30.64
OH
O
NO2
f1 (ppm)

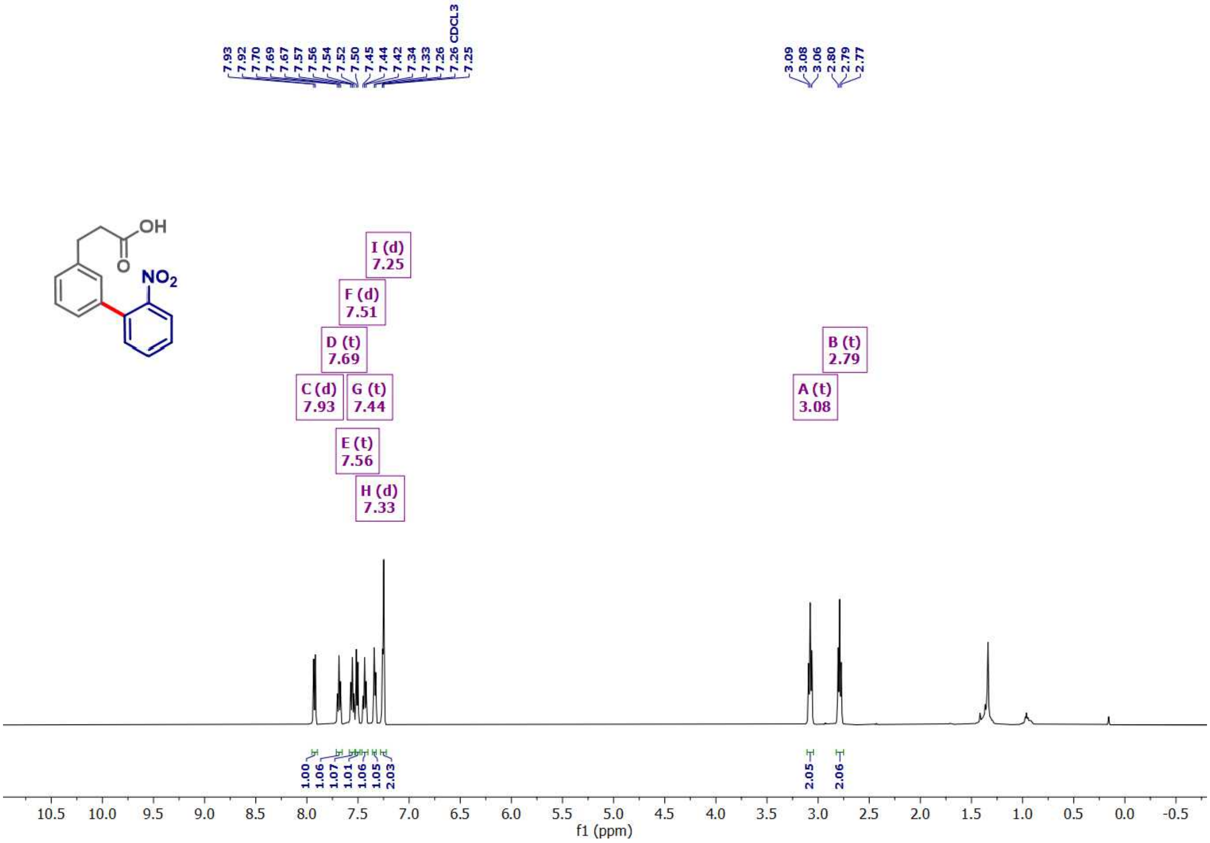

7.93
7.92
7.70
7.69
7.67
7.57
7.56
7.54
7.52
7.50
7.45
7.44
7.42
7.34
7.33
7.26 CDCL3
7.25
3.09
3.08
3.06
2.80
2.79
2.77
OH
O
NO2
I (d) 7.25
F (d) 7.51
D (t) 7.69
C (d) 7.93
G (t) 7.44
E (t) 7.56
H (d) 7.33
B (t) 2.79
A (t) 3.08
1.00
1.06
1.07
1.01
1.06
1.05
2.03
2.05
2.06
10.5 10.0 9.5 9.0 8.5 8.0 7.5 7.0 6.5 6.0 5.5 5.0 4.5 4.0 3.5 3.0 2.5 2.0 1.5 1.0 0.5 0.0 -0.5
f1 (ppm)

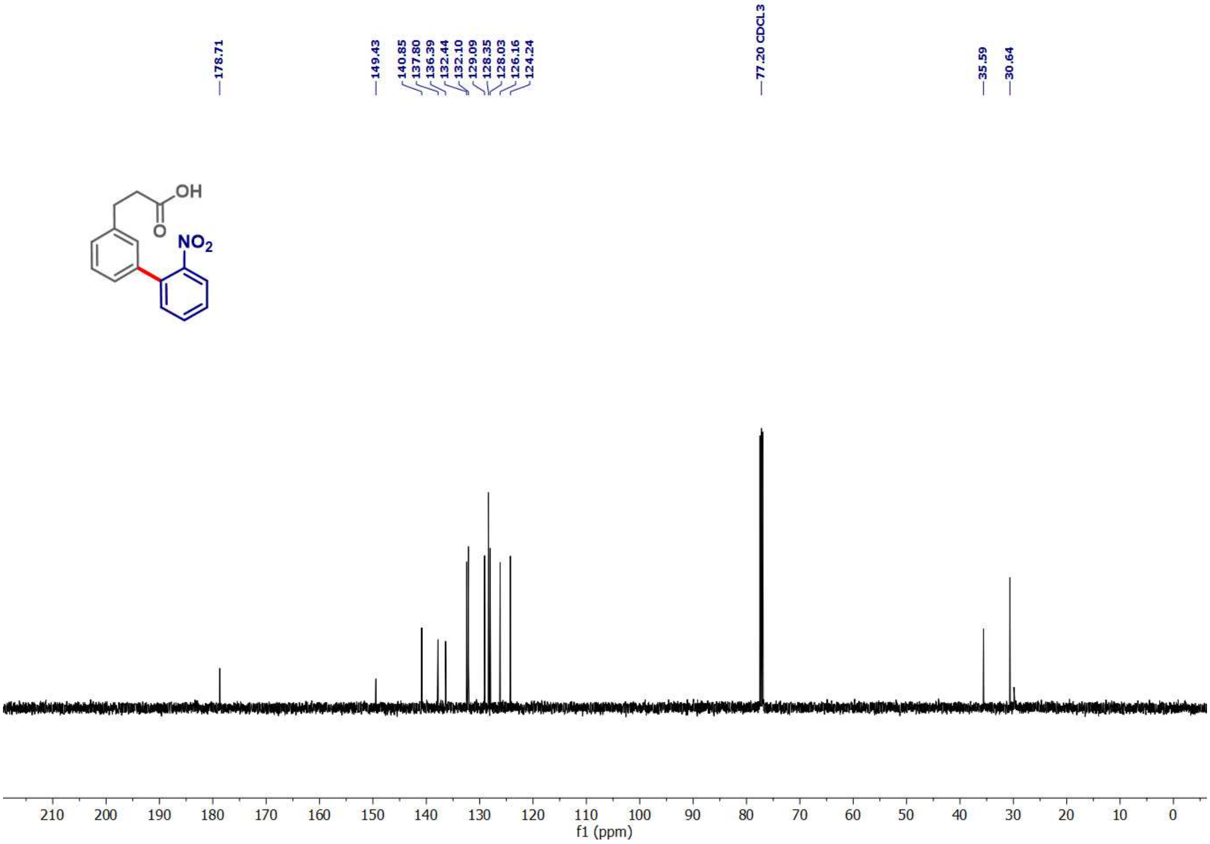

178.71
149.43
140.85
137.80
136.39
132.44
132.10
129.09
128.35
128.03
126.16
124.24
77.20 CDCL3
35.59
30.64
OH
O
NO2
210 200 190 180 170 160 150 140 130 120 110 100 90 80 70 60 50 40 30 20 10 0
f1 (ppm)

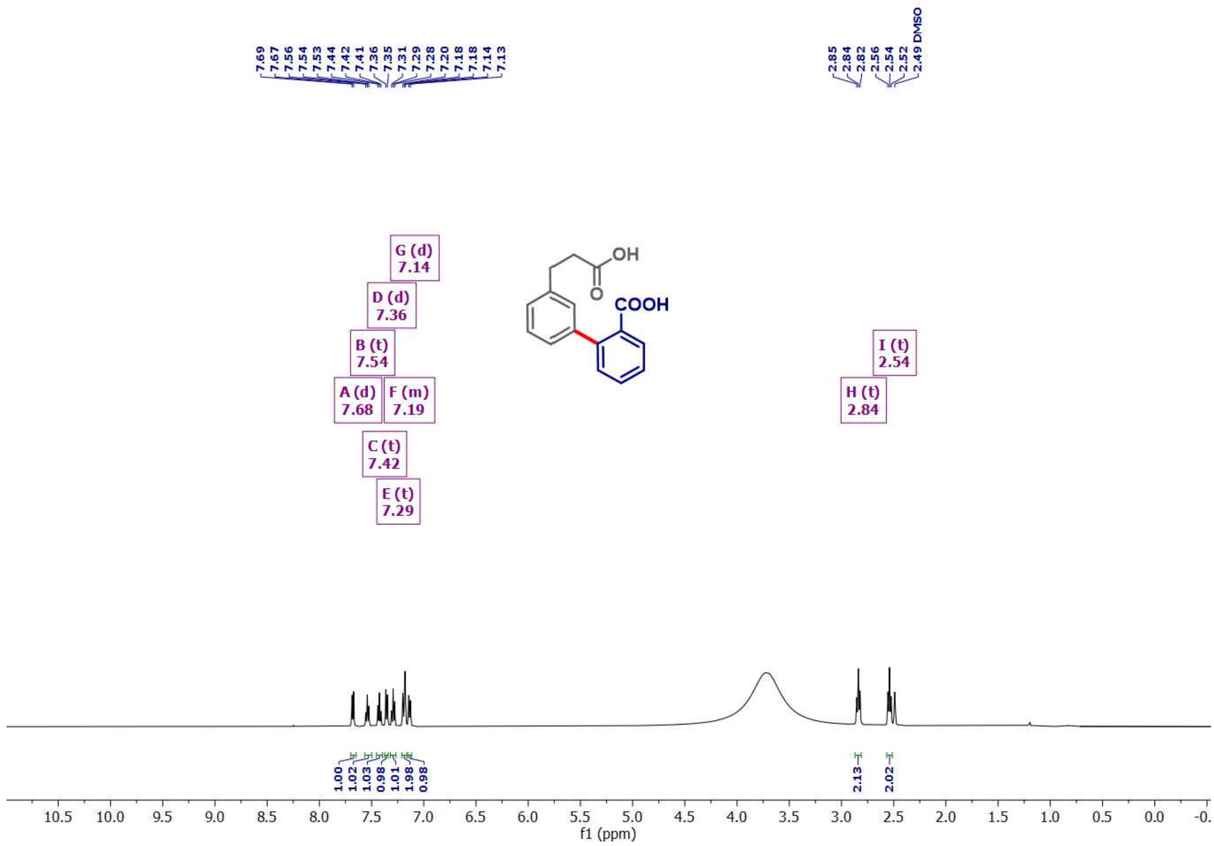

7.69
7.67
7.56
7.54
7.53
7.44
7.42
7.41
7.36
7.35
7.31
7.29
7.28
7.20
7.18
7.18
7.14
7.13
2.85
2.84
2.82
2.56
2.54
2.52
2.49 DMSO
OH
O
COOH
G (d) 7.14
D (d) 7.36
B (t) 7.54
A (d) 7.68
F (m) 7.19
C (t) 7.42
E (t) 7.29
I (t) 2.54
H (t) 2.84
1.00
1.02
1.03
0.98
1.01
1.98
0.98
2.13
2.02
f1 (ppm)

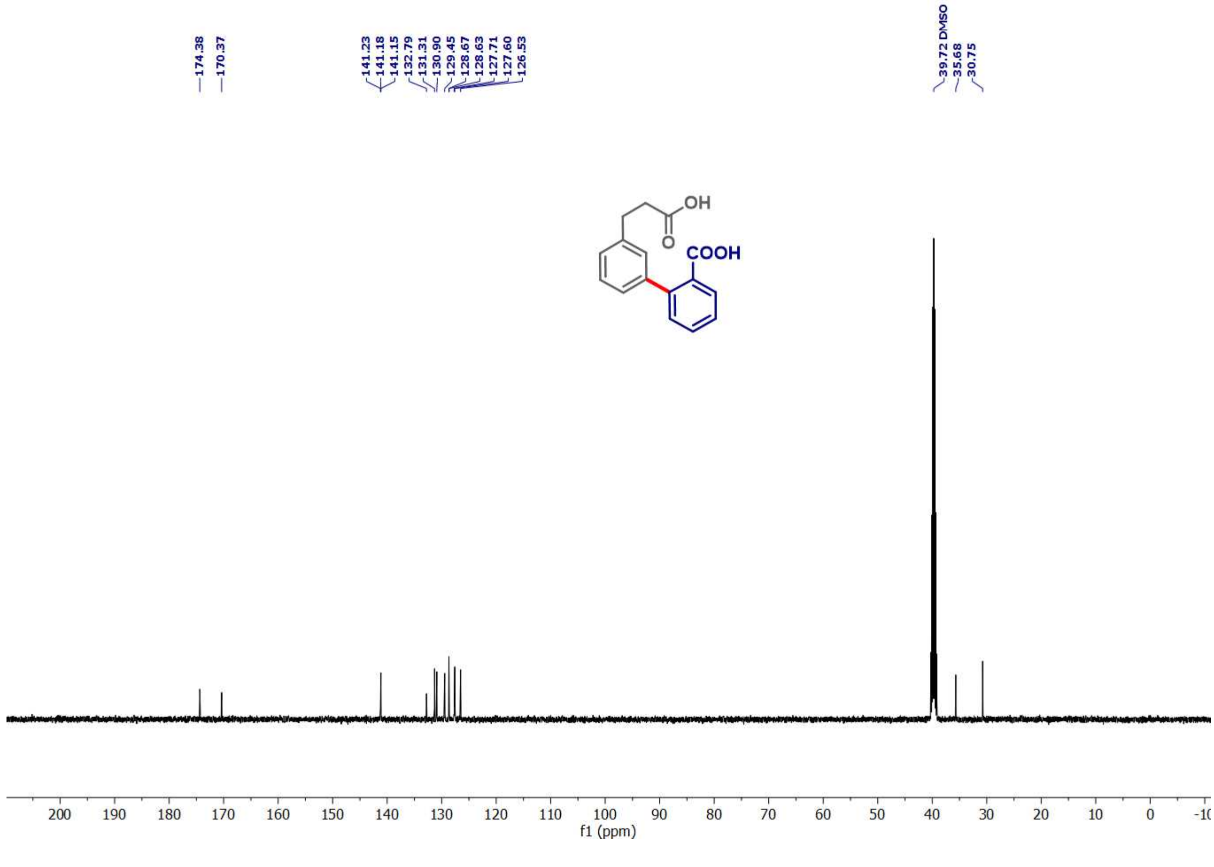

174.38
170.37
141.23
141.18
141.15
132.79
131.31
130.90
129.45
128.67
128.63
127.71
127.60
126.53
39.72 DMSO
35.68
30.75
OH
O
COOH
f1 (ppm)

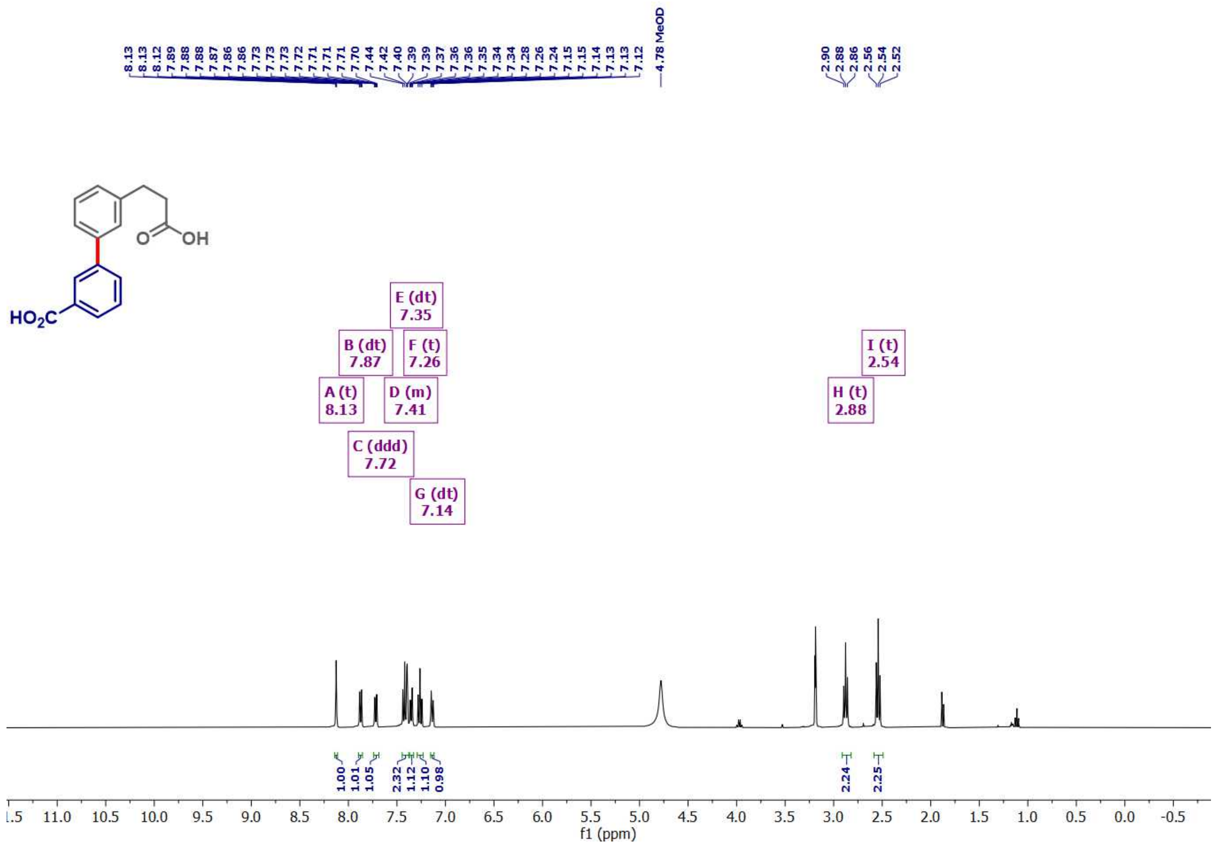

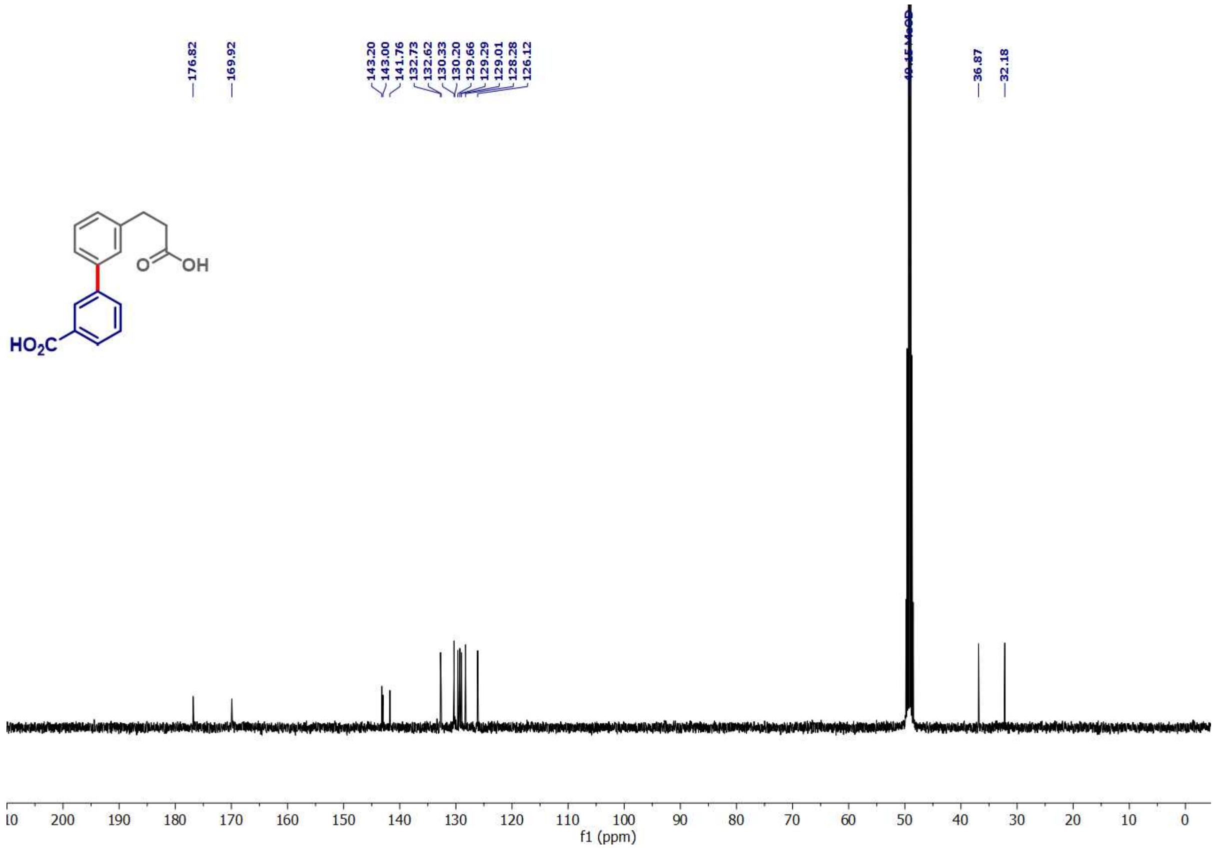

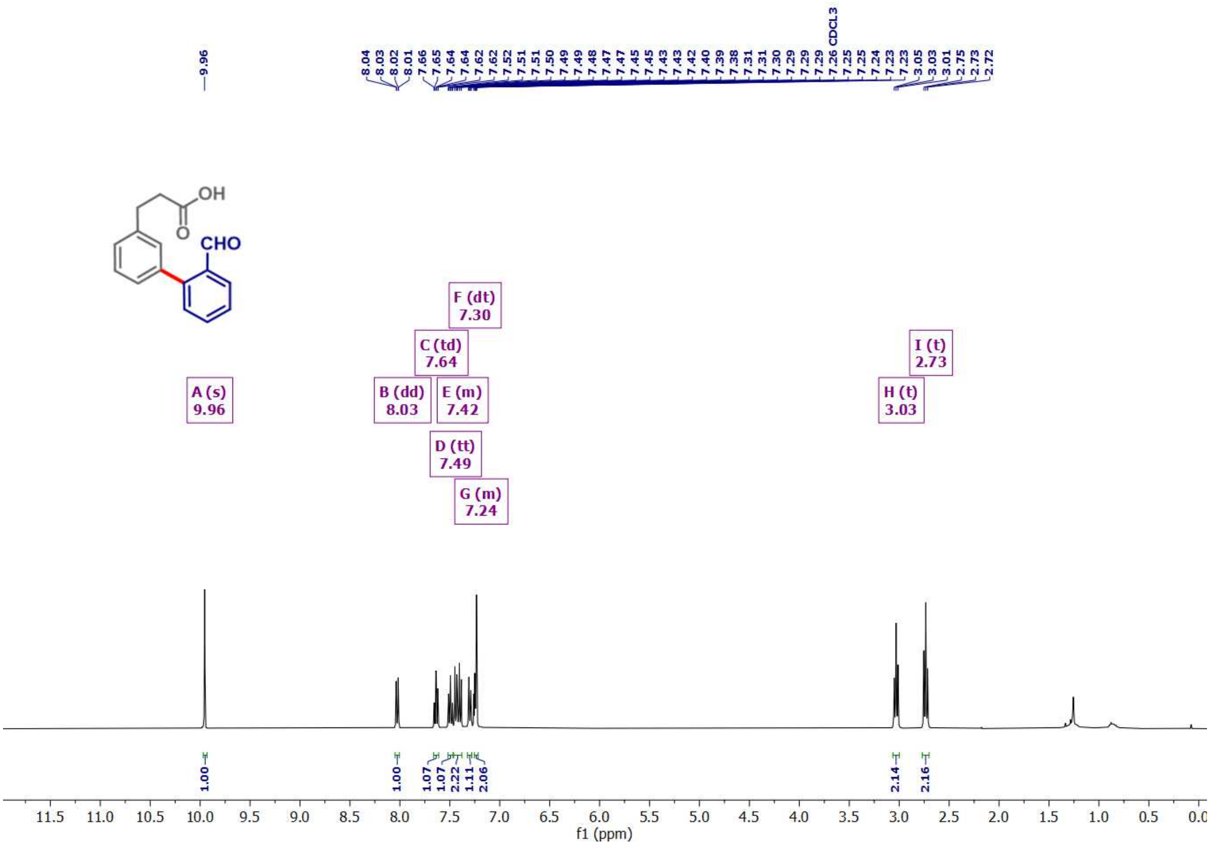
9.96
8.04
8.03
8.02
8.01
7.66
7.65
7.64
7.64
7.62
7.52
7.51
7.51
7.50
7.49
7.49
7.48
7.47
7.47
7.45
7.45
7.43
7.43
7.42
7.40
7.39
7.38
7.31
7.30
7.29
7.29
7.29
7.26 CDCL3
7.25
7.25
7.24
7.23
7.23
3.05
3.03
3.01
2.75
2.73
2.72
OH
O
CHO
F (dt)
7.30
C (td)
7.64
I (t)
2.73
A (s)
9.96
B (dd)
8.03
E (m)
7.42
H (t)
3.03
D (tt)
7.49
G (m)
7.24
1.00
1.00
1.07
1.07
2.22
1.11
2.06
2.14
2.16
f1 (ppm)

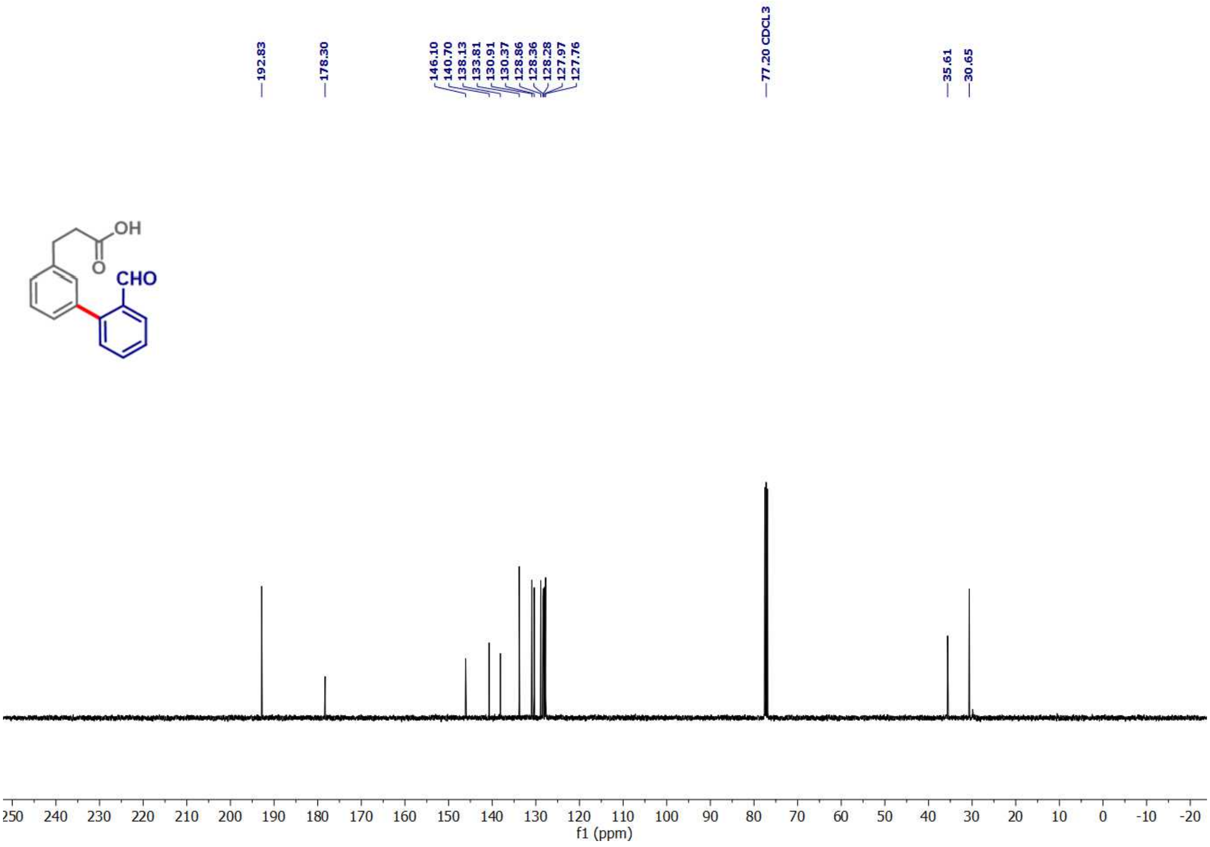
192.83
178.30
146.10
140.70
138.13
133.81
130.91
130.37
128.86
128.36
128.28
127.97
127.76
77.20 CDCL3
35.61
30.65
OH
O
CHO
f1 (ppm)

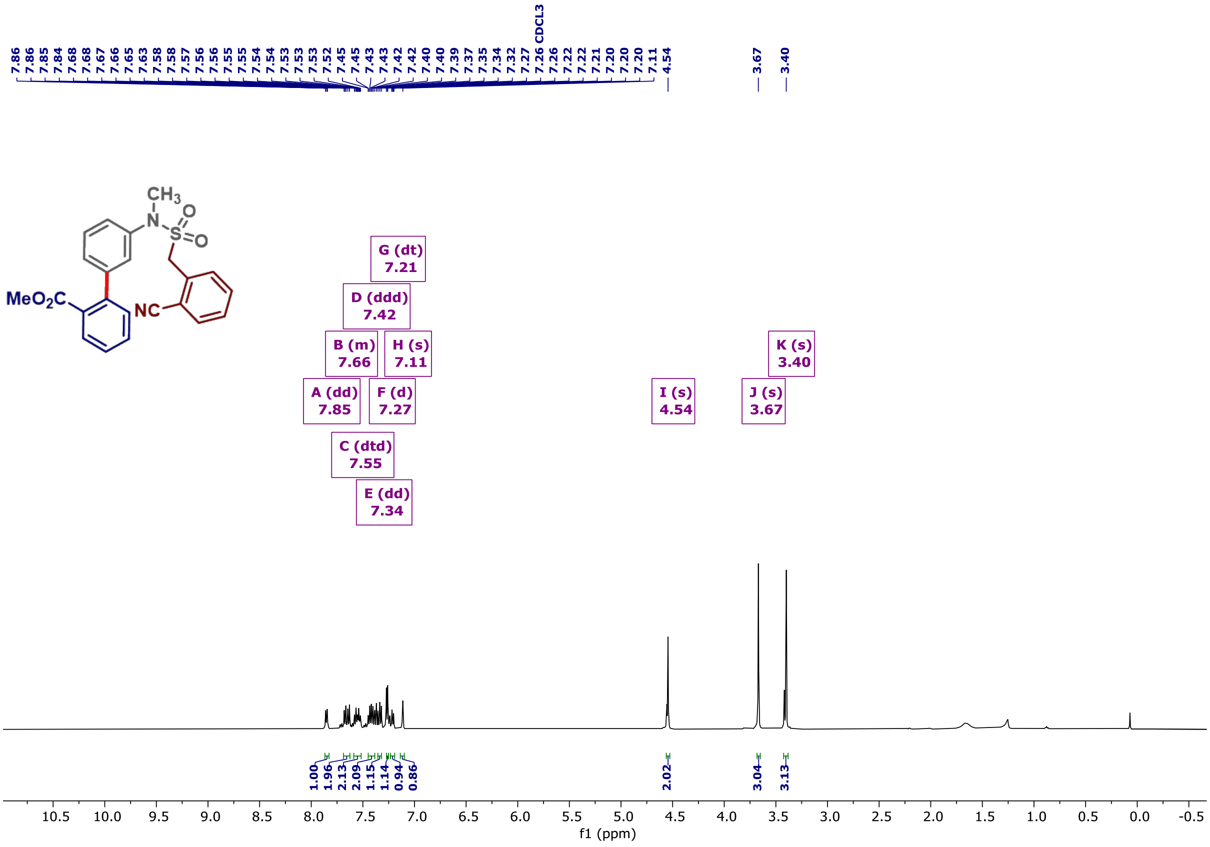
7.86 7.86 7.85 7.84 7.68 7.68 7.67 7.66 7.65 7.63 7.58 7.58 7.57 7.56 7.56 7.55 7.55 7.54 7.54 7.53 7.53 7.52 7.45 7.43 7.43 7.42 7.40 7.40 7.39 7.37 7.35 7.34 7.32 7.27 7.26 CDCL3 7.26 7.22 7.22 7.21 7.20 7.20 7.20 7.11 4.54 3.67 3.40
CH3
N
S
O
O
MeO2C
NC
G (dt) 7.21
D (ddd) 7.42
B (m) 7.66
H (s) 7.11
K (s) 3.40
A (dd) 7.85
F (d) 7.27
I (s) 4.54
J (s) 3.67
C (dtd) 7.55
E (dd) 7.34
1.00 1.96 2.13 2.09 1.15 1.14 0.94 0.86
2.02
3.04
3.13
f1 (ppm)

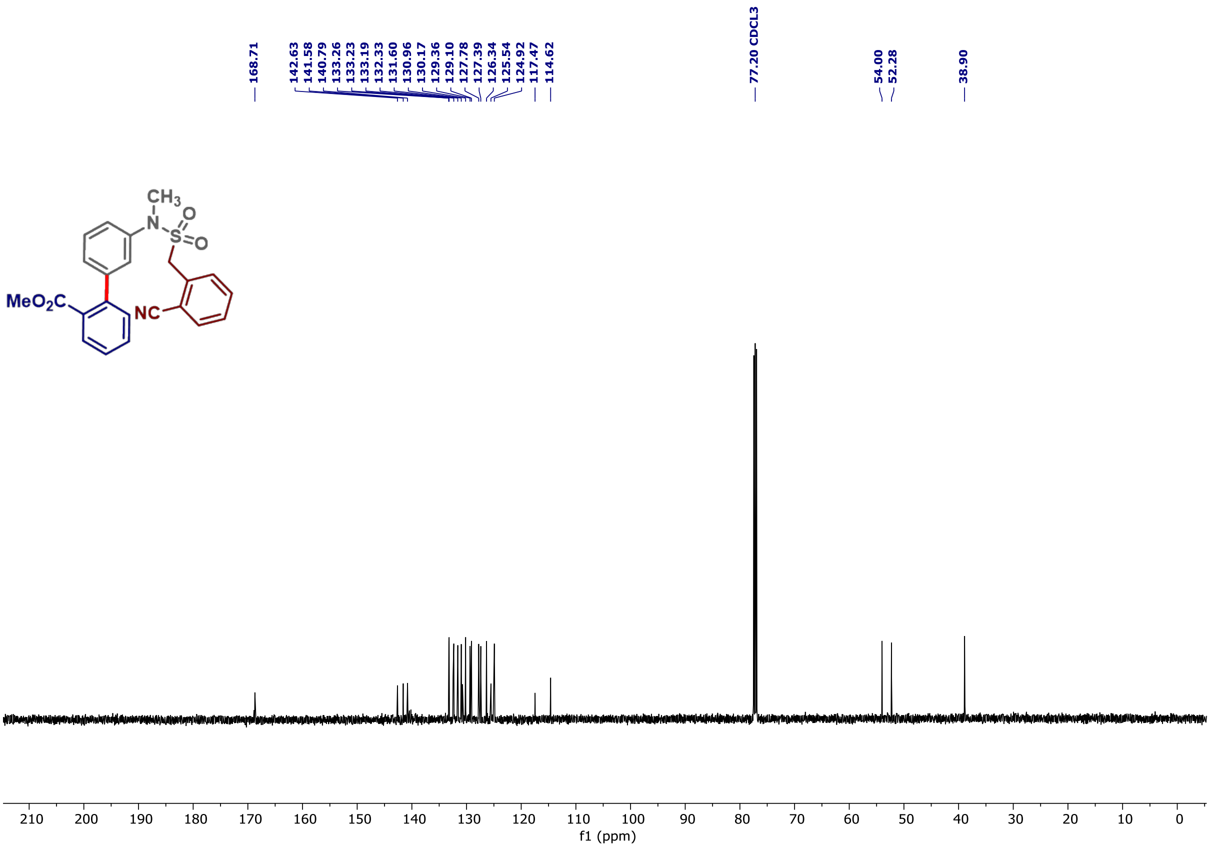
168.71
142.63 141.58 140.79 133.26 133.23 133.19 132.33 131.60 130.96 130.17 129.36 129.10 127.78 127.39 126.34 125.54 124.92 117.47 114.62
77.20 CDCL3
54.00 52.28
38.90
CH3
N
S
O
O
MeO2C
NC
f1 (ppm)

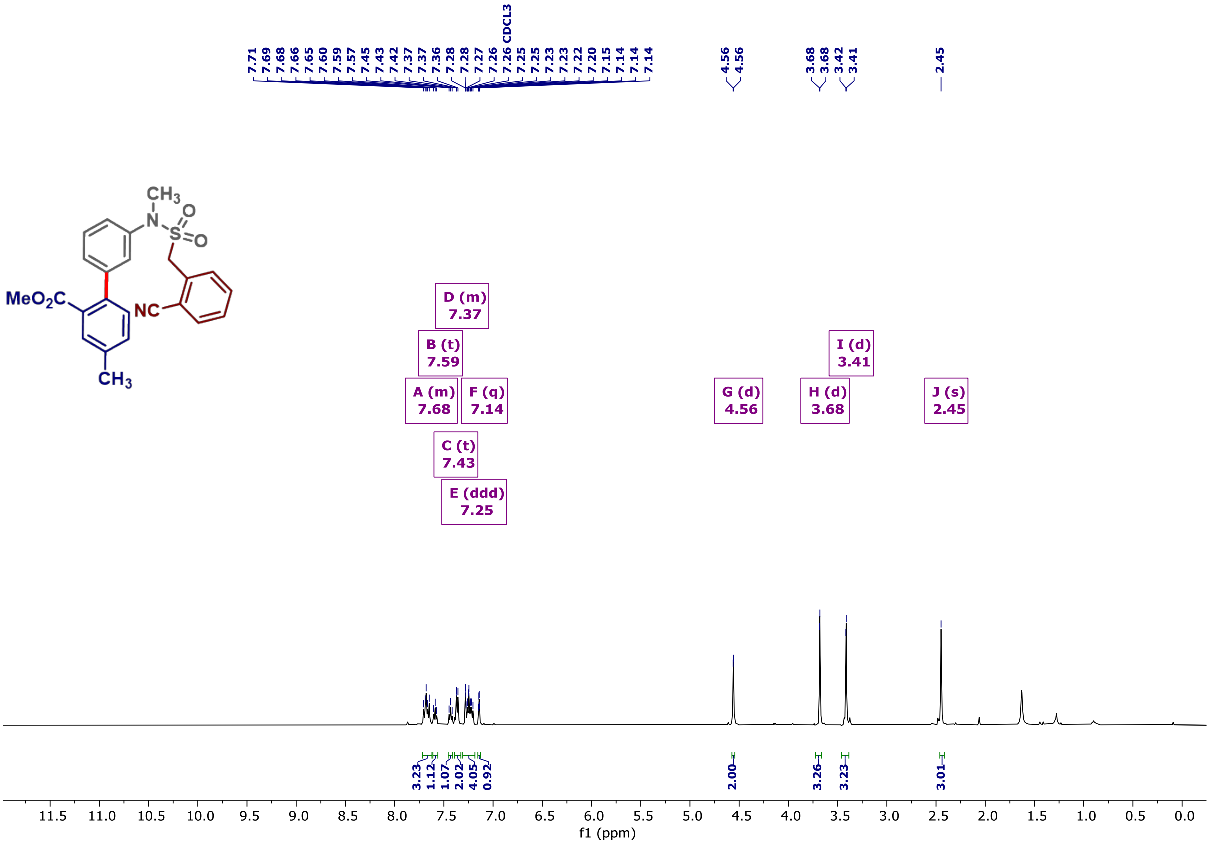
7.71 7.69 7.68 7.66 7.65 7.60 7.59 7.57 7.45 7.43 7.42 7.37 7.37 7.36 7.28 7.28 7.27 7.26 CDCL3 7.25 7.25 7.23 7.23 7.22 7.20 7.15 7.14 7.14 7.14
4.56 4.56
3.68 3.68 3.42 3.41
2.45
CH3
N
S
O
O
MeO2C
NC
CH3
D (m) 7.37
B (t) 7.59
A (m) 7.68
F (q) 7.14
C (t) 7.43
E (ddd) 7.25
G (d) 4.56
I (d) 3.41
H (d) 3.68
J (s) 2.45
3.23 1.12 1.07 2.02 4.05 0.92
2.00
3.26 3.23
3.01
f1 (ppm)

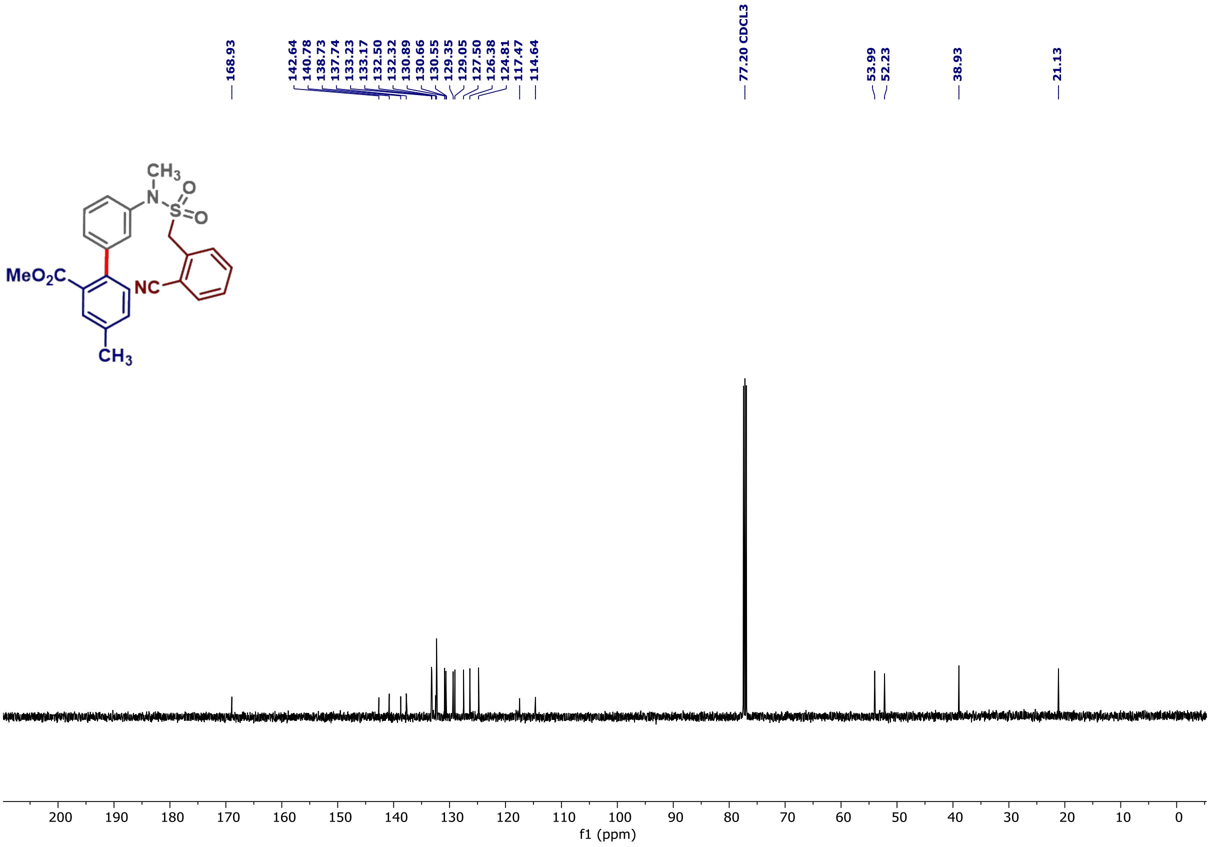
168.93
142.64 140.78 138.73 137.74 133.23 133.17 132.50 132.32 130.89 130.66 130.55 129.35 129.05 127.50 126.38 124.81 117.47 114.64
77.20 CDCL3
53.99 52.23
38.93
21.13
CH3
N
S
O
O
MeO2C
NC
CH3
f1 (ppm)

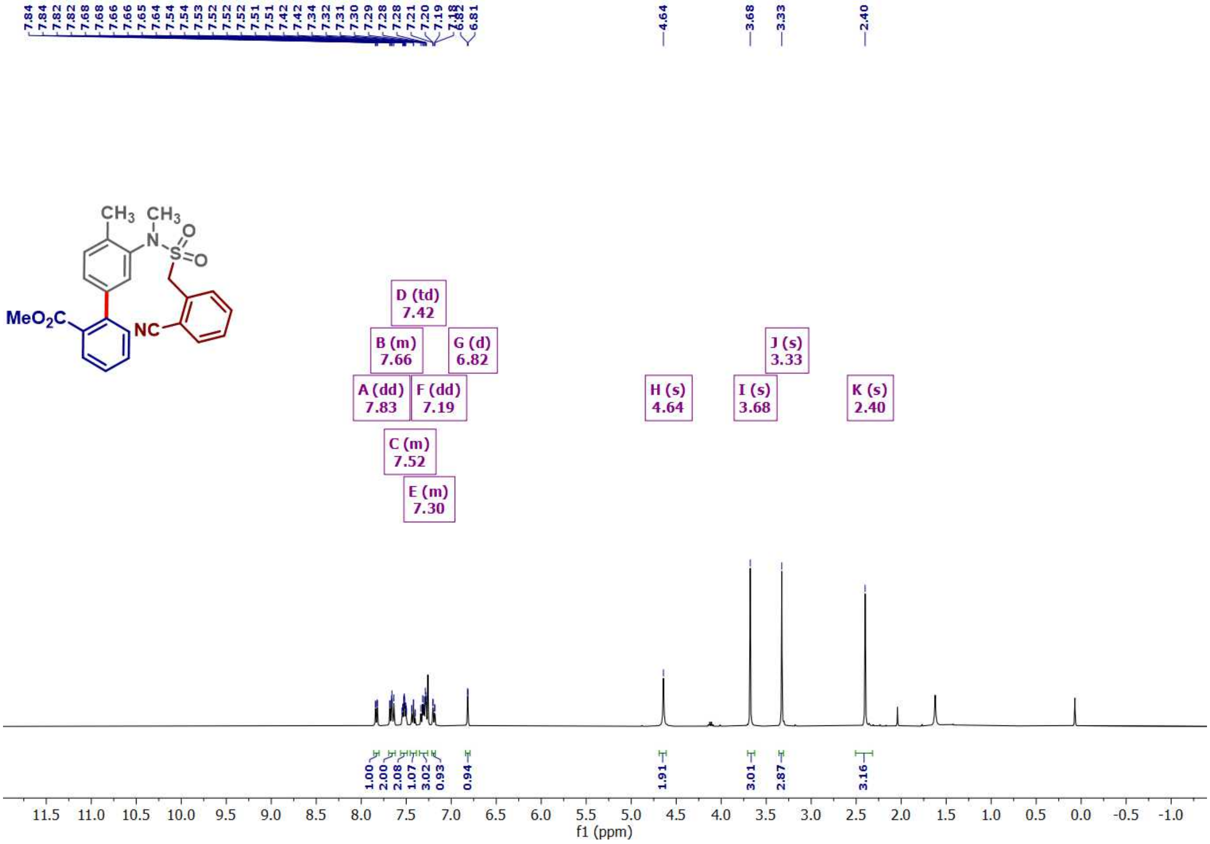

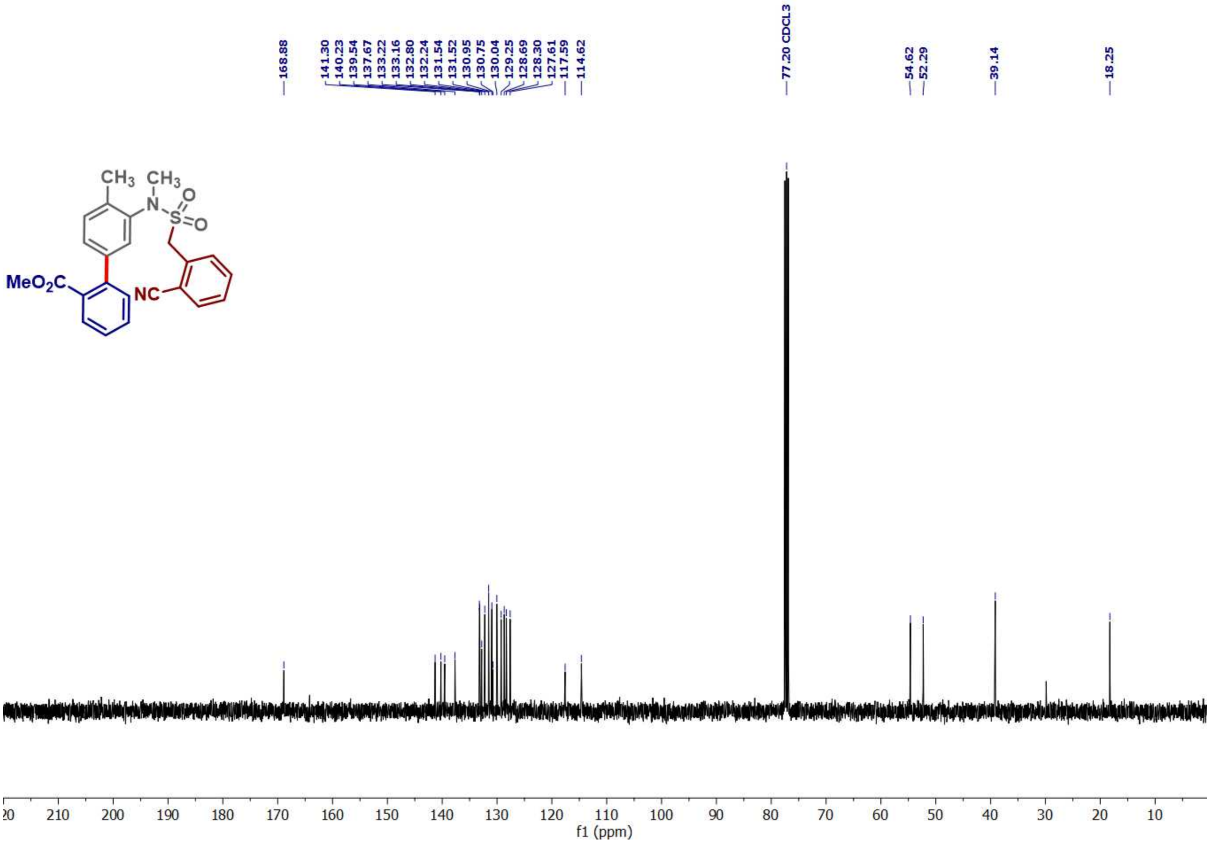

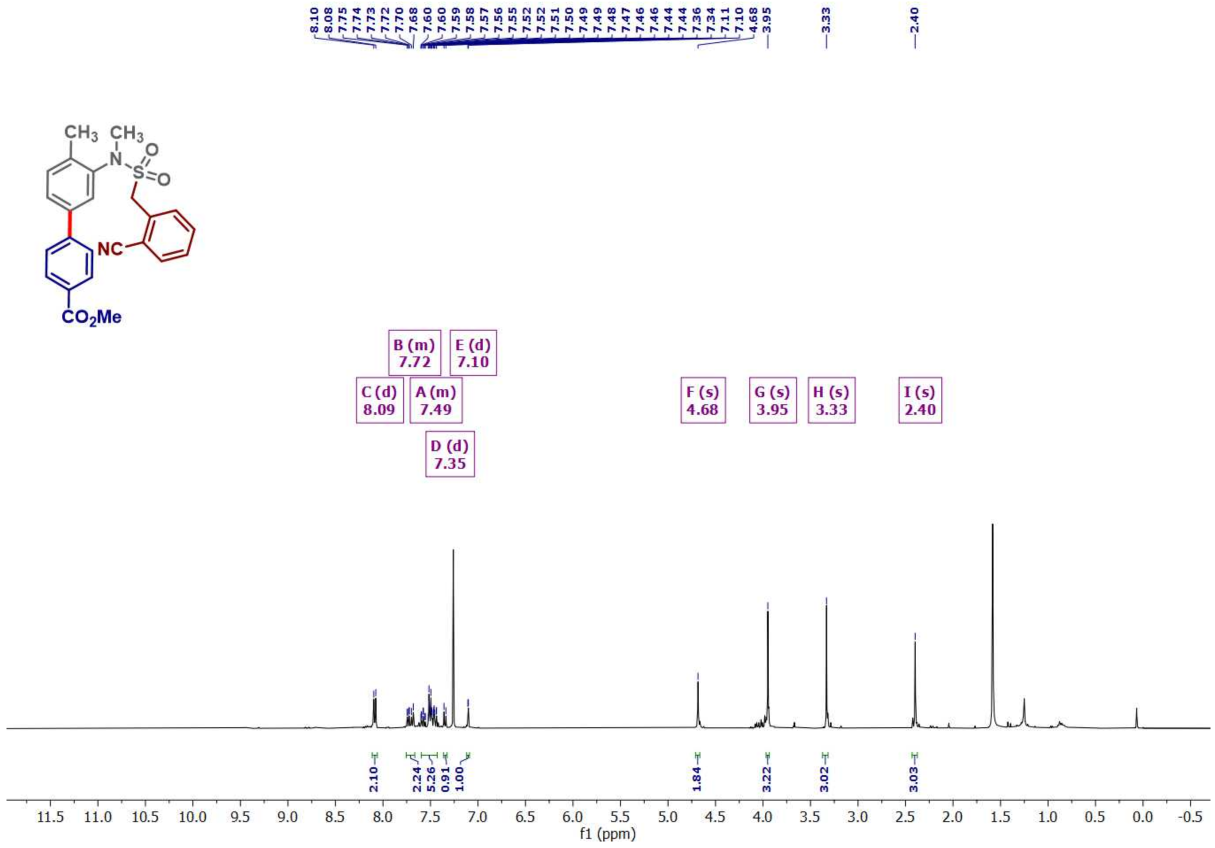
8.10 8.08 7.75 7.74 7.73 7.72 7.70 7.68 7.60 7.60 7.59 7.58 7.57 7.56 7.55 7.52 7.52 7.51 7.50 7.49 7.49 7.48 7.47 7.46 7.46 7.44 7.44 7.36 7.34 7.11 7.10 4.68 3.95
3.33
2.40
CH3 CH3
N
S
O
O
NC
CO2Me
B (m) 7.72
E (d) 7.10
C (d) 8.09
A (m) 7.49
D (d) 7.35
F (s) 4.68
G (s) 3.95
H (s) 3.33
I (s) 2.40
2.10
2.24
5.26
0.91
1.00
1.84
3.22
3.02
3.03
f1 (ppm)

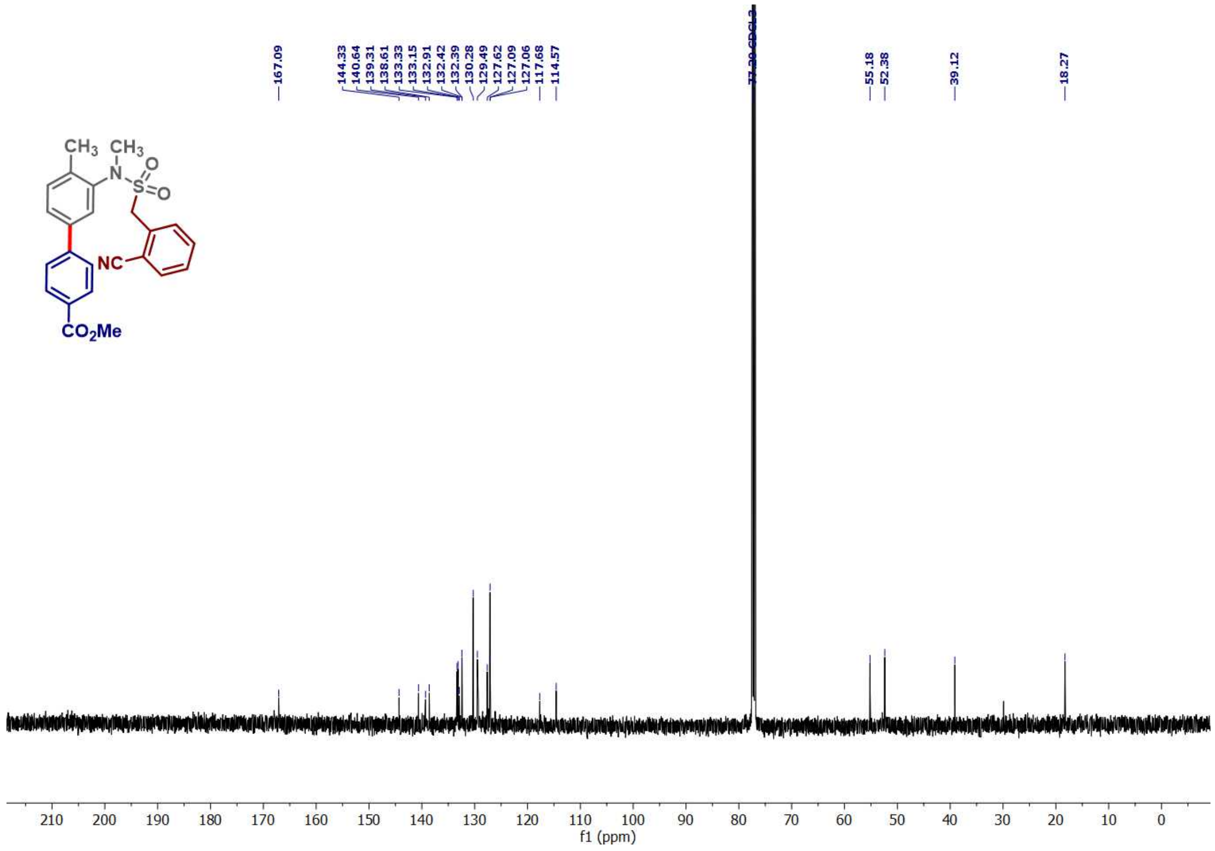
167.09
144.33 140.64 139.31 138.61 133.33 133.15 132.91 132.42 132.39 130.28 129.49 127.62 127.09 127.06 117.68 114.57
55.18
52.38
39.12
18.27
CH3 CH3
N
S
O
O
NC
CO2Me
f1 (ppm)

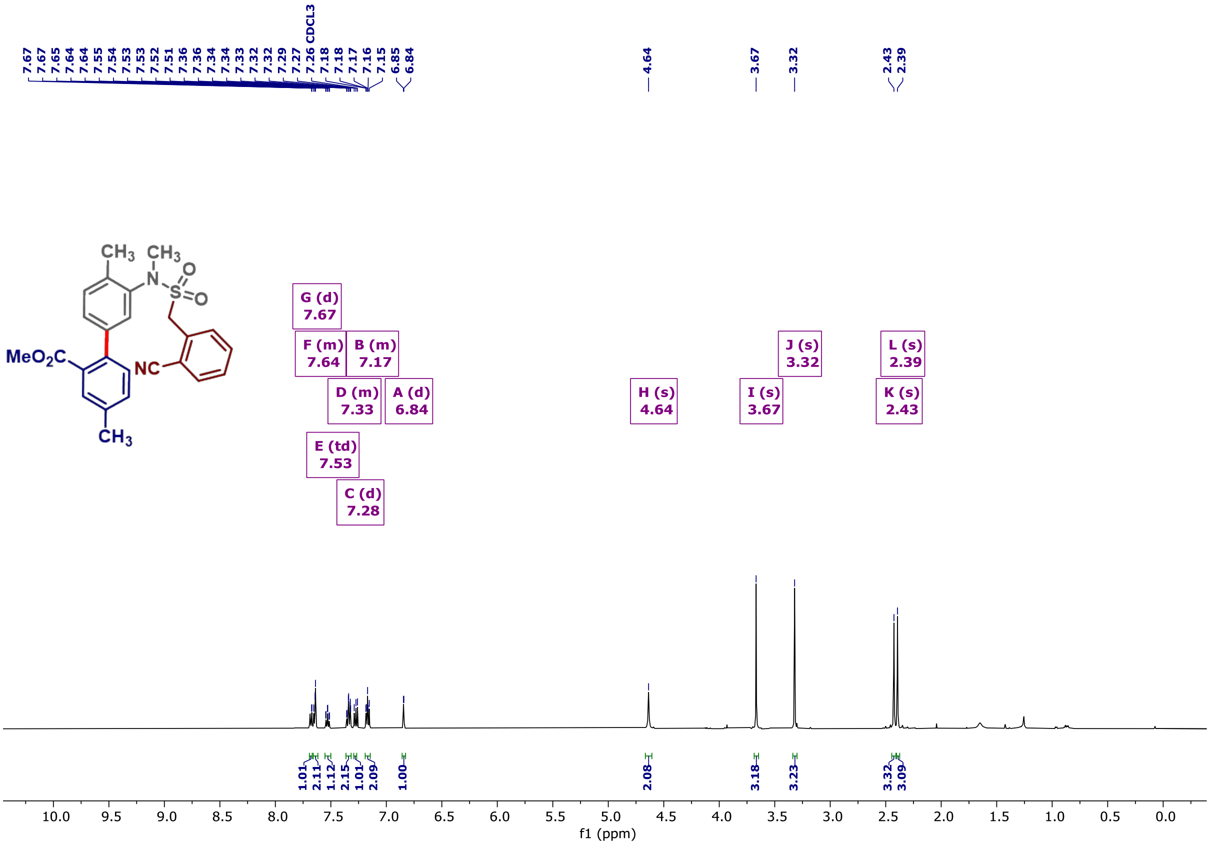

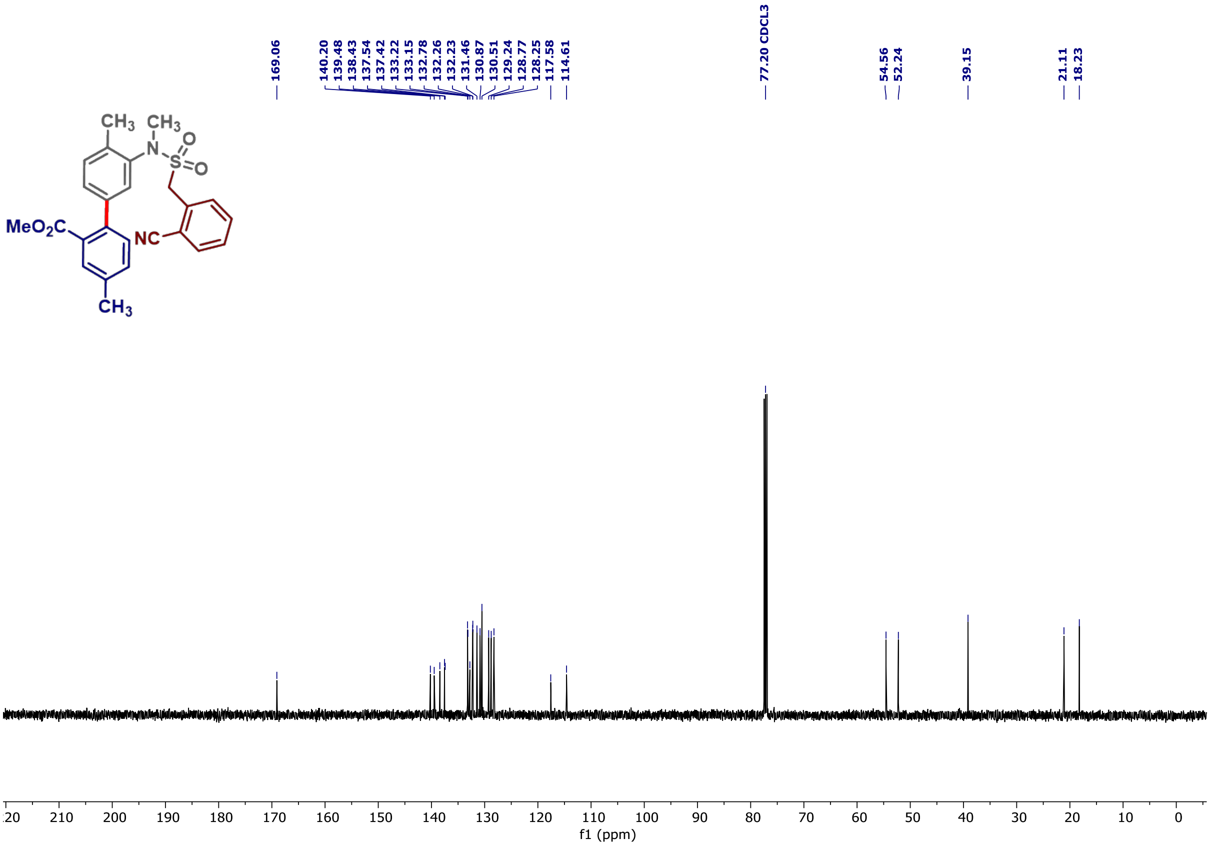

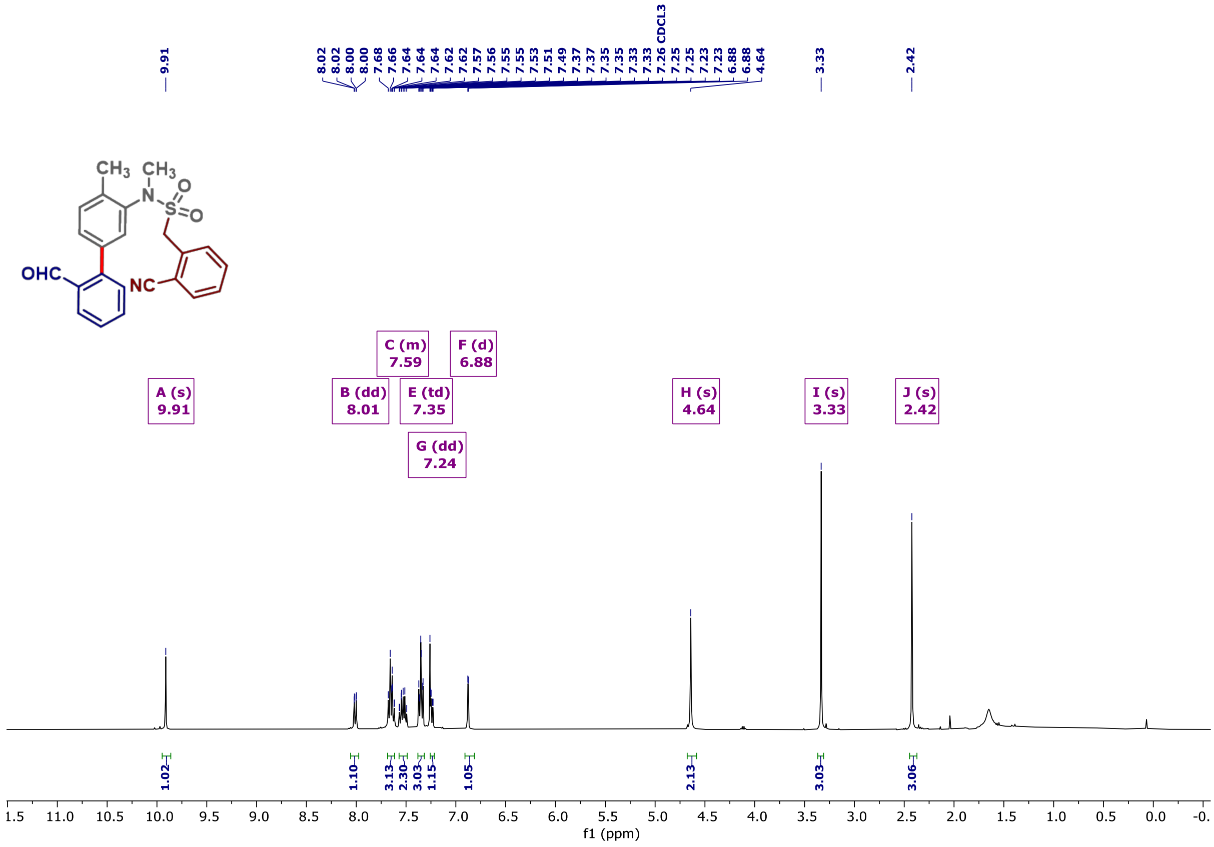
9.91
8.02
8.02
8.00
8.00
7.68
7.66
7.64
7.64
7.64
7.62
7.62
7.57
7.56
7.55
7.55
7.53
7.51
7.49
7.37
7.37
7.35
7.35
7.33
7.33
7.26 CDCL3
7.25
7.25
7.23
7.23
6.88
6.88
4.64
3.33
2.42
CH_3
CH_3
N
S
O
O
OHC
NC
C (m) 7.59
F (d) 6.88
A (s) 9.91
B (dd) 8.01
E (td) 7.35
H (s) 4.64
I (s) 3.33
J (s) 2.42
G (dd) 7.24
1.02
1.10
3.13
2.30
3.03
1.15
1.05
2.13
3.03
3.06
f1 (ppm)

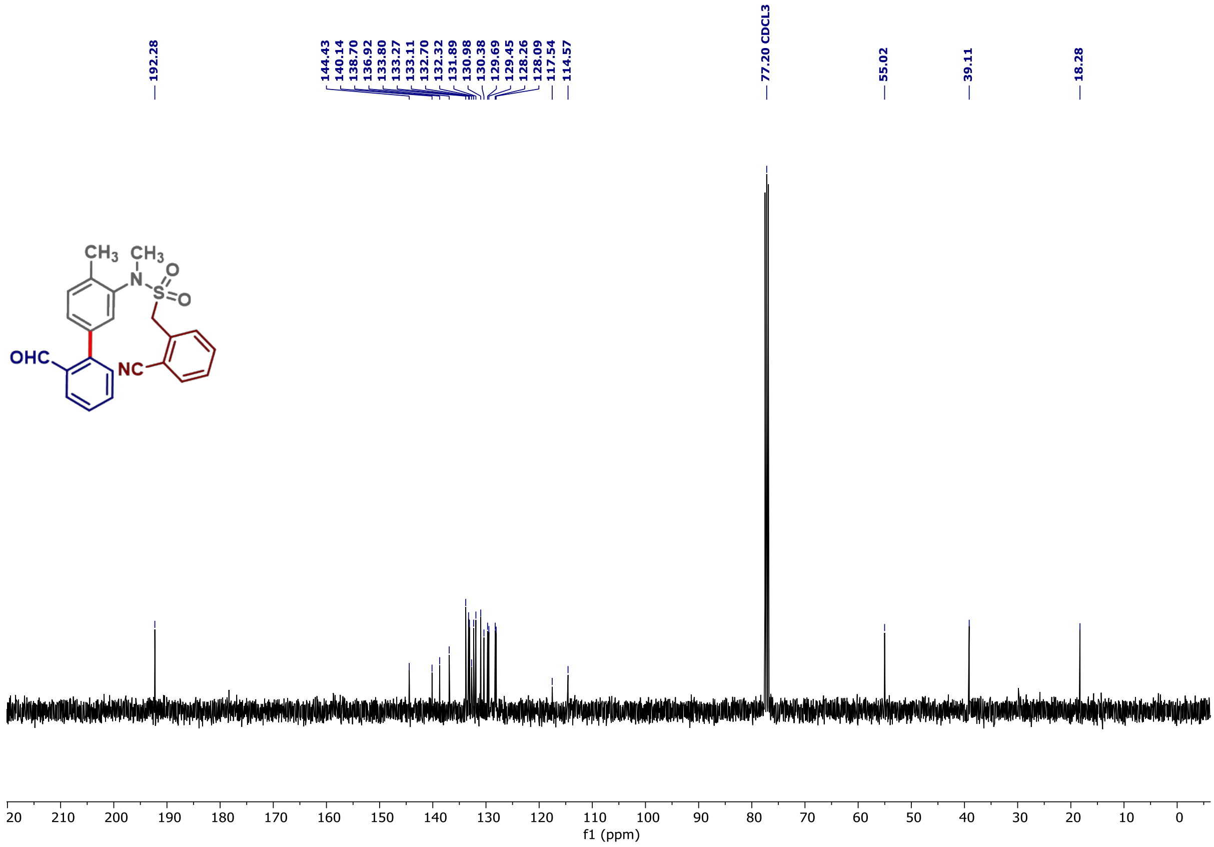
192.28
144.43
140.14
138.70
136.92
133.80
133.27
133.11
132.70
132.32
131.89
130.98
130.38
129.69
129.45
128.26
128.09
117.54
114.57
77.20 CDCL3
55.02
39.11
18.28
CH_3
CH_3
N
S
O
O
OHC
NC
f1 (ppm)

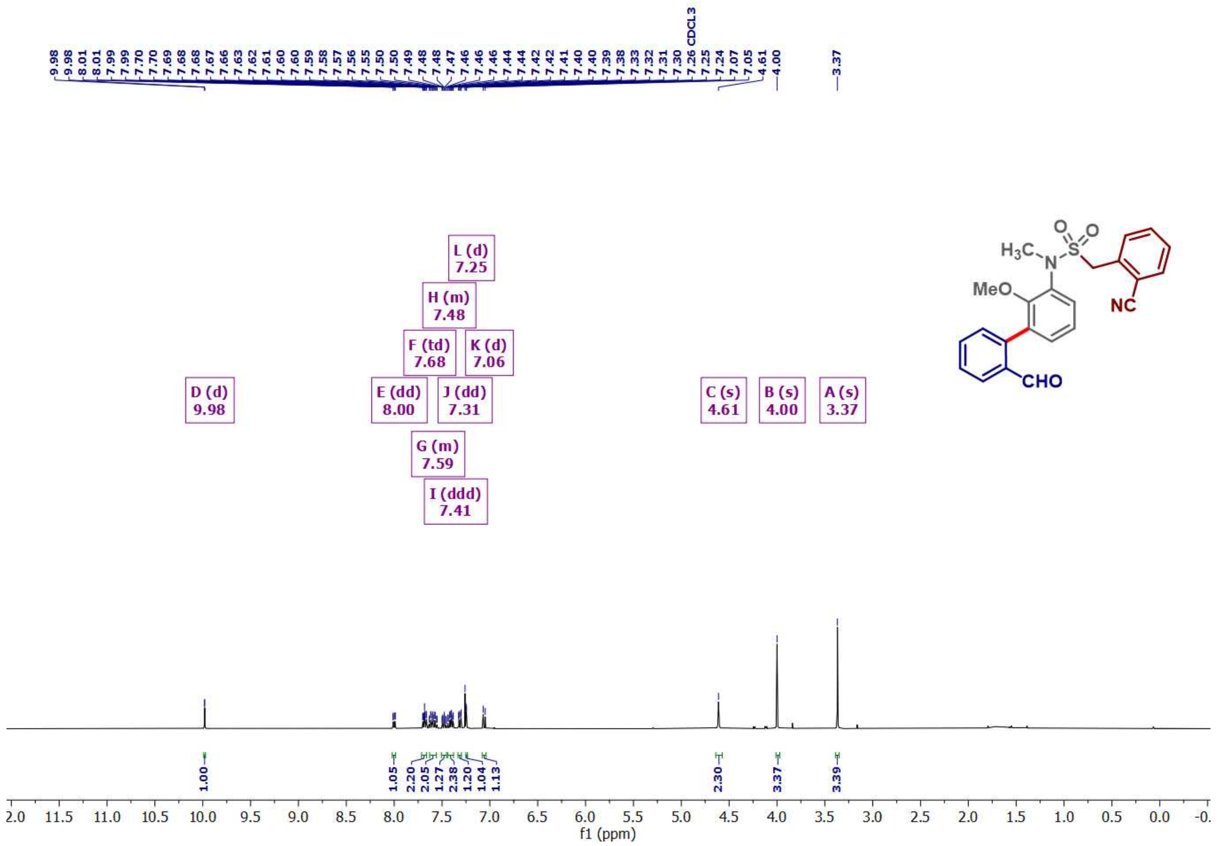
9.98 9.98 8.01 7.99 7.70 7.69 7.68 7.68 7.67 7.66 7.63 7.62 7.61 7.60 7.60 7.59 7.58 7.57 7.56 7.55 7.50 7.50 7.49 7.48 7.48 7.47 7.46 7.46 7.44 7.44 7.42 7.41 7.40 7.40 7.39 7.38 7.33 7.32 7.31 7.30 7.26 CDCL3 7.25 7.24 7.07 7.05 4.61 4.00 3.37
L (d) 7.25
H (m) 7.48
F (td) 7.68
K (d) 7.06
D (d) 9.98
E (dd) 8.00
J (dd) 7.31
C (s) 4.61
B (s) 4.00
A (s) 3.37
G (m) 7.59
I (ddd) 7.41
H_3C
N
S
O
O
MeO
NC
CHO
1.00 1.05 2.20 2.05 1.27 2.38 1.20 1.04 1.13 2.30 3.37 3.39
f1 (ppm)

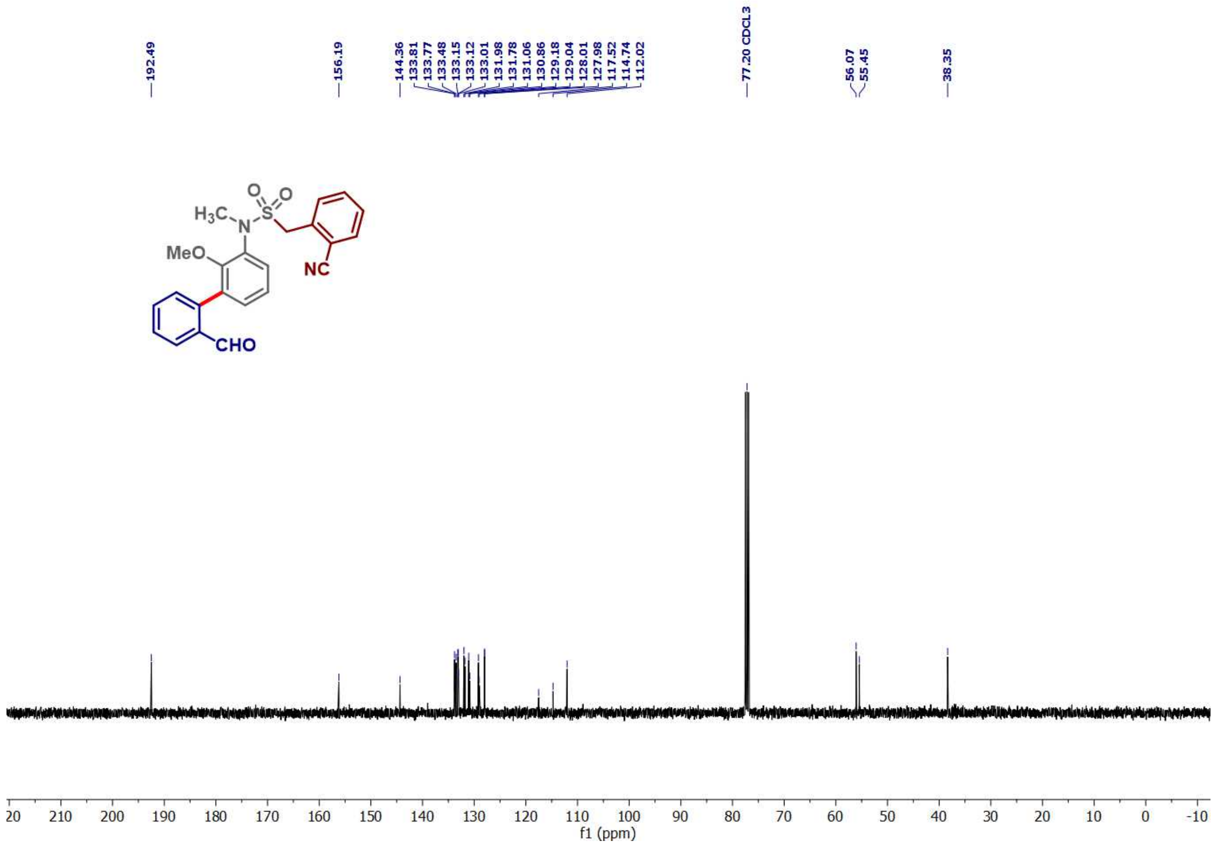
192.49 156.19 144.36 133.81 133.77 133.48 133.15 133.12 133.01 131.98 131.78 131.06 130.86 129.18 129.04 128.01 127.98 117.52 114.74 112.02 77.20 CDCL3 56.07 55.45 38.35
H_3C
N
S
O
O
MeO
NC
CHO
f1 (ppm)

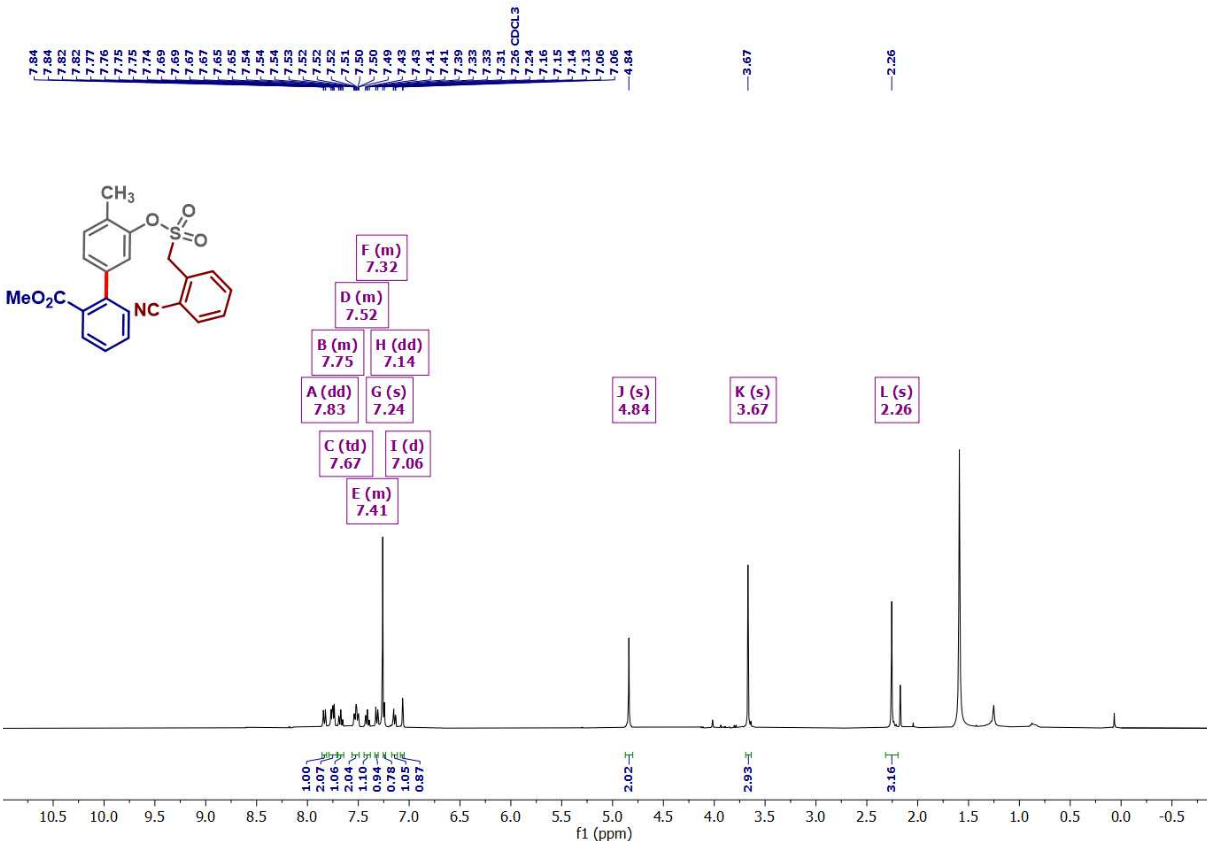
7.84 7.84 7.82 7.82 7.77 7.76 7.75 7.75 7.74 7.69 7.69 7.67 7.67 7.65 7.65 7.54 7.54 7.53 7.52 7.52 7.51 7.50 7.50 7.49 7.43 7.43 7.41 7.41 7.39 7.33 7.33 7.31 7.26 CDCL3 7.24 7.16 7.15 7.14 7.13 7.06 4.84
3.67
2.26
CH3
O
S
O
O
MeO2C
NC
F (m) 7.32
D (m) 7.52
B (m) 7.75
H (dd) 7.14
A (dd) 7.83
G (s) 7.24
C (td) 7.67
I (d) 7.06
E (m) 7.41
J (s) 4.84
K (s) 3.67
L (s) 2.26
1.00 2.07 1.06 2.04 1.10 0.94 0.78 1.05 0.87
2.02
2.93
3.16
10.5 10.0 9.5 9.0 8.5 8.0 7.5 7.0 6.5 6.0 5.5 5.0 4.5 4.0 3.5 3.0 2.5 2.0 1.5 1.0 0.5 0.0 -0.5
f1 (ppm)

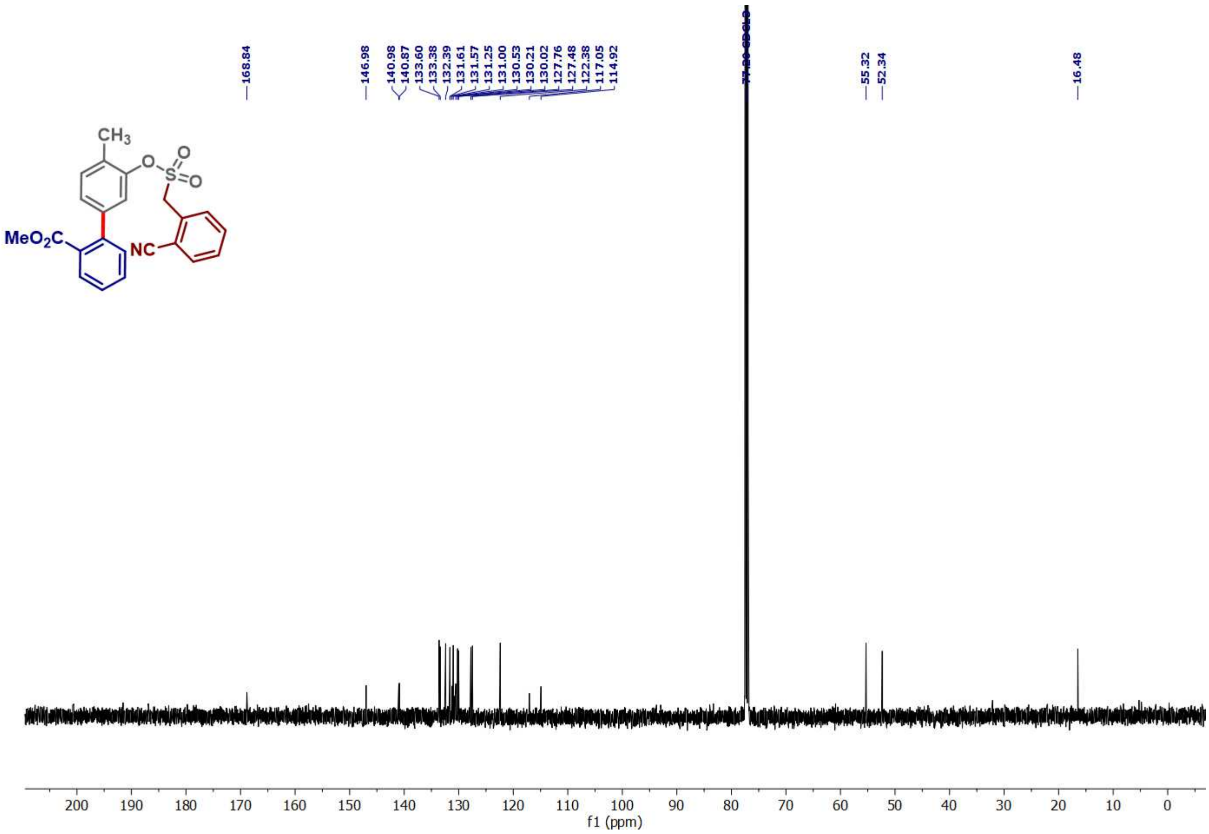
168.84
146.98 140.98 140.87 133.60 133.38 132.39 131.61 131.57 131.25 131.00 130.53 130.21 130.02 127.76 127.48 122.38 117.05 114.92
CDCl3
55.32
52.34
16.48
CH3
O
S
O
O
MeO2C
NC
200 190 180 170 160 150 140 130 120 110 100 90 80 70 60 50 40 30 20 10 0
f1 (ppm)

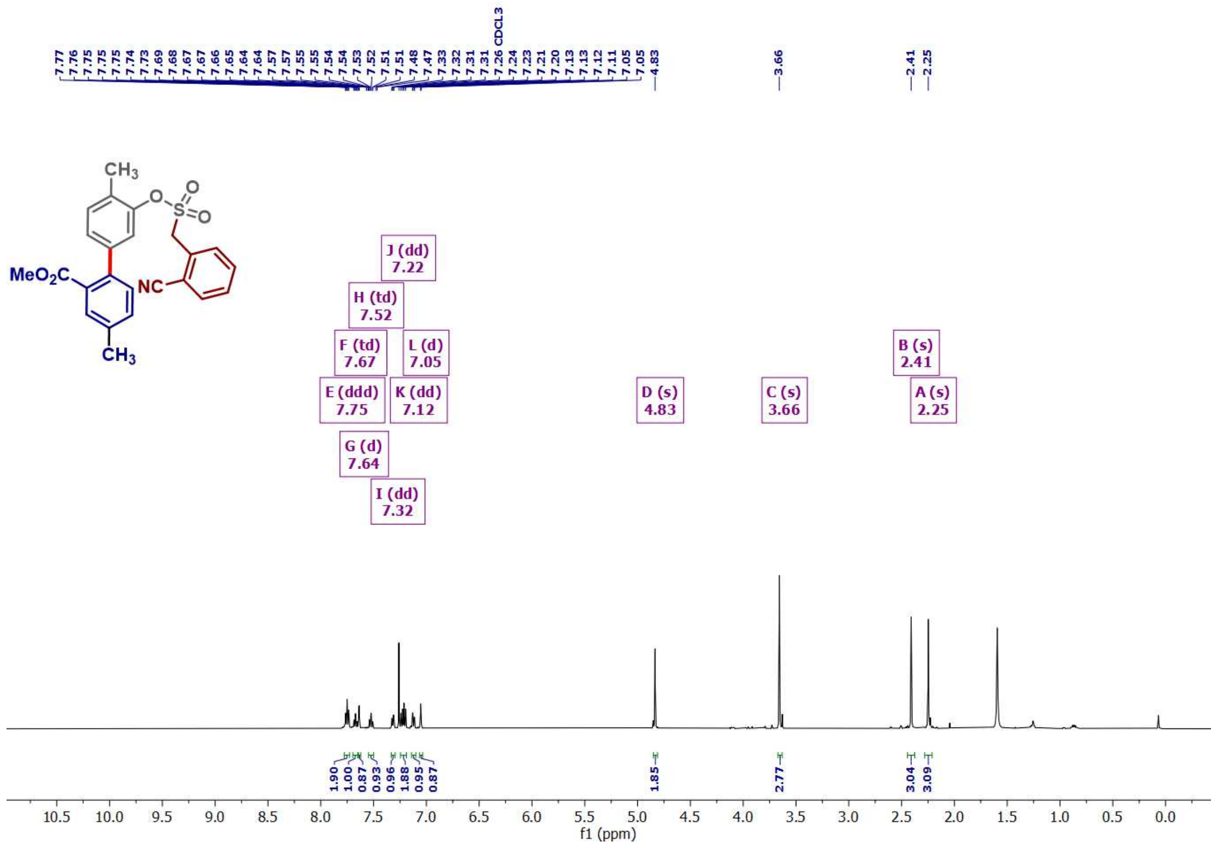
CDCL3
4.83
3.66
2.41
2.25
CH3
MeO2C
NC
J (dd) 7.22
H (td) 7.52
F (td) 7.67
L (d) 7.05
E (ddd) 7.75
K (dd) 7.12
G (d) 7.64
I (dd) 7.32
D (s) 4.83
C (s) 3.66
B (s) 2.41
A (s) 2.25
1.90
1.00
0.87
0.93
0.96
1.88
0.95
0.87
1.85
2.77
3.04
3.09
f1 (ppm)

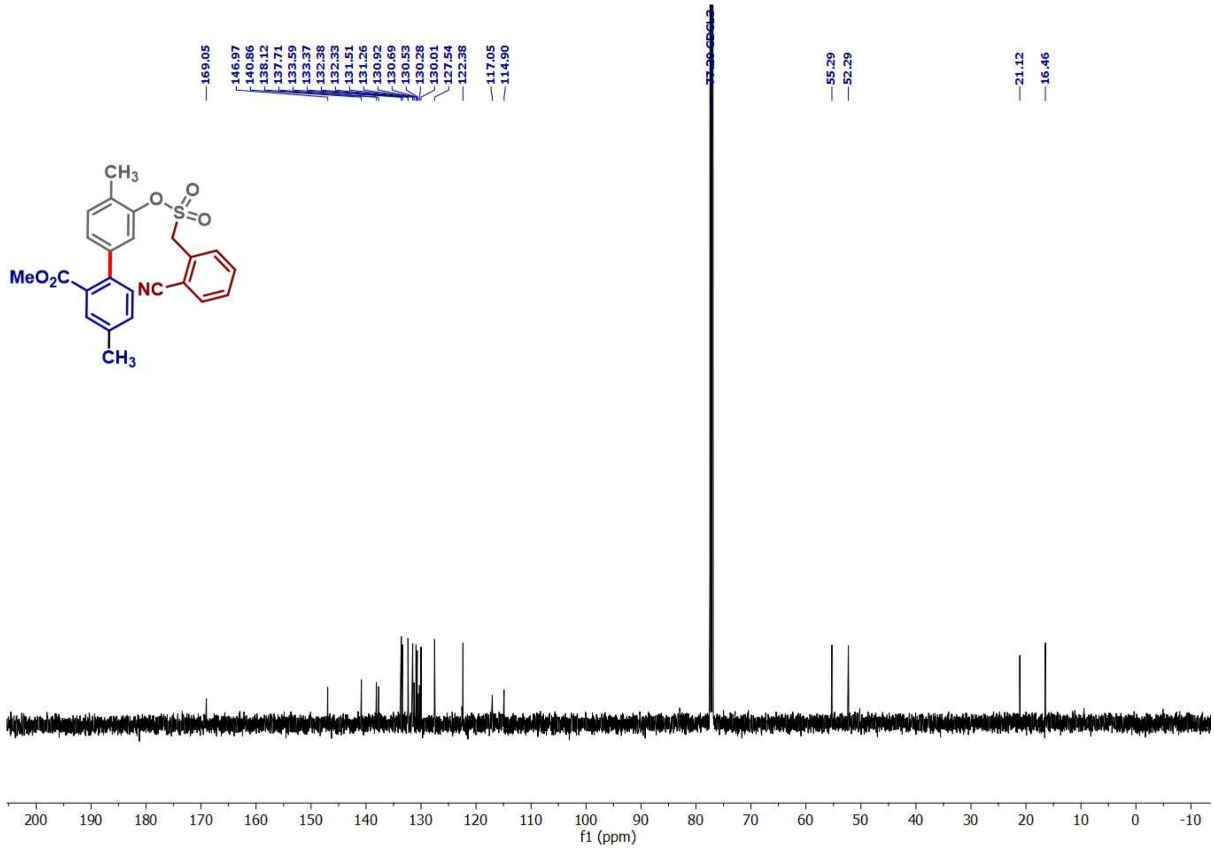
169.05
146.97
140.86
138.12
137.71
133.59
133.37
132.38
132.33
131.51
131.26
130.92
130.69
130.53
130.28
130.01
127.54
122.38
117.05
114.90
55.29
52.29
21.12
16.46
CH3
MeO2C
NC
f1 (ppm)

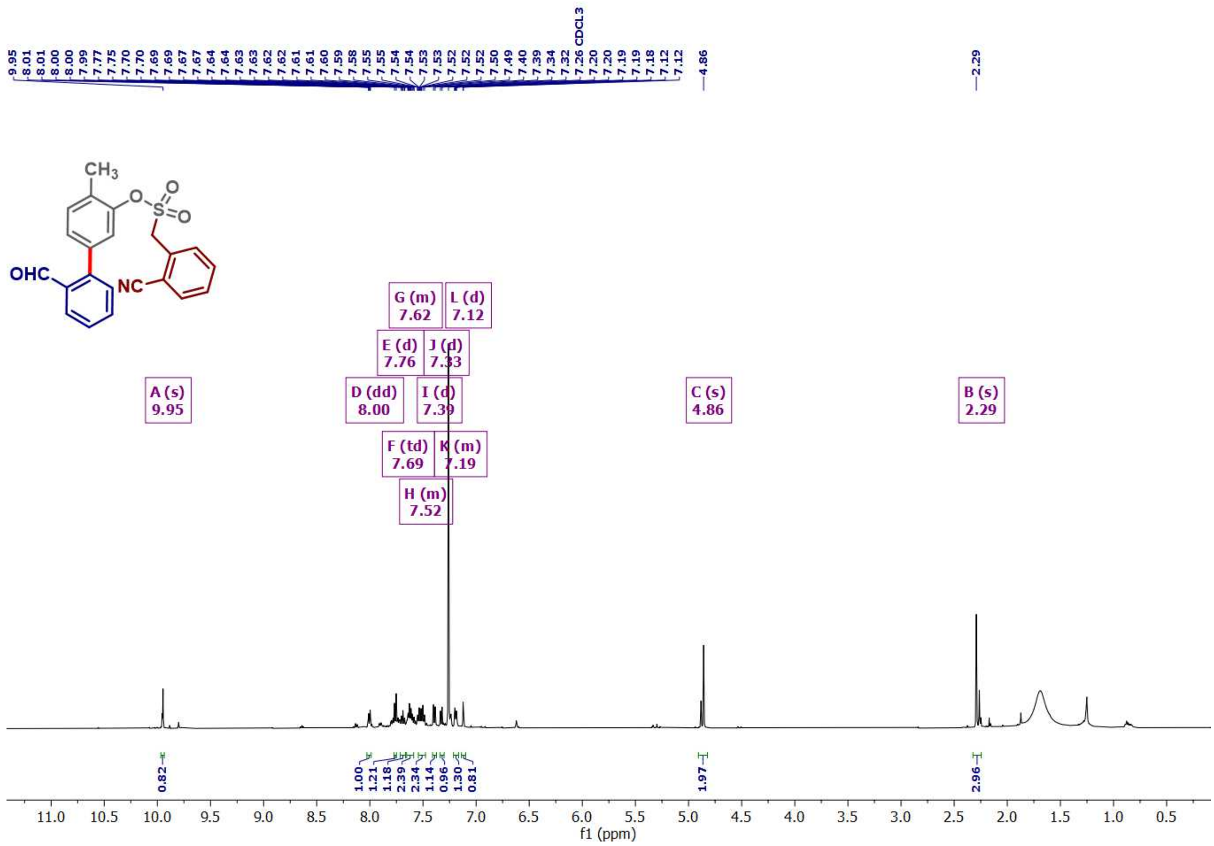
9.95
8.01
8.00
7.99
7.77
7.75
7.70
7.69
7.67
7.64
7.63
7.62
7.61
7.60
7.59
7.58
7.55
7.54
7.53
7.52
7.50
7.49
7.40
7.39
7.34
7.32
7.26 CDCL3
7.20
7.19
7.18
7.12
4.86
2.29
CH3
O
S
O
O
OHC
NC
A (s)
9.95
D (dd)
8.00
E (d)
7.76
F (td)
7.69
G (m)
7.62
H (m)
7.52
I (d)
7.39
J (d)
7.33
K (m)
7.19
L (d)
7.12
C (s)
4.86
B (s)
2.29
0.82
1.00
1.21
1.18
2.39
2.34
1.14
0.96
1.30
0.81
1.97
2.96
f1 (ppm)

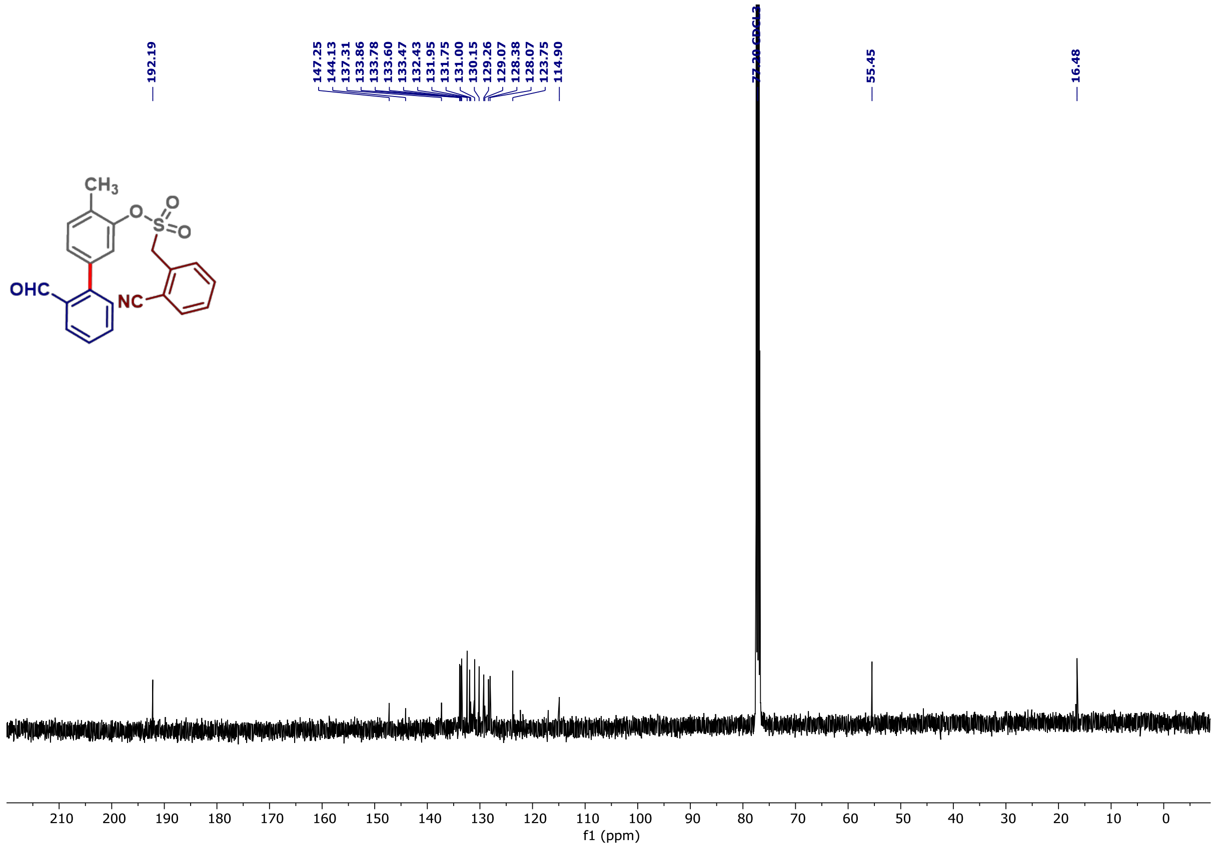
192.19
147.25
144.13
137.31
133.86
133.78
133.60
133.47
132.43
131.95
131.75
131.00
130.15
129.26
129.07
128.38
128.07
123.75
114.90
CDCL3
55.45
16.48
CH3
O
S
O
O
OHC
NC
f1 (ppm)

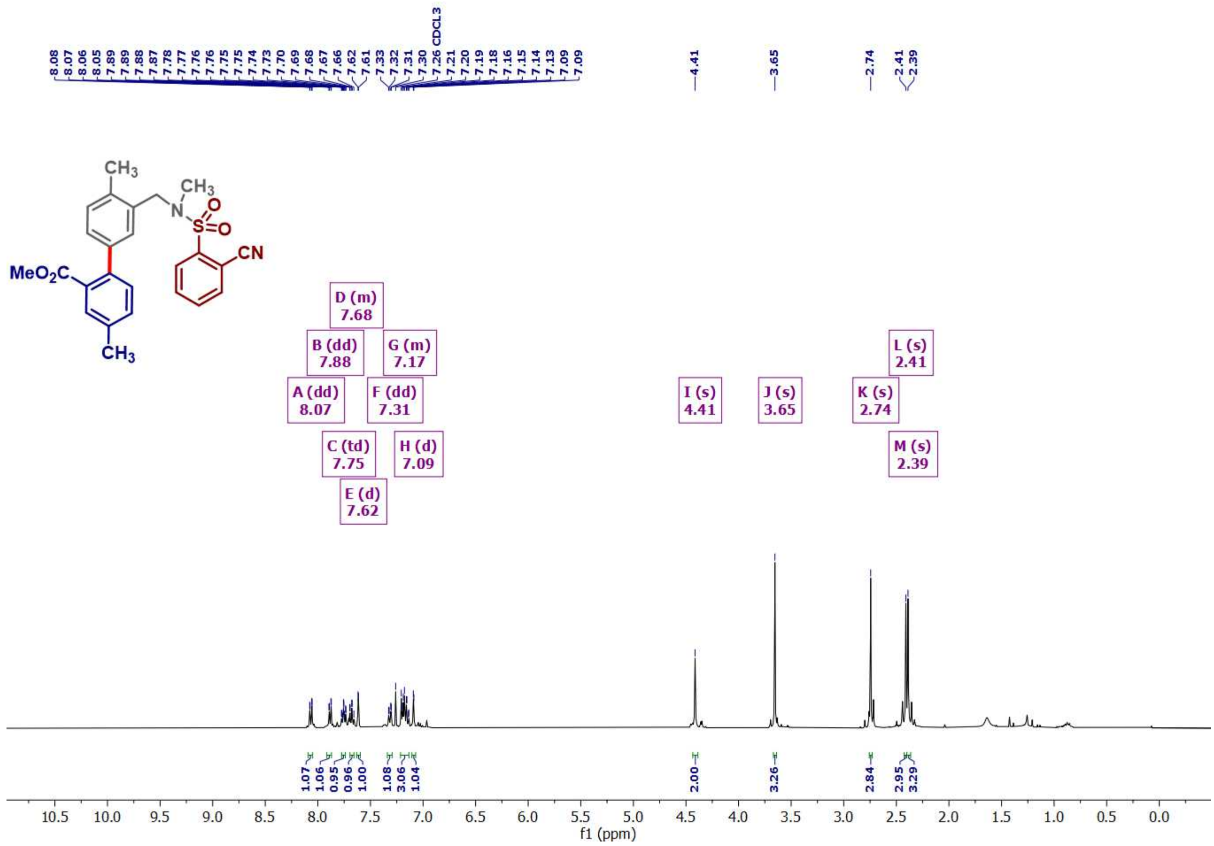
8.08
8.07
8.06
8.05
7.89
7.89
7.88
7.87
7.78
7.77
7.76
7.75
7.75
7.74
7.73
7.70
7.69
7.68
7.67
7.66
7.62
7.61
7.33
7.32
7.31
7.30
7.26 CDCL3
7.21
7.20
7.19
7.18
7.16
7.15
7.14
7.13
7.09
7.09
4.41
3.65
2.74
2.41
2.39
CH_3
CH_3
N
S
O
O
CN
MeO_2C
CH_3
A (dd) 8.07
B (dd) 7.88
C (td) 7.75
D (m) 7.68
E (d) 7.62
F (dd) 7.31
G (m) 7.17
H (d) 7.09
I (s) 4.41
J (s) 3.65
K (s) 2.74
L (s) 2.41
M (s) 2.39
1.07
1.06
0.95
0.96
1.00
1.08
3.06
1.04
2.00
3.26
2.84
2.95
3.29
f1 (ppm)

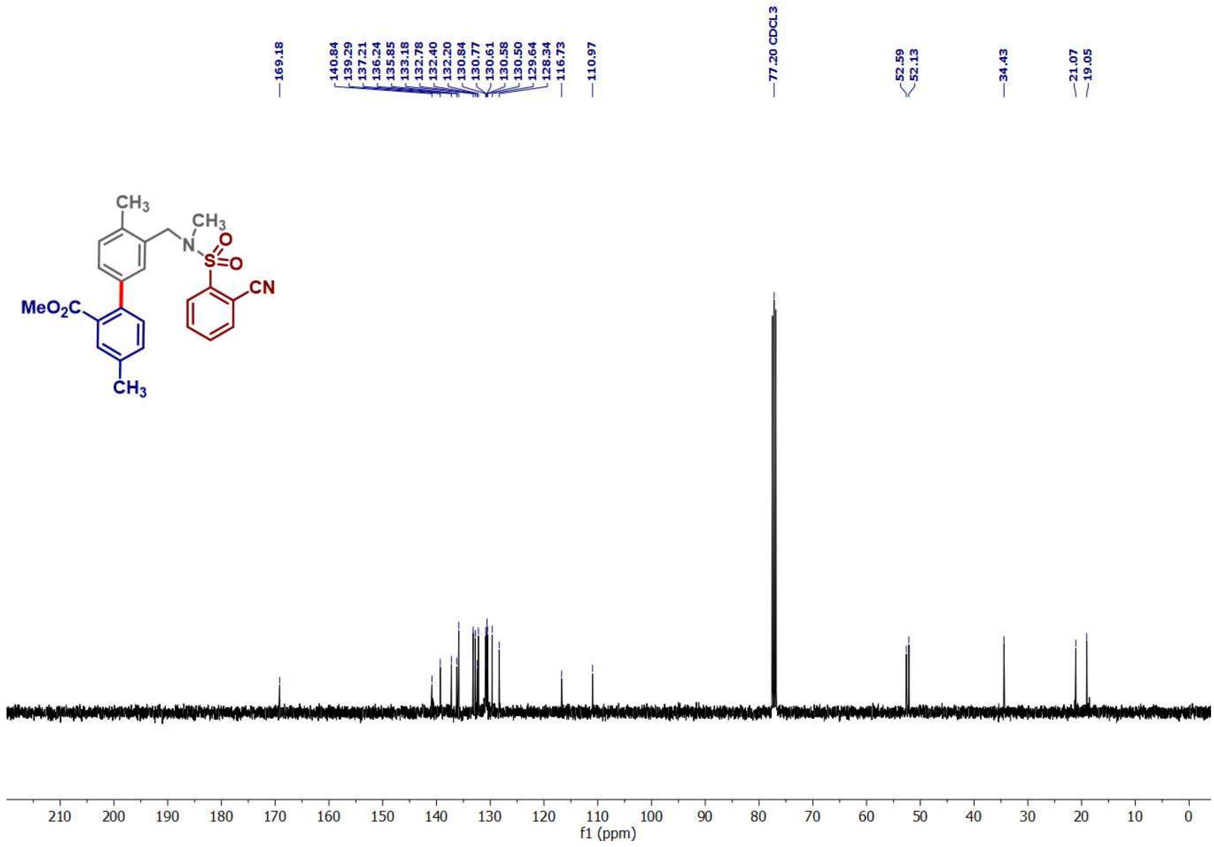
169.18
140.84
139.29
137.21
136.24
135.85
133.18
132.78
132.40
132.20
130.84
130.77
130.61
130.58
130.50
129.64
128.34
116.73
110.97
77.20 CDCL3
52.59
52.13
34.43
21.07
19.05
CH_3
CH_3
N
S
O
O
CN
MeO_2C
CH_3
f1 (ppm)

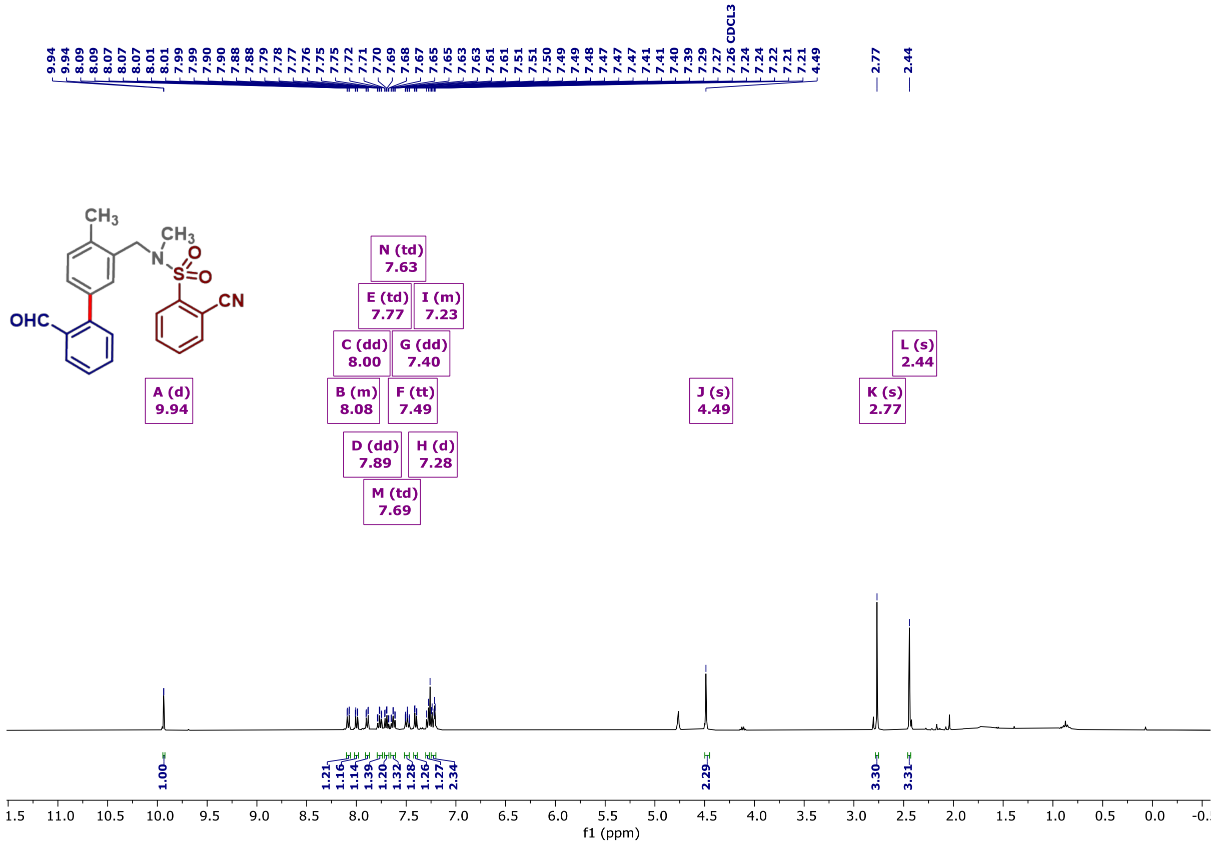
9.94 9.94 8.09 8.09 8.07 8.07 8.07 8.01 8.01 7.99 7.99 7.90 7.90 7.88 7.88 7.79 7.78 7.77 7.76 7.75 7.75 7.72 7.71 7.70 7.69 7.68 7.67 7.65 7.65 7.63 7.63 7.61 7.61 7.51 7.51 7.50 7.49 7.49 7.48 7.47 7.47 7.41 7.41 7.40 7.39 7.29 7.27 7.26 CDCL3 7.24 7.24 7.22 7.21 7.21 7.21 4.49
2.77
2.44
CH3
CH3
N
S
O
O
CN
OHC
N (td) 7.63
E (td) 7.77
I (m) 7.23
C (dd) 8.00
G (dd) 7.40
L (s) 2.44
A (d) 9.94
B (m) 8.08
F (tt) 7.49
J (s) 4.49
K (s) 2.77
D (dd) 7.89
H (d) 7.28
M (td) 7.69
1.00
1.21 1.16 1.14 1.39 1.20 1.32 1.28 1.26 1.27 2.34
2.29
3.30
3.31
f1 (ppm)

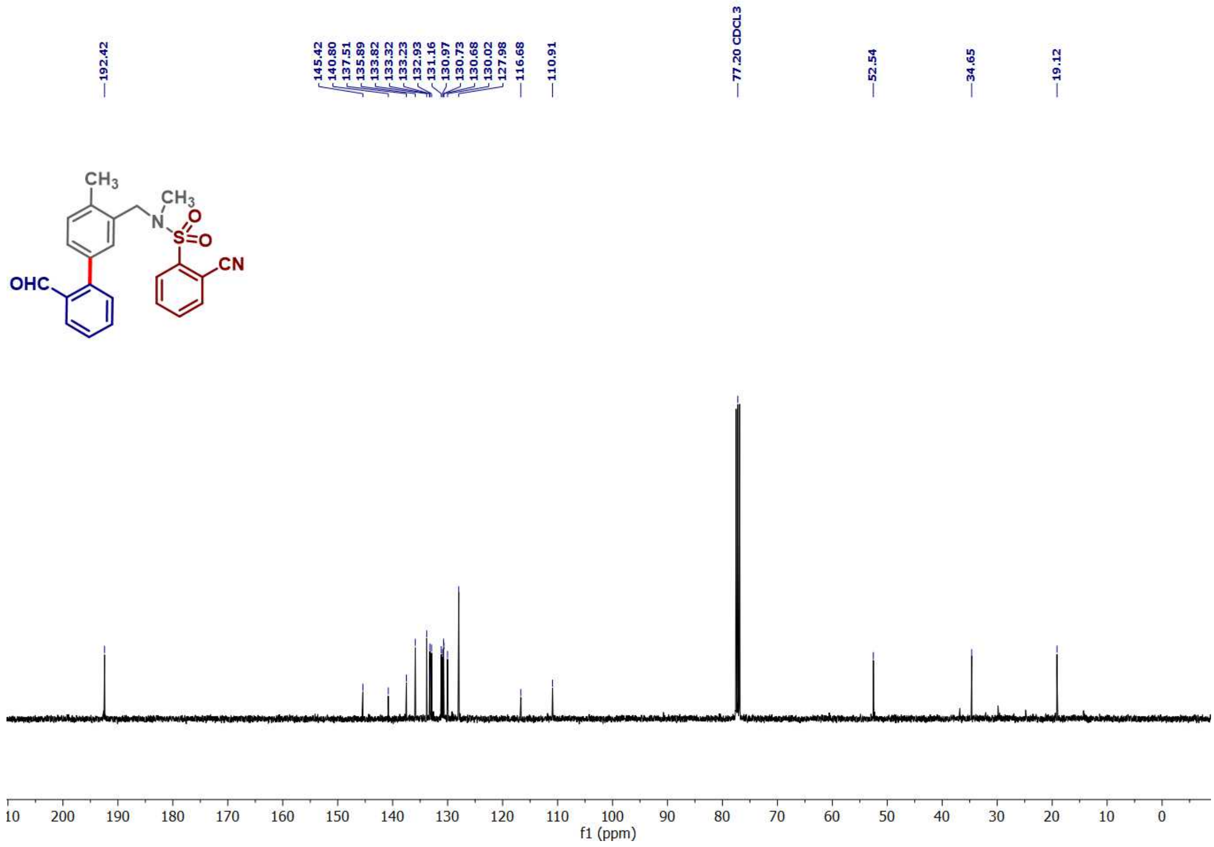
192.42
145.42 140.80 137.51 135.89 133.82 133.32 133.23 132.93 131.16 130.97 130.73 130.68 130.02 127.98
116.68
110.91
77.20 CDCL3
52.54
34.65
19.12
CH3
CH3
N
S
O
O
CN
OHC
f1 (ppm)

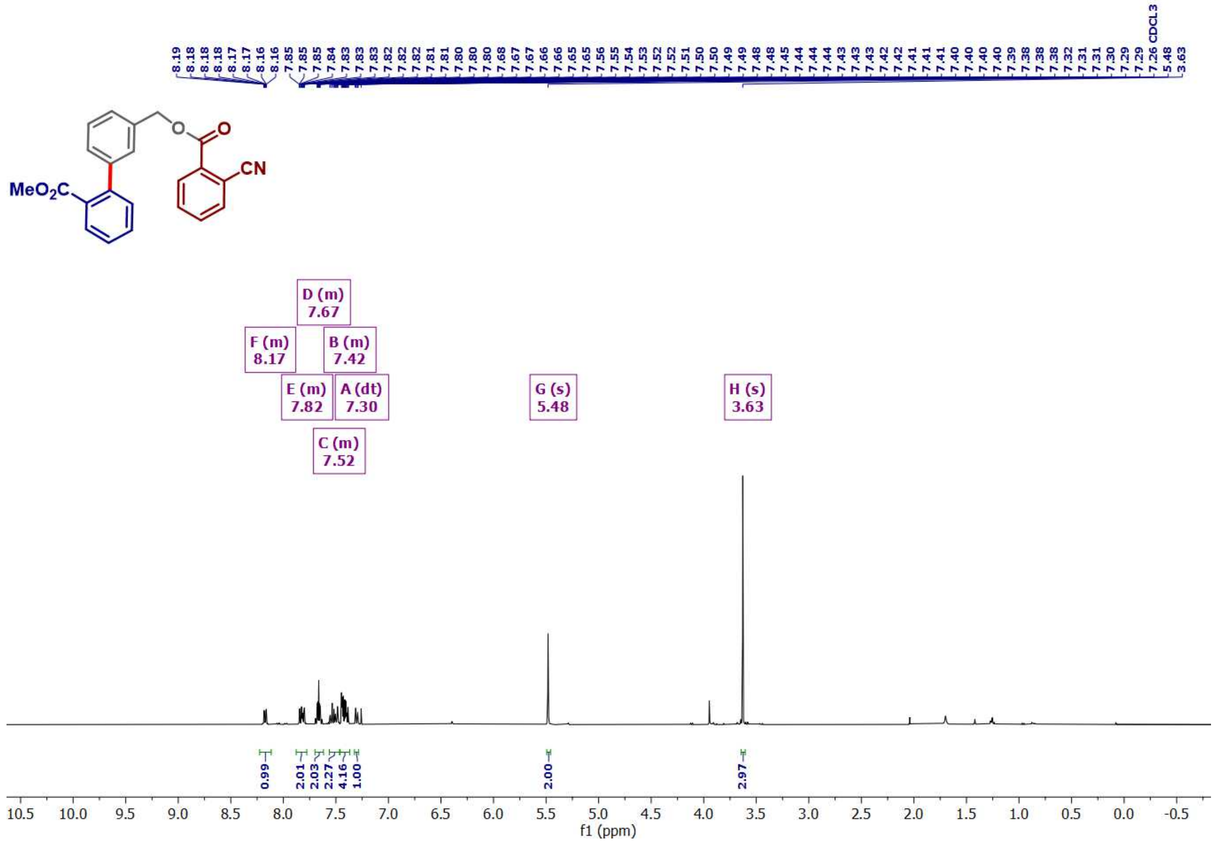

8.19 8.18 8.18 8.17 8.17 8.16 8.16 7.85 7.85 7.85 7.84 7.83 7.83 7.83 7.82 7.82 7.81 7.81 7.80 7.80 7.80 7.68 7.67 7.67 7.66 7.66 7.65 7.65 7.56 7.55 7.54 7.53 7.52 7.52 7.51 7.50 7.50 7.49 7.49 7.48 7.48 7.45 7.44 7.44 7.43 7.43 7.42 7.42 7.41 7.41 7.40 7.40 7.40 7.39 7.38 7.38 7.38 7.32 7.31 7.30 7.29 7.29 7.26 CDCL3 5.48 3.63
MeO2C
O
CN
D (m) 7.67
F (m) 8.17
B (m) 7.42
E (m) 7.82
A (dt) 7.30
C (m) 7.52
G (s) 5.48
H (s) 3.63
0.99 2.01 2.03 2.27 4.16 1.00 2.00 2.97
f1 (ppm)

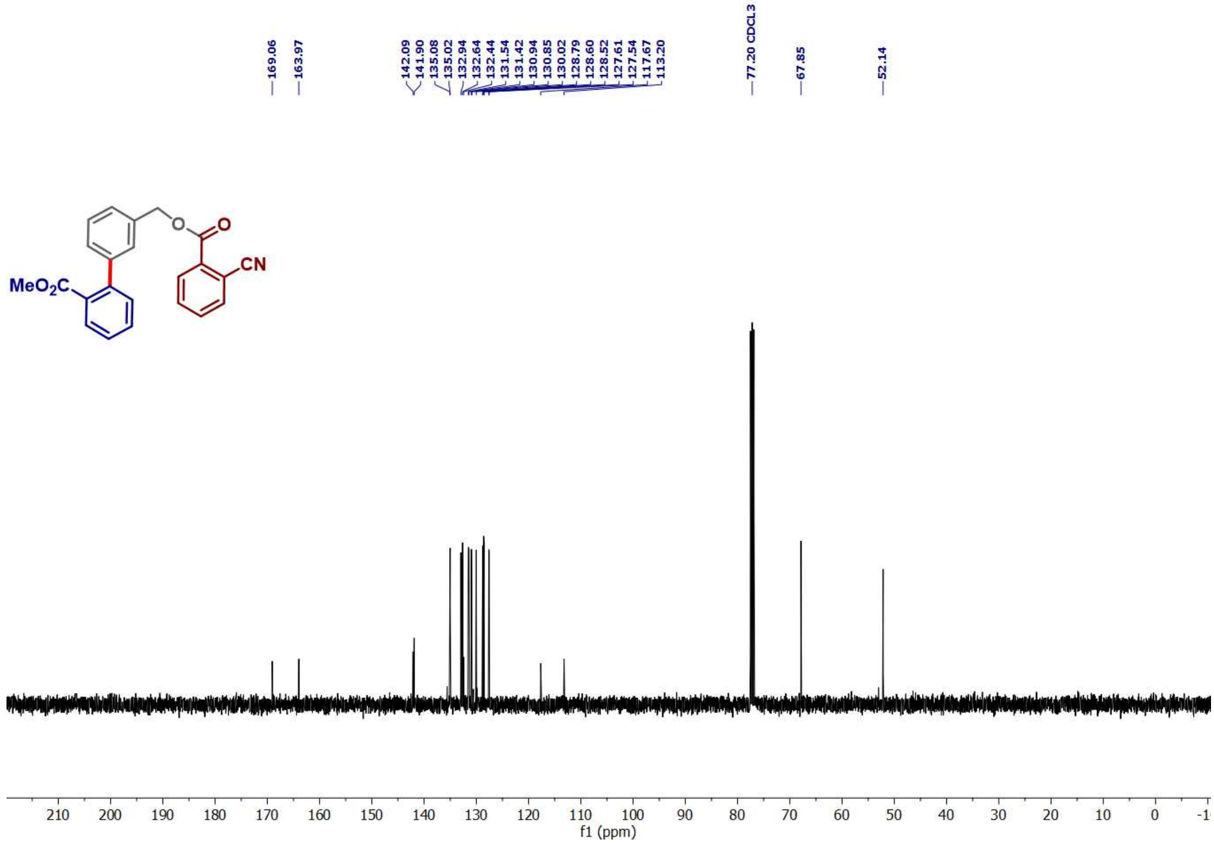

169.06 163.97 142.09 141.90 135.08 135.02 132.94 132.64 132.44 131.54 131.42 130.94 130.85 130.02 128.79 128.60 128.52 127.61 127.54 117.67 113.20 77.20 CDCL3 67.85 52.14
MeO2C
O
CN
f1 (ppm)

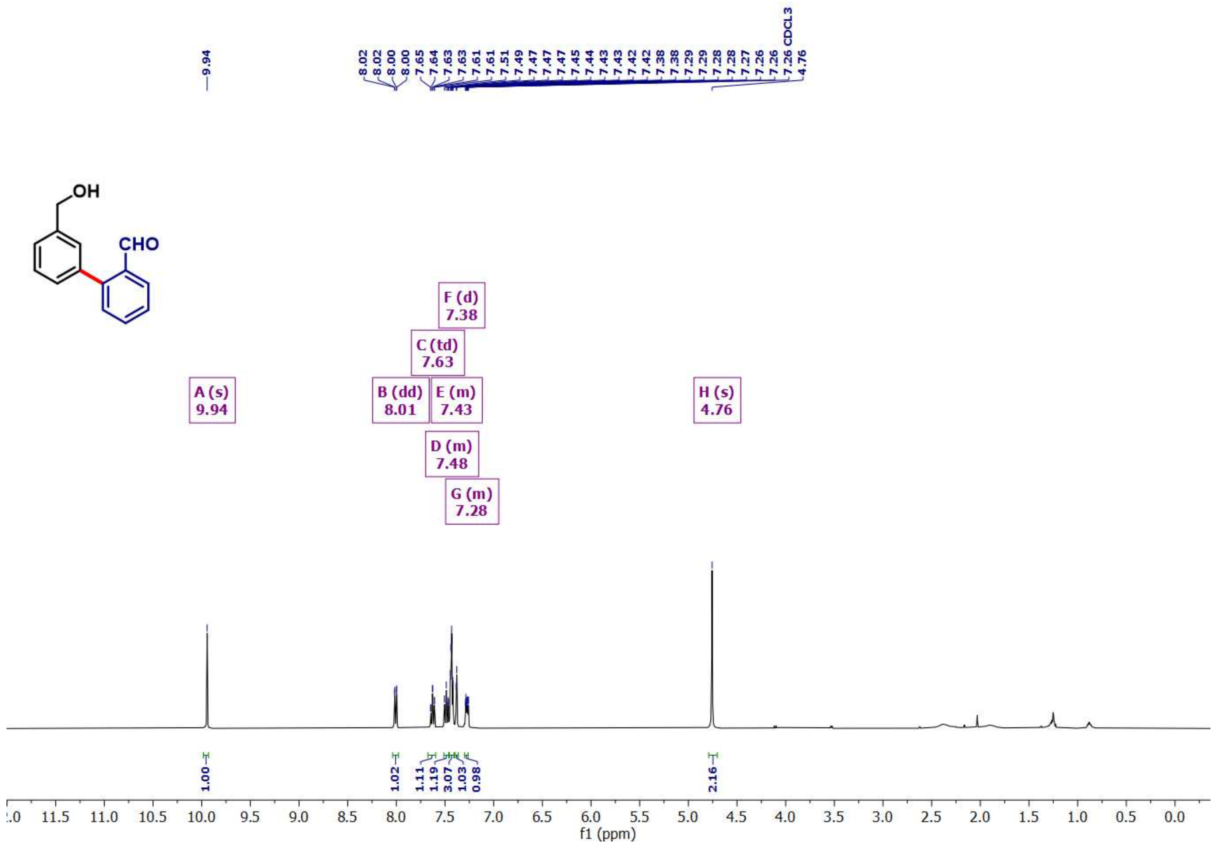
9.94
8.02 8.02 8.00 8.00 7.65 7.64 7.63 7.63 7.61 7.61 7.51 7.49 7.47 7.47 7.45 7.44 7.43 7.43 7.42 7.38 7.38 7.29 7.29 7.28 7.28 7.27 7.26 7.26 CDCL3 4.76
OH
CHO
F (d) 7.38
C (td) 7.63
A (s) 9.94
B (dd) 8.01
E (m) 7.43
H (s) 4.76
D (m) 7.48
G (m) 7.28
1.00
1.02
1.11
1.19
3.07
1.03
0.98
2.16
f1 (ppm)

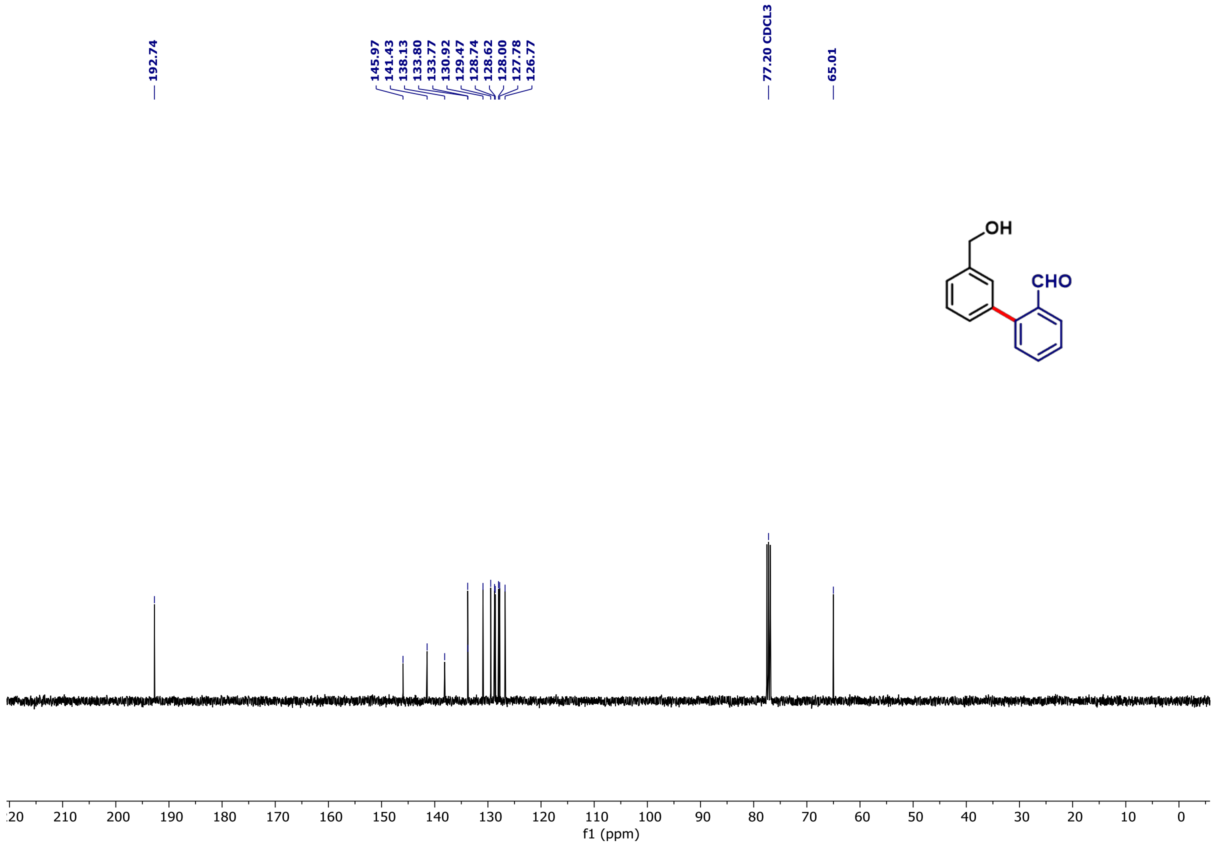
192.74
145.97 141.43 138.13 133.80 133.77 130.92 129.47 128.74 128.62 128.00 127.78 126.77
77.20 CDCL3
65.01
OH
CHO
f1 (ppm)

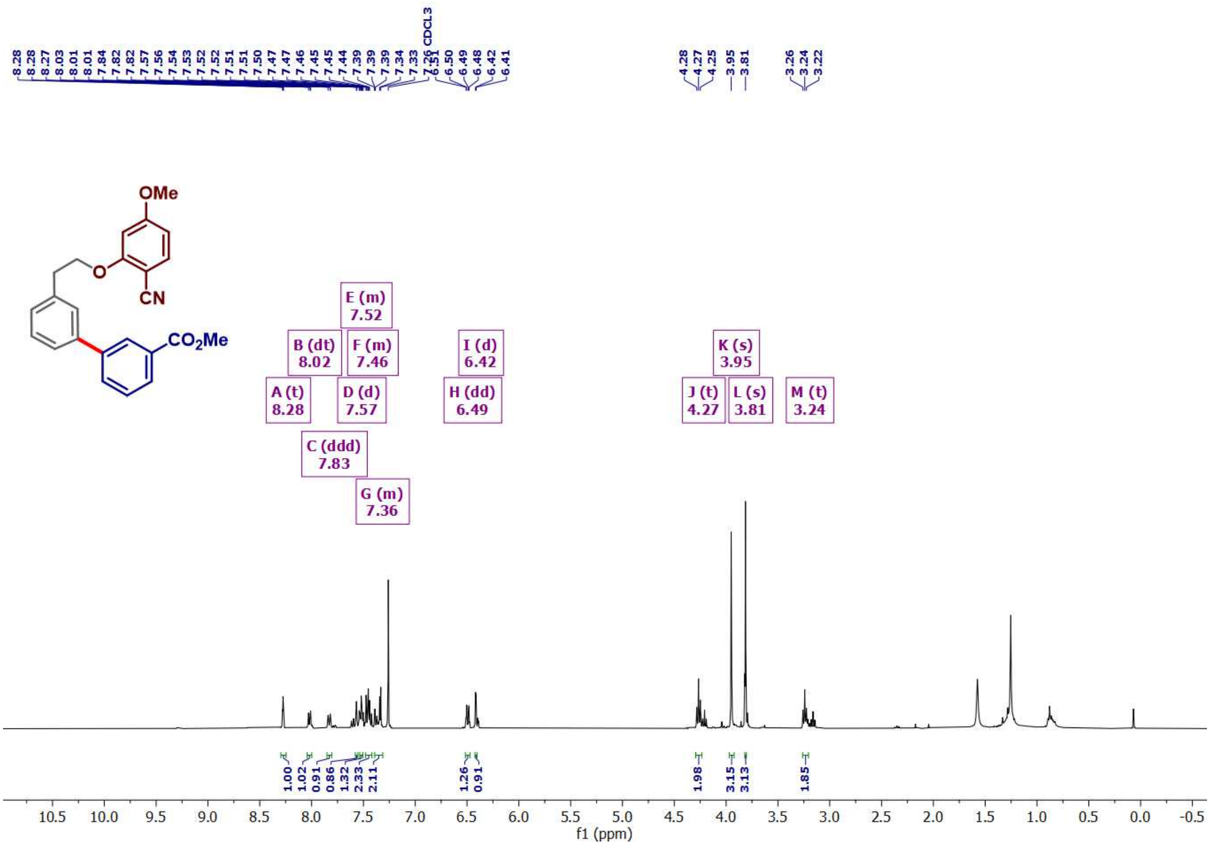
8.28 8.28 8.27 8.03 8.01 8.01 7.84 7.82 7.82 7.57 7.56 7.54 7.53 7.52 7.52 7.51 7.51 7.50 7.47 7.46 7.45 7.45 7.44 7.39 7.39 7.39 7.34 7.33 7.26 CDCL3 6.51 6.50 6.49 6.48 6.42 6.41
4.28 4.27 4.25 3.95 3.81 3.26 3.24 3.22
OMe
CN
CO_2Me
O
E (m) 7.52
B (dt) 8.02
F (m) 7.46
A (t) 8.28
D (d) 7.57
C (ddd) 7.83
G (m) 7.36
I (d) 6.42
H (dd) 6.49
K (s) 3.95
J (t) 4.27
L (s) 3.81
M (t) 3.24
1.00 1.02 0.91 0.86 1.32 2.33 2.11 1.26 0.91 1.98 3.15 3.13 1.85
f1 (ppm)

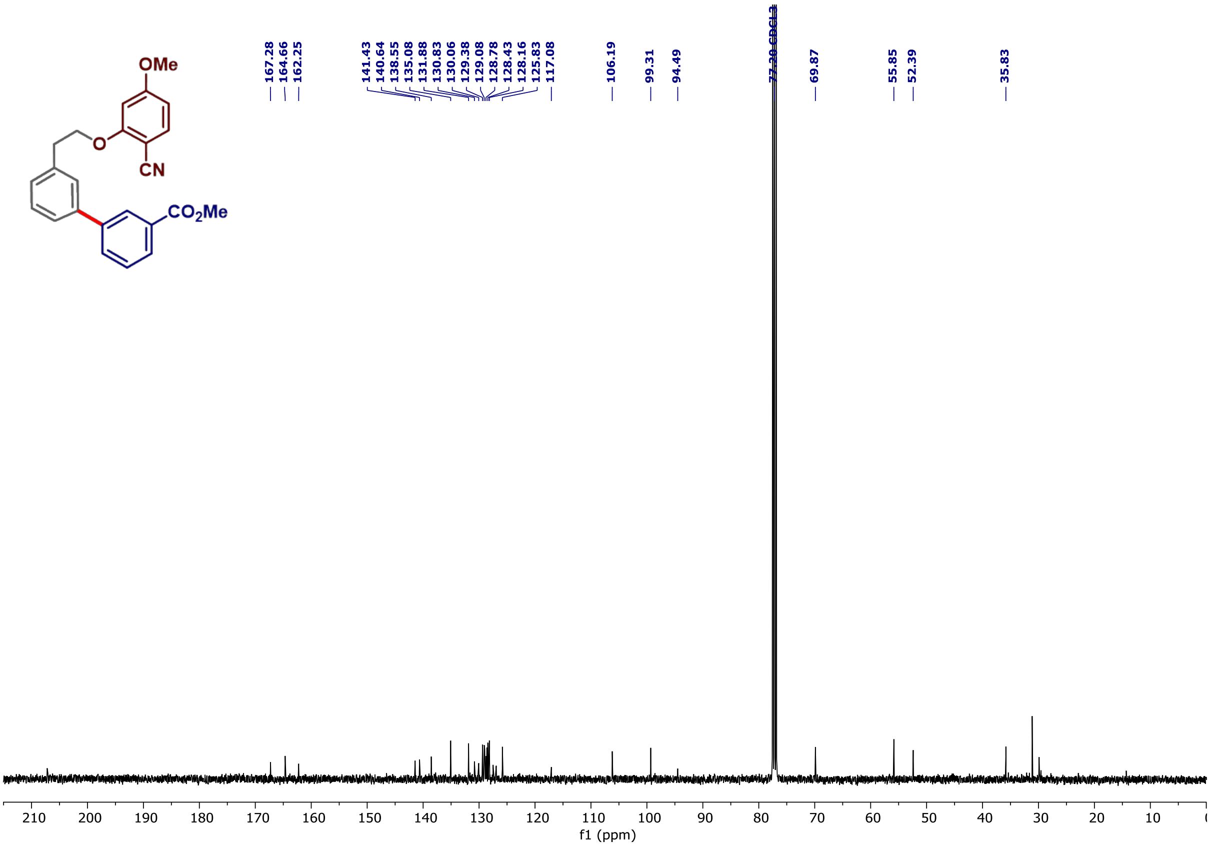
167.28 164.66 162.25 141.43 140.64 138.55 135.08 131.88 130.83 130.06 129.38 129.08 128.78 128.43 128.16 125.83 117.08 106.19 99.31 94.49 77.20 CDCL3 69.87 55.85 52.39 35.83
OMe
CN
CO_2Me
O
f1 (ppm)

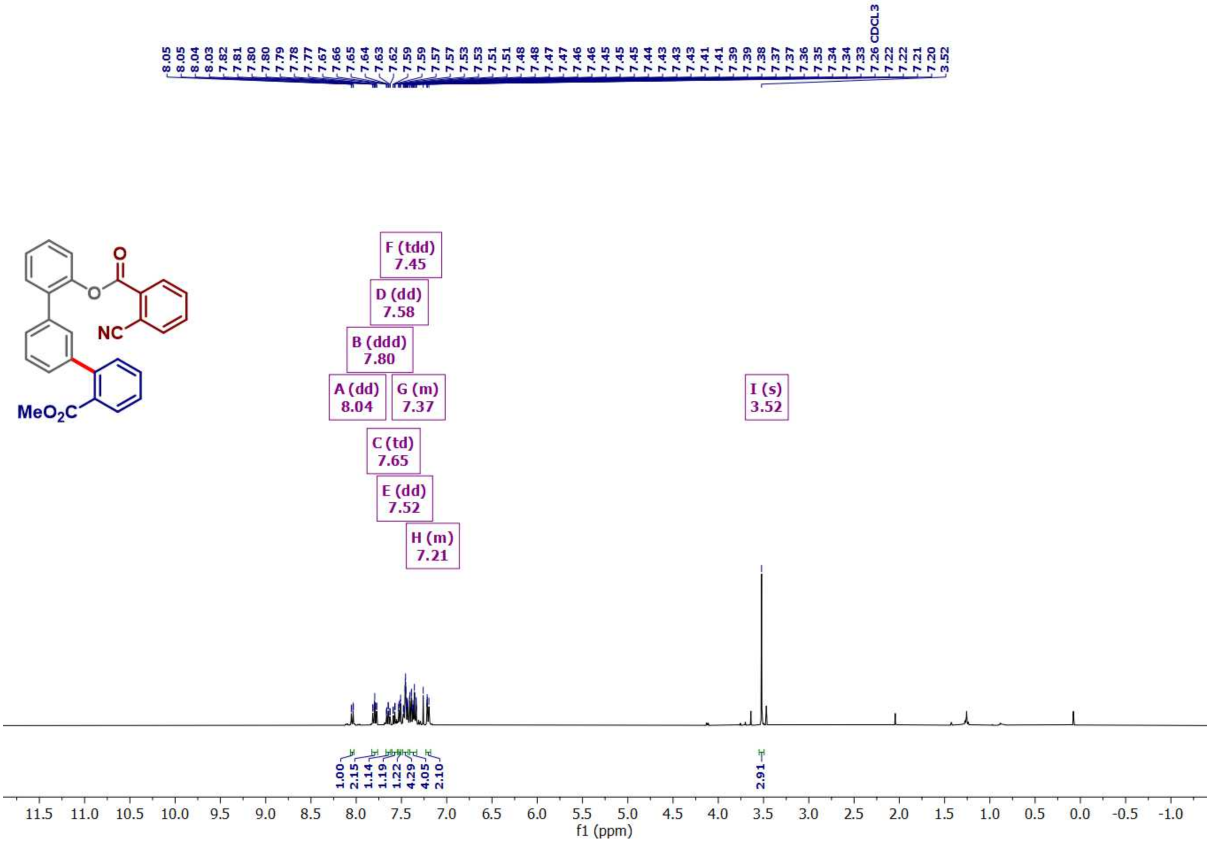

8.05
8.05
8.04
8.03
7.82
7.81
7.80
7.80
7.79
7.78
7.77
7.67
7.66
7.65
7.64
7.63
7.62
7.59
7.59
7.57
7.57
7.53
7.53
7.51
7.51
7.48
7.48
7.47
7.46
7.45
7.45
7.45
7.44
7.43
7.43
7.43
7.41
7.41
7.39
7.39
7.38
7.37
7.37
7.36
7.35
7.34
7.34
7.33
7.26 CDCL3
7.22
7.22
7.21
7.20
3.52
F (tdd) 7.45
D (dd) 7.58
B (ddd) 7.80
A (dd) 8.04
G (m) 7.37
I (s) 3.52
C (td) 7.65
E (dd) 7.52
H (m) 7.21
1.00
2.15
1.14
1.19
1.22
4.29
4.05
2.10
2.91
f1 (ppm)

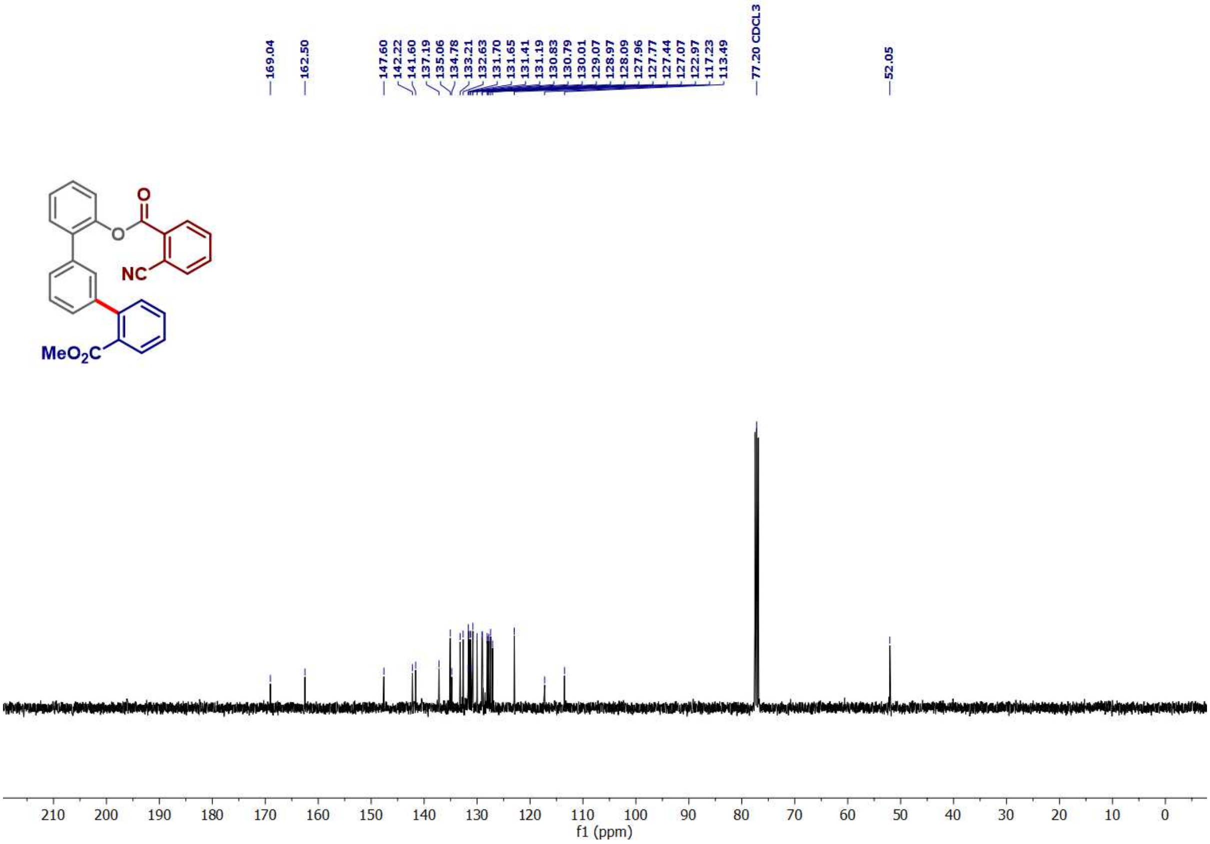

169.04
162.50
147.60
142.22
141.60
137.19
135.06
134.78
133.21
132.63
131.70
131.65
131.41
131.19
130.83
130.79
130.01
129.07
128.97
128.09
127.96
127.77
127.44
127.07
122.97
117.23
113.49
77.20 CDCL3
52.05
f1 (ppm)

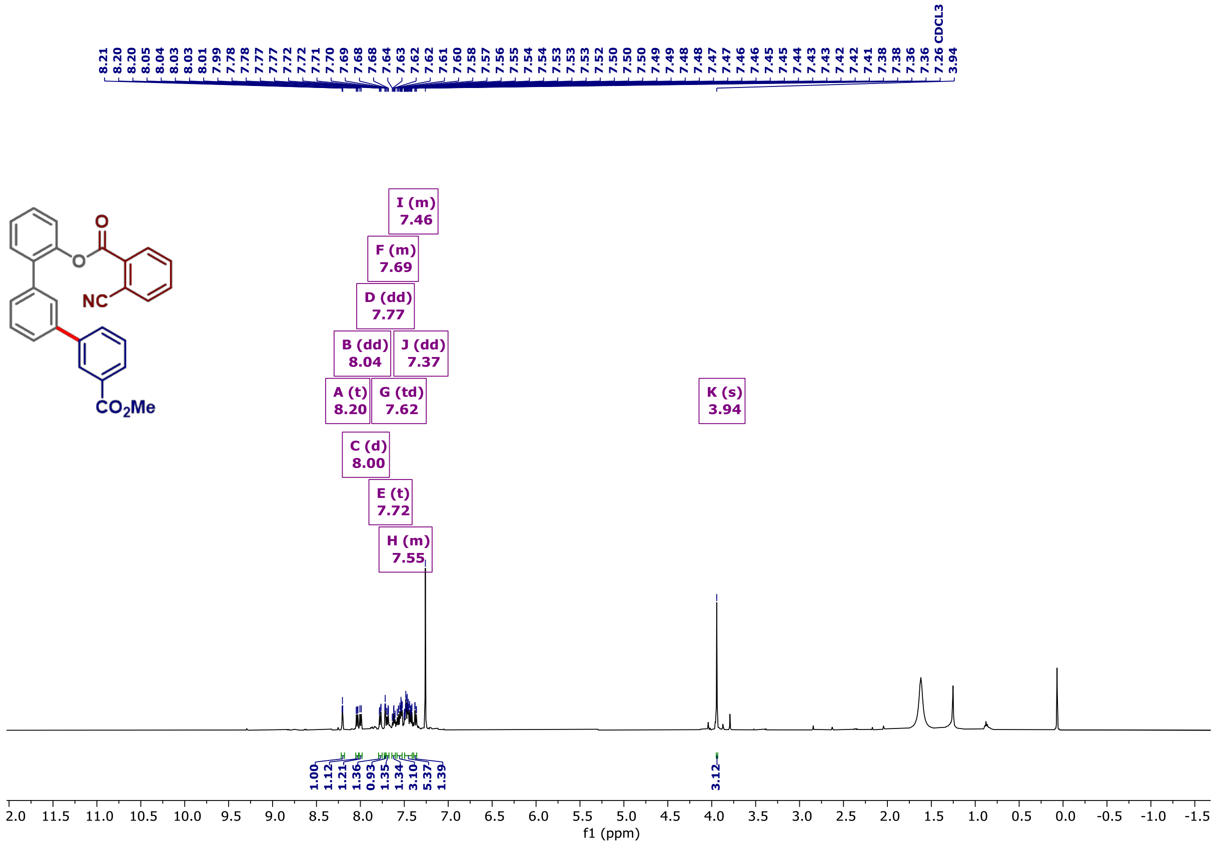

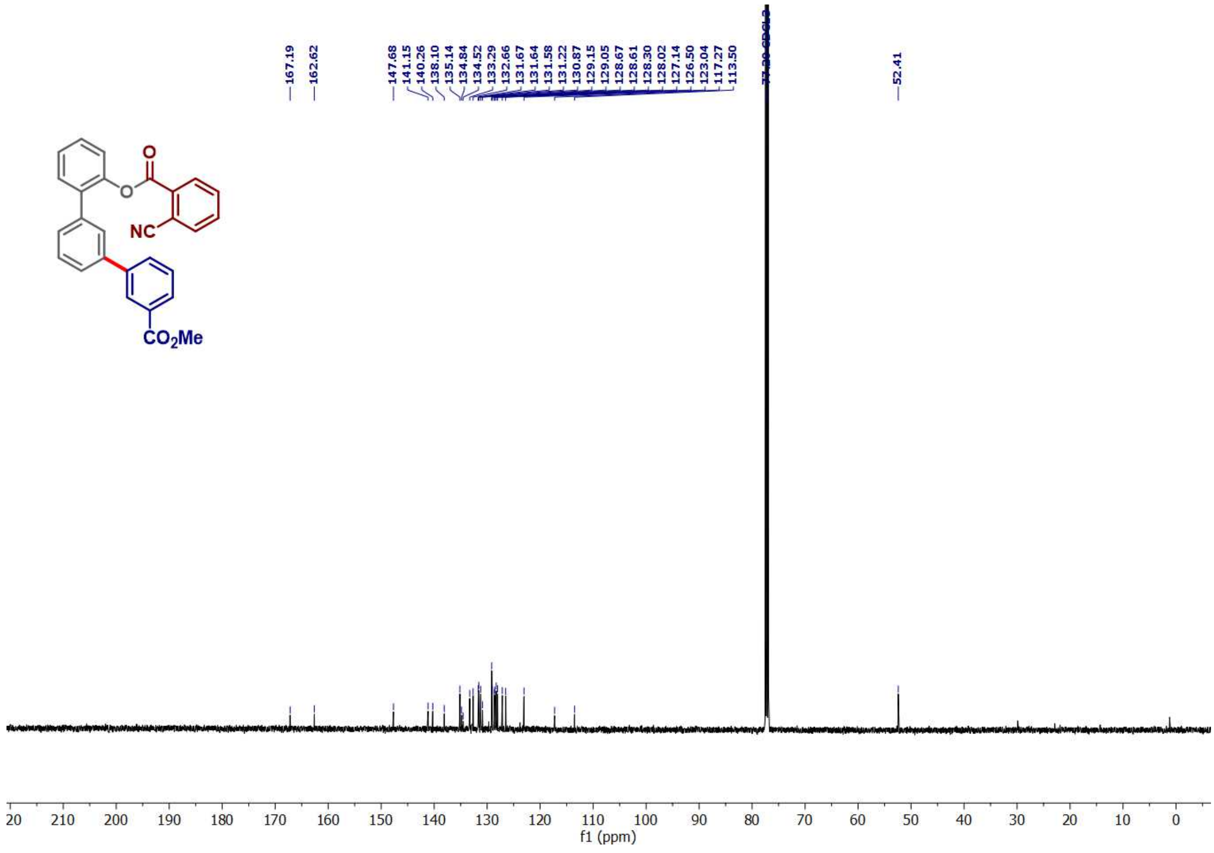

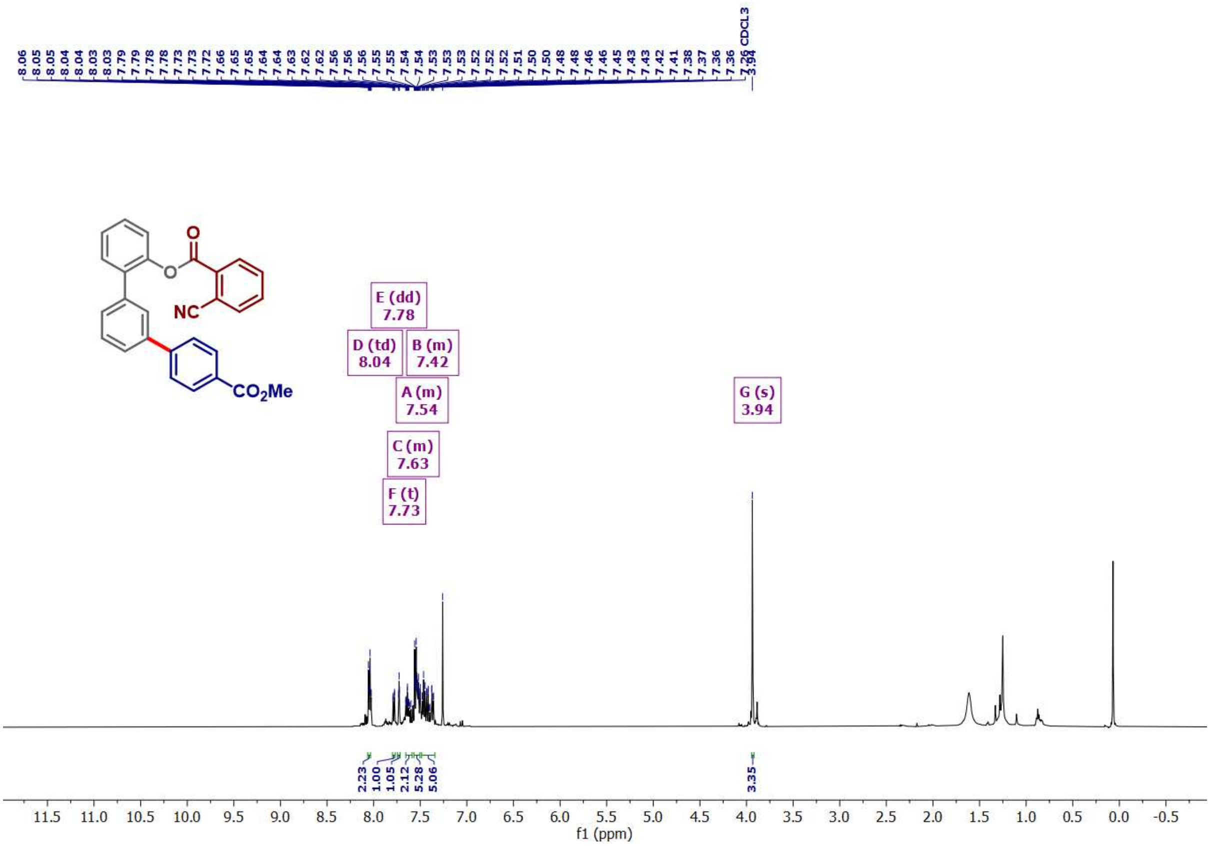

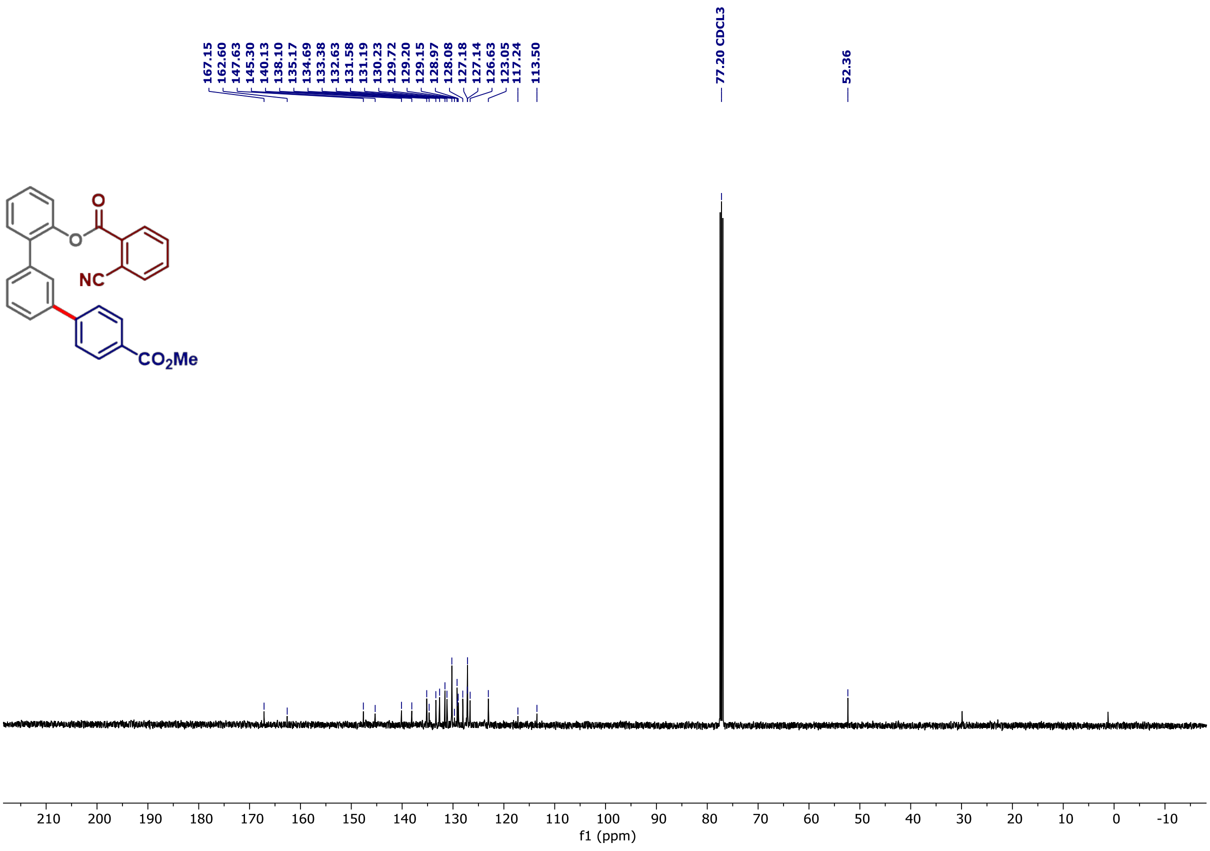

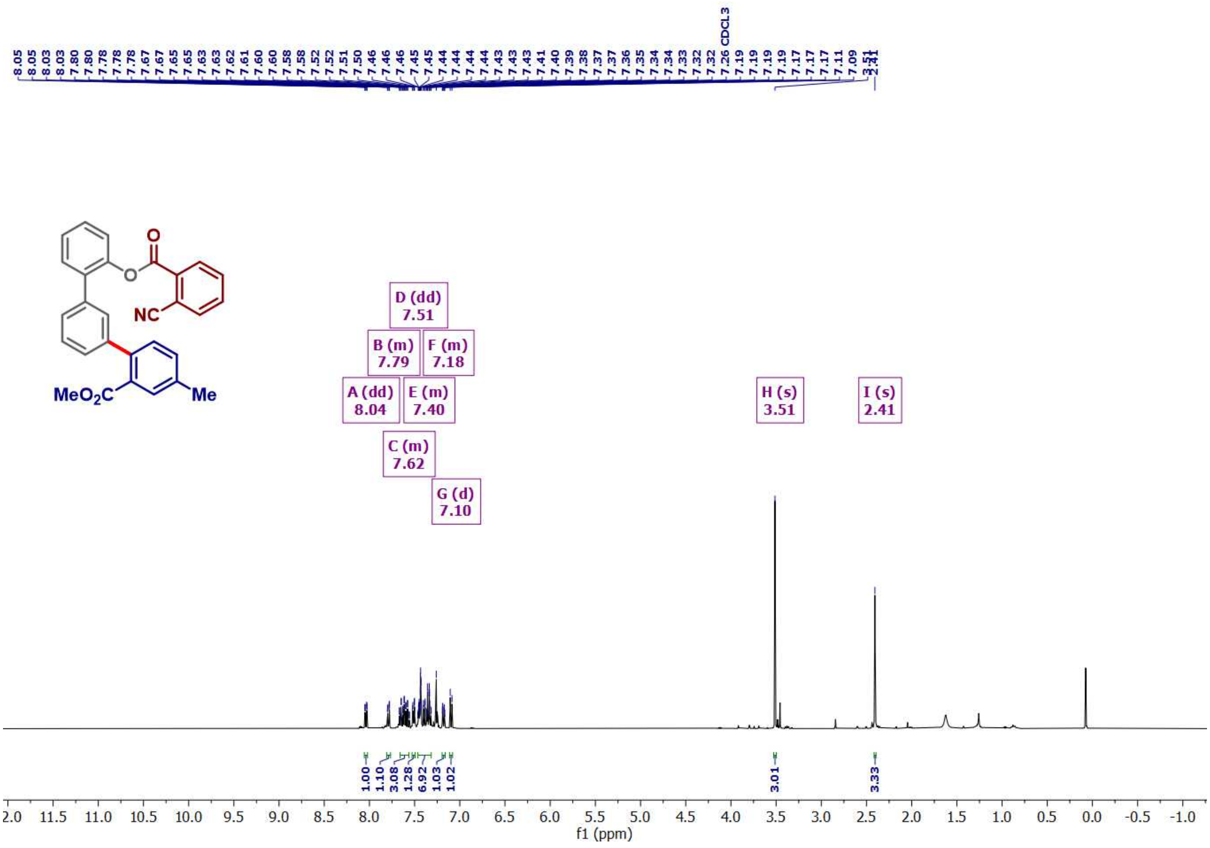
CDCL3
NC
MeO2C
Me
A (dd) 8.04
B (m) 7.79
C (m) 7.62
D (dd) 7.51
E (m) 7.40
F (m) 7.18
G (d) 7.10
H (s) 3.51
I (s) 2.41
1.00
1.10
3.08
1.28
6.92
1.03
1.02
3.01
3.33
f1 (ppm)

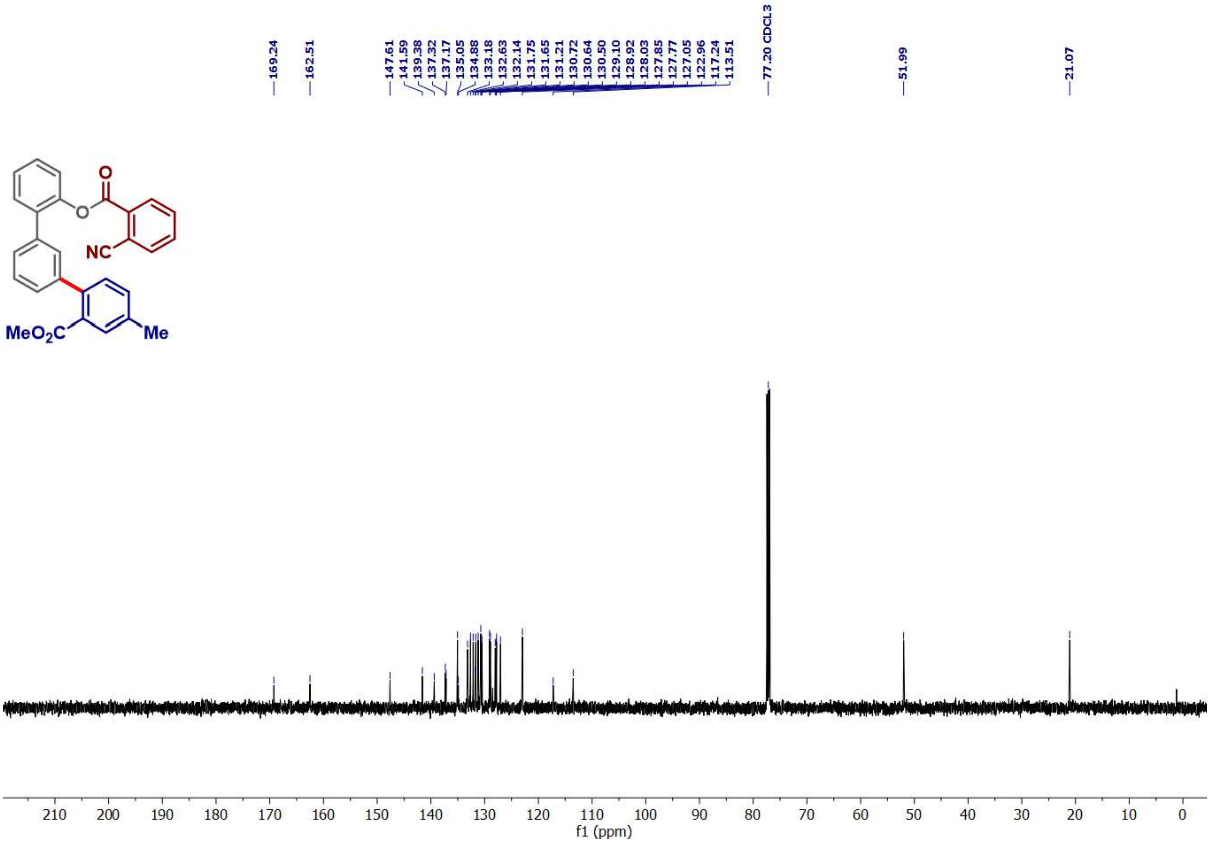
169.24
162.51
147.61
141.59
139.38
137.32
137.17
135.05
134.88
133.18
132.63
132.14
131.75
131.65
131.21
130.72
130.64
130.50
129.10
128.92
128.03
127.85
127.77
127.05
122.96
117.24
113.51
77.20 CDCL3
51.99
21.07
NC
MeO2C
Me
f1 (ppm)

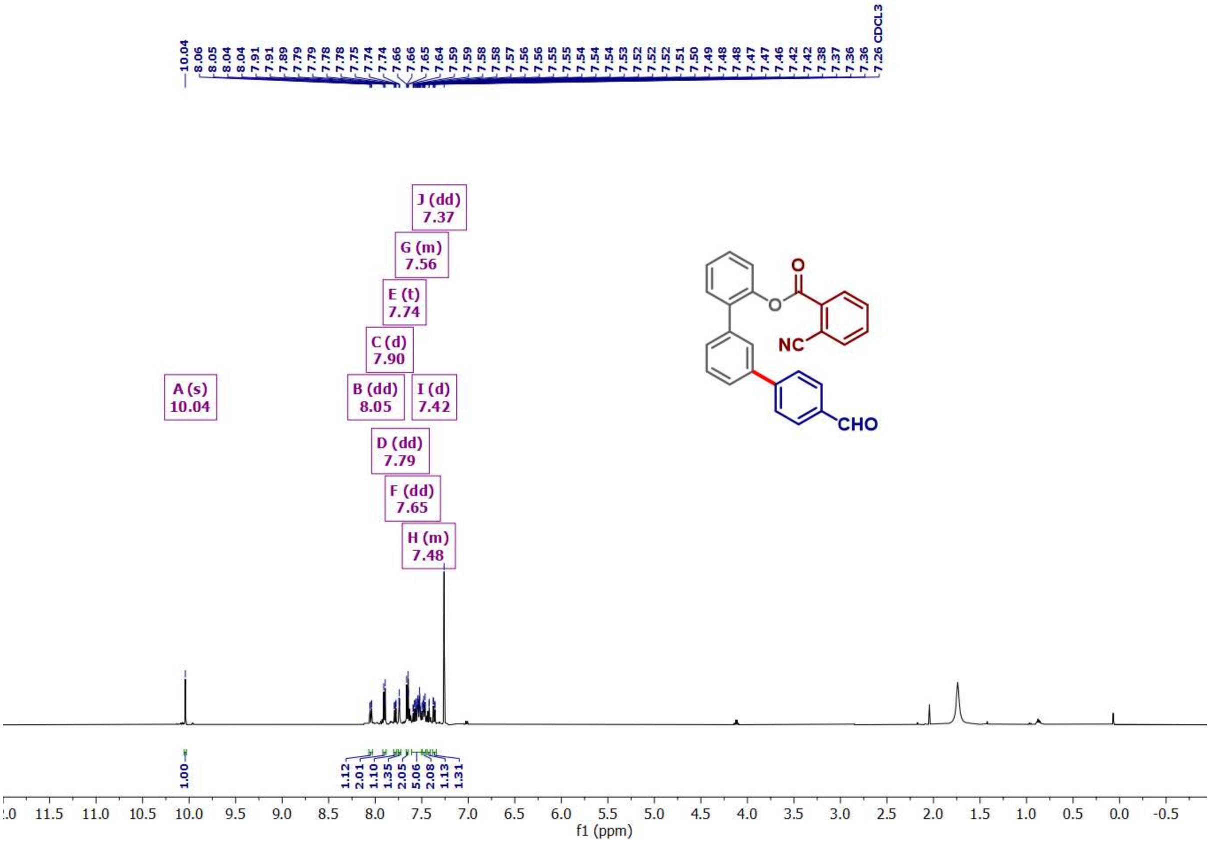
10.04
8.06
8.05
8.04
8.04
7.91
7.91
7.89
7.79
7.79
7.78
7.78
7.75
7.74
7.74
7.66
7.65
7.64
7.59
7.59
7.58
7.58
7.57
7.56
7.56
7.55
7.55
7.54
7.54
7.54
7.53
7.52
7.52
7.52
7.51
7.50
7.49
7.48
7.48
7.47
7.47
7.46
7.42
7.42
7.38
7.37
7.36
7.26 CDCL3
J (dd) 7.37
G (m) 7.56
E (t) 7.74
C (d) 7.90
A (s) 10.04
B (dd) 8.05
I (d) 7.42
D (dd) 7.79
F (dd) 7.65
H (m) 7.48
O
NC
CHO
1.00
1.12
2.01
1.10
1.35
2.05
5.06
2.08
1.13
1.31
f1 (ppm)

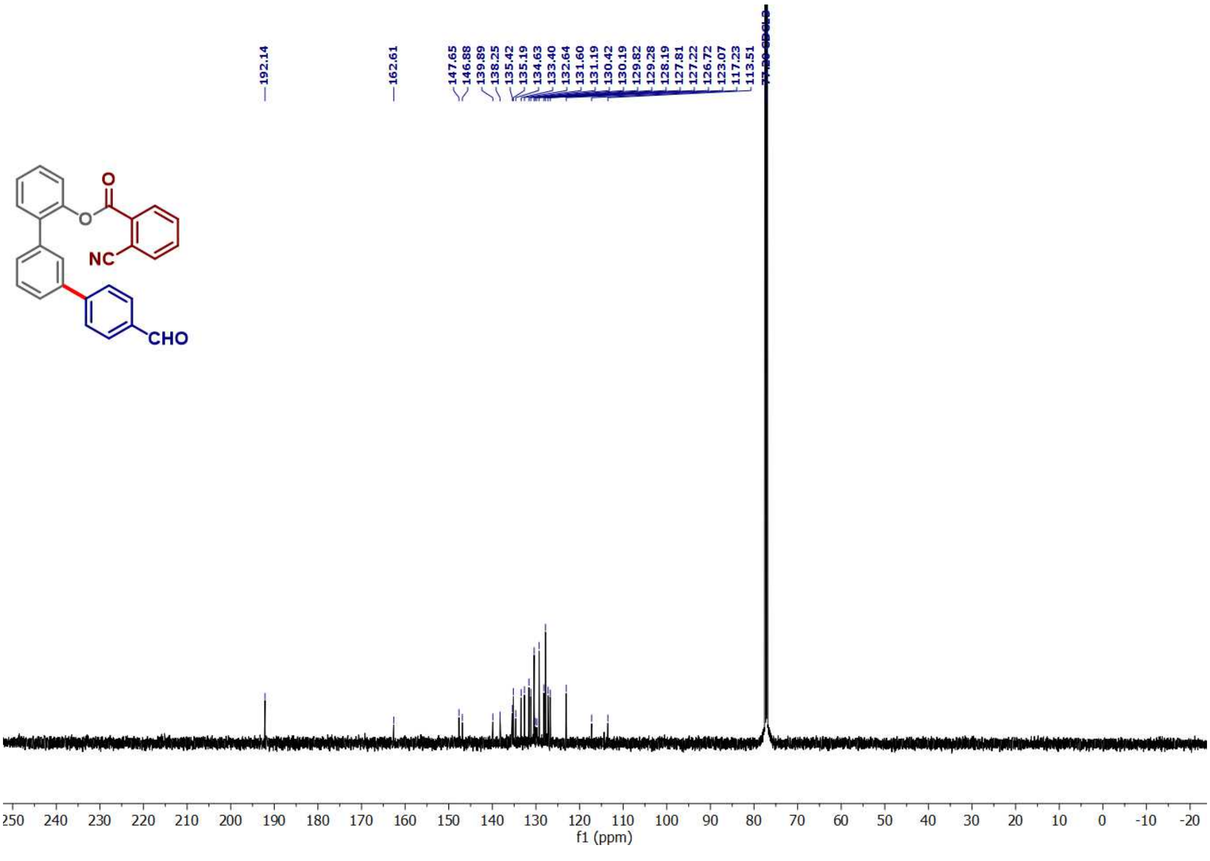
192.14
162.61
147.65
146.88
139.89
138.25
135.42
135.19
134.63
133.40
132.64
131.60
131.19
130.42
130.19
129.82
129.28
128.19
127.81
127.22
126.72
123.07
117.23
113.51
O
NC
CHO
f1 (ppm)

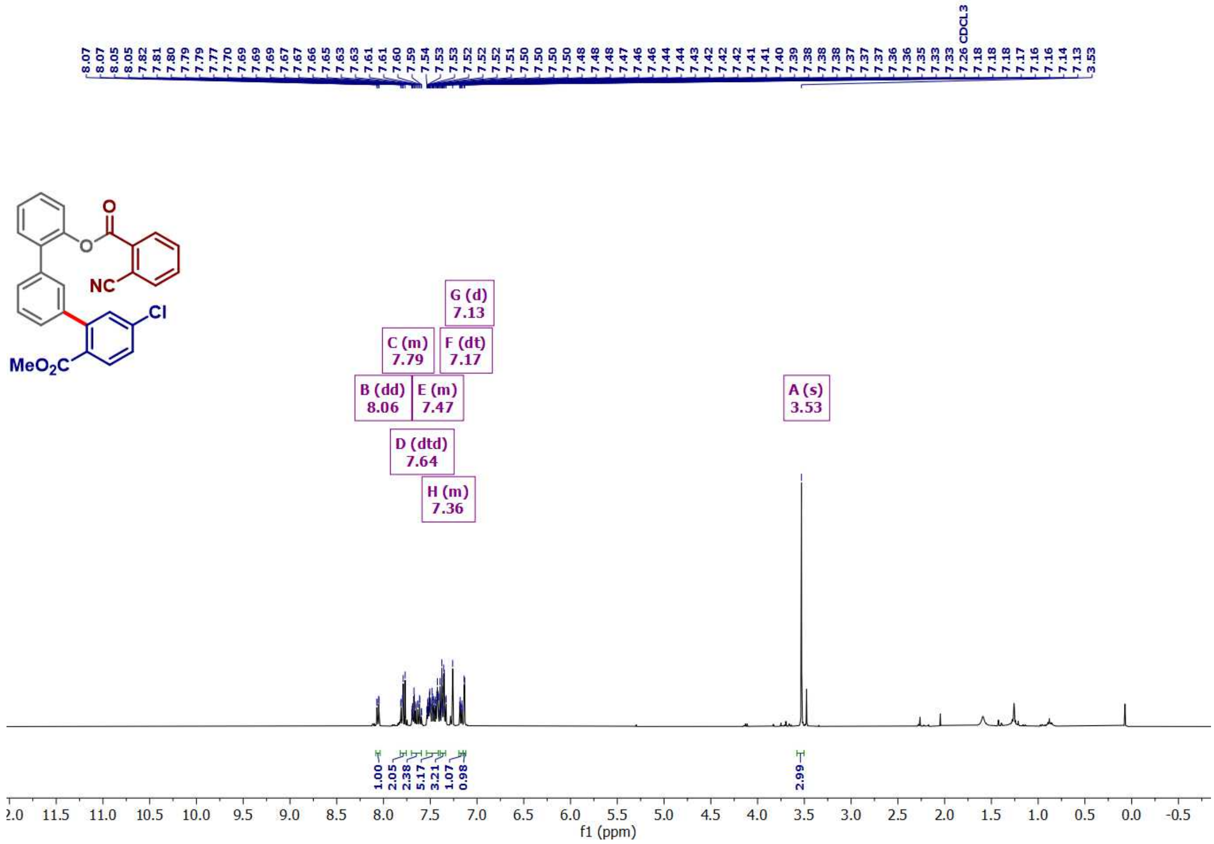
CDCL3
NC
Cl
MeO2C
G (d) 7.13
C (m) 7.79
F (dt) 7.17
B (dd) 8.06
E (m) 7.47
A (s) 3.53
D (dtd) 7.64
H (m) 7.36
1.00
2.05
2.38
5.17
3.21
1.07
0.98
2.99
f1 (ppm)

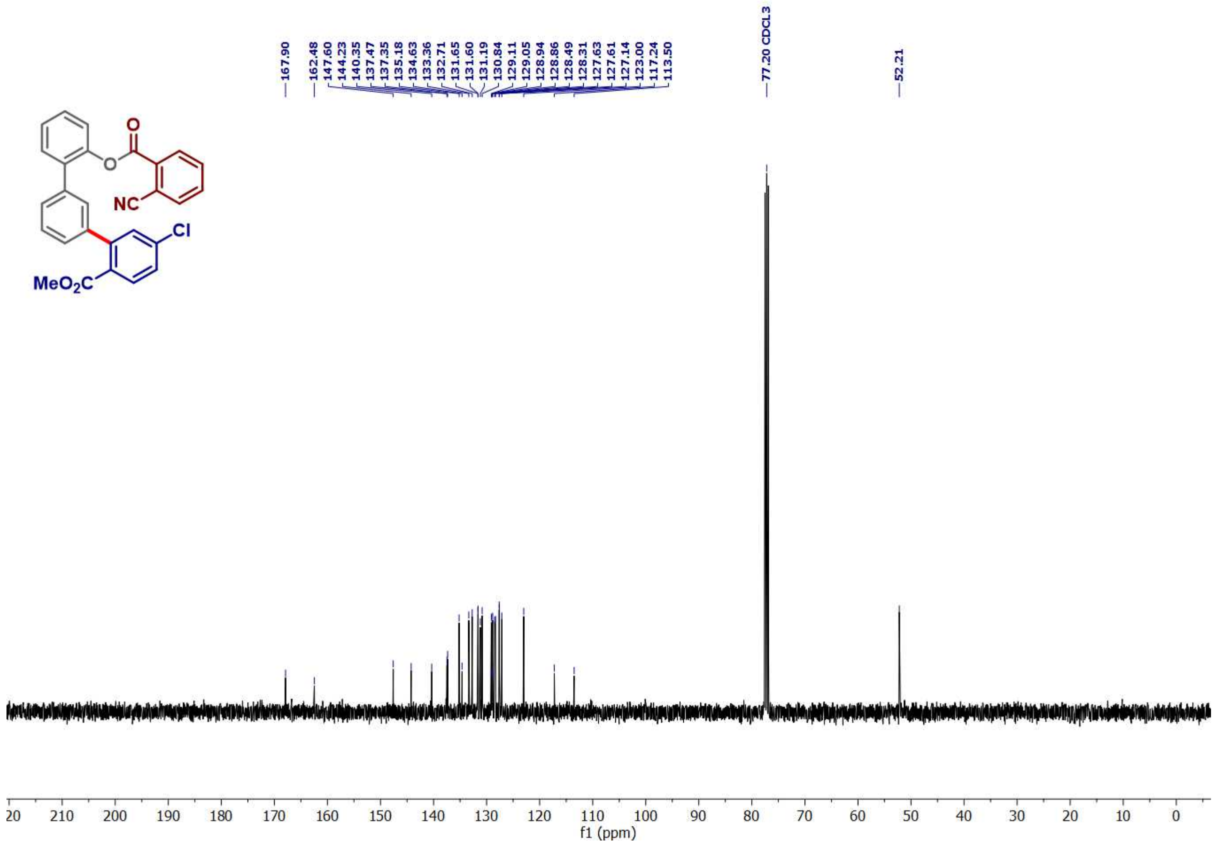
167.90
162.48
147.60
144.23
140.35
137.47
137.35
135.18
134.63
133.36
132.71
131.65
131.60
131.19
130.84
129.11
129.05
128.94
128.86
128.49
128.31
127.63
127.61
127.14
123.00
117.24
113.50
77.20 CDCL3
52.21
NC
Cl
MeO2C
f1 (ppm)

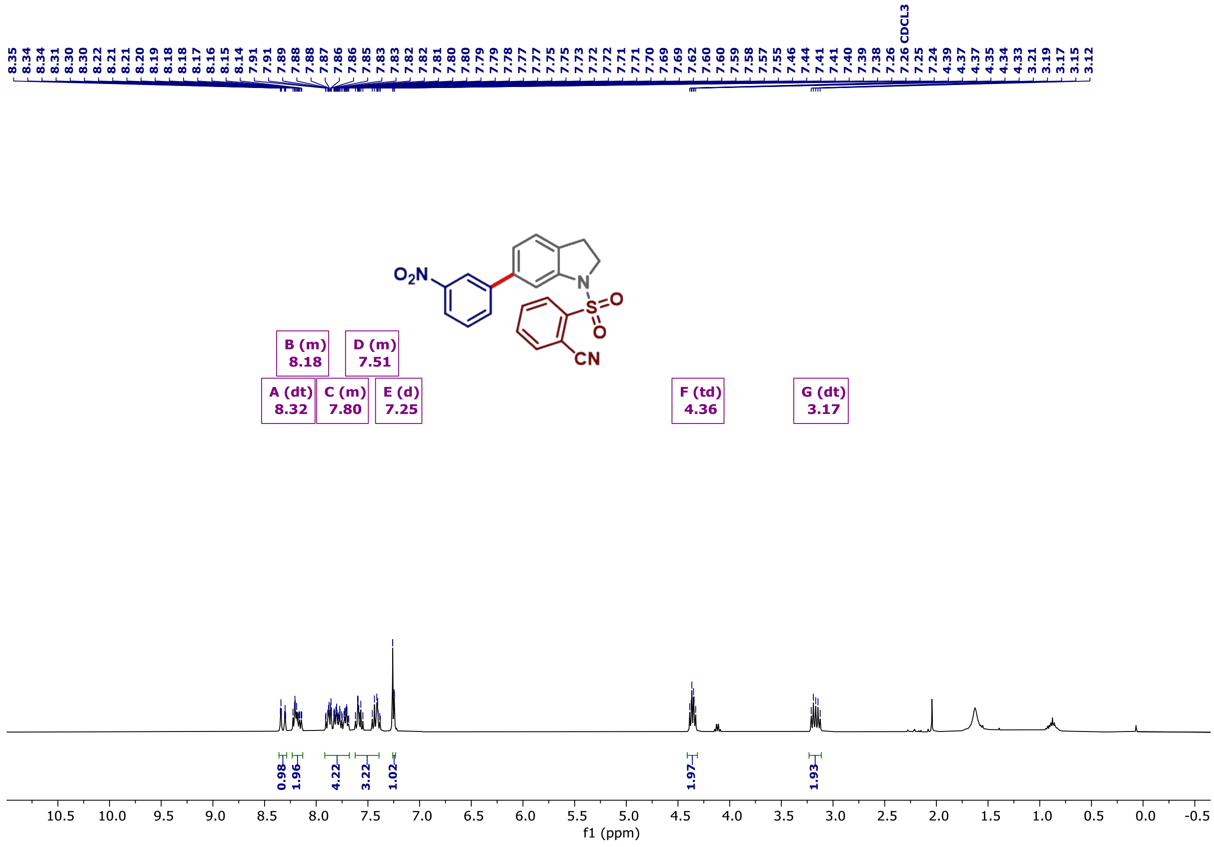

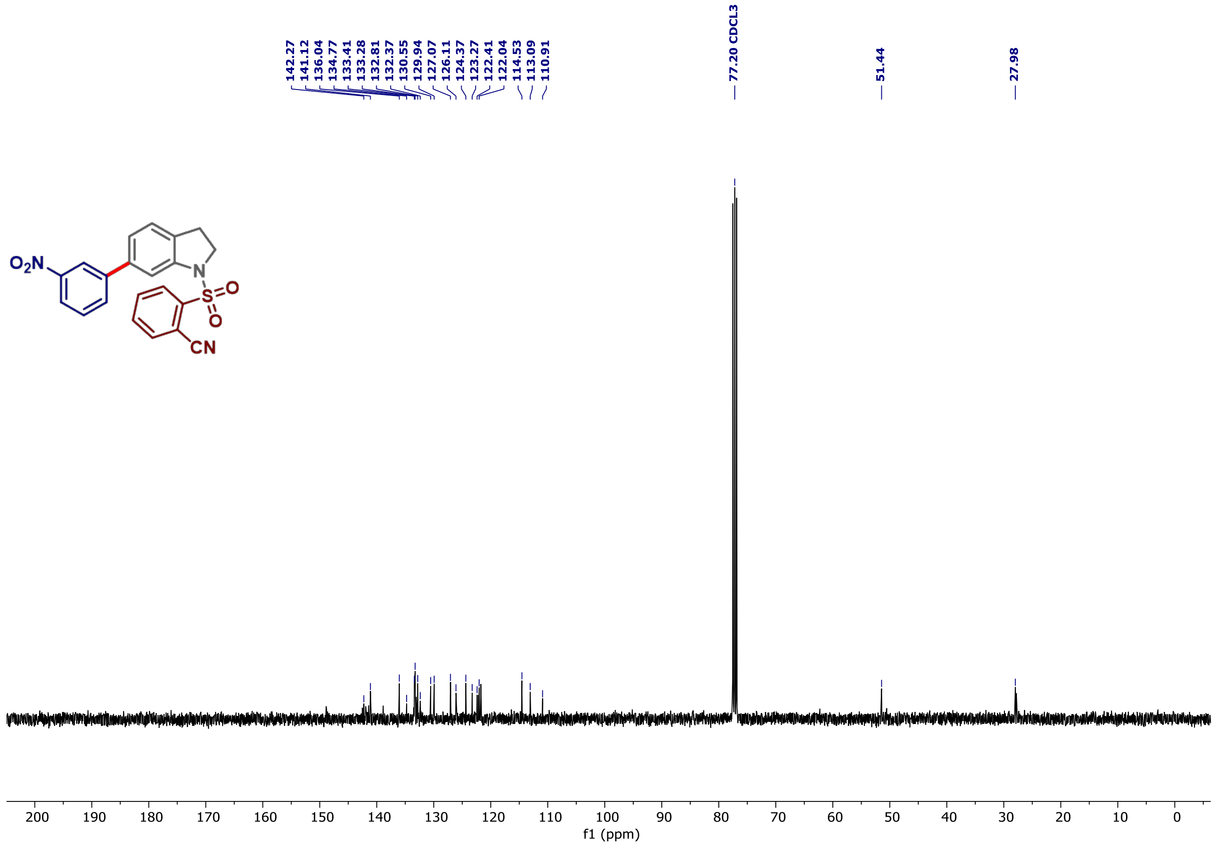

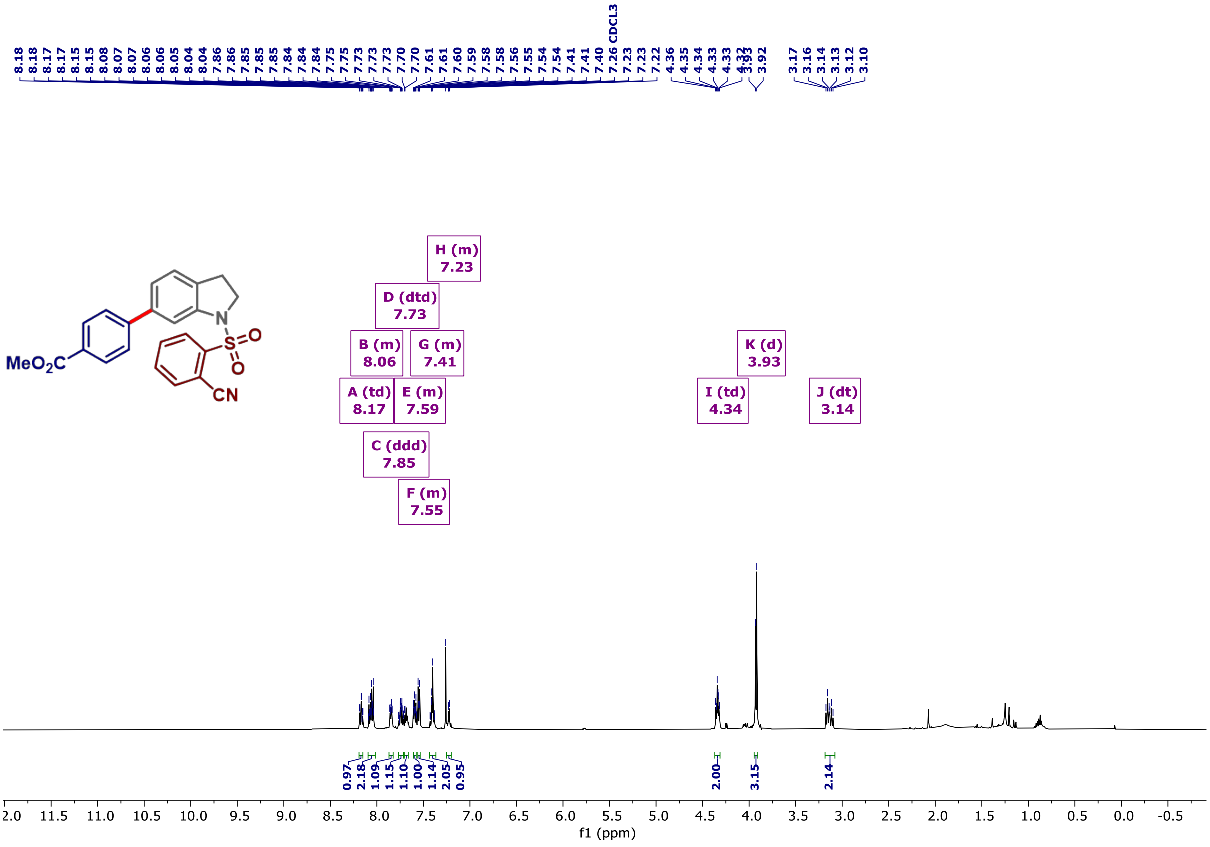
8.18 8.18 8.17 8.15 8.15 8.08 8.07 8.07 8.06 8.06 8.05 8.04 8.04 7.86 7.86 7.85 7.85 7.85 7.84 7.84 7.84 7.75 7.75 7.73 7.73 7.73 7.70 7.70 7.61 7.61 7.60 7.59 7.58 7.58 7.56 7.55 7.54 7.54 7.41 7.41 7.40 7.26 CDCL3 7.23 7.23 7.22 4.36 4.35 4.34 4.33 4.33 4.32 3.93 3.92 3.17 3.16 3.14 3.13 3.12 3.10
MeO2C
N
S
O
O
CN
H (m) 7.23
D (dtd) 7.73
B (m) 8.06
G (m) 7.41
K (d) 3.93
A (td) 8.17
E (m) 7.59
I (td) 4.34
J (dt) 3.14
C (ddd) 7.85
F (m) 7.55
0.97 2.18 1.09 1.15 1.10 1.00 1.14 2.05 0.95 2.00 3.15 2.14
f1 (ppm)

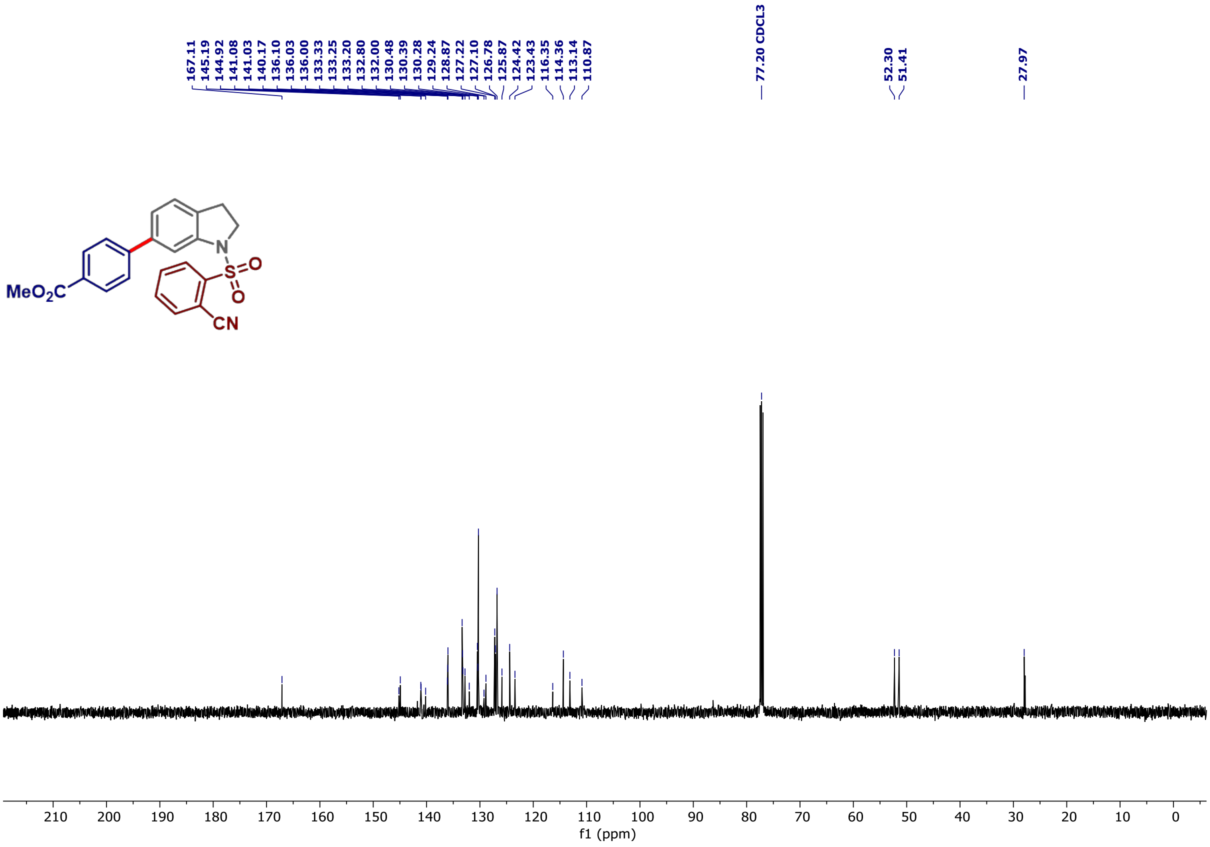
167.11 145.19 144.92 141.08 141.03 140.17 136.10 136.03 136.00 133.33 133.25 133.20 132.80 132.00 130.48 130.39 130.28 129.24 128.87 127.22 127.10 126.78 125.87 124.42 123.43 116.35 114.36 113.14 110.87
77.20 CDCL3
52.30 51.41
27.97
MeO2C
N
S
O
O
CN
f1 (ppm)

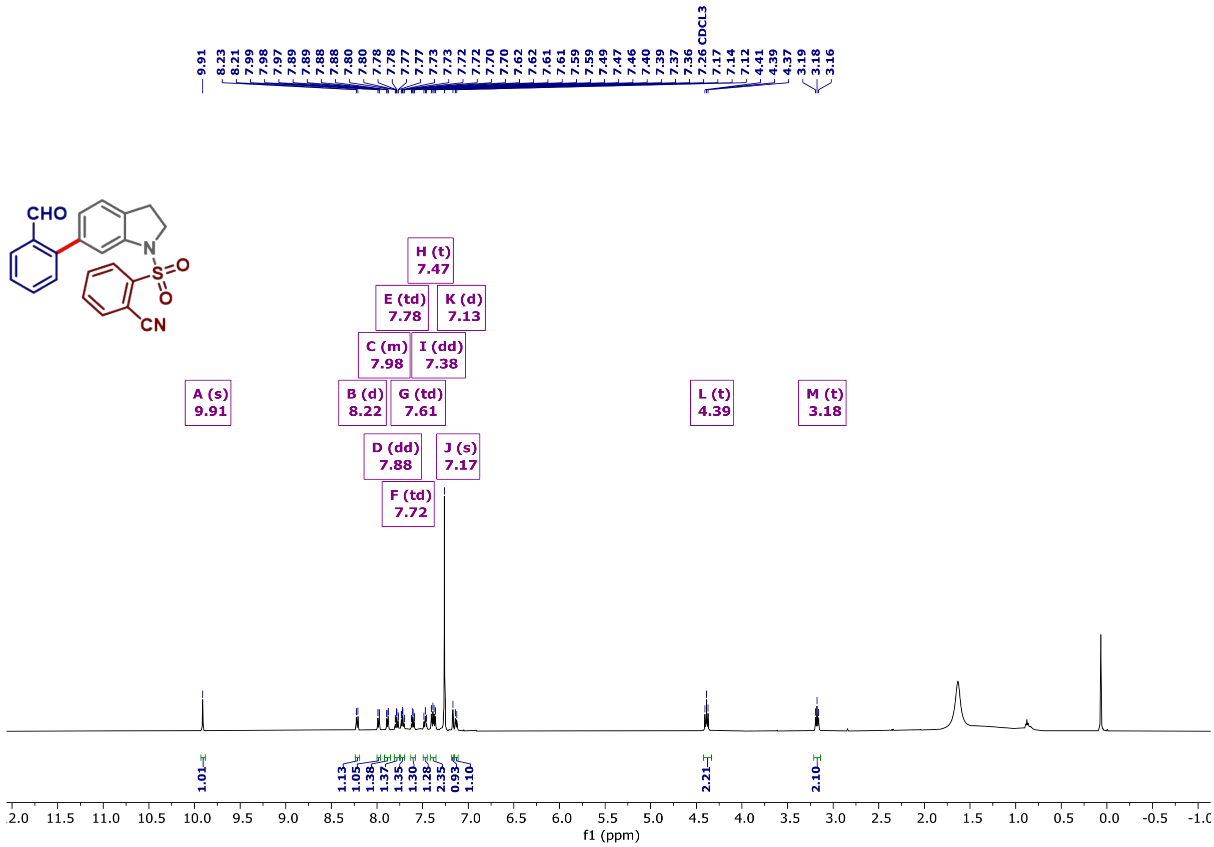

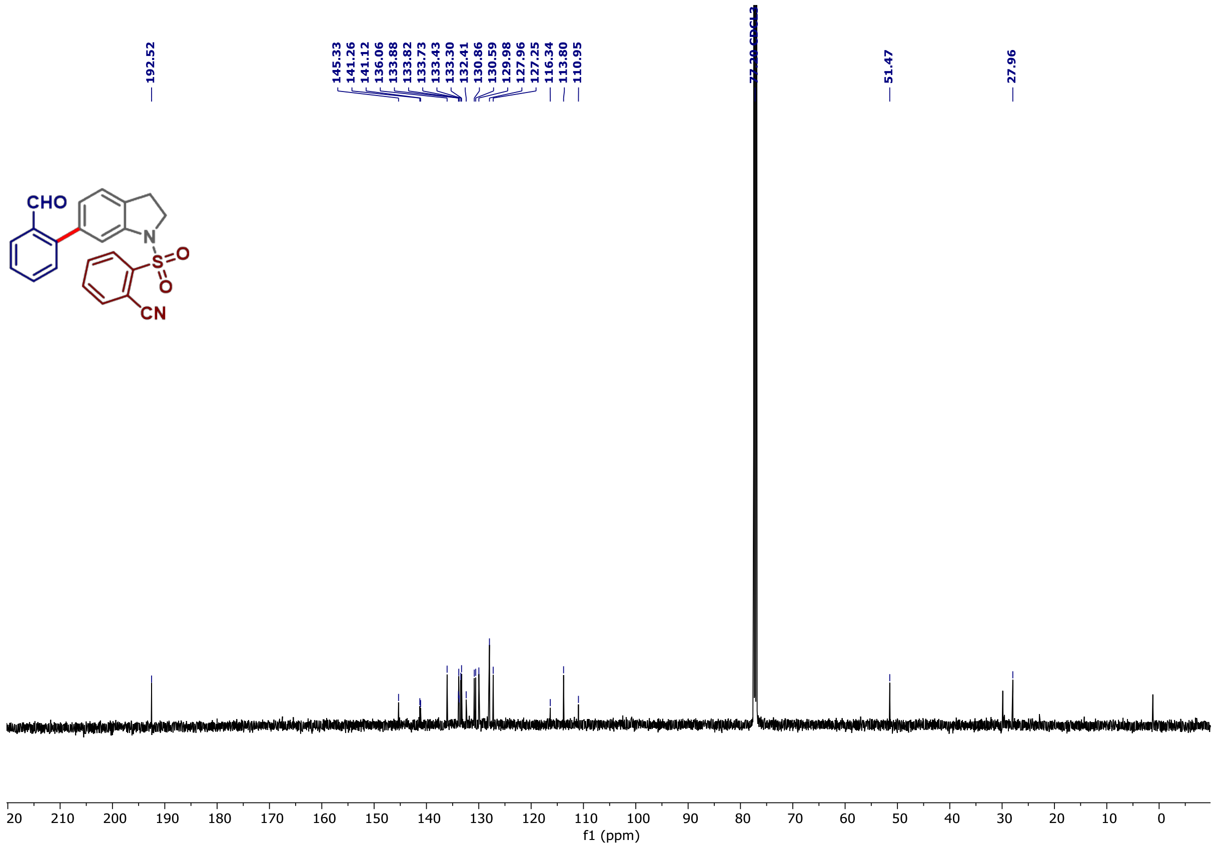

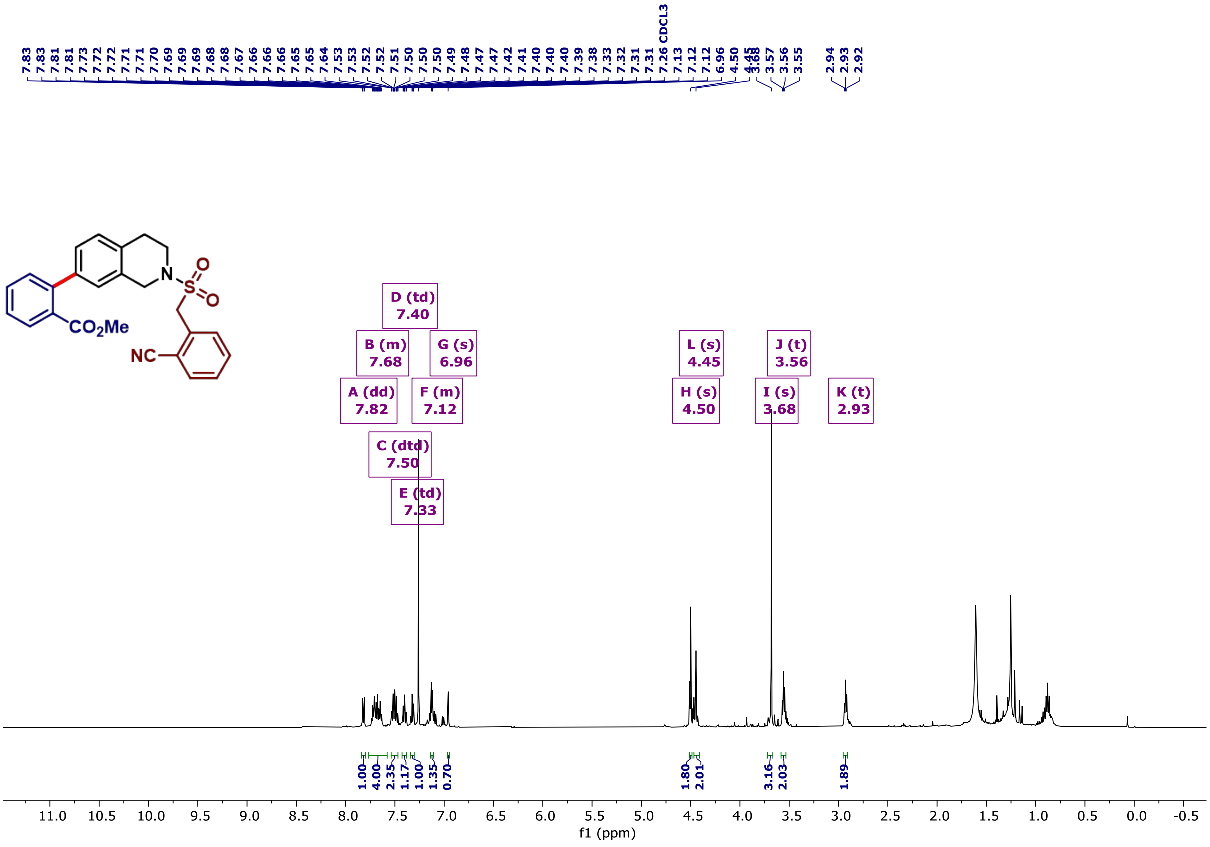
7.83
7.83
7.81
7.73
7.72
7.72
7.71
7.71
7.70
7.69
7.69
7.69
7.68
7.68
7.67
7.66
7.66
7.65
7.65
7.64
7.53
7.53
7.52
7.52
7.51
7.50
7.50
7.50
7.49
7.48
7.47
7.47
7.42
7.41
7.40
7.40
7.40
7.39
7.38
7.33
7.32
7.31
7.31
7.26 CDCL3
7.13
7.12
7.12
6.96
4.50
3.68
3.57
3.56
3.55
2.94
2.93
2.92
CO2Me
NC
N
S
O
O
D (td) 7.40
B (m) 7.68
G (s) 6.96
A (dd) 7.82
F (m) 7.12
C (dtd) 7.50
E (td) 7.33
L (s) 4.45
H (s) 4.50
J (t) 3.56
I (s) 3.68
K (t) 2.93
1.00
4.00
2.35
1.17
1.00
1.35
0.70
1.80
2.01
3.16
2.03
1.89
11.0
10.5
10.0
9.5
9.0
8.5
8.0
7.5
7.0
6.5
6.0
5.5
5.0
4.5
4.0
3.5
3.0
2.5
2.0
1.5
1.0
0.5
0.0
-0.5
f1 (ppm)

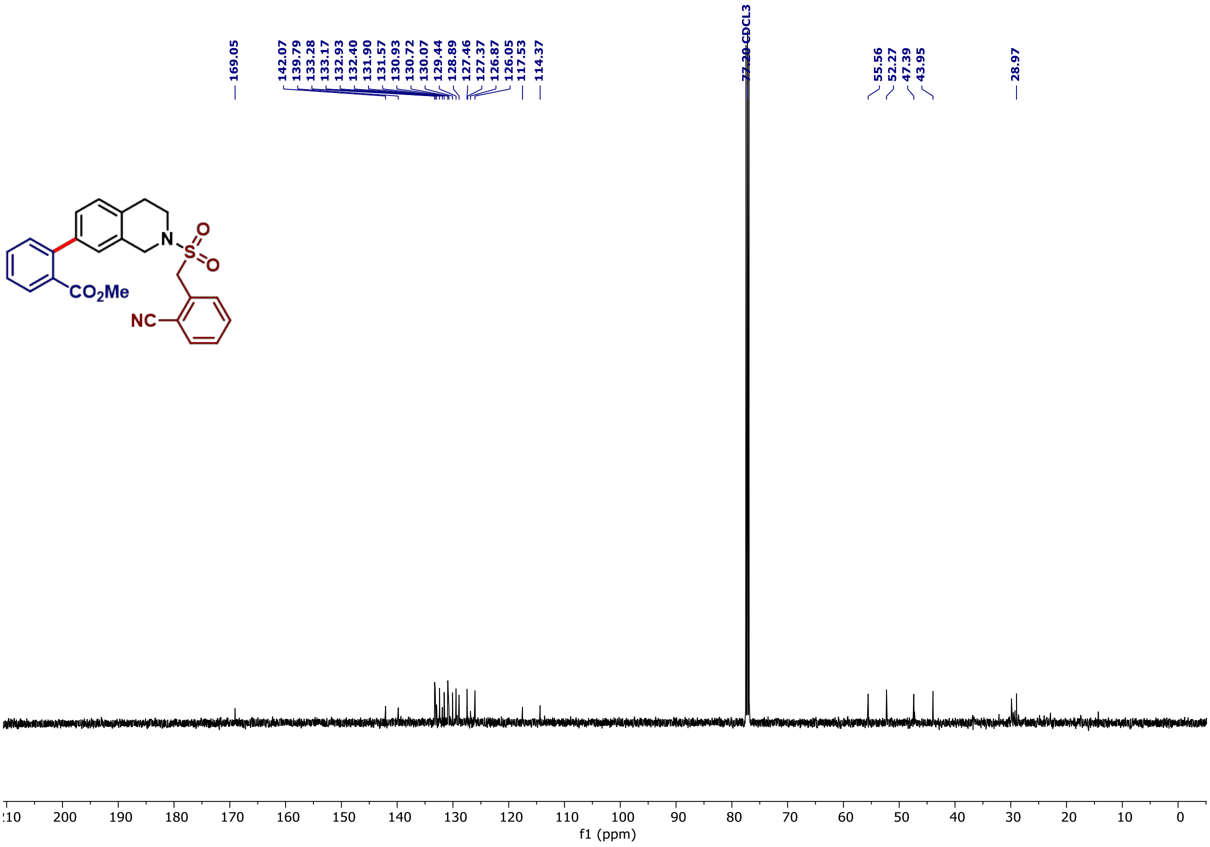
169.05
142.07
139.79
133.28
133.17
132.93
132.40
131.90
131.57
130.93
130.72
130.07
129.44
128.89
127.46
127.37
126.87
126.05
117.53
114.37
77.20 CDCL3
55.56
52.27
47.39
43.95
28.97
CO2Me
NC
N
S
O
O
200
190
180
170
160
150
140
130
120
110
100
90
80
70
60
50
40
30
20
10
0
f1 (ppm)

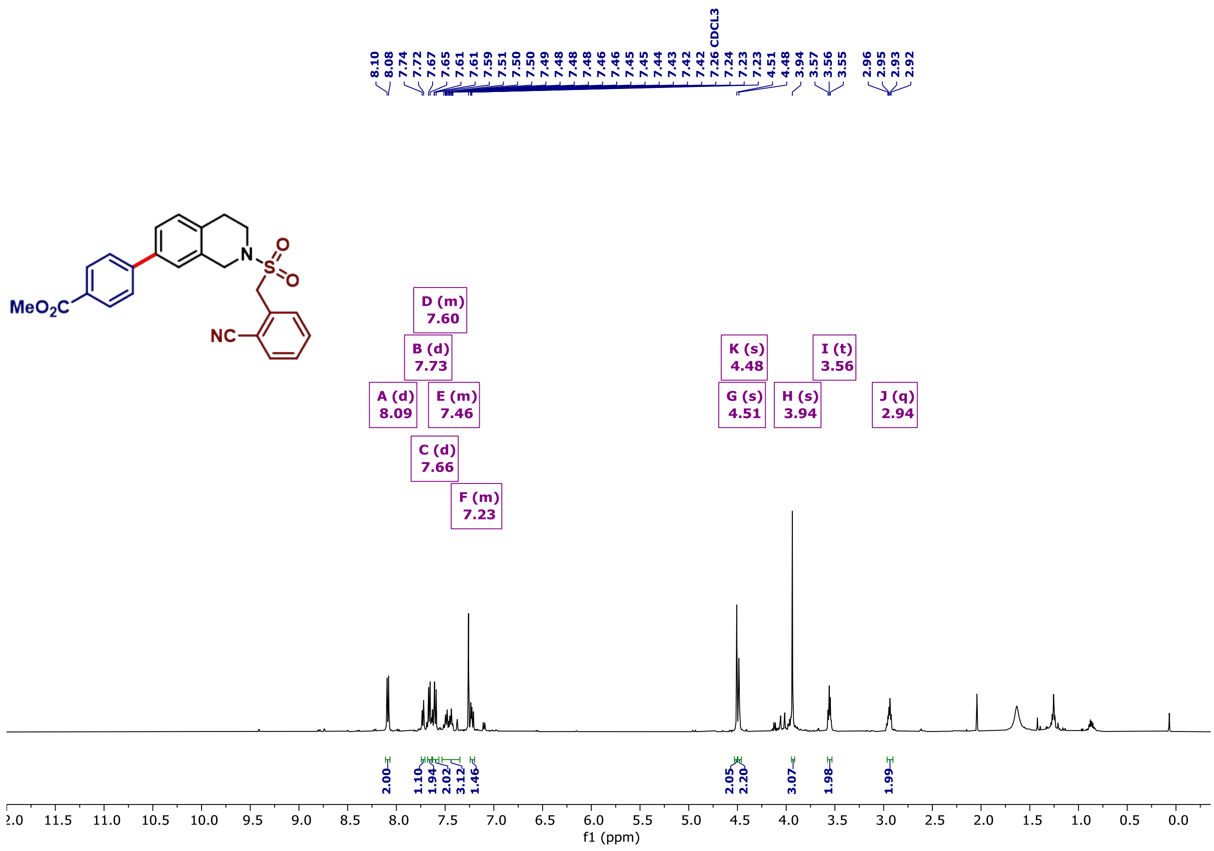

8.10
8.08
7.74
7.72
7.67
7.65
7.61
7.61
7.59
7.51
7.50
7.50
7.49
7.48
7.48
7.46
7.46
7.45
7.45
7.44
7.43
7.42
7.42
7.26 CDCL3
7.24
7.23
7.23
4.51
4.48
3.94
3.57
3.56
3.55
2.96
2.95
2.93
2.92
MeO2C
NC
N
S
O
O
D (m) 7.60
B (d) 7.73
A (d) 8.09
E (m) 7.46
C (d) 7.66
F (m) 7.23
K (s) 4.48
G (s) 4.51
H (s) 3.94
I (t) 3.56
J (q) 2.94
2.00
1.10
1.94
2.02
3.12
1.46
2.05
2.20
3.07
1.98
1.99
f1 (ppm)

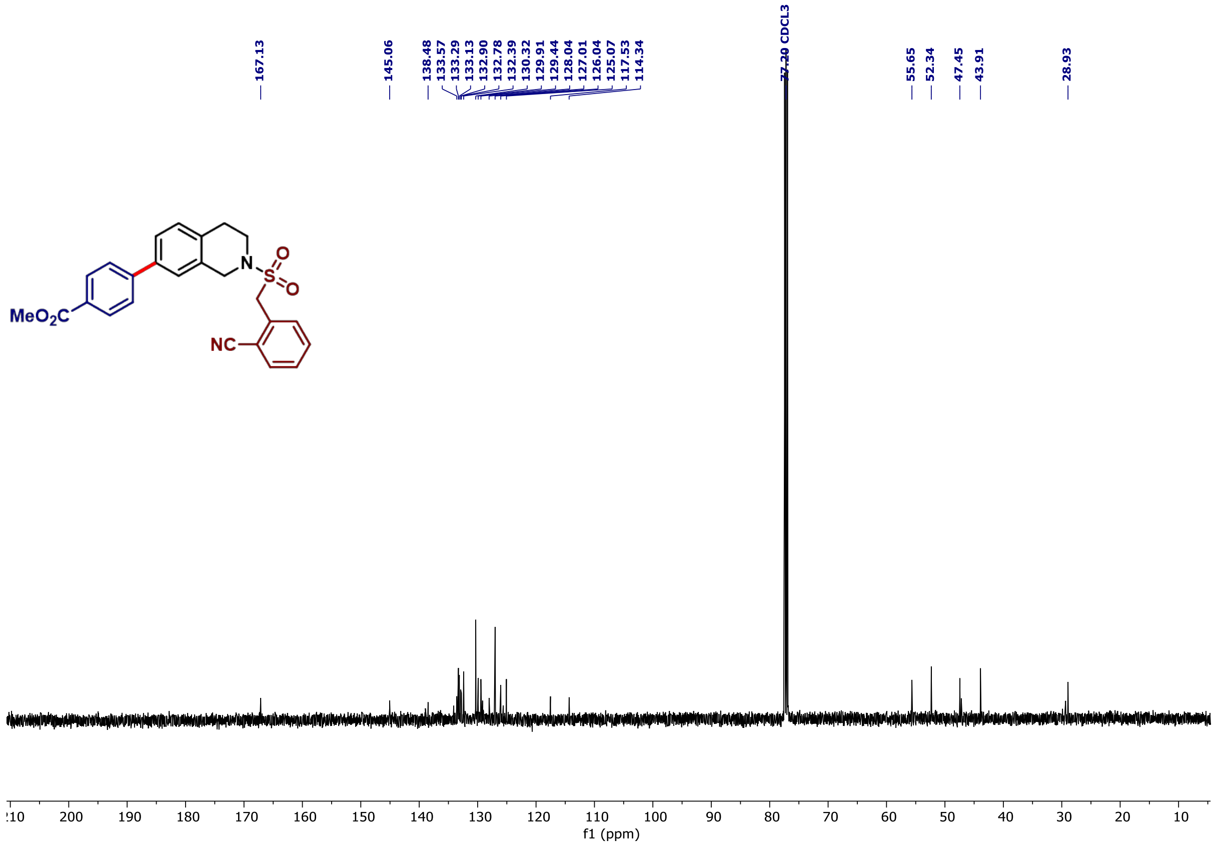

167.13
145.06
138.48
133.57
133.29
133.13
132.90
132.78
132.39
130.32
129.91
129.44
128.04
127.01
126.04
125.07
117.53
114.34
77.20 CDCL3
55.65
52.34
47.45
43.91
28.93
MeO2C
NC
N
S
O
O
f1 (ppm)

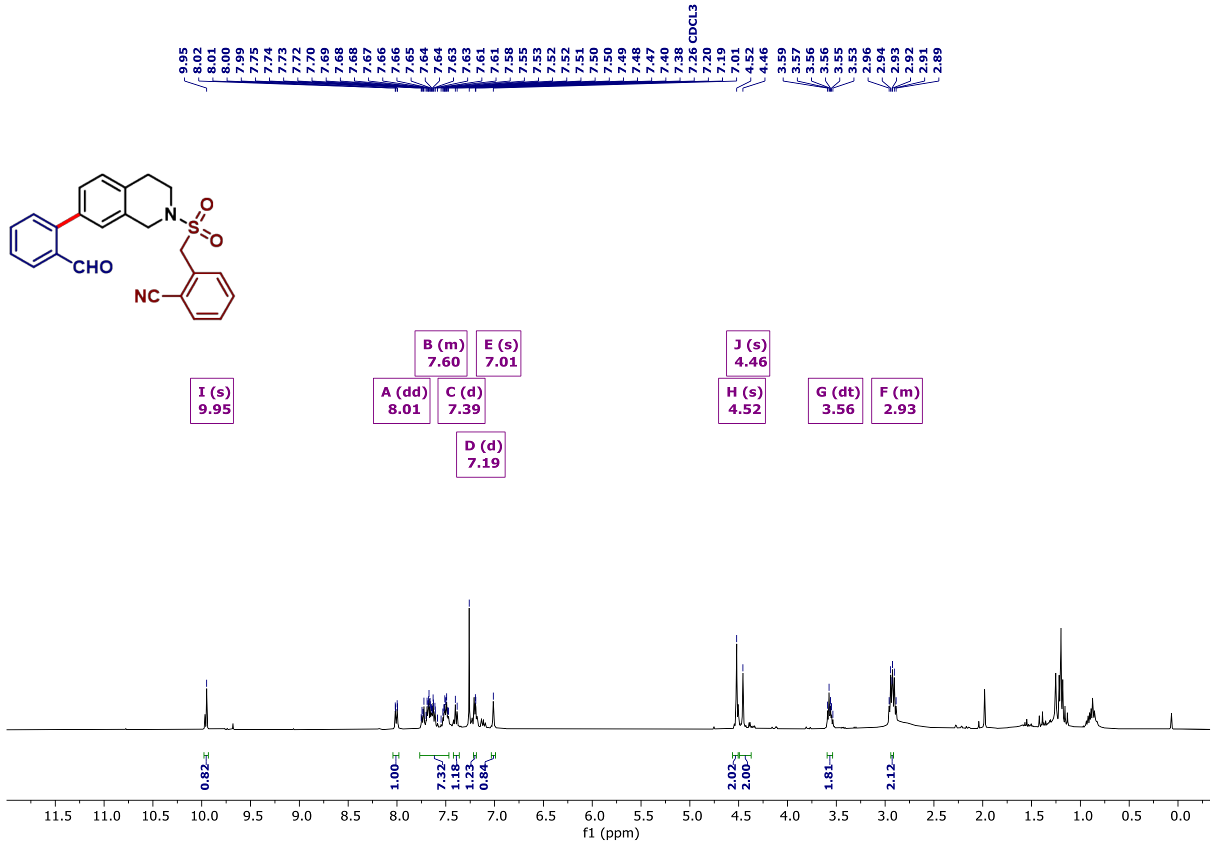
9.95 8.02 8.01 8.00 7.99 7.75 7.74 7.73 7.72 7.70 7.69 7.68 7.68 7.67 7.66 7.66 7.65 7.64 7.64 7.63 7.63 7.61 7.61 7.58 7.55 7.53 7.52 7.51 7.50 7.50 7.49 7.48 7.47 7.40 7.38 7.26 CDCL3 7.20 7.19 7.01 4.52 4.46 3.59 3.57 3.56 3.56 3.55 3.53 2.96 2.94 2.93 2.92 2.91 2.89
CHO
NC
N
S
O
O
B (m) 7.60
E (s) 7.01
J (s) 4.46
I (s) 9.95
A (dd) 8.01
C (d) 7.39
H (s) 4.52
G (dt) 3.56
F (m) 2.93
D (d) 7.19
0.82
1.00
7.32
1.18
1.23
0.84
2.02
2.00
1.81
2.12
11.5 11.0 10.5 10.0 9.5 9.0 8.5 8.0 7.5 7.0 6.5 6.0 5.5 5.0 4.5 4.0 3.5 3.0 2.5 2.0 1.5 1.0 0.5 0.0
f1 (ppm)

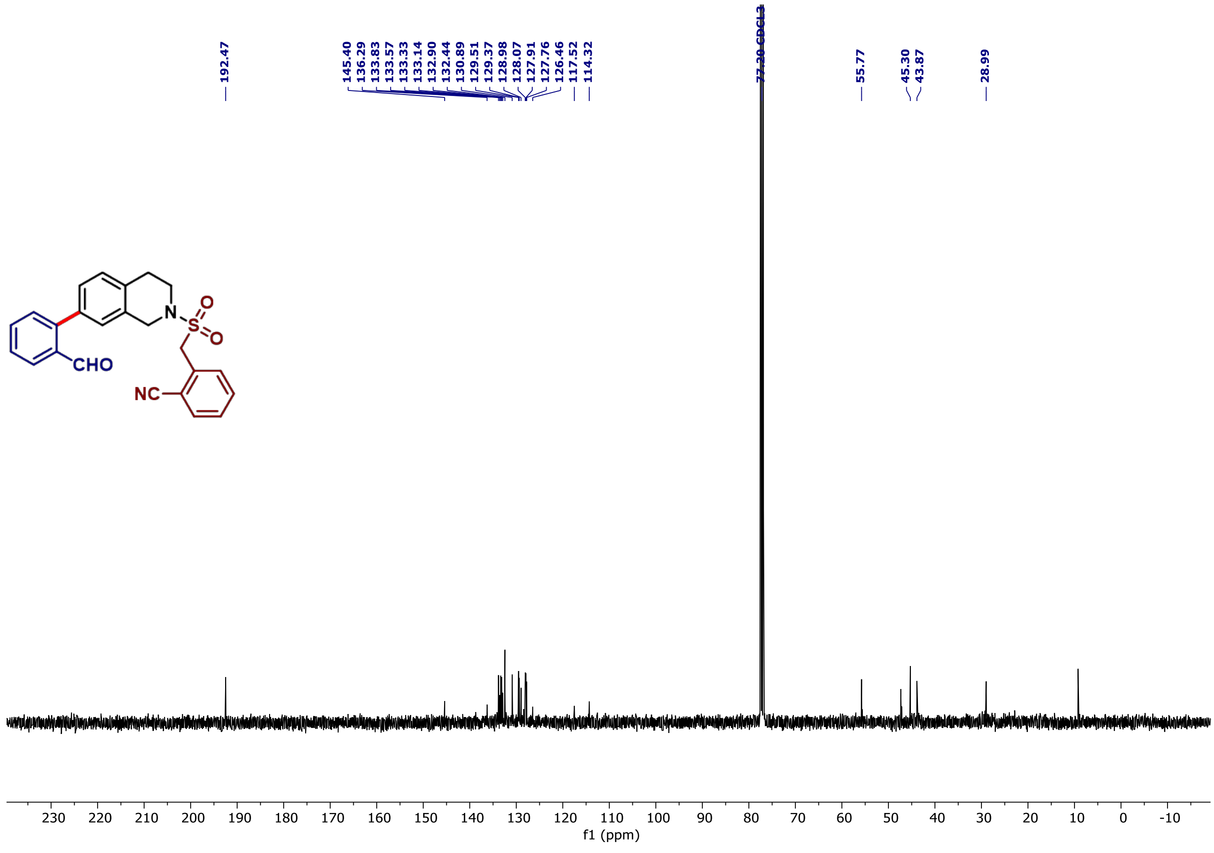
192.47
145.40 136.29 133.83 133.57 133.33 133.14 132.90 132.44 130.89 129.51 129.37 128.98 128.07 127.91 127.76 126.46 117.52 114.32
77.20 CDCL3
55.77
45.30 43.87
28.99
CHO
NC
N
S
O
O
230 220 210 200 190 180 170 160 150 140 130 120 110 100 90 80 70 60 50 40 30 20 10 0 -10
f1 (ppm)

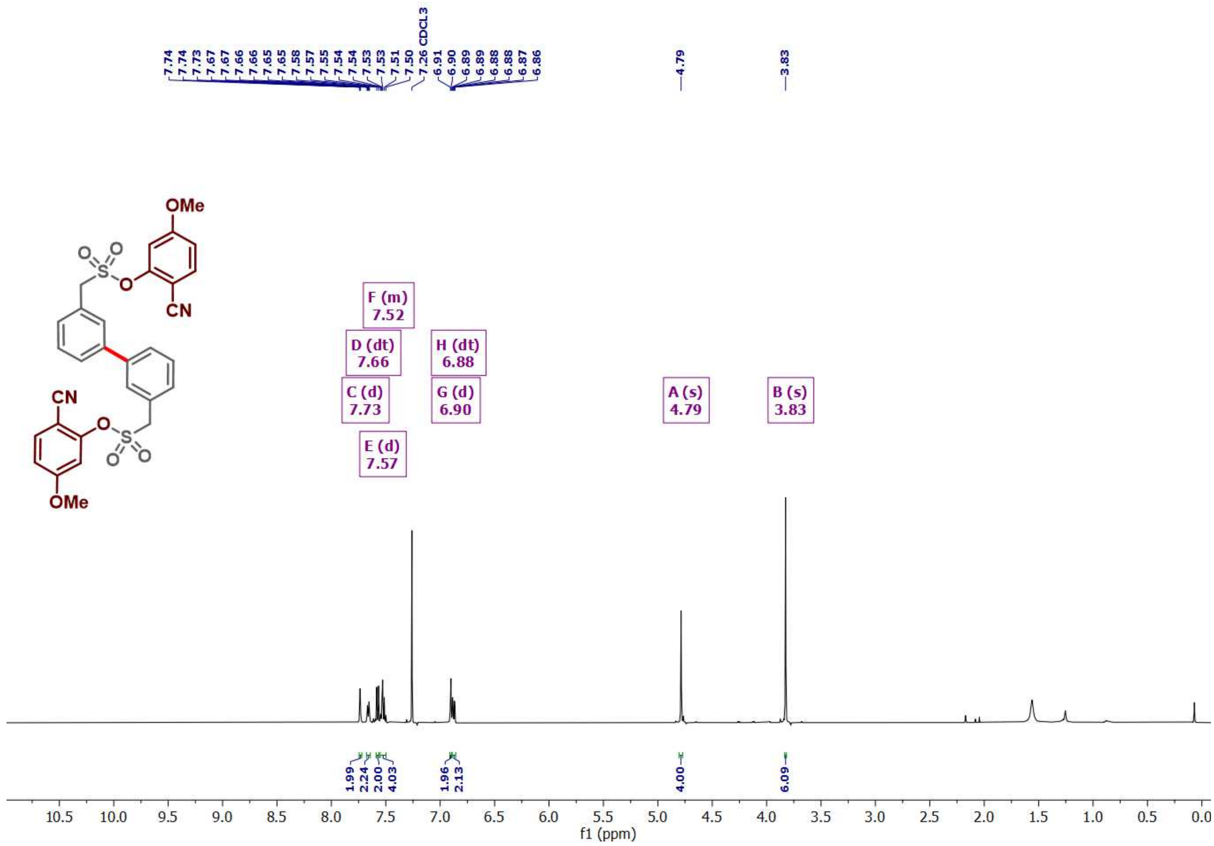
7.74
7.74
7.73
7.67
7.67
7.66
7.66
7.65
7.65
7.58
7.57
7.55
7.54
7.54
7.53
7.53
7.51
7.50
7.26 CDCL3
6.91
6.90
6.89
6.89
6.88
6.88
6.87
6.86
4.79
3.83
OMe
CN
CN
OMe
F (m) 7.52
D (dt) 7.66
H (dt) 6.88
C (d) 7.73
G (d) 6.90
A (s) 4.79
B (s) 3.83
E (d) 7.57
1.99
2.24
2.00
4.03
1.96
2.13
4.00
6.09
f1 (ppm)

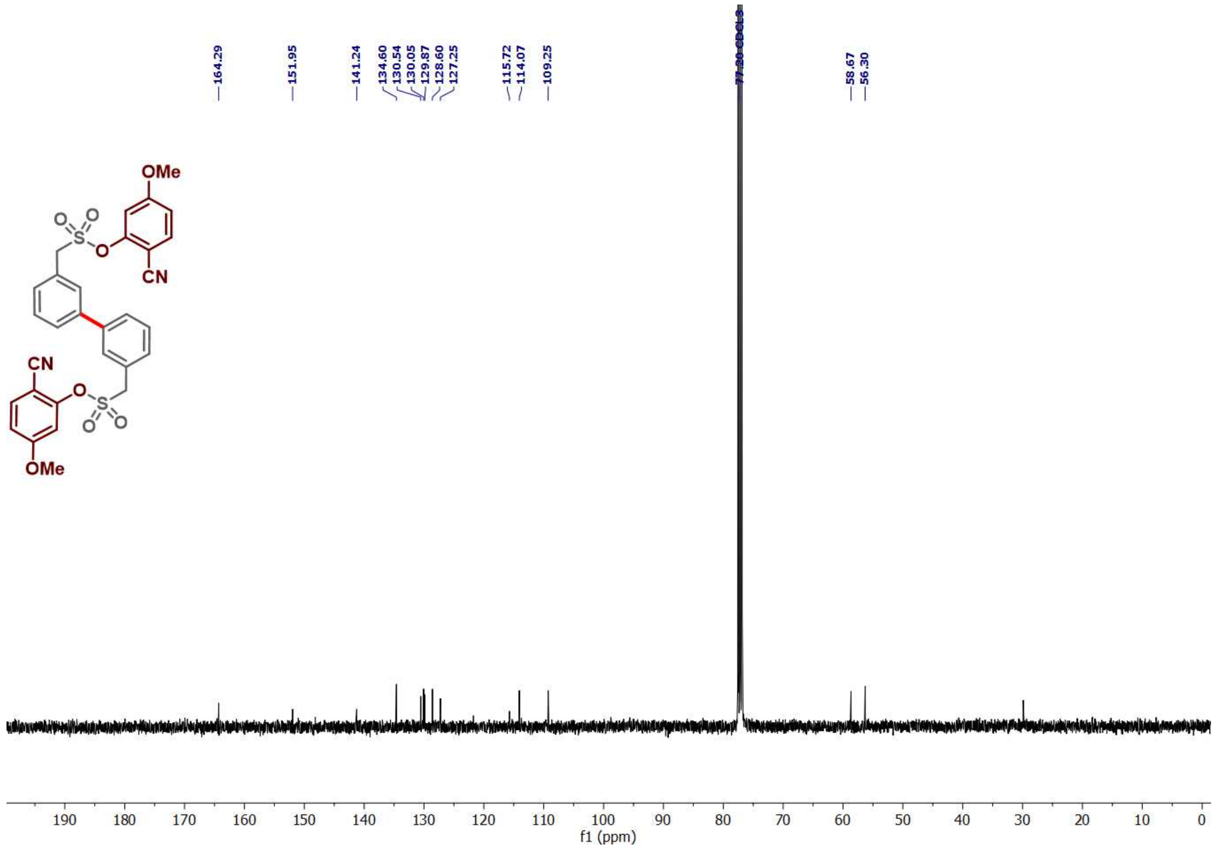
164.29
151.95
141.24
134.60
130.54
130.05
129.87
128.60
127.25
115.72
114.07
109.25
77.00 CDCL3
58.67
56.30
OMe
CN
CN
OMe
f1 (ppm)

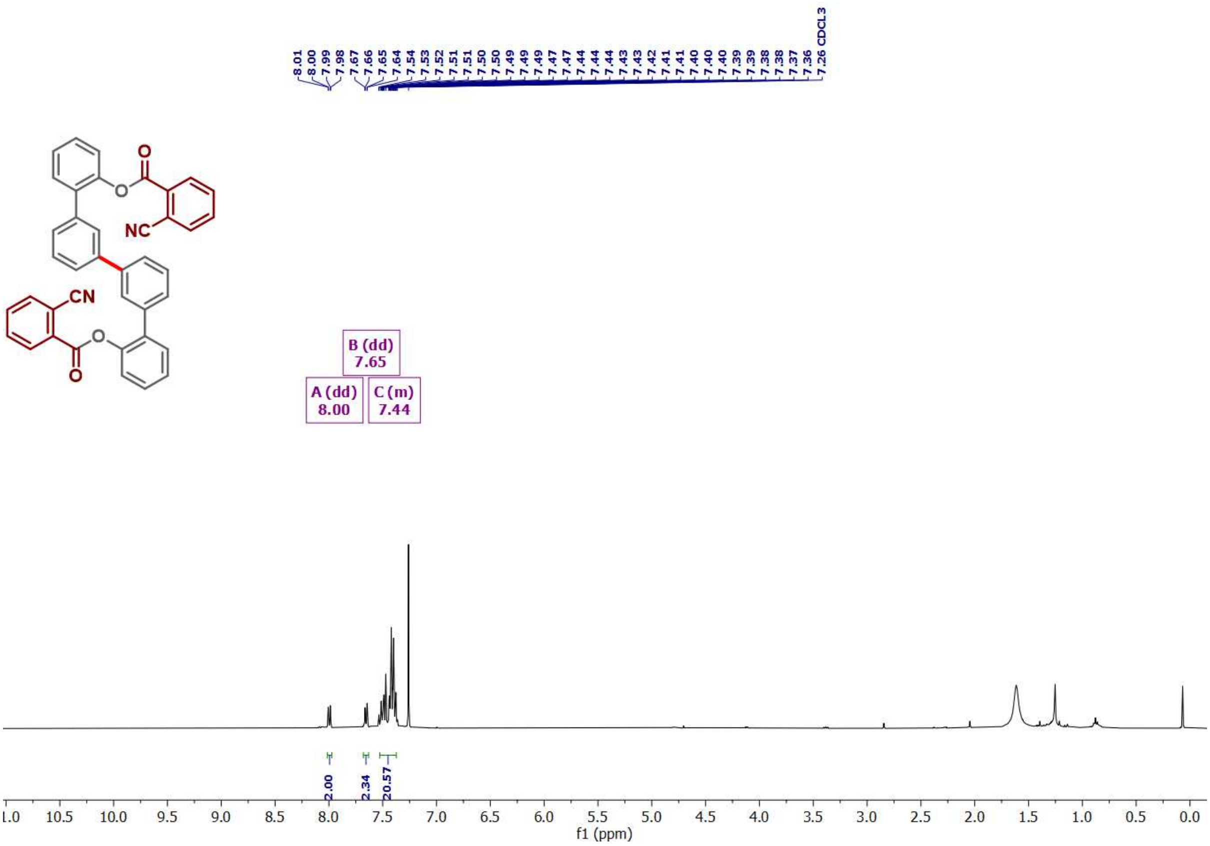
8.01
8.00
7.99
7.98
7.67
7.66
7.65
7.64
7.54
7.53
7.52
7.51
7.51
7.50
7.49
7.49
7.49
7.47
7.44
7.44
7.44
7.43
7.42
7.41
7.41
7.40
7.40
7.40
7.39
7.38
7.38
7.37
7.36
7.26 CDCL3
O
NC
CN
O
O
O
B (dd)
7.65
A (dd)
8.00
C (m)
7.44
2.00
2.34
20.57
f1 (ppm)

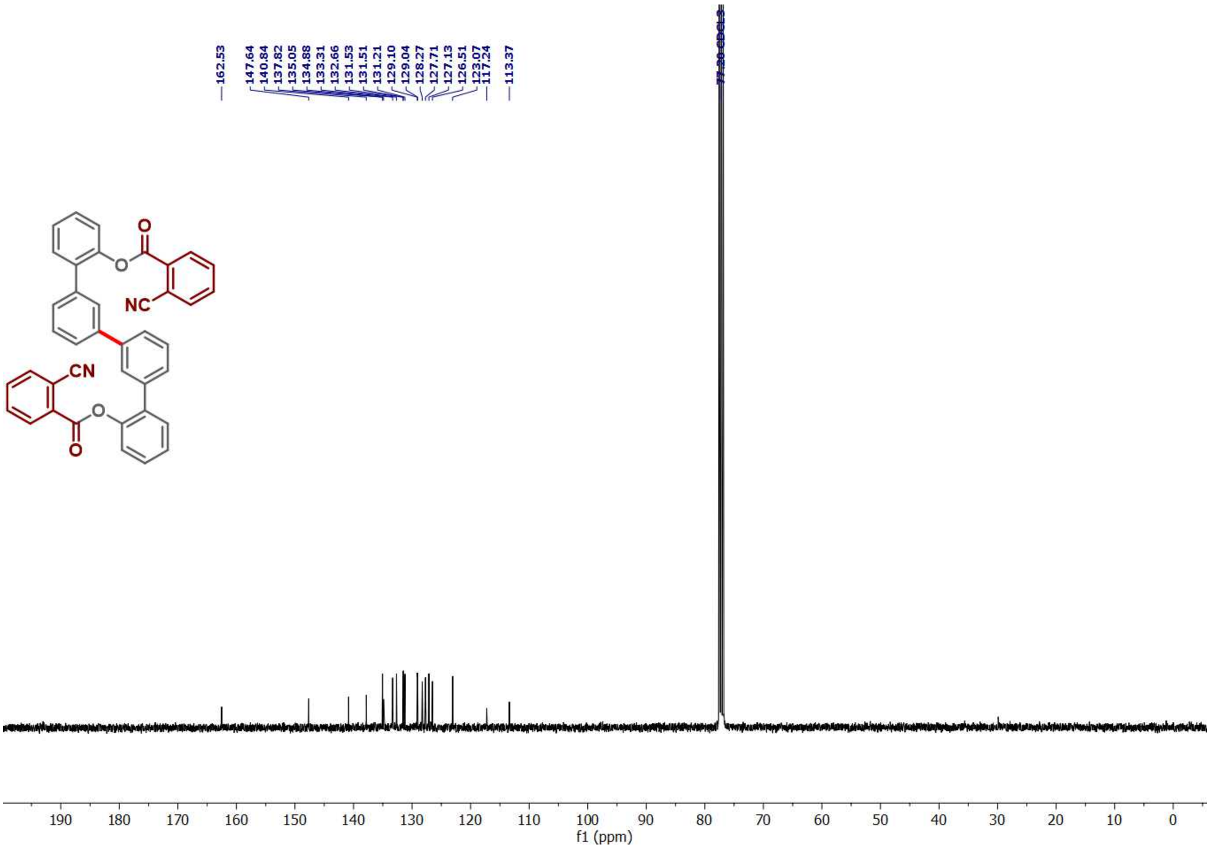
162.53
147.64
140.84
137.82
135.05
134.88
133.31
132.66
131.53
131.51
131.21
129.10
129.04
128.27
127.71
127.13
126.51
123.07
117.24
113.37
77.00 CDCL3
O
NC
CN
O
O
O
f1 (ppm)
