# Supplementary material for: A Generalized Approach for Distal C–H Arylation of Organic Building Blocks: Unveiling the Role of Counter Anion
Source: Adv Sci (Weinh). 2025 Dec 25;13(10):e19731. doi: 10.1002/advs.202519731 (PMC12915134; doi:10.1002/advs.202519731)

checkCIF/PLATON report

Structure factors have been supplied for datablock(s) DM_JG_764_RRR_autored

THIS REPORT IS FOR GUIDANCE ONLY. IF USED AS PART OF A REVIEW PROCEDURE FOR PUBLICATION, IT SHOULD NOT REPLACE THE EXPERTISE OF AN EXPERIENCED CRYSTALLOGRAPHIC REFEREE.

No syntax errors found. CIF dictionary Interpreting this report

Datablock: DM_JG_764_RRR_autored

Bond precision:	C-C = 0.0090 A		Wavelength=0.71073
Cell:	a=7.6531(15)	b=16.702(2)	c=15.820(2)
	alpha=90	beta=90.802(14)	gamma=90
Temperature:	150 K		

	Calculated	Reported
Volume	2022.0(5)	2022.0(5)
Space group	P 21/c	P 1 21/c 1
Hall group	-P 2ybc	-P 2ybc
Moiety formula	C23 H19 N O6 S	C23 H19 N O6 S
Sum formula	C23 H19 N O6 S	C23 H19 N O6 S
Mr	437.45	437.48
Dx,g cm-3	1.437	1.437
Z	4	4
Mu (mm-1)	0.202	0.202
F000	912.0	913.0
F000’	912.97	
h,k,lmax	9,19,18	9,19,18
Nref	3554	3513
Tmin,Tmax	0.977,0.981	0.220,1.000
Tmin’	0.977	

Correction method= # Reported T Limits: Tmin=0.220 Tmax=1.000
AbsCorr = MULTI-SCAN

Data completeness= 0.988 Theta(max)= 25.000

R(reflections)= 0.1474(2618) wR2(reflections)= 0.3963(3513)

S = 1.483 Npar= 282

The following ALERTS were generated. Each ALERT has the format
test-name_ALERT_alert-type_alert-level.
Click on the hyperlinks for more details of the test.

Alert level B

```
PLAT084_ALERT_3_B High wR2 Value (i.e. > 0.25) ...................       0.40 Report
```

Alert level C

```
DIFMX02_ALERT_1_C  The maximum difference density is > 0.1*ZMAX*0.75
            The relevant atom site should be identified.
RINTA01_ALERT_3_C  The value of Rint is greater than 0.12
            Rint given   0.168
PLAT020_ALERT_3_C The Value of Rint is Greater Than 0.12 .........      0.168 Report
PLAT082_ALERT_2_C High R1 Value ..................................       0.15 Report
PLAT097_ALERT_2_C Large Reported Max.  (Positive) Residual Density       1.22 eA-3
PLAT340_ALERT_3_C Low Bond Precision on  C-C Bonds ...............    0.00895 Ang.
PLAT906_ALERT_3_C Large K Value in the Analysis of Variance ......     21.214 Check
PLAT906_ALERT_3_C Large K Value in the Analysis of Variance ......      3.330 Check
PLAT911_ALERT_3_C Missing FCF Refl Between Thmin & STh/L=    0.595         39 Report
                0  2  1,  -1  7  1,   1 11  1,   1  6  2,   1  7  2,   1 10  2,
                1  3  3,  -1  7  3,   1  0  4,   1  2  4,   1  3  4,   1  8  4,
                1  7  5,   1  8  5,  -1 11  5,   1  0  6,  -1  3  6,  -1 11  6,
                1  1  7,  -1  3  7,  -1  3  8,   1 11  8,  -1  3  9,   1  4 10,
               -1  2 11,  -1  4 11,   1  9 11,  -2  5 12,   1 10 12,  -1  2 13,
               -6  3 13,  -1  4 13,   1  5 13,  -1  0 14,  -1  1 14,  -1  2 14,
               -1  3 14,  -5  4 14,  -1  1 15,
PLAT977_ALERT_2_C Check Negative Difference Density on H00E      .      -0.37 eA-3
PLAT977_ALERT_2_C Check Negative Difference Density on H00R      .      -0.43 eA-3
```

Alert level G

```
PLAT012_ALERT_1_G No       _shelx_res_checksum Found in CIF ......     Please Check
PLAT072_ALERT_2_G SHELXL First  Parameter in WGHT  Unusually Large       0.20 Report
PLAT073_ALERT_1_G H-atoms ref., but hydrogen treatment Reported as     constr Check
PLAT720_ALERT_4_G Number of Unusual/Non-Standard Labels ..........         49 Note
              O002    O003    O004    O005    O006    O007    N008    C00A
              C00B    H00B    C00C    C00D    H00D    C00E    H00E    C00F
              C00G    C00H    H00H    C00I    C00J    C00K    H00K    C00L
              C00M    H00M    C00N    H00A    H00C    C00O    C00P    H00P
              C00Q    H00Q    C00R    H00R    C00S    H00S    C00T    H00F
              H00G    H00I    C00U    H00U    C00V    H00J    H00L    H00N
              C009
PLAT769_ALERT_4_G CIF Embedded Explicitly Supplied Scattering Data     Please Note
PLAT909_ALERT_3_G Percentage of I>2sig(I) Data at Theta(Max) Still        51% Note
PLAT910_ALERT_3_G Missing # of FCF Reflection(s) Below Theta(Min).          1 Note
                0  1  1,
PLAT930_ALERT_2_G FCF-based   Twin Law ( 0 0 1)         Est.d BASF       0.29 Check
PLAT931_ALERT_5_G CIFcalcFCF  Twin Law ( 0 0 1)         Est.d BASF       0.29 Check
PLAT933_ALERT_2_G Number of HKL-OMIT Records in Embedded .res File         40 Note
                1  2  4,  -1  2 13,   1  7  2,  -1  1 14,  -1  2 14,  -1  2 11,
                0  1  1,  -1  3  8,  -1  3  7,   1  0  4,   1 10  2,  -1 11  6,
                1 11  1,   1 10 12,  -2  5 12,  -1  1 15,   1  3  3,  -1  0 14,
                1  5 13,  -1  7  3,  -1  3  6,   1  0  6,  -1  3  9,   1  4 10,
```

```
              -1   7  1,    1 11  8,    9  9 13,    1  3  4,  -1  4 11,  -1  3 14,
              -6   3 13,    1  6  2,  -1 11  5,    1  7  5,    1  1  7,  -1  4 13,
               1   9 11,    1  8  5,    1  8  4,  -6 12 10,
PLAT960_ALERT_3_G Number of Intensities with I < - 2*Sigma(I) ....          3 Check
PLAT967_ALERT_5_G Note: Two-Theta Cutoff Value in Embedded .res ..       50.0 Degree
PLAT969_ALERT_5_G The 'Henn et al.' R-Factor-gap value ..........      4.883 Note
              Predicted wR2: Based on SigI**2  8.11 or SHELX Weight 26.71
PLAT978_ALERT_2_G Number C-C Bonds with Positive Residual Density.          0 Info
PLAT983_ALERT_1_G The  S-f"=    0.1244 Deviates from  IT-Value =       0.1234 Check
```

```
   0 ALERT level A = Most likely a serious problem - resolve or explain
   1 ALERT level B = A potentially serious problem, consider carefully
  11 ALERT level C = Check. Ensure it is not caused by an omission or oversight
  15 ALERT level G = General information/check it is not something unexpected

   4 ALERT type 1 CIF construction/syntax error, inconsistent or missing data
   8 ALERT type 2 Indicator that the structure model may be wrong or deficient
  10 ALERT type 3 Indicator that the structure quality may be low
   2 ALERT type 4 Improvement, methodology, query or suggestion
   3 ALERT type 5 Informative message, check
```

It is advisable to attempt to resolve as many as possible of the alerts in all categories. Often the minor alerts point to easily fixed oversights, errors and omissions in your CIF or refinement strategy, so attention to these fine details can be worthwhile. In order to resolve some of the more serious problems it may be necessary to carry out additional measurements or structure refinements. However, the purpose of your study may justify the reported deviations and the more serious of these should normally be commented upon in the discussion or experimental section of a paper or in the "special_details" fields of the CIF. checkCIF was carefully designed to identify outliers and unusual parameters, but every test has its limitations and alerts that are not important in a particular case may appear. Conversely, the absence of alerts does not guarantee there are no aspects of the results needing attention. It is up to the individual to critically assess their own results and, if necessary, seek expert advice.

Publication of your CIF in IUCr journals

A basic structural check has been run on your CIF. These basic checks will be run on all CIFs submitted for publication in IUCr journals (*Acta Crystallographica*, *Journal of Applied Crystallography*, *Journal of Synchrotron Radiation*); however, if you intend to submit to *Acta Crystallographica Section C* or *E* or *IUCrData*, you should make sure that full publication checks are run on the final version of your CIF prior to submission.

Publication of your CIF in other journals

Please refer to the *Notes for Authors* of the relevant journal for any special instructions relating to CIF submission.

PLATON version of 11/11/2024; check.def file version of 11/11/2024

Datablock DM_JG_764_RRR_autored - ellipsoid plot

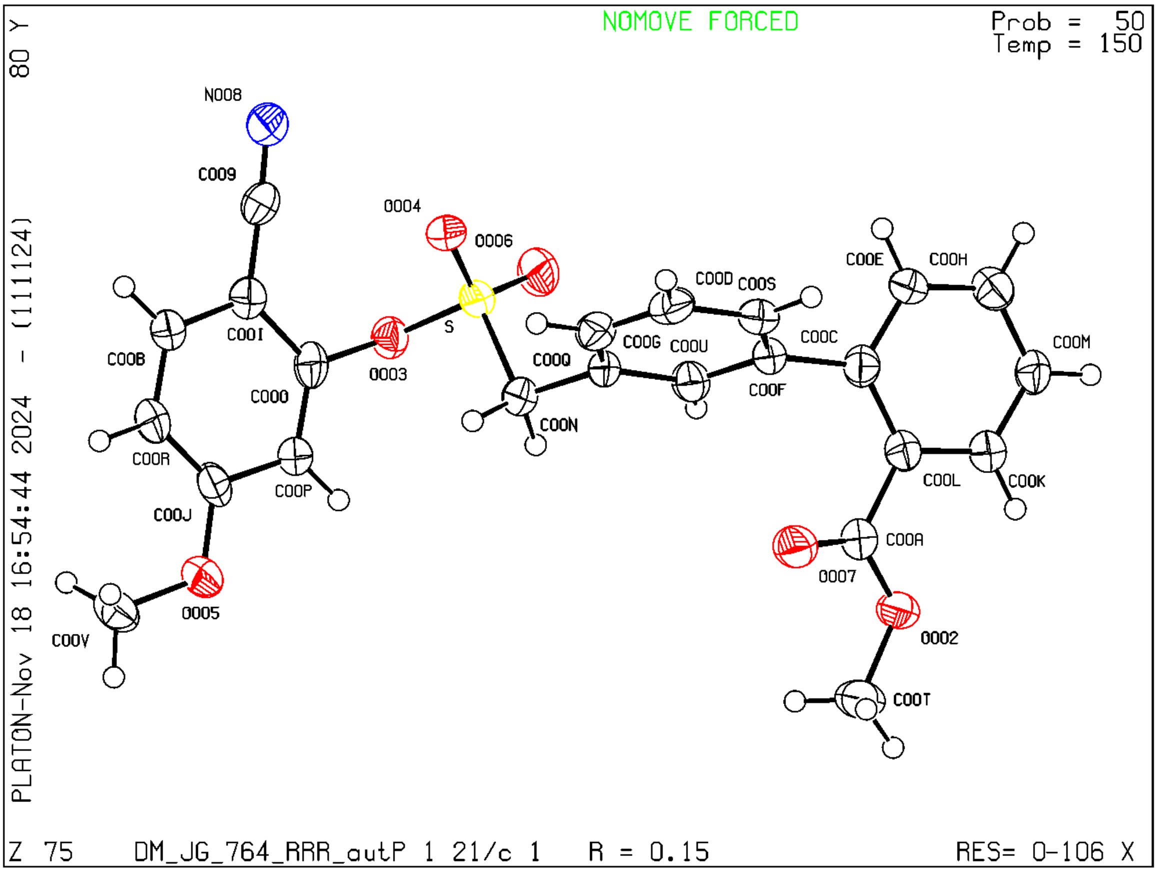

Supplement: Supplementary file 2 — Supporting File 2: advs73459‐sup‐0002‐DataFile.zip. [file ADVS-13-e19731-s002.zip › checkcif.pdf]
